# Comprehensive analysis of scRNA-Seq and bulk RNA-Seq reveals dynamic changes in the tumor immune microenvironment of bladder cancer and establishes a prognostic model

**DOI:** 10.1186/s12967-023-04056-z

**Published:** 2023-03-27

**Authors:** Zhiyong Tan, Xiaorong Chen, Jieming Zuo, Shi Fu, Haifeng Wang, Jiansong Wang

**Affiliations:** 1 Department of Urology, The Second Affiliated Hospital of Kunming Medical University, Yunnan Institute of Urology, No. 347, Dianmian Street, Wuhua District, Kunming, 650101 Yunnan People’s Republic of China; 2 Urological Disease Clinical Medical Center of Yunnan Province, The Second Affiliated Hospital of Kunming Medical University, No. 347, Dianmian Street, Wuhua District, Kunming, 650101 Yunnan People’s Republic of China; 3 Scientific and Technological Innovation Team of Basic and Clinical Research of Bladder Cancer in Yunnan Universities, The Second Affiliated Hospital of Kunming Medical University, No. 347, Dianmian Street, Wuhua District, Kunming, 650101 Yunnan People’s Republic of China; 4 Department of Urology, The First Hospital of Suzhou University, No.188, Shi Zi Street, Suzhou, 215006 Jiangsu People’s Republic of China; 5 Department of Urology, The Second People’s Hospital of Baoshan City, No. 13, Zhengyang South Road, Longyang District, Yunnan, People’s Republic of China

**Keywords:** Bladder cancer, scRNA-seq, Bulk RNA-seq, Prognosis, Immune landscape

## Abstract

**Background:**

The prognostic management of bladder cancer (BLCA) remains a great challenge for clinicians. Recently, bulk RNA-seq sequencing data have been used as a prognostic marker for many cancers but do not accurately detect core cellular and molecular functions in tumor cells. In the current study, bulk RNA-seq and single-cell RNA sequencing (scRNA-seq) data were combined to construct a prognostic model of BLCA.

**Methods:**

BLCA scRNA-seq data were downloaded from Gene Expression Omnibus (GEO) database. Bulk RNA-seq data were obtained from the UCSC Xena. The R package "Seurat" was used for scRNA-seq data processing, and the uniform manifold approximation and projection (UMAP) were utilized for downscaling and cluster identification. The FindAllMarkers function was used to identify marker genes for each cluster. The limma package was used to obtain differentially expressed genes (DEGs) affecting overall survival (OS) in BLCA patients. Weighted gene correlation network analysis (WGCNA) was used to identify BLCA key modules. The intersection of marker genes of core cells and genes of BLCA key modules and DEGs was used to construct a prognostic model by univariate Cox and Least Absolute Shrinkage and Selection Operator (LASSO) analyses. Differences in clinicopathological characteristics, immune microenvironment, immune checkpoints, and chemotherapeutic drug sensitivity between the high and low-risk groups were also investigated.

**Results:**

scRNA-seq data were analyzed to identify 19 cell subpopulations and 7 core cell types. The ssGSEA showed that all 7 core cell types were significantly downregulated in tumor samples of BLCA. We identified 474 marker genes from the scRNA-seq dataset, 1556 DEGs from the Bulk RNA-seq dataset, and 2334 genes associated with a key module identified by WGCNA. After performing intersection, univariate Cox, and LASSO analysis, we obtained a prognostic model based on the expression levels of 3 signature genes, namely MAP1B, PCOLCE2, and ELN. The feasibility of the model was validated by an internal training set and two external validation sets. Moreover, patients with high-risk scores are predisposed to experience poor OS, a larger prevalence of stage III-IV, a greater TMB, a higher infiltration of immune cells, and a lesser likelihood of responding favorably to immunotherapy.

**Conclusion:**

By integrating scRNA-seq and bulk RNA-seq data, we constructed a novel prognostic model to predict the survival of BLCA patients. The risk score is a promising independent prognostic factor that is closely correlated with the immune microenvironment and clinicopathological characteristics.

**Supplementary Information:**

The online version contains supplementary material available at 10.1186/s12967-023-04056-z.

## Introduction

Bladder cancer (BLCA), referred to as urothelial carcinoma, is one of the most common incident urological malignancies with more than 90% originating in the uroepithelium. It is estimated that more than 550,000 new cases are diagnosed and more than 200,000 deaths per year [1]. It has become the fourth and tenth most common malignancy among men and women, respectively [2]. There are two main types of BLCA pathologically, non-muscle-invasive bladder cancer (NMIBC) and muscle-invasive bladder cancer (MIBC). Approximately 75% of patients initially present with NMIBC while the remaining 25% are diagnosed with MIBC [3]. The evidence-based guideline recommends radical cystectomy with pelvic lymphadenectomy as the mainstay of treatment for patients with high-risk NMIBC or MIBC [4]. Although patients receive aggressive treatment including surgery, immunotherapy, chemotherapy, and radiotherapy, the 5-year overall survival (OS) rate remains unsatisfactory, with a median OS of approximately 14 months [5]. Reasons for this poor prognosis include delay in diagnosis and the lack of effective therapy. But most importantly, the unsatisfactory prognosis was closely related to the aggressive and highly proliferative capacity of cancer cells, as well as the heterogeneity of disease characteristics. Therefore, there is a compelling urge to uncover the molecular mechanisms involved in tumourigenesis and thereby explore novel potential molecular biomarkers, which are essential for the early diagnosis, targeted therapy, and prognostic assessment of BLCA patients.

With the rapid development of cancer genomics in recent decades, bulk transcriptome sequencing (bulk RNA-seq) has become a major tool for transcriptomics, and more and more gene alteration has been identified as an effective treatment target for BLCA [6]. For instance, Xie et al. found exonic circular circPTPRA could inhibit cancer progression through endogenous blocking of the recognition of IGF2BP1 to m6A-modified RNAs [7]. In addition, Yang et al. indicated that exosome-derived circTRPS1 could modulate the intracellular reactive oxygen species balance and CD8^+^ T cell exhaustion via the circTRPS1/miR141-3p/GLS1 axis in BLCA [8]. However, in contrast to bulk RNA-seq or microarray experiments, which probe average gene expression in cell populations. Currently, single-cell RNA-seq (scRNA-seq) elucidates information about cellular transcriptomic heterogeneity, allowing us to access underlying gene expression distributions [9, 10]. Using scRNA-seq, we can develop personalized therapeutic strategies that are potentially useful in cancer diagnosis and therapy resistance during cancer progression [11]. Xu et al. integrative analyses of scRNA-seq and scATAC-seq revealed CXCL14 as a key regulator of lymph node metastasis in breast cancer, which improves our understanding of the mechanism of tumor metastasis [12]. Besides, Obradovic A et al. demonstrated that HNCAF-0/3 could reduce TGFβ-dependent PD-1^+^TIM-3^+^ exhaustion of CD8^+^ T cells, increase CD103^+^NKG2A^+^ resident memory phenotypes, and enhance the overall cytolytic profile of T cells [13]. Given this advantage, numerous studies have focused on identifying potential biomarkers for BLCA by integrating bulk RNA-seq and scRNA-seq analysis, which could precisely stratify and recognize patients.

In this study, we performed systematic bioinformatics analyses using scRNA-seq and bulk RNA-seq data to construct a prognostic model of BLCA patients, with two external validation cohorts to validate its ability to stratify risk. Meanwhile, we outline the immune infiltration landscape and determine how it contributes to the development of BLCA. Moreover, we deliberated the relationship between the risk model and infiltrating immune cells to gain a better understanding of the potential molecular immunity process during the progression of BLCA. Overall, our study provides a novel insight that may benefit the clinical management of BLCA.

## Materials and methods

### Data sources and processing

Bulk RNA-seq data, clinical information, and SNP mutation site data of TCGA-BLCA were downloaded from the TCGA database (https://portal.gdc.cancer.gov/), containing 19 normal tissue samples and 411 BLCA samples. Samples with incomplete survival information and clinical information were excluded to obtain a training set of 406 BLCA patients for this study. The scRNA-seq dataset GSE129845 of BLCA was downloaded from the GEO (https://www.ncbi.nlm.nih.gov/) database, containing scRNA-seq of paracancerous tissues from 3 BLCA patients, the patients information and sequencing statistics were shown in Additional file 9: Table S1. GSE13507, containing 165 BLCA patients with complete survival information, was also downloaded as external data to validate the model feasibility. The samples were integrate using anchors method in the R package "Seurat" [14] and core cells were obtained by filtering scRNA-seq. Ineligible cells include genes that can only be detected in 3 or fewer cells and low-quality cells with less than 200 genes detected will be excluded from subsequent analysis. Gene expression of core cells was normalized using a linear regression model, and then the top 2000 genes with highly variable characteristics were screened by ANOVA. Principal component analysis (PCA) was performed on single-cell samples, and the top 20 principal components (PC) were selected for subsequent analysis. The umap algorithm [15] was used to perform an overall dimensionality reduction analysis on the top 20 PC pairs of samples. Using the R package "singleR" package [16], HumanPrimaryCellAtlasData, BlueprintEncodeData, and ImmuneCellExpressionData were used as reference data for auxiliary annotation, followed by the CellMarker database [17] and previous studies to find marker genes for manual annotation of different clusters.

### Screening of core cells and functional enrichment analysis of their marker genes

The FindAllMarkers function in the Seurat package was used to find marker genes for each cluster by setting the parameters min.pct = 0.2 and only.pos = TRUE, and the Wilcoxon rank sum test was used to identify DEGs in the process of screening marker genes. Based on the significantly different marker genes for each cell type, ssGSEA [18] scores were calculated for each cell type in the TCGA dataset (BLCA/normal) using ssGSEA, and the differences in scores between BLCA and normal samples for each cell type were analyzed by Wilcoxon, and cells with significant differences (p < 0.05) in the control and normal groups were recorded as core cells. The marker genes of core cells were enriched for GO and KEGG functions using the "clusterProfiler" [19] in R software, respectively. To explain the molecular mechanism of BLCA progression, pseudo-temporal analysis was performed on each of the seven cells using the Monocle 2 algorithm. CellPhone DB v2.0 was used to explore the potential interactions between core cells.

### Identification and functional enrichment analysis of DEGs in TCGA-BLCA

Differential analysis was performed on 19 control and 411 disease data using the limma package. p_value < 0.05 and |Log2FC|> 1 were designated as DEGs. The heatmap and volcano maps of DEGs were visualized using the ggplot2 [20] and pheatmap packages [21], respectively. Subsequently, the most significant enrichment pathways and biological processes of DEGs were investigated using the Kyoto Encyclopedia of Genes and Genomes (KEGG) and Gene Ontology (GO) analyses using the R software "clusterProfiler" package.

### WGCNA analysis

In the training set, the genes associated with BLCA are filtered using the R package WGCNA [22]. First, the goodSamplesGenes function of the R package "WGCNA" is used to check whether the genes of the samples need to be filtered and to select a suitable soft threshold. Then, the co-expression network was constructed by setting the minimum number of genes per gene module to 300 according to the criteria of the hybrid dynamic tree-cutting algorithm. Finally, Pearson correlation coefficients were used to analyze the association of module signature genes (ME) with BLCA.

### Construction and validation of a prognostic model

The mark genes of core cells and BLCA-related genes and DEGs were taken as the intersection set, and the obtained genes were defined as candidate genes. The 406 samples in the TCGA-BLCA dataset were divided into training and validation sets in the ratio of 7:3, with 7 as the training set (285 cases) and 3 as the validation set (121 cases). Univariate Cox proportional risk regression analysis was performed on candidate genes in the training set to screen the characteristic genes associated with prognosis. Variables with p-values < 0.05 were included in the least absolute shrinkage and selection operator (LASSO) regression analysis, which was performed with the R software "glmnet" package [23] to reduce the number of genes in the final risk model. The prognostic model was constructed according to the formula: risk score = gene exp1 × β1 + gene exp2 × β2 + … + gene expression n × βn (gene expression denotes the gene expression value and β denotes the corresponding LASSO regression coefficient). Patients' survival curves and risk maps were visualized by the R software, "survminer" and "ggrisk" packages. The ROC curves were plotted using the "survROC" package [24] to assess the performance of risk scores in predicting OS at 1, 2, 3, 4, and 5 years in BLCA patients. In addition, the validity of the prognostic model was verified by the internal validation set and external datasets GSE13507 and GSE32548.

### Analysis of subtype clinical characteristics

Samples with multiple clinicopathological characteristics were classified into the following subtypes, including age (> 60 and ≤ 60), sex, M stage, T stage, TME, stage, and OS status. Within each subtype, cancer samples were divided into two risk groups (high and low). The distribution of clinicopathological characteristics among subtypes was assessed using the Kruskal–Wallis test or the Wilcoxon rank test. To more closely understand the correlation between clinicopathological characteristics and survival, a stratified survival analysis of clinical factors was performed for high and low-risk groups.

### Independent prognostic analysis

Univariate analysis was used to assess the risk model and clinical parameters (age, T, M, N stage, state, RiskScore) for each predictive value, whereas multivariate Cox analysis for OS was used to identify independent risk factors. To predict the overall survival of BLCA. Based on the independent prognostic factors screened by multivariate Cox independent prognosis, the nomogram model was drawn using the "cph" function in R to visualize this prediction model and to predict the likely 1, 3, and 5-year survival plots of patients. Calibration curves were used to verify the validity of the bar graphs.

### GSEA enrichment analysis

GSEA enrichment analysis was performed using the clusterProfiler package for all genes in samples from the high and low-risk groups in TCGA to explore the differences in function and associated pathways between the high and low-risk groups. A set of 50 human cancer marker pathway genes was downloaded from the Molecular Signature Database (MSigDB) (http://www.gsea-msigdb.org/gsea/index.jsp), and GSVA enrichment analysis was performed on all genes from samples in the high- and low-risk groups and the differences in GSVA [25] scores between high- and low-risk samples were analyzed using the limma package.

### Immune microenvironment analysis

To analyze the immune cell characteristics between different risk groups, we used the ssGSEA based on R package gsva to obtain 28 immune cell infiltration statuses for each sample in TCGA-BLCA. The correlation between risk score and immune infiltrating cells was analyzed by the Pearson correlation coefficient. In parallel, T-cell inflammatory GEP (18 inflammatory genes) associated with ICB response were introduced to assess the predictive potential of the risk score for cancer immunotherapy. We also performed GO enrichment analysis of GEPs and used Cytoscape to plot the top 4 pathways with the highest enrichment significance with gene interaction network regulatory map and PPI network. Finally, we also extracted the expression levels of four immune check loci (PD-1, PD-L1, CTLA-4, and TIGIT) in BLCA and assessed their expression differences in high and low-risk groups using the Wilcoxon test. Differences in mutations between high and low-risk groups were analyzed using the "maftools" R package [26].

### Chemotherapy drug sensitivity analysis

To further explore the potential guidance of risk scores for chemotherapy. In this study, the IC50 values of drugs were obtained in Genomics of Drug Sensitivity in Cancer (GDSC) and Cancer Therapeutics Response Portal (CTRP) using the R package oncoPredict. The correlation between drug IC50 values and risk scores was analyzed by Spearman's analysis to screen drugs. We then compared the differences in IC50 between the high and low-risk groups for drugs with absolute values of correlation greater than 0.4. The results were then visualized by plotting box plots and lollipop plots using the R language ggplot2.

## Results

### Identification of BLCA cell subtypes

**Fig. 1:**
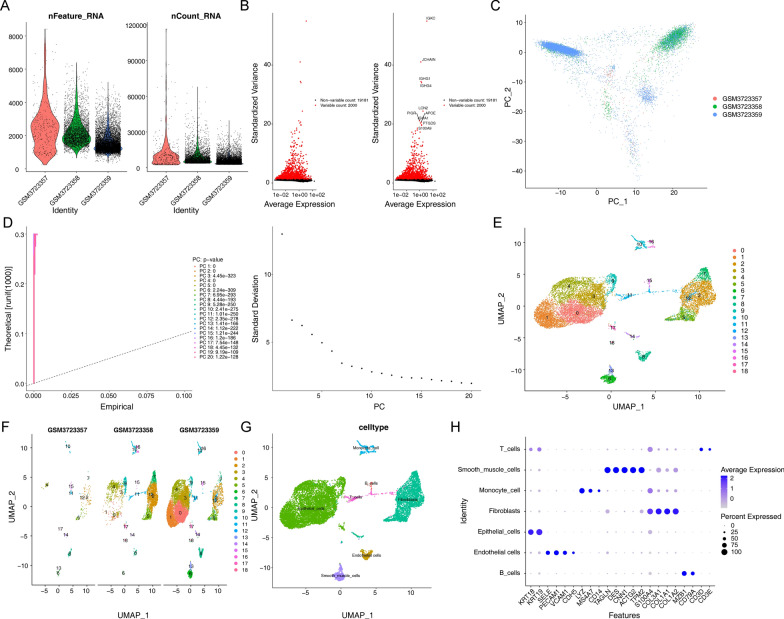
Identification of 7 cell clusters with diverse annotations revealing high cellular heterogeneity in BLCA tumors based on single-cell RNA-seq data. **A** After quality control of scRNA-seq, 13,490 core cells were identified. **B** The variance diagram shows the variation of gene expression in all cells of BLCA. The red dots represent highly variable genes and the black dots represent non-variable genes. **C** PCA showed a clear separation of cells in BLCA. **D** PCA identified the top 20 PCs at P < 0.05. **E** The umap algorithm was applied to the top 20 PCs for dimensionality reduction, and 19 cell clusters were successfully classified. **F** Classification of cell clusters in each sample. **G** All 7 cell clusters in BLCA were annotated with singleR and CellMarker according to the composition of marker genes. **H** Expression levels of marker genes for each cell cluster

The overall schematic outline of the present study was shown in Additional file 1: Fig. S1. First, we filtered ineligible cells and yielded 13,490 core cells for subsequent analysis (Fig. 1A). ANOVA of genes was performed on the core cells, and we found that 2000 genes were highly variable (Fig. 1B). PCA was performed on three single-cell samples (Fig. 1C), and the single-cell samples were scattered and distributed with logical results. Meanwhile, in the PCA, we also selected 20 principal components (PCs) with p.value < 0.05 for subsequent analysis (Fig. 1D). Then, the core cells were classified into 19 independent cell clusters using the umap algorithm (Fig. 1E, F). The different clusters were annotated by finding marker genes through the " singleR " package, CellMarker database, and references [17], resulting in seven cell clusters, namely B cells, endothelial cells, T cells, monocyte cell, fibroblasts, smooth muscle cells, and epithelial cells (Fig. 1G). The expression of important marker genes for each cell type was visualized by bubble plots (Fig. 1H). The scatter plots showed the expression of marker genes in different cell types (Additional file 2: Fig. S2). Furthermore, we exprlored the expression of marker genes (PDPN, THY1, PDGFRB, PDGFRA,and POSTN) for cancer-associated fibroblasts (CAFs) in different cell types, and found that all marker genes were highly expressed in fibroblasts (Additional file 3: Fig. S3). The high expression of each identified marker gene in a specific cell could be summarized, further illustrating the reliability of cell type determination.

### Identification of core cells and their marker gene functional enrichment analysis

**Fig. 2:**
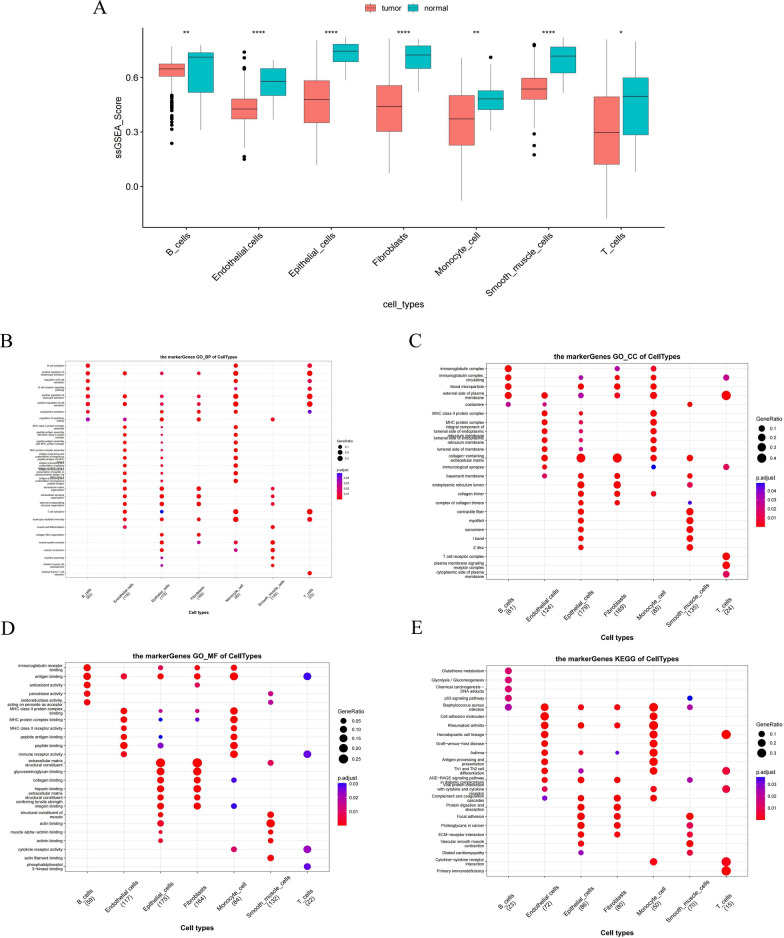
Functional enrichment analysis of marker genes based on 7 key cells. **A** Differentially expressed cells in BLCA and control samples were obtained by calculating the ssGSEA score of each cluster based on the marker genes. **B**–**E** Based on the marker genes of differentially expressed cells, ClusterProfiler package for GO and KEGG functional enrichment

**Fig. 3:**
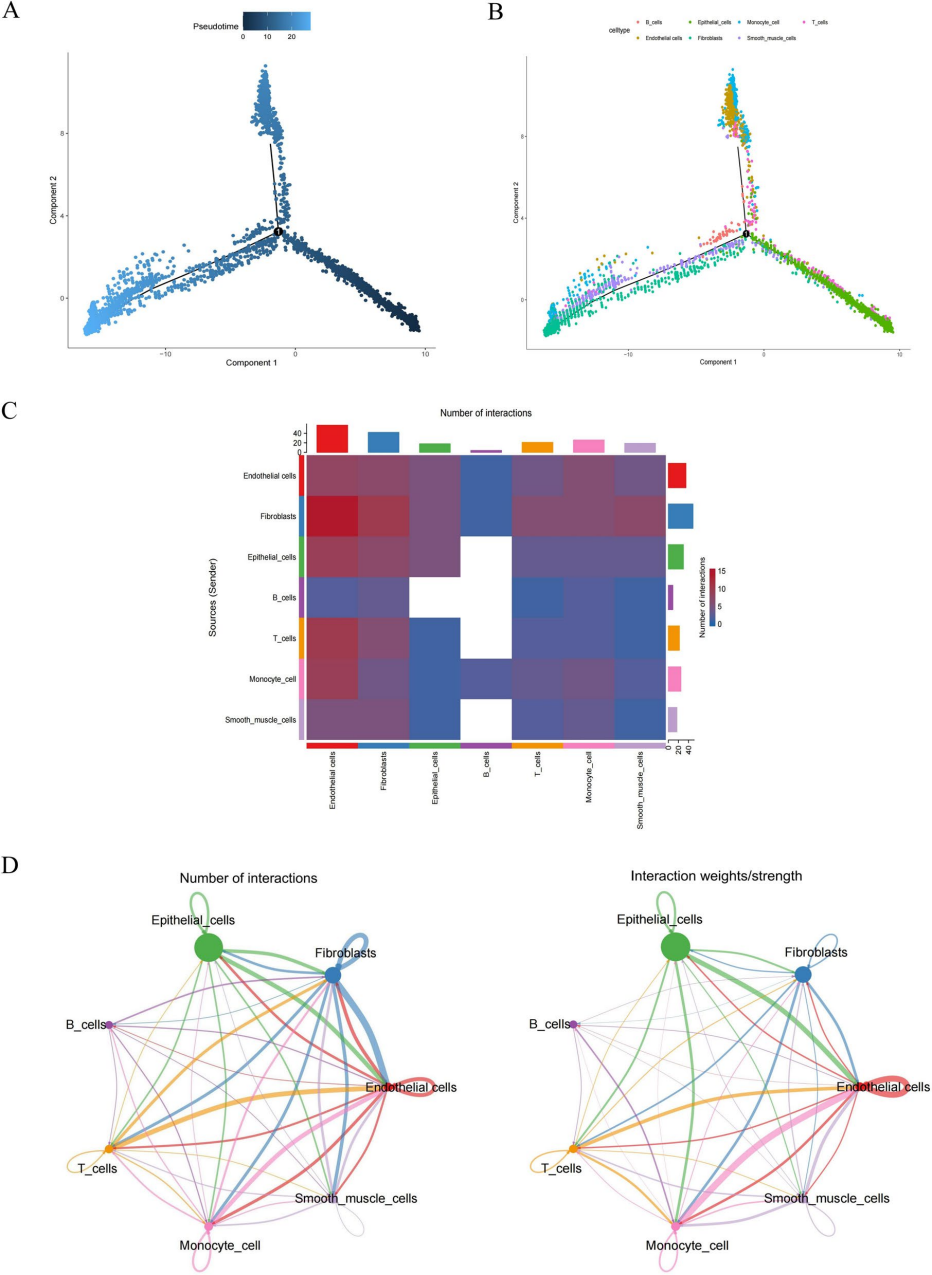
Trajectory and cell–cell communication analysis of three BLCA cell subsets with distinct differentiation patterns. **A**, **B** Trajectory analysis revealed three subsets of BLCA cells with distinct differentiation patterns. One of them differentiates into a branch dominated by Endothelial cells, and the other branch is dominated by smooth muscle cells, Fibroblasts cells. **C** Heatmap visualizes the number of potential ligand-receptor pairs in key cells. **D** Number and strength of interactions between key cells

By using the FindAllMarkers and Wilcoxon test, 474 significantly different marker genes were obtained to be identified (Additional file 10: Table S2). Calculating the ssGSEA scores of significantly different marker genes for each cell, we found that all seven cells were significantly down-regulated in BLCA, and therefore seven cells were considered core cells for subsequent analysis (Fig. 2A). The marker genes of core cells were enriched for GO and KEGG functions (Fig. 2B–E). We noted that, with the exception of smooth muscle cells, the marker genes for all six cell types were associated with positive regulation of cell activation, including lymphocytes and leukocytes (Fig. 2B). In addition, the marker genes of monocytes and T cells were associated with cytokine-cytokine receptor interactions. The marker genes of B cells were connected to the p53 signalling pathway. The marker genes of endothelial cells, epithelial cells and smooth muscle cells were linked to focal adhesion and ECM-receptor interaction (Fig. 2E). Pseudo-temporal analysis was performed separately for all cells annotated to explore their differentiation directions using the Monocle 2 algorithm. The results showed that BLCA cells gradually followed 3 differentiation directions (Fig. 3A). Epithelial cells differentiated earlier than other cells and differentiated into two branches, one of which was dominated by Endothelial cells and the other by smooth muscle cells, fibroblasts cells (Fig. 3B). Furthermore, we inferred cell–cell communication networks to predict intercellular communication based on specific pathways and ligand receptors. The heatmap of the number of ligand-receptor pairs showed that Fibroblasts, T cells, monocyte cell, endothelial cells, and Epithelial cells cellular communication occurred more frequently (Fig. 3C). In detail, the frequency and intensity of interactions between endothelial cells and epithelial cells, between endothelial cells and fibroblasts, and between endothelial cells and T cells were high (Fig. 3D). In addition, the interactions of B cells with other cells were relatively rare (Additional file 4: Fig. S4).

### Identification and functional enrichment analysis of DEGs in Bulk RNA-seq data

**Fig. 4:**
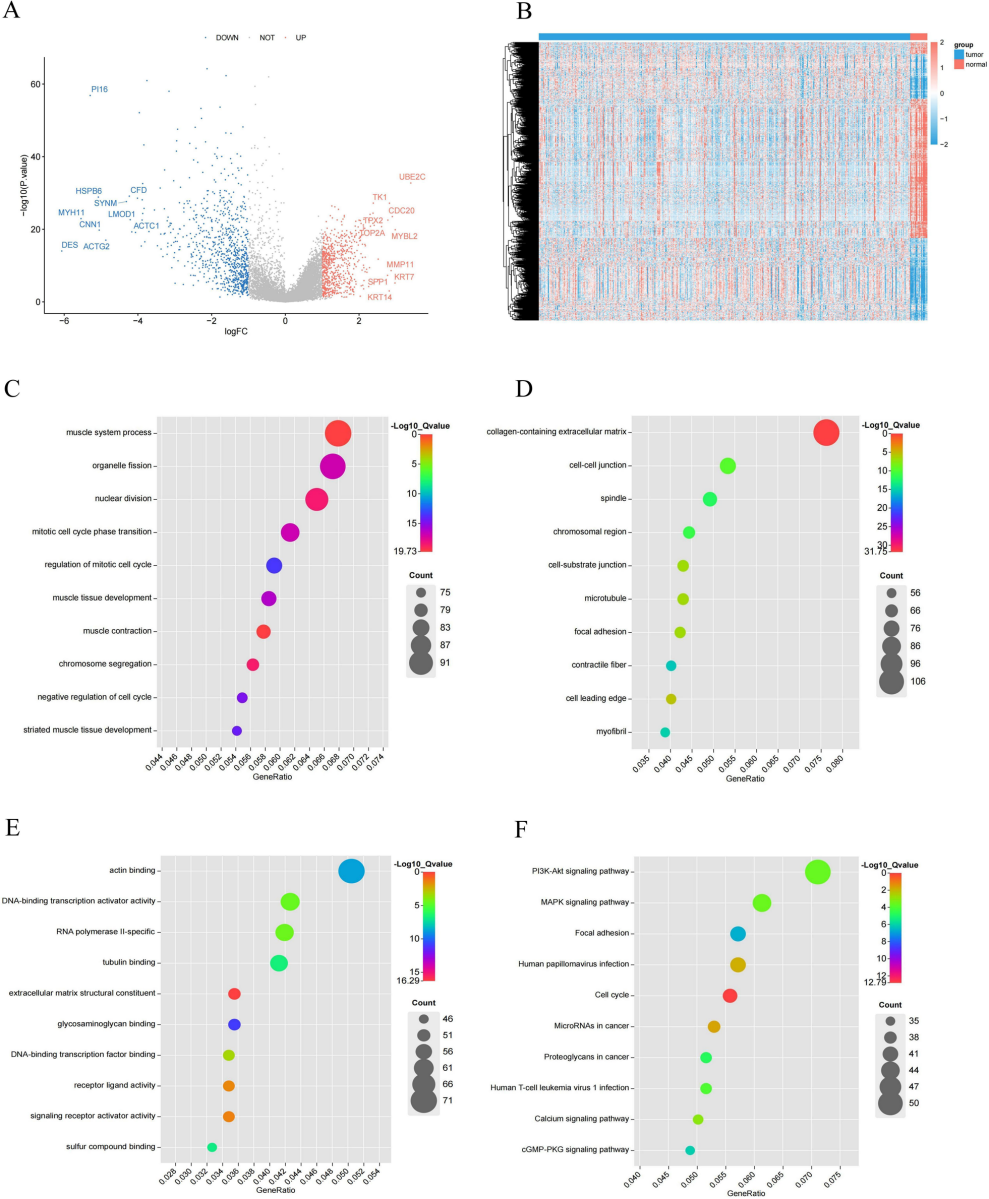
Identification and functional enrichment analysis of DEGs between BLCA patients and controls. **A** Volcano plot of DEGs between BLCA and control in TCGA. P < 0.05 and |log2FoldChange|> 1 were identified as significant DEGs. The red dots represent upregulated genes and the blue dots represent downregulated genes. **B** Heatmap of DEGs. **C–F** Bubble plots of the BP, CC, MF, and KEGG pathways of DEGs

A total of 1556 significant DEGs were obtained, including 708 up-regulated genes and 848 down-regulated genes (Fig. 4A, B). The GO analysis showed that DEGs were mainly enriched in the nuclear division, organelle fission, mitotic nuclear division, and other cell cycle-related functions (Fig. 4C–E). The KEGG enrichment results showed that the PI3K-Akt signaling pathway, MAPK signaling pathway, adherent spots, and cell cycle were the enriched pathways for DEGs (Fig. 4F).

### Identification of BLCA-related key modules

**Fig. 5:**
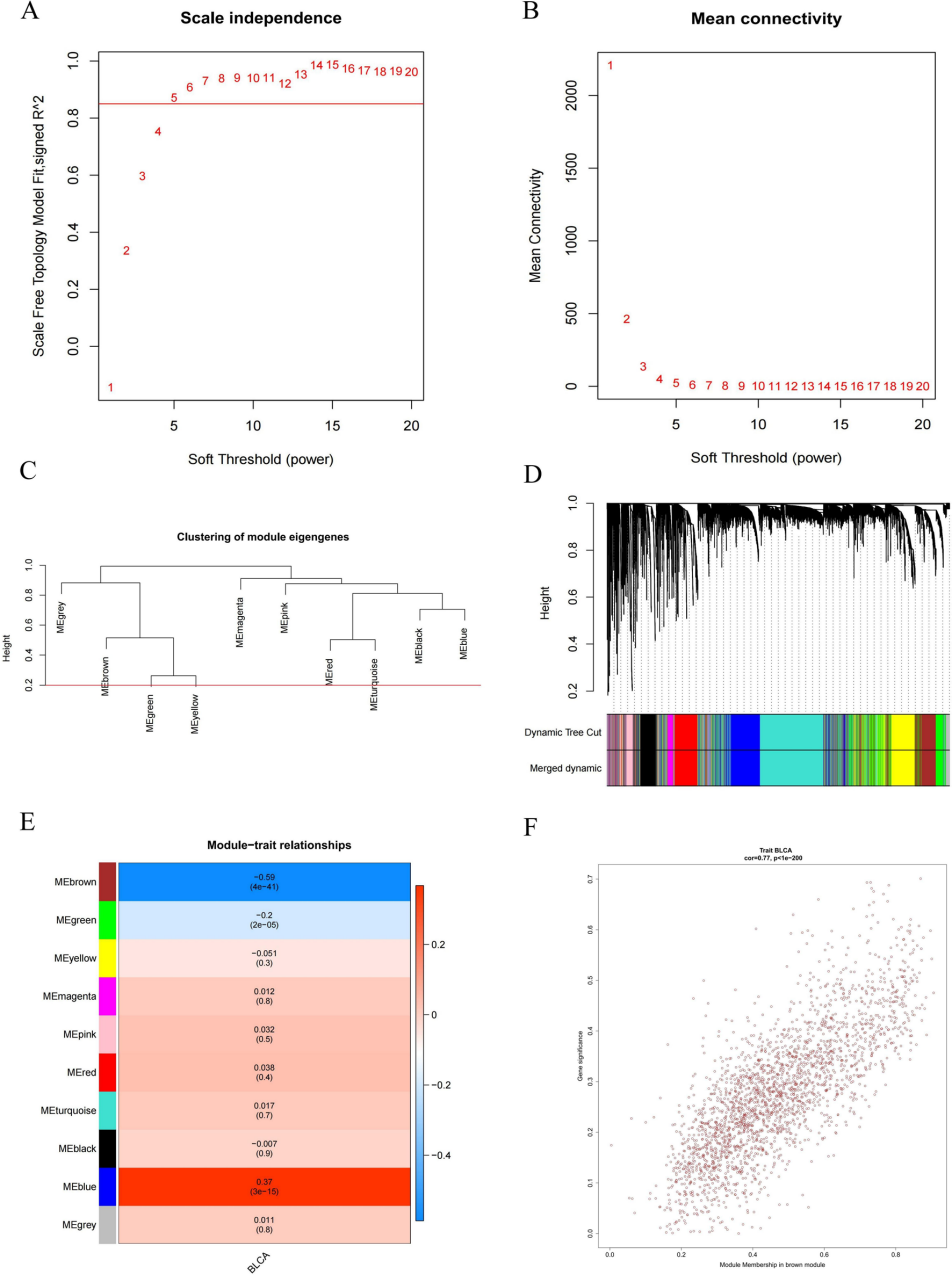
BLCA-related genes were screened by WGCNA. **A**, **B** Analysis of the scale-free index for various soft-threshold powers (β). **C** The minimum number of genes per module is 300, and 10 modules are obtained when MEDissThres is equal to 0.2. **D** Cluster dendrogram of the co-expression network modules (1-TOM). **E** Analysis of correlations between the modules and BLCA, p.values are shown. **F** Scatter plot analysis of the brown module

WGCNA was used to identify genes involved in the development and progression of BLCA. During the construction of the co-expression network, the soft threshold power β was 5 when the fit index of the scale-free topology reached 0.85 (Fig. 5A, B). MEDissThres was set to 0.2 to merge similar modules analyzed by the dynamic shear tree algorithm, and after merging, a total of 10 modules were finally available (Fig. 5C, D). Based on the correlation coefficient and P value, we selected MEbrown as the key module (containing 2334 genes) (Fig. 5E). The key module genes are detailed in Additional file 11: Table S3 as shown in Fig. 5F, the scatter plot of the brown module with clinical correlation.

### Construction and validation of a 3 characteristic gene-based prognostic model

**Fig. 6:**
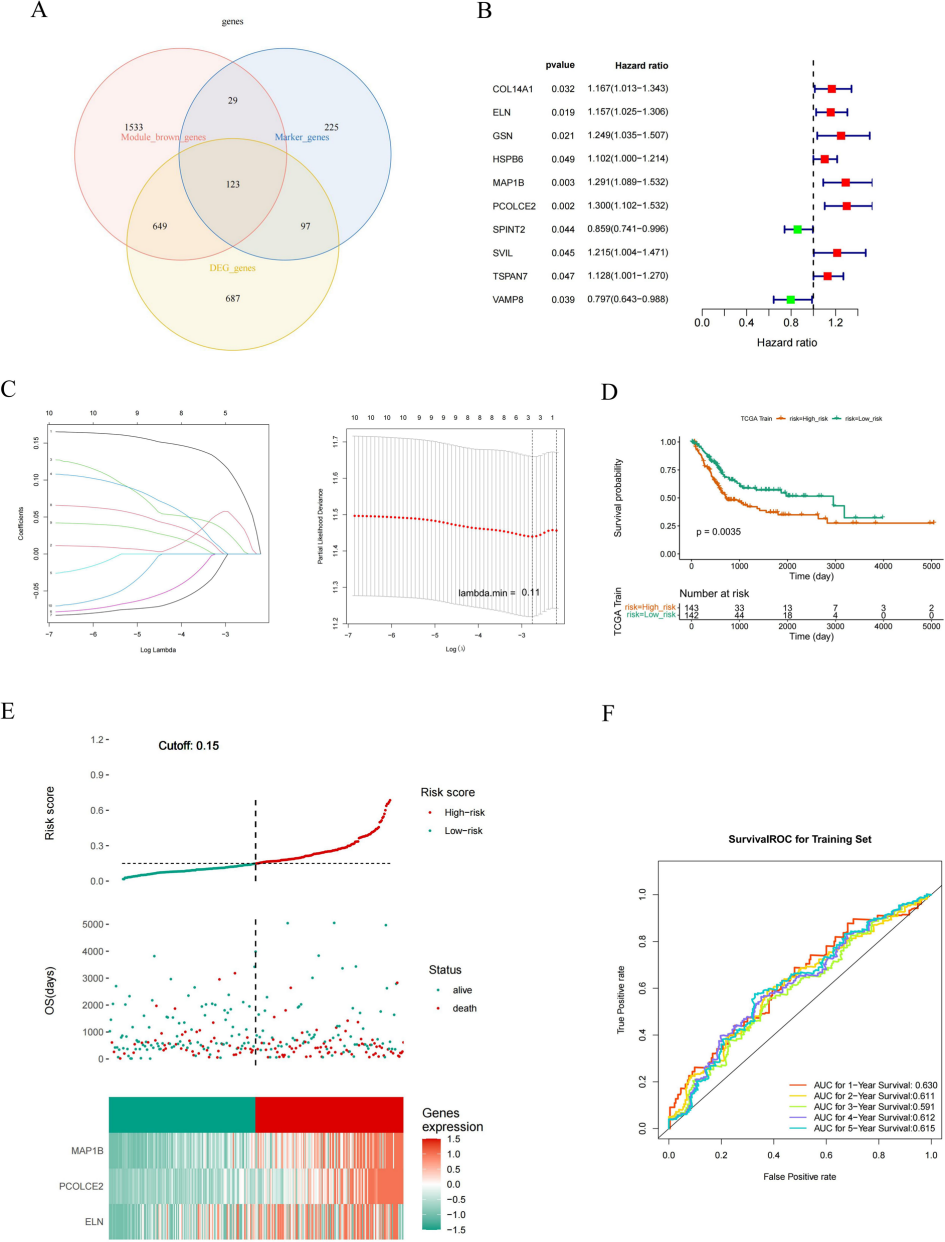
Construction of risk signature in the TCGA cohort. **A** Intersection of BLCA-related genes, DEGs in key cells, and DEGs in BLCA and controls. **B** Univariate cox regression analysis of OS. **C** LASSO regression of OS-related genes. **D** Kaplan–Meier curve result. **E** Risk survival status plot. **F** The AUC of the prediction of 1, 3, and 5-year survival rates of BLCA

The intersection of marker genes, BLCA module genes, and DEGs of cell subtypes was demonstrated using Ven plots, and a total of 123 intersecting genes were taken and defined as candidate genes (Fig. 6A). Then, univariate Cox regression analysis was performed using the training set in TCGA-BLCA, and 10 genes were significantly associated with OS (Fig. 6B). Next, genes were screened for model construction using the LASSO algorithm. The results are shown in Fig. 6C. 3 characteristic genes were screened at the lowest cross-validation error: PCOLCE2, MAP1B, and ELN. Risk score = 0.09876179 × PCOLCE2 + 0.04635731 × MAP1B + 0.01686333 × ELN. According to cut-off = 0.15, patients were divided into high- and low-risk groups (Fig. 6D). The Kaplan–Meier analysis showed that patients with high-risk scores had significantly lower OS and disease-free survival (DFS) than those with low-risk scores (Fig. 6E; Additional file 5: Fig. S5A). To further assess the validity of the risk model, the ROC curve for OS was calculated, and the AUC values at 1, 2, 3, 4, and 5 years were greater than 0.59, indicating better efficacy of the risk model (Fig. 6F). We also performed functional validation of the model in the internal validation set and external validation set GSE13507 and GSE32548, and the results showed that the model has an accuracy (Additional file 6: Fig. S6, Additional file 5: Fig. S5B). In summary, our prognostic model showed excellent predictive efficiency in BLCA.

### Analysis of risk scores and different clinical characteristics

**Fig. 7:**
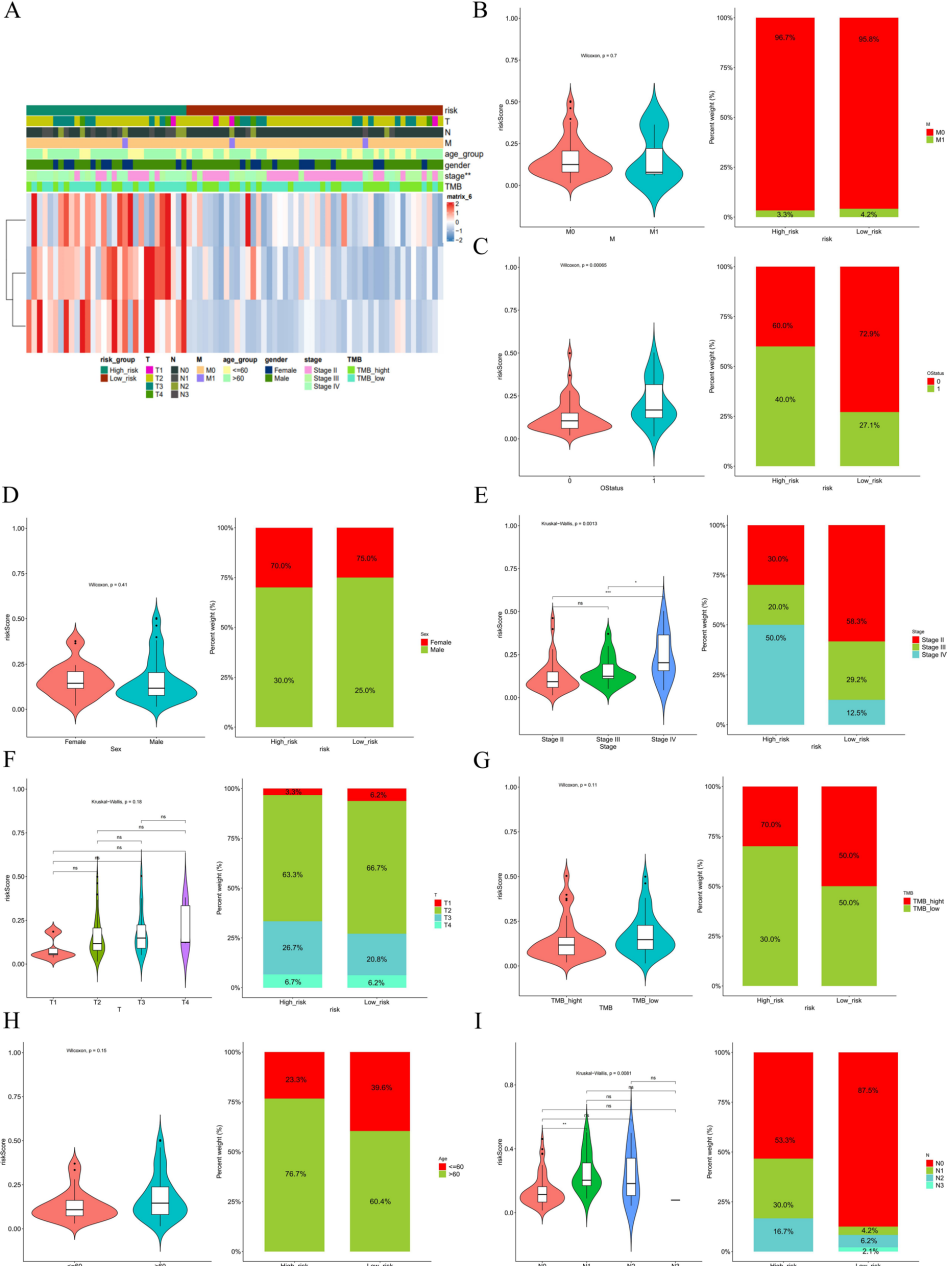
Correlation analysis of risk scores with clinical characteristics. **A** Heatmap of risk model and clinical characteristics. **B**–**I** Relationship between age, sex, M stage, N stage, T stage, TMB, tumor stage, and survival status with the analysis model

To analyze the correlation between the expression of risk scores and clinical characteristics, the differences in risk scores of patients were compared separately according to different groups of clinical characteristics. The results showed that risk scores were significantly different in N-stage, T-stage, and OS status (Fig. 7B). The heatmap of the risk model and clinical characteristics are shown in Fig. 7A. Stratified analysis of clinical characteristics showed that clinical stages M0, Male, Stage III-IV, T1-T2, age > 60, and TMB_hight were significantly different in survival in high and low-risk groups (Additional file 7: Fig. S7). Taken together, our prognostic model based on three characteristic genes had excellent prognostic value.

### Screening of independent prognostic factors and construction of nomogram

**Fig. 8:**
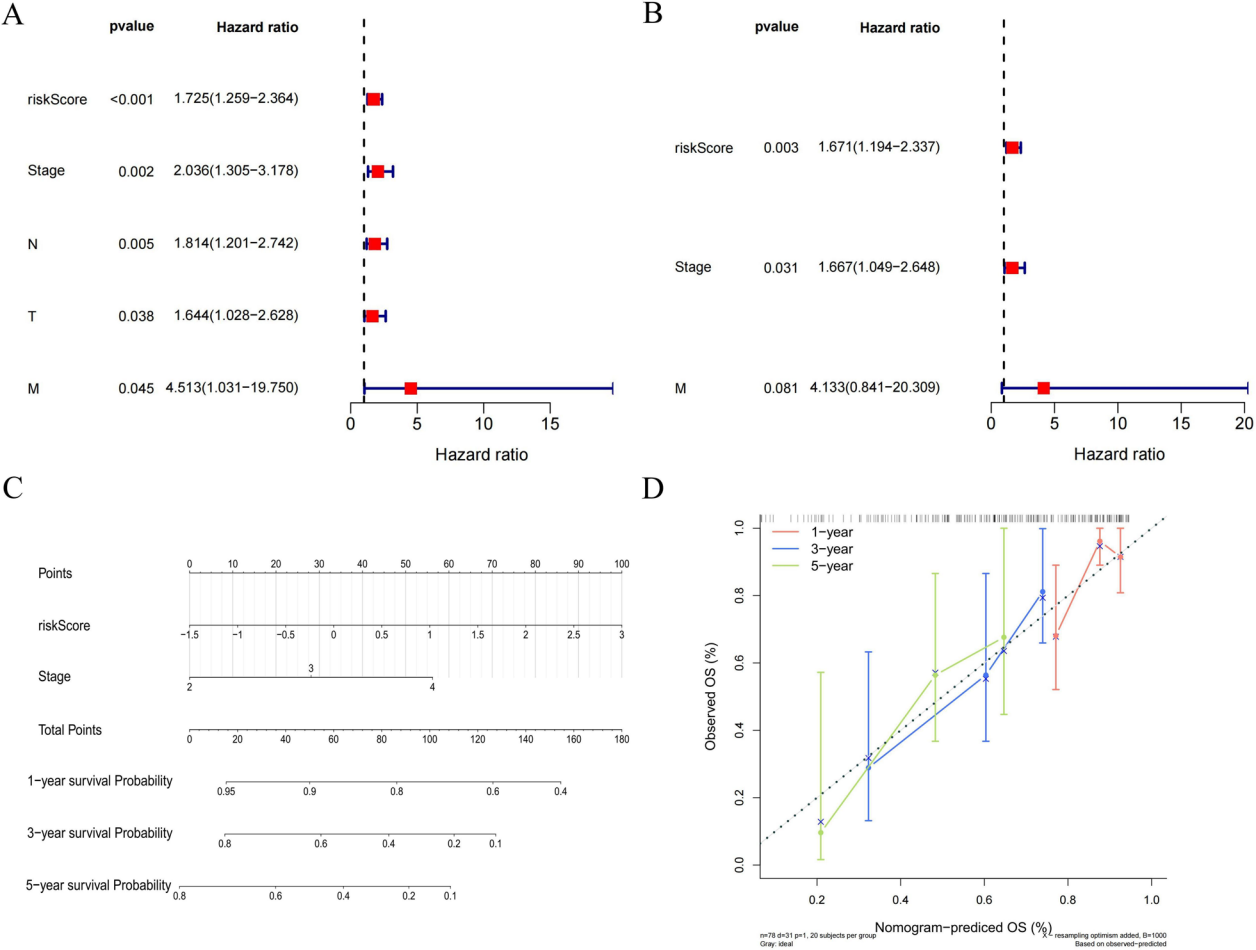
The nomogram model was constructed based on Univariate and multivariate cox regression analyses. **A** Univariate Cox analysis of risk scores and clinical characteristics. **B** Multifactorial Cox analysis. **C** Construction of the nomogram model. **D** The calibration curve of the nomogram

To screen independent prognostic factors, clinical characteristics and risk scores were subjected to univariate and multivariate Cox analyses. We found RiskScore, and Stage as independent prognostic factors for patients (Fig. 8A, B). The two independent prognostic factors were included in the nomogram model (Fig. 8C). In addition, the calibration curve showed that the model had a high predictive effect (Fig. 8D). Therefore, our results suggested that risk score was an independent prognostic factor and that the nomogram had high predictive efficacy for predicting OS of BLCA patients.

### GSEA between high- and low-risk groups

**Fig. 9:**
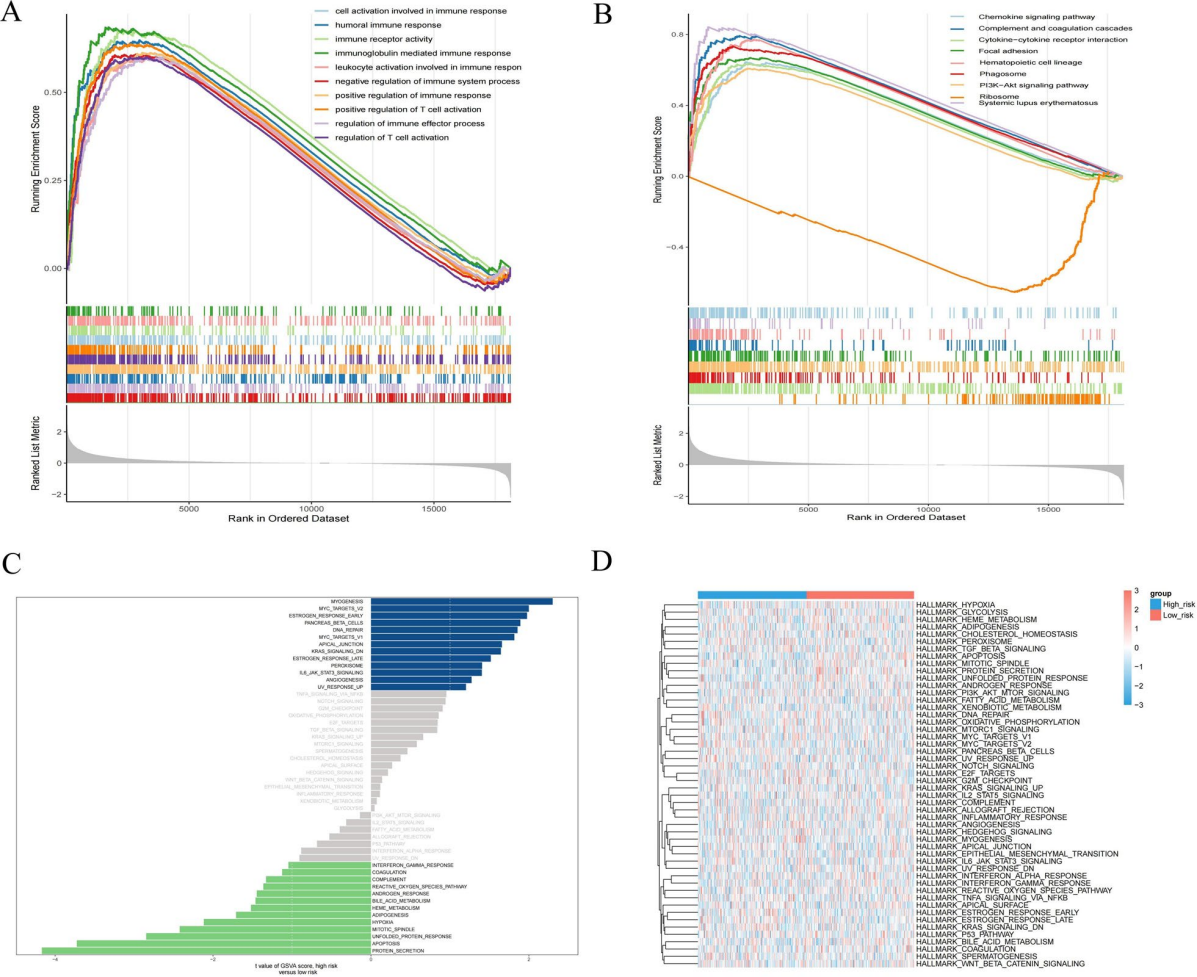
Biological characteristics between high-and low-risk groups. **A**, **B** GSEA analysis of GO and KEGG between high- and low-risk groups. **C**, **D** GSVA analysis of all genes in the high- and low-risk groups to obtain enriched pathways

To analyze the effect of high- and low-risk subgroups on cancer progression, we performed the GSEA to identify the most significant enrichment pathways between the two groups. The results showed that the high-risk group was significantly enriched in immune processes such as cell activation and humoral immune response involved in immune response (Fig. 9A). KEGG showed that pathways such as the chemokine signaling pathway, complement, and coagulation cascade were enriched in the high-risk group and phagosome-related pathways in the low-risk group (Fig. 9B). We also analyzed all genes in the high- and low-risk groups using GSVA. The results showed that the high expression group was activated in the marker entries of myogenesis, MYC target V2, early estrogen response, pancreatic β-cells, DNA repair, MYC target V1, apical junction, KRAS signaling pathway, peroxisome, IL6 JAK STAT3, and angiogenic MYC target, while the low expression group was activated in the marker entries of hypoxia, adipogenesis, heme metabolism, bile acid metabolism, interferon α response pathway, coagulation and other marker entries (Fig. 9C, D).

### Evaluation of the possibility of BLCA immunotherapy

**Fig. 10:**
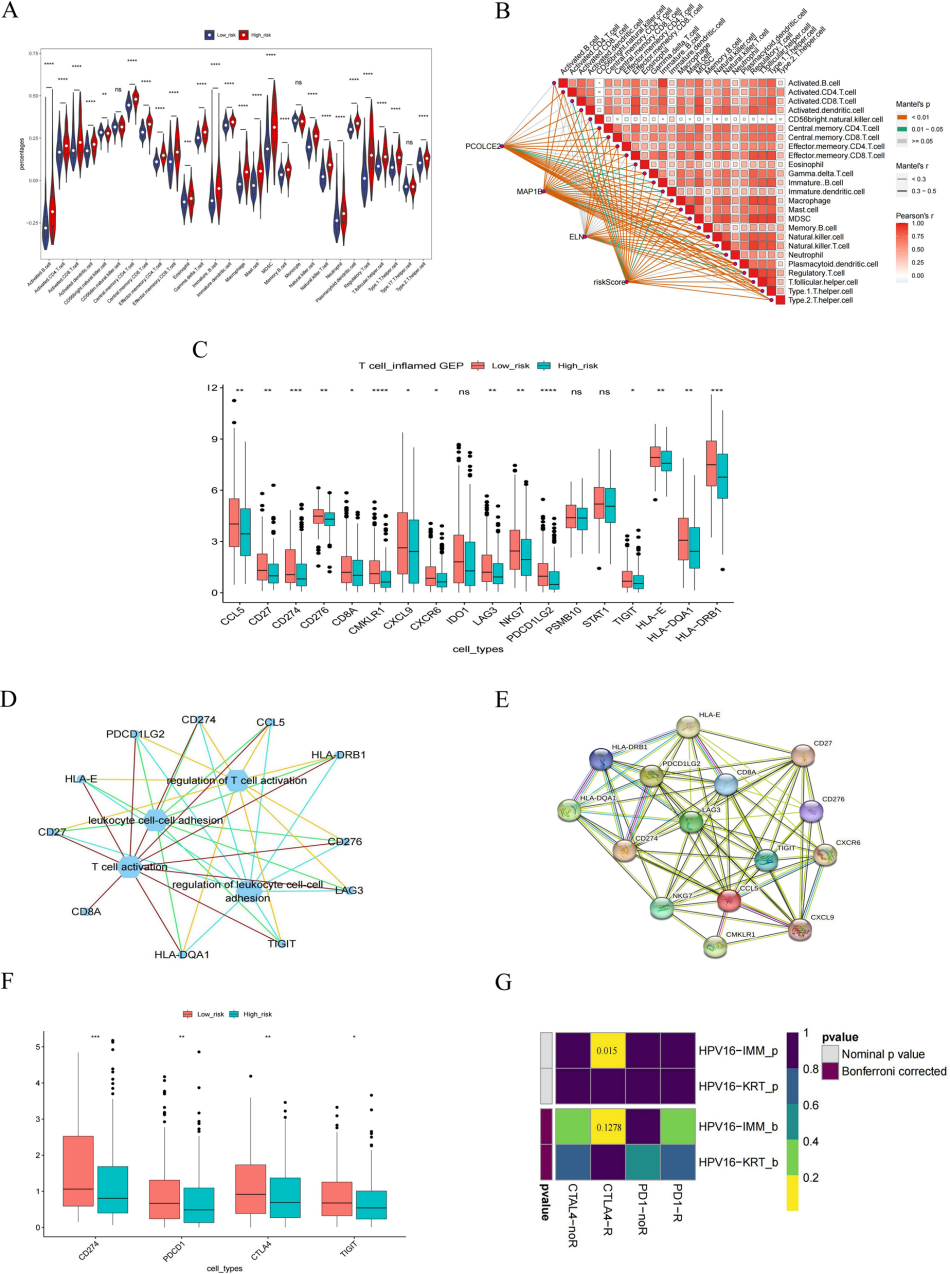
Analysis of the tumor immune microenvironment in high- and low-risk groups. **A** Violin plot visualizing the ssGSEA scores of 28 immune cells between high and low-risk groups. **B** Correlation analysis of risk scores with significantly different immune cells. **C** Box plot visualizing the expression levels of 18 inflammation-related genes between high and low-risk groups. **D** Pathway network map of significantly differentially expressed inflammation-related genes. **E** PPI network of significantly differentially expressed inflammation-related genes. **F** Expression analysis of PD-1, PD-L1, CTLA-4, and TIGIT between high and low-risk groups. **G** Assessment of ICB response in high and low-risk groups

**Fig. 11:**
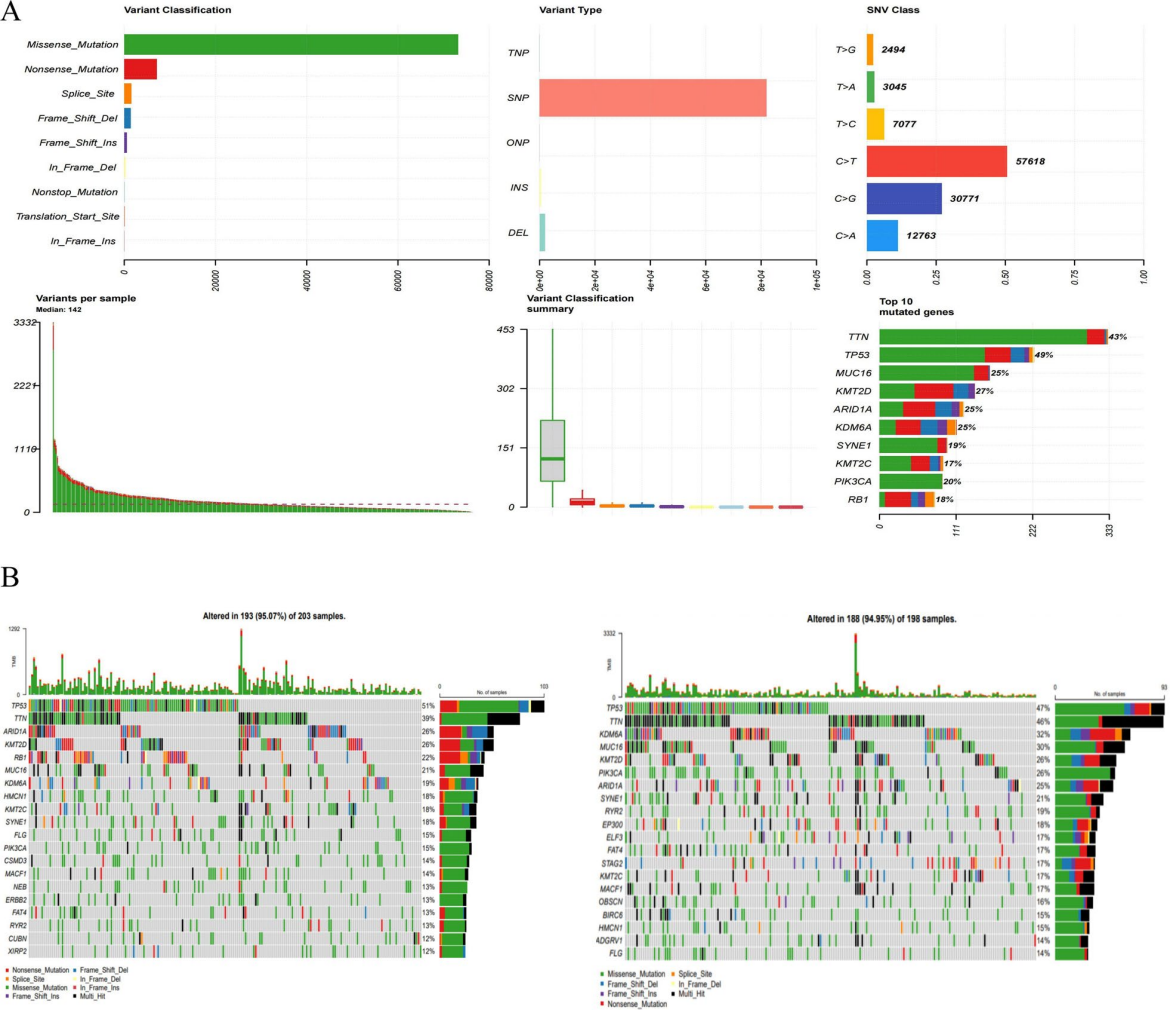
Mutation landscape analysis in BLCA. **A** Overall description of the TCGA-BLCA patient mutation landscape. **B** The tumor mutational burden (TMB) in the high and low‐risk groups was predicted by the risk model

The ssGSEA was used to estimate the infiltration scores of 28 immune cells in different risk groups. The results showed that the differences in the infiltration levels of the 25 immune cell species were statistically significant except for natural. killers.cell, Monocyte, and T.helper.cell (Fig. 10A). The Pearson correlation result displayed that both prognostic genes and risk score were significantly associated with infiltrating immune cells (Fig. 10B). Sixteen GEP genes (inflammatory genes) and four immune checkpoints were significantly different in the high- and low-risk groups (Fig. 10C). Heatmaps of immune cells and 16 differential GEP genes between high and low-risk groups are detailed in Additional file 8: Fig. S8. The interacting network of 16 differential GEP genes and the top 4 ranked pathways (T cell activation, regulation of T cell activation, regulation of leukocyte cell–cell adhesion, leukocyte cell–cell adhesion) is shown in Fig. 10D, which diaplayed the close association between GEP genes and these pathways. The PPI network of differential GEP genes showed the linkage between each GEP gene (Fig. 10E). PD-1, PD-L1, CTLA-4, and TIGIT were different in the high—and low-risk groups (Fig. 10F). By SubMap, in the immunotherapy cohort (Roh cohort), ICB was performed in the high and low-risk groups response was assessed. We found that the CTLA-4 immune loci were sensitive in the Roh cohort (Fig. 10G). We found that BLCA patients were mainly dominated by missense mutations and SNPs (Fig. 11A). The mutation results between high and low-risk groups showed that most mutation types in the high and low-risk groups were missense mutations. The proportion of mutations in the high-risk group was higher than that in the low-risk group, and the mutation load index TMB index was overall higher in the high-risk group than in the low-risk group (Fig. 11B). Above all, our results suggested that immunotherapy has the potential for development in BLCA.

### Drug sensitivity analysis for high- and low-risk groups

**Fig. 12:**
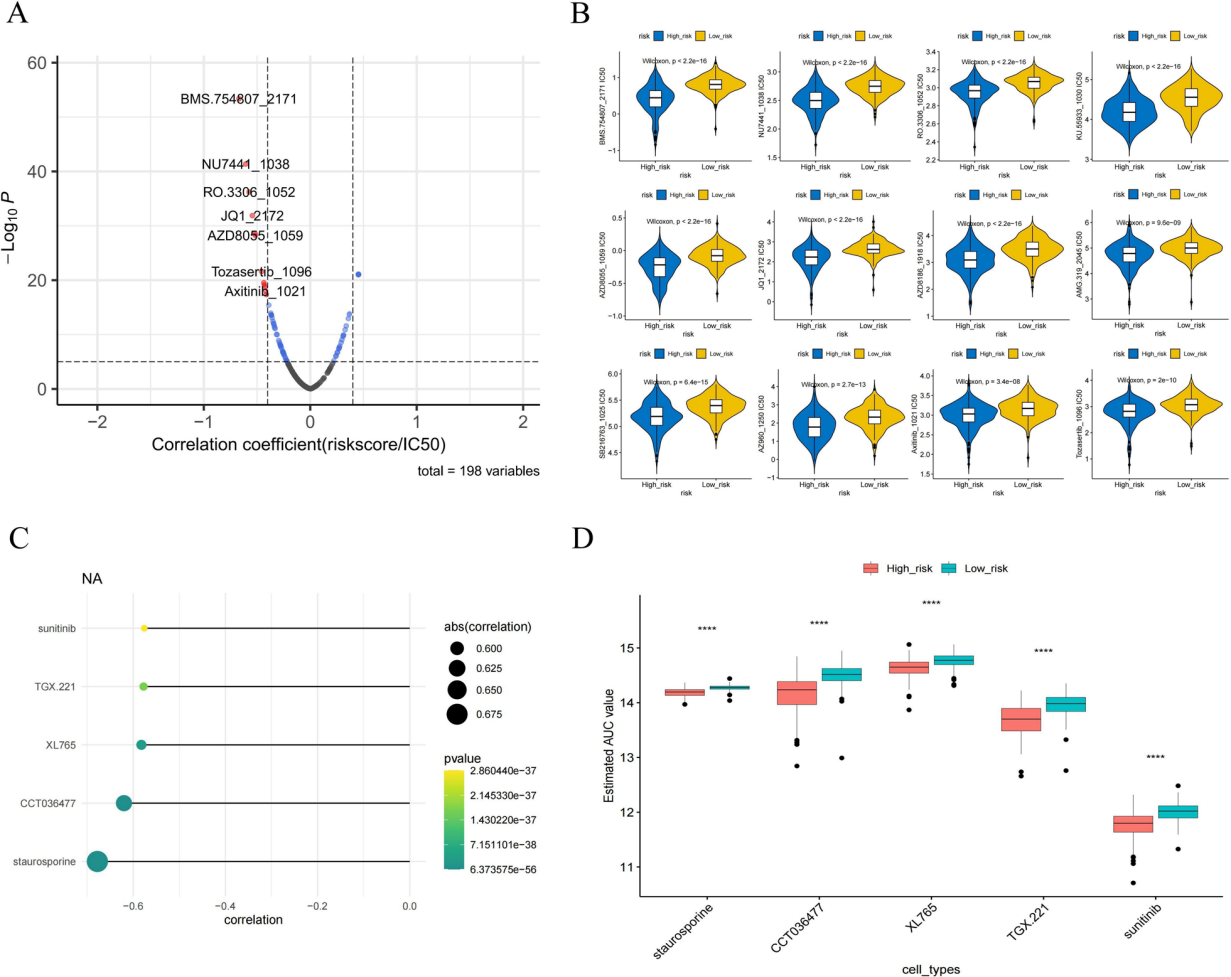
Screening of therapeutic agents for BLCA based on risk models. **A** For the GDSC database, Spearman correlation analysis was performed on BLCA and estimated IC50 values. With a filtering |R| greater than 0.4 and p-value less than 0.05, 12 candidate compounds were identified. **B** Sensitivity analysis of key drugs in high- and low-risk groups. **C** AUC values of CTRP compounds were estimated for each BLCA patient and Spearman analysis was performed on BLCA and AUC values. Dotted line plots visualize the 5 compounds with the highest negative correlation coefficients. **D** The AUC values estimated by the compounds were significantly lower in the high-risk group of BLCA

From the GDSC database, we found that 12 drugs were negatively associated with risk scores (R < -0.4 and p < 0.05), and only the top 7 drugs are shown in Fig. 12A. In addition, we explored the targets and pathways of 12 drugs, 5 of which had no corresponding data (Table 1). 12 chemotherapeutic drugs were significantly different between high and low-risk groups (Fig. 12B). In the CTRP database, the drugs staurosporine, CCT036477, XL765, TGX.221, and sunitinib had the strongest negative association with risk scores (Fig. 12C). Meanwhile, the AUC values of the five drugs were significantly different in both high and low-risk groups (Fig. 12D). In conclusion, these drugs may be promising for the treatment of BLCA.

**Table 1:** The names, IDs, targets, and pathways of the 7 drugs

Drug name	Drug ID	Drug target	Target Pathway	Screening Set
BMS-754807	184	IGF1R, IR	RTK signaling	GDSC1
NU7441	1038	DNAPK	Genome integrity	GDSC1
RO-3306	1052	CDK1	Cell cycle	GDSC1
JQ1	1218	BRD2, BRD3, BRD4, BRDT	Chromatin other	GDSC1
AZD8186	1444	PI3Kbeta, PI3Kdelta	PI3K/MTOR signaling	GDSC1
AZD8055	1059	MTORC1, MTORC2	PI3K/MTOR signaling	GDSC1
SB216763	1025	GSK3A, GSK3B	WNT signaling	GDSC1

## Discussion

BLCA is one of the most common malignancies worldwide, and its incidence is on the rise in many countries. Despite many efforts that have been made recently toward the management of BLCA, the heterogeneous and aggressive characteristics of BLCA are still limited for prognostic assessment [27]. Therefore, screening novel biomarkers to help develop patient-specific therapies and improve prognosis remains critical and urgent. Distinct from bulk RNA-seq focusing on the average expression level of genes in cells, scRNA-seq has emerged as a useful tool for transcriptional stratification to define the cell subpopulations and realize specific biomarkers and heterogeneity among different cell types in various cancers, including BLCA [28]. Therefore, in this study, we conducted a comprehensive analysis of bulk RNA-seq and scRNA-seq to develop a risk model that exhibited excellent prognostic and predictive efficacy for immunotherapy response in BLCA.

First, we identified 7 core cells in the scRNA-seq profile containing 13,490 cells, namely: fibroblasts, B cells, T cells, monocyte cells, endothelial cells, smooth muscle cells, and epithelial cells, in which cellular communication was highly frequent but expression levels were generally down-regulated in tumor samples, and precisely such heterogeneity and interaction with TME that is essential in tumorigenesis and therapy resistance [29]. The results of GO/KEGG analysis of DEGs obtained in TCGA were mainly enriched in the cell cycle and PI3K-Akt signaling pathway, and MAPK signaling pathway, which may be contributed to the proliferation and progression of BLCA. Existing studies indicated that alterations in cyclins, TP53, and Rb genes are ubiquitous in BLCA, particularly in MIBC with higher frequency, and therapy targeting against aberrant cell-cycle regulators may be beneficial in BLCA. Numerous studies have also confirmed that PI3K-Akt and MAPK signaling pathway activation play essential roles in the initiation and progression of BLCA [30–32]. Moreover, we recognized the brown module composed of 2334 genes as the key module using WGCNA. 123 candidate genes were collected by taking the intersection of the above three gene sets to enhance the stability of signatures.

Next, the 3-gene prognostic model was established by univariate Cox regression analysis and LASSO algorithm, involving: MAP1B, PCOLCE2, and ELN, with the ROC curve results demonstrating that it had promising predictive efficacy for prognosis and was an independent prognostic factor for OS in BLCA. Contrary to other models [33], our signatures were the results of integrating multiple datasets and algorithms, validated by internal and external validation sets inconsistent with the training set, and showed AUC values between 0.590–0.813, suggesting higher reliability and relevance, of which we also analyzed the relationship between the model and clinicopathological characteristics, and the results revealed that risk scores were significantly associated with patients' lymph node metastasis and tumor stage, indicating that the model is not limited to having predictive value for OS. Besides, we also observed 3 signature genes related to the tumor microenvironment. MAP1B is one of the Microtubule-associated proteins (MAPs), which is involved in cytoskeleton composition. It has been reported that MAP1B was remarkably overexpressed in BLCA tissues and positively correlated with tumor pathological tumor stage, grade, lymph node metastasis and vascular invasion, knockdown of MAP1B could reverse chemoresistance by interrupting the cell cycle [34]. PCOLCE2 is a collagen-binding protein that functions as a pivotal component in tumor microenvironment remodeling [35], and a previous study also demonstrated that down-regulation of PCOLCE2 expression resulted in better OS [36]. ELN is a crucial element of the extracellular matrix that promotes breast cancer progression by enhancing the activation of matrix metalloproteinases (MMPs) but is scarcely documented in BLCA [37]. We also considered that clinical characteristics could have an impact on the prognosis of patients, so clinical characteristics were subjected to multifactorial Cox analyses, and the findings revealed the independent influences of Stage and risk score on the OS of BLCA patients, which were further constructed as a nomogram model, with a calibration curve verifying the remarkably favorable predictive ability.

Furthermore, all samples were divided into low- and high-risk groups according to the calculated risk score, and we observed that the high-risk group was mainly enriched in immune processes and immune-related pathways, thus it was hypothesized that the risk score could be a potential predictive indicator for BLCA patients undergoing immunotherapy. We approached this from different perspectives evaluating TMB, immune infiltration, immune checkpoints, and associated inflammatory genes, and concluded that the high-risk group presented higher TMB and significantly elevated infiltrations of multiple immune cells, but significantly lower expression of inflammatory genes and immune checkpoints than the low-risk group, supporting that patients with low-risk scores are more likely to benefit from immunotherapy.

Finally, potential druggable targets and corresponding compounds for BLCA patients were identified from the GDSC and CTRP databases in light of developed prognostic models, primarily including cell cycle (staurosporine and RO-3306), PI3K/mTOR pathway (XL765, TGX-221, AZD8186, and AZD8055) and Wnt pathway inhibitors (CCT036477 and SB216763), which were compatible with the pathway enrichment results of DEGs. Owing to the potency and promiscuity of these drugs, they have not been adopted in the clinic yet, but they will become quite promising antitumor drugs in the future with technological renovation.

Taking into utmost consideration tumor heterogeneity, interactions of each cell population, immune infiltration, TMB, and clinical characteristics, the strength of this work lies in the identification and construction of a novel prognostic model capable of accurately discriminating survival outcomes and immunotherapeutic response in BLCA, and the findings acquired in this study provide direct evidence for stratified and precise treatment of BLCA patients. However, there are several inescapable limitations of this study: (1) the sample size of scRNA-seq data is relatively small; (2) the regulatory mechanisms of signature genes in BLCA remain ambiguous, and which is exactly what future work arising from this study should continue to explore.

## Conclusions

By integrating scRNA-seq and bulk RNA-seq data, we performed multiple machine-learning methods and established a novel prognostic model for OS prediction in BLCA patients that could be applied to predict the survival probability of BLCA patients. Moreover, the risk score is a promising independent prognostic factor that is closely correlated with the immune microenvironment and clinicopathological characteristics. Overall, this study could be used as a reliable predictor of BLCA efficacy, opening up new avenues for targeted treatment of BLCA in the future.

## Supplementary Information

**Additional file 1: Figure S1.** Flow chart of the present study.

**Additional file 2: Figure S2.** Expression of 22 marker genes in the corresponding cell clusters.

**Additional file 3: Figure S3.** The expression of several marker genes for CAFs in seven cell types.

**Additional file 4: Figure S4.** Interaction network between 7 key cells.

**Additional file 5: Figure S5.** DFS of patients with BLCA in the training set (A) and internal validation set (B) from the TCGA-BLCA cohort.

**Additional file 6: Figure S6.** Stratified survival analysis of risk models and clinical characteristics.

**Additional file 7: Figure S7.** Validation of (A-C) internal test set risk models; (D-F) risk model evaluation in GSE13607; (J-I)) risk model evaluation in GSE32548.

**Additional file 8: Figure S8.** Heatmaps of immune cells and 16 differential GEP genes between high- and low-risk groups.

**Additional file 9: Table S1** Patients information and sequencing statistics in GSE12984 dataset.

**Additional file 10: Table S2** 474 significantly different marker genes.

**Additional file 11: Table S3** The key module genes.

## Data Availability

The analyzed datasets generated during the study are available from the corresponding author upon reasonable request.
