# Supplementary figures and images for: Comprehensive analysis of scRNA-Seq and bulk RNA-Seq reveals dynamic changes in the tumor immune microenvironment of bladder cancer and establishes a prognostic model
Source: J Transl Med. 2023 Mar 27;21:223. doi: 10.1186/s12967-023-04056-z (PMC10044739; doi:10.1186/s12967-023-04056-z)

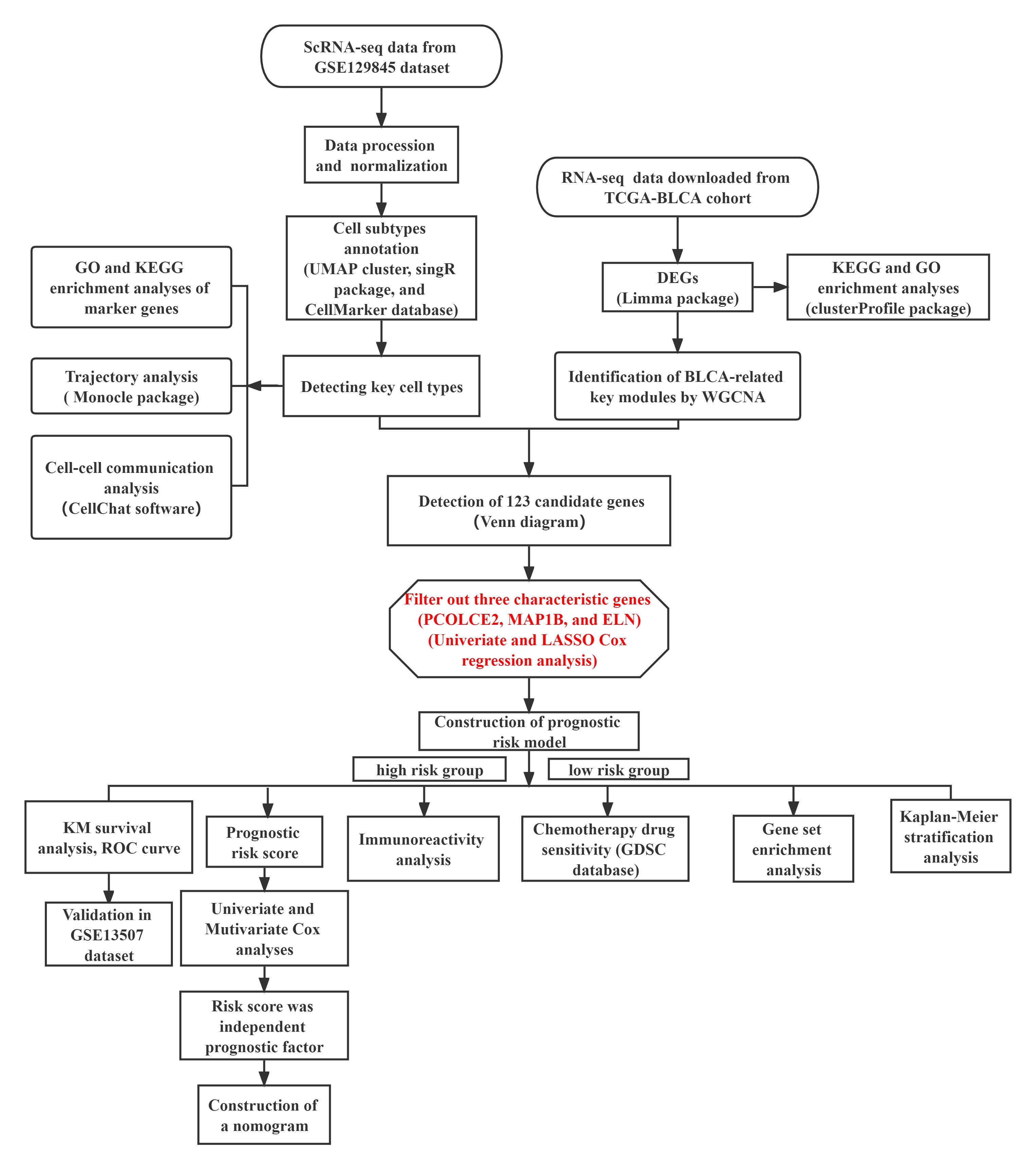

Supplement: Supplementary file 1 — Additional file 1: Figure S1. Flow chart of the present study. [file 12967_2023_4056_MOESM1_ESM.jpg]

# ACTG2

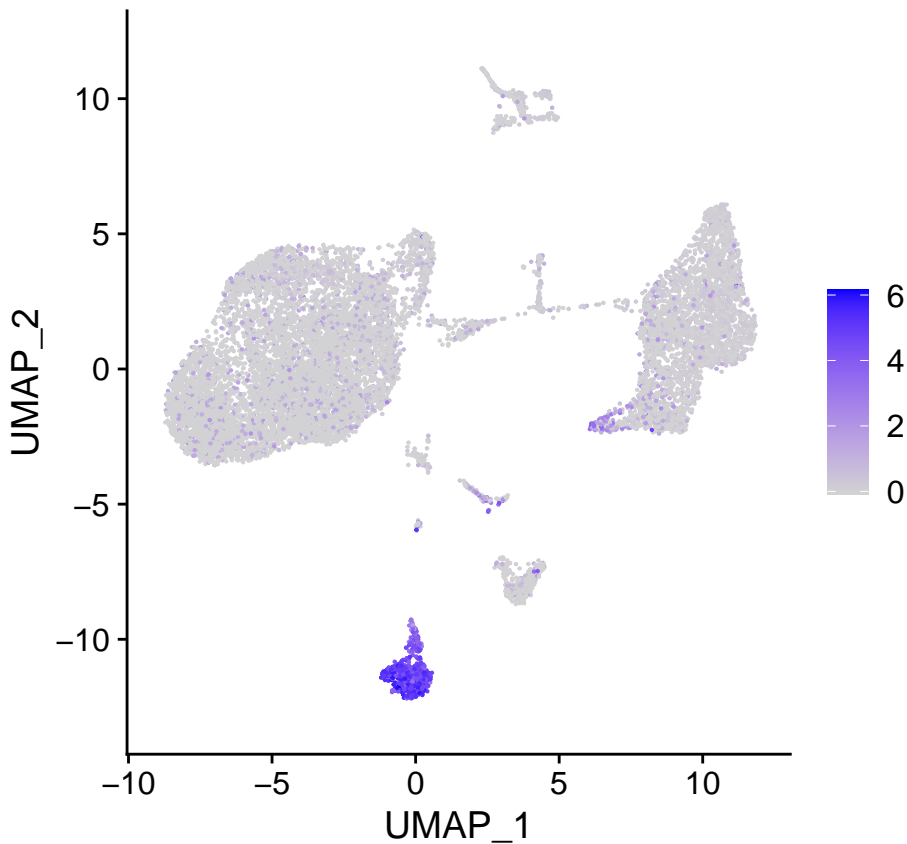

# CD3D

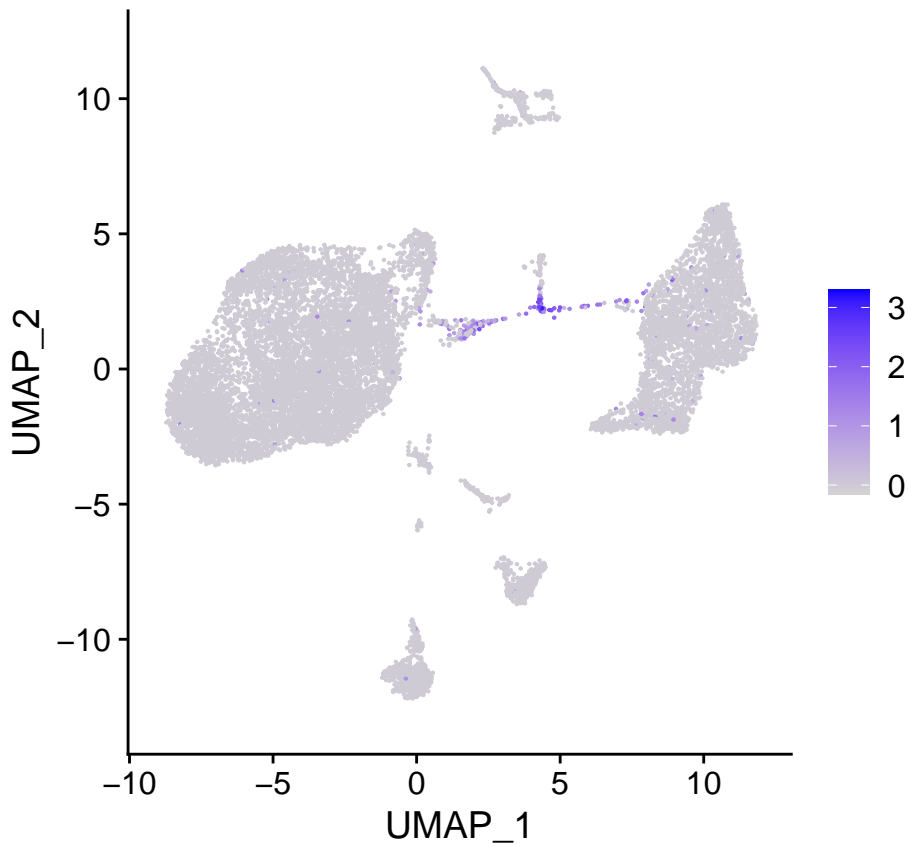

**CD3E**

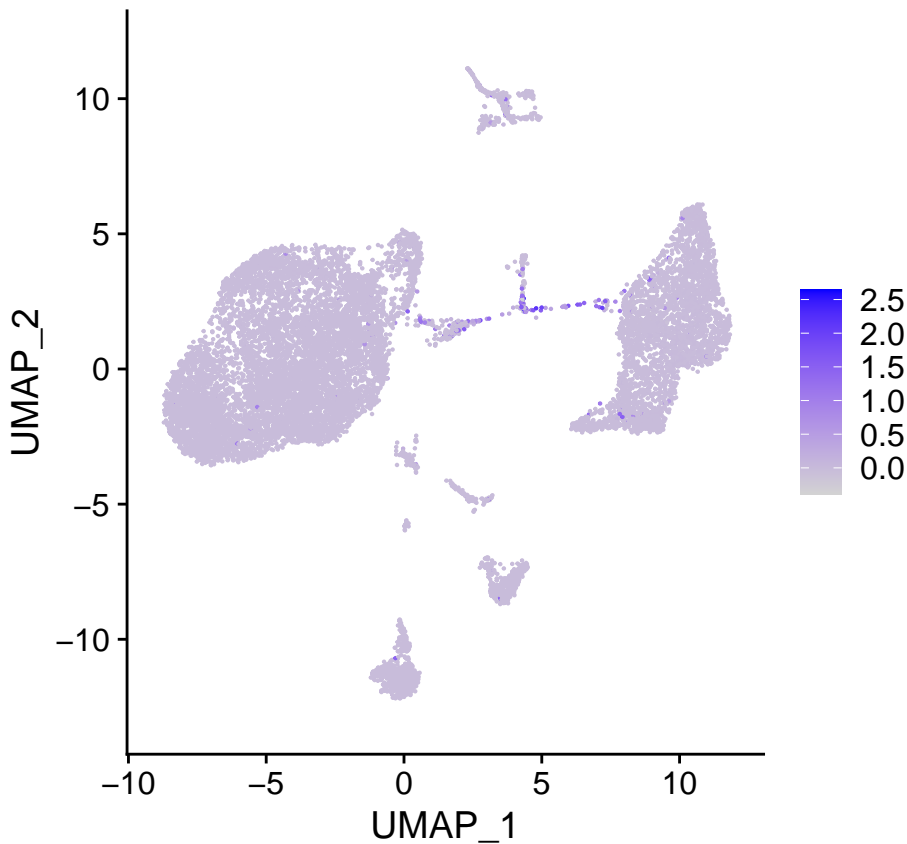

# CD14

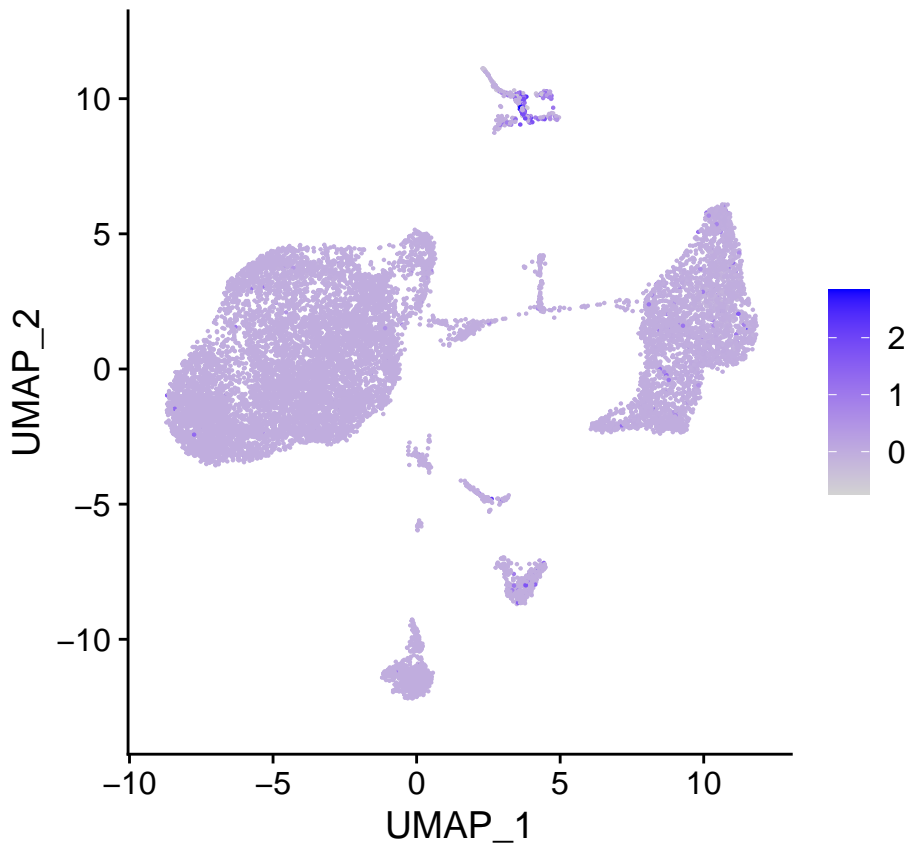

# CD79A

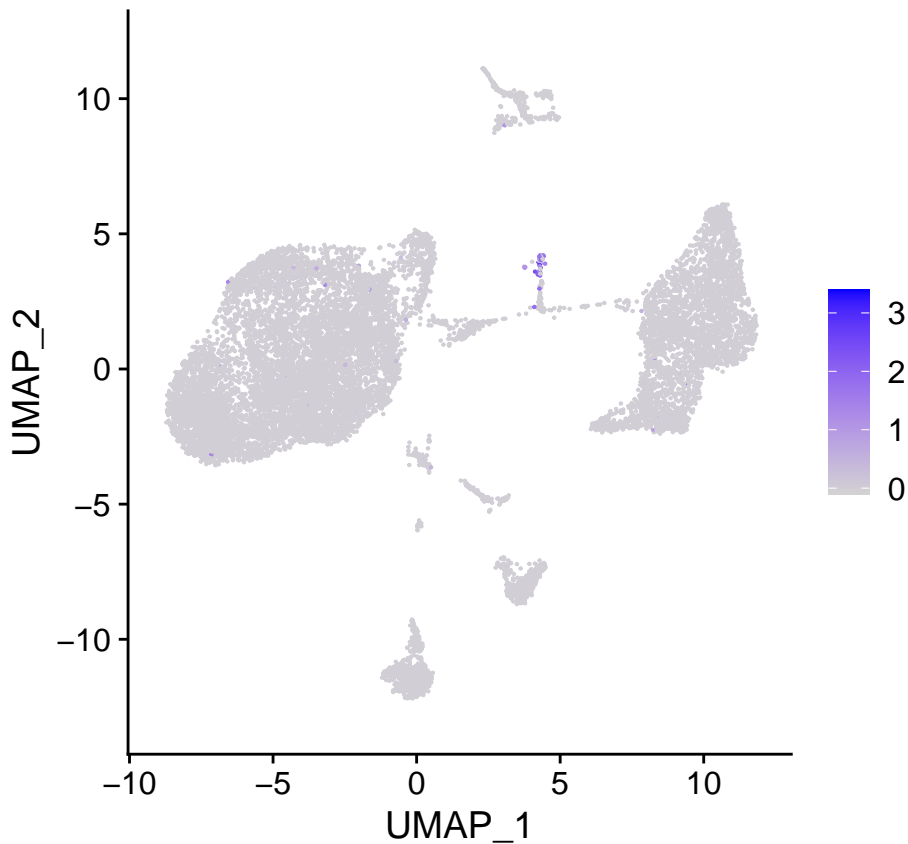

# CDH5

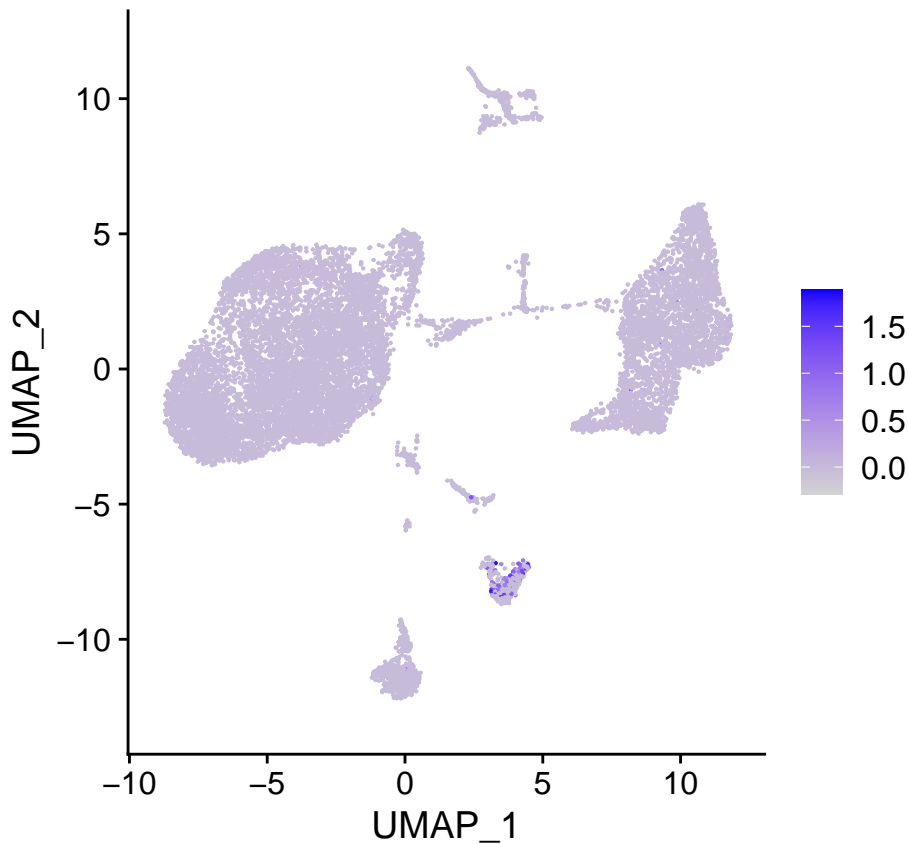

# CNN1

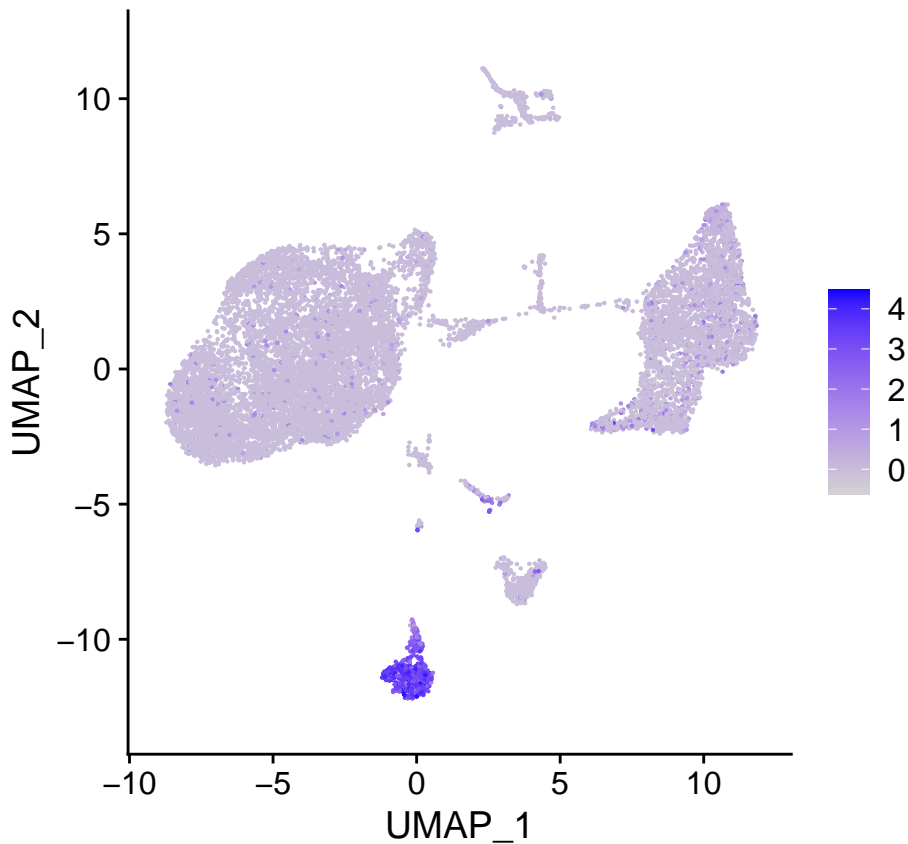

# COL1A1

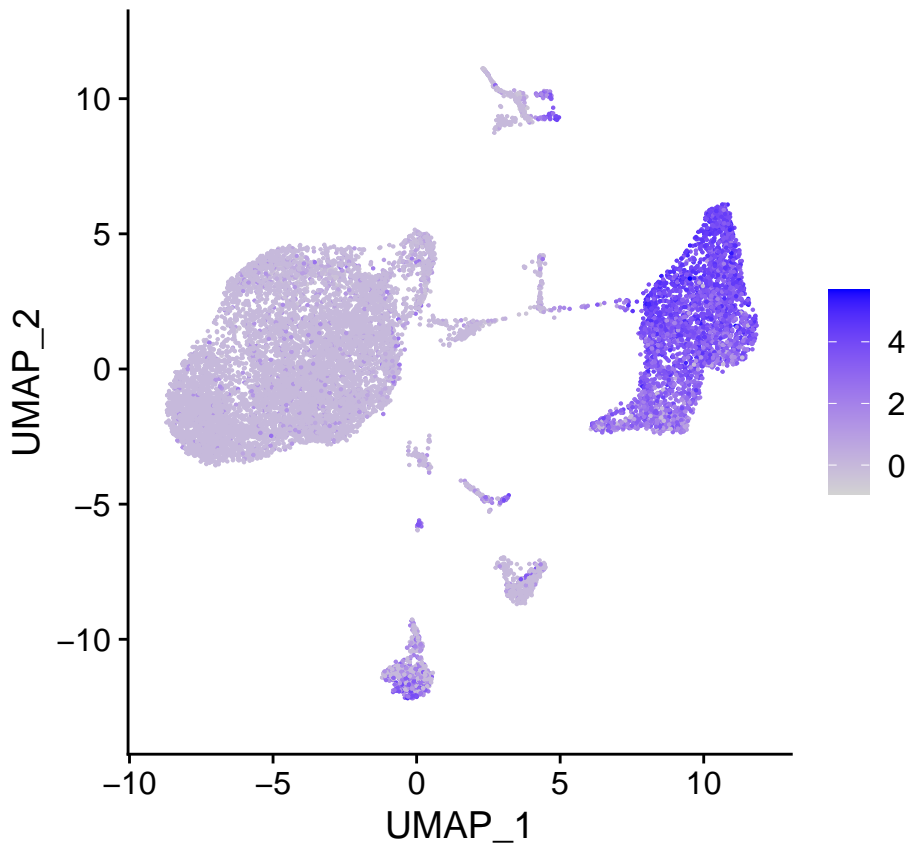

# COL1A2

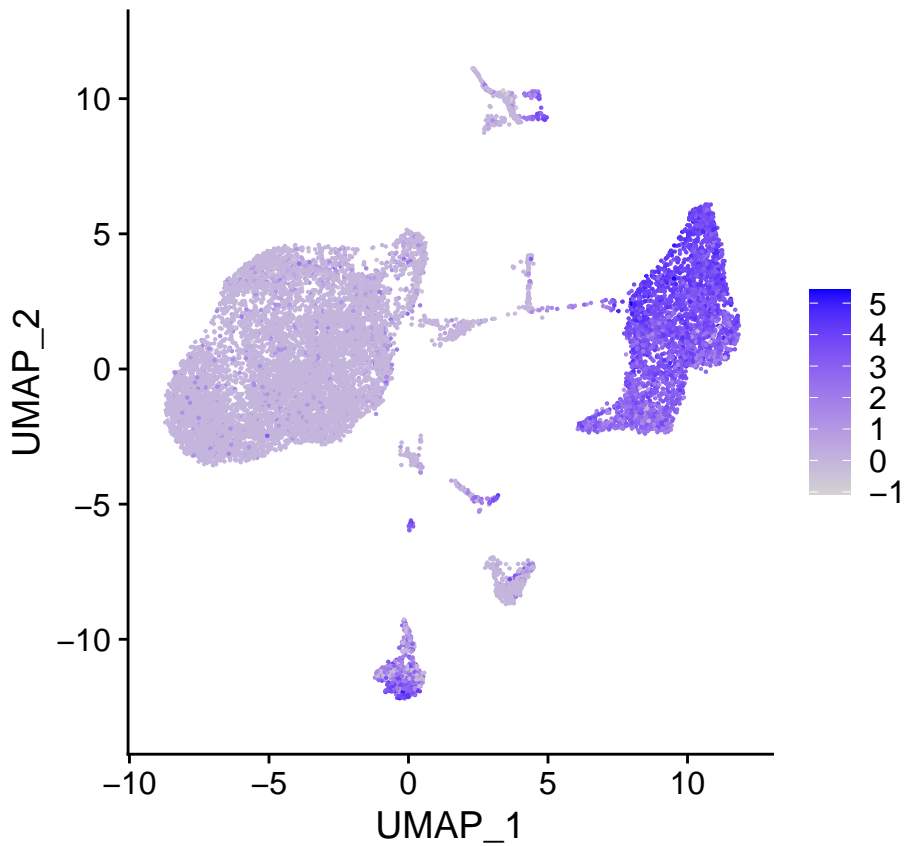

# COL3A1

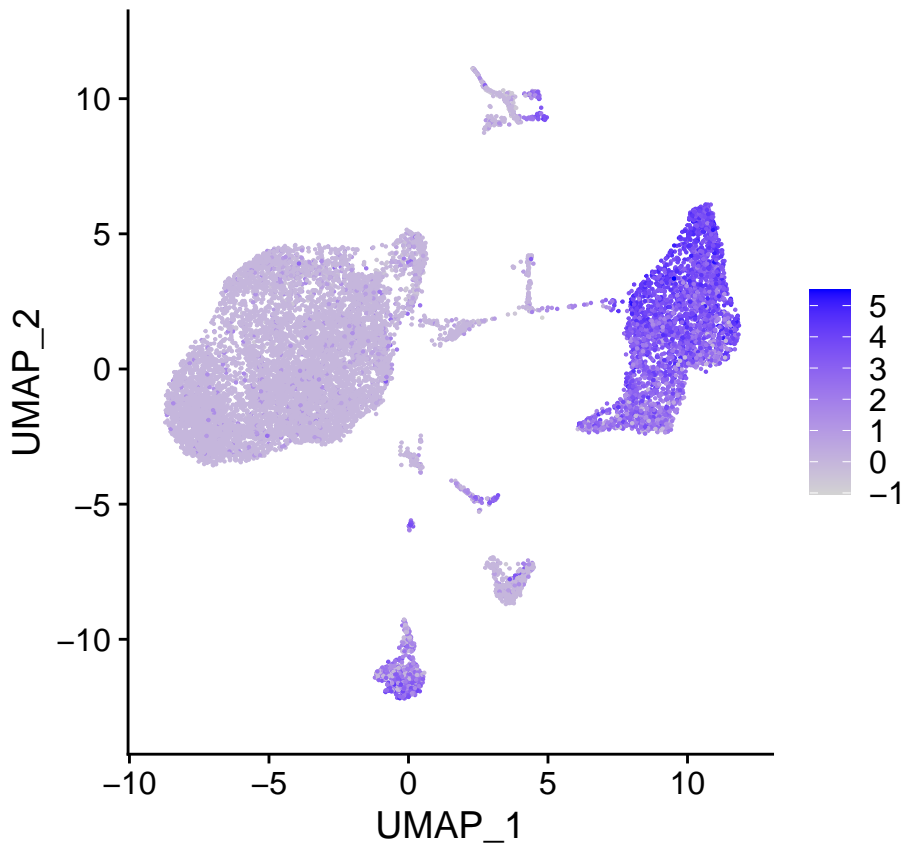

# DES

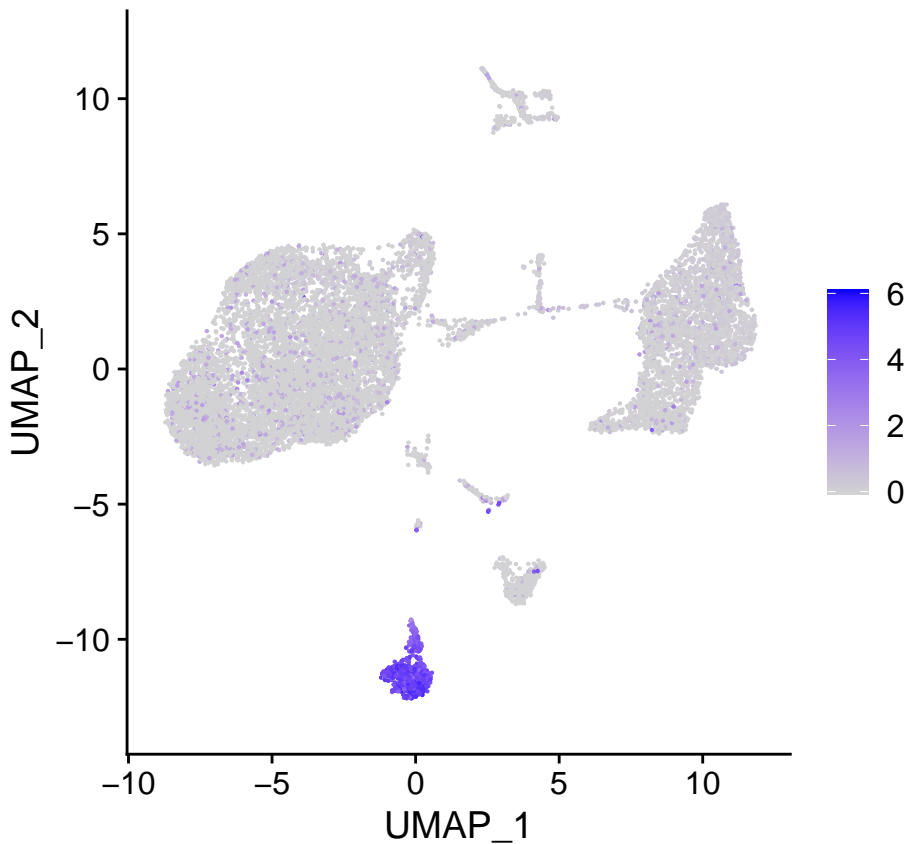

# KRT18

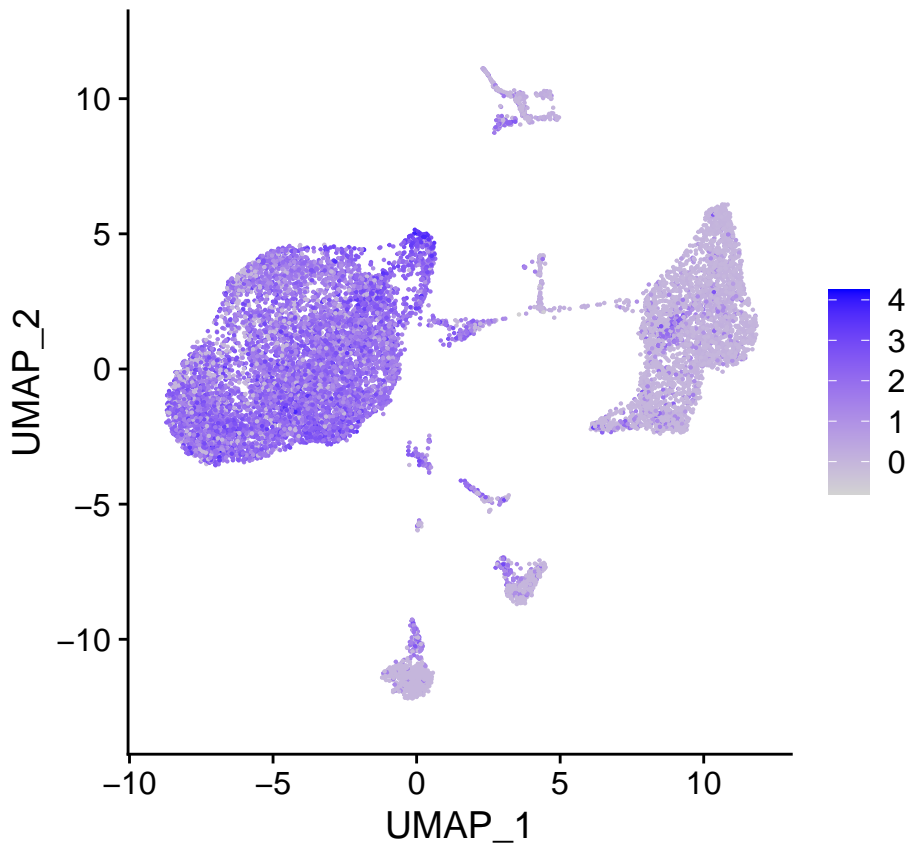

# KRT19

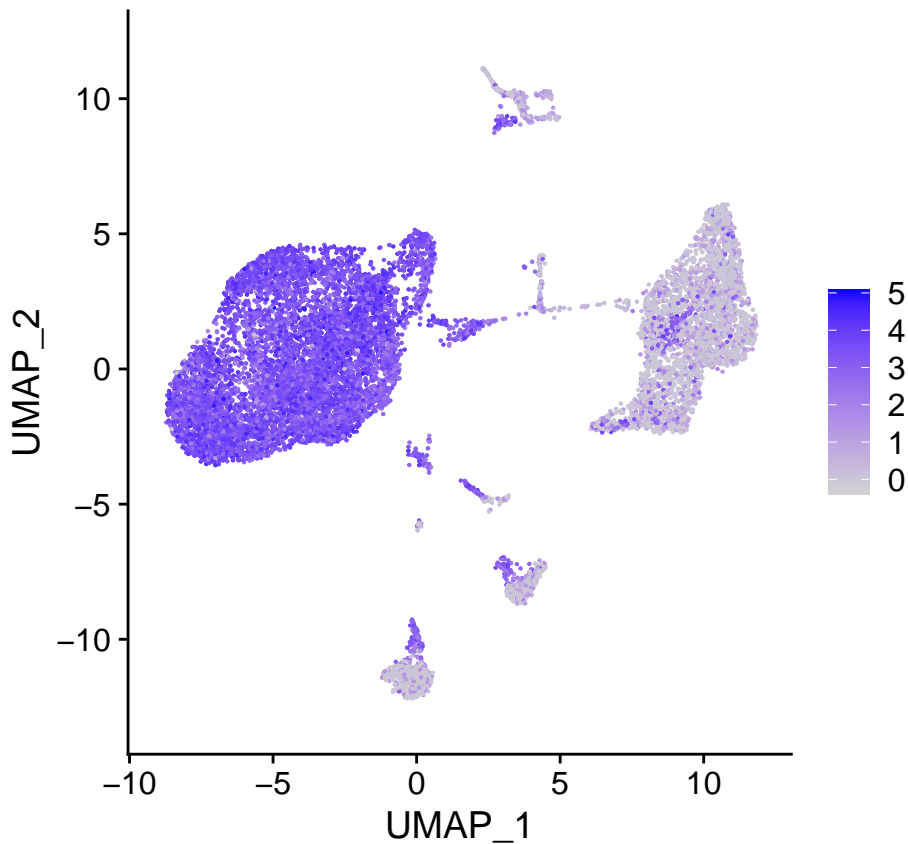

LYZ

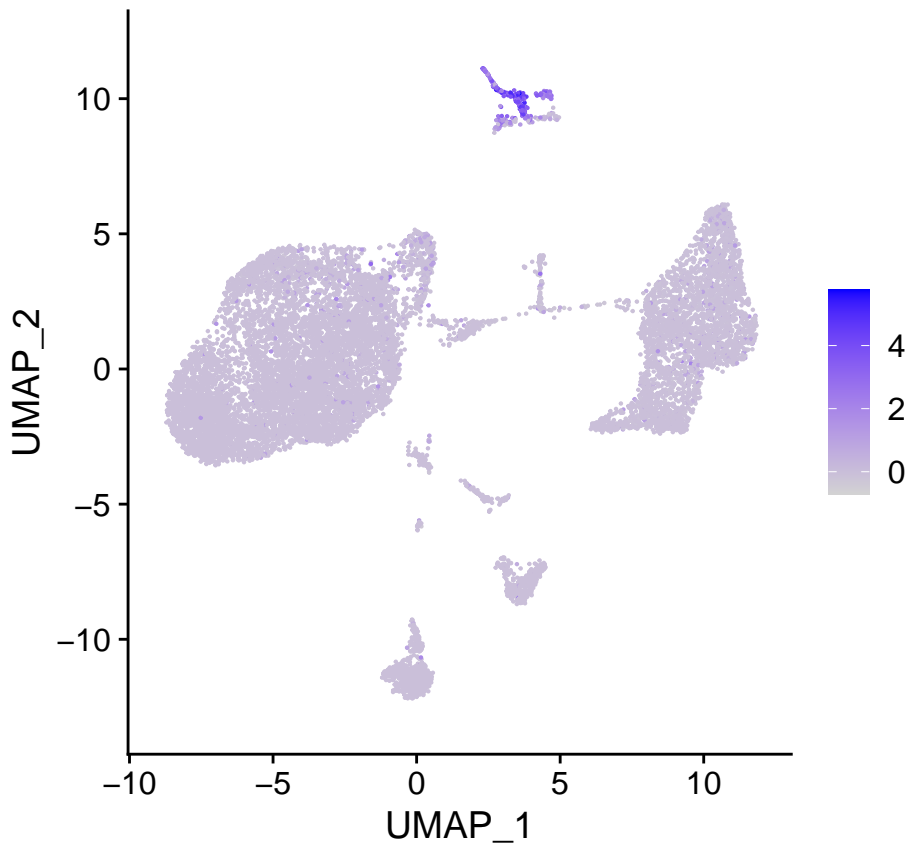

# MS4A7

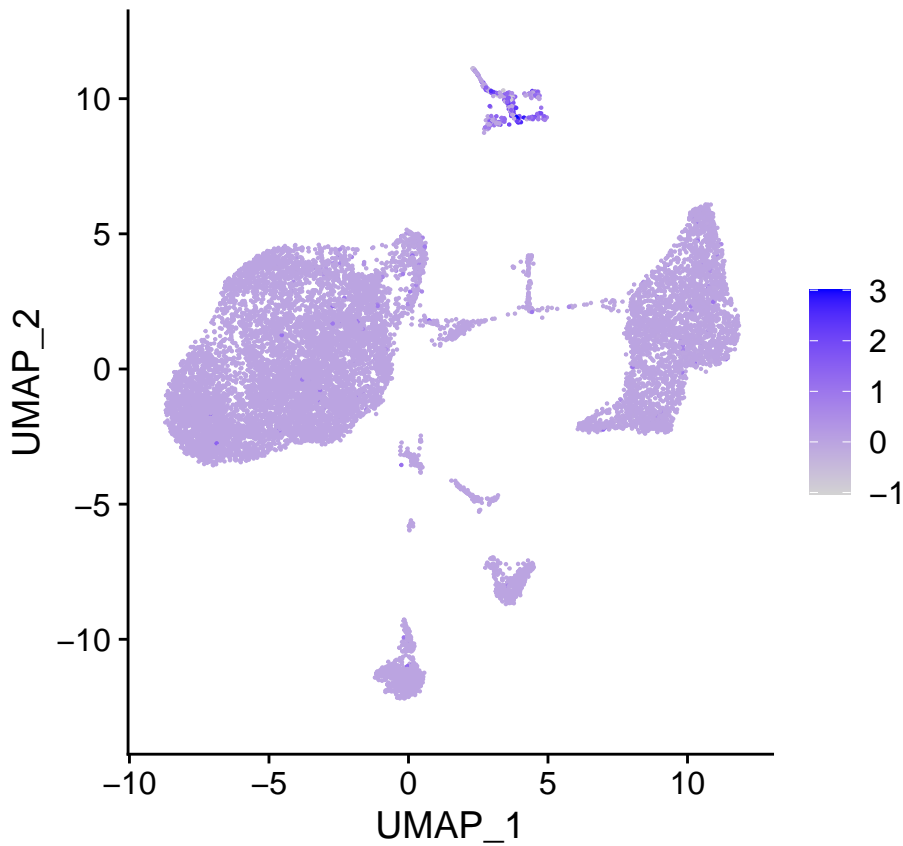

# MZB1

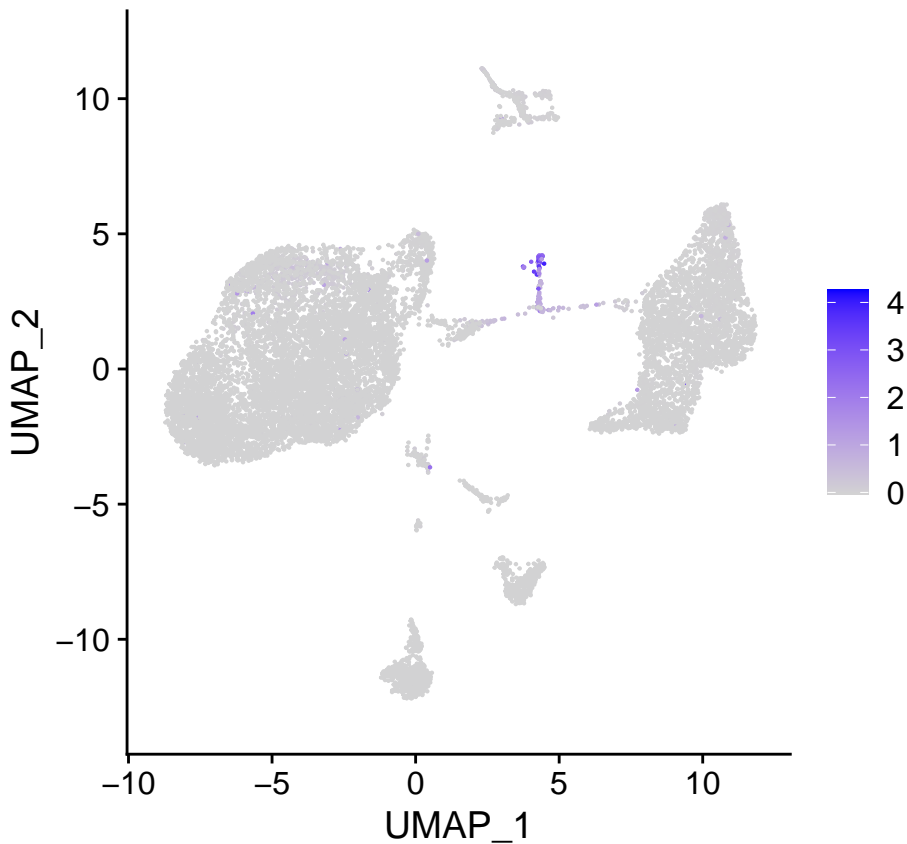

# PECAM1

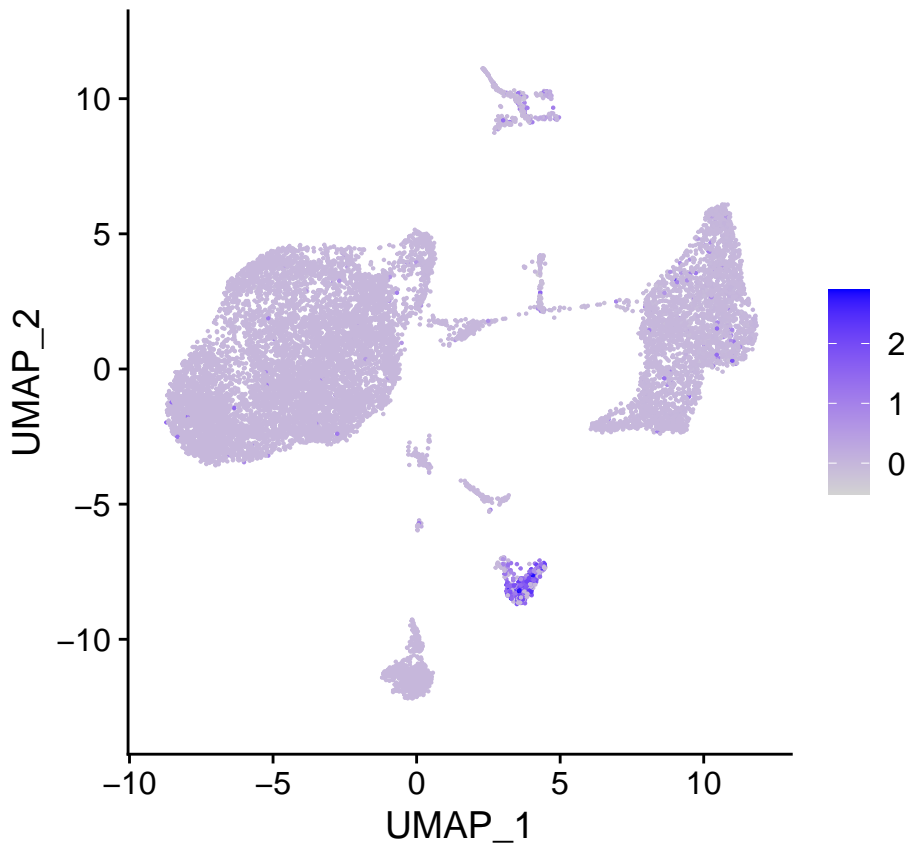

# S100A4

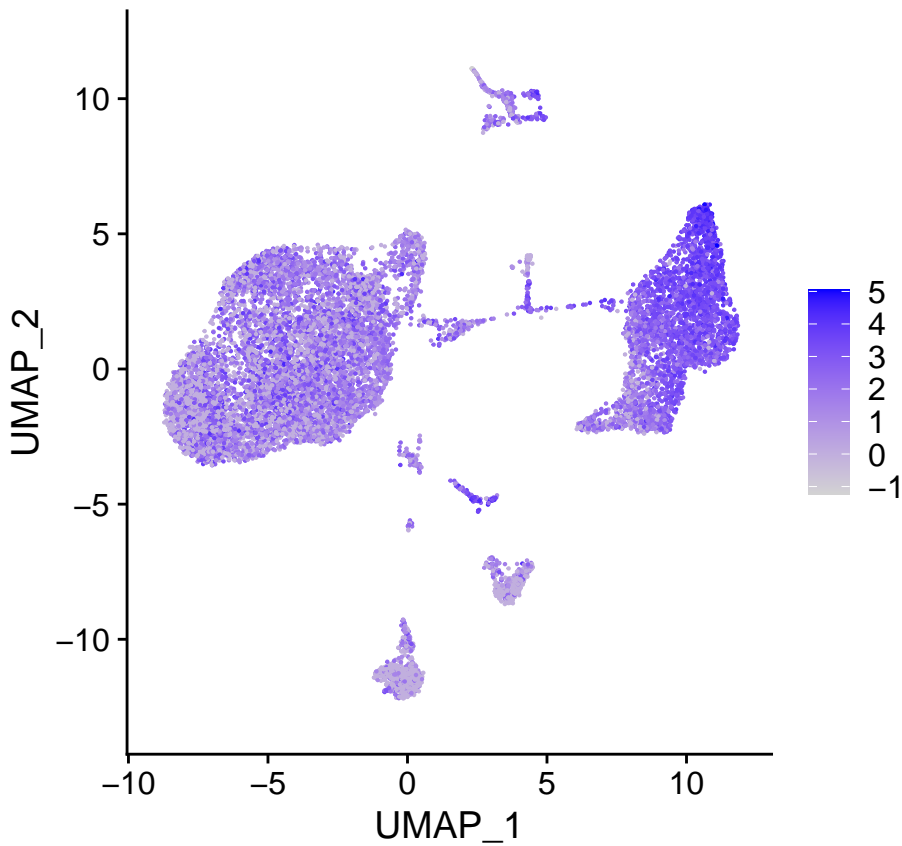



# TAGLN

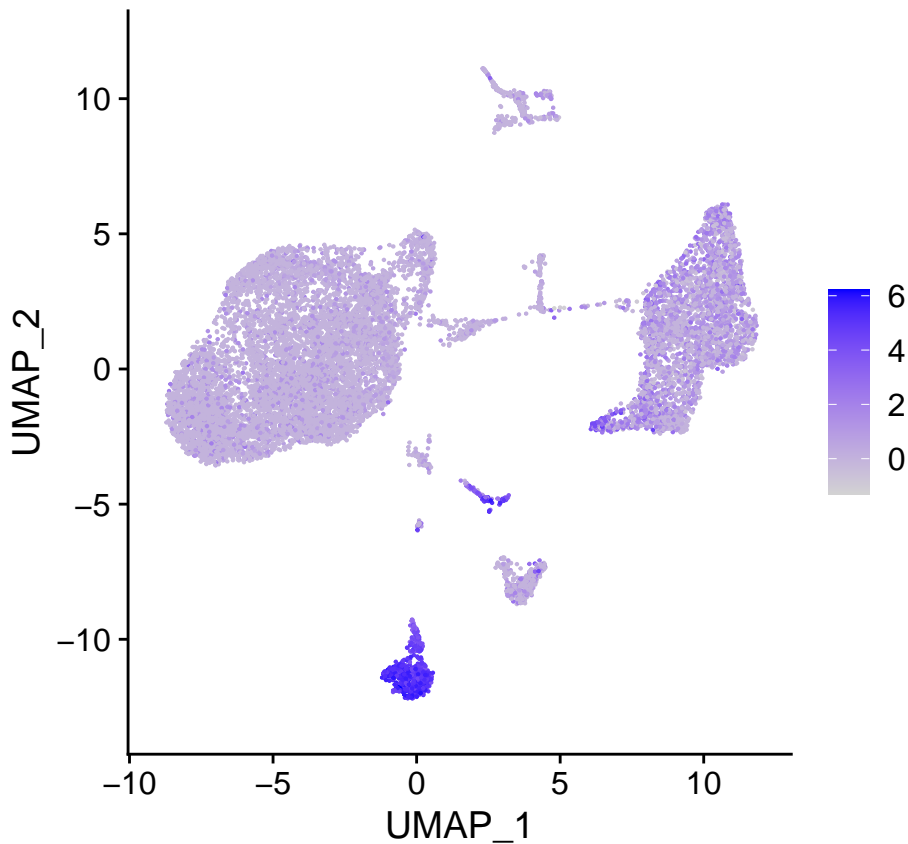

**TPM2**

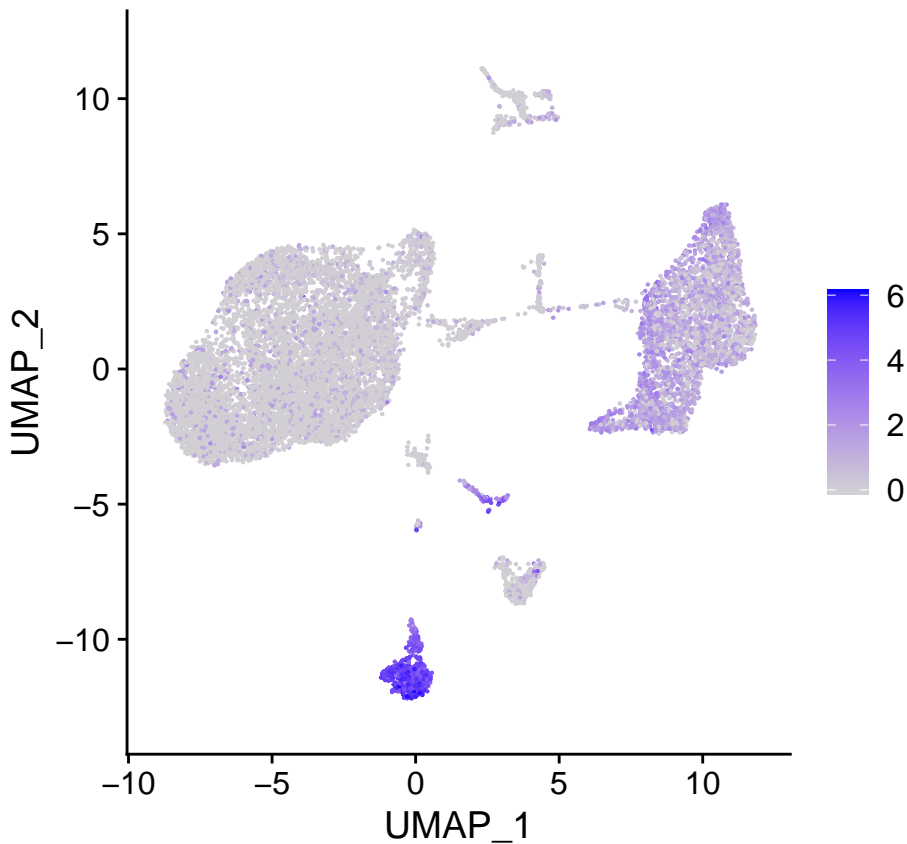

# VCAM1

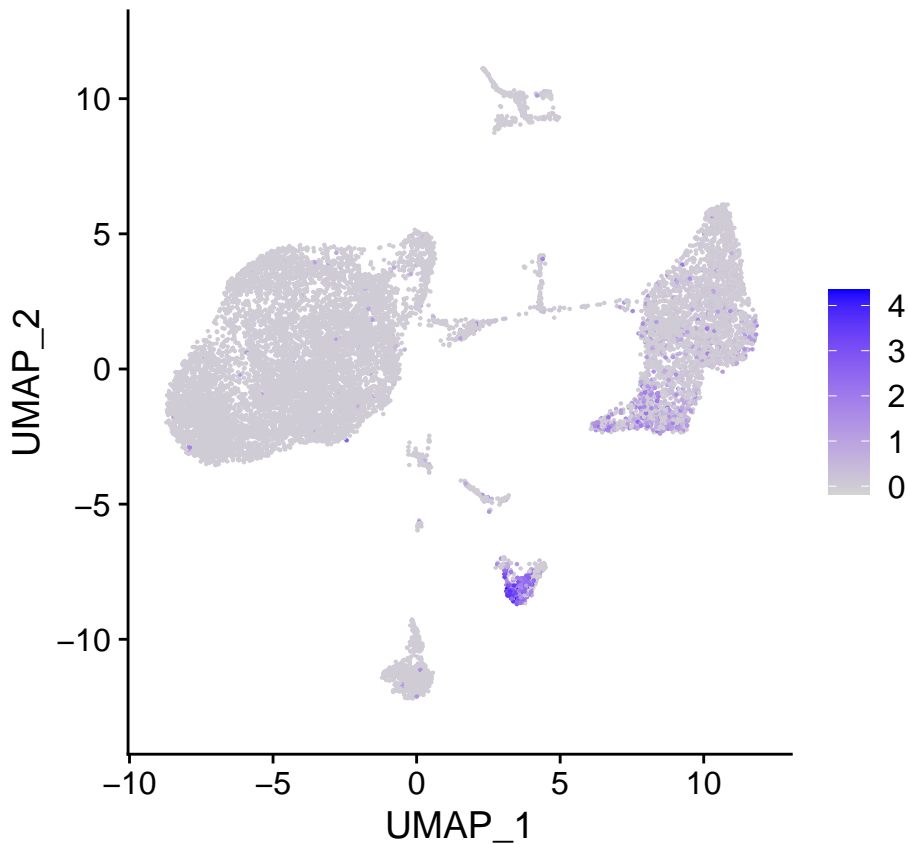

Supplement: Supplementary file 2 — Additional file 2: Figure S2. Expression of 22 marker genes in the corresponding cell clusters. [file 12967_2023_4056_MOESM2_ESM.pdf]

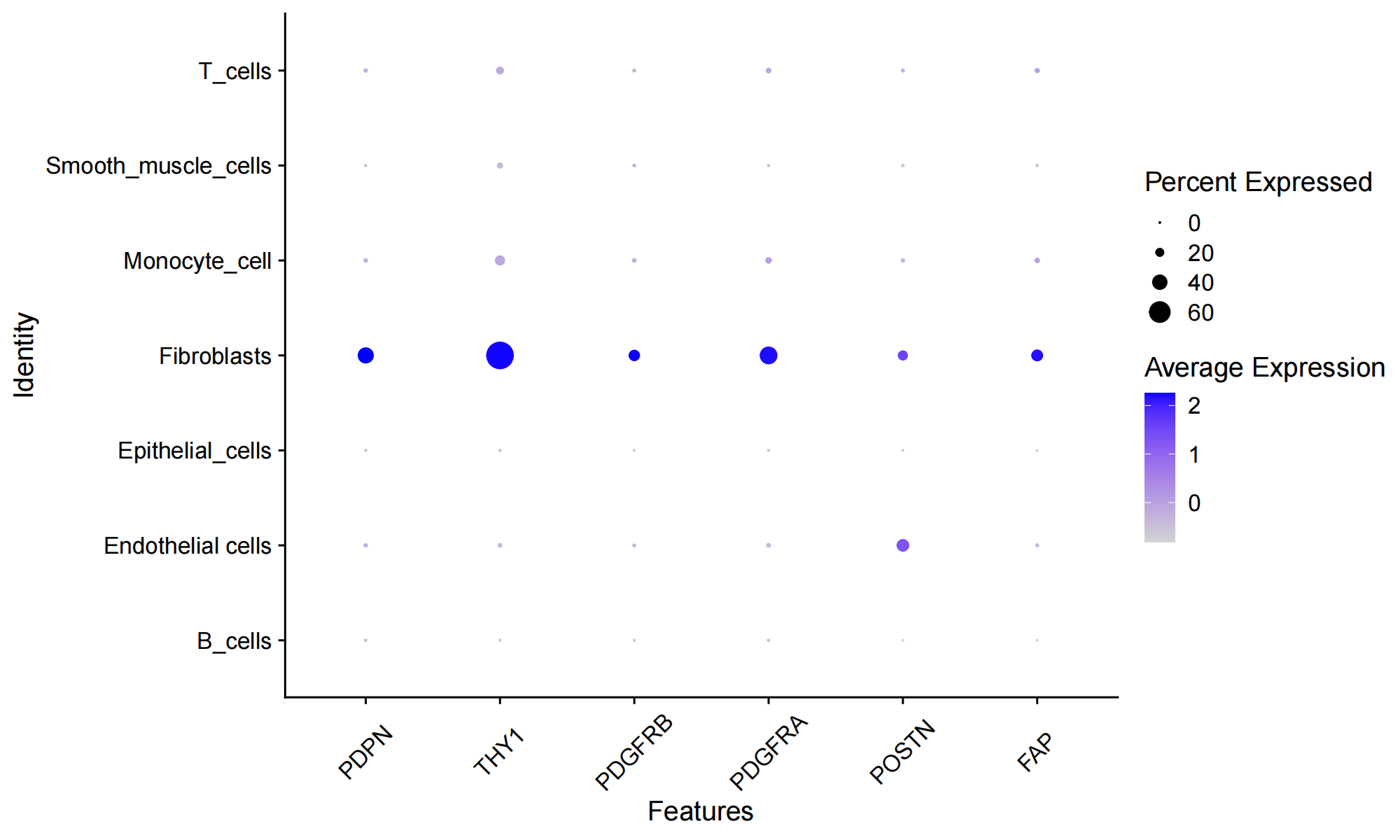

Supplement: Supplementary file 3 — Additional file 3: Figure S3. The expression of several marker genes for CAFs in seven cell types. [file 12967_2023_4056_MOESM3_ESM.tif]

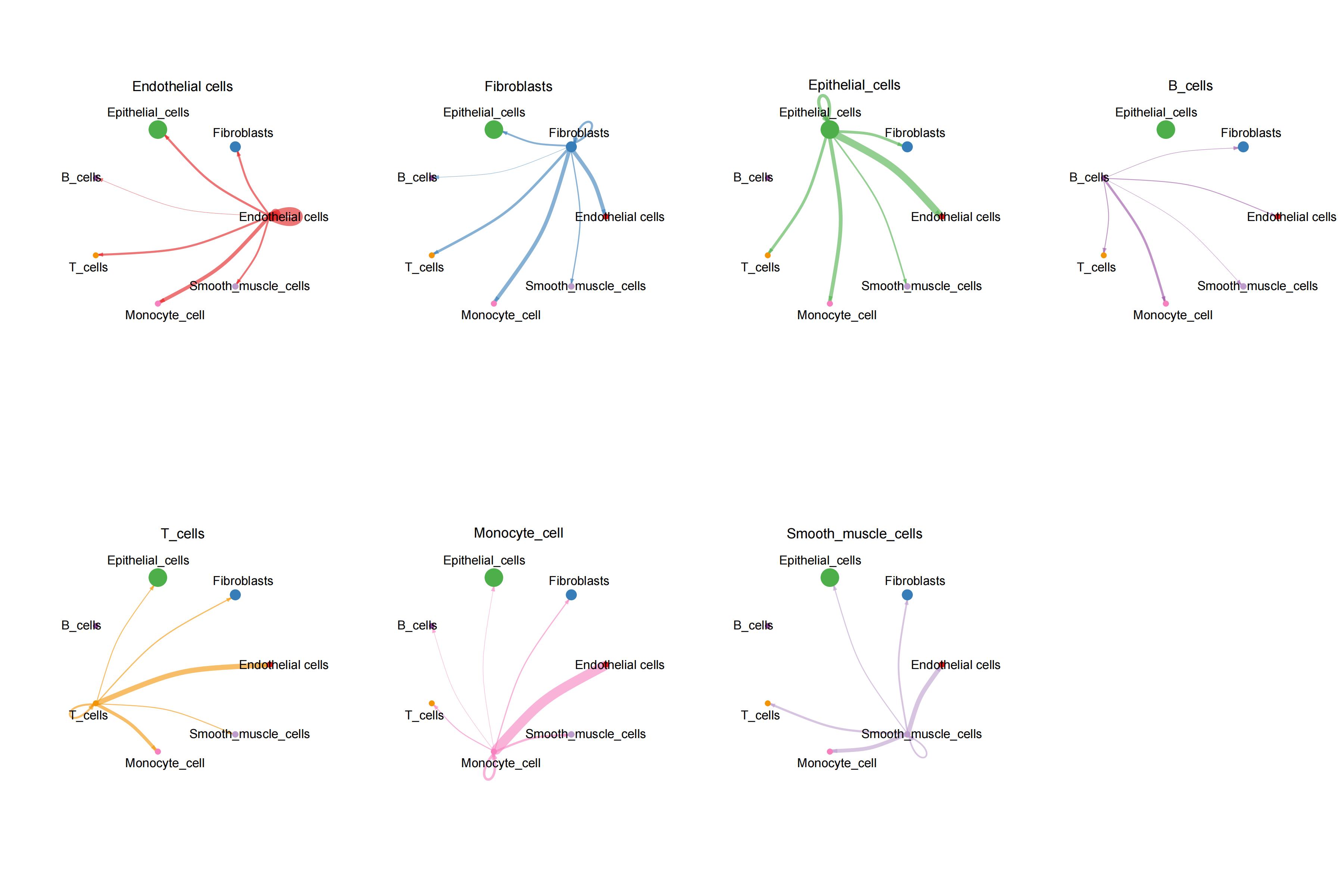

Supplement: Supplementary file 4 — Additional file 4: Figure S4. Interaction network between 7 key cells. [file 12967_2023_4056_MOESM4_ESM.jpg]

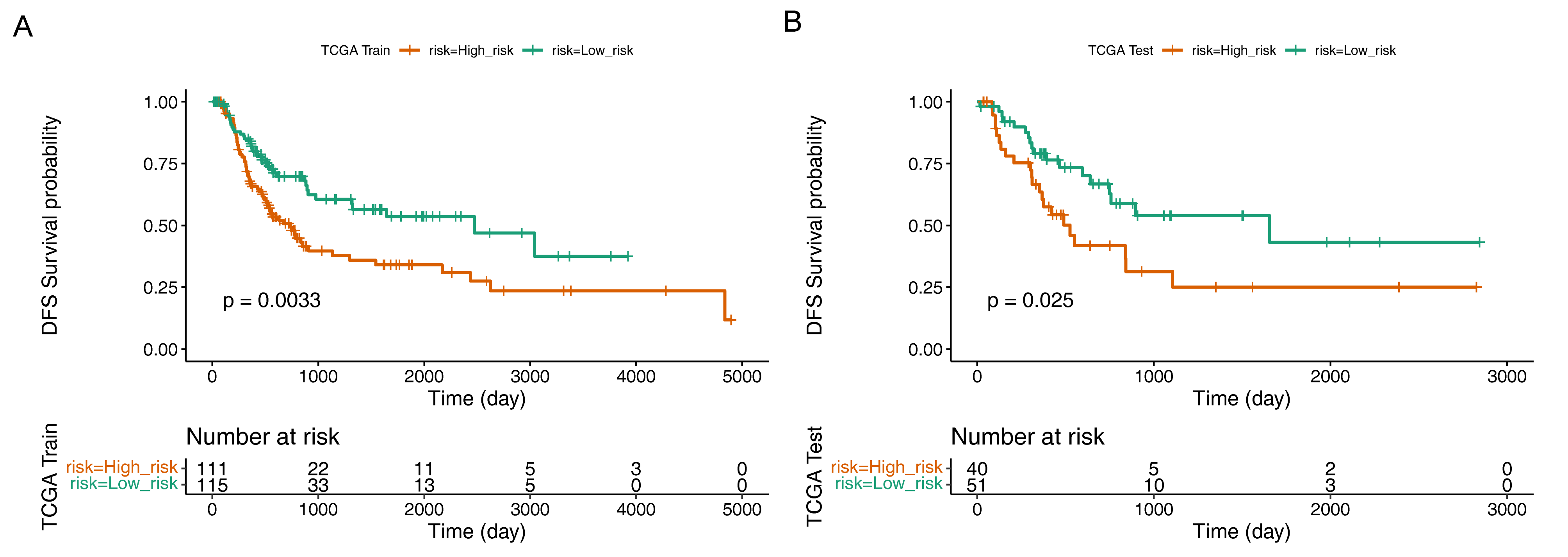

Supplement: Supplementary file 5 — Additional file 5: Figure S5. DFS of patients with BLCA in the training set (A) and internal validation set (B) from the TCGA-BLCA cohort. [file 12967_2023_4056_MOESM5_ESM.tif]

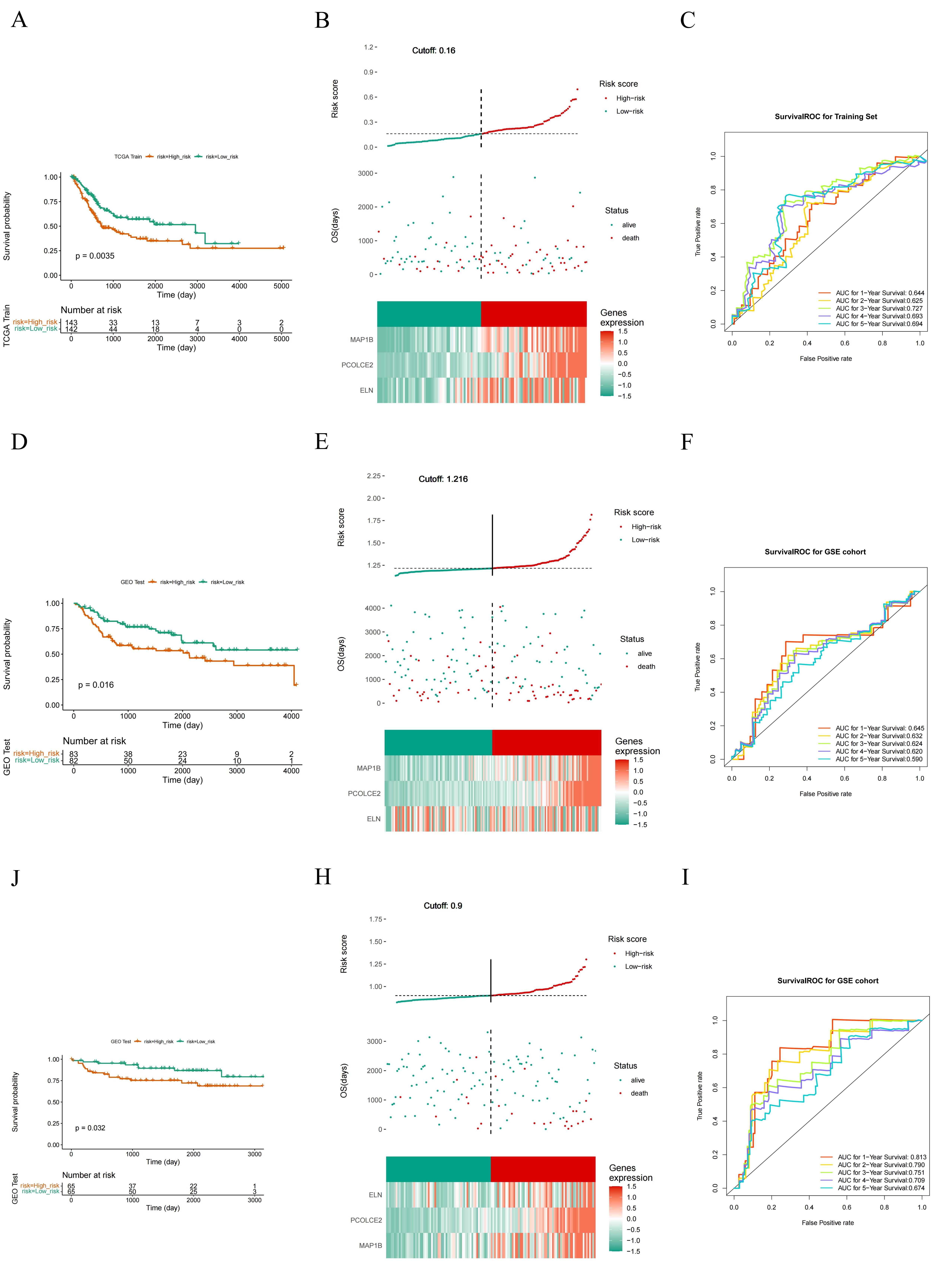

Supplement: Supplementary file 6 — Additional file 6: Figure S6. Stratified survival analysis of risk models and clinical characteristics. [file 12967_2023_4056_MOESM6_ESM.jpg]

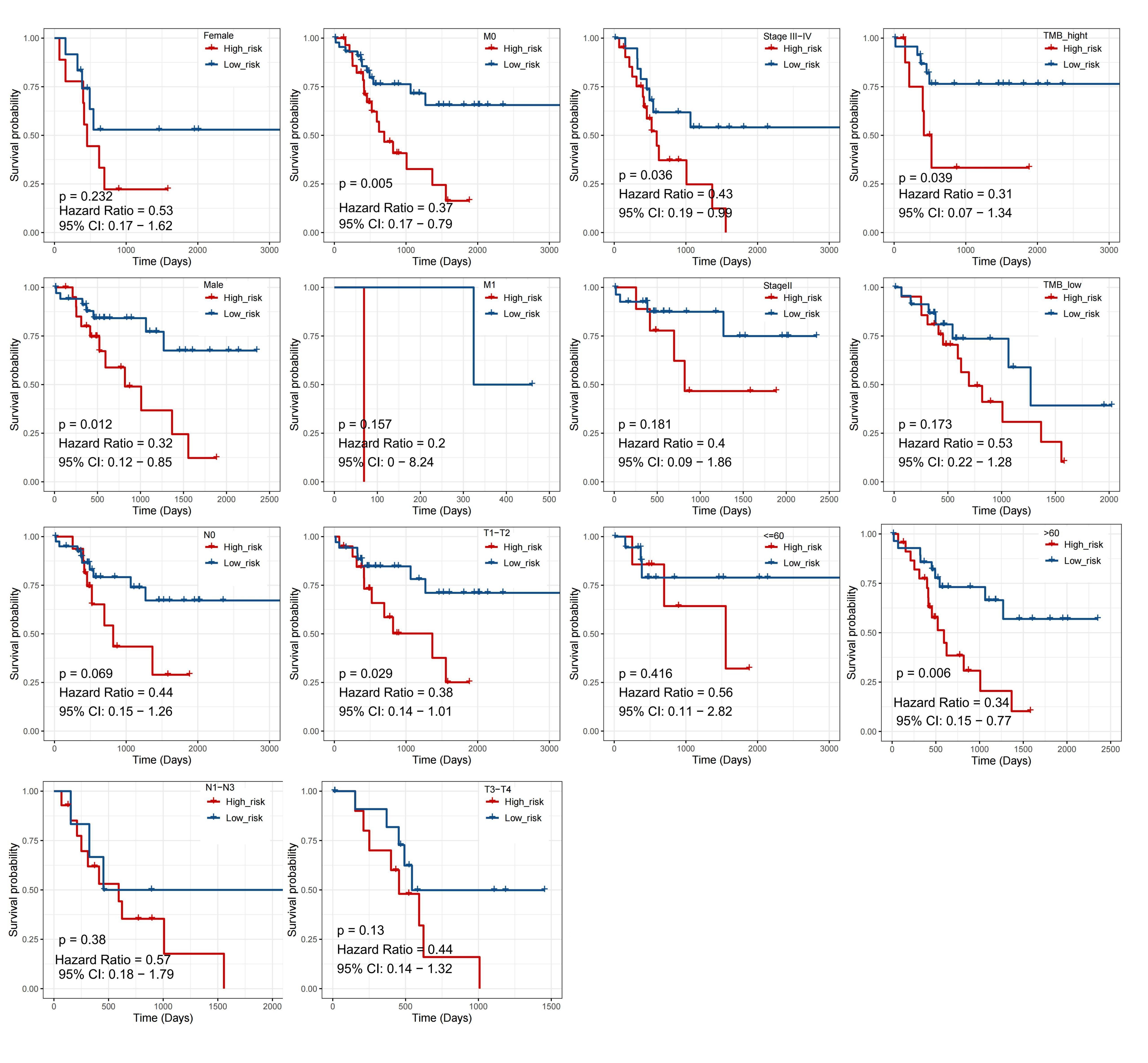

Supplement: Supplementary file 7 — Additional file 7: Figure S7. Validation of (A-C) internal test set risk models; (D-F) risk model evaluation in GSE13607; (J-I)) risk model evaluation in GSE32548. [file 12967_2023_4056_MOESM7_ESM.jpg]

# T cell\_inflamed GEP

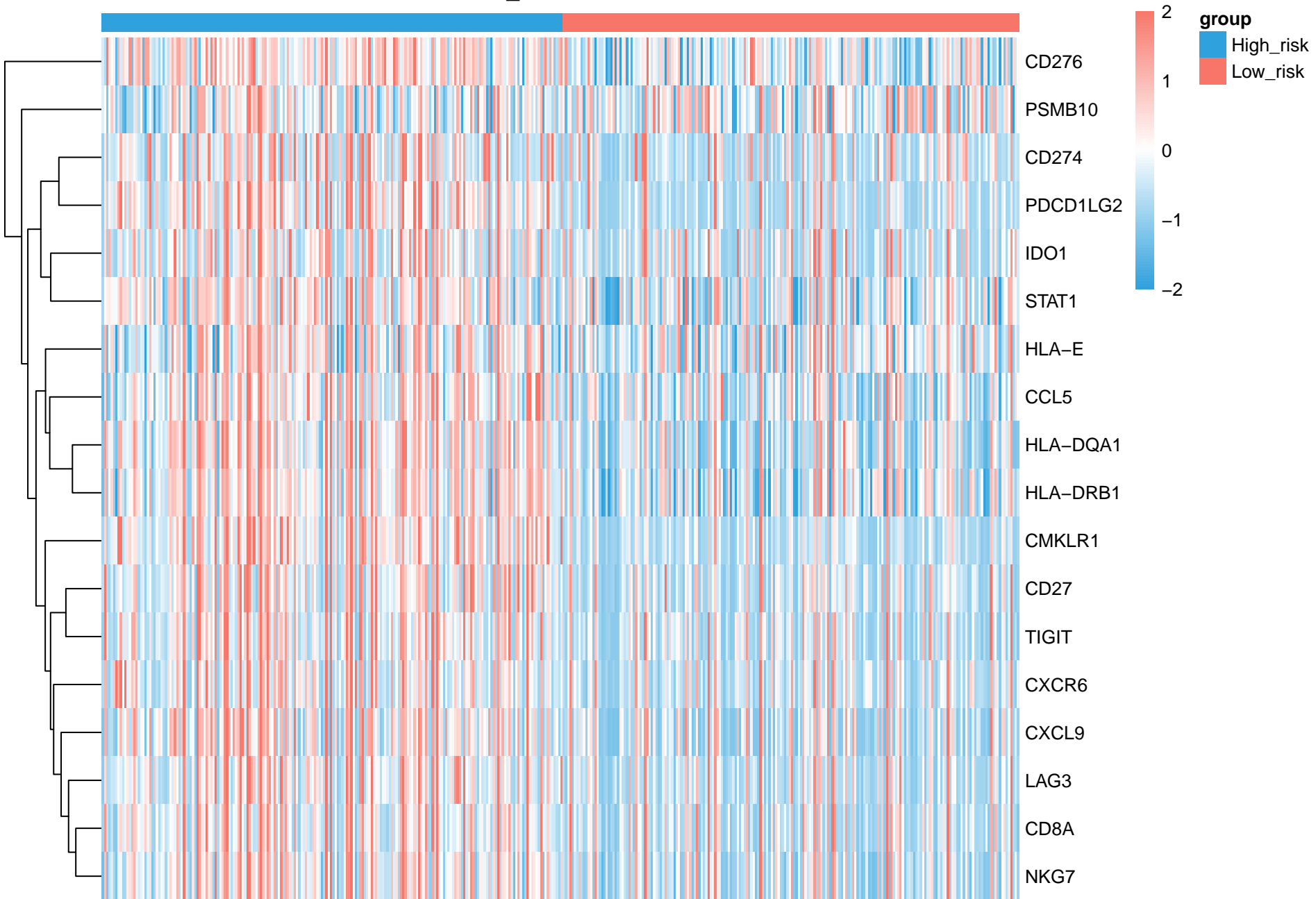

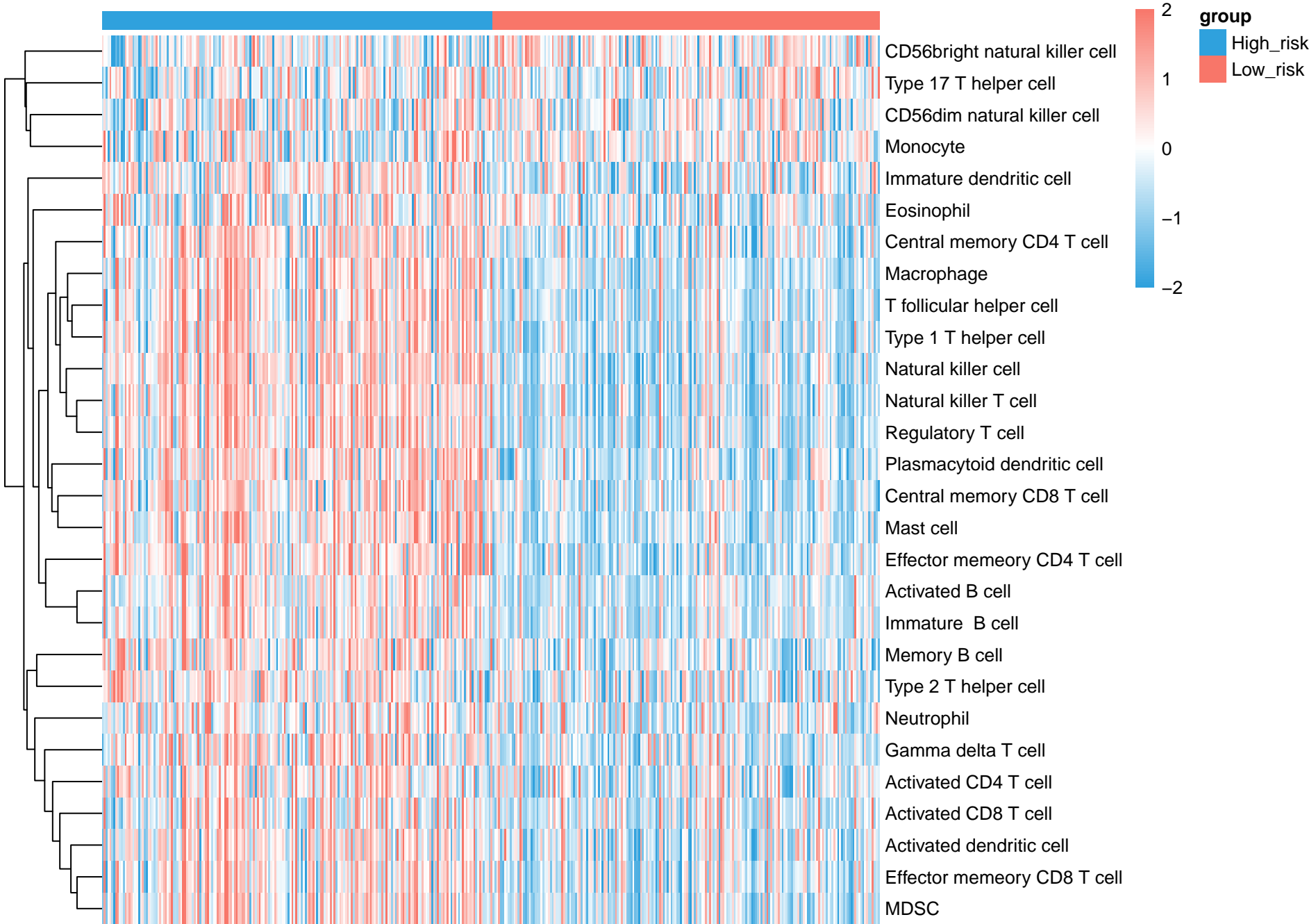

Supplement: Supplementary file 8 — Additional file 8: Figure S8. Heatmaps of immune cells and 16 differential GEP genes between high- and low-risk groups. [file 12967_2023_4056_MOESM8_ESM.pdf]
