# Supplementary material for: Comprehensive analysis of scRNA-Seq and bulk RNA-Seq reveals dynamic changes in the tumor immune microenvironment of bladder cancer and establishes a prognostic model
Source: J Transl Med. 2023 Mar 27;21:223. doi: 10.1186/s12967-023-04056-z (PMC10044739; doi:10.1186/s12967-023-04056-z)
Supplement: Supplementary file 9 — Additional file 9: Table S1 Patients information and sequencing statistics in GSE12984 dataset. [file 12967_2023_4056_MOESM9_ESM.docx]

Supplementary Table 1 Patients information and sequencing statistics in GSE12984 dataset.

| **ID** | **Hospital** | **Age/Sex** | **Ethnic** | **Histology** | **Smoker/Dringker** | **Height（cm）/Weight（kg）** | **Sample** | **Date** | **Sequencing Saturation(%)** | **Median. number of detected genes** | **Number of cells** | **Mean Reads per Cell** |
| --- | --- | --- | --- | --- | --- | --- | --- | --- | --- | --- | --- | --- |
|  |  |  |  |  |  |  |  |  |  |  |  |  |
| P1 | The first affiliated hospital of Guangxi Medical University | 48/male | The Zhuang nationality | Mucinous adenocarcinoma of the umbilical canal (invasive bladder) | N/N | 174/83 | normal bladder | 2018.8.7 | 90.9 | 2,336 | 345 | 245,241 |
|  |  |  |  |  |  |  |  |  |  |  |  |  |
|  |  |  |  |  |  |  |  |  |  |  |  |  |
|  |  |  |  |  |  |  |  |  |  |  |  |  |
|  |  |  |  |  |  |  |  |  |  |  |  |  |
|  |  |  |  |  |  |  |  |  |  |  |  |  |
|  |  |  |  |  |  |  |  |  |  |  |  |  |
|  |  |  |  |  |  |  |  |  |  |  |  |  |
|  |  |  |  |  |  |  |  |  |  |  |  |  |
|  |  |  |  |  |  |  |  |  |  |  |  |  |
|  |  |  |  |  |  |  |  |  |  |  |  |  |
| P2 | Affiliated tumor hospital of Guangxi Medical University | 35/female | The Han nationality | Highly invasive papillary urothelial carcinoma of the bladder | N/N | 157/50 | normal bladder | 2018.8.9 | 88.5 | 2,033 | 3,560 | 170,932 |
|  |  |  |  |  |  |  |  |  |  |  |  |  |
|  |  |  |  |  |  |  |  |  |  |  |  |  |
|  |  |  |  |  |  |  |  |  |  |  |  |  |
|  |  |  |  |  |  |  |  |  |  |  |  |  |
|  |  |  |  |  |  |  |  |  |  |  |  |  |
|  |  |  |  |  |  |  |  |  |  |  |  |  |
|  |  |  |  |  |  |  |  |  |  |  |  |  |
| P3 | The first affiliated hospital of Guangxi Medical University | 47/male | The Zhuang nationality | Highly invasive papillary urothelial carcinoma of the bladder | Y/Y | 165/64 | normal bladder | 2018.4.17 | 71.2 | 1,396 | 9,590 | 34,578 |
|  |  |  |  |  |  |  |  |  |  |  |  |  |
|  |  |  |  |  |  |  |  |  |  |  |  |  |
|  |  |  |  |  |  |  |  |  |  |  |  |  |
|  |  |  |  |  |  |  |  |  |  |  |  |  |
|  |  |  |  |  |  |  |  |  |  |  |  |  |
|  |  |  |  |  |  |  |  |  |  |  |  |  |
|  |  |  |  |  |  |  |  |  |  |  |  |  |
|  |  |  |  |  |  |  |  |  |  |  |  |  |
|  |  |  |  |  |  |  |  |  |  |  |  |  |
