# Supplementary material for: Comprehensive analysis of scRNA-Seq and bulk RNA-Seq reveals dynamic changes in the tumor immune microenvironment of bladder cancer and establishes a prognostic model
Source: J Transl Med. 2023 Mar 27;21:223. doi: 10.1186/s12967-023-04056-z (PMC10044739; doi:10.1186/s12967-023-04056-z)
Supplement: Supplementary file 10 — Additional file 10: Table S2 474 significantly different marker genes. [file 12967_2023_4056_MOESM10_ESM.pdf]

p_val	avg_log2F(	pct.1	pct.2	p_val_adj	cluster	gene	
CCL23	0	2.651228	0.634	0.078	0	Endotheli	CCL23
EMCN	0	2.2278348	0.821	0.075	0	Endotheli	EMCN
PCAT19	6.29E-267	2.2706199	0.844	0.159	1.26E-263	Endotheli	PCAT19
IFI27	3.36E-262	3.9424235	0.966	0.244	6.73E-259	Endotheli	IFI27
AQP1	4.88E-251	3.0063653	0.891	0.177	9.76E-248	Endotheli	AQP1
ECSCR.1	1.21E-250	2.3108478	0.838	0.093	2.41E-247	Endotheli	ECSCR.1
RNASE1	3.44E-243	2.6816132	0.877	0.159	6.88E-240	Endotheli	RNASE1
VWF	3.22E-240	2.8454161	0.844	0.146	6.44E-237	Endotheli	VWF
HLA-DRB5	1.20E-233	2.9518823	0.972	0.389	2.39E-230	Endotheli	HLA-DRB5
RAMP2	3.18E-231	2.7690945	0.863	0.2	6.35E-228	Endotheli	RAMP2
HLA-DRB1	2.15E-227	3.089671	0.978	0.323	4.29E-224	Endotheli	HLA-DRB1
GNG11	1.48E-219	2.5380538	0.941	0.282	2.96E-216	Endotheli	GNG11
PECAM1	5.75E-217	2.1870867	0.793	0.109	1.15E-213	Endotheli	PECAM1
HLA-DRA	7.39E-217	2.4660482	0.969	0.281	1.48E-213	Endotheli	HLA-DRA
C2CD4B	2.66E-213	2.9524438	0.799	0.246	5.32E-210	Endotheli	C2CD4B
TM4SF1	9.95E-212	3.5383685	0.989	0.528	1.99E-208	Endotheli	TM4SF1
ACKR1	2.49E-203	4.743122	0.832	0.23	4.99E-200	Endotheli	ACKR1
SELE	7.13E-200	3.7220063	0.782	0.239	1.43E-196	Endotheli	SELE
CD74	3.54E-196	2.9040327	0.994	0.6	7.07E-193	Endotheli	CD74
CAV1	1.42E-193	2.5561463	0.933	0.322	2.84E-190	Endotheli	CAV1
CLDN5	7.45E-187	2.6902631	0.732	0.114	1.49E-183	Endotheli	CLDN5
HLA-DQB1	2.69E-186	1.7972535	0.855	0.217	5.39E-183	Endotheli	HLA-DQB1
HLA-DPA1	4.63E-179	1.7838243	0.849	0.261	9.25E-176	Endotheli	HLA-DPA1
HLA-DPB1	6.77E-179	1.6985661	0.855	0.245	1.35E-175	Endotheli	HLA-DPB1
IFITM2	2.73E-178	1.9580479	0.939	0.336	5.46E-175	Endotheli	IFITM2
PLVAP	4.02E-178	2.1259452	0.721	0.056	8.03E-175	Endotheli	PLVAP
TCF4	8.93E-178	2.0424056	0.83	0.209	1.79E-174	Endotheli	TCF4
CXorf36	1.07E-177	1.411777	0.62	0.064	2.15E-174	Endotheli	CXorf36
HLA-DQA1	8.58E-177	1.6131452	0.768	0.134	1.72E-173	Endotheli	HLA-DQA1
SPARCL1	2.24E-175	2.3425083	0.955	0.361	4.48E-172	Endotheli	SPARCL1
ICAM1	6.94E-173	2.5485211	0.866	0.329	1.39E-169	Endotheli	ICAM1
IGFBP7	4.58E-168	2.4038812	0.983	0.537	9.16E-165	Endotheli	IGFBP7
ADAMTS9	5.75E-167	2.2962547	0.76	0.226	1.15E-163	Endotheli	ADAMTS9
ADGRL4	3.41E-163	1.5624201	0.626	0.095	6.81E-160	Endotheli	ADGRL4
CD93	8.28E-159	1.6731642	0.656	0.082	1.66E-155	Endotheli	CD93
VCAM1	1.85E-156	3.1750163	0.768	0.29	3.70E-153	Endotheli	VCAM1
SOX17	1.52E-154	1.2818282	0.536	0.1	3.04E-151	Endotheli	SOX17
GIMAP7	3.01E-149	1.8527124	0.642	0.098	6.02E-146	Endotheli	GIMAP7
CD200	5.92E-142	1.4005797	0.637	0.114	1.18E-138	Endotheli	CD200
TSPAN7	5.19E-141	1.729538	0.707	0.218	1.04E-137	Endotheli	TSPAN7
IFITM1	1.08E-140	1.6848541	0.74	0.246	2.15E-137	Endotheli	IFITM1
NNMT	6.63E-137	1.8470259	0.835	0.297	1.33E-133	Endotheli	NNMT
VIM	1.20E-135	1.5663884	0.994	0.403	2.41E-132	Endotheli	VIM
TMEM255B	1.24E-134	1.2406437	0.623	0.145	2.49E-131	Endotheli	TMEM255B
MYCT1	9.30E-134	1.0017154	0.52	0.067	1.86E-130	Endotheli	MYCT1
CALCRL	1.02E-132	1.6163621	0.659	0.108	2.04E-129	Endotheli	CALCRL
NPDC1	4.37E-132	1.7590539	0.777	0.251	8.75E-129	Endotheli	NPDC1
RCAN1	9.10E-131	1.7296204	0.723	0.184	1.82E-127	Endotheli	RCAN1
MCTP1	2.76E-128	1.3053751	0.628	0.18	5.52E-125	Endotheli	MCTP1
HLA-DMA	8.25E-128	1.5404673	0.743	0.227	1.65E-124	Endotheli	HLA-DMA

RND1	8.53E-126	1.738191	0.642	0.165	1.71E-122	Endotheli	RND1
IFITM3	2.78E-123	1.5846337	0.992	0.67	5.56E-120	Endotheli	IFITM3
ARHGAP29	2.81E-123	1.5390612	0.684	0.204	5.61E-120	Endotheli	ARHGAP29
SOX7	5.56E-123	1.3283656	0.603	0.143	1.11E-119	Endotheli	SOX7
RAMP3	3.56E-121	1.9011376	0.659	0.176	7.11E-118	Endotheli	RAMP3
STC1	7.37E-121	2.8565775	0.687	0.249	1.47E-117	Endotheli	STC1
ENG	5.31E-113	1.3097992	0.654	0.158	1.06E-109	Endotheli	ENG
NRN1	4.48E-112	1.1631582	0.511	0.08	8.97E-109	Endotheli	NRN1
ANGPT2	1.11E-111	1.4788921	0.55	0.121	2.22E-108	Endotheli	ANGPT2
ZNF385D	2.50E-111	1.6980139	0.634	0.179	5.01E-108	Endotheli	ZNF385D
IL6	4.10E-111	2.7918448	0.651	0.174	8.20E-108	Endotheli	IL6
SLC2A3	2.49E-110	1.6284175	0.696	0.196	4.98E-107	Endotheli	SLC2A3
MGST1	4.98E-109	-1.877363	0.397	0.853	9.96E-106	Endotheli	MGST1
CSF3	1.05E-106	2.4650963	0.67	0.293	2.10E-103	Endotheli	CSF3
FLT1	3.29E-106	1.1555852	0.531	0.095	6.58E-103	Endotheli	FLT1
FAM167B	1.09E-105	1.357732	0.561	0.167	2.19E-102	Endotheli	FAM167B
ADAMTS4	2.64E-99	1.3573787	0.626	0.209	5.28E-96	Endotheli	ADAMTS4
UPP1	6.99E-99	1.3948682	0.715	0.292	1.40E-95	Endotheli	UPP1
JAM2	4.74E-97	1.3817215	0.668	0.279	9.49E-94	Endotheli	JAM2
BCAM	2.65E-96	1.3930345	0.751	0.343	5.31E-93	Endotheli	BCAM
TNFSF10	1.27E-95	1.6352071	0.668	0.246	2.54E-92	Endotheli	TNFSF10
LY96	7.65E-95	1.2364722	0.626	0.201	1.53E-91	Endotheli	LY96
CNKSR3	5.14E-93	1.2997656	0.609	0.241	1.03E-89	Endotheli	CNKSR3
RHOJ	7.33E-89	1.1750116	0.57	0.2	1.47E-85	Endotheli	RHOJ
MT2A	6.56E-88	1.7536108	0.832	0.441	1.31E-84	Endotheli	MT2A
CCL2	9.14E-88	2.1304787	0.743	0.303	1.83E-84	Endotheli	CCL2
MEF2C	3.04E-86	1.2388495	0.659	0.257	6.08E-83	Endotheli	MEF2C
A2M	4.37E-85	1.2395102	0.804	0.41	8.74E-82	Endotheli	A2M
S1PR1	7.72E-85	1.1238405	0.545	0.196	1.54E-81	Endotheli	S1PR1
CXCL2	6.04E-84	2.2345501	0.807	0.453	1.21E-80	Endotheli	CXCL2
IL3RA	2.80E-81	1.107187	0.567	0.247	5.61E-78	Endotheli	IL3RA
FXYD3	1.89E-80	-1.941941	0.425	0.85	3.77E-77	Endotheli	FXYD3
FNIP2	1.31E-79	1.26549	0.69	0.302	2.61E-76	Endotheli	FNIP2
C1orf54	1.42E-78	1.0129749	0.595	0.207	2.84E-75	Endotheli	C1orf54
YPEL2	1.53E-78	1.1692188	0.603	0.254	3.05E-75	Endotheli	YPEL2
INSIG1	2.32E-77	1.2759292	0.735	0.378	4.64E-74	Endotheli	INSIG1
ESAM	1.57E-75	1.3230904	0.564	0.218	3.14E-72	Endotheli	ESAM
AQP3	8.39E-75	-1.924972	0.553	0.906	1.68E-71	Endotheli	AQP3
SELP	2.34E-74	1.2393511	0.503	0.117	4.69E-71	Endotheli	SELP
CSRP2	2.21E-73	1.3000715	0.651	0.305	4.41E-70	Endotheli	CSRP2
PTRF	2.54E-72	1.0019288	0.757	0.326	5.07E-69	Endotheli	PTRF
SOCS3	1.24E-71	1.6238831	0.765	0.422	2.48E-68	Endotheli	SOCS3
SPRY1	1.64E-71	1.8858208	0.642	0.259	3.28E-68	Endotheli	SPRY1
KRT19	2.02E-71	-2.142143	0.592	0.898	4.04E-68	Endotheli	KRT19
PALMD	3.82E-71	1.1998018	0.528	0.162	7.63E-68	Endotheli	PALMD
THBD	1.78E-70	1.2790803	0.645	0.276	3.55E-67	Endotheli	THBD
CD34	9.81E-70	1.0601834	0.556	0.162	1.96E-66	Endotheli	CD34
LY6D	1.69E-69	-2.097141	0.461	0.842	3.39E-66	Endotheli	LY6D
COL15A1	4.58E-69	1.1159484	0.615	0.27	9.15E-66	Endotheli	COL15A1
DUSP6	6.65E-69	1.0864983	0.615	0.24	1.33E-65	Endotheli	DUSP6
AGR2	1.88E-67	-1.81423	0.433	0.823	3.76E-64	Endotheli	AGR2

KLF2	7.37E-66	1.3050493	0.648	0.281	1.47E-62	Endotheli	KLF2
HPGD	8.06E-64	-1.59321	0.369	0.743	1.61E-60	Endotheli	HPGD
ARL4A	7.10E-63	1.366894	0.628	0.297	1.42E-59	Endotheli	ARL4A
CYP1B1	1.99E-62	1.4037417	0.511	0.175	3.98E-59	Endotheli	CYP1B1
HSPG2	4.62E-62	1.002473	0.598	0.22	9.24E-59	Endotheli	HSPG2
SFN	2.50E-61	-1.761701	0.466	0.827	5.00E-58	Endotheli	SFN
EZR	1.69E-60	-1.277491	0.492	0.832	3.38E-57	Endotheli	EZR
KRT8	1.04E-58	-1.510696	0.494	0.83	2.09E-55	Endotheli	KRT8
S100A4	1.27E-58	-1.877165	0.447	0.751	2.54E-55	Endotheli	S100A4
ADAMTS1	4.49E-58	1.2099241	0.564	0.198	8.98E-55	Endotheli	ADAMTS1
GPX2	4.99E-58	-1.427448	0.436	0.816	9.97E-55	Endotheli	GPX2
KRT18	1.24E-57	-1.441861	0.444	0.816	2.47E-54	Endotheli	KRT18
C19orf33	1.32E-57	-1.401047	0.411	0.79	2.63E-54	Endotheli	C19orf33
CFD	3.08E-57	-3.082278	0.332	0.686	6.16E-54	Endotheli	CFD
KRT13	6.19E-56	-1.882546	0.553	0.868	1.24E-52	Endotheli	KRT13
AKR1C2	1.87E-54	-1.400234	0.472	0.806	3.75E-51	Endotheli	AKR1C2
CLDN4	5.26E-50	-2.017136	0.433	0.764	1.05E-46	Endotheli	CLDN4
GPRC5A	9.95E-50	-1.138158	0.455	0.769	1.99E-46	Endotheli	GPRC5A
KLF5	1.55E-49	-1.284968	0.422	0.768	3.11E-46	Endotheli	KLF5
SMIM22	1.62E-49	-1.347696	0.38	0.738	3.25E-46	Endotheli	SMIM22
KRT7	4.07E-49	-1.564295	0.355	0.723	8.15E-46	Endotheli	KRT7
SPINT2	4.02E-47	-1.297382	0.422	0.757	8.04E-44	Endotheli	SPINT2
DHRS2	2.47E-43	-1.659521	0.453	0.76	4.95E-40	Endotheli	DHRS2
SERPINB5	8.18E-42	-1.054485	0.399	0.731	1.64E-38	Endotheli	SERPINB5
HN1	1.06E-41	-1.010216	0.321	0.69	2.12E-38	Endotheli	HN1
LYPD3	4.95E-39	-1.075226	0.254	0.57	9.89E-36	Endotheli	LYPD3
ANXA10	5.58E-39	-1.078144	0.355	0.659	1.12E-35	Endotheli	ANXA10
FAM3B	3.39E-38	-1.046532	0.363	0.671	6.77E-35	Endotheli	FAM3B
LAMB3	4.60E-36	-1.023802	0.391	0.701	9.20E-33	Endotheli	LAMB3
PTGDS	0	3.7084805	0.847	0.125	0	Fibroblas	PTGDS
PLA2G2A	0	3.3046148	0.685	0.155	0	Fibroblas	PLA2G2A
APOE	0	3.5003441	0.825	0.162	0	Fibroblas	APOE
CCL11	0	1.3848068	0.77	0.123	0	Fibroblas	CCL11
SFRP4	0	2.5848317	0.605	0.052	0	Fibroblas	SFRP4
PCP4	0	-1.658295	0.686	0.223	0	Fibroblas	PCP4
IGHG1	0	-2.160151	0.655	0.177	0	Fibroblas	IGHG1
APOD	0	2.0857326	0.843	0.174	0	Fibroblas	APOD
MYLK	0	-1.190416	0.701	0.232	0	Fibroblas	MYLK
MYH11	0	-1.216996	0.725	0.259	0	Fibroblas	MYH11
MT2A1	0	2.0212149	0.806	0.328	0	Fibroblas	MT2A
SFRP1	0	2.9111197	0.889	0.05	0	Fibroblas	SFRP1
CHRDL2	0	1.2314591	0.791	0.146	0	Fibroblas	CHRDL2
IGHA1	0	-1.506902	0.598	0.138	0	Fibroblas	IGHA1
MFAP5	0	3.312343	0.75	0.077	0	Fibroblas	MFAP5
TNFAIP6	0	3.1141523	0.802	0.109	0	Fibroblas	TNFAIP6
PCOLCE2	0	2.195435	0.735	0.081	0	Fibroblas	PCOLCE2
AGR21	0	-2.63162	0.582	0.893	0	Fibroblas	AGR2
PI16	0	2.5511948	0.723	0.092	0	Fibroblas	PI16
CTGF	0	1.6021967	0.668	0.085	0	Fibroblas	CTGF
POSTN	0	1.2101334	0.64	0.093	0	Fibroblas	POSTN
SERPINE2	0	2.9724156	0.855	0.136	0	Fibroblas	SERPINE2

KRT71	0	-2.228122	0.459	0.803	0	Fibroblas	KRT7
A2M1	0	1.6630751	0.9	0.253	0	Fibroblas	A2M
IGLC2	0	-2.053412	0.516	0.052	0	Fibroblas	IGLC2
CTHRC1	0	1.5168443	0.652	0.076	0	Fibroblas	CTHRC1
MEG3	0	2.248989	0.846	0.063	0	Fibroblas	MEG3
IGFBP5	0	3.1189015	0.933	0.42	0	Fibroblas	IGFBP5
IGFBP6	0	3.5028309	0.869	0.147	0	Fibroblas	IGFBP6
IGHM	0	-1.093852	0.652	0.15	0	Fibroblas	IGHM
SFRP2	0	4.4164523	0.936	0.112	0	Fibroblas	SFRP2
SRPX	0	1.4606192	0.831	0.058	0	Fibroblas	SRPX
TSTD1	0	-1.371031	0.265	0.647	0	Fibroblas	TSTD1
ATP1B1	0	-1.275924	0.287	0.591	0	Fibroblas	ATP1B1
BDKRB1	0	1.4675594	0.607	0.136	0	Fibroblas	BDKRB1
C11orf96	0	2.01182	0.864	0.465	0	Fibroblas	C11orf96
SMPDL3A	0	1.24155	0.822	0.137	0	Fibroblas	SMPDL3A
SOWAHC	0	-1.299318	0.267	0.605	0	Fibroblas	SOWAHC
TIMP1	0	3.5534666	0.991	0.673	0	Fibroblas	TIMP1
CILP	0	1.4925777	0.668	0.056	0	Fibroblas	CILP
MGP	0	5.386015	0.988	0.276	0	Fibroblas	MGP
DPT	0	2.9184114	0.836	0.053	0	Fibroblas	DPT
BASP1	0	1.4131067	0.793	0.166	0	Fibroblas	BASP1
CCDC80	0	4.1603701	0.937	0.094	0	Fibroblas	CCDC80
FGF7	0	1.3280437	0.666	0.045	0	Fibroblas	FGF7
RBP1	0	1.6936301	0.881	0.119	0	Fibroblas	RBP1
CD248	0	1.5195228	0.715	0.056	0	Fibroblas	CD248
FN1	0	1.540171	0.853	0.147	0	Fibroblas	FN1
ADH1B	0	1.7825533	0.769	0.065	0	Fibroblas	ADH1B
TIMP3	0	2.2707042	0.895	0.274	0	Fibroblas	TIMP3
VCAN	0	2.19523	0.86	0.069	0	Fibroblas	VCAN
C7	0	1.5606817	0.777	0.063	0	Fibroblas	C7
OGN	0	2.1636335	0.807	0.055	0	Fibroblas	OGN
BGN	0	2.1181429	0.844	0.105	0	Fibroblas	BGN
TMEM176B	0	1.5651935	0.812	0.145	0	Fibroblas	TMEM176B
EMP3	0	2.2485615	0.924	0.194	0	Fibroblas	EMP3
COL6A3	0	3.2433583	0.967	0.151	0	Fibroblas	COL6A3
IGF1	0	2.2593093	0.784	0.06	0	Fibroblas	IGF1
TCF21	0	1.5688606	0.843	0.123	0	Fibroblas	TCF21
CYR61	0	1.3028396	0.646	0.136	0	Fibroblas	CYR61
C3	0	1.8755212	0.749	0.095	0	Fibroblas	C3
HTRA3	0	1.175874	0.592	0.063	0	Fibroblas	HTRA3
LINC01082	0	1.0444682	0.79	0.125	0	Fibroblas	LINC01082
MMP23B	0	1.6761918	0.814	0.122	0	Fibroblas	MMP23B
MATN2	0	1.6137598	0.818	0.101	0	Fibroblas	MATN2
EFEMP1	0	2.8817072	0.855	0.077	0	Fibroblas	EFEMP1
ABI3BP	0	1.5256339	0.724	0.047	0	Fibroblas	ABI3BP
HTRA1	0	1.7545665	0.865	0.101	0	Fibroblas	HTRA1
ITM2A	0	2.3637313	0.846	0.127	0	Fibroblas	ITM2A
PLAT	0	1.3838696	0.747	0.225	0	Fibroblas	PLAT
COL1A1	0	4.4482216	0.973	0.163	0	Fibroblas	COL1A1
THY1	0	2.749879	0.878	0.113	0	Fibroblas	THY1
RARRES2	0	2.1984658	0.835	0.136	0	Fibroblas	RARRES2

TMEM176A	0	1.2457206	0.685	0.057	0	Fibroblas	TMEM176A
SERPINF1	0	3.6812893	0.96	0.102	0	Fibroblas	SERPINF1
LUM	0	5.4742675	0.978	0.209	0	Fibroblas	LUM
CTSK	0	2.1853777	0.88	0.063	0	Fibroblas	CTSK
SPON2	0	2.353187	0.895	0.084	0	Fibroblas	SPON2
PDPN	0	1.1500692	0.646	0.123	0	Fibroblas	PDPN
PLPP3	0	1.4791408	0.8	0.145	0	Fibroblas	PLPP3
FBLN2	0	1.5801163	0.762	0.058	0	Fibroblas	FBLN2
COL8A1	0	1.1477093	0.629	0.067	0	Fibroblas	COL8A1
MFAP4	0	2.8156155	0.885	0.1	0	Fibroblas	MFAP4
NNMT1	0	1.610783	0.804	0.139	0	Fibroblas	NNMT
ELN	0	1.6582484	0.77	0.065	0	Fibroblas	ELN
C1R	0	3.2479591	0.963	0.126	0	Fibroblas	C1R
CALD1	0	1.6583073	0.995	0.254	0	Fibroblas	CALD1
COL14A1	0	2.1668118	0.898	0.045	0	Fibroblas	COL14A1
GSN	0	3.8421753	0.974	0.333	0	Fibroblas	GSN
FSTL1	0	2.4877422	0.887	0.157	0	Fibroblas	FSTL1
PLAC9	0	3.7678601	0.974	0.087	0	Fibroblas	PLAC9
VAMP8	0	-1.59629	0.321	0.703	0	Fibroblas	VAMP8
SLIT3	0	1.0485549	0.631	0.101	0	Fibroblas	SLIT3
PLTP	0	1.8387061	0.877	0.093	0	Fibroblas	PLTP
SOD3	0	2.8393689	0.943	0.121	0	Fibroblas	SOD3
IGFBP4	0	3.0646583	0.969	0.299	0	Fibroblas	IGFBP4
HSPB6	0	1.2755276	0.81	0.163	0	Fibroblas	HSPB6
FBLN1	0	4.2510161	0.972	0.328	0	Fibroblas	FBLN1
COL3A1	0	4.0290772	0.974	0.188	0	Fibroblas	COL3A1
PCOLCE	0	3.0799144	0.932	0.068	0	Fibroblas	PCOLCE
PTN	0	2.0330148	0.874	0.119	0	Fibroblas	PTN
MMP2	0	3.1944958	0.949	0.162	0	Fibroblas	MMP2
SPARC	0	3.6341137	0.981	0.186	0	Fibroblas	SPARC
CFD1	0	4.9472906	0.975	0.573	0	Fibroblas	CFD
LOXL1	0	1.0384713	0.603	0.098	0	Fibroblas	LOXL1
COL5A1	0	1.350008	0.699	0.044	0	Fibroblas	COL5A1
TFPI	0	1.0902823	0.883	0.338	0	Fibroblas	TFPI
OLFML3	0	1.7157681	0.872	0.103	0	Fibroblas	OLFML3
NUPR1	0	1.9569851	0.936	0.235	0	Fibroblas	NUPR1
ISLR	0	1.0660747	0.586	0.052	0	Fibroblas	ISLR
COL1A2	0	3.9610052	0.985	0.191	0	Fibroblas	COL1A2
CFH	0	1.7595198	0.872	0.318	0	Fibroblas	CFH
SDC2	0	1.6889092	0.811	0.115	0	Fibroblas	SDC2
CXCL12	0	1.4005215	0.833	0.047	0	Fibroblas	CXCL12
PRRX1	0	1.0375804	0.65	0.047	0	Fibroblas	PRRX1
GPNMB	0	1.2088701	0.87	0.3	0	Fibroblas	GPNMB
FBN1	0	1.9570787	0.757	0.053	0	Fibroblas	FBN1
QSOX1	0	1.0328189	0.839	0.297	0	Fibroblas	QSOX1
DCN	0	5.3920398	0.978	0.199	0	Fibroblas	DCN
FOS	0	1.5491417	0.869	0.464	0	Fibroblas	FOS
LGALS1	0	3.6376676	0.999	0.319	0	Fibroblas	LGALS1
IGFBP71	0	2.1975243	0.974	0.4	0	Fibroblas	IGFBP7
VIM1	0	3.172578	0.997	0.216	0	Fibroblas	VIM
C1S	0	2.8582549	0.94	0.082	0	Fibroblas	C1S

SPARCL11	0	2.1867304	0.956	0.174	0	Fibroblas	SPARCL1
GNG111	0	1.3064716	0.839	0.111	0	Fibroblas	GNG11
COL6A2	0	3.473322	0.992	0.162	0	Fibroblas	COL6A2
SERPING1	0	2.2612009	0.914	0.15	0	Fibroblas	SERPING1
TUBA1A	0	2.07315	0.913	0.214	0	Fibroblas	TUBA1A
COL6A1	0	2.5863105	0.94	0.116	0	Fibroblas	COL6A1
SELM	0	1.8529339	0.973	0.262	0	Fibroblas	SELM
CLEC11A	0	1.9767204	0.882	0.113	0	Fibroblas	CLEC11A
PMP22	0	2.1147523	0.869	0.096	0	Fibroblas	PMP22
JUN	0	1.4157297	0.914	0.554	0	Fibroblas	JUN
EGR1	0	1.0248485	0.773	0.242	0	Fibroblas	EGR1
IFITM21	0	1.7451325	0.898	0.161	0	Fibroblas	IFITM2
MFGE8	0	1.5463695	0.741	0.122	0	Fibroblas	MFGE8
TNXB	0	1.6140629	0.745	0.047	0	Fibroblas	TNXB
IFITM31	0	2.4706339	0.998	0.567	0	Fibroblas	IFITM3
PRKCDBP	0	1.7345726	0.85	0.108	0	Fibroblas	PRKCDBP
FXYD1	0	1.7859575	0.868	0.055	0	Fibroblas	FXYD1
SEPP1	0	1.2651641	0.877	0.323	0	Fibroblas	SEPP1
AEBP1	0	1.476945	0.787	0.075	0	Fibroblas	AEBP1
AKAP12	0	1.2394203	0.739	0.153	0	Fibroblas	AKAP12
TIMP2	0	1.7350803	0.86	0.162	0	Fibroblas	TIMP2
PLPP1	0	1.4335226	0.874	0.276	0	Fibroblas	PLPP1
PTRF1	0	1.1592484	0.782	0.182	0	Fibroblas	PTRF
COL5A2	0	1.4642512	0.721	0.082	0	Fibroblas	COL5A2
FGFR1	0	1.1742718	0.679	0.118	0	Fibroblas	FGFR1
GYPC	0	1.3968434	0.76	0.123	0	Fibroblas	GYPC
PRSS23	0	1.4218756	0.742	0.226	0	Fibroblas	PRSS23
COX7A1	0	1.2210434	0.747	0.134	0	Fibroblas	COX7A1
RCN3	0	1.2432087	0.796	0.084	0	Fibroblas	RCN3
CYBRD1	0	1.420935	0.729	0.068	0	Fibroblas	CYBRD1
PPIC	0	1.9974764	0.913	0.289	0	Fibroblas	PPIC
FBLN5	0	1.0957273	0.648	0.04	0	Fibroblas	FBLN5
ECM1	0	1.1935911	0.771	0.027	0	Fibroblas	ECM1
SERPINH1	0	1.6668138	0.872	0.164	0	Fibroblas	SERPINH1
EFEMP2	0	1.2836762	0.785	0.086	0	Fibroblas	EFEMP2
FCGRT	0	1.5067195	0.907	0.259	0	Fibroblas	FCGRT
TSPAN4	0	1.224361	0.78	0.094	0	Fibroblas	TSPAN4
GPX8	0	1.1689807	0.726	0.081	0	Fibroblas	GPX8
GADD45B	1.79E-305	1.3372725	0.792	0.407	3.59E-302	Fibroblas	GADD45B
RGS16	2.70E-294	1.0969972	0.613	0.159	5.40E-291	Fibroblas	RGS16
C1QTNF3	8.52E-292	1.1195461	0.531	0.04	1.70E-288	Fibroblas	C1QTNF3
GEM	3.56E-280	1.3410398	0.614	0.119	7.13E-277	Fibroblas	GEM
PRELP	1.21E-269	1.1179181	0.574	0.081	2.41E-266	Fibroblas	PRELP
LINC01133	3.50E-236	1.0725482	0.538	0.08	7.00E-233	Fibroblas	LINC01133
DES	2.16E-234	-2.554137	0.725	0.305	4.33E-231	Fibroblas	DES
CYGB	1.05E-229	1.0550533	0.56	0.101	2.10E-226	Fibroblas	CYGB
CCL21	5.65E-224	1.6663389	0.65	0.197	1.13E-220	Fibroblas	CCL2
GPX3	1.15E-222	1.264973	0.557	0.049	2.30E-219	Fibroblas	GPX3
CXCL14	1.30E-222	2.9807283	0.523	0.064	2.60E-219	Fibroblas	CXCL14
TPPP3	4.22E-189	1.4074543	0.539	0.122	8.44E-186	Fibroblas	TPPP3
ACTG2	3.86E-179	-2.470269	0.741	0.318	7.71E-176	Fibroblas	ACTG2

JCHAIN	1.18E-154	-1.911375	0.579	0.173	2.35E-151	Fibroblas	JCHAIN
CEBPB	3.98E-154	1.0061601	0.727	0.416	7.97E-151	Fibroblas	CEBPB
HLA-DPB11	1.91E-128	-1.134222	0.565	0.155	3.82E-125	Fibroblas	HLA-DPB1
IGKC	2.13E-31	-4.774407	0.616	0.312	4.26E-28	Fibroblas	IGKC
HLA-DRB51	7.91E-20	-1.412061	0.636	0.323	1.58E-16	Fibroblas	HLA-DRB5
PTGDS1	0	-3.384456	0.098	0.66	0	Epithelia	PTGDS
PLA2G2A1	0	-3.488188	0.11	0.586	0	Epithelia	PLA2G2A
ACTG21	0	-3.822972	0.239	0.734	0	Epithelia	ACTG2
APOE1	0	-3.227118	0.146	0.636	0	Epithelia	APOE
HLA-DPB12	0	-2.121772	0.088	0.543	0	Epithelia	HLA-DPB1
DES1	0	-3.490191	0.225	0.72	0	Epithelia	DES
CCL111	0	-1.08245	0.103	0.594	0	Epithelia	CCL11
SPINK1	0	3.01671	0.772	0.462	0	Epithelia	SPINK1
ACTA2	0	-3.14401	0.186	0.685	0	Epithelia	ACTA2
RGS161	0	-1.218725	0.106	0.553	0	Epithelia	RGS16
PCP41	0	-2.426553	0.137	0.677	0	Epithelia	PCP4
HLA-DPA11	0	-1.639108	0.068	0.615	0	Epithelia	HLA-DPA1
PSCA	0	2.3866389	0.725	0.365	0	Epithelia	PSCA
MYL9	0	-3.72894	0.257	0.824	0	Epithelia	MYL9
TPM1	0	-3.352459	0.272	0.783	0	Epithelia	TPM1
TPM2	0	-3.840053	0.26	0.848	0	Epithelia	TPM2
TAGLN	0	-4.270883	0.274	0.687	0	Epithelia	TAGLN
IGHG11	0	-2.776929	0.148	0.549	0	Epithelia	IGHG1
APOD1	0	-1.730086	0.156	0.656	0	Epithelia	APOD
CCL22	0	-3.226986	0.124	0.623	0	Epithelia	CCL2
MYLK1	0	-2.580644	0.157	0.671	0	Epithelia	MYLK
RGS5	0	-1.205171	0.136	0.596	0	Epithelia	RGS5
MYH111	0	-2.649355	0.179	0.705	0	Epithelia	MYH11
CNN1	0	-2.145741	0.148	0.57	0	Epithelia	CNN1
MT2A2	0	-2.991302	0.251	0.776	0	Epithelia	MT2A
SFRP11	0	-2.547183	0.026	0.657	0	Epithelia	SFRP1
CHRDL21	0	-1.009716	0.11	0.641	0	Epithelia	CHRDL2
FXYD31	0	2.6133945	0.988	0.598	0	Epithelia	FXYD3
IGHA11	0	-2.603336	0.102	0.508	0	Epithelia	IGHA1
MFAP51	0	-2.996083	0.051	0.575	0	Epithelia	MFAP5
TNFAIP61	0	-2.725821	0.089	0.611	0	Epithelia	TNFAIP6
SYNM	0	-1.156258	0.127	0.586	0	Epithelia	SYNM
PCOLCE21	0	-1.877751	0.062	0.557	0	Epithelia	PCOLCE2
AGR22	0	2.5030248	0.967	0.562	0	Epithelia	AGR2
RRAD	0	-1.07985	0.139	0.572	0	Epithelia	RRAD
PI161	0	-2.215307	0.07	0.554	0	Epithelia	PI16
CTGF1	0	-1.693478	0.047	0.542	0	Epithelia	CTGF
POSTN1	0	-1.065466	0.066	0.506	0	Epithelia	POSTN
SERPINE21	0	-2.60262	0.116	0.657	0	Epithelia	SERPINE2
SYNPO2	0	-1.417789	0.136	0.569	0	Epithelia	SYNPO2
KRT72	0	2.209376	0.871	0.459	0	Epithelia	KRT7
A2M2	0	-2.183523	0.168	0.83	0	Epithelia	A2M
SNCG	0	2.3895871	0.951	0.64	0	Epithelia	SNCG
TACSTD2	0	2.4835243	0.977	0.61	0	Epithelia	TACSTD2
CTHRC11	0	-1.397203	0.031	0.54	0	Epithelia	CTHRC1
MEG31	0	-1.923306	0.04	0.631	0	Epithelia	MEG3

CSRP1	0	-1.374185	0.183	0.639	0	Epithelia	CSRP1
SPINT21	0	1.7560154	0.873	0.544	0	Epithelia	SPINT2
IGFBP51	0	-2.917133	0.401	0.797	0	Epithelia	IGFBP5
IGFBP61	0	-3.224921	0.122	0.676	0	Epithelia	IGFBP6
IGHM1	0	-1.657247	0.114	0.547	0	Epithelia	IGHM
SFRP21	0	-4.129773	0.094	0.701	0	Epithelia	SFRP2
SRPX1	0	-1.234458	0.02	0.642	0	Epithelia	SRPX
RAMP1	0	-1.165665	0.106	0.712	0	Epithelia	RAMP1
TSTD11	0	1.3951099	0.714	0.28	0	Epithelia	TSTD1
C11orf961	0	-1.844539	0.423	0.803	0	Epithelia	C11orf96
PHLDA2	0	2.3560913	0.978	0.659	0	Epithelia	PHLDA2
LINC00152	0	-1.156249	0.154	0.625	0	Epithelia	LINC00152
SOWAHC1	0	1.2663477	0.643	0.314	0	Epithelia	SOWAHC
CILP1	0	-1.223645	0.03	0.513	0	Epithelia	CILP
MGP1	0	-5.832094	0.211	0.864	0	Epithelia	MGP
DPT1	0	-2.588117	0.035	0.613	0	Epithelia	DPT
BASP11	0	-1.395692	0.122	0.662	0	Epithelia	BASP1
CCDC801	0	-3.965446	0.074	0.699	0	Epithelia	CCDC80
FGF71	0	-1.13217	0.02	0.507	0	Epithelia	FGF7
RBP11	0	-1.55687	0.083	0.693	0	Epithelia	RBP1
CD2481	0	-1.253843	0.028	0.549	0	Epithelia	CD248
FN11	0	-2.458646	0.07	0.751	0	Epithelia	FN1
ADH1B1	0	-1.457214	0.049	0.57	0	Epithelia	ADH1B
TIMP31	0	-1.995188	0.259	0.72	0	Epithelia	TIMP3
VCAN1	0	-1.970235	0.037	0.657	0	Epithelia	VCAN
C71	0	-1.359678	0.031	0.599	0	Epithelia	C7
OGN1	0	-1.851862	0.031	0.603	0	Epithelia	OGN
BGN1	0	-1.795679	0.084	0.639	0	Epithelia	BGN
TMEM176B1	0	-1.382473	0.115	0.646	0	Epithelia	TMEM176B
EMP31	0	-2.509401	0.11	0.825	0	Epithelia	EMP3
COL6A31	0	-3.09384	0.095	0.795	0	Epithelia	COL6A3
IGF11	0	-1.956651	0.027	0.603	0	Epithelia	IGF1
TCF211	0	-1.295146	0.097	0.654	0	Epithelia	TCF21
CTSH	0	1.3364695	0.719	0.384	0	Epithelia	CTSH
C31	0	-1.57942	0.08	0.563	0	Epithelia	C3
MMP23B1	0	-1.466903	0.083	0.655	0	Epithelia	MMP23B
MATN21	0	-1.349591	0.069	0.639	0	Epithelia	MATN2
EFEMP11	0	-2.584758	0.049	0.649	0	Epithelia	EFEMP1
CAV11	0	-1.902257	0.137	0.662	0	Epithelia	CAV1
ABI3BP1	0	-1.242873	0.03	0.532	0	Epithelia	ABI3BP
HTRA11	0	-1.586875	0.056	0.692	0	Epithelia	HTRA1
ITM2A1	0	-2.176619	0.086	0.68	0	Epithelia	ITM2A
COL1A11	0	-4.654939	0.113	0.793	0	Epithelia	COL1A1
THY11	0	-2.439101	0.085	0.677	0	Epithelia	THY1
RARRES21	0	-2.289522	0.079	0.702	0	Epithelia	RARRES2
TMEM176A1	0	-1.071057	0.022	0.539	0	Epithelia	TMEM176A
TXNDC17	0	1.5922032	0.875	0.509	0	Epithelia	TXNDC17
SERPINF11	0	-3.360431	0.076	0.726	0	Epithelia	SERPINF1
LUM1	0	-5.520532	0.188	0.764	0	Epithelia	LUM
CTSK1	0	-1.880768	0.035	0.662	0	Epithelia	CTSK
SPON21	0	-2.021446	0.057	0.676	0	Epithelia	SPON2

PLPP31	0	-1.356192	0.106	0.653	0	Epithelia	PLPP3
FBLN21	0	-1.378947	0.032	0.578	0	Epithelia	FBLN2
MFAP41	0	-2.589537	0.068	0.685	0	Epithelia	MFAP4
NNMT2	0	-1.969264	0.061	0.715	0	Epithelia	NNMT
ELN1	0	-1.496241	0.029	0.602	0	Epithelia	ELN
C1R1	0	-3.002423	0.084	0.761	0	Epithelia	C1R
CALD11	0	-3.860477	0.152	0.922	0	Epithelia	CALD1
COL14A11	0	-1.828175	0.02	0.665	0	Epithelia	COL14A1
GSN1	0	-4.000793	0.262	0.883	0	Epithelia	GSN
FSTL11	0	-2.442109	0.098	0.748	0	Epithelia	FSTL1
PLAC91	0	-3.519841	0.055	0.74	0	Epithelia	PLAC9
VAMP81	0	1.4456252	0.756	0.359	0	Epithelia	VAMP8
PLTP1	0	-1.55649	0.058	0.681	0	Epithelia	PLTP
SOD31	0	-2.913984	0.059	0.779	0	Epithelia	SOD3
IGFBP41	0	-2.961041	0.247	0.838	0	Epithelia	IGFBP4
HSPB61	0	-1.88706	0.087	0.724	0	Epithelia	HSPB6
FBLN11	0	-3.886262	0.336	0.752	0	Epithelia	FBLN1
COL3A11	0	-4.442375	0.115	0.839	0	Epithelia	COL3A1
PCOLCE1	0	-2.713931	0.053	0.679	0	Epithelia	PCOLCE
PTN1	0	-1.702959	0.101	0.661	0	Epithelia	PTN
MMP21	0	-2.83592	0.148	0.717	0	Epithelia	MMP2
SPARC1	0	-4.050659	0.102	0.86	0	Epithelia	SPARC
COL5A11	0	-1.170636	0.023	0.522	0	Epithelia	COL5A1
OLFML31	0	-1.450236	0.061	0.693	0	Epithelia	OLFML3
NUPR11	0	-2.306214	0.16	0.831	0	Epithelia	NUPR1
GEM1	0	-1.345052	0.067	0.537	0	Epithelia	GEM
COL1A21	0	-4.510668	0.126	0.835	0	Epithelia	COL1A2
CFH1	0	-1.377805	0.318	0.693	0	Epithelia	CFH
SDC21	0	-1.516264	0.08	0.645	0	Epithelia	SDC2
CXCL121	0	-1.208847	0.026	0.613	0	Epithelia	CXCL12
CYSTM1	0	1.8635313	0.973	0.663	0	Epithelia	CYSTM1
GADD45B1	0	-1.672451	0.347	0.765	0	Epithelia	GADD45B
FBN11	0	-1.687724	0.029	0.569	0	Epithelia	FBN1
DCN1	0	-5.476808	0.162	0.787	0	Epithelia	DCN
FOS1	0	-1.511006	0.428	0.796	0	Epithelia	FOS
LGALS11	0	-4.991387	0.215	0.95	0	Epithelia	LGALS1
IGFBP72	0	-3.560515	0.322	0.914	0	Epithelia	IGFBP7
VIM2	0	-4.18817	0.107	0.923	0	Epithelia	VIM
C1S1	0	-2.624027	0.043	0.726	0	Epithelia	C1S
SPARCL12	0	-3.191911	0.073	0.868	0	Epithelia	SPARCL1
GNG112	0	-1.853198	0.035	0.727	0	Epithelia	GNG11
COL6A21	0	-3.627504	0.089	0.843	0	Epithelia	COL6A2
SERPING11	0	-2.115354	0.088	0.768	0	Epithelia	SERPING1
TUBA1A1	0	-2.435328	0.131	0.822	0	Epithelia	TUBA1A
COL6A11	0	-2.522518	0.058	0.767	0	Epithelia	COL6A1
SELM1	0	-2.778871	0.169	0.895	0	Epithelia	SELM
CLEC11A1	0	-1.646516	0.078	0.689	0	Epithelia	CLEC11A
PMP221	0	-1.975098	0.035	0.718	0	Epithelia	PMP22
1-Jun	0	-1.402587	0.532	0.833	0	Epithelia	JUN
EGR11	0	-1.279118	0.191	0.684	0	Epithelia	EGR1
IFITM22	0	-1.960427	0.107	0.749	0	Epithelia	IFITM2

KLF21	0	-1.091949	0.128	0.555	0	Epithelia	KLF2
MFGE81	0	-1.47662	0.086	0.6	0	Epithelia	MFGE8
TNXB1	0	-1.349061	0.025	0.556	0	Epithelia	TNXB
IFITM32	0	-2.830348	0.517	0.94	0	Epithelia	IFITM3
CTSL	0	-1.00704	0.19	0.714	0	Epithelia	CTSL
PRKCDBP1	0	-1.792141	0.052	0.702	0	Epithelia	PRKCDBP
FXYD11	0	-1.5483	0.032	0.643	0	Epithelia	FXYD1
COL4A2	0	-1.307657	0.105	0.629	0	Epithelia	COL4A2
SEPP11	0	-1.088773	0.329	0.688	0	Epithelia	SEPP1
AEBP11	0	-1.374563	0.032	0.626	0	Epithelia	AEBP1
AKAP121	0	-1.433725	0.083	0.664	0	Epithelia	AKAP12
TIMP21	0	-1.831336	0.095	0.742	0	Epithelia	TIMP2
PLPP11	0	-1.182296	0.258	0.711	0	Epithelia	PLPP1
PTRF2	0	-1.71294	0.1	0.721	0	Epithelia	PTRF
COL5A21	0	-1.311166	0.052	0.564	0	Epithelia	COL5A2
FGFR11	0	-1.114224	0.065	0.585	0	Epithelia	FGFR1
GYPC1	0	-1.435936	0.061	0.656	0	Epithelia	GYPC
PRSS231	0	-1.573043	0.172	0.663	0	Epithelia	PRSS23
COX7A11	0	-1.6509	0.058	0.673	0	Epithelia	COX7A1
CYBRD11	0	-1.241132	0.033	0.573	0	Epithelia	CYBRD1
PPIC1	0	-1.90453	0.239	0.794	0	Epithelia	PPIC
PKIG	0	-1.006462	0.132	0.631	0	Epithelia	PKIG
SERPINH11	0	-1.467905	0.124	0.709	0	Epithelia	SERPINH1
EFEMP21	0	-1.109954	0.043	0.63	0	Epithelia	EFEMP2
FCGRT1	0	-1.454033	0.219	0.762	0	Epithelia	FCGRT
TSPAN41	0	-1.141079	0.053	0.625	0	Epithelia	TSPAN4
ACTC1	1.53E-302	-1.522205	0.136	0.548	3.06E-299	Epithelia	ACTC1
CYR611	2.34E-284	-1.406717	0.099	0.541	4.69E-281	Epithelia	CYR61
CLIC4	7.95E-284	-1.044283	0.098	0.545	1.59E-280	Epithelia	CLIC4
MAP1B	3.69E-281	-1.053143	0.128	0.55	7.37E-278	Epithelia	MAP1B
BDKRB11	3.99E-278	-1.228715	0.108	0.5	7.98E-275	Epithelia	BDKRB1
SPRY11	5.90E-271	-1.100414	0.113	0.523	1.18E-267	Epithelia	SPRY1
COL4A1	5.46E-245	-1.140175	0.103	0.528	1.09E-241	Epithelia	COL4A1
PALLD	7.14E-244	-1.321478	0.153	0.567	1.43E-240	Epithelia	PALLD
PLAT1	2.72E-243	-1.07885	0.219	0.589	5.44E-240	Epithelia	PLAT
JCHAIN1	3.91E-230	-2.70234	0.136	0.507	7.82E-227	Epithelia	JCHAIN
FHL1	4.32E-218	-1.224551	0.049	0.501	8.64E-215	Epithelia	FHL1
PDLIM7	4.73E-198	-1.036312	0.139	0.539	9.45E-195	Epithelia	PDLIM7
CRYAB	1.45E-194	-1.190452	0.083	0.5	2.89E-191	Epithelia	CRYAB
FLNA	4.71E-192	-1.703683	0.174	0.568	9.42E-189	Epithelia	FLNA
HLA-DRB52	1.35E-138	-1.028754	0.276	0.612	2.71E-135	Epithelia	HLA-DRB5
DERL3	9.02E-103	2.1014336	0.864	0.139	1.80E-99	B_cells	DERL3
MZB1	2.03E-94	3.805643	1	0.263	4.06E-91	B_cells	MZB1
IGKV1-12	3.73E-92	4.0436898	0.795	0.138	7.45E-89	B_cells	IGKV1-12
JCHAIN2	9.68E-84	8.2976138	1	0.273	1.94E-80	B_cells	JCHAIN
IGKC1	1.13E-55	11.146061	0.932	0.387	2.25E-52	B_cells	IGKC
SRGN	1.61E-44	2.1370931	0.852	0.192	3.23E-41	B_cells	SRGN
CD79A	4.34E-44	2.1368271	0.727	0.12	8.67E-41	B_cells	CD79A
SEC11C	2.26E-41	1.8524031	0.852	0.287	4.53E-38	B_cells	SEC11C
IGKV3-15	4.40E-40	1.725619	0.716	0.262	8.79E-37	B_cells	IGKV3-15
ADIRF	4.08E-38	-3.178994	0.477	0.944	8.16E-35	B_cells	ADIRF

CYTIP	7.79E-37	1.0214472	0.636	0.153	1.56E-33	B_cells	CYTIP
IGHM2	1.94E-34	6.9972857	0.693	0.277	3.88E-31	B_cells	IGHM
PPDPF	3.39E-34	-1.924269	0.489	0.93	6.78E-31	B_cells	PPDPF
CD9	6.27E-34	-2.088227	0.511	0.948	1.25E-30	B_cells	CD9
CD27	9.00E-34	1.0122751	0.557	0.072	1.80E-30	B_cells	CD27
IGHG12	4.77E-33	8.2900865	0.705	0.298	9.54E-30	B_cells	IGHG1
IGHG4	1.86E-30	8.5941292	0.67	0.25	3.72E-27	B_cells	IGHG4
MGST11	2.96E-30	-2.134562	0.443	0.844	5.91E-27	B_cells	MGST1
DNAAF1	3.56E-29	1.4170049	0.534	0.123	7.11E-26	B_cells	DNAAF1
SOD2	9.38E-29	-2.26139	0.477	0.862	1.88E-25	B_cells	SOD2
LINC00152	1.11E-28	1.4648201	0.83	0.33	2.23E-25	B_cells	LINC00152
IER3	3.83E-28	-2.220049	0.466	0.862	7.66E-25	B_cells	IER3
PKM	5.79E-28	-1.638476	0.455	0.887	1.16E-24	B_cells	PKM
C9orf16	6.84E-26	-1.670593	0.557	0.893	1.37E-22	B_cells	C9orf16
LDHB	1.85E-24	-1.286564	0.409	0.888	3.70E-21	B_cells	LDHB
CYB5A	2.69E-24	-1.724328	0.409	0.804	5.39E-21	B_cells	CYB5A
SNCG1	3.62E-24	-2.578677	0.42	0.835	7.24E-21	B_cells	SNCG
KRT191	8.60E-24	-2.945209	0.58	0.892	1.72E-20	B_cells	KRT19
AGR23	2.60E-23	-2.399277	0.364	0.816	5.20E-20	B_cells	AGR2
FABP5	4.15E-23	-2.864732	0.534	0.899	8.29E-20	B_cells	FABP5
TACSTD21	4.31E-23	-2.323951	0.409	0.84	8.62E-20	B_cells	TACSTD2
KRT81	7.50E-23	-2.027362	0.375	0.824	1.50E-19	B_cells	KRT8
PHLDA21	8.83E-23	-2.223649	0.477	0.859	1.77E-19	B_cells	PHLDA2
KRT131	4.08E-22	-2.910372	0.511	0.862	8.16E-19	B_cells	KRT13
TXNDC171	5.99E-22	-1.707723	0.239	0.739	1.20E-18	B_cells	TXNDC17
HIST1H1C	1.12E-21	1.3348897	0.705	0.346	2.24E-18	B_cells	HIST1H1C
FXYD32	1.62E-21	-2.430501	0.511	0.841	3.24E-18	B_cells	FXYD3
IGFBP2	2.01E-21	-1.929687	0.398	0.791	4.02E-18	B_cells	IGFBP2
AKR1C21	2.74E-21	-1.913544	0.455	0.799	5.48E-18	B_cells	AKR1C2
KRT181	5.31E-21	-2.024873	0.42	0.809	1.06E-17	B_cells	KRT18
PERP	1.65E-20	-1.696926	0.432	0.8	3.30E-17	B_cells	PERP
IGHA12	2.20E-20	8.0838336	0.614	0.255	4.41E-17	B_cells	IGHA1
SPINT22	3.88E-20	-1.532851	0.295	0.751	7.76E-17	B_cells	SPINT2
C19orf331	4.67E-20	-1.796673	0.386	0.783	9.35E-17	B_cells	C19orf33
GPRC5A1	7.56E-20	-1.729398	0.432	0.762	1.51E-16	B_cells	GPRC5A
SFN1	1.17E-19	-2.488299	0.477	0.82	2.34E-16	B_cells	SFN
MDK	4.51E-19	-1.533782	0.307	0.674	9.02E-16	B_cells	MDK
LY6D1	6.36E-19	-2.892074	0.523	0.834	1.27E-15	B_cells	LY6D
MGST2	7.15E-19	-1.503632	0.375	0.74	1.43E-15	B_cells	MGST2
PHLDA1	7.95E-19	-1.834884	0.443	0.782	1.59E-15	B_cells	PHLDA1
ENO1	1.13E-18	-1.16895	0.341	0.757	2.26E-15	B_cells	ENO1
KLF51	4.80E-18	-1.780056	0.398	0.761	9.60E-15	B_cells	KLF5
EMP1	5.32E-18	-1.663556	0.33	0.671	1.06E-14	B_cells	EMP1
HEBP2	7.51E-18	-1.1719	0.307	0.739	1.50E-14	B_cells	HEBP2
GPX21	1.04E-17	-2.00341	0.466	0.809	2.08E-14	B_cells	GPX2
SMIM221	9.88E-16	-1.693365	0.386	0.73	1.98E-12	B_cells	SMIM22
ETS2	1.97E-15	-1.33626	0.375	0.696	3.94E-12	B_cells	ETS2
FAM162A	2.04E-15	-1.084198	0.386	0.734	4.07E-12	B_cells	FAM162A
SERPINB51	3.38E-15	-1.457574	0.386	0.724	6.77E-12	B_cells	SERPINB5
LRRFIP1	1.18E-14	-1.258676	0.33	0.725	2.35E-11	B_cells	LRRFIP1
IGFBP73	1.19E-14	-2.606519	0.205	0.551	2.39E-11	B_cells	IGFBP7

MAST4	2.61E-14	-1.320732	0.284	0.653	5.22E-11	B_cells	MAST4
TSC22D1	1.09E-13	-1.296151	0.261	0.64	2.18E-10	B_cells	TSC22D1
TIMP11	2.09E-12	-2.471624	0.455	0.758	4.18E-09	B_cells	TIMP1
LGALS12	0.0603353	-1.861244	0.83	0.493	1	B_cells	LGALS1
PTPRC	6.33E-149	1.6842981	0.727	0.102	1.27E-145	T_cells	PTPRC
SRGN1	4.95E-137	1.7653709	0.909	0.183	9.91E-134	T_cells	SRGN
CD7	1.41E-113	1.4989636	0.711	0.17	2.82E-110	T_cells	CD7
CCL5	1.10E-109	3.3364297	0.74	0.107	2.20E-106	T_cells	CCL5
CD52	1.55E-106	1.8667379	0.723	0.115	3.10E-103	T_cells	CD52
CD3D	4.39E-106	1.8417475	0.64	0.05	8.78E-103	T_cells	CD3D
TRBC1	2.76E-97	1.6034135	0.624	0.107	5.52E-94	T_cells	TRBC1
CXCR4	5.18E-93	1.7741436	0.669	0.107	1.04E-89	T_cells	CXCR4
HCST	5.66E-86	1.7988911	0.599	0.078	1.13E-82	T_cells	HCST
CD2	8.33E-83	1.5778222	0.607	0.146	1.67E-79	T_cells	CD2
CD69	8.19E-79	1.3522142	0.591	0.114	1.64E-75	T_cells	CD69
CD3E	1.43E-74	1.1688134	0.583	0.12	2.85E-71	T_cells	CD3E
TRBC2	1.87E-68	1.5633328	0.579	0.06	3.74E-65	T_cells	TRBC2
TRAC	5.81E-67	1.5756673	0.603	0.12	1.16E-63	T_cells	TRAC
AC092580.4	1.28E-66	1.3765119	0.57	0.079	2.55E-63	T_cells	AC092580.4
CREM	2.22E-66	1.3852195	0.888	0.452	4.45E-63	T_cells	CREM
CORO1A	2.20E-63	1.413063	0.55	0.102	4.41E-60	T_cells	CORO1A
IL32	4.26E-63	2.4133781	0.702	0.282	8.53E-60	T_cells	IL32
RGS1	6.89E-62	1.3857141	0.591	0.091	1.38E-58	T_cells	RGS1
IL2RG	2.31E-61	1.0670785	0.554	0.122	4.62E-58	T_cells	IL2RG
CST7	2.16E-54	1.1650933	0.525	0.058	4.32E-51	T_cells	CST7
NKG7	2.63E-50	1.9561448	0.562	0.149	5.26E-47	T_cells	NKG7
ALOX5AP	1.56E-48	1.028269	0.558	0.111	3.11E-45	T_cells	ALOX5AP
ARHGDIB	3.19E-44	1.2885209	0.789	0.452	6.38E-41	T_cells	ARHGDIB
IL7R	7.29E-31	1.2046833	0.517	0.158	1.46E-27	T_cells	IL7R
SDS	0	2.2137675	0.869	0.153	0	Monocyte_(	SDS
MMP9	0	1.2712564	0.81	0.045	0	Monocyte_(	MMP9
SRGN2	3.83E-292	4.5100366	0.997	0.175	7.65E-289	Monocyte_(	SRGN
LYZ	3.20E-279	4.652962	0.929	0.063	6.40E-276	Monocyte_(	LYZ
TYROBP	6.71E-279	4.0061224	0.935	0.048	1.34E-275	Monocyte_(	TYROBP
FCER1G	4.74E-269	3.159808	0.912	0.038	9.48E-266	Monocyte_(	FCER1G
AIF1	1.67E-267	2.8283883	0.912	0.078	3.34E-264	Monocyte_(	AIF1
C1QC	1.60E-266	2.3738594	0.81	0.016	3.21E-263	Monocyte_(	C1QC
C1QB	2.11E-263	3.110663	0.83	0.015	4.22E-260	Monocyte_(	C1QB
CCR7	9.45E-263	1.5429371	0.812	0.059	1.89E-259	Monocyte_(	CCR7
HLA-DQA11	5.58E-259	4.037803	0.938	0.129	1.12E-255	Monocyte_(	HLA-DQA1
HLA-DPB13	5.74E-258	5.0539742	0.972	0.242	1.15E-254	Monocyte_(	HLA-DPB1
CST71	9.89E-254	1.5948887	0.818	0.047	1.98E-250	Monocyte_(	CST7
HLA-DPA12	3.41E-251	4.3809692	0.952	0.259	6.82E-248	Monocyte_(	HLA-DPA1
FCER1A	1.47E-248	1.2902683	0.608	0.002	2.94E-245	Monocyte_(	FCER1A
HLA-DRA1	2.29E-246	4.9992694	0.994	0.281	4.57E-243	Monocyte_(	HLA-DRA
C1QA	5.95E-243	3.3489484	0.844	0.024	1.19E-239	Monocyte_(	C1QA
C15orf48	7.24E-241	3.5043465	0.929	0.156	1.45E-237	Monocyte_(	C15orf48
GPR183	2.19E-239	3.4339396	0.892	0.064	4.38E-236	Monocyte_(	GPR183
HLA-DRB53	1.48E-229	3.4692385	0.994	0.389	2.96E-226	Monocyte_(	HLA-DRB5
HLA-DQB11	4.85E-228	4.2346477	0.943	0.215	9.69E-225	Monocyte_(	HLA-DQB1
HLA-DRB11	1.37E-222	3.944887	0.98	0.323	2.73E-219	Monocyte_(	HLA-DRB1

CLEC10A	3.24E-218	1.4879792	0.699	0.012	6.49E-215	Monocyte_(	CLEC10A
VMO1	3.14E-215	1.7824465	0.824	0.151	6.27E-212	Monocyte_(	VMO1
DNASE1L3	1.21E-211	1.494473	0.784	0.153	2.41E-208	Monocyte_(	DNASE1L3
CD741	1.66E-196	4.1576292	1	0.6	3.32E-193	Monocyte_(	CD74
LST1	1.02E-189	2.5588406	0.838	0.045	2.03E-186	Monocyte_(	LST1
HLA-DMA1	2.82E-186	2.1988294	0.898	0.223	5.63E-183	Monocyte_(	HLA-DMA
MS4A6A	2.58E-171	1.9947493	0.787	0.04	5.16E-168	Monocyte_(	MS4A6A
OLR1	1.11E-162	1.4273274	0.759	0.147	2.21E-159	Monocyte_(	OLR1
EREG	1.03E-146	3.0818487	0.747	0.032	2.05E-143	Monocyte_(	EREG
HLA-DMB	2.88E-145	1.3251034	0.713	0.047	5.77E-142	Monocyte_(	HLA-DMB
CREM1	1.88E-140	2.3624535	0.963	0.447	3.76E-137	Monocyte_(	CREM
MMP19	3.51E-138	1.0649613	0.73	0.099	7.02E-135	Monocyte_(	MMP19
MS4A7	2.18E-128	1.6327493	0.676	0.026	4.37E-125	Monocyte_(	MS4A7
G0S2	1.83E-126	3.0511177	0.906	0.385	3.67E-123	Monocyte_(	G0S2
LAPTM5	2.62E-125	1.7649771	0.747	0.043	5.23E-122	Monocyte_(	LAPTM5
SERPINB9	4.83E-117	1.9597853	0.756	0.18	9.66E-114	Monocyte_(	SERPINB9
CD83	4.36E-115	1.7219033	0.747	0.206	8.73E-112	Monocyte_(	CD83
SPP1	2.21E-114	1.7012299	0.648	0.13	4.42E-111	Monocyte_(	SPP1
SAMSN1	6.04E-108	1.7423411	0.705	0.046	1.21E-104	Monocyte_(	SAMSN1
EMP32	6.91E-104	1.3634824	0.929	0.369	1.38E-100	Monocyte_(	EMP3
CSF2RA	6.88E-96	1.2141063	0.668	0.098	1.38E-92	Monocyte_(	CSF2RA
RGS2	7.51E-93	1.7965775	0.747	0.169	1.50E-89	Monocyte_(	RGS2
PLEK	2.54E-91	1.4631046	0.651	0.014	5.09E-88	Monocyte_(	PLEK
BASP12	5.39E-91	1.2221352	0.841	0.315	1.08E-87	Monocyte_(	BASP1
C5AR1	7.93E-91	1.0367089	0.642	0.08	1.59E-87	Monocyte_(	C5AR1
PLAUR	2.92E-83	1.4989995	0.912	0.532	5.83E-80	Monocyte_(	PLAUR
CXCL16	1.74E-82	1.2365426	0.824	0.37	3.48E-79	Monocyte_(	CXCL16
RP11-1143(	9.01E-82	1.3538665	0.631	0.065	1.80E-78	Monocyte_(	RP11-1143G9.4
CXCL3	3.82E-81	2.5403488	0.767	0.322	7.65E-78	Monocyte_(	CXCL3
HCST1	2.30E-80	1.3459015	0.676	0.072	4.60E-77	Monocyte_(	HCST
BCL2A1	3.05E-80	1.9273884	0.656	0.053	6.11E-77	Monocyte_(	BCL2A1
JCHAIN3	4.72E-78	-1.622427	0.716	0.266	9.45E-75	Monocyte_(	JCHAIN
CLEC7A	1.10E-73	1.1477624	0.594	0.055	2.21E-70	Monocyte_(	CLEC7A
CTSL1	1.31E-71	1.3059362	0.861	0.378	2.62E-68	Monocyte_(	CTSL
IL1B	7.29E-71	2.2578719	0.705	0.213	1.46E-67	Monocyte_(	IL1B
IGSF6	1.13E-69	1.1339799	0.597	0.079	2.27E-66	Monocyte_(	IGSF6
IGHG41	3.75E-68	-2.019854	0.707	0.241	7.50E-65	Monocyte_(	IGHG4
PCP42	5.56E-68	-1.338077	0.747	0.332	1.11E-64	Monocyte_(	PCP4
INSIG11	6.99E-68	1.3808992	0.821	0.376	1.40E-64	Monocyte_(	INSIG1
CTSS	1.22E-67	1.4882786	0.815	0.475	2.44E-64	Monocyte_(	CTSS
LGALS2	5.75E-67	1.3927004	0.565	0.01	1.15E-63	Monocyte_(	LGALS2
CXCL21	1.93E-65	1.2519588	0.864	0.451	3.85E-62	Monocyte_(	CXCL2
RGS11	5.69E-59	1.7696007	0.619	0.086	1.14E-55	Monocyte_(	RGS1
ITGB2	8.60E-57	1.0118585	0.602	0.075	1.72E-53	Monocyte_(	ITGB2
ID2	2.82E-56	1.5001083	0.767	0.364	5.63E-53	Monocyte_(	ID2
CPVL	3.22E-56	1.5478216	0.67	0.224	6.45E-53	Monocyte_(	CPVL
IGHA13	3.02E-54	-1.588084	0.67	0.246	6.03E-51	Monocyte_(	IGHA1
IGKC2	3.50E-51	-3.981527	0.781	0.381	6.99E-48	Monocyte_(	IGKC
FGL2	4.90E-47	1.0285583	0.693	0.296	9.80E-44	Monocyte_(	FGL2
FCN1	4.60E-45	1.1764397	0.514	0.022	9.19E-42	Monocyte_(	FCN1
SPI1	1.47E-38	1.1375962	0.543	0.022	2.94E-35	Monocyte_(	SPI1

CCL3	9.14E-36	2.486751	0.528	0.057	1.83E-32	Monocyte_(	CCL3
IGHG13	1.14E-34	-1.823251	0.636	0.292	2.27E-31	Monocyte_(	IGHG1
CXCR41	1.62E-32	1.12075	0.554	0.106	3.25E-29	Monocyte_(	CXCR4
MYH112	2.11E-27	-1.627176	0.73	0.37	4.21E-24	Monocyte_(	MYH11
FAM26F	3.19E-26	1.0913656	0.543	0.147	6.37E-23	Monocyte_(	FAM26F
JAML	6.83E-23	1.0176488	0.506	0.05	1.37E-19	Monocyte_(	JAML
RNASE6	4.99E-22	1.1854835	0.52	0.075	9.98E-19	Monocyte_(	RNASE6
RGS10	2.89E-19	1.0042753	0.599	0.265	5.78E-16	Monocyte_(	RGS10
ACTG22	3.63E-17	-2.651627	0.764	0.419	7.26E-14	Monocyte_(	ACTG2
DES2	1.32E-16	-2.413718	0.75	0.405	2.63E-13	Monocyte_(	DES
CNN11	5.28E-16	-1.182671	0.622	0.301	1.06E-12	Monocyte_(	CNN1
ACTA21	1.38E-15	-1.825293	0.73	0.367	2.76E-12	Monocyte_(	ACTA2
ALOX5AP1	4.75E-15	1.3033542	0.5	0.109	9.49E-12	Monocyte_(	ALOX5AP
APOC1	1.12E-14	1.4983441	0.554	0.238	2.24E-11	Monocyte_(	APOC1
ACTG23	0	6.6097999	1	0.4	0	Smooth_mu:	ACTG2
DES3	0	6.3908863	1	0.386	0	Smooth_mu:	DES
ACTA22	0	4.9034472	1	0.346	0	Smooth_mu:	ACTA2
PCP43	0	5.200022	0.981	0.313	0	Smooth_mu:	PCP4
ACTC11	0	4.042585	0.864	0.266	0	Smooth_mu:	ACTC1
MYL91	0	5.490399	1	0.448	0	Smooth_mu:	MYL9
TPM11	0	5.2370574	1	0.442	0	Smooth_mu:	TPM1
TPM21	0	5.7737995	1	0.46	0	Smooth_mu:	TPM2
TAGLN1	0	5.9120234	1	0.405	0	Smooth_mu:	TAGLN
MYLK2	0	5.1173276	1	0.322	0	Smooth_mu:	MYLK
MYH113	0	5.196913	1	0.35	0	Smooth_mu:	MYH11
CNN12	0	4.6390379	0.997	0.276	0	Smooth_mu:	CNN1
PPP1R14A	0	4.2086205	0.984	0.219	0	Smooth_mu:	PPP1R14A
SYNM1	0	3.4370616	0.932	0.272	0	Smooth_mu:	SYNM
SMTN	0	3.2627129	0.898	0.227	0	Smooth_mu:	SMTN
SYNPO21	0	3.6701761	0.958	0.27	0	Smooth_mu:	SYNPO2
CSRP11	0	3.4967428	0.969	0.328	0	Smooth_mu:	CSRP1
RAMP11	0	2.584655	0.856	0.313	0	Smooth_mu:	RAMP1
PALLD1	0	2.9363263	0.942	0.281	0	Smooth_mu:	PALLD
FN12	0	2.5473464	0.919	0.301	0	Smooth_mu:	FN1
SORBS1	0	2.4310925	0.788	0.201	0	Smooth_mu:	SORBS1
FHL11	0	2.3002403	0.819	0.193	0	Smooth_mu:	FHL1
FLNA1	0	3.7408624	0.974	0.293	0	Smooth_mu:	FLNA
CKB	0	2.601891	0.945	0.476	0	Smooth_mu:	CKB
CALD12	0	3.4511065	1	0.419	0	Smooth_mu:	CALD1
LPP	0	3.1801937	0.974	0.414	0	Smooth_mu:	LPP
SVIL	0	2.4056324	0.864	0.297	0	Smooth_mu:	SVIL
SELM2	0	2.3552784	0.977	0.42	0	Smooth_mu:	SELM
TNS1	2.00E-294	2.2393472	0.782	0.217	3.99E-291	Smooth_mu:	TNS1
CAV12	2.67E-294	2.1267775	0.895	0.311	5.33E-291	Smooth_mu:	CAV1
PDLIM71	1.41E-293	2.2549533	0.822	0.266	2.83E-290	Smooth_mu:	PDLIM7
HSPB62	3.80E-288	2.2747748	0.868	0.304	7.60E-285	Smooth_mu:	HSPB6
PPP1R12B	8.43E-286	2.1721348	0.763	0.185	1.69E-282	Smooth_mu:	PPP1R12B
PKIG1	1.40E-272	2.2877365	0.808	0.299	2.79E-269	Smooth_mu:	PKIG
PDLIM3	1.40E-248	2.0282849	0.729	0.198	2.80E-245	Smooth_mu:	PDLIM3
SORBS2	4.05E-239	2.3009825	0.724	0.249	8.10E-236	Smooth_mu:	SORBS2
CRYAB1	3.33E-231	2.071205	0.724	0.219	6.65E-228	Smooth_mu:	CRYAB

RGS51	1.30E-227	2.7845489	0.729	0.291	2.59E-224	Smooth_mu:RGS5
MAP1B1	4.49E-223	1.9572404	0.737	0.268	8.98E-220	Smooth_mu:MAP1B
LMOD1	3.23E-222	1.9839639	0.664	0.197	6.47E-219	Smooth_mu:LMOD1
CLIC41	2.28E-219	1.9954825	0.714	0.248	4.56E-216	Smooth_mu:CLIC4
FILIP1	7.50E-215	2.0031188	0.635	0.17	1.50E-211	Smooth_mu:FILIP1
FLNC	3.49E-208	1.9977894	0.63	0.171	6.99E-205	Smooth_mu:FLNC
PTRF3	8.22E-207	1.8196126	0.782	0.316	1.64E-203	Smooth_mu:PTRF
PLN	3.26E-201	2.147012	0.654	0.248	6.52E-198	Smooth_mu:PLN
COL4A21	1.95E-199	1.9357733	0.721	0.285	3.91E-196	Smooth_mu:COL4A2
HOPX	4.32E-199	2.1392364	0.612	0.168	8.63E-196	Smooth_mu:HOPX
COX7A12	4.80E-198	1.7736964	0.74	0.271	9.59E-195	Smooth_mu:COX7A1
NUPR12	9.07E-187	1.5626341	0.866	0.395	1.81E-183	Smooth_mu:NUPR1
COL4A11	1.85E-186	1.992503	0.669	0.246	3.70E-183	Smooth_mu:COL4A1
IGFBP74	7.20E-184	1.4543433	0.971	0.528	1.44E-180	Smooth_mu:IGFBP7
ADIRF1	1.02E-168	-1.988945	0.515	0.962	2.05E-165	Smooth_mu:ADIRF
ITGA5	4.12E-167	1.6339506	0.559	0.157	8.24E-164	Smooth_mu:ITGA5
AQP31	1.92E-164	-2.064272	0.37	0.922	3.84E-161	Smooth_mu:AQP3
KRT192	6.29E-164	-2.299237	0.383	0.915	1.26E-160	Smooth_mu:KRT19
PTGS1	3.52E-162	1.673683	0.504	0.123	7.05E-159	Smooth_mu:PTGS1
S100P	1.09E-161	-2.423716	0.498	0.947	2.17E-158	Smooth_mu:S100P
KRT182	1.21E-160	-1.668414	0.22	0.834	2.42E-157	Smooth_mu:KRT18
LGALS13	2.36E-160	1.1309054	1	0.471	4.73E-157	Smooth_mu:LGALS1
FABP51	4.76E-160	-2.092174	0.352	0.923	9.52E-157	Smooth_mu:FABP5
SFN2	9.82E-158	-1.971438	0.241	0.845	1.96E-154	Smooth_mu:SFN
SLMAP	3.71E-156	1.7279406	0.698	0.363	7.42E-153	Smooth_mu:SLMAP
FXYD33	2.35E-155	-1.93786	0.296	0.865	4.70E-152	Smooth_mu:FXYD3
AKR1C22	2.73E-154	-1.580099	0.218	0.825	5.46E-151	Smooth_mu:AKR1C2
C19orf332	4.21E-154	-1.526192	0.199	0.808	8.42E-151	Smooth_mu:C19orf33
GPX22	5.26E-154	-1.601698	0.228	0.834	1.05E-150	Smooth_mu:GPX2
SPARCL13	2.27E-152	1.0486111	0.872	0.353	4.54E-149	Smooth_mu:SPARCL1
SNCG2	1.58E-151	-1.940264	0.276	0.859	3.16E-148	Smooth_mu:SNCG
TACSTD22	1.59E-149	-1.935798	0.288	0.863	3.17E-146	Smooth_mu:TACSTD2
KRT17	6.91E-147	-2.309224	0.412	0.924	1.38E-143	Smooth_mu:KRT17
KRT132	2.59E-146	-2.051697	0.328	0.886	5.17E-143	Smooth_mu:KRT13
KCNMB1	1.81E-145	1.630595	0.557	0.206	3.62E-142	Smooth_mu:KCNMB1
PHLDA22	1.82E-145	-1.946379	0.334	0.881	3.64E-142	Smooth_mu:PHLDA2
AGR24	4.25E-144	-1.913628	0.25	0.84	8.50E-141	Smooth_mu:AGR2
NEXN	4.69E-144	1.5745803	0.548	0.194	9.37E-141	Smooth_mu:NEXN
HSPG21	2.34E-136	1.5489818	0.572	0.214	4.67E-133	Smooth_mu:HSPG2
GAS6	2.94E-136	1.4010159	0.556	0.194	5.87E-133	Smooth_mu:GAS6
A2M3	1.28E-135	1.4601261	0.745	0.405	2.55E-132	Smooth_mu:A2M
CCL24	9.16E-135	2.3216197	0.666	0.298	1.83E-131	Smooth_mu:CCL2
LY6D2	5.84E-134	-2.103964	0.312	0.857	1.17E-130	Smooth_mu:LY6D
SERPINB52	6.49E-133	-1.183983	0.166	0.748	1.30E-129	Smooth_mu:SERPINB5
CXCL8	1.18E-132	-2.201451	0.373	0.889	2.37E-129	Smooth_mu:CXCL8
IGFBP21	5.34E-131	-1.537868	0.249	0.815	1.07E-127	Smooth_mu:IGFBP2
AREG	7.54E-130	-1.92462	0.242	0.816	1.51E-126	Smooth_mu:AREG
SMIM222	1.36E-127	-1.363714	0.179	0.755	2.72E-124	Smooth_mu:SMIM22
RARRES22	2.06E-127	1.113014	0.716	0.298	4.12E-124	Smooth_mu:RARRES2
FABP4	3.08E-126	-2.103362	0.439	0.923	6.16E-123	Smooth_mu:FABP4
CYSTM11	1.99E-123	-1.494568	0.402	0.877	3.97E-120	Smooth_mu:CYSTM1

CAPG	6.95E-120	-1.125882	0.129	0.683	1.39E-116	Smooth_mu:CAPG
HPGD1	7.28E-120	-1.480592	0.204	0.759	1.46E-116	Smooth_mu:HPGD
DHRS21	1.41E-119	-1.785662	0.231	0.776	2.83E-116	Smooth_mu:DHRS2
MPZL2	6.47E-119	-1.120132	0.16	0.723	1.29E-115	Smooth_mu:MPZL2
MGST12	8.65E-119	-1.224667	0.388	0.863	1.73E-115	Smooth_mu:MGST1
LAMB31	9.64E-116	-1.137818	0.171	0.718	1.93E-112	Smooth_mu:LAMB3
S100A2	1.25E-114	-2.391003	0.491	0.928	2.51E-111	Smooth_mu:S100A2
KLF52	6.97E-114	-1.182901	0.234	0.784	1.39E-110	Smooth_mu:KLF5
S100A41	1.38E-113	-2.031026	0.305	0.764	2.75E-110	Smooth_mu:S100A4
CLDN7	5.50E-112	-1.338183	0.141	0.698	1.10E-108	Smooth_mu:CLDN7
CFD2	4.40E-111	-3.319641	0.204	0.7	8.80E-108	Smooth_mu:CFD
FAM3B1	2.88E-106	-1.066621	0.16	0.688	5.75E-103	Smooth_mu:FAM3B
ANXA101	1.30E-105	-1.07635	0.147	0.676	2.61E-102	Smooth_mu:ANXA10
TUBA1A2	7.77E-104	1.222717	0.727	0.379	1.55E-100	Smooth_mu:TUBA1A
EZR1	1.04E-102	-1.201656	0.378	0.844	2.07E-99	Smooth_mu:EZR
PRSS232	2.93E-101	1.0915889	0.675	0.344	5.86E-98	Smooth_mu:PRSS23
CDC42EP5	3.53E-100	-1.083002	0.15	0.665	7.06E-97	Smooth_mu:CDC42EP5
CLDN41	4.04E-100	-2.032216	0.286	0.777	8.09E-97	Smooth_mu:CLDN4
KRT73	9.22E-100	-1.775827	0.213	0.738	1.84E-96	Smooth_mu:KRT7
PMAIP1	1.86E-98	-1.118676	0.199	0.716	3.72E-95	Smooth_mu:PMAIP1
HLA-B	7.08E-98	-1.363871	0.596	0.926	1.42E-94	Smooth_mu:HLA-B
PERP1	1.83E-95	-1.109463	0.341	0.819	3.66E-92	Smooth_mu:PERP
KRT82	3.53E-95	-1.263291	0.394	0.842	7.07E-92	Smooth_mu:KRT8
DUSP5	4.28E-95	-1.146458	0.317	0.812	8.56E-92	Smooth_mu:DUSP5
CRABP2	1.64E-94	-1.321935	0.132	0.613	3.28E-91	Smooth_mu:CRABP2
EMP11	2.26E-91	-1.22536	0.2	0.691	4.53E-88	Smooth_mu:EMP1
DUOXA2	8.59E-88	-1.261611	0.136	0.621	1.72E-84	Smooth_mu:DUOXA2
TSPAN1	1.02E-87	-1.120411	0.16	0.661	2.04E-84	Smooth_mu:TSPAN1
ELF3	3.88E-87	-1.412503	0.184	0.686	7.76E-84	Smooth_mu:ELF3
C9orf161	4.98E-87	-1.039489	0.593	0.905	9.95E-84	Smooth_mu:C9orf16
HN11	8.72E-87	-1.254374	0.199	0.703	1.74E-83	Smooth_mu:HN1
LYPD31	1.02E-84	-1.01322	0.105	0.584	2.04E-81	Smooth_mu:LYPD3
HMGA1	3.42E-84	-1.08943	0.342	0.803	6.85E-81	Smooth_mu:HMGA1
PHLDA11	3.81E-83	-1.280291	0.342	0.801	7.63E-80	Smooth_mu:PHLDA1
VSIG2	9.32E-83	-1.042509	0.123	0.613	1.86E-79	Smooth_mu:VSIG2
CD742	1.82E-73	-2.004872	0.179	0.632	3.64E-70	Smooth_mu:CD74
MMP1	1.31E-69	-1.479761	0.121	0.543	2.61E-66	Smooth_mu:MMP1
SLPI	1.38E-68	-1.546295	0.116	0.565	2.76E-65	Smooth_mu:SLPI
TXNDC172	1.97E-67	-1.126484	0.33	0.755	3.94E-64	Smooth_mu:TXNDC17
HES4	2.00E-67	-1.064664	0.373	0.777	4.01E-64	Smooth_mu:HES4
FBLN12	3.24E-67	-2.71215	0.116	0.513	6.48E-64	Smooth_mu:FBLN1
PLIN2	3.87E-64	-1.006115	0.183	0.617	7.75E-61	Smooth_mu:PLIN2
SPINK11	1.40E-63	-2.236172	0.242	0.673	2.80E-60	Smooth_mu:SPINK1
GDF15	8.12E-63	-1.426796	0.165	0.576	1.62E-59	Smooth_mu:GDF15
VAMP82	1.48E-58	-1.211587	0.162	0.626	2.96E-55	Smooth_mu:VAMP8
HES1	9.43E-54	-1.541323	0.202	0.594	1.89E-50	Smooth_mu:HES1
PSCA1	6.47E-51	-1.925238	0.192	0.607	1.29E-47	Smooth_mu:PSCA
CCL20	1.31E-50	-1.593518	0.166	0.561	2.62E-47	Smooth_mu:CCL20
TSTD12	1.01E-41	-1.033013	0.142	0.568	2.03E-38	Smooth_mu:TSTD1
DKK1	4.88E-40	-1.440604	0.157	0.547	9.76E-37	Smooth_mu:DKK1
TM4SF11	3.52E-37	-1.004361	0.205	0.557	7.04E-34	Smooth_mu:TM4SF1
