# Supplementary material for: Comprehensive analysis of scRNA-Seq and bulk RNA-Seq reveals dynamic changes in the tumor immune microenvironment of bladder cancer and establishes a prognostic model
Source: J Transl Med. 2023 Mar 27;21:223. doi: 10.1186/s12967-023-04056-z (PMC10044739; doi:10.1186/s12967-023-04056-z)
Supplement: Supplementary file 11 — Additional file 11: Table S3 The key module genes. [file 12967_2023_4056_MOESM11_ESM.pdf]

probes	moduleColor	GS.IDD	p.GS.IDD	MEbrown	MEbrown
MFSD3	brown	0.364637168	5.73E-15	-0.525506455	6.71E-32
ESM1	brown	0.30799055	6.69E-11	-0.270231243	1.24E-08
TOMM40	brown	0.351119675	6.39E-14	-0.588242927	2.21E-41
LHX5	brown	0.157096968	0.00108132	-0.194022966	5.12E-05
MYOM2	brown	-0.537213589	1.61E-33	0.586076023	5.09E-41
SPCS2	brown	0.215827354	6.31E-06	-0.274979919	6.73E-09
SETDB2	brown	-0.10312656	0.032520298	0.302713664	1.46E-10
COX17	brown	0.277426023	4.88E-09	-0.334641389	1.04E-12
FANK1	brown	0.187505344	9.17E-05	-0.230928515	1.30E-06
PTPRB	brown	-0.11035711	0.022094044	0.405483779	1.90E-18
TRABD2B	brown	-0.234198697	9.07E-07	0.377277438	5.41E-16
SLC2A1	brown	0.122305456	0.011138178	-0.370636689	1.89E-15
GLYCTK	brown	0.206737867	1.55E-05	-0.221297209	3.60E-06
KCNE4	brown	-0.333452069	1.26E-12	0.763926791	1.91E-83
HOXC13	brown	0.190971067	6.74E-05	-0.2761926	5.74E-09
NME2	brown	0.125078317	0.009422479	-0.231816611	1.18E-06
PHLDA2	brown	0.031541308	0.514199313	-0.303491407	1.30E-10
EPHA3	brown	-0.427695488	1.50E-20	0.766499213	2.49E-84
C1orf35	brown	0.365892545	4.55E-15	-0.584230368	1.03E-40
PDE5A	brown	-0.411569267	5.22E-19	0.754489887	2.70E-80
PLIN4	brown	-0.306277418	8.63E-11	0.389952842	4.56E-17
HEY2	brown	-0.127411798	0.008165105	0.355398352	3.01E-14
ACOX2	brown	-0.456211717	1.72E-23	0.743458124	8.63E-77
OR51E2	brown	-0.028615689	0.553996926	0.342946464	2.60E-13
ASB2	brown	-0.482268742	1.98E-26	0.740926369	5.19E-76
EXOSC7	brown	0.218905519	4.61E-06	-0.430010958	8.83E-21
FAM86C1	brown	0.221943002	3.37E-06	-0.278294635	4.35E-09
SH3D21	brown	0.210265274	1.10E-05	-0.309809003	5.10E-11
PPP4C	brown	0.356716772	2.39E-14	-0.566748487	6.65E-38
JAKMIP3	brown	-0.160128854	0.000861113	0.297459186	3.11E-10
ALDOA	brown	0.128956147	0.007417479	-0.395433914	1.51E-17
RCAN3	brown	0.240346743	4.57E-07	-0.291651045	7.06E-10
LCN6	brown	-0.43309584	4.36E-21	0.580621563	4.05E-40
MED31	brown	0.173005814	0.000312999	-0.348584506	9.91E-14
LEMD2	brown	0.404226618	2.47E-18	-0.460821904	5.41E-24
FAM69B	brown	-0.046252493	0.338651848	0.17073553	0.000376133
MPLKIP	brown	0.359942899	1.34E-14	-0.434863872	2.89E-21
MRPL22	brown	0.225939691	2.21E-06	-0.3790607	3.84E-16
GALNT16	brown	-0.20796216	1.38E-05	0.478368709	5.65E-26
NLGN3	brown	-0.21893783	4.59E-06	0.373005658	1.21E-15
ITGB6	brown	0.178887489	0.000192384	-0.274959462	6.74E-09
SLC18A2	brown	-0.186165192	0.000103106	0.495342335	5.29E-28
DENND5A	brown	-0.263185578	3.03E-08	0.680729028	7.92E-60
ADCY2	brown	-0.321683529	8.28E-12	0.476643415	8.96E-26
EIF3I	brown	0.234848675	8.44E-07	-0.451202899	5.91E-23
PDE1C	brown	-0.52110252	2.63E-31	0.811692071	5.35E-102
PRG4	brown	-0.097840942	0.042577114	0.417782468	1.36E-19
PCDHGA2	brown	-0.149516901	0.001877747	0.390280959	4.27E-17
TULP2	brown	-0.191353034	6.51E-05	0.301763937	1.67E-10
POU6F1	brown	-0.303579036	1.28E-10	0.670459439	1.90E-57

CSPG4	brown	-0.223593493	2.83E-06	0.546057544	8.69E-35
PLP1	brown	-0.693532981	6.19E-63	0.729553505	1.27E-72
ZFYVE19	brown	0.3109771	4.28E-11	-0.393955016	2.04E-17
NR2F1	brown	-0.319096344	1.24E-11	0.545713498	9.75E-35
PTGS2	brown	-0.275803796	6.04E-09	0.290465894	8.33E-10
OSBPL5	brown	-0.236258431	7.22E-07	0.490283571	2.19E-27
C8orf76	brown	0.327470863	3.31E-12	-0.478229385	5.87E-26
DDR1	brown	0.213241531	8.18E-06	-0.48288265	1.67E-26
CDH6	brown	-0.106956095	0.026568608	0.408431069	1.02E-18
PLIN1	brown	-0.181483576	0.000154429	0.402117454	3.84E-18
PDIA3	brown	0.371761448	1.53E-15	-0.398471831	8.15E-18
KLHL10	brown	-0.381033762	2.63E-16	0.615981109	2.83E-46
QRFP	brown	-0.323699392	6.03E-12	0.371956865	1.48E-15
MAPK4	brown	-0.276200357	5.73E-09	0.405552801	1.87E-18
VSTM2A	brown	-0.464237393	2.27E-24	0.233872416	9.40E-07
FGF10	brown	-0.564362106	1.56E-37	0.786255924	1.60E-91
RASD2	brown	-0.13831145	0.004058719	0.379047877	3.85E-16
TYK2	brown	0.304640439	1.10E-10	-0.280051734	3.44E-09
TMOD1	brown	-0.49843159	2.20E-28	0.786887372	9.13E-92
FXYD6	brown	-0.416817303	1.68E-19	0.824905469	4.31E-108
ANTXR2	brown	-0.357195252	2.19E-14	0.706366986	3.23E-66
PBX1	brown	-0.257149813	6.37E-08	0.362426973	8.57E-15
COL19A1	brown	-0.460535468	5.82E-24	0.618275701	1.06E-46
SORBS2	brown	-0.483138964	1.56E-26	0.706117861	3.76E-66
ENTPD6	brown	0.342602103	2.75E-13	-0.414791599	2.61E-19
CDO1	brown	-0.314556598	2.49E-11	0.559857792	7.67E-37
PLCD4	brown	-0.532793653	6.69E-33	0.778739262	1.07E-88
FAT3	brown	-0.215220225	6.71E-06	0.35912314	1.55E-14
CDON	brown	-0.344682536	1.93E-13	0.597307332	6.27E-43
PRX	brown	-0.285913675	1.56E-09	0.501713474	8.57E-29
HSPA1L	brown	-0.153573621	0.001401852	0.259684105	4.67E-08
KCNE3	brown	0.052527068	0.277123318	0.139214086	0.003821904
SCN2B	brown	-0.408422979	1.02E-18	0.559156874	9.81E-37
RNF112	brown	-0.466810057	1.17E-24	0.668349039	5.69E-57
AC011530.4	brown	-0.478932482	4.86E-26	0.625682646	4.20E-48
FAM46B	brown	-0.355325051	3.05E-14	0.46942216	5.98E-25
ANO4	brown	-0.216869667	5.68E-06	0.273693168	7.95E-09
CFL2	brown	-0.449523852	8.90E-23	0.828108617	1.20E-109
NRN1	brown	-0.205200399	1.80E-05	0.345397875	1.71E-13
NFASC	brown	-0.464683185	2.03E-24	0.648181771	1.33E-52
HGH1	brown	0.454107654	2.89E-23	-0.515065033	1.66E-30
GTPBP2	brown	0.311752302	3.81E-11	-0.366185577	4.31E-15
CRTAP	brown	-0.31106048	4.23E-11	0.579683916	5.76E-40
DDR2	brown	-0.390549818	4.05E-17	0.844010252	7.16E-118
ADAM8	brown	0.243948236	3.03E-07	-0.250257248	1.45E-07
FBXL22	brown	-0.458055768	1.08E-23	0.797945025	3.77E-96
KLF4	brown	-0.38619554	9.60E-17	0.261508768	3.73E-08
KIAA1614	brown	-0.330864216	1.92E-12	0.67800695	3.46E-59
KCNA5	brown	-0.490135115	2.28E-27	0.69738137	6.70E-64
MOCOS	brown	0.237217708	6.49E-07	-0.366588275	4.01E-15
CHMP1A	brown	0.210363017	1.09E-05	-0.383702348	1.57E-16

EPHA7	brown	-0.429769211	9.34E-21	0.605664979	2.12E-44
DCAF13	brown	0.259967218	4.52E-08	-0.343215994	2.48E-13
CAMKK1	brown	-0.224310729	2.63E-06	0.240735887	4.37E-07
C1QTNF4	brown	-0.161776285	0.000759618	0.269916993	1.30E-08
PROC	brown	0.128786275	0.007496607	-0.179052915	0.000189726
RRP9	brown	0.257183285	6.35E-08	-0.434321193	3.28E-21
RRAGD	brown	-0.168489785	0.00045009	0.367538619	3.36E-15
FABP5	brown	0.054206973	0.262027224	-0.363480318	7.07E-15
C8orf88	brown	-0.312261519	3.53E-11	0.616758375	2.03E-46
PCGF5	brown	-0.19640915	4.11E-05	0.399185593	7.04E-18
F11R	brown	0.270795157	1.16E-08	-0.466903428	1.15E-24
MYOZ2	brown	-0.320311771	1.03E-11	0.597972175	4.81E-43
EYA4	brown	-0.26611542	2.10E-08	0.367263106	3.54E-15
TYSND1	brown	0.268156418	1.62E-08	-0.497772231	2.66E-28
LPAR2	brown	0.336655072	7.44E-13	-0.562649906	2.87E-37
C1QTNF9	brown	-0.49187422	1.40E-27	0.643480241	1.25E-51
DDT	brown	0.150160327	0.001793532	-0.328164664	2.97E-12
ITIH5	brown	-0.546572175	7.31E-35	0.787852908	3.87E-92
LPHN2	brown	-0.111348441	0.020919448	0.277641906	4.74E-09
F2RL3	brown	-0.003108858	0.948747958	0.278885687	4.02E-09
MYOC	brown	-0.641255272	3.54E-51	0.681589675	4.95E-60
GPCPD1	brown	-0.186433293	0.000100717	0.332403854	1.49E-12
LRCH2	brown	-0.375087278	8.20E-16	0.687112441	2.34E-61
PDZD4	brown	-0.408837252	9.36E-19	0.603339924	5.50E-44
CD200	brown	-0.276564431	5.46E-09	0.649305028	7.75E-53
CADPS2	brown	-0.046880705	0.33212498	0.242387373	3.62E-07
CTDSPL	brown	-0.141367259	0.003306703	0.170536343	0.000382203
DNAJB5	brown	-0.407317802	1.29E-18	0.750586418	4.92E-79
USHBP1	brown	-0.217362383	5.40E-06	0.497266725	3.07E-28
DYNC1I1	brown	-0.244222965	2.94E-07	0.476138295	1.02E-25
RASSF7	brown	0.33590123	8.43E-13	-0.594982282	1.58E-42
CC2D1B	brown	0.312575117	3.36E-11	-0.37368356	1.07E-15
DDX56	brown	0.390265589	4.29E-17	-0.569192195	2.75E-38
MPC1	brown	-0.200113404	2.92E-05	0.196020818	4.26E-05
IQSEC1	brown	-0.106658447	0.026994965	0.283511979	2.16E-09
F10	brown	-0.654439638	6.37E-54	0.738191918	3.52E-75
C16orf74	brown	0.135986736	0.004730929	-0.459738509	7.11E-24
NTF4	brown	0.067344947	0.163313825	-0.290280362	8.55E-10
ITPR1	brown	-0.413412551	3.51E-19	0.716968517	4.58E-69
ABCB4	brown	-0.273946009	7.70E-09	0.636765472	2.84E-50
FAM83H	brown	0.358034141	1.89E-14	-0.685435317	5.96E-61
ATP2B4	brown	-0.282081322	2.62E-09	0.519172279	4.76E-31
MOCS2	brown	-0.106024655	0.027922468	0.214764029	7.02E-06
CDC37	brown	0.218336572	4.89E-06	-0.393769109	2.12E-17
FAM162B	brown	-0.28609454	1.52E-09	0.305184968	1.01E-10
DCLK1	brown	-0.342463972	2.82E-13	0.648738066	1.02E-52
EFNB1	brown	0.012047439	0.803281215	-0.286076994	1.53E-09
RNF135	brown	0.241850698	3.85E-07	-0.240412483	4.54E-07
INPP5B	brown	-0.155166198	0.001247464	0.424147027	3.32E-20
COASY	brown	0.42135724	6.18E-20	-0.625021327	5.62E-48
RPS6KA2	brown	-0.356314785	2.56E-14	0.577970522	1.09E-39

DNALI1	brown	-0.14402799	0.002757461	0.233397025	9.91E-07
ASB1	brown	-0.237626101	6.20E-07	0.421198116	6.40E-20
POLR2H	brown	0.464740857	2.00E-24	-0.662498109	1.14E-55
RER1	brown	0.320136639	1.05E-11	-0.454885621	2.39E-23
MPZ	brown	-0.414339491	2.87E-19	0.464638591	2.05E-24
TP63	brown	0.118031238	0.014325767	-0.366950161	3.75E-15
TMEM41A	brown	0.314380141	2.56E-11	-0.40764239	1.21E-18
RBM24	brown	-0.520058747	3.63E-31	0.724381559	3.90E-71
GNAO1	brown	-0.430963569	7.11E-21	0.744755144	3.41E-77
USP2	brown	-0.345474714	1.69E-13	0.33550736	8.99E-13
TRIM7	brown	0.106135713	0.027757992	-0.286042596	1.53E-09
CACNA2D1	brown	-0.300309148	2.06E-10	0.680123617	1.10E-59
TEF	brown	-0.286314942	1.48E-09	0.276921479	5.21E-09
DESI1	brown	0.136771916	0.004493417	-0.220721784	3.82E-06
ITGA10	brown	-0.118292625	0.014109983	0.416013187	2.00E-19
OXLD1	brown	0.394707283	1.76E-17	-0.555273967	3.78E-36
TNS4	brown	0.079123673	0.101309691	-0.3966042	1.19E-17
PPIF	brown	0.21287652	8.48E-06	-0.365077478	5.29E-15
ARHGEF25	brown	-0.34199173	3.05E-13	0.650884806	3.61E-53
NEURL1	brown	-0.228606465	1.67E-06	0.467761737	9.19E-25
RPUSD3	brown	0.294388227	4.81E-10	-0.358294739	1.80E-14
EHBP1L1	brown	-0.204407895	1.94E-05	0.259191869	4.97E-08
PCDH12	brown	-0.031150545	0.5194279	0.446843464	1.70E-22
PLSCR4	brown	-0.312815815	3.24E-11	0.683313952	1.92E-60
RGL1	brown	-0.234352108	8.92E-07	0.607562749	9.71E-45
RARB	brown	-0.199238805	3.16E-05	0.468460851	7.67E-25
IGSF10	brown	-0.493861757	8.04E-28	0.526469358	4.96E-32
PEMT	brown	0.212846564	8.51E-06	-0.376148343	6.71E-16
SNRPE	brown	0.326380245	3.94E-12	-0.48528797	8.69E-27
TBX18	brown	-0.126298091	0.008745237	0.303554727	1.29E-10
ELN	brown	-0.298253924	2.77E-10	0.675679925	1.20E-58
LEFTY2	brown	-0.057263295	0.236036036	0.49658003	3.73E-28
IGIP	brown	-0.288863006	1.04E-09	0.526926378	4.30E-32
OTUB1	brown	0.30332345	1.33E-10	-0.5456362	1.00E-34
DMRTA2	brown	0.137857164	0.004182856	-0.200273512	2.87E-05
EID1	brown	-0.266913021	1.90E-08	0.505772404	2.63E-29
FBLN5	brown	-0.386212863	9.57E-17	0.737372779	6.21E-75
IQCJ-SCHIP	brown	-0.257385983	6.19E-08	0.541486391	3.97E-34
SGCG	brown	-0.560114118	7.01E-37	0.602228695	8.65E-44
RBP7	brown	-0.240491753	4.50E-07	0.437014225	1.76E-21
DBNDD2	brown	-0.1849836	0.00011428	0.285009471	1.76E-09
SEMA3A	brown	-0.247223441	2.08E-07	0.556167087	2.78E-36
PRSS22	brown	0.173658571	0.000296767	-0.338191229	5.77E-13
PCGF1	brown	0.417557348	1.43E-19	-0.563547736	2.09E-37
LAMTOR5	brown	0.261676669	3.66E-08	-0.527512153	3.58E-32
SEPP1	brown	-0.247564477	1.99E-07	0.463084117	3.05E-24
SLC8A2	brown	-0.230412924	1.37E-06	0.364749333	5.61E-15
HAPLN2	brown	-0.131452452	0.006337733	0.263069251	3.08E-08
KCTD10	brown	-0.292294227	6.45E-10	0.639058222	9.86E-51
IL11RA	brown	-0.275370799	6.39E-09	0.478685456	5.19E-26
TACC1	brown	-0.258126275	5.66E-08	0.497919635	2.55E-28

GUCA1C	brown	-0.373235831	1.16E-15	0.162142697	0.000738603
SDC3	brown	-0.212009	9.25E-06	0.548379797	3.98E-35
ATP1A2	brown	-0.60900609	5.34E-45	0.838928967	3.81E-115
NUDT7	brown	-0.22776567	1.82E-06	0.320560058	9.87E-12
RABEP1	brown	-0.16434911	0.000623024	0.429052207	1.10E-20
WFDC5	brown	0.032390926	0.50292589	-0.201484009	2.56E-05
LPAR1	brown	-0.316770754	1.77E-11	0.413670671	3.32E-19
PPIA	brown	0.351651475	5.82E-14	-0.555503517	3.49E-36
ALKBH4	brown	0.372775069	1.27E-15	-0.44269024	4.61E-22
BCL6B	brown	-0.165842246	0.000554579	0.537101549	1.67E-33
RBM38	brown	-0.108404649	0.024575444	0.200795562	2.74E-05
NDN	brown	-0.239763425	4.88E-07	0.441939076	5.51E-22
CARD14	brown	0.135936979	0.004746355	-0.304133503	1.18E-10
NFATC4	brown	-0.300299709	2.07E-10	0.513447064	2.70E-30
TNS2	brown	-0.435283233	2.63E-21	0.794931029	6.28E-95
PPIE	brown	0.182519931	0.000141339	-0.273273399	8.40E-09
SRF	brown	-0.46876555	7.09E-25	0.563480606	2.14E-37
NOP14	brown	0.083446912	0.083921804	-0.26345249	2.93E-08
FAM83A	brown	0.133305533	0.005629728	-0.306884242	7.89E-11
RNF126	brown	0.258246227	5.57E-08	-0.560239603	6.71E-37
ZC3H10	brown	-0.020287862	0.674838591	0.211434669	9.79E-06
ALDH1B1	brown	-0.303291197	1.34E-10	0.650309546	4.77E-53
XPNPEP2	brown	-0.521172185	2.58E-31	0.756901544	4.36E-81
ADCYAP1R1	brown	-0.432937559	4.52E-21	0.69889857	2.76E-64
HSPB2-C11o	brown	-0.35532829	3.05E-14	0.503345688	5.34E-29
ECSCR	brown	-0.271580071	1.05E-08	0.549473079	2.75E-35
NSFL1C	brown	0.200390724	2.84E-05	-0.374601005	8.99E-16
STC1	brown	-0.200251761	2.88E-05	0.416926421	1.64E-19
PPM1L	brown	-0.332138288	1.56E-12	0.444506904	2.99E-22
MTHFR	brown	-0.207550722	1.43E-05	0.298590257	2.64E-10
LDLRAD2	brown	-0.45850467	9.69E-24	0.802585878	4.50E-98
SMPX	brown	-0.294477267	4.75E-10	0.522897948	1.51E-31
3-Mar	brown	-0.056400159	0.243184787	0.211370888	9.85E-06
TMEM99	brown	0.227995729	1.78E-06	-0.390874558	3.80E-17
PAK4	brown	0.280574862	3.21E-09	-0.445467898	2.37E-22
MTURN	brown	-0.379035047	3.86E-16	0.479810162	3.84E-26
XPA	brown	-0.229011586	1.59E-06	0.259542967	4.76E-08
MYO1C	brown	-0.13578095	0.004795023	0.266705891	1.95E-08
ATAD3B	brown	0.296259849	3.69E-10	-0.488274053	3.82E-27
GAB2	brown	-0.18169255	0.000151702	0.439151032	1.06E-21
ZHX2	brown	-0.234292057	8.98E-07	0.428183978	1.34E-20
PRDM11	brown	-0.238886287	5.39E-07	0.325873825	4.27E-12
CCDC136	brown	-0.337566429	6.40E-13	0.535256607	3.03E-33
MYL12B	brown	0.280075955	3.43E-09	-0.44949848	8.96E-23
LMCD1	brown	-0.276208286	5.73E-09	0.685601266	5.43E-61
USF1	brown	0.260989781	3.98E-08	-0.310890203	4.34E-11
ZNF540	brown	-0.273915745	7.73E-09	0.42215591	5.18E-20
TMEM259	brown	0.279395665	3.76E-09	-0.457057782	1.39E-23
CDK15	brown	-0.35822232	1.83E-14	0.687959072	1.46E-61
THRA	brown	-0.335812937	8.55E-13	0.588826923	1.76E-41
ANKRD22	brown	0.077181518	0.109999368	-0.227269059	1.92E-06

ACTA1	brown	-0.223897138	2.75E-06	0.376554367	6.21E-16
YWHAE	brown	0.225650898	2.28E-06	-0.367280192	3.53E-15
C18orf21	brown	0.270651073	1.18E-08	-0.431802412	5.86E-21
FLAD1	brown	0.402690937	3.41E-18	-0.532751896	6.78E-33
CACNA1H	brown	-0.448427841	1.16E-22	0.765451676	5.73E-84
HEPH	brown	-0.291452222	7.26E-10	0.714358169	2.37E-68
ARRDC1	brown	0.279473767	3.72E-09	-0.478641692	5.25E-26
CCDC89	brown	-0.083068334	0.085341206	0.208591795	1.30E-05
SH3BGR	brown	-0.417320121	1.50E-19	0.636331699	3.47E-50
NTNG1	brown	-0.297062052	3.29E-10	0.537223329	1.60E-33
PCDHGB7	brown	-0.298046063	2.86E-10	0.60091287	1.47E-43
SPNS2	brown	-0.023320505	0.629633893	0.185914723	0.000105385
NME1-NME2	brown	0.232087485	1.14E-06	-0.478613926	5.29E-26
NFIX	brown	-0.360347739	1.25E-14	0.646930441	2.42E-52
CHRDL2	brown	-0.152616556	0.001502886	0.61167674	1.75E-45
ADCY4	brown	-0.237737442	6.13E-07	0.592214197	4.70E-42
MRGPRF	brown	-0.429400091	1.02E-20	0.730909152	5.13E-73
DACT3	brown	-0.432498398	5.00E-21	0.831940046	1.51E-111
GID8	brown	0.283232194	2.25E-09	-0.428876399	1.14E-20
JUP	brown	0.252685054	1.09E-07	-0.573634003	5.45E-39
CKM	brown	-0.194212393	5.03E-05	0.221991218	3.35E-06
VAMP8	brown	0.317281477	1.64E-11	-0.530249774	1.51E-32
KCNJ3	brown	-0.335875592	8.46E-13	0.474905023	1.42E-25
ALAS2	brown	-0.07746036	0.108716847	0.196380105	4.12E-05
TBC1D19	brown	-0.109390315	0.023294204	0.379791617	3.34E-16
SLC2A4	brown	-0.578329983	9.56E-40	0.848281809	3.07E-120
ATP13A1	brown	0.39069492	3.94E-17	-0.492942244	1.04E-27
RPP25	brown	0.150738489	0.001720826	-0.248797129	1.73E-07
TMEM216	brown	0.236020616	7.42E-07	-0.352024516	5.45E-14
PAFAH1B3	brown	0.411162068	5.70E-19	-0.597100394	6.81E-43
DRG2	brown	0.157439511	0.001054066	-0.22784765	1.81E-06
PLXNA4	brown	-0.171741894	0.00034681	0.518808532	5.32E-31
C8orf59	brown	0.251280236	1.29E-07	-0.439991107	8.73E-22
FNBP1	brown	-0.402751858	3.36E-18	0.779974047	3.73E-89
FABP3	brown	-0.076544297	0.112974958	0.163961252	0.000642042
MAMDC2	brown	-0.462023104	3.99E-24	0.653604809	9.60E-54
GFRA1	brown	-0.517940682	6.93E-31	0.611991508	1.53E-45
STX18	brown	0.237499169	6.29E-07	-0.250950414	1.34E-07
ZNF354C	brown	-0.112370536	0.019765561	0.338472247	5.50E-13
NAP1L2	brown	-0.364944961	5.42E-15	0.435281287	2.63E-21
CSTF1	brown	0.260056308	4.47E-08	-0.336557738	7.56E-13
ABI3BP	brown	-0.50433111	4.01E-29	0.842148794	7.32E-117
NAPRT	brown	0.148095819	0.002076714	-0.377095708	5.60E-16
MBNL1	brown	-0.283759937	2.09E-09	0.607158556	1.15E-44
MECR	brown	0.278208905	4.40E-09	-0.325360723	4.64E-12
PTCH2	brown	-0.316790576	1.77E-11	0.601041688	1.40E-43
TMEM44	brown	0.29305527	5.80E-10	-0.29761652	3.04E-10
DCUN1D3	brown	-0.40029404	5.60E-18	0.499717985	1.52E-28
NBEA	brown	-0.408358882	1.04E-18	0.618717585	8.76E-47
FAIM2	brown	-0.553104992	7.98E-36	0.727850142	3.97E-72
CRABP2	brown	0.076129994	0.114943266	-0.24699602	2.13E-07

BMP5	brown	-0.422382238	4.92E-20	0.325199667	4.76E-12
C1QTNF2	brown	-0.414380608	2.85E-19	0.66331204	7.55E-56
RASSF3	brown	-0.331630841	1.69E-12	0.675969176	1.03E-58
PGAP2	brown	0.245942012	2.41E-07	-0.508214228	1.28E-29
TMEM234	brown	0.30712072	7.62E-11	-0.392337067	2.83E-17
MAGEE1	brown	-0.102577895	0.033459896	0.226525218	2.08E-06
MFSD5	brown	0.244555489	2.83E-07	-0.490899535	1.84E-27
DLG2	brown	-0.481008097	2.78E-26	0.563491402	2.13E-37
HIPK4	brown	-0.358992349	1.59E-14	0.560916863	5.29E-37
SHISA3	brown	-0.379527626	3.51E-16	0.5141409	2.19E-30
LPP	brown	-0.399756587	6.26E-18	0.753152762	7.33E-80
NT5DC3	brown	-0.329629756	2.34E-12	0.675815778	1.12E-58
LY6K	brown	0.156996797	0.001089411	-0.273141233	8.55E-09
FARSA	brown	0.285998357	1.54E-09	-0.486463846	6.29E-27
FOXO1	brown	-0.225321107	2.36E-06	0.41345384	3.48E-19
CRTC2	brown	0.301886931	1.64E-10	-0.359704489	1.40E-14
ATL1	brown	-0.265602435	2.24E-08	0.482053038	2.10E-26
RPUSD2	brown	0.296333266	3.65E-10	-0.468142337	8.33E-25
GCSAML	brown	-0.37968455	3.41E-16	0.647773093	1.62E-52
SPARCL1	brown	-0.436117709	2.16E-21	0.826097324	1.15E-108
NUDT8	brown	0.26934949	1.39E-08	-0.538793805	9.60E-34
GKAP1	brown	-0.18100466	0.000160855	0.334903429	9.93E-13
SERINC2	brown	0.295824261	3.92E-10	-0.581403226	3.01E-40
TMEM63B	brown	0.202923219	2.24E-05	-0.426724691	1.86E-20
PEAR1	brown	-0.157998452	0.001010953	0.407689958	1.19E-18
SLC2A13	brown	-0.195016626	4.67E-05	0.520733444	2.95E-31
HECTD3	brown	0.262693173	3.22E-08	-0.264089608	2.71E-08
LPPR4	brown	-0.448240817	1.22E-22	0.45188304	5.00E-23
RABGGTA	brown	0.212220262	9.06E-06	-0.441440895	6.20E-22
POP5	brown	0.279871998	3.52E-09	-0.453176672	3.64E-23
FAM135B	brown	-0.430128	8.60E-21	0.268468841	1.56E-08
GMDS	brown	0.215981247	6.21E-06	-0.321549337	8.45E-12
TPM1	brown	-0.382183556	2.10E-16	0.743535866	8.16E-77
PRKD1	brown	-0.198657903	3.34E-05	0.585624395	6.05E-41
HDGFRP3	brown	-0.152398102	0.001526865	0.261755115	3.62E-08
ACSM5	brown	-0.291259801	7.46E-10	0.632433607	2.05E-49
NOVA2	brown	-0.041385213	0.391969688	0.386203127	9.59E-17
LRRC70	brown	-0.306236304	8.68E-11	0.618952216	7.91E-47
GPR17	brown	-0.336623646	7.48E-13	0.691208575	2.33E-62
BTNL9	brown	-0.12693929	0.008406907	0.211954704	9.30E-06
JAM2	brown	-0.510790219	5.98E-30	0.851888367	2.70E-122
HSPB7	brown	-0.384518555	1.33E-16	0.784405692	8.11E-91
PARK2	brown	-0.516878927	9.58E-31	0.674302435	2.50E-58
NOC4L	brown	0.295863243	3.90E-10	-0.489793706	2.51E-27
FBXO32	brown	-0.213356988	8.09E-06	0.614106543	6.27E-46
DDO	brown	-0.111647602	0.020575805	0.219599835	4.29E-06
ZNF205	brown	0.305169603	1.02E-10	-0.445053247	2.62E-22
WBSCR22	brown	0.267245976	1.82E-08	-0.517542859	7.83E-31
CRYM	brown	-0.364169427	6.24E-15	0.518054593	6.70E-31
KIAA1210	brown	-0.20989671	1.14E-05	0.281779624	2.73E-09
SCAMP2	brown	0.209672263	1.17E-05	-0.294630332	4.65E-10

RERG	brown	-0.396273659	1.28E-17	0.712148008	9.39E-68
NEXN	brown	-0.406821743	1.43E-18	0.798703526	1.84E-96
CCNJL	brown	0.082289898	0.088320558	-0.18846549	8.42E-05
ICAM2	brown	-0.180639331	0.000165925	0.31859143	1.34E-11
NOP10	brown	0.29051703	8.27E-10	-0.473513892	2.05E-25
SETBP1	brown	-0.318213564	1.42E-11	0.629314691	8.35E-49
EBF3	brown	-0.302909743	1.41E-10	0.636179666	3.72E-50
DBX2	brown	-0.151563176	0.001621785	0.261770711	3.61E-08
LINC00493	brown	0.171066895	0.000366232	-0.349071714	9.11E-14
SMTNL2	brown	-0.2084399	1.31E-05	0.386307455	9.39E-17
ARMC6	brown	0.325156816	4.79E-12	-0.425584583	2.41E-20
CACNB2	brown	-0.420407824	7.63E-20	0.787614711	4.79E-92
TRIM11	brown	0.331420033	1.75E-12	-0.498405507	2.22E-28
GIT1	brown	0.314086509	2.67E-11	-0.414316025	2.89E-19
LYPD3	brown	0.061468372	0.203327579	-0.319071017	1.24E-11
TRMU	brown	0.356236249	2.60E-14	-0.502917007	6.05E-29
COL21A1	brown	-0.358784119	1.65E-14	0.615817195	3.03E-46
CLEC14A	brown	-0.207188865	1.48E-05	0.501334688	9.55E-29
HPS6	brown	0.176662888	0.00023169	-0.330678359	1.98E-12
HSD17B10	brown	0.213314498	8.12E-06	-0.461982231	4.03E-24
ELTD1	brown	-0.229156754	1.57E-06	0.617553531	1.44E-46
PRKAR2B	brown	-0.337620178	6.34E-13	0.543635029	1.95E-34
CABP1	brown	-0.239494907	5.03E-07	0.313570711	2.89E-11
RNF7	brown	0.279030336	3.94E-09	-0.423509153	3.83E-20
GARNL3	brown	-0.342631046	2.74E-13	0.377389991	5.29E-16
RARA	brown	-0.15539289	0.001226807	0.261763598	3.62E-08
CYP2U1	brown	-0.225513876	2.32E-06	0.547945894	4.61E-35
TNNT2	brown	-0.300431438	2.03E-10	0.462699183	3.36E-24
LRRTM1	brown	-0.37897819	3.91E-16	0.385597928	1.08E-16
FAM124A	brown	-0.311112784	4.20E-11	0.704331858	1.10E-65
EHD4	brown	0.04506747	0.351185437	-0.196129802	4.22E-05
TNFAIP8L3	brown	-0.38586932	1.02E-16	0.703303473	2.04E-65
RRP7A	brown	0.242806707	3.46E-07	-0.399103586	7.16E-18
EIF6	brown	0.281528415	2.82E-09	-0.60520717	2.56E-44
PYCRL	brown	0.34991471	7.87E-14	-0.466949253	1.13E-24
DPH7	brown	0.260827474	4.06E-08	-0.392203215	2.91E-17
TSTA3	brown	0.350244414	7.44E-14	-0.520736004	2.95E-31
DEDD2	brown	0.252525714	1.11E-07	-0.452587247	4.21E-23
ITGB1BP2	brown	-0.405385348	1.94E-18	0.6416356	2.96E-51
ZDHHC8	brown	0.20937603	1.20E-05	-0.231744272	1.19E-06
FOXN3	brown	-0.345667265	1.64E-13	0.621911955	2.19E-47
ORMDL2	brown	0.28480725	1.81E-09	-0.519888738	3.82E-31
NUDT22	brown	0.251928787	1.19E-07	-0.450359081	7.26E-23
ZNF707	brown	0.320699907	9.65E-12	-0.325057324	4.87E-12
PFDN2	brown	0.287208488	1.31E-09	-0.402863252	3.29E-18
NUDT10	brown	-0.25672475	6.71E-08	0.539379436	7.93E-34
PTRH2	brown	0.299964091	2.17E-10	-0.483821096	1.30E-26
TMED3	brown	0.282519819	2.47E-09	-0.418627893	1.13E-19
RFX2	brown	-0.291830116	6.89E-10	0.231059894	1.28E-06
SLC50A1	brown	0.341319265	3.42E-13	-0.416254485	1.90E-19
TDRD10	brown	-0.245039073	2.67E-07	0.498291761	2.29E-28

CCDC110	brown	-0.292269294	6.48E-10	0.550418089	2.00E-35
RSPO2	brown	-0.251513647	1.25E-07	0.652330297	1.79E-53
FBXL19	brown	0.297625244	3.03E-10	-0.500935574	1.07E-28
CA3	brown	-0.232248751	1.12E-06	0.542287797	3.04E-34
RGS9BP	brown	-0.082383686	0.087957233	0.210794861	1.04E-05
HOXA11	brown	-0.078542786	0.103849631	0.194525855	4.89E-05
PRRG3	brown	-0.47320477	2.22E-25	0.718829407	1.41E-69
ZHX3	brown	-0.338060577	5.89E-13	0.604190711	3.89E-44
SLC35A2	brown	0.368870854	2.63E-15	-0.415802667	2.09E-19
CBX6	brown	-0.223360827	2.90E-06	0.429220312	1.06E-20
GAR1	brown	0.227783955	1.82E-06	-0.440048063	8.62E-22
PSMG2	brown	0.178234956	0.000203213	-0.281159445	2.97E-09
MAP3K8	brown	-0.252899856	1.06E-07	0.193958321	5.15E-05
CDH5	brown	-0.188306893	8.54E-05	0.594770003	1.72E-42
GID4	brown	-0.178586844	0.000197305	0.320794736	9.51E-12
SH3D19	brown	-0.307615873	7.08E-11	0.532280526	7.88E-33
APBB1	brown	-0.334605127	1.04E-12	0.649073645	8.67E-53
RIC3	brown	-0.297709706	3.00E-10	0.339903984	4.33E-13
CREBRF	brown	-0.29424958	4.90E-10	0.5953227	1.38E-42
ST14	brown	0.312326306	3.49E-11	-0.513986895	2.30E-30
IL17B	brown	-0.294350597	4.83E-10	0.633858191	1.07E-49
C1QTNF7	brown	-0.656092969	2.82E-54	0.777178221	3.98E-88
REEP4	brown	0.29398328	5.09E-10	-0.602149577	8.93E-44
MYH2	brown	-0.376080295	6.79E-16	0.445234766	2.51E-22
PHKG2	brown	0.282499233	2.48E-09	-0.446046073	2.07E-22
MYL9	brown	-0.37920662	3.74E-16	0.71494276	1.64E-68
TES	brown	-0.230877454	1.30E-06	0.384187264	1.42E-16
RSPO1	brown	-0.37070165	1.87E-15	0.527091492	4.08E-32
ADCK5	brown	0.310011162	4.95E-11	-0.525376469	6.99E-32
TCF21	brown	-0.628925759	9.94E-49	0.735386054	2.45E-74
LRRK2	brown	-0.449107531	9.85E-23	0.784420252	8.00E-91
CLDN11	brown	-0.17104033	0.000367016	0.361133978	1.08E-14
NR2C2AP	brown	0.35346783	4.24E-14	-0.548094387	4.38E-35
UBE2Q2	brown	-0.194444683	4.92E-05	0.412732344	4.07E-19
SYNC	brown	-0.340128765	4.17E-13	0.759180476	7.64E-82
WNT2B	brown	-0.382484582	1.98E-16	0.440996847	6.89E-22
SLC16A13	brown	0.276589858	5.44E-09	-0.293488275	5.46E-10
SLC25A39	brown	0.416642679	1.74E-19	-0.645863534	4.03E-52
CYTL1	brown	-0.344436562	2.02E-13	0.530132173	1.56E-32
GJB7	brown	0.211627697	9.61E-06	-0.257426907	6.16E-08
IGF1	brown	-0.185998726	0.000104615	0.595333397	1.38E-42
SMAGP	brown	0.156979009	0.001090854	-0.310782621	4.41E-11
SLC16A4	brown	-0.243245627	3.29E-07	0.373837517	1.04E-15
HDAC4	brown	-0.413054612	3.79E-19	0.688622359	1.01E-61
ASPA	brown	-0.588456861	2.03E-41	0.566901352	6.29E-38
BCHE	brown	-0.408702734	9.63E-19	0.627762667	1.67E-48
CLDN7	brown	0.208836844	1.26E-05	-0.468419095	7.75E-25
LDLRAD4	brown	-0.240035685	4.73E-07	0.545176101	1.17E-34
PRDM8	brown	-0.299536401	2.31E-10	0.518387224	6.05E-31
KCNMB1	brown	-0.475998752	1.06E-25	0.826354295	8.62E-109
TSEN34	brown	0.231295433	1.25E-06	-0.375906407	7.02E-16

CBX7	brown	-0.489412699	2.79E-27	0.626598554	2.80E-48
NT5C1A	brown	-0.153557016	0.00140355	0.384916587	1.23E-16
SH3BP2	brown	0.304845374	1.07E-10	-0.309261595	5.54E-11
PELI3	brown	-0.068394043	0.156840232	0.111153719	0.021145791
EXOSC1	brown	0.227106856	1.96E-06	-0.351000612	6.52E-14
SIRT7	brown	0.346782144	1.35E-13	-0.56263407	2.88E-37
TSHZ3	brown	-0.325868199	4.28E-12	0.715624535	1.07E-68
SKIV2L	brown	0.257036553	6.46E-08	-0.345683157	1.63E-13
EPHB1	brown	-0.272886885	8.83E-09	0.475034673	1.37E-25
TMEM88	brown	-0.308261998	6.43E-11	0.366188773	4.31E-15
FRZB	brown	-0.131350543	0.006378891	0.457227233	1.33E-23
SLC25A4	brown	-0.321646606	8.33E-12	0.37686706	5.85E-16
TAF6L	brown	0.305704116	9.39E-11	-0.45427332	2.78E-23
ALG8	brown	0.311152126	4.17E-11	-0.381821755	2.26E-16
SYPL2	brown	-0.351447837	6.03E-14	0.679512157	1.53E-59
PLXND1	brown	-0.012520891	0.795717731	0.398074694	8.84E-18
NEK9	brown	-0.187369306	9.28E-05	0.490786527	1.90E-27
EGR3	brown	-0.528001274	3.07E-32	0.625483489	4.58E-48
ADAMTS9	brown	-0.268210849	1.61E-08	0.682384998	3.20E-60
ACTC1	brown	-0.421650658	5.79E-20	0.763542969	2.58E-83
HSBP1L1	brown	0.271870102	1.01E-08	-0.428767977	1.17E-20
C8orf89	brown	-0.091842079	0.057046059	0.252061109	1.17E-07
DCHS1	brown	-0.343639615	2.31E-13	0.840040384	9.83E-116
GPS1	brown	0.3492477	8.84E-14	-0.46328253	2.90E-24
FAM212B	brown	-0.303243871	1.35E-10	0.314299669	2.59E-11
HSPA2	brown	-0.317590469	1.56E-11	0.44963354	8.67E-23
PPP1R3F	brown	-0.094515308	0.050160531	0.175157701	0.000262415
CCDC64	brown	0.175832646	0.000248194	-0.267545664	1.75E-08
NPR2	brown	-0.35017654	7.53E-14	0.636454166	3.28E-50
ADPGK	brown	0.399043155	7.25E-18	-0.305375088	9.86E-11
TPPP	brown	-0.556502108	2.47E-36	0.528270128	2.82E-32
C15orf59	brown	-0.30667426	8.14E-11	0.356087687	2.67E-14
MXRA7	brown	-0.363135652	7.53E-15	0.694432573	3.69E-63
MATN2	brown	-0.367430035	3.43E-15	0.482739169	1.74E-26
NDUFA12	brown	0.170374837	0.000387193	-0.324472666	5.34E-12
PITPNM2	brown	-0.229238564	1.56E-06	0.413785219	3.24E-19
TRPC3	brown	-0.419488026	9.34E-20	0.601015597	1.41E-43
TRPC4AP	brown	0.286003969	1.54E-09	-0.324241053	5.54E-12
MORN5	brown	-0.508956437	1.03E-29	0.71681791	5.04E-69
DCAF12L1	brown	-0.296801716	3.41E-10	0.365783356	4.65E-15
PDE3A	brown	-0.220462986	3.93E-06	0.660033396	3.96E-55
TMEM69	brown	0.35818278	1.84E-14	-0.520973139	2.74E-31
GATA6	brown	-0.454764614	2.46E-23	0.758816574	1.01E-81
SNRPD3	brown	0.28813598	1.15E-09	-0.448565806	1.12E-22
HSPB8	brown	-0.349657689	8.23E-14	0.523090367	1.42E-31
DTNB	brown	0.171971319	0.00034043	-0.239242564	5.18E-07
YIPF2	brown	0.312251313	3.53E-11	-0.314180027	2.64E-11
PPP1R14A	brown	-0.389640127	4.86E-17	0.638228343	1.45E-50
CX3CL1	brown	-0.254917917	8.35E-08	0.369624335	2.29E-15
ANKRD35	brown	-0.219843322	4.19E-06	0.298631607	2.63E-10
CD164L2	brown	0.128098132	0.007824924	-0.205975408	1.67E-05

COL14A1	brown	-0.43867554	1.19E-21	0.795735952	2.98E-95
DDRGK1	brown	0.221856178	3.40E-06	-0.369384932	2.39E-15
CD302	brown	-0.324367474	5.43E-12	0.653109989	1.22E-53
RAB30	brown	-0.275961817	5.91E-09	0.637466954	2.06E-50
PUS1	brown	0.331534257	1.72E-12	-0.568025358	4.20E-38
SMIM10	brown	-0.295938665	3.86E-10	0.553470655	7.04E-36
DST	brown	-0.155904384	0.001181349	0.295780536	3.95E-10
ANGPT2	brown	0.068938735	0.153556527	0.062645247	0.194792301
ACTG1	brown	0.149613411	0.001864888	-0.325477332	4.55E-12
FAM171A1	brown	-0.169847507	0.000403909	0.452563126	4.23E-23
COA6	brown	0.357559098	2.05E-14	-0.472085356	2.98E-25
DGKZ	brown	0.220515063	3.91E-06	-0.234477352	8.80E-07
JPH2	brown	-0.367439004	3.43E-15	0.705932879	4.20E-66
ARID5B	brown	-0.312301632	3.51E-11	0.587922467	2.50E-41
NEURL1B	brown	-0.226920522	1.99E-06	0.525179589	7.43E-32
PPP1CA	brown	0.368980694	2.58E-15	-0.613944467	6.72E-46
INO80B	brown	0.244603131	2.81E-07	-0.439189276	1.05E-21
MRVI1	brown	-0.425930704	2.23E-20	0.83484811	5.03E-113
TRIM61	brown	-0.138424988	0.004028218	0.227621396	1.85E-06
NFIB	brown	-0.274591803	7.08E-09	0.514858929	1.77E-30
ACACB	brown	-0.49797782	2.50E-28	0.720896003	3.74E-70
CTAGE8	brown	0.130333832	0.006802821	-0.227832336	1.81E-06
CTSG	brown	-0.394215031	1.94E-17	0.757758259	2.27E-81
DES	brown	-0.361363284	1.04E-14	0.670368824	1.99E-57
FAM117A	brown	-0.224199005	2.66E-06	0.368910682	2.61E-15
BMP4	brown	-0.153037019	0.001457703	0.322939713	6.80E-12
MRPS28	brown	0.152812082	0.001481717	-0.312755718	3.27E-11
IGSF8	brown	0.283240734	2.24E-09	-0.409889481	7.48E-19
ACTA2	brown	-0.368188279	2.98E-15	0.74571911	1.71E-77
SYNPO2	brown	-0.505184817	3.12E-29	0.858535017	3.11E-126
ANGPT1	brown	-0.179045961	0.000189837	0.430867305	7.27E-21
ADH1B	brown	-0.602718319	7.09E-44	0.731012378	4.79E-73
PRIMA1	brown	-0.514585329	1.92E-30	0.561048272	5.05E-37
ZNF154	brown	-0.300292647	2.07E-10	0.558965704	1.05E-36
MYH11	brown	-0.457355361	1.29E-23	0.7924479	6.15E-94
ISCA2	brown	0.31182589	3.77E-11	-0.341239275	3.46E-13
STAC	brown	-0.249727794	1.55E-07	0.362139116	9.02E-15
BCAP31	brown	0.264353431	2.62E-08	-0.587698962	2.72E-41
GSTP1	brown	0.101864832	0.034715531	-0.421078553	6.58E-20
STON1-GTF2	brown	-0.484983227	9.44E-27	0.732522608	1.73E-73
RBP4	brown	-0.125071027	0.009426663	0.255274988	7.99E-08
NELFCD	brown	0.350312787	7.35E-14	-0.511036938	5.55E-30
C1orf233	brown	0.214815062	6.99E-06	-0.300530627	2.00E-10
MRGPRE	brown	-0.244648209	2.80E-07	0.408857556	9.31E-19
DCAF12L2	brown	-0.195234932	4.58E-05	0.336563598	7.55E-13
CEP68	brown	-0.251892817	1.20E-07	0.471940679	3.10E-25
SCARF1	brown	-0.101761991	0.034899892	0.39752018	9.90E-18
PIH1D1	brown	0.270829893	1.15E-08	-0.433995255	3.54E-21
SMARCD2	brown	0.270315594	1.23E-08	-0.409607965	7.94E-19
CORO1B	brown	0.238429235	5.67E-07	-0.454566954	2.58E-23
CASP8	brown	0.173841005	0.000292373	-0.246596228	2.23E-07

CYGB	brown	-0.31513245	2.28E-11	0.468904656	6.84E-25
AQP1	brown	-0.414316305	2.89E-19	0.714659193	1.96E-68
SLC25A25	brown	-0.478546594	5.39E-26	0.562404903	3.13E-37
MRPS9	brown	0.093414926	0.052905802	-0.311474971	3.97E-11
PRDM5	brown	-0.222342694	3.23E-06	0.44477505	2.80E-22
CLIC4	brown	-0.234819506	8.47E-07	0.638527524	1.26E-50
RPUSD1	brown	0.348977682	9.26E-14	-0.520186628	3.49E-31
NOSTRIN	brown	-0.208754535	1.27E-05	0.335411599	9.13E-13
ZMAT1	brown	-0.193795063	5.22E-05	0.306129037	8.82E-11
PELP1	brown	0.152127402	0.001557066	-0.294455593	4.76E-10
DYSF	brown	-0.234336615	8.94E-07	0.638229222	1.45E-50
GPR115	brown	0.13822153	0.004083022	-0.291002036	7.73E-10
FAM124B	brown	-0.154240152	0.001335217	0.626490159	2.94E-48
ACTN2	brown	-0.379481455	3.55E-16	0.598884551	3.34E-43
STXBP6	brown	-0.278881437	4.02E-09	0.471860731	3.16E-25
KLHL42	brown	-0.253866474	9.47E-08	0.586122171	5.00E-41
MSX1	brown	-0.254259867	9.03E-08	0.434547011	3.12E-21
MAP6	brown	-0.229211585	1.56E-06	0.41182797	4.94E-19
RBKS	brown	-0.320258447	1.03E-11	0.293405062	5.52E-10
AC004754.3	brown	-0.301164156	1.82E-10	0.422864428	4.42E-20
ANXA8L1	brown	0.130839661	0.006588854	-0.296615121	3.51E-10
RPS6KB2	brown	0.253523513	9.87E-08	-0.467511447	9.81E-25
MTA2	brown	0.268807612	1.49E-08	-0.524671193	8.71E-32
FERMT1	brown	0.233622169	9.67E-07	-0.377956633	4.75E-16
SCUBE3	brown	-0.259942962	4.53E-08	0.582032146	2.38E-40
MLXIP	brown	-0.278111498	4.45E-09	0.582865099	1.73E-40
MYO18B	brown	-0.242438325	3.60E-07	0.314960531	2.34E-11
CTAGE4	brown	0.133393486	0.005597965	-0.209246923	1.21E-05
CFD	brown	-0.509559279	8.62E-30	0.533006409	6.25E-33
STX6	brown	0.259346231	4.87E-08	-0.388415151	6.19E-17
CDKN1C	brown	-0.172209421	0.000333925	0.26720377	1.83E-08
PPARGC1A	brown	-0.227218865	1.93E-06	0.340379182	4.00E-13
SPR	brown	0.157567452	0.001044051	-0.247866271	1.93E-07
NACAD	brown	-0.28362985	2.13E-09	0.505075493	3.22E-29
PANO1	brown	0.220247369	4.02E-06	-0.254514814	8.76E-08
DAGLB	brown	0.358403729	1.77E-14	-0.409472278	8.17E-19
IL17RA	brown	0.214511938	7.20E-06	-0.17591174	0.000246576
CSTA	brown	0.089663709	0.063220549	-0.323592503	6.13E-12
RP11-195F1	brown	-0.144329342	0.0027008	0.267656989	1.73E-08
YARS2	brown	0.317785973	1.52E-11	-0.483606723	1.37E-26
C12orf45	brown	0.212301201	8.98E-06	-0.317713552	1.53E-11
RASSF2	brown	-0.172707657	0.000320686	0.407713267	1.19E-18
EIF1B	brown	-0.182934507	0.0001364	0.298785412	2.57E-10
SCRIB	brown	0.256794152	6.65E-08	-0.477005521	8.13E-26
GPR20	brown	-0.067835708	0.160260958	0.215398009	6.59E-06
MAP1LC3A	brown	-0.105703826	0.028402322	0.171660493	0.0003491
DLG4	brown	-0.088634999	0.066322199	0.323815922	5.92E-12
UFSP2	brown	-0.264883586	2.45E-08	0.399889174	6.09E-18
LRRC45	brown	0.403549036	2.85E-18	-0.520733974	2.95E-31
FGF2	brown	-0.430927488	7.17E-21	0.749980546	7.68E-79
NTF3	brown	-0.381471883	2.41E-16	0.327217911	3.45E-12

ELF4	brown	0.182608102	0.000140275	-0.322569101	7.21E-12
PIN4	brown	0.231736107	1.19E-06	-0.399041334	7.25E-18
ADCY5	brown	-0.482334998	1.94E-26	0.762957823	4.09E-83
MRPS18A	brown	0.122221697	0.011194051	-0.3627281	8.11E-15
CLDN5	brown	-0.364561939	5.81E-15	0.542963036	2.43E-34
PRR13	brown	0.216864839	5.68E-06	-0.351133591	6.37E-14
DMKN	brown	0.097661875	0.042959287	-0.201551256	2.55E-05
PDK2	brown	-0.173273759	0.000306239	0.315145134	2.28E-11
PPAP2B	brown	-0.433804588	3.70E-21	0.663101143	8.41E-56
PCDH10	brown	-0.153113955	0.001449572	0.377009123	5.69E-16
TNIK	brown	-0.197685837	3.66E-05	0.294833222	4.51E-10
ANXA8	brown	0.124206539	0.009934678	-0.292080085	6.65E-10
ITGA3	brown	0.131388256	0.006363632	-0.276888892	5.23E-09
VARS	brown	0.309703956	5.18E-11	-0.416111763	1.96E-19
ZBTB16	brown	-0.497325792	3.02E-28	0.734848066	3.54E-74
HSPB2	brown	-0.414111834	3.02E-19	0.70350818	1.80E-65
DERL2	brown	0.218410179	4.85E-06	-0.25837837	5.49E-08
CBLC	brown	0.213604139	7.89E-06	-0.495976458	4.42E-28
NMB	brown	0.27409424	7.55E-09	-0.357397571	2.11E-14
MRM1	brown	0.341007447	3.60E-13	-0.418479753	1.17E-19
CCND2	brown	-0.292406282	6.35E-10	0.546941662	6.46E-35
TLE3	brown	0.127528726	0.008106233	-0.204981057	1.84E-05
CLIP3	brown	-0.375664428	7.35E-16	0.787248537	6.63E-92
RGAG4	brown	-0.360879487	1.13E-14	0.567405457	5.25E-38
CLNS1A	brown	0.192204823	6.03E-05	-0.349491291	8.47E-14
PRR29	brown	-0.040686378	0.400021217	0.069308156	0.151359279
WDR74	brown	0.274738851	6.94E-09	-0.50784919	1.43E-29
CAMLG	brown	-0.137785316	0.004202801	0.232988642	1.04E-06
NHSL2	brown	-0.331287134	1.79E-12	0.646816011	2.56E-52
ABCC10	brown	0.298255556	2.77E-10	-0.326222676	4.04E-12
PIP5KL1	brown	0.155986613	0.001174188	-0.243706083	3.12E-07
SOX5	brown	-0.408064483	1.10E-18	0.662520337	1.13E-55
FAM218A	brown	-0.080966567	0.093576669	0.259041404	5.06E-08
FAM131A	brown	-0.179740924	0.000179036	0.210394748	1.09E-05
LMO3	brown	-0.424058944	3.39E-20	0.706695837	2.65E-66
TBRG4	brown	0.365523233	4.87E-15	-0.590510875	9.15E-42
AHSA1	brown	0.26672988	1.94E-08	-0.366695873	3.93E-15
SEL1L2	brown	-0.221830501	3.41E-06	0.513823665	2.41E-30
QPCTL	brown	0.287731657	1.22E-09	-0.388513688	6.08E-17
MGLL	brown	-0.361214106	1.07E-14	0.516134181	1.20E-30
ATP1B2	brown	-0.484110475	1.20E-26	0.678025705	3.42E-59
PTGIS	brown	-0.355316774	3.06E-14	0.733557164	8.54E-74
ARHGEF26	brown	-0.288278619	1.13E-09	0.432009415	5.59E-21
TELO2	brown	0.270802226	1.16E-08	-0.465969283	1.46E-24
CNIH4	brown	0.304191508	1.17E-10	-0.44379331	3.54E-22
IRF6	brown	0.161050399	0.000802891	-0.436655356	1.91E-21
ACSS3	brown	-0.149269936	0.00191102	0.302733002	1.45E-10
STAP2	brown	0.259633947	4.70E-08	-0.558126208	1.41E-36
ACOT13	brown	0.207540367	1.43E-05	-0.260336065	4.31E-08
LONRF3	brown	-0.210086178	1.12E-05	0.281765235	2.74E-09
SCARA5	brown	-0.687940632	1.47E-61	0.780455014	2.47E-89

SCARA3	brown	-0.190263955	7.18E-05	0.398629981	7.89E-18
RCAN2	brown	-0.499158277	1.79E-28	0.824534947	6.50E-108
CSDC2	brown	-0.271054187	1.12E-08	0.705610088	5.11E-66
DBI	brown	0.251214366	1.30E-07	-0.407982372	1.12E-18
SERPINB5	brown	-0.003700032	0.939019393	-0.251539511	1.25E-07
STX1A	brown	0.196597944	4.04E-05	-0.268785229	1.50E-08
GYG2	brown	-0.067672408	0.161271985	0.294009539	5.07E-10
CPSF3L	brown	0.237196153	6.51E-07	-0.428168867	1.34E-20
GSPT2	brown	-0.133828885	0.005443084	0.270394713	1.22E-08
EDNRB	brown	-0.33255301	1.46E-12	0.564997369	1.24E-37
DISP1	brown	-0.322127907	7.72E-12	0.525821892	6.08E-32
ATPIF1	brown	0.326921766	3.62E-12	-0.597833451	5.08E-43
SMPDL3A	brown	-0.162834486	0.000700388	0.20950261	1.18E-05
FAM129A	brown	-0.376181592	6.66E-16	0.740132161	9.07E-76
COPS5	brown	0.256505579	6.89E-08	-0.368380771	2.88E-15
RP11-766F1	brown	-0.242750508	3.48E-07	0.490830432	1.88E-27
PDE1A	brown	-0.466326371	1.33E-24	0.884691562	6.01E-144
S1PR1	brown	-0.325467685	4.56E-12	0.727503859	4.99E-72
SYBU	brown	-0.178156079	0.00020456	0.307861525	6.82E-11
KCNH2	brown	-0.382210575	2.09E-16	0.619557953	6.09E-47
GIPC3	brown	-0.011740038	0.808201918	0.324274776	5.51E-12
EGFL7	brown	-0.140471664	0.003512812	0.290767244	7.99E-10
IFNGR2	brown	0.24768763	1.97E-07	-0.287593906	1.24E-09
MGAT4B	brown	0.342950419	2.60E-13	-0.418673778	1.12E-19
EDA2R	brown	-0.140801496	0.003435588	0.341714259	3.20E-13
DVL1	brown	0.175137225	0.000262858	-0.375213255	8.01E-16
AUP1	brown	0.40436149	2.40E-18	-0.622251934	1.89E-47
ATP5J2	brown	0.373153558	1.18E-15	-0.601927612	9.78E-44
CCDC50	brown	-0.19755629	3.70E-05	0.507316874	1.67E-29
DTNA	brown	-0.474165503	1.73E-25	0.78920646	1.16E-92
MBNL2	brown	-0.140918225	0.003408629	0.378229809	4.51E-16
RND1	brown	-0.286637429	1.41E-09	0.227249354	1.93E-06
TFAP4	brown	0.336380148	7.79E-13	-0.463250641	2.92E-24
MAPRE2	brown	-0.263966244	2.75E-08	0.715500096	1.16E-68
BRE	brown	-0.151727131	0.001602729	0.163637565	0.000658324
FLT4	brown	-0.045745693	0.343976676	0.38516966	1.17E-16
AKT3	brown	-0.269378153	1.39E-08	0.673668814	3.50E-58
WWOX	brown	-0.133853459	0.005434459	0.243607657	3.15E-07
FBXO31	brown	-0.28504941	1.75E-09	0.516591419	1.05E-30
ARNT2	brown	-0.15059866	0.001738158	0.2873404	1.28E-09
OPN1SW	brown	-0.179669081	0.000180125	0.532390952	7.61E-33
PLCB3	brown	0.322484449	7.30E-12	-0.53556768	2.74E-33
SELENBP1	brown	-0.215615331	6.44E-06	0.181990186	0.000147895
CD58	brown	0.080526427	0.095379261	-0.276744891	5.33E-09
LYNX1	brown	-0.231749727	1.19E-06	0.20282733	2.26E-05
TMTC1	brown	-0.286981915	1.35E-09	0.54636597	7.83E-35
INMT-FAM18	brown	-0.565532524	1.03E-37	0.622052694	2.06E-47
MOV10	brown	0.332629979	1.44E-12	-0.427993848	1.40E-20
RRS1	brown	0.169667709	0.00040976	-0.350193477	7.50E-14
SLC22A18	brown	0.129215066	0.007298307	-0.329763024	2.29E-12
IER2	brown	-0.227290006	1.92E-06	0.06029199	0.212127254

C2CD4D	brown	0.104651467	0.030026314	-0.18576058	0.000106811
CIAO1	brown	0.269173019	1.42E-08	-0.355890557	2.76E-14
MBD2	brown	0.105600415	0.028558492	-0.267562864	1.75E-08
SEMA4C	brown	-0.101881493	0.034685742	0.183783152	0.000126791
LGI4	brown	-0.456139157	1.75E-23	0.564276999	1.61E-37
THBS4	brown	-0.051888917	0.283009724	0.555762581	3.19E-36
TSHB	brown	-0.46701255	1.12E-24	0.656160701	2.73E-54
LXN	brown	-0.04262059	0.377978331	0.273000561	8.70E-09
COX4I2	brown	-0.141958202	0.003176782	0.191635342	6.35E-05
MSANTD3-TM	brown	-0.186043578	0.000104206	0.424169201	3.31E-20
TCEAL4	brown	-0.206216122	1.63E-05	0.24538355	2.57E-07
PBXIP1	brown	-0.27857124	4.19E-09	0.361011949	1.11E-14
OR5K2	brown	-0.240637983	4.42E-07	0.457926454	1.12E-23
ABRACL	brown	0.329118095	2.54E-12	-0.506813829	1.94E-29
STK40	brown	-0.343812051	2.24E-13	0.424643292	2.97E-20
MFSD10	brown	0.294628071	4.65E-10	-0.517692503	7.48E-31
GPR135	brown	-0.286298783	1.48E-09	0.652990558	1.30E-53
PLEK2	brown	0.201641736	2.53E-05	-0.459714476	7.15E-24
CIB1	brown	0.177087833	0.000223645	-0.450257696	7.45E-23
PABPC5	brown	-0.335329944	9.26E-13	0.612924005	1.03E-45
EEF1E1	brown	0.328190922	2.95E-12	-0.427108056	1.71E-20
ZDBF2	brown	-0.201374351	2.59E-05	0.361258542	1.06E-14
GDF10	brown	-0.515596985	1.41E-30	0.64626645	3.33E-52
SNTA1	brown	-0.205309382	1.78E-05	0.268065997	1.64E-08
MS4A2	brown	-0.403631975	2.80E-18	0.706346301	3.27E-66
ARL10	brown	-0.084967332	0.078412008	0.351854271	5.62E-14
RHOJ	brown	-0.376406847	6.38E-16	0.787866276	3.83E-92
CACTIN	brown	0.24298621	3.38E-07	-0.360706613	1.17E-14
AXIN1	brown	0.162771045	0.000703815	-0.314821196	2.39E-11
PA2G4	brown	0.241384347	4.06E-07	-0.501305405	9.64E-29
PIAS4	brown	0.255576793	7.71E-08	-0.379396141	3.60E-16
VAMP2	brown	-0.286549853	1.43E-09	0.389371819	5.12E-17
OSBPL1A	brown	-0.220276324	4.00E-06	0.40081941	5.02E-18
ADIPOR1	brown	0.167420899	0.000489852	-0.250785366	1.37E-07
TMUB1	brown	0.377829896	4.87E-16	-0.548041638	4.46E-35
TRIM27	brown	0.276526055	5.49E-09	-0.3621786	8.96E-15
CSRP1	brown	-0.459901981	6.82E-24	0.76872451	4.19E-85
PCDHGA9	brown	-0.161530818	0.000774004	0.460680402	5.61E-24
IL23A	brown	0.105484874	0.028733852	-0.186397315	0.000101035
G6PC3	brown	0.23799761	5.95E-07	-0.340616774	3.85E-13
ADPRM	brown	-0.158732416	0.000956808	0.274183683	7.46E-09
HM13	brown	0.359772561	1.38E-14	-0.423358186	3.96E-20
COA4	brown	0.269691224	1.33E-08	-0.4746122	1.53E-25
RAP1A	brown	-0.360952893	1.12E-14	0.622523921	1.68E-47
LMO4	brown	-0.066119038	0.171130442	0.166763617	0.000515898
SLC24A3	brown	-0.381025851	2.63E-16	0.562152083	3.42E-37
RANBP3L	brown	-0.507495904	1.59E-29	0.82437517	7.75E-108
CTTN	brown	0.129013357	0.007390998	-0.289203821	9.92E-10
C14orf80	brown	0.250922857	1.34E-07	-0.46157788	4.47E-24
LPAR5	brown	0.201217918	2.63E-05	-0.266948781	1.89E-08
LRRC2	brown	-0.597743363	5.27E-43	0.833418957	2.69E-112

DGKG	brown	-0.347945864	1.11E-13	0.501838571	8.26E-29
PRAC2	brown	-0.201550963	2.55E-05	0.1864486	0.000100583
SLC19A1	brown	0.353146935	4.48E-14	-0.36838142	2.88E-15
CPNE6	brown	-0.422298228	5.02E-20	0.523154704	1.40E-31
MPHOSPH6	brown	0.15989608	0.000876424	-0.314575354	2.48E-11
FBXL6	brown	0.441275726	6.45E-22	-0.574141615	4.52E-39
BMPER	brown	-0.179859551	0.000177251	0.280134693	3.40E-09
RHOQ	brown	-0.169565472	0.000413123	0.397141052	1.07E-17
ARHGEF15	brown	-0.245261482	2.61E-07	0.539398939	7.88E-34
RASGEF1C	brown	-0.209068965	1.24E-05	0.229327128	1.54E-06
BAX	brown	0.370290117	2.02E-15	-0.425234626	2.60E-20
TLDC1	brown	0.1639959	0.000640322	-0.322498322	7.29E-12
ZFP2	brown	-0.279844726	3.54E-09	0.457553246	1.23E-23
GSS	brown	0.349769533	8.08E-14	-0.592466357	4.26E-42
STK32B	brown	-0.306914315	7.85E-11	0.662825731	9.67E-56
MCRS1	brown	0.279105892	3.90E-09	-0.414744987	2.63E-19
TFAP2A	brown	0.190460305	7.06E-05	-0.326778513	3.70E-12
KLHL41	brown	-0.580561826	4.14E-40	0.821497382	1.80E-106
NME1	brown	0.461708922	4.32E-24	-0.631793122	2.74E-49
CAPN6	brown	-0.110244297	0.022231268	0.303434039	1.31E-10
FIGLA	brown	-0.172439448	0.000327751	0.173359557	0.000304103
IER5L	brown	0.366173825	4.32E-15	-0.354671152	3.43E-14
TRPA1	brown	-0.247340858	2.05E-07	0.221370438	3.57E-06
CSNK1A1L	brown	-0.11849949	0.01394124	0.264888233	2.45E-08
KIT	brown	-0.210557078	1.07E-05	0.322543482	7.23E-12
ATP6V1G1	brown	0.16586668	0.00055352	-0.288868802	1.04E-09
SEMA3E	brown	-0.393736922	2.14E-17	0.480684627	3.03E-26
CAPZA2	brown	-0.248229124	1.85E-07	0.501784986	8.39E-29
EPM2A	brown	-0.479444626	4.24E-26	0.740222532	8.51E-76
GRIN2D	brown	0.23607211	7.38E-07	-0.256563665	6.84E-08
SHE	brown	-0.336004286	8.28E-13	0.729938618	9.85E-73
PCDHB4	brown	-0.178615616	0.000196829	0.301179347	1.82E-10
AKAP6	brown	-0.481675125	2.32E-26	0.835556845	2.17E-113
KRT10	brown	0.12443134	0.009800288	-0.309008983	5.75E-11
MANF	brown	0.348119726	1.07E-13	-0.360433592	1.23E-14
TPSG1	brown	-0.51466033	1.87E-30	0.76724714	1.37E-84
PCSK1	brown	-0.042526283	0.379035481	0.403275095	3.02E-18
CAMK1	brown	-0.201701374	2.51E-05	0.501866401	8.19E-29
SEMA6D	brown	-0.157361022	0.001060255	0.413700699	3.30E-19
HECW2	brown	0.04370688	0.365932276	0.277091481	5.10E-09
KCNQ4	brown	-0.345142248	1.79E-13	0.594657929	1.80E-42
VWF	brown	-0.203058486	2.21E-05	0.562117605	3.46E-37
SDF2	brown	0.205754897	1.71E-05	-0.308607896	6.11E-11
RASL11A	brown	-0.345780787	1.60E-13	0.519714441	4.03E-31
VSTM4	brown	-0.468656372	7.29E-25	0.822375668	6.93E-107
PALM	brown	-0.245380249	2.57E-07	0.476552797	9.17E-26
EBP	brown	0.35987745	1.36E-14	-0.58066829	3.98E-40
ARMCX1	brown	-0.283000031	2.32E-09	0.527036794	4.15E-32
TREX2	brown	0.112713563	0.019390916	-0.21055575	1.07E-05
BDKRB1	brown	-0.4015229	4.34E-18	0.449679231	8.57E-23
SYMPK	brown	0.308458061	6.24E-11	-0.385833141	1.03E-16

TLN2	brown	-0.197281142	3.80E-05	0.404594786	2.29E-18
LYPLA2	brown	0.370270229	2.03E-15	-0.604956434	2.84E-44
GTPBP3	brown	0.389059365	5.45E-17	-0.508322987	1.24E-29
FAM167B	brown	0.05273374	0.27523489	0.140715516	0.003455569
FGF13	brown	-0.292707228	6.09E-10	0.458951287	8.67E-24
PKP3	brown	0.274418597	7.24E-09	-0.570774031	1.55E-38
ARHGEF5	brown	0.158644446	0.000963154	-0.323459057	6.26E-12
PERP	brown	0.165693626	0.000561065	-0.428709954	1.19E-20
MEOX2	brown	-0.293924362	5.13E-10	0.657198995	1.63E-54
EIF4EBP1	brown	0.316707414	1.79E-11	-0.383566612	1.61E-16
ZFHX4	brown	-0.280102357	3.42E-09	0.558873022	1.08E-36
KHDRBS3	brown	-0.099275735	0.039617139	0.287594842	1.24E-09
LIMS2	brown	-0.466086553	1.42E-24	0.806400851	1.08E-99
SAMD4A	brown	-0.365150218	5.22E-15	0.641243854	3.56E-51
LHX4-AS1	brown	0.268476733	1.56E-08	-0.301301293	1.79E-10
CRYAB	brown	-0.404775986	2.20E-18	0.707728772	1.42E-66
DCLK2	brown	-0.184444472	0.000119748	0.545679298	9.86E-35
SOBP	brown	-0.496941811	3.36E-28	0.828822385	5.36E-110
MAPKAPK3	brown	0.142856755	0.002988099	-0.237512629	6.28E-07
VIPR2	brown	-0.59741603	6.01E-43	0.620778414	3.59E-47
COLEC11	brown	-0.217682555	5.22E-06	0.26383605	2.80E-08
GSTK1	brown	0.192810843	5.71E-05	-0.294186767	4.95E-10
PYGB	brown	-0.237098847	6.58E-07	0.388573658	6.00E-17
RGS22	brown	-0.550705195	1.81E-35	0.743127934	1.09E-76
SYCE3	brown	0.137063909	0.004407875	-0.225288131	2.37E-06
BVES	brown	-0.338286523	5.68E-13	0.615643739	3.27E-46
HSPB6	brown	-0.507522907	1.57E-29	0.816258541	4.76E-104
TRMT2A	brown	0.333379855	1.27E-12	-0.480986709	2.80E-26
SCAMP4	brown	0.296333023	3.65E-10	-0.384144735	1.44E-16
NOS3	brown	-0.143493212	0.00286068	0.483380743	1.46E-26
ABL1	brown	-0.346359043	1.45E-13	0.685104536	7.16E-61
NFS1	brown	0.280090024	3.42E-09	-0.369873672	2.18E-15
DTD1	brown	0.247538461	2.00E-07	-0.357222664	2.18E-14
MEN1	brown	0.487881186	4.26E-27	-0.603015189	6.28E-44
MITD1	brown	0.282000228	2.65E-09	-0.350904549	6.63E-14
SRR	brown	-0.094881511	0.049273723	0.265194094	2.36E-08
ZSWIM4	brown	0.239726327	4.90E-07	-0.193692709	5.27E-05
DCP1B	brown	-0.109335753	0.023363585	0.249338735	1.62E-07
MAGOH	brown	0.306277694	8.63E-11	-0.524679372	8.69E-32
PYGM	brown	-0.607446724	1.02E-44	0.864831354	3.74E-130
HES4	brown	0.171551014	0.000352202	-0.355460958	2.98E-14
FAM32A	brown	0.204269023	1.97E-05	-0.295442053	4.14E-10
ARTN	brown	0.14261262	0.003038329	-0.271713985	1.03E-08
CD93	brown	-0.213571284	7.92E-06	0.634132978	9.48E-50
DIRC3	brown	-0.235299769	8.03E-07	0.452662464	4.13E-23
DPY30	brown	0.333254003	1.30E-12	-0.493299696	9.42E-28
KLHL30	brown	-0.299002722	2.49E-10	0.460436312	5.96E-24
NEDD9	brown	-0.155695895	0.001199688	0.2438665	3.06E-07
TMEM136	brown	-0.130856485	0.006581843	0.371075887	1.74E-15
BAI3	brown	-0.447857424	1.33E-22	0.601776809	1.04E-43
PEAK1	brown	-0.214003336	7.58E-06	0.662067884	1.42E-55

INMT	brown	-0.389512583	4.98E-17	0.679678106	1.40E-59
IL3RA	brown	-0.171034422	0.000367191	0.422478597	4.82E-20
MRPS15	brown	0.35065942	6.92E-14	-0.540331994	5.80E-34
PER1	brown	-0.414926165	2.53E-19	0.551996647	1.17E-35
LCN10	brown	-0.30432917	1.15E-10	0.501189613	9.96E-29
LAMB3	brown	0.066268904	0.170160158	-0.274535778	7.13E-09
B4GAT1	brown	-0.126858294	0.008448991	0.346852648	1.34E-13
EXOSC5	brown	0.275192753	6.54E-09	-0.514422841	2.01E-30
NAALAD2	brown	-0.184463433	0.000119552	0.32291458	6.82E-12
DIRAS3	brown	-0.270504344	1.20E-08	0.578374884	9.40E-40
HRSP12	brown	0.170490638	0.000383609	-0.262151034	3.45E-08
KBTBD13	brown	-0.268083997	1.64E-08	0.597206619	6.53E-43
DUS2	brown	0.235856626	7.55E-07	-0.407573417	1.22E-18
ADAMTSL3	brown	-0.620247794	4.52E-47	0.841666234	1.33E-116
SHF	brown	-0.177355874	0.000218705	0.239365501	5.10E-07
BLOC1S2	brown	0.270631158	1.18E-08	-0.349558872	8.38E-14
TLN1	brown	-0.334023094	1.15E-12	0.743410515	8.92E-77
ANGPTL7	brown	-0.486490399	6.25E-27	0.792645959	5.14E-94
P2RX1	brown	-0.495141614	5.60E-28	0.828569346	7.14E-110
SDPR	brown	-0.526416615	5.05E-32	0.835081214	3.82E-113
CYYR1	brown	-0.310071394	4.91E-11	0.607608968	9.53E-45
GNB1L	brown	0.175241574	0.000260607	-0.27093047	1.14E-08
LSM11	brown	-0.278153725	4.43E-09	0.677100536	5.63E-59
TMEM54	brown	0.216016875	6.19E-06	-0.516694971	1.01E-30
LAD1	brown	0.240205391	4.64E-07	-0.524077823	1.05E-31
CA9	brown	0.196975594	3.90E-05	-0.359947977	1.34E-14
MRPL15	brown	0.25179479	1.21E-07	-0.380093921	3.15E-16
RCVRN	brown	-0.350421975	7.21E-14	0.619752563	5.60E-47
AGRN	brown	0.389653364	4.84E-17	-0.469414462	5.99E-25
FXYD1	brown	-0.584683507	8.67E-41	0.651545955	2.62E-53
PPP2R5A	brown	-0.283519065	2.16E-09	0.293017166	5.83E-10
GALT	brown	0.120527473	0.012378732	-0.213053347	8.34E-06
TSFM	brown	0.252669195	1.09E-07	-0.416275823	1.89E-19
TRABD	brown	0.26193026	3.54E-08	-0.531783066	9.24E-33
ZDHHC16	brown	0.402242091	3.74E-18	-0.486468126	6.29E-27
ZFPM2	brown	-0.403992847	2.60E-18	0.79275311	4.66E-94
COPZ1	brown	0.36892753	2.60E-15	-0.448519307	1.14E-22
PRRG2	brown	0.253772483	9.58E-08	-0.515633614	1.40E-30
PTPN6	brown	0.283966693	2.03E-09	-0.409518112	8.09E-19
PTH1R	brown	-0.459316936	7.91E-24	0.68984835	5.04E-62
TRMT1	brown	0.255439834	7.84E-08	-0.461215853	4.90E-24
KLF2	brown	-0.473705597	1.95E-25	0.670622787	1.74E-57
PCDHB7	brown	-0.111123247	0.021181404	0.323018765	6.71E-12
KIAA1462	brown	-0.24763026	1.98E-07	0.67407064	2.83E-58
TRUB2	brown	0.266230963	2.07E-08	-0.440168964	8.37E-22
WIPF3	brown	-0.12815062	0.007799437	0.34313516	2.52E-13
FLNC	brown	-0.453770381	3.15E-23	0.822173039	8.63E-107
C1QBP	brown	0.127437123	0.008152322	-0.291891763	6.83E-10
CUX1	brown	-0.134106239	0.005346444	0.333431229	1.26E-12
ANKZF1	brown	0.237509755	6.29E-07	-0.419738737	8.84E-20
ACOT1	brown	-0.126876793	0.008439363	0.348324833	1.04E-13

IRF3	brown	0.329880476	2.25E-12	-0.525744417	6.23E-32
ICT1	brown	0.333530599	1.24E-12	-0.529297813	2.04E-32
SMUG1	brown	0.402490772	3.55E-18	-0.554120102	5.63E-36
GATA5	brown	-0.467826139	9.04E-25	0.798908517	1.52E-96
SLITRK3	brown	-0.205267865	1.79E-05	0.455460426	2.07E-23
C10orf107	brown	-0.25658097	6.83E-08	0.475353174	1.26E-25
PLCB4	brown	-0.439248929	1.04E-21	0.611261048	2.08E-45
VTN	brown	-0.070139682	0.146500933	0.3576138	2.03E-14
ANGPTL5	brown	-0.246186609	2.34E-07	0.25968974	4.67E-08
ANP32A	brown	0.274865495	6.83E-09	-0.391250527	3.52E-17
CXorf40B	brown	0.154628662	0.001297732	-0.263026235	3.09E-08
TACR2	brown	-0.459885271	6.85E-24	0.77635468	7.95E-88
ZNF575	brown	-0.044229064	0.360227613	0.255487392	7.79E-08
ITGA7	brown	-0.367358317	3.48E-15	0.801035859	2.00E-97
IGSF9	brown	0.259003309	5.08E-08	-0.404598775	2.29E-18
FGF6	brown	-0.41770887	1.38E-19	0.557140673	1.98E-36
CDH3	brown	0.208764132	1.27E-05	-0.299284483	2.39E-10
ZDHHC15	brown	-0.216434189	5.93E-06	0.379702015	3.40E-16
ELOVL1	brown	0.226749556	2.03E-06	-0.41752267	1.44E-19
PRKD2	brown	0.293071338	5.79E-10	-0.488227613	3.87E-27
GPR39	brown	0.116528934	0.015623108	-0.221997392	3.35E-06
PI16	brown	-0.670515214	1.84E-57	0.765680955	4.78E-84
HOXA13	brown	-0.168545665	0.000448096	0.234701698	8.58E-07
MASP1	brown	-0.426217818	2.09E-20	0.617994634	1.19E-46
EPS8L2	brown	0.2306899	1.33E-06	-0.489120408	3.02E-27
PRMT1	brown	0.241569919	3.98E-07	-0.468754167	7.11E-25
COL4A4	brown	-0.260764974	4.09E-08	0.551107002	1.58E-35
S1PR5	brown	0.218197397	4.96E-06	-0.355176803	3.13E-14
TMEM60	brown	0.391238945	3.53E-17	-0.40263391	3.45E-18
TYRP1	brown	-0.017994706	0.709825755	0.372733924	1.28E-15
CCDC86	brown	0.237433666	6.34E-07	-0.382703013	1.90E-16
ARRB1	brown	-0.143338725	0.002891146	0.303824952	1.24E-10
ADRM1	brown	0.297288083	3.18E-10	-0.536474153	2.04E-33
MAS1L	brown	-0.410952946	5.96E-19	0.737454054	5.87E-75
PTRHD1	brown	0.213968865	7.61E-06	-0.462845082	3.24E-24
MITF	brown	-0.334634245	1.04E-12	0.687307826	2.10E-61
PPP1R35	brown	0.3442407	2.09E-13	-0.532476921	7.40E-33
TBC1D10B	brown	0.335572859	8.90E-13	-0.406843029	1.43E-18
C1orf53	brown	0.295956799	3.85E-10	-0.407716674	1.19E-18
ITIH3	brown	-0.098945141	0.040283257	0.357194413	2.19E-14
RORA	brown	-0.253567003	9.81E-08	0.545971457	8.94E-35
PCDHB3	brown	-0.037199636	0.441650548	0.217025458	5.59E-06
CLCN2	brown	0.347048747	1.29E-13	-0.393290631	2.34E-17
ALPK3	brown	-0.164129123	0.000633745	0.427145285	1.69E-20
MPV17L2	brown	0.261342712	3.81E-08	-0.354099619	3.79E-14
SMOC2	brown	-0.393144022	2.41E-17	0.757753023	2.28E-81
AP1S1	brown	0.369819359	2.20E-15	-0.54480656	1.32E-34
PCDH9	brown	-0.241766303	3.89E-07	0.4206414	7.24E-20
UNC119	brown	0.325421663	4.59E-12	-0.422284397	5.03E-20
HDAC8	brown	0.256654508	6.77E-08	-0.320583287	9.83E-12
PCK2	brown	0.307216399	7.51E-11	-0.392132334	2.95E-17

EFNA3	brown	0.231248898	1.25E-06	-0.337443249	6.53E-13
SPOP	brown	-0.274510578	7.15E-09	0.514681917	1.86E-30
MLIP	brown	-0.247961643	1.90E-07	0.342179636	2.96E-13
SLC25A23	brown	-0.329897462	2.24E-12	0.449121018	9.82E-23
FAM189A2	brown	-0.456593856	1.56E-23	0.532261915	7.93E-33
TUFM	brown	0.234766589	8.52E-07	-0.464794603	1.97E-24
CHRM2	brown	-0.554731657	4.56E-36	0.847510922	8.32E-120
LNP1	brown	-0.161397072	0.000781948	0.334703798	1.03E-12
C4orf3	brown	-0.212569203	8.75E-06	0.201423139	2.58E-05
EEPD1	brown	-0.21306458	8.33E-06	0.399570409	6.50E-18
ETV4	brown	0.300379117	2.04E-10	-0.428184185	1.34E-20
UBAC2	brown	0.316254328	1.92E-11	-0.323194303	6.53E-12
PRELP	brown	-0.439977309	8.76E-22	0.790016286	5.58E-93
PVRL1	brown	0.167168542	0.000499704	-0.476462178	9.40E-26
CD34	brown	-0.412863911	3.95E-19	0.706432111	3.11E-66
TMEM189	brown	0.244994811	2.69E-07	-0.442769666	4.52E-22
PRUNE2	brown	-0.444127172	3.27E-22	0.880907938	3.93E-141
CUTC	brown	-0.26249302	3.30E-08	0.301343724	1.78E-10
SCAF1	brown	0.259623541	4.71E-08	-0.435471094	2.51E-21
DTX2	brown	0.221531645	3.52E-06	-0.431260653	6.64E-21
C6orf136	brown	0.312993872	3.16E-11	-0.576744242	1.73E-39
BAIAP2L1	brown	0.294132694	4.98E-10	-0.549975688	2.32E-35
CDK7	brown	0.266933996	1.89E-08	-0.408220715	1.07E-18
ZNF454	brown	-0.189361778	7.78E-05	0.438291964	1.30E-21
DDA1	brown	0.201247183	2.62E-05	-0.442872635	4.41E-22
FREM3	brown	-0.062797414	0.193708124	0.184754018	0.000116579
MBOAT7	brown	0.292053947	6.67E-10	-0.459189502	8.16E-24
SHISA6	brown	-0.387065172	8.09E-17	0.420858587	6.90E-20
CPTP	brown	0.307134025	7.60E-11	-0.55417445	5.52E-36
FOLH1	brown	0.070028292	0.147144765	0.054347109	0.260794012
CXorf36	brown	-0.275339162	6.42E-09	0.635249471	5.70E-50
EDEM2	brown	0.357818691	1.96E-14	-0.372022466	1.46E-15
ENPP6	brown	-0.419682165	8.95E-20	0.387601956	7.28E-17
DNAJC18	brown	-0.339287124	4.80E-13	0.618184756	1.10E-46
ENPP2	brown	-0.219861376	4.18E-06	0.491143208	1.72E-27
PPP1R12C	brown	-0.211577424	9.65E-06	0.300156055	2.11E-10
FAM47E-STB	brown	-0.18590245	0.000105498	0.258430236	5.45E-08
PHYHIP	brown	-0.451664542	5.28E-23	0.590566308	8.96E-42
MAP1B	brown	-0.39419296	1.95E-17	0.820149556	7.68E-106
TMEM79	brown	0.152802227	0.001482777	-0.403939902	2.63E-18
PITX2	brown	-0.269612551	1.35E-08	0.47802955	6.19E-26
PROM2	brown	0.145630861	0.002468064	-0.399124943	7.12E-18
EPHA2	brown	-0.041548506	0.390102553	-0.208026641	1.37E-05
KCNQ5	brown	-0.219753126	4.23E-06	0.360958517	1.12E-14
S100A14	brown	0.17502832	0.000265226	-0.372288539	1.39E-15
ZNF185	brown	0.062700063	0.194401227	-0.231023585	1.28E-06
PCDH19	brown	-0.136704941	0.004513249	0.267743184	1.71E-08
DENND2A	brown	-0.359121743	1.55E-14	0.589916434	1.15E-41
SNRPA1	brown	0.402166803	3.80E-18	-0.590584984	8.89E-42
UCKL1	brown	0.314682	2.44E-11	-0.467828755	9.03E-25
DDX49	brown	0.324464505	5.34E-12	-0.478385316	5.63E-26

GNG5	brown	0.369488283	2.34E-15	-0.50920934	9.56E-30
CHCHD6	brown	0.225553958	2.31E-06	-0.348918288	9.36E-14
NAT9	brown	0.378357685	4.40E-16	-0.465634112	1.59E-24
REM1	brown	-0.20713844	1.49E-05	0.472632924	2.58E-25
CES1	brown	-0.295534851	4.09E-10	0.458468658	9.78E-24
GRIK5	brown	-0.297537304	3.07E-10	0.544455767	1.48E-34
IRX3	brown	0.058910802	0.222804927	-0.196150267	4.21E-05
PGR	brown	-0.572606811	7.94E-39	0.551320453	1.47E-35
HBA1	brown	-0.08992059	0.062464957	0.16453642	0.000614027
FGF7	brown	-0.367480023	3.40E-15	0.771490349	4.45E-86
KCNK3	brown	-0.360252223	1.27E-14	0.619559197	6.09E-47
RGN	brown	-0.38091619	2.69E-16	0.628488837	1.21E-48
DUSP1	brown	-0.417751224	1.37E-19	0.510528929	6.46E-30
TMEM147	brown	0.287255907	1.30E-09	-0.366736029	3.90E-15
ZNF628	brown	0.185306831	0.000111115	-0.367312164	3.51E-15
SLCO2A1	brown	-0.125335819	0.009275746	0.399141814	7.10E-18
NIPSNAP3B	brown	-0.210597029	1.06E-05	0.440419708	7.89E-22
TRPV4	brown	0.107223011	0.026191213	-0.237670909	6.17E-07
TFAP2C	brown	0.207705058	1.41E-05	-0.333944679	1.16E-12
FAM180B	brown	-0.681727948	4.59E-60	0.732412117	1.86E-73
PIDD1	brown	0.26296036	3.12E-08	-0.366074996	4.40E-15
PARM1	brown	-0.295098676	4.35E-10	0.494707038	6.34E-28
ACOT2	brown	-0.07461993	0.122345714	0.326157724	4.09E-12
LAMC3	brown	-0.422804687	4.48E-20	0.470936707	4.03E-25
TP53I3	brown	0.171937451	0.000341365	-0.322320048	7.49E-12
OPCML	brown	-0.1497544	0.001846249	0.253553842	9.83E-08
ACVRL1	brown	-0.220252422	4.01E-06	0.543971098	1.74E-34
BMX	brown	-0.262371535	3.35E-08	0.391642032	3.26E-17
CHTF18	brown	0.389363384	5.13E-17	-0.533237319	5.80E-33
SLC25A22	brown	0.327145128	3.49E-12	-0.453848891	3.09E-23
PPP1R1A	brown	-0.35697796	2.28E-14	0.626930213	2.42E-48
ABCB1	brown	-0.381797453	2.27E-16	0.519076245	4.90E-31
C19orf25	brown	0.295652389	4.02E-10	-0.351091574	6.42E-14
CLPTM1	brown	0.290858867	7.89E-10	-0.411531267	5.26E-19
PKNOX2	brown	-0.258977342	5.10E-08	0.360991425	1.11E-14
KCNK1	brown	0.086262572	0.073953014	-0.264324677	2.63E-08
ILK	brown	-0.363820576	6.65E-15	0.729902651	1.01E-72
KANK2	brown	-0.404672237	2.25E-18	0.807447585	3.84E-100
IGSF3	brown	0.184400905	0.000120201	-0.333837412	1.18E-12
SLC43A1	brown	-0.201327448	2.60E-05	0.453054903	3.75E-23
WDR24	brown	0.208834233	1.26E-05	-0.288365165	1.11E-09
RCAN1	brown	-0.358701407	1.68E-14	0.604872867	2.94E-44
MSRB3	brown	-0.390827376	3.83E-17	0.844083319	6.53E-118
SLC8B1	brown	0.238605149	5.56E-07	-0.266334572	2.04E-08
TMEM102	brown	0.263888115	2.78E-08	-0.560231011	6.73E-37
GNB5	brown	-0.218609241	4.75E-06	0.259142356	5.00E-08
NPM3	brown	0.234148836	9.12E-07	-0.375535371	7.53E-16
ELMO3	brown	0.216324846	6.00E-06	-0.532352778	7.70E-33
ARHGAP39	brown	0.394322753	1.90E-17	-0.423111599	4.19E-20
TMEM35	brown	-0.372992479	1.22E-15	0.583827847	1.20E-40
SNPH	brown	-0.132461787	0.005942775	0.328933326	2.62E-12

COA3	brown	0.20810585	1.36E-05	-0.483581502	1.38E-26
KLHL17	brown	0.277981418	4.53E-09	-0.395000083	1.65E-17
ZNF34	brown	-0.124956731	0.009492476	0.242191596	3.71E-07
DGUOK	brown	0.326986757	3.58E-12	-0.531553408	9.94E-33
GLRB	brown	-0.071637047	0.138053344	0.299895726	2.19E-10
ADRA1D	brown	-0.443444225	3.85E-22	0.504845475	3.45E-29
GPR56	brown	0.23212717	1.14E-06	-0.406936132	1.40E-18
SVIL	brown	-0.331116268	1.84E-12	0.656411814	2.41E-54
SMARCD3	brown	-0.213289889	8.14E-06	0.382158878	2.11E-16
GPD1	brown	-0.095964757	0.046726925	0.320828399	9.46E-12
KDR	brown	-0.120127637	0.012673959	0.451973099	4.89E-23
EFNA1	brown	0.266464575	2.01E-08	-0.321057442	9.13E-12
INHBB	brown	-0.214910109	6.92E-06	0.37890959	3.96E-16
LSM4	brown	0.399985571	5.97E-18	-0.615127706	4.07E-46
UBALD2	brown	0.360309108	1.26E-14	-0.571198667	1.33E-38
CKMT2	brown	-0.389199391	5.30E-17	0.700659565	9.79E-65
MRPL14	brown	0.315092712	2.29E-11	-0.531467255	1.02E-32
CCL16	brown	-0.148298226	0.002047244	0.34624012	1.48E-13
THSD7A	brown	-0.154920086	0.001270253	0.478572377	5.35E-26
CFAP58	brown	-0.081117977	0.092962905	0.295905393	3.88E-10
RAMP1	brown	-0.293993867	5.08E-10	0.58377211	1.23E-40
PSD	brown	-0.467167614	1.07E-24	0.802758793	3.81E-98
KCND3	brown	-0.469744404	5.50E-25	0.608574744	6.39E-45
NOV	brown	-0.085125457	0.077856232	0.326225828	4.04E-12
FILIP1	brown	-0.542401914	2.93E-34	0.900557889	6.19E-157
PYGL	brown	0.121425729	0.011737523	-0.232381368	1.11E-06
LIMK2	brown	0.225933142	2.22E-06	-0.24031918	4.58E-07
DNAJC7	brown	0.259651845	4.69E-08	-0.434652094	3.04E-21
RGCC	brown	-0.247608928	1.98E-07	0.4009538	4.89E-18
C11orf95	brown	-0.187385877	9.26E-05	0.504430984	3.89E-29
KLHL40	brown	-0.370089979	2.10E-15	0.259776708	4.62E-08
TERF2IP	brown	-0.091483711	0.058026054	0.172390873	0.000329045
PNMA2	brown	-0.244025764	3.00E-07	0.511848585	4.36E-30
PLAC9	brown	-0.492935673	1.04E-27	0.609153509	5.02E-45
THOC6	brown	0.268730481	1.51E-08	-0.406061445	1.68E-18
KIF13A	brown	-0.227708985	1.83E-06	0.372918801	1.23E-15
SDC1	brown	0.236903328	6.72E-07	-0.532660596	6.98E-33
RAET1G	brown	0.025136291	0.603201513	-0.29060865	8.16E-10
FAM110A	brown	0.395417319	1.52E-17	-0.634878787	6.75E-50
RADIL	brown	-0.20863983	1.29E-05	0.379233892	3.72E-16
RNF113A	brown	0.204731776	1.88E-05	-0.396368753	1.25E-17
CTTNBP2	brown	-0.162922871	0.000695639	0.309109601	5.66E-11
ZEB1	brown	-0.392208505	2.91E-17	0.886340767	3.31E-145
EGFLAM	brown	-0.007750868	0.872676293	0.328884503	2.64E-12
CAMK2A	brown	-0.521986264	2.00E-31	0.781843158	7.50E-90
GRIK3	brown	-0.310256592	4.77E-11	0.381825687	2.25E-16
TMX2-CTNND	brown	-0.128839034	0.007471951	0.268406757	1.57E-08
CTAGE9	brown	0.098117308	0.041992906	-0.208896013	1.26E-05
STX10	brown	0.306720865	8.08E-11	-0.479858403	3.79E-26
CNKSR3	brown	-0.309089633	5.68E-11	0.375634176	7.39E-16
COL4A3	brown	-0.114248604	0.017789137	0.298437377	2.70E-10

CNN1	brown	-0.426697307	1.87E-20	0.754401564	2.88E-80
LMX1A	brown	-0.344666103	1.94E-13	0.326320289	3.98E-12
ZNF788	brown	-0.079947163	0.097793739	0.341483911	3.32E-13
PRPH	brown	-0.203567352	2.10E-05	0.387363618	7.63E-17
C3orf18	brown	-0.277075821	5.11E-09	0.565791458	9.37E-38
SYNE1	brown	-0.487667097	4.52E-27	0.827573066	2.20E-109
PTGS1	brown	-0.415332212	2.32E-19	0.721556569	2.44E-70
NUDT19	brown	0.237309111	6.43E-07	-0.286357764	1.47E-09
PDGFD	brown	-0.316142936	1.95E-11	0.540000374	6.47E-34
FOXD3	brown	-0.321683293	8.28E-12	0.485710122	7.74E-27
C19orf33	brown	0.189453945	7.72E-05	-0.461477791	4.58E-24
RALY	brown	0.320999914	9.21E-12	-0.448924423	1.03E-22
SLC7A2	brown	-0.116072037	0.016037579	0.284457854	1.90E-09
CACNB4	brown	-0.314420795	2.54E-11	0.548456836	3.88E-35
GRASP	brown	-0.355241683	3.10E-14	0.561865012	3.78E-37
TUSC5	brown	-0.189341674	7.79E-05	0.410957401	5.95E-19
ADAMTS1	brown	-0.481280122	2.58E-26	0.654520291	6.12E-54
SNTB1	brown	-0.078611162	0.103548062	0.376572415	6.19E-16
CGNL1	brown	-0.49765155	2.75E-28	0.757255572	3.33E-81
CALCRL	brown	-0.076010222	0.115517273	0.539500409	7.62E-34
NFIC	brown	-0.302891346	1.42E-10	0.557947286	1.50E-36
KIF26A	brown	-0.234209706	9.06E-07	0.432163629	5.40E-21
TIGD5	brown	0.241156073	4.17E-07	-0.360639503	1.18E-14
SPINT2	brown	0.236352934	7.15E-07	-0.422544999	4.75E-20
TMEM9B	brown	0.121065894	0.011990775	-0.256847975	6.61E-08
STYXL1	brown	0.309678595	5.20E-11	-0.462798951	3.28E-24
GREM2	brown	-0.500303123	1.29E-28	0.584007718	1.12E-40
RNF122	brown	-0.353997872	3.86E-14	0.535289238	3.00E-33
GPR124	brown	-0.354591671	3.48E-14	0.807398703	4.03E-100
PFKFB4	brown	0.328343502	2.88E-12	-0.353119822	4.50E-14
ZNF385D	brown	-0.214399742	7.28E-06	0.530943961	1.21E-32
PTGER3	brown	-0.265453486	2.28E-08	0.689720759	5.42E-62
KCNB1	brown	-0.570136662	1.96E-38	0.827073597	3.86E-109
HPD	brown	-0.283784118	2.08E-09	0.534192809	4.27E-33
C8orf4	brown	-0.250933729	1.34E-07	0.219772803	4.22E-06
EMCN	brown	-0.370666706	1.88E-15	0.667026127	1.13E-56
TIGD1	brown	0.216273825	6.03E-06	-0.266995353	1.88E-08
THEM5	brown	0.107855966	0.025314684	-0.292286448	6.46E-10
FP325317.1	brown	-0.259159224	4.99E-08	0.536104592	2.30E-33
TNNI3K	brown	-0.219672242	4.26E-06	0.295442022	4.14E-10
AKAP13	brown	-0.286905626	1.36E-09	0.646319721	3.24E-52
LPIN1	brown	-0.157217523	0.001071655	0.441213485	6.54E-22
ABCA9	brown	-0.285280814	1.70E-09	0.4402486	8.22E-22
NRG2	brown	-0.293299526	5.60E-10	0.28950129	9.52E-10
TSHZ2	brown	-0.2447004	2.78E-07	0.396152882	1.31E-17
RBM10	brown	0.186983376	9.60E-05	-0.317780509	1.52E-11
HDAC5	brown	-0.249844889	1.53E-07	0.330015345	2.20E-12
CRTC3	brown	-0.251972006	1.19E-07	0.60903765	5.27E-45
ASMTL	brown	-0.129739036	0.007062366	0.242338896	3.64E-07
PDZRN4	brown	-0.538377461	1.10E-33	0.820372874	6.04E-106
ANKRD53	brown	-0.297453847	3.11E-10	0.506859086	1.91E-29

USP43	brown	0.225137034	2.41E-06	-0.324176534	5.59E-12
DDX54	brown	0.27677693	5.31E-09	-0.412974877	3.86E-19
EIF5A	brown	0.248492156	1.79E-07	-0.482171457	2.03E-26
STARD3	brown	0.266654891	1.96E-08	-0.34452083	1.99E-13
USP20	brown	-0.165719496	0.000559931	0.250803387	1.36E-07
GALE	brown	0.30638027	8.50E-11	-0.587336406	3.13E-41
AP4S1	brown	-0.122582519	0.010955112	0.330746954	1.96E-12
DCT	brown	-0.286078463	1.52E-09	0.465565161	1.62E-24
MPP2	brown	-0.183639277	0.000128374	0.336592748	7.52E-13
HDC	brown	-0.352208915	5.28E-14	0.673577225	3.68E-58
HSD17B6	brown	-0.384684634	1.29E-16	0.715143167	1.45E-68
PTPRD	brown	-0.275061254	6.65E-09	0.537111805	1.66E-33
ST8SIA1	brown	-0.324246784	5.53E-12	0.526378782	5.11E-32
PECAM1	brown	-0.222778071	3.09E-06	0.609833021	3.78E-45
KLF17	brown	-0.404236731	2.47E-18	0.75972526	5.03E-82
MYLK	brown	-0.444823922	2.77E-22	0.867395423	8.31E-132
SRRT	brown	0.268342811	1.58E-08	-0.498432132	2.20E-28
CRLF2	brown	-0.316624965	1.81E-11	0.399612172	6.45E-18
CNNM1	brown	-0.285206375	1.72E-09	0.381210246	2.54E-16
KANK3	brown	-0.239894598	4.81E-07	0.453202416	3.62E-23
PLCL1	brown	-0.427561528	1.54E-20	0.787528036	5.17E-92
ATP6AP1	brown	0.303224223	1.35E-10	-0.395988052	1.35E-17
ARHGEF9	brown	-0.315896646	2.03E-11	0.496279833	4.06E-28
AQP2	brown	-0.138006462	0.004141686	0.218746475	4.69E-06
PLA2G5	brown	-0.346499824	1.42E-13	0.775447464	1.70E-87
DNAJB4	brown	-0.365008695	5.35E-15	0.608116895	7.72E-45
ZNF671	brown	-0.186157527	0.000103175	0.411396352	5.42E-19
KDELR1	brown	0.378614852	4.19E-16	-0.427963817	1.41E-20
PAMR1	brown	-0.493186141	9.72E-28	0.633694828	1.16E-49
PRDM16	brown	-0.130198316	0.006861191	0.194722635	4.80E-05
TARS2	brown	0.298234688	2.78E-10	-0.399369769	6.78E-18
C4A	brown	-0.199590653	3.06E-05	0.63156327	3.04E-49
KCND1	brown	-0.036849485	0.445963452	0.245282009	2.60E-07
GEMIN7	brown	0.351544782	5.93E-14	-0.556583155	2.40E-36
STEAP3	brown	0.244153054	2.96E-07	-0.390310798	4.25E-17
EMID1	brown	-0.069341547	0.151161856	0.128302256	0.007726221
PLVAP	brown	-0.07662808	0.112580149	0.35110518	6.40E-14
ABCA8	brown	-0.550445285	1.98E-35	0.661627149	1.77E-55
HGF	brown	-0.211926133	9.33E-06	0.593121945	3.29E-42
PGM5	brown	-0.535286061	3.00E-33	0.826188559	1.04E-108
ALG14	brown	0.158507502	0.000973109	-0.23611549	7.34E-07
GEM	brown	-0.416285459	1.88E-19	0.795336263	4.31E-95
ROBO4	brown	-0.150807067	0.001712383	0.459958135	6.73E-24
KCNN2	brown	-0.272328289	9.49E-09	0.492886226	1.06E-27
SMPDL3B	brown	0.183728783	0.000127387	-0.24256303	3.55E-07
ZNF516	brown	-0.266121493	2.10E-08	0.393101457	2.43E-17
ERC1	brown	-0.242657556	3.51E-07	0.52785691	3.21E-32
PIGU	brown	0.316431379	1.87E-11	-0.443726497	3.60E-22
ACTG2	brown	-0.397140948	1.07E-17	0.715229352	1.37E-68
RAB38	brown	0.074772236	0.121582668	-0.232439143	1.10E-06
PLEKHJ1	brown	0.316220431	1.93E-11	-0.440305717	8.11E-22

TXLNB	brown	-0.322335719	7.47E-12	0.625233405	5.12E-48
GSTM5	brown	-0.375383971	7.75E-16	0.390480323	4.11E-17
LRRC4B	brown	-0.351599821	5.87E-14	0.471180288	3.78E-25
TCEAL3	brown	-0.235545983	7.82E-07	0.405041766	2.08E-18
DMD	brown	-0.464817659	1.96E-24	0.78503005	4.69E-91
PDE1B	brown	-0.099402307	0.039364586	0.315842892	2.05E-11
LRRC3B	brown	-0.601844772	1.01E-43	0.409025764	8.99E-19
NPR1	brown	-0.344297024	2.07E-13	0.622844475	1.46E-47
TMEM47	brown	-0.165868099	0.000553458	0.297483977	3.10E-10
CAPN1	brown	0.200696778	2.76E-05	-0.495223195	5.48E-28
RBPMS2	brown	-0.402323663	3.68E-18	0.779004758	8.50E-89
SLC9A9	brown	-0.380053046	3.18E-16	0.610712239	2.62E-45
ADAT3	brown	0.220018101	4.11E-06	-0.366883367	3.80E-15
HPGDS	brown	-0.388266673	6.38E-17	0.663251469	7.79E-56
SLC25A12	brown	-0.233379225	9.93E-07	0.340383688	4.00E-13
MRPS22	brown	0.173252126	0.000306779	-0.356763227	2.37E-14
LEP	brown	-0.075339717	0.118772231	0.208522884	1.30E-05
RD3L	brown	-0.499626606	1.56E-28	0.262572199	3.27E-08
PREX2	brown	-0.350949443	6.58E-14	0.608152228	7.61E-45
CMA1	brown	-0.407425397	1.26E-18	0.773883454	6.22E-87
FBLN1	brown	-0.176750226	0.000230014	0.204838994	1.86E-05
UGP2	brown	-0.188423992	8.45E-05	0.313398926	2.97E-11
HN1	brown	0.450576138	6.89E-23	-0.603795704	4.57E-44
DUSP3	brown	-0.274568	7.10E-09	0.545908521	9.13E-35
TPRKB	brown	0.263824423	2.80E-08	-0.429025553	1.11E-20
GNAL	brown	-0.484670757	1.03E-26	0.706376671	3.21E-66
STAT5B	brown	-0.328407674	2.85E-12	0.595239774	1.43E-42
PCDH17	brown	8.36E-05	0.998621513	0.360324721	1.25E-14
DLC1	brown	-0.29879729	2.57E-10	0.568304989	3.80E-38
CLEC3A	brown	-0.431678139	6.03E-21	0.623969948	8.92E-48
CILP	brown	-0.341030745	3.59E-13	0.709015904	6.46E-67
PDZD11	brown	0.215546392	6.49E-06	-0.374475079	9.21E-16
THAP8	brown	-0.03921233	0.417325894	0.235911863	7.51E-07
KPTN	brown	0.296392087	3.62E-10	-0.426989647	1.75E-20
PACSIN3	brown	0.17192251	0.000341778	-0.322807503	6.94E-12
LURAP1	brown	-0.3036341	1.27E-10	0.426875114	1.80E-20
IMP4	brown	0.248779592	1.73E-07	-0.389238068	5.26E-17
C14orf28	brown	-0.262921773	3.13E-08	0.468186442	8.24E-25
PELI2	brown	-0.405944393	1.72E-18	0.610484708	2.88E-45
AKAP12	brown	-0.357835213	1.96E-14	0.599989354	2.14E-43
AXIN2	brown	-0.137860323	0.004181981	0.38428301	1.40E-16
GSN	brown	-0.424877358	2.82E-20	0.619046592	7.60E-47
CPXM2	brown	-0.319526508	1.16E-11	0.765006396	8.15E-84
TMX4	brown	-0.289518887	9.50E-10	0.509397512	9.04E-30
BCO2	brown	-0.273689758	7.96E-09	0.235127753	8.19E-07
RAB9B	brown	-0.464484801	2.13E-24	0.764546029	1.17E-83
TRPC4	brown	-0.295048072	4.38E-10	0.699095045	2.46E-64
AOX1	brown	-0.518385857	6.05E-31	0.822802883	4.35E-107
FAM57A	brown	0.081193487	0.092658019	-0.225708176	2.27E-06
AVPR2	brown	-0.289570037	9.43E-10	0.460907345	5.30E-24
CASQ2	brown	-0.460974605	5.21E-24	0.818273134	5.69E-105

2-Mar	brown	-0.162935249	0.000694977	0.381865912	2.24E-16
ESYT2	brown	-0.181985924	0.000147948	0.419544314	9.23E-20
DHRS1	brown	0.051793713	0.283895089	-0.3245554	5.27E-12
CFAP221	brown	0.059187903	0.220632552	0.166450404	0.000528757
CASP14	brown	0.149763263	0.001845083	-0.234403863	8.87E-07
EBF1	brown	-0.438721011	1.18E-21	0.846205132	4.44E-119
ADAMTSL2	brown	-0.051041902	0.29095226	0.160039553	0.000866958
CALM1	brown	-0.164071231	0.000636595	0.283212524	2.25E-09
TECR	brown	0.24651768	2.25E-07	-0.550826839	1.74E-35
MROH6	brown	0.211206267	1.00E-05	-0.255245997	8.02E-08
ACAN	brown	0.107430854	0.025900549	0.194410664	4.94E-05
OXER1	brown	-0.348094548	1.08E-13	0.380173475	3.10E-16
PCSK7	brown	-0.396221503	1.29E-17	0.767348561	1.27E-84
ACTN3	brown	-0.176605366	0.000232799	0.30878305	5.95E-11
FHL1	brown	-0.470029594	5.10E-25	0.854596803	7.08E-124
PEX14	brown	-0.229629689	1.49E-06	0.123279936	0.010506044
CHRDL1	brown	-0.454882402	2.39E-23	0.728781197	2.13E-72
ENDOG	brown	0.121115267	0.011955743	-0.36848919	2.82E-15
FAT4	brown	-0.400575289	5.28E-18	0.841164202	2.47E-116
WWC1	brown	0.21781391	5.15E-06	-0.269243848	1.41E-08
GPM6B	brown	-0.336106212	8.15E-13	0.552635548	9.37E-36
WDR17	brown	-0.205210399	1.80E-05	0.332363834	1.50E-12
TRAF5	brown	-0.120840644	0.012151751	0.362337316	8.71E-15
CDIP1	brown	-0.255099696	8.16E-08	0.348288729	1.04E-13
ENPP3	brown	-0.262449902	3.32E-08	0.485061188	9.25E-27
RHOV	brown	0.183817756	0.000126413	-0.409287475	8.50E-19
C11orf80	brown	0.265827323	2.18E-08	-0.309482409	5.36E-11
TACC2	brown	-0.384091758	1.45E-16	0.418668612	1.12E-19
IL13	brown	-0.299771816	2.23E-10	0.360204052	1.28E-14
KRT18	brown	0.331288376	1.79E-12	-0.48242581	1.90E-26
TACR1	brown	-0.406596635	1.50E-18	0.611302088	2.05E-45
HABP4	brown	-0.300446771	2.02E-10	0.604334799	3.67E-44
TMEM258	brown	0.335050147	9.69E-13	-0.481608932	2.36E-26
PDE4D	brown	-0.426767928	1.84E-20	0.60523322	2.54E-44
RNF180	brown	-0.485687792	7.79E-27	0.735632591	2.06E-74
DOK6	brown	-0.460657721	5.64E-24	0.754781624	2.17E-80
PRSS8	brown	0.274354423	7.30E-09	-0.310932018	4.31E-11
MEGF8	brown	-0.131827923	0.00618814	0.291055981	7.67E-10
CSRNP1	brown	-0.490086352	2.31E-27	0.550036555	2.27E-35
MRPL27	brown	0.294037678	5.05E-10	-0.519989767	3.71E-31
DUS3L	brown	0.165467573	0.000571065	-0.327844578	3.12E-12
SRL	brown	-0.538118854	1.20E-33	0.780909201	1.67E-89
LEPR	brown	-0.423526312	3.82E-20	0.596258864	9.53E-43
CPQ	brown	-0.278167427	4.42E-09	0.542682275	2.67E-34
GPIHBP1	brown	-0.482402376	1.91E-26	0.687419349	1.97E-61
AGMAT	brown	0.231927037	1.16E-06	-0.347847922	1.13E-13
UTRN	brown	-0.282787492	2.38E-09	0.603751548	4.65E-44
KIAA1377	brown	-0.270664408	1.18E-08	0.420921132	6.81E-20
PLA2G2A	brown	-0.200906844	2.71E-05	0.399019717	7.28E-18
LRFN5	brown	-0.47586836	1.10E-25	0.718282455	1.99E-69
KCNMA1	brown	-0.498116369	2.41E-28	0.84495863	2.17E-118

OAS1	brown	0.2010952	2.66E-05	-0.375692917	7.31E-16
KCP	brown	0.153660115	0.001393035	-0.204403349	1.94E-05
C7	brown	-0.482237177	1.99E-26	0.76298798	3.99E-83
EBF2	brown	-0.404614981	2.28E-18	0.760984644	1.90E-82
TMEM233	brown	-0.224549353	2.56E-06	0.404605872	2.28E-18
TMEM229A	brown	-0.294149487	4.97E-10	0.532565845	7.19E-33
ZMAT3	brown	-0.13339043	0.005599066	0.434172993	3.40E-21
SSBP2	brown	-0.317302571	1.63E-11	0.496903474	3.40E-28
LDB3	brown	-0.450213532	7.53E-23	0.658491104	8.57E-55
ITGB4	brown	0.16695298	0.000508265	-0.396641945	1.18E-17
UBE2J2	brown	0.27769044	4.71E-09	-0.472795879	2.48E-25
NR4A3	brown	-0.540725442	5.10E-34	0.61346379	8.24E-46
LAMA2	brown	-0.418542871	1.15E-19	0.798340315	2.60E-96
CMTM5	brown	-0.693333227	6.94E-63	0.719385558	9.85E-70
PTGFR	brown	-0.492896365	1.05E-27	0.838526309	6.20E-115
MYOT	brown	-0.489654738	2.61E-27	0.604131179	3.98E-44
TMED9	brown	0.28892852	1.03E-09	-0.296798912	3.41E-10
GPRASP1	brown	-0.50667391	2.02E-29	0.819158803	2.22E-105
CEP85L	brown	-0.410793529	6.16E-19	0.593676533	2.65E-42
HOXB7	brown	0.283493113	2.17E-09	-0.441311521	6.39E-22
SLC8A1	brown	-0.388730252	5.82E-17	0.737679478	5.02E-75
PAK3	brown	-0.058247513	0.228066734	0.200104395	2.92E-05
ANG	brown	-0.211142558	1.01E-05	0.278746577	4.09E-09
PHYHD1	brown	-0.266276527	2.06E-08	0.394946894	1.67E-17
PARD3B	brown	-0.413774491	3.25E-19	0.729013156	1.83E-72
TUB	brown	-0.33648516	7.65E-13	0.61191893	1.58E-45
HSF1	brown	0.206280431	1.62E-05	-0.345141774	1.79E-13
NKIRAS1	brown	-0.132098068	0.006082478	0.342902952	2.62E-13
BOLA3	brown	0.327309292	3.40E-12	-0.459700214	7.18E-24
PDXK	brown	0.321511218	8.51E-12	-0.330601791	2.00E-12
AAAS	brown	0.294282866	4.88E-10	-0.369019766	2.56E-15
TMEM256-PL	brown	0.162749147	0.000705001	-0.288449231	1.10E-09
PIP5K1C	brown	-0.277139417	5.06E-09	0.490694786	1.95E-27
SELE	brown	-0.295161562	4.31E-10	0.458950303	8.67E-24
DCXR	brown	0.238378551	5.70E-07	-0.446030446	2.07E-22
FAM107A	brown	-0.626159552	3.40E-48	0.718595761	1.63E-69
EBPL	brown	0.13362516	0.005515067	-0.276215465	5.72E-09
SLC40A1	brown	-0.131881958	0.006166875	0.352275659	5.22E-14
IL33	brown	-0.280230925	3.36E-09	0.471409723	3.56E-25
ANKRD16	brown	0.198395538	3.42E-05	-0.279823822	3.55E-09
MAOB	brown	-0.40694448	1.40E-18	0.680874918	7.31E-60
MKX	brown	-0.374980414	8.37E-16	0.729989075	9.52E-73
BAG2	brown	-0.369572182	2.31E-15	0.698454002	3.58E-64
PISD	brown	0.202914934	2.24E-05	-0.250720674	1.38E-07
TP53I11	brown	-0.049498703	0.305803635	0.168679476	0.000443356
ZCCHC24	brown	-0.408199599	1.07E-18	0.812136555	3.40E-102
BCL2	brown	-0.33420244	1.11E-12	0.536593352	1.97E-33
ZBTB47	brown	-0.326590303	3.81E-12	0.593164456	3.24E-42
RUNX1T1	brown	-0.402213159	3.76E-18	0.831308491	3.12E-111
CACNA1G	brown	-0.002116107	0.965101408	0.157870816	0.001020652
CPE	brown	-0.205534144	1.74E-05	0.376781665	5.94E-16

TTLL7	brown	-0.343127268	2.52E-13	0.555284095	3.77E-36
TCEAL2	brown	-0.459664061	7.24E-24	0.730690406	5.95E-73
FAM155A	brown	-0.220254723	4.01E-06	0.333295826	1.29E-12
CAPN15	brown	0.254138	9.17E-08	-0.43710398	1.72E-21
BRAT1	brown	0.262805129	3.18E-08	-0.397323897	1.03E-17
ZFYVE27	brown	0.198152256	3.50E-05	-0.296251131	3.69E-10
RAC1	brown	0.172933573	0.000314846	-0.357478383	2.08E-14
ESAM	brown	-0.095167067	0.048591345	0.379068472	3.84E-16
OR2C1	brown	-0.10128629	0.035763548	0.226962192	1.99E-06
FHL5	brown	-0.382631404	1.93E-16	0.692116089	1.39E-62
TMEM220	brown	-0.457061203	1.39E-23	0.672049968	8.23E-58
SNRK	brown	-0.30901532	5.75E-11	0.575523984	2.71E-39
INPP5A	brown	-0.318566349	1.34E-11	0.459932922	6.77E-24
TBC1D1	brown	-0.16027047	0.00085192	0.339297358	4.80E-13
HSPB3	brown	-0.19819473	3.49E-05	0.367209156	3.57E-15
LDB2	brown	-0.34475035	1.91E-13	0.704393035	1.06E-65
ZDHHC24	brown	0.361793299	9.61E-15	-0.425515268	2.44E-20
ESR1	brown	-0.310968781	4.29E-11	0.233318757	9.99E-07
MTG2	brown	0.331738154	1.67E-12	-0.383727999	1.56E-16
THEGL	brown	-0.152149418	0.00155459	0.356876569	2.32E-14
DMGDH	brown	-0.600549255	1.71E-43	0.807998044	2.22E-100
NCALD	brown	-0.3808374	2.73E-16	0.666996443	1.14E-56
KLF12	brown	-0.197503144	3.72E-05	0.504847042	3.45E-29
RXRG	brown	-0.506607055	2.06E-29	0.569946706	2.10E-38
TARBP2	brown	0.414942313	2.52E-19	-0.573906365	4.93E-39
SSBP1	brown	0.252429914	1.12E-07	-0.48167996	2.32E-26
FUCA2	brown	0.254772251	8.49E-08	-0.265061323	2.40E-08
FAM110D	brown	-0.356638121	2.42E-14	0.523317617	1.33E-31
TOMM34	brown	0.283910595	2.05E-09	-0.340222233	4.11E-13
MPRIP	brown	-0.129828412	0.007022808	0.253192819	1.03E-07
RAD51L3-RF	brown	-0.116055992	0.016052308	0.186306541	0.00010184
WWTR1	brown	-0.351575162	5.90E-14	0.732241361	2.09E-73
HHIP	brown	-0.341340841	3.41E-13	0.294120446	4.99E-10
PTP4A3	brown	-0.077191582	0.109952872	0.394203782	1.94E-17
STARD13	brown	-0.371686174	1.56E-15	0.770771113	7.99E-86
PSMA4	brown	0.292917447	5.91E-10	-0.482120979	2.06E-26
MYOCD	brown	-0.597403658	6.04E-43	0.840448848	5.96E-116
SLIT2	brown	-0.448169608	1.24E-22	0.819507674	1.53E-105
CHCHD1	brown	0.329875542	2.25E-12	-0.509989055	7.59E-30
THOC3	brown	0.271576091	1.05E-08	-0.481449413	2.47E-26
SV2B	brown	-0.064452693	0.182198475	0.304635365	1.10E-10
MEOX1	brown	-0.379260788	3.70E-16	0.607495457	9.99E-45
WNT9A	brown	-0.247239175	2.07E-07	0.262374469	3.35E-08
THRSP	brown	-0.077516274	0.108461094	0.244529476	2.84E-07
C11orf98	brown	0.275728058	6.10E-09	-0.493867894	8.03E-28
CCDC3	brown	-0.267415294	1.78E-08	0.518963126	5.07E-31
NPRL3	brown	0.248085387	1.88E-07	-0.35016007	7.55E-14
TSPAN18	brown	-0.3696386	2.28E-15	0.753812788	4.48E-80
RAI2	brown	-0.458094239	1.07E-23	0.706051894	3.91E-66
KIAA2013	brown	0.248143078	1.86E-07	-0.360114661	1.30E-14
ANKRD34A	brown	-0.05560495	0.249903928	0.304847161	1.07E-10

ZNF135	brown	-0.155559236	0.00121185	0.285148364	1.73E-09
SLC39A11	brown	0.329424832	2.42E-12	-0.363560929	6.97E-15
TIAM2	brown	-0.200520468	2.81E-05	0.478948663	4.84E-26
FLT1	brown	-0.041983733	0.385152413	0.260356395	4.30E-08
SHROOM3	brown	-0.142760237	0.003007867	0.288792939	1.05E-09
AARD	brown	-0.454204878	2.83E-23	0.750681027	4.59E-79
VARS2	brown	0.244401146	2.88E-07	-0.333709109	1.21E-12
DPCD	brown	0.230670132	1.33E-06	-0.372097797	1.44E-15
CCDC176	brown	-0.085291219	0.077277047	0.379687418	3.41E-16
TMEM179B	brown	0.279590659	3.66E-09	-0.406836227	1.43E-18
CDC42EP3	brown	-0.252152257	1.16E-07	0.534090193	4.41E-33
SGCD	brown	-0.414921613	2.53E-19	0.821291771	2.24E-106
CBX4	brown	0.246208437	2.34E-07	-0.290330449	8.49E-10
NT5C3A	brown	0.247071506	2.11E-07	-0.370271954	2.03E-15
RBMX2	brown	0.123818267	0.01017065	-0.284584708	1.87E-09
BLOC1S3	brown	0.384952956	1.23E-16	-0.380861564	2.72E-16
HSPA12B	brown	-0.241776568	3.89E-07	0.671722346	9.78E-58
CST11	brown	-0.065916725	0.172446828	0.194069906	5.10E-05
NKAPL	brown	-0.569311095	2.64E-38	0.803703897	1.52E-98
PTPN21	brown	-0.219496431	4.34E-06	0.498412361	2.21E-28
HIF3A	brown	-0.42612373	2.13E-20	0.620158755	4.70E-47
SBDS	brown	-0.332302068	1.52E-12	0.432316189	5.21E-21
C6orf223	brown	0.123746091	0.010215059	-0.178348581	0.000201287
ARMCX2	brown	-0.079571855	0.099383905	0.230929606	1.30E-06
FAM150B	brown	-0.347744688	1.15E-13	0.31055269	4.56E-11
F2RL1	brown	0.025810359	0.59351729	-0.193647918	5.30E-05
CCDC69	brown	-0.433619813	3.86E-21	0.631674275	2.89E-49
MAPK13	brown	0.3343335	1.09E-12	-0.570157246	1.94E-38
TRADD	brown	0.229463593	1.52E-06	-0.32394126	5.81E-12
PRDM6	brown	-0.49185568	1.41E-27	0.7906239	3.23E-93
RYR2	brown	-0.370793911	1.84E-15	0.726331735	1.08E-71
ZNF835	brown	-0.20456933	1.91E-05	0.421009374	6.68E-20
DGKB	brown	-0.390366502	4.20E-17	0.663463479	6.99E-56
PTGES3L	brown	-0.4160107	2.00E-19	0.599926386	2.19E-43
CSTB	brown	0.179854645	0.000177324	-0.517762764	7.32E-31
PGPEP1L	brown	-0.187246471	9.38E-05	0.34056575	3.88E-13
PDK4	brown	-0.478431429	5.56E-26	0.670871527	1.53E-57
SEPT4	brown	-0.056959753	0.238532983	0.474500352	1.58E-25
PRKCSH	brown	0.308794963	5.94E-11	-0.45623738	1.71E-23
RGS11	brown	-0.158724452	0.000957381	0.332921087	1.37E-12
THSD7B	brown	-0.166509443	0.000526311	0.245436215	2.55E-07
SPON1	brown	-0.350841838	6.70E-14	0.732185153	2.17E-73
LRCH1	brown	-0.286189966	1.50E-09	0.64700491	2.34E-52
LY6D	brown	0.065203832	0.17714555	-0.28463072	1.86E-09
ERO1L	brown	0.24865667	1.76E-07	-0.368402992	2.87E-15
TIMM23	brown	0.259729076	4.65E-08	-0.425493091	2.46E-20
TMEM150C	brown	-0.204100481	2.00E-05	0.469623339	5.67E-25
DYNLT1	brown	0.137834064	0.004189259	-0.286754556	1.39E-09
DCAF15	brown	0.339977251	4.28E-13	-0.518789005	5.35E-31
PRICKLE2	brown	-0.421259487	6.32E-20	0.734505405	4.47E-74
PQLC2	brown	0.431960762	5.66E-21	-0.427701727	1.49E-20

SPEG	brown	-0.3836784	1.57E-16	0.735479654	2.29E-74
ISL2	brown	0.249379582	1.61E-07	-0.292962551	5.88E-10
ACKR3	brown	-0.185330427	0.000110887	0.295628506	4.03E-10
ADIPOQ	brown	-0.166501085	0.000526656	0.41853657	1.15E-19
ULBP2	brown	0.280343811	3.31E-09	-0.309420768	5.41E-11
RBFOX3	brown	-0.58113935	3.33E-40	0.822740314	4.65E-107
UNC5C	brown	-0.333492121	1.25E-12	0.623361442	1.17E-47
MTTP	brown	-0.316249241	1.92E-11	0.578809598	7.99E-40
YTHDF1	brown	0.280086897	3.43E-09	-0.35277572	4.78E-14
CYFIP2	brown	-0.15981855	0.00088158	0.295488711	4.11E-10
HAAO	brown	-0.442188992	5.19E-22	0.560902484	5.31E-37
SRP19	brown	0.170886532	0.00037159	-0.283531769	2.16E-09
MAGI2	brown	-0.402751477	3.36E-18	0.582369516	2.09E-40
TSPYL2	brown	-0.36559085	4.81E-15	0.46476241	1.99E-24
FAM127B	brown	0.211063607	1.02E-05	-0.361041904	1.10E-14
MDK	brown	0.242170031	3.72E-07	-0.306849623	7.93E-11
LSR	brown	0.321117766	9.05E-12	-0.535383615	2.91E-33
CDK5	brown	0.363589178	6.94E-15	-0.425370248	2.53E-20
HSBP1	brown	0.228254084	1.73E-06	-0.374882299	8.53E-16
LDOC1	brown	-0.122491396	0.011015024	0.195763738	4.37E-05
ZNF25	brown	-0.312619316	3.34E-11	0.69292103	8.79E-63
BTBD6	brown	0.178248452	0.000202983	-0.303304486	1.34E-10
DDX28	brown	0.155157329	0.001248279	-0.391220756	3.54E-17
TXNDC17	brown	0.185354323	0.000110657	-0.55575229	3.21E-36
ACE	brown	-0.003646068	0.939907092	0.226070346	2.18E-06
FDXR	brown	0.222967662	3.03E-06	-0.463036268	3.09E-24
CPSF1	brown	0.296575729	3.53E-10	-0.357799074	1.97E-14
KRTCAP2	brown	0.308880492	5.86E-11	-0.455957846	1.83E-23
TSLP	brown	-0.332069925	1.58E-12	0.329923784	2.23E-12
WDR18	brown	0.31089225	4.34E-11	-0.501169582	1.00E-28
SERTM1	brown	-0.375865743	7.08E-16	0.550770549	1.77E-35
ZNRD1	brown	0.20651419	1.59E-05	-0.486780223	5.77E-27
FREM1	brown	-0.250167353	1.47E-07	0.34456832	1.97E-13
NRXN2	brown	-0.281162196	2.97E-09	0.635117818	6.05E-50
CLUAP1	brown	-0.214868253	6.95E-06	0.36838186	2.88E-15
TMEM255B	brown	-0.091216161	0.058766754	0.432825342	4.64E-21
CSRNP3	brown	-0.306523103	8.32E-11	0.51367753	2.52E-30
TMEM11	brown	0.236372314	7.13E-07	-0.376649645	6.10E-16
C16orf45	brown	-0.349854155	7.96E-14	0.573795776	5.13E-39
AGT	brown	-0.120660247	0.012282047	0.357029065	2.26E-14
CLEC3B	brown	-0.648797612	9.90E-53	0.725666738	1.68E-71
TTC23	brown	-0.180679007	0.000165367	0.36632001	4.21E-15
C11orf31	brown	0.281379068	2.88E-09	-0.456846596	1.47E-23
ERGIC3	brown	0.263706777	2.84E-08	-0.414159806	2.99E-19
RBCK1	brown	0.283015202	2.31E-09	-0.400450126	5.42E-18
STARD9	brown	-0.426962675	1.76E-20	0.796825862	1.08E-95
MCAM	brown	-0.274422664	7.23E-09	0.683886289	1.40E-60
TCEAL7	brown	-0.457288096	1.31E-23	0.784782811	5.83E-91
ANKS1B	brown	-0.403128688	3.11E-18	0.639857554	6.80E-51
ADAMTS4	brown	-0.27527186	6.47E-09	0.572179827	9.28E-39
RBMS3	brown	-0.393205931	2.38E-17	0.768513818	4.97E-85

C7orf43	brown	0.305741797	9.34E-11	-0.420055758	8.24E-20
NOVA1	brown	-0.41369419	3.31E-19	0.602850543	6.72E-44
CNTNAP1	brown	-0.228271807	1.73E-06	0.702769983	2.80E-65
C6orf132	brown	0.097151785	0.044063819	-0.34689002	1.33E-13
EGR1	brown	-0.415557181	2.21E-19	0.556337336	2.62E-36
ZC3H3	brown	0.329543245	2.38E-12	-0.423212576	4.09E-20
FAXC	brown	-0.2631475	3.05E-08	0.512114775	4.03E-30
STON1	brown	-0.405403004	1.93E-18	0.727993364	3.61E-72
LYVE1	brown	-0.389264966	5.23E-17	0.729172175	1.64E-72
BHMT2	brown	-0.5980988	4.57E-43	0.897116968	6.12E-154
C16orf89	brown	-0.675615454	1.25E-58	0.73812846	3.68E-75
NLGN1	brown	-0.418409892	1.18E-19	0.594569702	1.86E-42
GPR146	brown	-0.343535722	2.35E-13	0.637878932	1.70E-50
TUBB4B	brown	0.109463924	0.023200884	-0.438886937	1.13E-21
ROR1	brown	-0.392959032	2.50E-17	0.740280673	8.17E-76
GJA4	brown	-0.148659056	0.001995656	0.472237971	2.87E-25
SPRY1	brown	-0.382621141	1.93E-16	0.644898307	6.37E-52
CTSO	brown	-0.168346059	0.000455256	0.428032395	1.39E-20
CMC1	brown	0.302592815	1.48E-10	-0.360678404	1.17E-14
CPNE1	brown	0.259433392	4.82E-08	-0.340421405	3.97E-13
SPRY2	brown	-0.2315843	1.21E-06	0.324900723	4.99E-12
RPP38	brown	0.250977083	1.34E-07	-0.432343736	5.18E-21
HBM	brown	0.00103587	0.982912428	0.175534075	0.000254394
TIMP4	brown	-0.151292672	0.00165368	0.311192262	4.15E-11
LMOD3	brown	-0.614382284	5.58E-46	0.750151544	6.78E-79
GNG7	brown	-0.516659302	1.02E-30	0.684099533	1.25E-60
RDH5	brown	-0.209480789	1.19E-05	0.529079916	2.18E-32
WISP2	brown	-0.286367787	1.47E-09	0.615498136	3.47E-46
METTL1	brown	0.323068752	6.66E-12	-0.454121059	2.88E-23
TMEM252	brown	-0.584194461	1.04E-40	0.801748066	1.01E-97
DAZAP1	brown	0.394161724	1.96E-17	-0.597550307	5.69E-43
HOPX	brown	-0.040282623	0.404717848	0.197100765	3.86E-05
RNF149	brown	0.2420375	3.77E-07	-0.400111624	5.81E-18
PTPN3	brown	0.204477457	1.93E-05	-0.302608323	1.48E-10
LPPR2	brown	-0.044612081	0.356078861	0.203244914	2.17E-05
GPR87	brown	0.13762304	0.004248164	-0.286307796	1.48E-09
TLCD1	brown	0.303339178	1.33E-10	-0.495114999	5.65E-28
E2F4	brown	0.220496715	3.91E-06	-0.295725674	3.98E-10
pk	brown	-0.40599625	1.71E-18	0.732169651	2.19E-73
NTRK2	brown	-0.150934306	0.00169682	0.326017478	4.18E-12
TGFBR2	brown	-0.282342342	2.53E-09	0.605936448	1.90E-44
TMEM240	brown	-0.214264404	7.38E-06	0.388389309	6.23E-17
NR3C2	brown	-0.489975245	2.39E-27	0.680242817	1.03E-59
PLD2	brown	0.098725295	0.040731447	-0.23594794	7.48E-07
C2CD4B	brown	-0.359269545	1.51E-14	0.435435772	2.54E-21
ZNF728	brown	-0.300668548	1.96E-10	0.422942985	4.35E-20
P4HA1	brown	0.195481702	4.48E-05	-0.199712598	3.03E-05
PRKAG2	brown	-0.366145227	4.35E-15	0.529642325	1.83E-32
SLC39A4	brown	0.365218601	5.15E-15	-0.551540481	1.36E-35
SH2D3A	brown	0.183622115	0.000128564	-0.511908636	4.28E-30
TMEM81	brown	0.216457486	5.92E-06	-0.20756327	1.43E-05

FBXL7	brown	-0.432264427	5.27E-21	0.847314527	1.07E-119
KRT222	brown	-0.210500724	1.07E-05	0.350970551	6.56E-14
CCDC81	brown	-0.212711814	8.62E-06	0.387175279	7.92E-17
SYNM	brown	-0.49769608	2.71E-28	0.859918227	4.45E-127
S100A11	brown	0.297165772	3.24E-10	-0.565355576	1.09E-37
PINK1	brown	-0.14281787	0.002996049	0.287293076	1.29E-09
KIAA0408	brown	-0.502336148	7.15E-29	0.636939411	2.62E-50
IL1RL1	brown	-0.120327469	0.012525641	0.27927597	3.82E-09
PER2	brown	-0.467683599	9.38E-25	0.415887051	2.05E-19
CGB7	brown	0.124313481	0.009870543	-0.221249359	3.62E-06
TCEAL1	brown	-0.308592198	6.12E-11	0.399565801	6.51E-18
ANK2	brown	-0.487195014	5.15E-27	0.79115283	2.00E-93
NANOS1	brown	-0.08503256	0.078182357	0.290586219	8.19E-10
PACSIN2	brown	-0.110621632	0.021775158	0.3249613	4.94E-12
PCDH18	brown	-0.325018151	4.90E-12	0.767336772	1.28E-84
CHRM3	brown	-0.371325236	1.66E-15	0.6172349	1.66E-46
HTR2A	brown	-0.309671214	5.21E-11	0.697700743	5.56E-64
SLC6A16	brown	-0.107454414	0.025867777	0.253542455	9.84E-08
MECP2	brown	-0.198710515	3.32E-05	0.362375346	8.65E-15
C6	brown	-0.166796802	0.000514553	0.42613222	2.13E-20
ZCWPW2	brown	-0.417539193	1.43E-19	0.605154701	2.62E-44
SCRG1	brown	-0.293666572	5.32E-10	0.771541887	4.26E-86
GNB2	brown	0.233153293	1.02E-06	-0.380094051	3.15E-16
CABS1	brown	-0.39759673	9.75E-18	0.345975146	1.55E-13
RPP25L	brown	0.128768525	0.007504919	-0.404639547	2.27E-18
ADAMTS17	brown	-0.050753221	0.293693053	0.269515545	1.36E-08
TGIF1	brown	0.136778429	0.004491493	-0.250234737	1.46E-07
DDX41	brown	0.318351982	1.39E-11	-0.428488711	1.25E-20
TSPAN2	brown	-0.319698072	1.13E-11	0.611869844	1.61E-45
UBE2L3	brown	0.220684196	3.84E-06	-0.341126447	3.53E-13
AKR1A1	brown	0.270301309	1.23E-08	-0.378762702	4.07E-16
C22orf39	brown	-0.214787418	7.01E-06	0.307005431	7.75E-11
TP53INP2	brown	-0.404606744	2.28E-18	0.512045646	4.11E-30
NGFR	brown	-0.19759026	3.69E-05	0.469958816	5.20E-25
NOTCH4	brown	-0.062404952	0.196513404	0.382737717	1.89E-16
PDZRN3	brown	-0.425075995	2.70E-20	0.801867636	9.00E-98
SVEP1	brown	-0.542661732	2.69E-34	0.768965572	3.45E-85
CC2D2A	brown	-0.347019457	1.30E-13	0.584889387	8.01E-41
SHROOM4	brown	-0.107232964	0.02617723	0.326523636	3.85E-12
PCDHGA12	brown	-0.240061622	4.72E-07	0.682208767	3.53E-60
PLEKHH2	brown	-0.199255194	3.16E-05	0.312363903	3.47E-11
ADARB1	brown	-0.309871733	5.05E-11	0.694804792	2.98E-63
SCN4B	brown	-0.399210023	7.00E-18	0.525032812	7.78E-32
VSX1	brown	-0.208327232	1.33E-05	0.367782423	3.22E-15
PALMD	brown	-0.107269107	0.026126508	0.163397933	0.000670624
ATP5H	brown	0.184155749	0.000122777	-0.41124974	5.59E-19
TBX5	brown	-0.41181919	4.95E-19	0.683661097	1.59E-60
PKIB	brown	0.135124359	0.005004791	-0.158306178	0.000987917
NEGR1	brown	-0.584348474	9.85E-41	0.877551874	1.03E-138
NUDT14	brown	0.219643469	4.27E-06	-0.377370105	5.31E-16
FIGF	brown	-0.521373301	2.42E-31	0.653200577	1.17E-53

PRKAA2	brown	-0.361626074	9.90E-15	0.495804267	4.65E-28
CDC42BPA	brown	-0.290793479	7.96E-10	0.571470576	1.20E-38
NNAT	brown	-0.168294056	0.000457139	0.219167547	4.49E-06
DIXDC1	brown	-0.475428874	1.24E-25	0.905930734	7.75E-162
CERS4	brown	-0.161187308	0.000794559	0.234243509	9.03E-07
COL4A6	brown	-0.27584848	6.00E-09	0.284767632	1.82E-09
SFN	brown	0.23029397	1.39E-06	-0.475682508	1.16E-25
APOLD1	brown	-0.426441812	1.98E-20	0.659843197	4.35E-55
KIAA0513	brown	-0.33829324	5.67E-13	0.467612169	9.55E-25
DSEL	brown	-0.224757048	2.51E-06	0.512361041	3.74E-30
MICU3	brown	-0.55187074	1.22E-35	0.840465209	5.84E-116
HRASLS5	brown	-0.201578356	2.54E-05	0.410456986	6.62E-19
KDF1	brown	0.306924222	7.84E-11	-0.603655243	4.84E-44
PKHD1L1	brown	-0.190602865	6.97E-05	0.319165449	1.23E-11
MIEN1	brown	0.259117277	5.01E-08	-0.458515396	9.67E-24
MRPL4	brown	0.205153693	1.81E-05	-0.425848923	2.27E-20
DPP9	brown	0.24097305	4.26E-07	-0.25662118	6.79E-08
PRKG1	brown	-0.420849393	6.92E-20	0.873042183	1.42E-135
SHKBP1	brown	0.291779805	6.93E-10	-0.366176783	4.32E-15
BID	brown	0.354902711	3.29E-14	-0.446072662	2.05E-22
SGCA	brown	-0.457845526	1.14E-23	0.793889452	1.64E-94
TIMM17B	brown	0.253685865	9.68E-08	-0.495379678	5.24E-28
ZER1	brown	-0.186987793	9.59E-05	0.206346886	1.61E-05
SOX17	brown	-0.5011429	1.01E-28	0.647924397	1.51E-52
CAP2	brown	-0.310586848	4.54E-11	0.587315823	3.16E-41
CD300LG	brown	-0.474492646	1.58E-25	0.654127315	7.43E-54
SLC22A17	brown	-0.192676614	5.78E-05	0.322255871	7.57E-12
GPRASP2	brown	-0.311786576	3.79E-11	0.518145174	6.52E-31
MED18	brown	0.211492321	9.74E-06	-0.375476006	7.62E-16
RAD9A	brown	0.298042415	2.86E-10	-0.493046938	1.01E-27
ARHGEF17	brown	-0.236267445	7.22E-07	0.683962816	1.35E-60
NDUFB4	brown	0.214108444	7.50E-06	-0.396320012	1.26E-17
PPME1	brown	0.195256945	4.57E-05	-0.289096988	1.01E-09
VPS16	brown	0.267007191	1.88E-08	-0.276466095	5.53E-09
A2M	brown	-0.356852543	2.33E-14	0.734480029	4.55E-74
GNL2	brown	0.242021983	3.78E-07	-0.394355097	1.89E-17
RHBDD3	brown	0.321546725	8.46E-12	-0.365439607	4.95E-15
HSPG2	brown	-0.210581697	1.07E-05	0.69892577	2.72E-64
ARHGAP24	brown	-0.334664788	1.03E-12	0.762686978	5.05E-83
RBFA	brown	0.12097491	0.012055569	-0.223387193	2.90E-06
HBA2	brown	-0.21899284	4.57E-06	0.257927098	5.80E-08
LPAR3	brown	0.082734868	0.086607437	-0.231008725	1.29E-06
TNFRSF25	brown	0.277050886	5.12E-09	-0.429151662	1.07E-20
FSCN1	brown	0.199276478	3.15E-05	-0.297014305	3.31E-10
KIAA1644	brown	-0.514944842	1.72E-30	0.866362501	3.89E-131
FOSB	brown	-0.420091892	8.18E-20	0.341571032	3.28E-13
TSTD1	brown	0.251604552	1.24E-07	-0.464761193	1.99E-24
ADPRHL2	brown	0.333303708	1.29E-12	-0.484374967	1.12E-26
KL	brown	-0.089445488	0.063868325	0.296352994	3.64E-10
ZNF331	brown	-0.182489608	0.000141707	0.21735523	5.40E-06
ITGA9	brown	-0.364914273	5.45E-15	0.711502591	1.40E-67

GIPC1	brown	0.264921769	2.44E-08	-0.572240496	9.08E-39
SLC25A30	brown	-0.107281547	0.026109069	0.40548125	1.90E-18
DUSP23	brown	0.224853574	2.48E-06	-0.285271324	1.70E-09
SPINK13	brown	-0.149466821	0.001884451	0.205007908	1.83E-05
SUV420H2	brown	0.268863835	1.48E-08	-0.351826656	5.65E-14
ALDH7A1	brown	-0.13005824	0.006921997	0.184835949	0.000115754
PRAC1	brown	-0.356502503	2.48E-14	0.26394583	2.76E-08
LTBR	brown	0.272543344	9.23E-09	-0.466846494	1.16E-24
BDNF	brown	-0.287591809	1.24E-09	0.415446335	2.26E-19
RAMP3	brown	-0.157612461	0.001040548	0.437412404	1.60E-21
RUVBL2	brown	0.334189716	1.12E-12	-0.547910817	4.66E-35
TMEM138	brown	0.311490903	3.96E-11	-0.34462103	1.96E-13
CALCOCO1	brown	-0.284578381	1.87E-09	0.467751623	9.22E-25
KCNN3	brown	-0.324514198	5.30E-12	0.590569062	8.95E-42
TRIM3	brown	-0.123460595	0.010392412	0.404810495	2.19E-18
HLX	brown	-0.067711585	0.161028995	0.412904248	3.92E-19
ACTR5	brown	0.300504242	2.01E-10	-0.391760836	3.18E-17
WLS	brown	-0.235442836	7.91E-07	0.405860113	1.76E-18
POMGNT1	brown	0.24265296	3.52E-07	-0.361312873	1.05E-14
NLE1	brown	0.22026642	4.01E-06	-0.364288051	6.11E-15
AGTR1	brown	-0.472318029	2.81E-25	0.734259954	5.29E-74
LONRF2	brown	-0.372244552	1.40E-15	0.582445295	2.03E-40
RGS5	brown	-0.340221589	4.11E-13	0.660425653	3.25E-55
AFF3	brown	-0.546208394	8.26E-35	0.752651122	1.07E-79
PKD2	brown	-0.233789434	9.49E-07	0.600129508	2.02E-43
WBSCR17	brown	-0.476573047	9.12E-26	0.794384979	1.04E-94
POR	brown	0.210998576	1.02E-05	-0.357257618	2.17E-14
ANGPTL1	brown	-0.398101711	8.79E-18	0.815250787	1.37E-103
TMEM177	brown	0.307428774	7.28E-11	-0.390484685	4.10E-17
APEH	brown	0.196920783	3.92E-05	-0.334540877	1.05E-12
MEIS2	brown	-0.329130945	2.54E-12	0.565474566	1.05E-37
ENOX1	brown	-0.179389241	0.000184428	0.513723738	2.49E-30
ARHGAP10	brown	-0.305422159	9.79E-11	0.414627416	2.70E-19
FAXDC2	brown	-0.496453399	3.86E-28	0.769191723	2.88E-85
PMF1	brown	0.239263335	5.16E-07	-0.39611907	1.32E-17
MYH3	brown	-0.284947079	1.78E-09	0.568845083	3.12E-38
JTB	brown	0.343197564	2.49E-13	-0.415293738	2.34E-19
CHURC1	brown	-0.233308323	1.00E-06	0.445028103	2.64E-22
ENG	brown	-0.021059558	0.663215927	0.309203183	5.59E-11
RNASE4	brown	-0.431496233	6.29E-21	0.539273795	8.21E-34
TSPAN7	brown	-0.294738768	4.57E-10	0.430986931	7.07E-21
GYG1	brown	-0.203858403	2.05E-05	0.394556376	1.81E-17
GTF2E2	brown	0.163829897	0.000648604	-0.240498965	4.49E-07
FZR1	brown	0.188266785	8.57E-05	-0.34666323	1.38E-13
ARHGAP20	brown	-0.539417418	7.83E-34	0.87245608	3.57E-135
C3orf70	brown	-0.360261778	1.27E-14	0.645886645	3.99E-52
SYNGR2	brown	0.335073441	9.66E-13	-0.415980203	2.01E-19
ZSCAN18	brown	-0.167302507	0.000494451	0.253960209	9.36E-08
MYOZ1	brown	-0.03951696	0.413714082	0.243229537	3.29E-07
DIRAS1	brown	-0.239213028	5.19E-07	0.470149331	4.95E-25
SPRY4	brown	0.079459874	0.099862324	0.112982442	0.019101592

NHP2	brown	0.143040285	0.002950837	-0.366308028	4.22E-15
PDLIM3	brown	-0.376668883	6.07E-16	0.793111367	3.35E-94
DSTN	brown	-0.414510798	2.77E-19	0.59167271	5.81E-42
PPP1R16A	brown	0.310571133	4.55E-11	-0.53666114	1.92E-33
DACT2	brown	-0.11969478	0.013000553	0.185811561	0.000106337
WSCD1	brown	-0.097607569	0.04307576	0.352904532	4.68E-14
SLMAP	brown	-0.421249279	6.33E-20	0.718130087	2.19E-69
HIST3H2A	brown	0.351626736	5.85E-14	-0.40627818	1.61E-18
ZBTB4	brown	-0.35519698	3.12E-14	0.504487626	3.83E-29
ZNF423	brown	-0.180194367	0.000172302	0.367450828	3.42E-15
TUT1	brown	0.249740427	1.55E-07	-0.424803714	2.87E-20
CNTN1	brown	-0.371635697	1.57E-15	0.664949227	3.28E-56
SEMA6B	brown	-0.135024878	0.005037285	0.411509438	5.29E-19
CPT1A	brown	-0.12646012	0.008658617	0.220125543	4.07E-06
MRPL21	brown	0.168578791	0.000446918	-0.406308906	1.60E-18
UQCC2	brown	0.262903351	3.14E-08	-0.445383394	2.42E-22
MST1R	brown	0.108936238	0.023877084	-0.291133703	7.59E-10
C20orf194	brown	-0.274062801	7.58E-09	0.38364932	1.58E-16
NPTX1	brown	-0.212931323	8.44E-06	0.311937562	3.70E-11
SOX18	brown	-0.105561481	0.028617479	0.295552225	4.08E-10
APLNR	brown	-0.010339401	0.830715496	0.421955052	5.41E-20
COX19	brown	0.296882983	3.37E-10	-0.396261905	1.28E-17
VLDLR	brown	-0.169860527	0.000403488	0.361284584	1.05E-14
ASL	brown	0.217676745	5.23E-06	-0.29810541	2.83E-10
RANGAP1	brown	0.298534694	2.66E-10	-0.536050523	2.34E-33
LIFR	brown	-0.330441767	2.05E-12	0.570856241	1.51E-38
ERG	brown	-0.220373552	3.96E-06	0.60223759	8.62E-44
NACC2	brown	-0.414488455	2.78E-19	0.732590716	1.65E-73
IL17RD	brown	-0.206548075	1.58E-05	0.452489368	4.31E-23
PARVA	brown	-0.310083371	4.90E-11	0.673233159	4.41E-58
KLHL13	brown	-0.353522109	4.20E-14	0.494970397	5.88E-28
HSPA12A	brown	-0.342416661	2.84E-13	0.662463004	1.16E-55
GNA14	brown	-0.21347985	7.99E-06	0.391512985	3.34E-17
C1QTNF3	brown	-0.158007698	0.001010254	0.437531184	1.56E-21
PPP3CB	brown	-0.322131324	7.72E-12	0.555186507	3.90E-36
FYCO1	brown	-0.3639972	6.44E-15	0.628189808	1.38E-48
MCAT	brown	0.238575448	5.58E-07	-0.451206084	5.91E-23
TRIM63	brown	-0.285326433	1.69E-09	0.51470613	1.85E-30
PODN	brown	-0.324672313	5.17E-12	0.69146746	2.01E-62
RUNDC3B	brown	-0.418062579	1.28E-19	0.546043204	8.73E-35
SSTR1	brown	-0.322678497	7.08E-12	0.392628989	2.67E-17
GNAZ	brown	-0.336435783	7.71E-13	0.60428396	3.74E-44
PPP1R13L	brown	0.261118359	3.92E-08	-0.530632114	1.33E-32
SCN7A	brown	-0.564220553	1.64E-37	0.666521528	1.46E-56
AMOTL1	brown	-0.262529686	3.29E-08	0.52439409	9.49E-32
CADM3	brown	-0.473242586	2.20E-25	0.653374802	1.07E-53
CCDC22	brown	0.111095391	0.021214004	-0.302257864	1.56E-10
RNASEK	brown	0.191720658	6.30E-05	-0.285194152	1.72E-09
FZD4	brown	-0.118271684	0.014127165	0.385312385	1.14E-16
MRPL2	brown	0.272869604	8.85E-09	-0.448883971	1.04E-22
PKD1	brown	-0.355807813	2.80E-14	0.610782225	2.54E-45

PALLD	brown	-0.363502966	7.05E-15	0.744021613	5.77E-77
PSMG4	brown	0.244717974	2.77E-07	-0.342230148	2.93E-13
TMEM55A	brown	-0.244441367	2.86E-07	0.568109617	4.07E-38
KCNK6	brown	-0.149231001	0.001916315	0.182361107	0.000143275
PUSL1	brown	0.294956794	4.44E-10	-0.51984015	3.88E-31
PRKCA	brown	-0.15380994	0.001377884	0.331434174	1.75E-12
C1orf21	brown	-0.299639688	2.27E-10	0.300733479	1.94E-10
SPECC1L	brown	-0.077047019	0.110622236	0.350498813	7.12E-14
ATP6V0A1	brown	-0.036710827	0.447677919	0.217176642	5.50E-06
HMGCLL1	brown	-0.426537096	1.94E-20	0.385061142	1.20E-16
LRRN4CL	brown	-0.487754612	4.41E-27	0.72291322	1.01E-70
ZNF366	brown	-0.142236897	0.003117134	0.564373701	1.55E-37
VWA1	brown	-0.01998157	0.679473427	0.181988022	0.000147922
FAM110B	brown	-0.310925462	4.32E-11	0.662416479	1.19E-55
RP11-216L1	brown	-0.169183981	0.000425897	0.259534322	4.76E-08
VCL	brown	-0.305218198	1.01E-10	0.615162602	4.01E-46
TPSD1	brown	-0.35546885	2.98E-14	0.613030265	9.89E-46
ACKR1	brown	-0.375878335	7.06E-16	0.616828603	1.97E-46
DTWD1	brown	-0.202067497	2.43E-05	0.423422465	3.91E-20
MFAP4	brown	-0.391923028	3.08E-17	0.688219463	1.26E-61
TSPAN17	brown	0.275004161	6.70E-09	-0.335644054	8.79E-13
SMARCA4	brown	0.281299513	2.91E-09	-0.350838924	6.71E-14
MRPS34	brown	0.327839668	3.12E-12	-0.577921243	1.11E-39
EFHD2	brown	0.204878669	1.86E-05	-0.482948156	1.64E-26
PEBP4	brown	-0.170616038	0.000379764	0.331153875	1.83E-12
CYP7A1	brown	-0.098093926	0.04204207	0.223362954	2.90E-06
HLF	brown	-0.700995479	8.03E-65	0.869508252	3.39E-133
TIMM13	brown	0.287161331	1.31E-09	-0.507551275	1.56E-29
TPRA1	brown	0.321685786	8.28E-12	-0.43380988	3.69E-21
MEF2D	brown	-0.417193742	1.55E-19	0.653032226	1.27E-53
ERCC2	brown	0.247296004	2.06E-07	-0.301182045	1.82E-10
CCDC102B	brown	-0.075467618	0.118145868	0.443005876	4.27E-22
GJB6	brown	0.161651625	0.000766892	-0.332201736	1.54E-12
REEP2	brown	-0.258974968	5.10E-08	0.56936138	2.59E-38
VDAC1	brown	0.305861785	9.17E-11	-0.409509945	8.11E-19
CAND2	brown	-0.186576557	9.95E-05	0.431504532	6.28E-21
EMC4	brown	0.20046713	2.82E-05	-0.385382647	1.13E-16
ADRB3	brown	-0.5073951	1.63E-29	0.819294119	1.92E-105
RP11-903H1	brown	-0.451001793	6.21E-23	0.617271402	1.63E-46
RELL2	brown	0.280793749	3.12E-09	-0.325818469	4.31E-12
PTBP2	brown	-0.240108071	4.69E-07	0.464334537	2.22E-24
ALDH16A1	brown	0.257976746	5.76E-08	-0.389578801	4.92E-17
MYCT1	brown	-0.332389234	1.50E-12	0.684696866	8.97E-61
WFS1	brown	-0.163342347	0.000673507	0.207080717	1.50E-05
SHPK	brown	0.216692341	5.78E-06	-0.309124358	5.65E-11
KRT19	brown	0.200831783	2.73E-05	-0.470790085	4.19E-25
H1FNT	brown	-0.078125921	0.105703259	0.212164467	9.11E-06
MIF	brown	0.277000763	5.16E-09	-0.553131531	7.90E-36
MEIS1	brown	-0.380589501	2.86E-16	0.530728175	1.29E-32
RHBDD2	brown	0.190730921	6.89E-05	-0.29606162	3.79E-10
SYNJ2BP-CO	brown	-0.195738353	4.38E-05	0.258623689	5.32E-08

GPR4	brown	-0.023328111	0.629522141	0.314403808	2.55E-11
TMEM238	brown	0.255597645	7.69E-08	-0.439289181	1.03E-21
UNC93B1	brown	0.233035246	1.03E-06	-0.439856087	9.01E-22
NIT2	brown	0.339490235	4.64E-13	-0.503741013	4.76E-29
OLFM1	brown	-0.407086529	1.36E-18	0.616357466	2.41E-46
GLIPR1L2	brown	-0.32661435	3.80E-12	0.314275237	2.60E-11
SLC51B	brown	-0.401975027	3.95E-18	0.688067236	1.37E-61
NCAM1	brown	-0.478087475	6.09E-26	0.801313926	1.53E-97
MRPL32	brown	0.265396271	2.30E-08	-0.461531729	4.52E-24
LTBP4	brown	-0.438889757	1.13E-21	0.50341118	5.24E-29
DALRD3	brown	0.169371514	0.000419572	-0.224207558	2.66E-06
RP5-877J2.	brown	-0.265397422	2.30E-08	0.330774215	1.95E-12
ELANE	brown	-0.598273022	4.26E-43	0.553945177	5.98E-36
MPDU1	brown	0.2258626	2.23E-06	-0.357145506	2.21E-14
RPP21	brown	0.252717888	1.09E-07	-0.525688783	6.34E-32
TXN	brown	0.163182673	0.000681854	-0.380281308	3.04E-16
SMN1	brown	0.140557032	0.003492675	-0.329107936	2.55E-12
STXBP2	brown	0.366379012	4.16E-15	-0.645139973	5.68E-52
WISP3	brown	0.101998507	0.034477134	-0.186188733	0.000102894
NRTN	brown	-0.132494637	0.0059303	0.297865364	2.93E-10
NR4A1	brown	-0.501620515	8.80E-29	0.559727994	8.03E-37
GJB3	brown	0.124642457	0.009675551	-0.364466591	5.91E-15
P4HB	brown	0.38393487	1.50E-16	-0.370583642	1.91E-15
NDRG1	brown	0.060267414	0.212313968	-0.214979237	6.87E-06
TBCC	brown	0.174840442	0.000269359	-0.273917273	7.73E-09
OSTF1	brown	0.110754889	0.021616033	-0.278369738	4.30E-09
ADAM33	brown	-0.505012919	3.28E-29	0.804682138	5.87E-99
PLEKHG5	brown	0.167806334	0.000475152	-0.271703515	1.03E-08
ANKRD39	brown	0.21726278	5.45E-06	-0.354105692	3.79E-14
FGD5	brown	-0.129365088	0.007230043	0.293565892	5.40E-10
TMEM204	brown	-0.097254801	0.043838845	0.331881373	1.63E-12
MEF2C	brown	-0.365839131	4.60E-15	0.799024962	1.36E-96
RP9	brown	0.306038745	8.94E-11	-0.327370291	3.37E-12
AP000350.1	brown	0.120995075	0.012041182	-0.214608121	7.13E-06
SLC45A1	brown	-0.212677729	8.65E-06	0.437470528	1.58E-21
CKB	brown	-0.305795339	9.26E-11	0.33848528	5.49E-13
UCHL3	brown	0.229215798	1.56E-06	-0.479466522	4.21E-26
THBS3	brown	-0.051880024	0.283092348	0.236394774	7.12E-07
FGFBP2	brown	-0.131304093	0.00639773	0.213693742	7.82E-06
EXOC3L2	brown	0.011213748	0.81664392	0.141115242	0.003363562
MMP27	brown	-0.594315389	2.06E-42	0.538552104	1.04E-33
FKBPL	brown	0.35006181	7.68E-14	-0.485164522	8.99E-27
HTR1B	brown	-0.335944868	8.37E-13	0.590538926	9.05E-42
WDR49	brown	-0.192026729	6.13E-05	0.336902962	7.14E-13
ITGA8	brown	-0.574378619	4.14E-39	0.719969496	6.78E-70
PIP5K1B	brown	-0.155613612	0.001206997	0.359983796	1.33E-14
RHOB	brown	-0.404250266	2.46E-18	0.501306672	9.63E-29
TMC3	brown	-0.008671295	0.857708613	0.169739746	0.000407407
EIF4E3	brown	-0.283735185	2.10E-09	0.413772146	3.25E-19
EPHX1	brown	-0.186194129	0.000102845	0.21979821	4.21E-06
C1orf95	brown	-0.424259936	3.24E-20	0.736738686	9.63E-75

ZFP36	brown	-0.410945356	5.97E-19	0.405579994	1.86E-18
TTC28	brown	-0.236770343	6.83E-07	0.513024757	3.07E-30
MARC2	brown	-0.149797411	0.001840597	0.300210704	2.09E-10
TMEM223	brown	0.377253164	5.43E-16	-0.554150246	5.57E-36
MAP3K12	brown	-0.08035065	0.096106851	0.376157832	6.69E-16
HYAL1	brown	-0.18660173	9.92E-05	0.259335901	4.88E-08
AIF1L	brown	-0.142111031	0.003143946	0.210525574	1.07E-05
METTL24	brown	-0.519030678	4.97E-31	0.825235845	2.99E-108
TRAK2	brown	-0.261686374	3.65E-08	0.608993082	5.37E-45
NECAB1	brown	-0.434220225	3.36E-21	0.715702083	1.02E-68
CYP46A1	brown	-0.219077005	4.53E-06	0.505279879	3.04E-29
MARCKS	brown	0.177923811	0.000208575	-0.202312049	2.37E-05
SMYD5	brown	0.304091257	1.19E-10	-0.431425431	6.39E-21
TMC6	brown	0.377578976	5.11E-16	-0.450113904	7.71E-23
SLCO1C1	brown	-0.12628053	0.008754671	0.491985705	1.36E-27
MRPL16	brown	0.214548755	7.18E-06	-0.43149467	6.29E-21
CSGALNACT1	brown	-0.439795829	9.14E-22	0.697155343	7.64E-64
RGMA	brown	-0.21503071	6.84E-06	0.386973784	8.24E-17
TSEN54	brown	0.352416952	5.09E-14	-0.553273331	7.53E-36
DHH	brown	-0.228924847	1.61E-06	0.612270397	1.36E-45
PNPLA7	brown	-0.312978604	3.16E-11	0.402299794	3.70E-18
MYL3	brown	-0.438743871	1.17E-21	0.582888034	1.72E-40
TGFBR3	brown	-0.342734877	2.69E-13	0.443113474	4.17E-22
SS18L2	brown	0.287062062	1.33E-09	-0.447164041	1.58E-22
SNX9	brown	-0.273371154	8.29E-09	0.587543284	2.89E-41
ST3GAL3	brown	-0.26513951	2.37E-08	0.511268885	5.18E-30
RCCD1	brown	0.308184385	6.50E-11	-0.426455328	1.98E-20
EPHA6	brown	-0.3522653	5.23E-14	0.498170432	2.37E-28
RNF115	brown	-0.153258737	0.001434383	0.473278359	2.18E-25
SOCS2	brown	-0.193648123	5.29E-05	0.269623376	1.34E-08
ZDHHC12	brown	0.345886701	1.58E-13	-0.546456107	7.60E-35
LA16c-306E	brown	-0.211732995	9.51E-06	0.250848951	1.36E-07
DLL4	brown	0.00559898	0.907838097	0.286583179	1.42E-09
SMG9	brown	0.34009965	4.19E-13	-0.315614946	2.12E-11
NAP1L3	brown	-0.31240793	3.45E-11	0.711037101	1.87E-67
DAAM2	brown	-0.328549522	2.79E-12	0.763794205	2.12E-83
TRPC1	brown	-0.26302548	3.09E-08	0.555327887	3.71E-36
PHKG1	brown	-0.22082442	3.78E-06	0.620176299	4.66E-47
LSM8	brown	0.3140078	2.71E-11	-0.350012379	7.74E-14
DDIT4L	brown	-0.087656027	0.06938883	0.423100362	4.20E-20
ABCC9	brown	-0.370405655	1.98E-15	0.814567856	2.78E-103
NCAM2	brown	-0.089168756	0.064697633	0.305754474	9.32E-11
RASL12	brown	-0.431474787	6.32E-21	0.851982264	2.38E-122
IL1RL2	brown	0.140723221	0.003453774	-0.221981111	3.35E-06
PCP4	brown	-0.378340727	4.41E-16	0.727964506	3.68E-72
CDKL1	brown	-0.45769959	1.19E-23	0.507753338	1.47E-29
YDJC	brown	0.408136033	1.09E-18	-0.65052626	4.30E-53
GALNT14	brown	0.173981991	0.000289019	-0.329692899	2.32E-12
SPATA4	brown	-0.348205384	1.06E-13	0.513821545	2.41E-30
NTRK3	brown	-0.527611046	3.47E-32	0.495444503	5.14E-28
MRPS12	brown	0.31707367	1.69E-11	-0.525033943	7.78E-32

PENK	brown	-0.197723373	3.64E-05	0.405414985	1.93E-18
GRPEL1	brown	0.108974121	0.023827978	-0.372374129	1.37E-15
CSNK1G2	brown	0.34557842	1.66E-13	-0.472935561	2.39E-25
MAD2L1BP	brown	0.212727099	8.61E-06	-0.397854579	9.25E-18
TBX4	brown	-0.312902644	3.20E-11	0.701548477	5.79E-65
TKT	brown	0.125876972	0.008973986	-0.252972209	1.05E-07
TMEM41B	brown	0.201144429	2.65E-05	-0.313115589	3.10E-11
CCDC149	brown	-0.126571514	0.008599509	0.368796274	2.67E-15
CDH19	brown	-0.39297993	2.49E-17	0.497175285	3.15E-28
CCM2L	brown	-0.239370661	5.10E-07	0.521680006	2.20E-31
HAND1	brown	-0.398443238	8.20E-18	0.745610866	1.85E-77
SLC52A2	brown	0.465188617	1.78E-24	-0.579661454	5.80E-40
MICAL1	brown	-0.177134061	0.000222785	0.382933256	1.82E-16
SNX21	brown	-0.136169628	0.004674616	0.197511347	3.72E-05
SHFM1	brown	0.341278655	3.44E-13	-0.580089603	4.94E-40
FAM198A	brown	-0.100654694	0.036938251	0.331285441	1.79E-12
PDE2A	brown	-0.505927689	2.51E-29	0.758467122	1.32E-81
TMEM108	brown	-0.29273287	6.07E-10	0.599465161	2.64E-43
MYOM1	brown	-0.568645803	3.36E-38	0.860071397	3.58E-127
LCMT1	brown	0.223137552	2.97E-06	-0.312953762	3.18E-11
FERMT2	brown	-0.376564521	6.20E-16	0.842071564	8.06E-117
JAM3	brown	-0.420574862	7.35E-20	0.83673058	5.37E-114
PPP1R12B	brown	-0.499101492	1.82E-28	0.847997601	4.44E-120
SNRPF	brown	0.338511824	5.47E-13	-0.582679965	1.86E-40
FAM46A	brown	-0.298984535	2.50E-10	0.416987314	1.62E-19
WDR4	brown	0.351815416	5.66E-14	-0.387921533	6.83E-17
MICU1	brown	-0.052990302	0.27290281	0.265624658	2.23E-08
PTGES3L-AA	brown	-0.273648324	8.00E-09	0.324698673	5.15E-12
FAM134B	brown	-0.096211074	0.046163481	0.271713122	1.03E-08
EPAS1	brown	-0.077053394	0.110592649	0.21373008	7.79E-06
RGS9	brown	-0.299856233	2.20E-10	0.517807052	7.22E-31
CACNA1C	brown	-0.373043162	1.21E-15	0.857748923	9.32E-126
ANKRD29	brown	-0.311355063	4.04E-11	0.620095896	4.83E-47
TTLL11	brown	-0.272383593	9.43E-09	0.479048297	4.71E-26
ATP6V0B	brown	0.359896149	1.35E-14	-0.455300415	2.15E-23
KIZ	brown	-0.25231919	1.14E-07	0.234529528	8.75E-07
ADRBK1	brown	0.193216575	5.51E-05	-0.416735979	1.71E-19
GSK3A	brown	0.263114384	3.06E-08	-0.37741661	5.27E-16
FAM109A	brown	0.264198745	2.67E-08	-0.282185666	2.59E-09
TPSB2	brown	-0.330788809	1.94E-12	0.603401819	5.37E-44
ZDHHC11B	brown	-0.315098217	2.29E-11	0.238764354	5.46E-07
CNFN	brown	0.096082766	0.046456266	-0.276188593	5.74E-09
VIT	brown	-0.588240357	2.21E-41	0.698906841	2.75E-64
PKIG	brown	-0.351498228	5.98E-14	0.70523049	6.42E-66
IMP3	brown	0.20692179	1.52E-05	-0.553743787	6.41E-36
LMAN2	brown	0.242219089	3.69E-07	-0.405546088	1.88E-18
HBB	brown	-0.210238637	1.10E-05	0.33215698	1.56E-12
MTMR14	brown	0.24562602	2.50E-07	-0.281674826	2.77E-09
TM4SF18	brown	-0.028910537	0.549918965	0.280906049	3.07E-09
GPR116	brown	-0.139330605	0.003792264	0.460737043	5.53E-24
CPA3	brown	-0.322924715	6.81E-12	0.69073468	3.05E-62

JADE1	brown	-0.203405278	2.14E-05	0.448413777	1.17E-22
CNGA3	brown	-0.37034336	2.00E-15	0.49089132	1.85E-27
TECPR2	brown	-0.238056841	5.91E-07	0.508440076	1.20E-29
ELOVL2	brown	-0.114511364	0.017526857	0.401224069	4.62E-18
EWSR1	brown	0.245278912	2.60E-07	-0.404933571	2.13E-18
TACR3	brown	-0.454566908	2.58E-23	0.275027945	6.68E-09
HUNK	brown	-0.165207844	0.00058276	0.353738992	4.04E-14
PYGO1	brown	-0.202211966	2.39E-05	0.477487782	7.15E-26
MDFI	brown	0.203276193	2.16E-05	-0.3314129	1.76E-12
SMPD2	brown	0.216339314	5.99E-06	-0.457992445	1.10E-23
ABCB5	brown	-0.475887706	1.09E-25	0.62978848	6.75E-49
LTBP3	brown	-0.187682169	9.03E-05	0.400563644	5.30E-18
C2orf74	brown	-0.13126025	0.006415558	0.266003949	2.13E-08
ITPKB	brown	-0.427680247	1.50E-20	0.681952382	4.06E-60
PCDHB15	brown	-0.193766759	5.24E-05	0.335222729	9.42E-13
PPP1R3B	brown	-0.282589165	2.45E-09	0.508163838	1.30E-29
HS1BP3	brown	-0.046348644	0.3376476	0.153643007	0.001394775
PHACTR1	brown	-0.211949301	9.30E-06	0.475696566	1.15E-25
CSK	brown	0.288361474	1.11E-09	-0.363922685	6.53E-15
ITGA1	brown	-0.274406115	7.25E-09	0.741205726	4.26E-76
FAM149A	brown	-0.331340789	1.78E-12	0.441163105	6.62E-22
PCED1A	brown	0.251512604	1.25E-07	-0.34630572	1.47E-13
PER3	brown	-0.287923201	1.18E-09	0.405000571	2.10E-18
GABRG1	brown	-0.269114494	1.44E-08	0.221256864	3.62E-06
RDH16	brown	0.12904532	0.007376241	-0.189204116	7.89E-05
BOC	brown	-0.361988095	9.27E-15	0.841631113	1.39E-116
DEF6	brown	0.18545727	0.00010967	-0.390588117	4.02E-17
CDCP1	brown	0.24261406	3.53E-07	-0.347728209	1.15E-13
ARHGEF37	brown	-0.283566118	2.15E-09	0.41173698	5.04E-19
SCUBE1	brown	-0.389639418	4.86E-17	0.304109049	1.19E-10
TRIM29	brown	0.081179334	0.092715106	-0.371836944	1.51E-15
TEK	brown	-0.251056043	1.32E-07	0.725244968	2.21E-71
OSTN	brown	-0.381095234	2.60E-16	0.55257113	9.58E-36
TNRC6C	brown	-0.248933876	1.70E-07	0.527569408	3.51E-32
PRRG4	brown	0.095270813	0.048345395	-0.314908342	2.36E-11
TAGLN	brown	-0.340865871	3.69E-13	0.696757553	9.63E-64
HDHD1	brown	0.167182037	0.000499173	-0.22409143	2.69E-06
SAFB	brown	0.225298071	2.37E-06	-0.429667335	9.55E-21
AKT1S1	brown	0.277710058	4.70E-09	-0.416888863	1.65E-19
PRR7	brown	0.219071526	4.53E-06	-0.387119455	8.01E-17
BUD31	brown	0.289611978	9.38E-10	-0.433232852	4.22E-21
PPP1R15A	brown	-0.267724411	1.71E-08	0.141185761	0.003347564
CHMP7	brown	-0.138981559	0.003881683	0.222993884	3.02E-06
EOGT	brown	-0.171482362	0.000354161	0.334788425	1.01E-12
NES	brown	-0.234933131	8.37E-07	0.497252365	3.08E-28
EML1	brown	-0.323393872	6.33E-12	0.717534936	3.20E-69
ABCA7	brown	0.16985589	0.000403638	-0.233727782	9.55E-07
PABPN1	brown	0.334375956	1.08E-12	-0.511226518	5.25E-30
TMEM101	brown	0.185644707	0.000107895	-0.269759945	1.32E-08
BNC2	brown	-0.345720595	1.62E-13	0.811866614	4.47E-102
SEMA3D	brown	-0.152500502	0.001515581	0.284597883	1.87E-09

SSBP3	brown	-0.140150744	0.003589457	0.190654225	6.93E-05
F12	brown	0.153051653	0.001456153	-0.387217767	7.85E-17
PPP1R14B	brown	0.424093381	3.36E-20	-0.621683305	2.42E-47
EFHC2	brown	-0.194755947	4.79E-05	0.249012124	1.68E-07
HMGA1	brown	0.324533654	5.29E-12	-0.501983874	7.92E-29
ARHGAP1	brown	-0.249113002	1.66E-07	0.53896535	9.08E-34
CCL14	brown	-0.535583359	2.73E-33	0.687581052	1.80E-61
MRGBP	brown	0.387214101	7.86E-17	-0.487414266	4.85E-27
SEMA3G	brown	-0.34868386	9.74E-14	0.644991369	6.10E-52
GNE	brown	-0.110457613	0.021972412	0.254258302	9.03E-08
COX16	brown	0.274344811	7.31E-09	-0.478436405	5.55E-26
MPDZ	brown	-0.270517806	1.20E-08	0.633109547	1.51E-49
POLR3K	brown	0.259385422	4.85E-08	-0.414670522	2.68E-19
PSD4	brown	0.145933597	0.002416621	-0.310353968	4.70E-11
PID1	brown	-0.512768424	3.31E-30	0.734471444	4.58E-74
POPDC2	brown	-0.405931179	1.73E-18	0.826184464	1.04E-108
PPCS	brown	0.183791984	0.000126695	-0.236320543	7.18E-07
DOC2B	brown	-0.130360275	0.006791483	0.288171878	1.14E-09
TARSL2	brown	-0.287758763	1.21E-09	0.444679762	2.87E-22
LATS2	brown	-0.306629385	8.19E-11	0.673195186	4.50E-58
HIGD1B	brown	-0.094345571	0.050576068	0.129333224	0.007244494
USP51	brown	-0.182165491	0.000145694	0.460740935	5.52E-24
KRT17	brown	0.129050054	0.007374057	-0.357838698	1.95E-14
ATF3	brown	-0.365710055	4.71E-15	0.340185279	4.13E-13
DUS1L	brown	0.413161376	3.71E-19	-0.655686274	3.45E-54
MYBBP1A	brown	0.254608075	8.66E-08	-0.377027063	5.67E-16
TNS1	brown	-0.451727455	5.20E-23	0.873650957	5.45E-136
POLR1C	brown	0.278940042	3.99E-09	-0.566613361	6.98E-38
GFRA3	brown	-0.323915814	5.83E-12	0.388735111	5.81E-17
SORCS1	brown	-0.659788703	4.47E-55	0.583469053	1.38E-40
APOD	brown	-0.229138164	1.57E-06	0.446947623	1.66E-22
IL6ST	brown	-0.328649968	2.74E-12	0.715205641	1.39E-68
SBSPON	brown	-0.369189904	2.48E-15	0.749788011	8.85E-79
C17orf96	brown	0.183610442	0.000128694	-0.385164869	1.18E-16
C2orf40	brown	-0.592882181	3.62E-42	0.839315975	2.38E-115
MYPOP	brown	0.333152661	1.32E-12	-0.412462622	4.31E-19
C3orf36	brown	0.00464106	0.923552784	0.234757909	8.53E-07
PLN	brown	-0.390235935	4.31E-17	0.804772429	5.37E-99
NDNF	brown	-0.390976328	3.72E-17	0.395659227	1.45E-17
ETHE1	brown	0.133028582	0.005730809	-0.278980828	3.97E-09
TMEM132C	brown	-0.629545057	7.53E-49	0.512756607	3.32E-30
WFDC1	brown	-0.453800446	3.12E-23	0.613483908	8.17E-46
MRPS7	brown	0.262735306	3.21E-08	-0.446225331	1.98E-22
EDA	brown	-0.167672207	0.00048022	0.241343655	4.08E-07
POLB	brown	0.20360915	2.10E-05	-0.327433361	3.33E-12
SH2D3C	brown	-0.112243384	0.019906024	0.473002543	2.35E-25
LIN7A	brown	-0.056864635	0.239319236	0.286193841	1.50E-09
GABRD	brown	0.17433576	0.00028076	-0.004073421	0.932879351
ABHD4	brown	0.007816804	0.871602473	0.191733298	6.29E-05
PNKP	brown	0.34875634	9.62E-14	-0.511284853	5.16E-30
FAH	brown	0.123146082	0.010590946	-0.255601745	7.68E-08

COMMD5	brown	0.308284048	6.41E-11	-0.366875407	3.80E-15
HK2	brown	0.172401063	0.000328773	-0.367772462	3.22E-15
ZBTB8OS	brown	0.259044542	5.06E-08	-0.314178961	2.64E-11
GPR133	brown	-0.562491081	3.03E-37	0.871517712	1.54E-134
CHCHD7	brown	0.131258706	0.006416187	-0.232257625	1.12E-06
LURAP1L	brown	-0.106264548	0.027568232	0.258214327	5.60E-08
NAP1L5	brown	-0.362611621	8.28E-15	0.458614897	9.43E-24
GHR	brown	-0.392629937	2.67E-17	0.664740112	3.65E-56
EFNA4	brown	0.386073887	9.84E-17	-0.501153972	1.01E-28
ASB5	brown	-0.520745391	2.94E-31	0.815733548	8.25E-104
CAPG	brown	0.234415912	8.86E-07	-0.259858126	4.58E-08
GPR155	brown	-0.24022625	4.63E-07	0.543610399	1.96E-34
HAND2	brown	-0.451715031	5.21E-23	0.811205082	8.78E-102
KLHDC1	brown	-0.33007341	2.18E-12	0.542351292	2.98E-34
PLCXD3	brown	-0.02225958	0.645302415	0.186129945	0.000103424
SERPINI2	brown	-0.112184064	0.019971851	0.223874447	2.75E-06
SHISA5	brown	0.198268001	3.46E-05	-0.319126686	1.23E-11
GNG11	brown	-0.350336499	7.32E-14	0.660789669	2.71E-55
ACR	brown	-0.178900481	0.000192174	0.330650592	1.99E-12
TNXB	brown	-0.605087371	2.69E-44	0.815845196	7.34E-104
CTU1	brown	0.289470104	9.56E-10	-0.455504547	2.05E-23
RAMP2	brown	-0.159800279	0.000882799	0.395938466	1.37E-17
SMTN	brown	-0.465225112	1.77E-24	0.702745857	2.84E-65
S100A2	brown	0.074847277	0.121208084	-0.32879199	2.68E-12
MAP1A	brown	-0.390272937	4.28E-17	0.839984775	1.05E-115
COQ3	brown	0.13087352	0.00657475	-0.352374393	5.13E-14
NXPH3	brown	-0.42173926	5.68E-20	0.728907373	1.96E-72
CNPY2	brown	0.357992381	1.90E-14	-0.484888982	9.69E-27
ADAMTS8	brown	-0.480773506	2.96E-26	0.77921424	7.12E-89
GJB5	brown	0.072210928	0.134916746	-0.380661713	2.82E-16
CNTN4	brown	-0.38109635	2.60E-16	0.590862847	7.98E-42
MMAB	brown	0.164188846	0.000630818	-0.350556382	7.04E-14
PRCD	brown	-0.241636786	3.95E-07	0.345451172	1.70E-13
PBX3	brown	-0.186776248	9.77E-05	0.518357526	6.11E-31
RCE1	brown	0.265372747	2.31E-08	-0.450847331	6.45E-23
DMPK	brown	-0.33183313	1.64E-12	0.496856289	3.45E-28
C2orf71	brown	-0.235181463	8.14E-07	0.247974516	1.90E-07
C15orf61	brown	0.227929639	1.79E-06	-0.300510343	2.00E-10
NRIP2	brown	-0.239539727	5.01E-07	0.484182196	1.18E-26
CRY2	brown	-0.484896744	9.67E-27	0.65107184	3.30E-53
ARHGEF35	brown	0.156512276	0.001129343	-0.232725931	1.07E-06
TACSTD2	brown	0.224681597	2.53E-06	-0.475594908	1.18E-25
MMACHC	brown	0.288020223	1.17E-09	-0.35095179	6.58E-14
SORBS1	brown	-0.445600009	2.30E-22	0.858238972	4.71E-126
AGA	brown	0.137858371	0.004182522	-0.186349274	0.00010146
ABCA3	brown	-0.095789845	0.047130523	0.351788226	5.68E-14
SLC12A9	brown	0.302612985	1.48E-10	-0.371523653	1.60E-15
C10orf32	brown	-0.219742578	4.23E-06	0.32169138	8.27E-12
ERP29	brown	0.205899187	1.68E-05	-0.316255888	1.92E-11
PSENEN	brown	0.275640281	6.17E-09	-0.447440384	1.48E-22
LY75	brown	0.100041097	0.038110676	-0.225278825	2.37E-06

STARD10	brown	0.207483397	1.44E-05	-0.468264965	8.07E-25
NCLN	brown	0.350661773	6.92E-14	-0.509412066	9.00E-30
ADAMTS5	brown	-0.323395168	6.33E-12	0.607757361	8.96E-45
BIK	brown	0.117791128	0.014526538	-0.250825728	1.36E-07
SFRP5	brown	-0.292615608	6.17E-10	0.476118073	1.03E-25
PRSS35	brown	-0.283635599	2.13E-09	0.649138516	8.40E-53
MYRIP	brown	-0.387393343	7.58E-17	0.526839708	4.42E-32
TPM2	brown	-0.321449886	8.59E-12	0.647663873	1.71E-52
IL17D	brown	-0.168602299	0.000446084	0.295210759	4.28E-10
GRB10	brown	-0.220796417	3.79E-06	0.344026477	2.16E-13
NACC1	brown	0.284855321	1.80E-09	-0.511468848	4.88E-30
FOXP2	brown	-0.453506159	3.36E-23	0.713050059	5.36E-68
CYB561D2	brown	0.259841475	4.59E-08	-0.315900235	2.03E-11
DIO3	brown	-0.181137125	0.000159053	0.423496378	3.84E-20
GPER1	brown	-0.340682848	3.80E-13	0.593297442	3.07E-42
RERGL	brown	-0.572736228	7.57E-39	0.610322464	3.08E-45
FLNA	brown	-0.320701659	9.65E-12	0.664501052	4.12E-56
DIP2C	brown	-0.234598495	8.68E-07	0.517512088	7.90E-31
P2RY2	brown	0.147552273	0.002157781	-0.294024248	5.06E-10
GNA15	brown	0.135539531	0.004871212	-0.414860095	2.57E-19
FILIP1L	brown	-0.380028465	3.19E-16	0.777102095	4.25E-88
SNX29	brown	-0.136839174	0.004473582	0.496781807	3.52E-28
CCDC27	brown	-0.402917263	3.25E-18	0.488885404	3.23E-27
NUDT16	brown	-0.126002006	0.008905519	0.272795491	8.94E-09
NMUR1	brown	-0.289105595	1.01E-09	0.486449527	6.32E-27
MRPS26	brown	0.345732293	1.62E-13	-0.548704388	3.57E-35
GALNT3	brown	0.200393141	2.84E-05	-0.31054925	4.57E-11
MUSTN1	brown	-0.322570686	7.20E-12	0.430161394	8.54E-21
PHF21A	brown	-0.137669235	0.004235206	0.384655117	1.30E-16
ACOT8	brown	0.263585383	2.88E-08	-0.382935677	1.82E-16
NFIA	brown	-0.380088449	3.15E-16	0.545748356	9.63E-35
TBXA2R	brown	-0.081367938	0.091956698	0.418862528	1.07E-19
TMEM161A	brown	0.313498741	2.92E-11	-0.481454429	2.47E-26
CIDEC	brown	-0.220012628	4.11E-06	0.475005131	1.38E-25
TTLL12	brown	0.158538481	0.000970849	-0.340198412	4.13E-13
ST6GALNAC3	brown	-0.249297856	1.63E-07	0.511942146	4.24E-30
C14orf180	brown	-0.163781026	0.000651061	0.363988038	6.45E-15
MGARP	brown	-0.317473113	1.59E-11	0.55638221	2.58E-36
TXNL4A	brown	0.22599923	2.20E-06	-0.355783742	2.82E-14
TSPO	brown	0.179226491	0.000186974	-0.495028416	5.79E-28
BAHCC1	brown	-0.077435778	0.108829433	0.266210588	2.07E-08
ABT1	brown	0.277873851	4.60E-09	-0.484251117	1.15E-26
PSMG3	brown	0.449623762	8.69E-23	-0.567917723	4.36E-38
CLU	brown	-0.259335713	4.88E-08	0.410737283	6.24E-19
AHDC1	brown	-0.156078676	0.001166217	0.177220269	0.000221191
FRMD8	brown	0.193455626	5.39E-05	-0.330439698	2.06E-12
ARPC3	brown	0.34850079	1.01E-13	-0.555041645	4.10E-36
PFDN4	brown	0.275554638	6.24E-09	-0.430935065	7.15E-21
WFDC10B	brown	0.018230923	0.70619191	0.167438605	0.000489167
RGMB	brown	-0.114049735	0.017989915	0.262474974	3.31E-08
CTIF	brown	-0.235759418	7.64E-07	0.514587314	1.92E-30

KY	brown	-0.524257937	9.90E-32	0.661013055	2.42E-55
TRNAU1AP	brown	0.205224808	1.79E-05	-0.300256829	2.08E-10
COL6A5	brown	-0.219547652	4.32E-06	0.10044645	0.037332673
ADCK3	brown	-0.14638745	0.002341328	0.26424221	2.66E-08
COX7A1	brown	-0.417552609	1.43E-19	0.699220877	2.28E-64
CABLES1	brown	-0.101683701	0.035040799	0.186311846	0.000101793
SAMD1	brown	0.33974421	4.45E-13	-0.468189818	8.23E-25
STAB2	brown	-0.379198921	3.74E-16	0.634574949	7.75E-50
CCDC137	brown	0.405277255	1.98E-18	-0.566826005	6.47E-38
CPEB1	brown	-0.560348284	6.46E-37	0.816979061	2.24E-104
SLIT3	brown	-0.48439477	1.11E-26	0.646700227	2.70E-52
SFRP1	brown	-0.375583875	7.46E-16	0.658713948	7.66E-55
KCNAB1	brown	-0.336243099	7.96E-13	0.618328583	1.04E-46
PKDCC	brown	-0.331098985	1.85E-12	0.727152704	6.30E-72
CCDC74A	brown	-0.025338506	0.600288893	0.208527856	1.30E-05
FAM50B	brown	-0.218879363	4.62E-06	0.274613537	7.06E-09
HTR2B	brown	-0.164708503	0.000605868	0.467991053	8.66E-25
RNF150	brown	-0.476643532	8.96E-26	0.800702746	2.75E-97
TMEM8B	brown	-0.275612103	6.19E-09	0.282750614	2.40E-09
INO80C	brown	0.166622582	0.000521652	-0.416416637	1.83E-19
SSPN	brown	-0.349137508	9.01E-14	0.57995849	5.19E-40
OTC	brown	-0.313060273	3.13E-11	0.470993969	3.97E-25
HBD	brown	0.014232386	0.768540232	0.16036344	0.000845933
COQ4	brown	0.206030354	1.66E-05	-0.493016657	1.02E-27
SF3A2	brown	0.275304091	6.45E-09	-0.415549814	2.21E-19
TOP1MT	brown	0.20031876	2.86E-05	-0.411601515	5.19E-19
R3HDM4	brown	0.159414051	0.000908934	-0.378436237	4.33E-16
BLOC1S4	brown	0.262299166	3.38E-08	-0.426547428	1.94E-20
FAM229B	brown	-0.233818554	9.46E-07	0.411774571	5.00E-19
OGN	brown	-0.560890738	5.34E-37	0.850222191	2.44E-121
MOGS	brown	0.485860603	7.43E-27	-0.589311213	1.46E-41
SPSB2	brown	0.253037425	1.05E-07	-0.324842386	5.03E-12
OSBPL10	brown	-0.341631982	3.24E-13	0.647808376	1.59E-52
F8	brown	-0.351075237	6.44E-14	0.68586521	4.69E-61
TLE4	brown	-0.245338751	2.58E-07	0.330025641	2.20E-12
RRP1	brown	0.295883772	3.89E-10	-0.522790851	1.56E-31
SAMD11	brown	-0.191183397	6.61E-05	0.29216626	6.57E-10
GOT1L1	brown	-0.090490056	0.060816444	0.214757353	7.03E-06
PFDN6	brown	0.364590444	5.78E-15	-0.58935672	1.43E-41
C4B	brown	-0.197930501	3.57E-05	0.633182932	1.46E-49
ADCYAP1	brown	-0.357917348	1.93E-14	0.62343724	1.13E-47
APRT	brown	0.272810503	8.92E-09	-0.524314409	9.73E-32
FAM131C	brown	0.137747058	0.004213456	-0.250607787	1.40E-07
CTD-2369P2	brown	0.17203047	0.000338803	-0.220268662	4.01E-06
MREG	brown	0.262855809	3.16E-08	-0.353548195	4.18E-14
ODF3L1	brown	-0.481355645	2.53E-26	0.351190438	6.31E-14
ARF5	brown	0.278417602	4.28E-09	-0.513761321	2.46E-30
ARL14EP	brown	-0.161383917	0.000782733	0.318533515	1.35E-11
GPX3	brown	-0.241704435	3.92E-07	0.442792976	4.50E-22
RAB23	brown	-0.360624683	1.19E-14	0.797536132	5.53E-96
RASD1	brown	-0.306367423	8.51E-11	0.395606805	1.46E-17

SH3BGRL	brown	-0.261238807	3.86E-08	0.512447358	3.65E-30
SSUH2	brown	-0.071152515	0.140744942	0.272118767	9.76E-09
NSDHL	brown	0.255354962	7.92E-08	-0.474820941	1.45E-25
PIEZO2	brown	-0.174109063	0.000286026	0.50283061	6.20E-29
KLHL38	brown	-0.176573407	0.000233418	0.58143366	2.98E-40
MRPL17	brown	0.357631032	2.03E-14	-0.501303142	9.64E-29
CWC15	brown	0.232299835	1.12E-06	-0.394107267	1.98E-17
HPSE2	brown	-0.464983242	1.88E-24	0.816185029	5.15E-104
GPBAR1	brown	-0.371996215	1.47E-15	0.680646821	8.28E-60
LHX6	brown	-0.125773664	0.009030909	0.431966018	5.65E-21
REEP1	brown	-0.447294125	1.53E-22	0.723329232	7.74E-71
TMEM100	brown	-0.454118289	2.89E-23	0.750590232	4.91E-79
MTFP1	brown	0.274172357	7.47E-09	-0.472553671	2.64E-25
SEZ6L2	brown	0.16714679	0.000500562	-0.17654151	0.000234037
IPO4	brown	0.246939271	2.15E-07	-0.384866425	1.25E-16
RFTN2	brown	-0.264592486	2.54E-08	0.628953088	9.82E-49
RPS6KA1	brown	0.297542317	3.07E-10	-0.439572285	9.64E-22
PIPOX	brown	-0.25630454	7.06E-08	0.4576448	1.20E-23
HMCN2	brown	-0.503627248	4.92E-29	0.767861734	8.39E-85
ANO5	brown	-0.336439534	7.71E-13	0.575901218	2.36E-39
SMAD9	brown	-0.281308815	2.91E-09	0.463741948	2.58E-24
CCDC58	brown	0.396272265	1.28E-17	-0.632340913	2.14E-49
CYP21A2	brown	-0.142548707	0.003051606	0.501506388	9.09E-29
MEX3D	brown	0.278007184	4.52E-09	-0.336487539	7.65E-13
LETM1	brown	0.229022389	1.59E-06	-0.409939924	7.40E-19
TSC22D1	brown	-0.367557481	3.35E-15	0.504991492	3.31E-29
GJC2	brown	-0.383635009	1.59E-16	0.558908749	1.07E-36
CLEC4F	brown	-0.132083566	0.006088109	0.218330984	4.89E-06
CYB561	brown	0.39308334	2.44E-17	-0.483845466	1.29E-26
MRPL48	brown	0.245408128	2.56E-07	-0.458076407	1.08E-23
NOC2L	brown	0.28174987	2.74E-09	-0.478145737	6.00E-26
CEP131	brown	0.328697818	2.72E-12	-0.403818031	2.69E-18
CYB5R2	brown	0.191958739	6.17E-05	-0.388435988	6.17E-17
TBX20	brown	-0.457911883	1.12E-23	0.705071322	7.06E-66
LGI1	brown	-0.346033965	1.54E-13	0.338793089	5.22E-13
P2RY14	brown	-0.480377791	3.30E-26	0.804367377	7.98E-99
MMRN1	brown	-0.434227763	3.35E-21	0.705879682	4.34E-66
C19orf52	brown	0.303984988	1.21E-10	-0.349329176	8.72E-14
SLC1A5	brown	0.090293341	0.061381794	-0.280116971	3.41E-09
TAL1	brown	-0.176057813	0.000243613	0.452969095	3.83E-23
NR4A2	brown	-0.454749447	2.47E-23	0.405517271	1.89E-18
FOXJ2	brown	-0.137874791	0.004177976	0.367134624	3.62E-15
RYR3	brown	-0.57844755	9.15E-40	0.723551226	6.70E-71
GPR112	brown	-0.592692556	3.90E-42	0.506582481	2.07E-29
ITPA	brown	0.358986748	1.59E-14	-0.516111103	1.21E-30
GFRA2	brown	-0.393325954	2.32E-17	0.663799906	5.89E-56
GATA1	brown	-0.276874468	5.24E-09	0.48082481	2.92E-26
FUNDC1	brown	0.205205185	1.80E-05	-0.375353312	7.80E-16
STK11IP	brown	0.15090302	0.001700634	-0.2220046	3.35E-06
APITD1	brown	0.297657308	3.02E-10	-0.441983068	5.45E-22
GYPE	brown	-0.293495714	5.45E-10	0.616674451	2.10E-46

NCS1	brown	-0.31343566	2.95E-11	0.471910166	3.12E-25
IGSF9B	brown	-0.479127731	4.61E-26	0.716431915	6.44E-69
IRS2	brown	-0.085283498	0.077303947	0.334947739	9.86E-13
CTSF	brown	-0.217419328	5.37E-06	0.369285442	2.43E-15
AKAP2	brown	-0.396365995	1.25E-17	0.693567432	6.07E-63
ITGB3	brown	-0.290468148	8.33E-10	0.664159647	4.90E-56
LMOD1	brown	-0.454570491	2.58E-23	0.817005595	2.17E-104
ADCY9	brown	-0.284007504	2.02E-09	0.607847578	8.63E-45
C17orf70	brown	0.312886827	3.21E-11	-0.406215176	1.63E-18
AOC3	brown	-0.429424958	1.01E-20	0.781850589	7.45E-90
ESD	brown	-0.167730669	0.000478005	0.313734079	2.82E-11
PCOLCE2	brown	-0.493541035	8.80E-28	0.6452548	5.38E-52
TIE1	brown	-0.189259687	7.85E-05	0.600691993	1.61E-43
SOD3	brown	-0.293465028	5.48E-10	0.592184754	4.76E-42
CLEC1A	brown	-0.167829045	0.000474299	0.413779327	3.24E-19
STARD8	brown	-0.280635285	3.18E-09	0.744619221	3.76E-77
ZNF853	brown	-0.167347873	0.000492684	0.283219691	2.25E-09
ZHX1-C8orf	brown	0.291460265	7.25E-10	-0.375415255	7.71E-16
SPINT1	brown	0.338769705	5.24E-13	-0.510890697	5.80E-30
FAM102A	brown	-0.194371722	4.96E-05	0.23526697	8.06E-07
PDCD5	brown	0.332058955	1.58E-12	-0.443242987	4.04E-22
CYSRT1	brown	0.065440273	0.175576731	-0.25569454	7.60E-08
MAPT	brown	-0.306055178	8.92E-11	0.46942172	5.98E-25
GLP2R	brown	-0.620788852	3.58E-47	0.79321436	3.05E-94
TSC22D4	brown	0.242050654	3.77E-07	-0.366169528	4.33E-15
FZD7	brown	-0.351119836	6.39E-14	0.573356205	6.03E-39
SLC35C2	brown	0.346817183	1.34E-13	-0.472056851	3.01E-25
CPED1	brown	-0.520938797	2.77E-31	0.847134753	1.35E-119
TPSAB1	brown	-0.345665624	1.64E-13	0.612922006	1.04E-45
SELP	brown	-0.269329446	1.40E-08	0.535907726	2.45E-33
OCIAD2	brown	0.360542464	1.20E-14	-0.61795163	1.22E-46
BOLA1	brown	0.259203437	4.96E-08	-0.313836922	2.78E-11
ST8SIA6	brown	-0.11855167	0.013898957	0.324165103	5.60E-12
FAM83G	brown	0.13534931	0.004932011	-0.309667524	5.21E-11
POP7	brown	0.405494019	1.90E-18	-0.585314965	6.81E-41
FBXW9	brown	0.367086629	3.66E-15	-0.470614288	4.38E-25
FOXF1	brown	-0.483210586	1.53E-26	0.751292932	2.92E-79
ATPAF2	brown	0.169105991	0.000428554	-0.270603772	1.19E-08
DUSP19	brown	-0.238592389	5.57E-07	0.404741481	2.22E-18
CNDP2	brown	0.166411466	0.000530376	-0.245558743	2.52E-07

MEgreen	MEgreen	MEyellow	MEyellow	MEmagenta
-0.322832941	6.91E-12	-0.120848392	0.012146183	0.068486495
-0.271229496	1.09E-08	-0.307545312	7.15E-11	0.009240594
-0.216474287	5.91E-06	-0.038112094	0.430524084	0.052456588
-0.009434212	0.845340596	-0.012941243	0.789018488	-0.021921574
0.211414587	9.81E-06	0.094356353	0.050549588	0.01463407
-0.240835349	4.32E-07	-0.114782222	0.017260037	0.018677967
0.046333324	0.337807485	0.123319487	0.010481073	-0.023764898
-0.188158928	8.65E-05	-0.0017816	0.970615352	0.096179583
-0.252683734	1.09E-07	-0.229059707	1.59E-06	-0.02433345
0.07625775	0.114333469	-0.050597557	0.295178096	0.056420876
0.229897272	1.45E-06	0.125558686	0.009150395	-0.053942549
-0.128704793	0.007534829	-0.164545371	0.0006136	-0.102486411
-0.287925166	1.18E-09	-0.065396247	0.175868066	0.122492472
0.710493101	2.61E-67	0.413748115	3.27E-19	-0.081699723
-0.232095006	1.14E-06	-0.251384854	1.27E-07	0.017726118
-0.13142108	0.006350378	-0.154048583	0.001354064	0.072756592
-0.010506475	0.828022317	0.155432424	0.001223237	-0.03657866
0.493113027	9.93E-28	0.217649134	5.24E-06	-0.04684731
-0.502767431	6.31E-29	-0.311330595	4.06E-11	0.173952563
0.445666179	2.26E-22	0.184141504	0.000122928	-0.023938347
-0.152509793	0.001514562	-0.196614013	4.04E-05	0.066671613
0.222412081	3.21E-06	0.064805325	0.1798132	0.009887261
0.504984068	3.31E-29	0.299317204	2.38E-10	-0.025735992
0.228620467	1.66E-06	0.156416366	0.001137406	0.022000845
0.482998343	1.62E-26	0.433291471	4.16E-21	-0.043627965
-0.348547047	9.98E-14	-0.218759105	4.68E-06	0.045410573
-0.119270535	0.013327823	-0.033071911	0.493984889	0.033275143
-0.230864229	1.31E-06	-0.183571666	0.000129125	0.058175495
-0.321409354	8.64E-12	-0.134644319	0.00516335	0.090609735
0.153676385	0.001391383	0.082188696	0.088713959	0.035372602
-0.240101562	4.70E-07	-0.188704918	8.25E-05	-0.038907097
-0.298010983	2.87E-10	-0.259389317	4.85E-08	0.0289364
0.28524579	1.71E-09	0.277632441	4.75E-09	0.006706751
-0.244432689	2.87E-07	-0.114013479	0.018026731	-0.016488578
-0.295853503	3.91E-10	-0.119804016	0.012917441	0.029332175
-0.016607626	0.731294333	-0.147787111	0.002122408	0.139718233
-0.082355009	0.088068196	0.019328313	0.689398791	0.024414191
-0.206761457	1.55E-05	0.012641766	0.793789756	0.050527586
0.248141014	1.86E-07	0.095231532	0.048438395	-0.002567901
0.046716998	0.333817981	-0.094669026	0.049786673	0.081819754
-0.119777346	0.012937689	-0.04919726	0.308762112	-0.090022227
0.357900817	1.93E-14	0.235522113	7.84E-07	-0.000657491
0.66381447	5.85E-56	0.473443073	2.09E-25	-0.061294703
0.211126545	1.01E-05	0.025308708	0.600717694	0.009789641
-0.188110897	8.69E-05	-0.084696978	0.079369699	-0.021420886
0.3546618	3.43E-14	0.092719725	0.054703873	-0.003412436
0.295650466	4.02E-10	0.193506602	5.36E-05	0.011171559
0.340787033	3.74E-13	0.157095653	0.001081425	0.060547204
0.054696232	0.257739119	0.039577274	0.413001166	0.130341183
0.532189386	8.11E-33	0.394055246	2.00E-17	0.087722367

0.5707107	1.59E-38	0.292719374	6.08E-10	-0.064504951
0.345204621	1.77E-13	0.149975254	0.001817394	-0.021996815
-0.339785702	4.42E-13	-0.194145372	5.06E-05	0.123141424
0.287728475	1.22E-09	0.124658313	0.00966624	-0.067380252
0.23547134	7.88E-07	0.117526873	0.01475035	-0.101039509
0.424173458	3.30E-20	0.296264089	3.69E-10	-0.021506835
-0.258163886	5.63E-08	-0.046536212	0.335694034	0.097174831
-0.478972137	4.81E-26	-0.35566843	2.87E-14	-0.046490774
0.195969055	4.28E-05	0.019043664	0.693740488	0.001007067
0.194563391	4.87E-05	0.166880447	0.000511176	0.085018743
-0.054018139	0.263695326	0.105510469	0.028694925	-0.006628497
0.279234029	3.84E-09	0.082576258	0.087214988	0.084208678
0.103855949	0.031306149	-0.058349856	0.227249154	0.078174507
0.164122574	0.000634067	-0.020307428	0.674542937	-0.0058566
-0.030276959	0.53121499	-0.052098267	0.281069417	0.093211267
0.430650595	7.63E-21	0.179417904	0.000183983	-0.037673265
0.389664965	4.83E-17	0.209804314	1.15E-05	-0.055903261
-0.308503263	6.20E-11	-0.001898993	0.968680094	0.121394964
0.4850381	9.30E-27	0.289335288	9.74E-10	-0.043752672
0.614235946	5.94E-46	0.360918243	1.13E-14	-0.038588255
0.59858711	3.76E-43	0.394790594	1.73E-17	-0.104867465
-0.095750339	0.047222085	-0.245532052	2.53E-07	-0.019290945
0.269817047	1.31E-08	0.073625275	0.127420925	-0.007627031
0.272195945	9.66E-09	0.034295824	0.478130943	-0.040408752
-0.302628633	1.47E-10	-0.128286638	0.007733734	0.047617171
0.417021003	1.60E-19	0.222240125	3.27E-06	-0.019343157
0.415571959	2.20E-19	0.211741277	9.50E-06	-0.010612202
0.061794312	0.200936982	-0.085694195	0.0758836	-0.005301392
0.400637962	5.22E-18	0.225433914	2.34E-06	-0.001614994
0.327398975	3.35E-12	0.191055878	6.69E-05	-0.001404805
-0.022438035	0.642655611	0.029348079	0.543894721	0.115722463
0.038749947	0.422843363	0.04449514	0.35734235	0.014690293
0.452233518	4.59E-23	0.24220462	3.70E-07	0.005437479
0.330821963	1.93E-12	0.189117864	7.95E-05	0.022035656
0.332646229	1.44E-12	0.073121742	0.130051663	-0.040628564
0.491586472	1.52E-27	0.358946672	1.60E-14	-0.069832484
0.247596915	1.99E-07	0.208789306	1.27E-05	0.016337888
0.727425796	5.25E-72	0.489456783	2.75E-27	-0.079074425
0.230886435	1.30E-06	0.239137475	5.24E-07	0.061643383
0.366100428	4.38E-15	0.156049429	0.001168744	0.017901392
-0.262800132	3.18E-08	-0.142429892	0.003076426	0.084003149
-0.281278811	2.92E-09	-0.121258642	0.011854526	-0.049322137
0.597723708	5.31E-43	0.428207107	1.33E-20	-0.107865687
0.75981339	4.70E-82	0.487338636	4.95E-27	-0.060893703
-0.076552417	0.112936649	0.131176039	0.006449925	0.008182924
0.363877355	6.58E-15	0.071694711	0.137735665	-0.041827055
0.238600109	5.56E-07	0.173176017	0.000308689	-0.091229497
0.487180169	5.17E-27	0.268408009	1.57E-08	0.026879974
0.293082513	5.78E-10	0.120041914	0.012738057	-0.011008344
-0.201546772	2.55E-05	0.023266208	0.630431838	-0.000689215
-0.113815008	0.018229437	0.044776398	0.354308261	0.00887089

0.167627646	0.000481915	-0.090047844	0.062093419	0.008885955
-0.161371961	0.000783448	-0.175355837	0.000258163	0.00035581
0.023933005	0.620662498	-0.094133097	0.051100279	0.002899414
0.122857272	0.010776208	0.046374034	0.337382732	0.062257607
-0.123292256	0.01049826	-0.081101804	0.093028311	0.020188064
-0.151477269	0.001631853	0.001668541	0.972479307	0.046685359
0.348229322	1.05E-13	0.26850254	1.55E-08	0.035710667
-0.246086867	2.37E-07	-0.158615639	0.00096524	0.022564634
0.510792331	5.97E-30	0.223421014	2.89E-06	-0.016469968
0.317146094	1.67E-11	0.357916778	1.93E-14	-0.059214706
-0.407328753	1.29E-18	-0.274350476	7.30E-09	0.024897852
0.366091733	4.39E-15	0.230672226	1.33E-06	0.022929809
0.333794059	1.19E-12	0.183056041	0.000134984	0.010344071
-0.423242958	4.06E-20	-0.218846798	4.64E-06	0.031973873
-0.63826028	1.43E-50	-0.404527828	2.32E-18	0.124076001
0.306065029	8.90E-11	0.141945241	0.003179581	0.024077565
-0.235736393	7.66E-07	-0.126088566	0.008858392	0.014466119
0.30584766	9.19E-11	0.133534259	0.005547461	-0.04277381
0.276565886	5.46E-09	0.115035338	0.01701391	-0.065764546
0.364507863	5.87E-15	0.331981901	1.60E-12	-0.008789143
0.284289518	1.95E-09	0.064941775	0.178896461	-0.0319381
0.258802922	5.21E-08	0.287160257	1.31E-09	-0.03443556
0.501121455	1.02E-28	0.263681289	2.85E-08	-0.02891611
0.303000928	1.40E-10	0.122853185	0.01077885	0.183527547
0.523970511	1.08E-31	0.38519341	1.17E-16	-0.060486663
0.046351635	0.337616392	0.078346727	0.104718195	-0.011106281
0.097758365	0.042752997	0.050721106	0.293999015	-0.008482957
0.755657342	1.12E-80	0.498342541	2.26E-28	-0.073713617
0.203953192	2.03E-05	0.197502861	3.72E-05	0.079756546
0.155117719	0.001251923	-0.082232671	0.088542846	0.053713344
-0.582691222	1.85E-40	-0.287738958	1.21E-09	0.069950526
-0.248719135	1.74E-07	-0.025916639	0.591996883	0.07826603
-0.304394817	1.14E-10	-0.058652748	0.224841655	0.12261815
-0.141691726	0.003234783	-0.062105902	0.198670814	0.030391462
0.05021497	0.298849265	0.07071074	0.143233902	-0.01565012
0.381000681	2.65E-16	0.205505155	1.75E-05	-0.01369422
-0.413279244	3.61E-19	-0.247640315	1.98E-07	0.009180755
-0.278801453	4.06E-09	-0.100845916	0.03657919	0.011696738
0.335648973	8.78E-13	0.188548651	8.36E-05	0.002312213
0.400238065	5.66E-18	0.381197622	2.55E-16	0.041237016
-0.520520432	3.15E-31	-0.307319152	7.40E-11	0.045158066
0.441640481	5.91E-22	0.211109942	1.01E-05	-0.112228996
0.137810002	0.004195938	0.09599696	0.046652935	-0.034298279
-0.276844052	5.27E-09	-0.072694639	0.132315854	0.087678539
-0.064970938	0.178700978	-0.062335733	0.197011236	0.003780211
0.535552968	2.75E-33	0.355425901	3.00E-14	0.015255321
-0.194505231	4.90E-05	-0.073270557	0.129269836	0.016688698
-0.238745859	5.47E-07	-0.060145107	0.213244943	0.091801099
0.35700703	2.27E-14	0.339816343	4.40E-13	-0.030142844
-0.418460107	1.17E-19	-0.184988799	0.000114228	0.033306065
0.218813169	4.65E-06	0.126547595	0.008612171	-0.004149382

0.041703	0.388340992	-0.052010839	0.281878621	0.024636149
0.407099486	1.35E-18	0.314997242	2.33E-11	0.011568485
-0.47936931	4.32E-26	-0.245115366	2.65E-07	0.156744113
-0.350946435	6.58E-14	-0.208203972	1.35E-05	0.068173048
0.085742996	0.075716247	0.012763906	0.79184287	0.058950646
-0.322707325	7.05E-12	-0.243637511	3.14E-07	0.010334666
-0.307364502	7.35E-11	-0.240104816	4.70E-07	-0.029260399
0.44624129	1.97E-22	0.224721632	2.52E-06	-0.01537473
0.478481518	5.48E-26	0.22033645	3.98E-06	-0.020528291
0.207589611	1.43E-05	0.109201314	0.023535304	-0.005431381
-0.102377422	0.033808935	-0.053060204	0.272269761	-0.021606784
0.612872967	1.06E-45	0.364234436	6.17E-15	-0.028009025
-0.009750694	0.840220905	-0.042890349	0.374964434	0.008306056
0.009397157	0.845940462	0.141233146	0.003336852	-0.016039231
0.252261188	1.15E-07	0.143119038	0.002934978	0.110606953
-0.498536974	2.13E-28	-0.256422757	6.96E-08	0.127706109
-0.281935894	2.67E-09	-0.160649211	0.000827775	-0.027172311
-0.225781144	2.25E-06	-0.110906421	0.021436311	-0.00248351
0.476507718	9.28E-26	0.190621948	6.95E-05	0.061881235
0.27776616	4.66E-09	0.079017342	0.101770903	-0.027781398
-0.240304967	4.59E-07	-0.186291615	0.000101973	0.01682685
0.255932301	7.38E-08	0.341092372	3.55E-13	-0.045489227
0.504268423	4.08E-29	0.420865712	6.89E-20	-0.007426129
0.657624224	1.32E-54	0.482780876	1.72E-26	-0.086343636
0.482085676	2.08E-26	0.465168469	1.79E-24	-0.066547407
0.35593133	2.74E-14	0.205680708	1.72E-05	-0.046907422
0.280845243	3.10E-09	0.091618956	0.057654586	-0.010161609
-0.308117454	6.57E-11	-0.205334037	1.78E-05	-0.010294411
-0.311184101	4.15E-11	-0.140262355	0.003562631	0.09531068
0.263429308	2.94E-08	0.145532517	0.00248499	-0.01920289
0.439901812	8.92E-22	0.222265427	3.26E-06	-0.044341653
0.435106394	2.74E-21	0.198947311	3.25E-05	-0.015876336
0.039407537	0.415009309	-0.098953894	0.040265499	-0.003180753
-0.273926572	7.72E-09	-0.063918337	0.185857403	0.04314182
-0.178468043	0.000199281	-0.157848298	0.001022372	0.014628505
0.251244755	1.29E-07	0.061539773	0.202802131	-0.061332501
0.604864414	2.95E-44	0.452725403	4.07E-23	-0.076200804
0.536782029	1.85E-33	0.312148323	3.59E-11	-0.102707101
0.355563284	2.93E-14	0.180363725	0.000169848	0.020201308
0.4279907	1.40E-20	0.289466712	9.57E-10	0.019864067
0.222237703	3.27E-06	0.119947431	0.012809036	-0.039385906
0.575147034	3.12E-39	0.359194418	1.53E-14	-0.030822196
-0.30310439	1.38E-10	-0.205716617	1.71E-05	-0.025972832
-0.263124809	3.05E-08	-0.095236479	0.048426676	0.189396726
-0.421368047	6.17E-20	-0.223360832	2.90E-06	0.061314146
0.186092412	0.000103763	0.176484936	0.000235138	-0.041544107
0.046630939	0.334710191	-0.157944013	0.001015079	0.060083894
0.128637656	0.007566453	0.067945736	0.159582441	0.030615848
0.596445111	8.84E-43	0.273900034	7.74E-09	-0.155020887
0.307785225	6.90E-11	0.27612765	5.79E-09	0.057740637
0.157947115	0.001014844	-0.038996459	0.419896537	-0.053639475

0.021916603	0.650401935	0.017726879	0.71395383	0.002452614
0.507847956	1.43E-29	0.480527626	3.17E-26	-0.015787016
0.375299754	7.88E-16	0.137097357	0.00439817	-0.039345589
0.097566457	0.04316411	0.040902996	0.397514953	-0.020865953
0.379946094	3.24E-16	0.276493672	5.51E-09	-0.068228897
-0.045034588	0.35153735	-0.035821456	0.458763106	0.054667861
0.369390018	2.39E-15	0.342263703	2.92E-13	-0.118326238
-0.137352088	0.004324894	0.098864114	0.040447956	0.078574991
-0.366301327	4.22E-15	-0.223766627	2.78E-06	0.087666061
0.508672186	1.12E-29	0.374191596	9.72E-16	-0.016541623
0.153687617	0.001390243	0.20595656	1.67E-05	0.039444657
0.340695644	3.80E-13	0.184590894	0.000118239	-0.039536294
-0.240811633	4.34E-07	-0.223010099	3.01E-06	0.001692516
0.248333523	1.82E-07	0.048003271	0.32066508	0.035008189
0.450310495	7.35E-23	0.31235182	3.48E-11	-0.012922739
-0.152063356	0.001564292	-0.067577509	0.161861729	0.084866767
0.482282664	1.97E-26	0.341518052	3.31E-13	-0.143143367
-0.200260872	2.88E-05	-0.127537257	0.008101953	-0.066958368
0.04710093	0.329856233	0.121717771	0.011535472	-0.098434181
-0.430292927	8.28E-21	-0.213354785	8.09E-06	0.080043494
0.048419847	0.316478707	0.046438309	0.336712809	0.023169693
0.60767317	9.28E-45	0.363014893	7.70E-15	-0.097895245
0.453667431	3.23E-23	0.320152005	1.05E-11	0.003739875
0.487494461	4.74E-27	0.231576426	1.21E-06	0.018784277
0.326417957	3.92E-12	0.191669131	6.33E-05	-0.038715321
0.444752295	2.82E-22	0.447402563	1.49E-22	0.004442421
-0.158715713	0.00095801	-0.055214847	0.253246944	0.012630115
0.479841533	3.81E-26	0.330342453	2.09E-12	-0.07626934
-0.172832923	0.000317435	-0.363645016	6.87E-15	0.00101038
0.031708559	0.511969793	0.167733718	0.00047789	0.088813911
0.47101044	3.95E-25	0.227330278	1.91E-06	-0.024847136
0.263905815	2.77E-08	0.05497071	0.255354813	0.02179315
0.030469288	0.528608375	0.011393588	0.813756743	-0.022874642
-0.355928555	2.74E-14	-0.312071455	3.63E-11	0.066001203
-0.43767832	1.50E-21	-0.400505379	5.36E-18	0.008203963
0.083733668	0.082859396	-0.110087139	0.022423664	0.019235727
0.096160104	0.046279603	0.146319976	0.002352384	-0.010460988
0.182072595	0.000146856	0.077143712	0.110174169	-0.137723351
-0.340662991	3.82E-13	-0.143483122	0.002862661	0.201465511
0.222034679	3.34E-06	0.094888634	0.049256607	0.096988698
0.268615009	1.53E-08	0.25819714	5.61E-08	-0.097223638
0.166100422	0.000543477	0.079253859	0.10074727	0.004968441
0.24293051	3.41E-07	0.088665407	0.066228755	0.093474694
-0.208798521	1.27E-05	0.00211213	0.965166959	-0.01712746
0.671625524	1.03E-57	0.466563749	1.25E-24	-0.047833614
-0.297573746	3.06E-10	-0.014981773	0.756726942	0.147089883
-0.109244283	0.023480302	-0.061890049	0.200238702	0.079123552
-0.503817417	4.65E-29	-0.217873507	5.12E-06	0.111364509
0.586657132	4.07E-41	0.392943591	2.51E-17	-0.000315321
0.453358652	3.48E-23	0.236671838	6.90E-07	-0.0663613
-0.248778206	1.73E-07	-0.011434472	0.813100739	-0.000251454

0.281274617	2.92E-09	0.074896226	0.120964229	0.031203661
-0.175844998	0.000247941	-0.131435491	0.006344567	-0.045773907
-0.313364865	2.98E-11	-0.123530141	0.010348959	0.053319007
-0.379991162	3.21E-16	-0.19380888	5.22E-05	0.153747707
0.358306484	1.80E-14	0.106207014	0.027652836	-0.040263373
0.677077367	5.70E-59	0.383923121	1.50E-16	-0.080581354
-0.48939036	2.81E-27	-0.232085835	1.14E-06	0.05917916
0.078021564	0.106171364	-0.007964391	0.869199803	-0.043812777
0.196882254	3.94E-05	-0.010712171	0.824709323	0.028018101
0.373383956	1.13E-15	0.175036509	0.000265047	-0.025102054
0.446715265	1.76E-22	0.22250991	3.17E-06	-0.039564931
0.026318036	0.586270757	-0.026124494	0.589028539	-0.058255581
-0.244988715	2.69E-07	-0.086175094	0.074247518	0.022670022
0.630508093	4.89E-49	0.472123989	2.95E-25	-0.070452446
0.588727019	1.83E-41	0.420929043	6.80E-20	-0.047656405
0.311400482	4.02E-11	0.326010874	4.18E-12	0.028198722
0.570434506	1.75E-38	0.347876549	1.12E-13	-0.056627001
0.567680379	4.75E-38	0.277505748	4.83E-09	0.011077248
-0.406663999	1.48E-18	-0.359339888	1.49E-14	-0.001304276
-0.420830329	6.95E-20	-0.337267315	6.72E-13	-0.01412769
0.150118716	0.001798872	-0.006061419	0.900264257	0.012797374
-0.371065106	1.75E-15	-0.034883866	0.470613486	0.040289027
0.185512525	0.000109144	0.036058763	0.455790391	-0.006457568
0.107449764	0.025874242	0.040379028	0.403593453	0.036379386
0.284640803	1.85E-09	0.109760568	0.022828074	-0.032806656
0.416720622	1.71E-19	0.156780552	0.001107069	-0.047870323
-0.458349777	1.01E-23	-0.231766687	1.18E-06	0.119289316
-0.147964217	0.002096082	-0.017631281	0.715429338	0.12983602
-0.335162954	9.52E-13	-0.214211563	7.42E-06	0.011451693
-0.530294072	1.48E-32	-0.370983447	1.77E-15	0.119527537
-0.135462923	0.004895616	-0.025074719	0.604089626	-0.045882442
0.501318174	9.60E-29	0.28146641	2.85E-09	-0.017910883
-0.343454236	2.38E-13	-0.19227191	6.00E-05	0.103329823
0.528967022	2.26E-32	0.476493148	9.32E-26	-0.054146647
-0.028752307	0.552105568	-0.041572462	0.389829084	0.055422554
0.558014965	1.46E-36	0.355637893	2.89E-14	-0.048185426
0.252097507	1.17E-07	0.167901272	0.000471595	0.17366089
-0.24626125	2.32E-07	-0.090476439	0.060855442	-0.032801672
0.091202813	0.058803909	-0.081501262	0.091423585	0.073145245
0.141532598	0.003269875	0.013674985	0.77736212	0.02676807
-0.299402682	2.35E-10	-0.197997281	3.55E-05	0.059105078
0.52432645	9.70E-32	0.412644677	4.14E-19	-0.047221435
-0.340781009	3.74E-13	-0.11519655	0.016858753	0.091455113
0.50745174	1.61E-29	0.435935832	2.26E-21	-0.084267946
-0.241203845	4.15E-07	-0.194739391	4.79E-05	0.042143626
0.258489295	5.41E-08	0.120673209	0.012272644	0.036864173
-0.179698518	0.000179678	-0.083928636	0.082143277	0.101732212
0.357728128	1.99E-14	0.235389115	7.96E-07	-0.028160616
0.224447689	2.59E-06	0.045241502	0.349326636	-0.044248075
0.427123679	1.70E-20	0.303157084	1.36E-10	0.005050275
-0.21797808	5.07E-06	-0.190382562	7.10E-05	0.023963212

−0. 107952026	0. 025183895	−0. 156927003	0. 001095082	−0. 011827543
0. 592694827	3. 90E−42	0. 42544269	2. 48E−20	−0. 024434417
0. 490500823	2. 06E−27	0. 318655907	1. 33E−11	−0. 084107034
−0. 482414505	1. 90E−26	−0. 334068643	1. 14E−12	0. 042366013
−0. 384235966	1. 41E−16	−0. 201003103	2. 68E−05	0. 091610451
0. 214772736	7. 02E−06	0. 150394577	0. 001763742	−0. 012390503
−0. 490207776	2. 24E−27	−0. 277594583	4. 77E−09	0. 029806776
0. 140010939	0. 003623318	−0. 039571274	0. 413072043	0. 028785528
0. 307834195	6. 85E−11	0. 063283189	0. 190276567	0. 002272899
0. 261264125	3. 85E−08	0. 173718604	0. 000295314	−0. 028582592
0. 294814258	4. 53E−10	0. 034409395	0. 476673988	−0. 059233211
0. 554047352	5. 77E−36	0. 280952031	3. 05E−09	−0. 044711036
−0. 009238452	0. 848510664	0. 04996411	0. 301272839	−0. 04857934
−0. 199738844	3. 02E−05	−0. 019308045	0. 689707599	0. 013114425
0. 284796193	1. 82E−09	0. 203378285	2. 14E−05	−0. 057379134
−0. 269733007	1. 33E−08	−0. 055476794	0. 250998769	0. 103628721
0. 225237715	2. 38E−06	−0. 034105246	0. 48058123	−0. 050443476
−0. 357636162	2. 03E−14	−0. 184518953	0. 000118978	0. 054054196
0. 394182026	1. 95E−17	0. 276959405	5. 19E−09	0. 034757932
0. 518901334	5. 17E−31	0. 304534474	1. 12E−10	−0. 080428211
−0. 344766656	1. 91E−13	−0. 142886041	0. 002982125	0. 118562328
−0. 07123113	0. 14030552	−0. 13567674	0. 004827778	0. 101447365
−0. 565162221	1. 17E−37	−0. 40612505	1. 66E−18	0. 031959831
−0. 286299462	1. 48E−09	−0. 165371462	0. 000575367	−0. 050821158
0. 244318191	2. 91E−07	0. 183264167	0. 000132589	−0. 064325003
0. 424294324	3. 21E−20	0. 211465773	9. 76E−06	−0. 044048734
−0. 238139535	5. 86E−07	−0. 19653643	4. 07E−05	−0. 055790641
0. 281567499	2. 81E−09	0. 160028998	0. 000867651	−0. 047250708
−0. 211322341	9. 90E−06	0. 041771749	0. 387558661	0. 015150798
−0. 318531671	1. 35E−11	−0. 100696769	0. 03685899	0. 150665918
0. 050913226	0. 292171805	0. 00191958	0. 968340727	0. 038664875
−0. 165077072	0. 000588731	0. 054447888	0. 259909623	−0. 086354081
0. 726373901	1. 05E−71	0. 418941259	1. 05E−19	−0. 101053484
0. 238761122	5. 46E−07	0. 000708485	0. 988312448	−0. 045011223
0. 233230597	1. 01E−06	0. 128832298	0. 007475095	0. 018671248
0. 462487153	3. 55E−24	0. 457709534	1. 18E−23	0. 027151441
0. 305762459	9. 31E−11	0. 195544866	4. 45E−05	0. 056169688
0. 286179777	1. 50E−09	0. 137117111	0. 004392448	−0. 000726372
0. 368150398	3. 00E−15	0. 192260278	6. 00E−05	0. 061836619
−0. 185333975	0. 000110853	−0. 164204963	0. 00063003	0. 099696983
0. 523043811	1. 44E−31	0. 3535189	4. 20E−14	−0. 033196571
0. 575306626	2. 94E−39	0. 293317523	5. 59E−10	−0. 053765978
0. 118541847	0. 013906908	−0. 066565144	0. 1682543	−0. 025201924
−0. 089823525	0. 062749587	0. 139325294	0. 003793611	0. 015286775
0. 630663538	4. 56E−49	0. 450143139	7. 66E−23	−0. 076459913
0. 048077544	0. 319916048	0. 122512305	0. 011001251	−0. 060891894
−0. 448918009	1. 03E−22	−0. 303429891	1. 31E−10	0. 162611877
−0. 468263551	8. 07E−25	−0. 253723867	9. 63E−08	0. 073224377
0. 074151676	0. 124715006	−0. 058333826	0. 227377076	−0. 008790221
0. 131341185	0. 006382683	0. 010019832	0. 835872364	0. 000847634
−0. 273731501	7. 91E−09	−0. 09083784	0. 059827453	−0. 041873506

0.414075856	3.04E-19	0.205060626	1.82E-05	-0.039796542
0.724390072	3.87E-71	0.538564143	1.03E-33	-0.09443795
-0.220935561	3.74E-06	-0.124540574	0.009735571	0.017044532
0.174431947	0.000278553	0.34004842	4.23E-13	-0.045828758
-0.171934212	0.000341454	-0.01165971	0.809489016	0.075251866
0.50788452	1.41E-29	0.25395083	9.37E-08	-0.048829061
0.382623359	1.93E-16	0.2433911	3.23E-07	-0.005901852
0.098274062	0.041664559	0.03387383	0.483565727	0.056734593
-0.320910342	9.34E-12	-0.208117056	1.36E-05	0.071636347
0.098189545	0.041841324	-0.102810675	0.033058466	-0.007465
-0.274427074	7.23E-09	-0.099283721	0.039601165	0.088080414
0.27354599	8.11E-09	0.006026989	0.900827852	0.069598144
-0.471931206	3.11E-25	-0.228698073	1.65E-06	0.085457254
-0.26109479	3.93E-08	-0.113351801	0.018710282	0.09400365
-0.226789153	2.02E-06	-0.214736444	7.04E-06	-0.08826921
-0.503052335	5.81E-29	-0.294347271	4.83E-10	0.178160525
0.329509683	2.39E-12	0.102337724	0.03387842	0.01689837
0.268604502	1.53E-08	0.250556977	1.40E-07	-0.019557542
-0.194985445	4.69E-05	-0.009818943	0.839117723	-0.092331622
-0.219022761	4.55E-06	0.002141784	0.96467823	0.089136843
0.467804201	9.09E-25	0.354896906	3.29E-14	-0.0541114
0.276387653	5.59E-09	0.23477097	8.52E-07	-0.004784336
0.025186643	0.60247567	-0.144894953	0.002597307	0.062304024
-0.363780009	6.70E-15	-0.239883195	4.82E-07	0.013827284
-0.163836955	0.00064825	-0.259107238	5.02E-08	0.03845492
0.1495575	0.001872328	0.130722658	0.006637804	-0.062405951
0.276738149	5.34E-09	0.206193328	1.64E-05	-0.007638949
0.169280494	0.000422631	-0.063998405	0.185305729	0.015100663
0.174699062	0.000272508	0.061363566	0.204100638	0.032872252
0.606056939	1.81E-44	0.502605927	6.62E-29	0.003047344
-0.271536157	1.05E-08	-0.115689121	0.016392327	-0.008514361
0.71006303	3.40E-67	0.553307122	7.44E-36	-0.032723741
-0.23546971	7.88E-07	-0.038545852	0.425292234	0.073774813
-0.353737823	4.04E-14	-0.118881633	0.013634184	-0.045786818
-0.118354075	0.014059671	0.068374497	0.156959043	0.09891115
-0.389740853	4.76E-17	-0.145227392	0.002538178	0.090490749
-0.174952335	0.00026689	0.021634122	0.654614284	0.064473484
-0.415831597	2.08E-19	-0.200001686	2.95E-05	0.03470948
0.217562153	5.29E-06	0.000706006	0.988353344	0.166483326
-0.144093045	0.002745138	-0.14595812	0.002412496	-0.029470289
0.290776283	7.98E-10	0.086937641	0.071712216	-0.052495784
-0.263462247	2.93E-08	-0.040987203	0.396543228	0.041650092
-0.369470331	2.35E-15	-0.07591131	0.115992995	0.03494528
-0.214239018	7.40E-06	-0.068251503	0.157708231	0.110868899
-0.117488084	0.014783456	-0.004352095	0.928299375	0.098231703
0.418812684	1.08E-19	0.158763561	0.000954571	0.001680496
-0.33103325	1.87E-12	-0.16731996	0.000493771	0.115678158
-0.352997042	4.60E-14	-0.239338755	5.12E-07	0.048106165
-0.025485	0.598182814	-0.133867117	0.00542967	0.051801246
-0.399366377	6.78E-18	-0.245888857	2.42E-07	0.068399336
0.345229086	1.76E-13	0.323823375	5.91E-12	0.084851478

0.424223605	3.27E-20	0.208685117	1.28E-05	0.018693431
0.452233132	4.59E-23	0.210845275	1.04E-05	-0.03154
-0.495666935	4.83E-28	-0.368356843	2.89E-15	0.115477458
0.278579882	4.19E-09	0.145266227	0.002531351	0.009434428
-0.013133777	0.785955179	-0.143453737	0.002868437	0.026156566
0.120225338	0.012601252	0.030241838	0.531691678	-0.034518761
0.525440813	6.85E-32	0.205822175	1.69E-05	-0.043864717
0.540160508	6.14E-34	0.247449697	2.02E-07	-0.04685545
-0.165438697	0.000572355	-0.087608224	0.069541496	-0.063333896
0.115072516	0.016978018	-0.03396411	0.482400223	0.014807078
-0.216375874	5.97E-06	-0.071512863	0.138739393	0.080809892
-0.251793933	1.21E-07	-0.076344434	0.113921156	0.024558251
-0.045051014	0.351361531	0.052280783	0.27938517	-0.020408658
0.420649403	7.23E-20	0.291858607	6.86E-10	-0.0578705
0.220776268	3.80E-06	-0.004085943	0.932673504	-0.098355862
0.289485779	9.54E-10	-0.015548428	0.747831533	-0.111512761
0.543632536	1.95E-34	0.348852082	9.47E-14	0.004368058
-0.063529331	0.188554921	-0.051398802	0.287587548	0.089911161
0.114686127	0.01735429	0.052419621	0.278108566	0.01170237
-0.420216945	7.95E-20	-0.230729328	1.32E-06	0.007158637
0.376105164	6.76E-16	0.190793021	6.85E-05	0.059977383
0.278076716	4.47E-09	0.036102687	0.455241348	-0.040293618
-0.412803028	4.01E-19	-0.231822803	1.18E-06	0.002188626
0.207827162	1.40E-05	-0.006621261	0.89110729	0.008746977
-0.418050264	1.28E-19	-0.161819059	0.000757136	0.176780331
0.646048312	3.69E-52	0.41932611	9.68E-20	-0.062500032
0.326871741	3.65E-12	0.133077011	0.005713017	-0.140182763
0.321610786	8.37E-12	0.242330081	3.65E-07	0.013290583
-0.456588469	1.56E-23	-0.237616155	6.21E-07	0.226313815
0.31282156	3.24E-11	0.131273369	0.006410219	-0.056170629
0.601722776	1.06E-43	0.526481113	4.94E-32	-0.024911501
0.179505894	0.000182623	0.027275283	0.572719504	0.018112561
-0.446122301	2.03E-22	-0.276741141	5.34E-09	0.162090922
0.442136851	5.26E-22	0.251113169	1.31E-07	-0.102869379
0.741406509	3.70E-76	0.430865163	7.27E-21	-0.086427132
0.221300532	3.60E-06	0.078025285	0.106154643	0.01682418
-0.273441493	8.22E-09	-0.240936245	4.27E-07	0.049316119
-0.262080453	3.48E-08	-0.023822021	0.622283985	0.084770067
0.470730632	4.25E-25	0.384816183	1.26E-16	-0.029522343
-0.231734053	1.19E-06	-0.264822181	2.47E-08	0.045598556
0.481771128	2.26E-26	0.381784359	2.27E-16	-0.023795163
-0.196113845	4.23E-05	-0.164175874	0.000631453	0.001886638
0.253655452	9.71E-08	0.183677847	0.000127948	-0.003454698
0.513780337	2.44E-30	0.297407386	3.13E-10	-0.000165267
0.089633091	0.06331111	0.05348758	0.268421118	0.000284098
0.445911844	2.13E-22	0.176460853	0.000235609	-0.04714157
-0.481229314	2.62E-26	-0.338596378	5.39E-13	0.004530873
0.275478332	6.30E-09	0.227442373	1.89E-06	-0.034597654
0.35184028	5.63E-14	0.249227735	1.64E-07	-0.047023103
0.568827535	3.14E-38	0.35153282	5.94E-14	-0.06241857
-0.13079326	0.006608228	-0.064841061	0.179572775	-0.042949295

0.028117661	0.560918342	-0.006626412	0.891023105	-0.003601699
0.388699838	5.85E-17	0.331228803	1.81E-12	0.064081743
-0.225282561	2.37E-06	0.035080924	0.468108941	0.115105257
0.112259698	0.019887954	0.034523369	0.475214307	0.068604155
-0.204407819	1.94E-05	-0.023052762	0.633572838	0.03714789
-0.657553335	1.37E-54	-0.390624384	3.99E-17	0.138144532
0.658514202	8.47E-55	0.37332886	1.14E-15	-0.087689052
-0.118025999	0.014330122	0.079763245	0.098570446	0.028462856
0.272618374	9.14E-09	0.149336568	0.00190199	0.002901994
-0.065171027	0.177364034	-0.090751673	0.060071238	0.070210387
0.12779923	0.007971488	0.036110984	0.455137674	0.000343141
-0.036234773	0.453592527	-0.167857317	0.000473239	-0.010797396
-0.386931452	8.31E-17	-0.140813237	0.003432868	0.105901172
-0.286704807	1.40E-09	-0.183212654	0.000133178	0.09377449
0.412972284	3.86E-19	0.142251073	0.003114127	-0.022955827
0.636547289	3.14E-50	0.608678783	6.12E-45	-0.043214075
0.263757337	2.82E-08	0.109549046	0.023093371	0.017517733
0.50559698	2.77E-29	0.369630817	2.28E-15	-0.064952459
0.494947634	5.92E-28	0.30903384	5.73E-11	-0.025014114
0.48147753	2.45E-26	0.24621989	2.33E-07	-0.049252551
-0.443937867	3.42E-22	-0.230593691	1.34E-06	0.075325797
0.188150273	8.66E-05	0.108175502	0.024881876	0.010781148
0.618718553	8.75E-47	0.272171656	9.69E-09	-0.041083143
-0.264497366	2.57E-08	-0.108168622	0.024891127	0.100358481
0.08356465	0.083484273	0.004374708	0.927927836	-0.030098787
0.182588517	0.000140511	-0.095421222	0.047990674	-0.008638745
0.073449821	0.128332869	0.071924188	0.136477021	0.08729238
-0.240623	4.43E-07	-0.043192513	0.37160613	0.134175664
0.267335733	1.80E-08	0.045557762	0.34596468	0.041048776
-0.162582029	0.000714117	0.102384586	0.033796409	0.037537973
0.221075163	3.69E-06	0.031526756	0.514393539	-0.025821033
0.208278162	1.34E-05	0.069217896	0.151893916	-0.031972443
0.710179691	3.16E-67	0.370323592	2.01E-15	-0.075593664
0.169334982	0.000420798	0.06284751	0.193352155	0.038253595
-0.15068732	0.00172715	-0.028984986	0.548891616	-0.045075473
0.478930049	4.86E-26	0.341882423	3.11E-13	0.053738623
0.339798449	4.41E-13	0.105008638	0.029466433	0.042536447
-0.221501925	3.53E-06	-0.016245736	0.736930966	-0.00217322
0.312092197	3.62E-11	0.053018384	0.272648371	-0.003262605
0.124606524	0.009696682	-0.008890094	0.854157851	0.004455828
0.548136926	4.32E-35	0.280652565	3.18E-09	-0.032983608
-0.428770189	1.17E-20	-0.361508542	1.01E-14	-0.026783021
0.616434282	2.33E-46	0.417257837	1.52E-19	-0.072344151
-0.26339511	2.95E-08	-0.13845953	0.00401898	0.017121295
0.248117497	1.87E-07	0.068923682	0.15364657	-0.108215581
-0.086465622	0.073273098	0.027636942	0.567638525	0.084985745
-0.218796249	4.66E-06	-0.194471408	4.91E-05	-0.030429251
0.360198668	1.28E-14	0.138363956	0.004044588	-0.016624444
0.267710992	1.72E-08	0.322294956	7.52E-12	-0.068028494
0.113106123	0.018969774	0.001221951	0.979843466	-0.020112727
-0.005974751	0.901683044	-0.011639388	0.809814716	0.049506171

0.56962335	2.36E-38	0.331695043	1.68E-12	-0.056644307
-0.193819172	5.21E-05	-0.034760907	0.472179984	0.048406217
0.480295175	3.37E-26	0.348832126	9.50E-14	-0.020095706
0.479883576	3.76E-26	0.374588589	9.01E-16	-0.01996346
-0.326898999	3.63E-12	-0.080089655	0.09719534	0.077366406
0.389386326	5.11E-17	0.238097573	5.89E-07	-0.003064655
0.291153378	7.57E-10	0.137067928	0.004406708	-0.023270174
-0.015529308	0.748131139	-0.098784267	0.040610812	-0.030225805
0.061064755	0.206316401	0.119859902	0.012875101	-0.082396395
0.366407096	4.14E-15	0.238970798	5.34E-07	0.041901882
-0.144999988	0.002578491	0.032475469	0.501811271	0.060904161
-0.062062853	0.198982791	0.109939218	0.022606067	0.150311719
0.585973478	5.29E-41	0.33682745	7.23E-13	-0.059825355
0.339820723	4.39E-13	0.204021909	2.01E-05	-0.096320902
0.452323843	4.49E-23	0.318889095	1.28E-11	0.024080948
-0.224503574	2.58E-06	0.057353684	0.235296063	0.017777875
-0.483875076	1.28E-26	-0.27032822	1.23E-08	0.156436799
0.57312245	6.57E-39	0.285720339	1.60E-09	-0.065232616
0.116173978	0.015944279	0.170304098	0.000389397	-0.003225177
0.27258993	9.18E-09	0.200377049	2.84E-05	0.074803107
0.253735488	9.62E-08	0.175593059	0.000253158	-0.007501062
-0.145381653	0.002511159	-0.029715101	0.538866794	0.053109798
0.521299257	2.48E-31	0.410262339	6.91E-19	-0.044251617
0.515705693	1.37E-30	0.311434663	4.00E-11	-0.050774616
0.031815032	0.510553075	0.001880038	0.96899256	-0.04693765
0.186766821	9.78E-05	0.021384516	0.65834555	0.032793147
-0.169583674	0.000412522	-0.090209256	0.06162477	-0.043615518
-0.244423153	2.87E-07	-0.116959388	0.015241246	0.026347669
0.636508452	3.20E-50	0.371813789	1.52E-15	-0.063883038
0.469383404	6.04E-25	0.23149037	1.22E-06	-0.063831405
0.434789802	2.94E-21	0.246128527	2.36E-07	-0.022510406
0.315606748	2.12E-11	0.188741765	8.22E-05	0.023703638
0.216426204	5.94E-06	0.082847756	0.086177099	0.039668475
0.205278225	1.79E-05	0.120437645	0.012444524	0.001925782
0.44945731	9.05E-23	0.212520652	8.79E-06	-0.063062799
-0.146670208	0.002295508	0.085749151	0.075695162	-0.005211037
0.40764983	1.20E-18	0.329135572	2.54E-12	-0.030954815
-0.462372787	3.65E-24	-0.286862578	1.37E-09	0.054519385
-0.198657786	3.34E-05	-0.04350991	0.368098605	-0.022929517
0.412068544	4.69E-19	0.141231726	0.003337172	-0.034052584
0.172415996	0.000328375	0.096204816	0.046177724	0.032883649
-0.52054084	3.13E-31	-0.438371951	1.28E-21	0.078695089
-0.201771961	2.49E-05	-0.181404991	0.000155467	0.109505846
0.231897016	1.17E-06	0.142783252	0.003003142	0.017408566
0.098525658	0.041142079	-0.061413532	0.203731817	-0.007895547
0.105142688	0.029258626	-0.063145126	0.191247284	0.09281829
0.312678985	3.31E-11	0.453147712	3.67E-23	0.016006058
-0.290072732	8.80E-10	-0.124627845	0.009684139	0.073457807
-0.407380958	1.27E-18	-0.308916045	5.83E-11	0.026644212
-0.493011299	1.02E-27	-0.245623529	2.50E-07	0.025477404
-0.218843107	4.64E-06	0.051545041	0.286216453	-0.058194134

0.312354822	3.48E-11	0.268597008	1.53E-08	-0.061347005
0.34085516	3.70E-13	0.206063318	1.66E-05	-0.043604332
0.439797334	9.14E-22	0.317208465	1.66E-11	-0.105587263
-0.162492765	0.00071903	-0.029831255	0.537280413	0.049367889
0.323938477	5.81E-12	0.184148108	0.000122858	-4.15E-05
0.858959089	1.72E-126	0.566153424	8.23E-38	-0.077970305
-0.306018332	8.97E-11	-0.068214514	0.157934074	0.077701168
-0.087406538	0.070188638	-0.036939437	0.44485321	0.069871856
-0.105231063	0.029122313	-0.045097883	0.350860155	0.153738046
-0.236350859	7.15E-07	-0.1666837	0.000519151	0.097132557
0.701654973	5.43E-65	0.556418083	2.55E-36	-0.053999825
-0.235905558	7.51E-07	-0.212665015	8.66E-06	-0.109301806
0.487771629	4.39E-27	0.374653512	8.90E-16	-0.028270983
0.36661764	3.99E-15	0.09076381	0.06003685	0.003830063
0.30022735	2.09E-10	0.12557299	0.009142401	-0.010814039
0.403333388	2.98E-18	0.105133632	0.029272624	-0.016291634
0.354268161	3.68E-14	0.211386149	9.84E-06	0.032918441
0.114407745	0.017629881	-0.00280855	0.953692938	0.002222186
0.164699285	0.000606303	0.232255588	1.12E-06	0.029069216
0.104616452	0.030081688	-0.044163487	0.360940948	0.074322178
-0.020179153	0.676482183	0.040342484	0.404019463	-0.022790354
-0.138350126	0.004048305	0.074911752	0.120886963	0.036569451
-0.432307558	5.22E-21	-0.24896975	1.69E-07	0.074963473
-0.182203945	0.000145216	-0.143784075	0.002804112	-0.059126692
0.357230577	2.18E-14	0.062579779	0.195260117	-0.022877443
0.535326593	2.96E-33	0.33892683	5.10E-13	-0.054840297
0.072897703	0.131235589	-0.067205219	0.164190969	0.044328794
-0.126023596	0.008893744	-0.030654935	0.526098499	0.154688929
0.218753977	4.68E-06	0.259943747	4.53E-08	-0.013869049
-0.213368054	8.08E-06	-0.149275606	0.00191025	0.003994981
0.121488636	0.011693738	0.028870931	0.550465887	0.060864039
0.133757192	0.005468319	0.013925807	0.773388783	0.015129082
-0.223731509	2.79E-06	-0.143132036	0.002932368	0.122859831
0.446397626	1.90E-22	0.200268583	2.87E-05	0.032997253
-0.188456386	8.43E-05	-0.07939012	0.100161263	0.057966731
-0.133042033	0.005725862	0.056037164	0.246236058	0.120399866
0.037713514	0.435364286	0.220938797	3.74E-06	0.004522216
-0.142690673	0.003022187	0.018964595	0.694948309	0.007675608
0.137184476	0.004372984	0.039961596	0.408475509	-0.014814548
-0.183151382	0.000133882	-0.067712412	0.161023868	-0.060464587
-0.028156899	0.560371525	0.202040655	2.43E-05	0.043095463
0.111884683	0.020306967	0.155242057	0.001240516	0.062498427
0.187919556	8.84E-05	0.119934132	0.012819054	-0.053744844
-0.450514959	6.99E-23	-0.362317793	8.74E-15	0.08163651
0.115806998	0.016282397	0.017412275	0.718813562	0.010556679
0.08016401	0.096884246	0.109931495	0.022615626	0.111483809
0.188871066	8.13E-05	0.063799515	0.186678329	0.074699333
0.435999191	2.22E-21	0.354738411	3.39E-14	-0.105370355
-0.384949404	1.23E-16	-0.189887527	7.43E-05	0.181694337
0.578792379	8.04E-40	0.438677074	1.19E-21	-0.019943405
0.034452517	0.476121435	-0.085046235	0.07813428	0.016642405

-0.366428173	4.13E-15	-0.247528423	2.00E-07	-0.031197255
-0.27472837	6.95E-09	-0.099987284	0.038214985	0.1536142
0.300629223	1.97E-10	0.134722748	0.005137139	-0.007626222
-0.23034188	1.38E-06	-0.082571829	0.087232003	-0.085911084
0.320815988	9.48E-12	0.292685509	6.11E-10	0.019397628
-0.327415049	3.34E-12	-0.003455492	0.943042601	-0.00121406
-0.10151407	0.035347763	-0.147294373	0.002197247	0.072352066
0.028783645	0.551672164	-0.089735599	0.063008339	-0.070703538
0.249335757	1.62E-07	0.105162384	0.029228199	-0.100972466
0.178493098	0.000198863	0.027547258	0.5688965	0.003553331
0.15281164	0.001481765	0.097655115	0.042973772	-0.030587544
-0.038170758	0.429814325	0.044545466	0.356798261	-0.048371202
-0.009076867	0.851129151	0.167869631	0.000472778	-0.084762627
-0.088187307	0.067710502	0.008525276	0.860079868	-0.020860835
0.341999666	3.05E-13	0.15216161	0.00155322	-0.02549419
0.569702135	2.29E-38	0.362099933	9.09E-15	-0.031566689
-0.102120163	0.034261384	0.098956641	0.040259927	-0.028432066
-0.500420642	1.24E-28	-0.328695952	2.72E-12	0.062896888
-0.264231441	2.66E-08	-0.19022452	7.21E-05	0.020150309
-0.394386853	1.87E-17	-0.263523713	2.91E-08	0.088385689
0.495320323	5.33E-28	0.44726665	1.54E-22	-0.046600132
-0.068037858	0.159016018	0.024757765	0.608670601	-0.020939864
0.568212932	3.92E-38	0.286414036	1.46E-09	-0.012601249
0.278915021	4.00E-09	0.191136794	6.64E-05	-0.033534405
-0.192844076	5.70E-05	-0.031184004	0.518979141	0.012213278
-0.157482727	0.001050673	-0.024483024	0.612653875	0.022135014
-0.18166133	0.000152106	0.033667051	0.486240942	0.069947855
0.1183522	0.014061203	0.048487562	0.315801601	-0.043318623
0.351755089	5.72E-14	0.071270081	0.140088194	-0.011118609
-0.335136553	9.56E-13	-0.179321128	0.00018549	-0.016201125
-0.081708107	0.090601389	-0.053426529	0.268968613	0.007565966
0.356382769	2.53E-14	0.173238411	0.000307123	0.031751424
0.086919984	0.071770111	0.027329174	0.571961018	0.119108509
0.167343266	0.000492863	0.046582614	0.335211867	-0.040650187
0.26654575	1.99E-08	0.048558956	0.315088725	0.018157856
-0.259066693	5.04E-08	-0.070044995	0.147048086	0.095247557
-0.056082057	0.245857253	-0.010348645	0.830566435	0.00244062
0.293027077	5.82E-10	0.160475799	0.000838751	0.142446209
-0.296067916	3.79E-10	-0.118379084	0.01403924	0.005396254
0.469725568	5.52E-25	0.34075974	3.75E-13	-0.060339177
0.253266172	1.02E-07	0.059026355	0.221897185	-0.012837356
0.556836072	2.20E-36	0.308900033	5.84E-11	-0.066760347
0.110768726	0.021599567	-0.070220105	0.146037424	0.034800329
-0.480492335	3.20E-26	-0.244678076	2.79E-07	0.165742161
-0.141179617	0.003348955	-0.054547262	0.259039592	0.088540787
-0.299989192	2.16E-10	-0.219853839	4.18E-06	-0.007870184
0.162706694	0.000707306	0.036289482	0.452910576	-0.001402237
-0.636710368	2.92E-50	-0.397502847	9.94E-18	0.074204387
-0.224436027	2.59E-06	-0.116139396	0.015975876	0.016887115
0.047009786	0.330793979	0.041360683	0.392250638	-0.021293146
0.362397088	8.61E-15	0.239078093	5.27E-07	-0.035890231

0.406428316	1.56E-18	0.278749479	4.09E-09	-0.059806885
0.462676979	3.38E-24	0.230863377	1.31E-06	-0.071039321
0.599427073	2.68E-43	0.321290765	8.80E-12	-0.033861638
-0.39651266	1.22E-17	-0.230946541	1.29E-06	0.102497188
-0.016400312	0.734521609	0.004098771	0.932462622	-0.10525957
-0.142574695	0.003046201	-0.082586519	0.087175578	-0.016459851
0.132195609	0.006044726	-0.019500248	0.686781219	0.03525795
-0.442768722	4.52E-22	-0.309770673	5.13E-11	0.154844931
0.116198314	0.015922075	0.087994227	0.068316556	0.044437948
0.217756877	5.19E-06	0.110787255	0.021577536	-0.044967335
0.039578764	0.412983559	-0.099300979	0.03956666	-0.014280966
-0.566581508	7.06E-38	-0.357111096	2.22E-14	0.081740645
0.08694115	0.071700714	0.096678386	0.045110178	-0.053565111
0.653763656	8.88E-54	0.450754413	6.60E-23	-0.095726292
-0.286301118	1.48E-09	-0.173277794	0.000306138	0.091640187
0.360525878	1.21E-14	0.207067077	1.50E-05	-0.019648618
0.53467772	3.65E-33	0.28139326	2.88E-09	0.029689832
0.528003835	3.06E-32	0.424159354	3.31E-20	-0.077956774
0.140213993	0.003574233	0.158148167	0.000999684	-0.027030128
0.291404226	7.31E-10	0.058917109	0.222755312	0.02222293
0.290899273	7.84E-10	0.215017206	6.85E-06	0.043892297
0.268918288	1.47E-08	0.237364421	6.39E-07	0.055888129
-0.100126272	0.037946066	0.095807031	0.047090738	-0.040754014
-0.170045926	0.000397542	0.062103657	0.198687075	0.050371554
0.160477801	0.000838623	-0.005688711	0.906367812	-0.017062862
-0.212760578	8.58E-06	-0.247336014	2.05E-07	0.059537552
-0.370269705	2.03E-15	-0.194600379	4.86E-05	0.19637193
-0.414884605	2.55E-19	-0.187382465	9.27E-05	0.103514094
0.438533089	1.23E-21	0.358655453	1.69E-14	0.00415689
0.428838058	1.15E-20	0.156040911	0.001169481	0.015812821
0.152962702	0.001465597	-0.04075277	0.399252066	-0.064483868
-0.114555151	0.01748348	-0.053246282	0.270589474	0.004477532
-0.322041721	7.83E-12	-0.2260428	2.19E-06	0.098515645
0.601770804	1.04E-43	0.461467255	4.60E-24	-0.058669481
0.004749503	0.921772166	0.012820401	0.790942794	0.108775021
0.28240119	2.51E-09	0.162260441	0.000731966	0.004522642
0.651146665	3.18E-53	0.423078633	4.22E-20	-0.040138865
0.084597918	0.079722971	0.019578738	0.685587508	0.032139928
0.457368976	1.29E-23	0.364413982	5.97E-15	-0.018003534
0.062265037	0.19752064	-0.045089057	0.350954533	0.090858938
0.597561679	5.67E-43	0.314215768	2.62E-11	-0.07525914
-0.237578305	6.24E-07	-0.147797581	0.002120843	0.021346499
-0.18427144	0.000121555	-0.069923825	0.147750542	0.068589754
-0.043562597	0.367518362	0.094509961	0.050173576	-0.075739717
0.206515938	1.58E-05	0.128704257	0.007535082	-0.024160054
0.377162668	5.53E-16	0.22566207	2.28E-06	-0.034378486
0.232875782	1.05E-06	0.054214526	0.261960654	0.069184983
-0.126799143	0.008479843	0.214626069	7.12E-06	0.080392383
-0.205678274	1.72E-05	-0.105516528	0.028685718	0.061942199
-0.319725449	1.12E-11	-0.039893418	0.4092762	0.081940688
-0.073118217	0.13007023	0.064412125	0.182474376	0.073170071

-0.141239577	0.0033354	-0.02615989	0.588523736	0.032979865
-0.32617405	4.08E-12	-0.20776614	1.40E-05	0.11253138
-0.098335226	0.041537031	0.08893311	0.065410775	-0.044434538
0.056683623	0.240820522	-0.05914455	0.220971419	-0.023611514
0.260162666	4.41E-08	0.202461647	2.34E-05	0.007795081
0.42384503	3.55E-20	0.235844674	7.56E-07	0.004138522
0.359463385	1.46E-14	0.102233166	0.034062011	0.012592435
0.137992888	0.004145414	0.170903856	0.000371072	-0.05370579
-0.01555406	0.747743276	0.005117489	0.91573286	0.067935529
0.420601167	7.31E-20	0.202846081	2.25E-05	-0.058112436
-0.033487647	0.488568431	-0.187552156	9.13E-05	-0.012652635
-0.041522231	0.390402623	-0.103275724	0.03226878	0.001682663
0.08264913	0.086935427	0.022288317	0.644875884	0.1934332
-0.422190731	5.14E-20	-0.152029283	0.001568148	0.019159058
0.348945844	9.31E-14	0.289221571	9.90E-10	-0.050916346
-0.345649952	1.64E-13	-0.159147268	0.000927401	0.076094801
0.418049793	1.28E-19	0.232662037	1.07E-06	0.004686708
-0.12645775	0.008659878	0.023295943	0.629994803	-0.04377181
-0.425043885	2.72E-20	-0.116549645	0.015604544	-0.015222324
0.33861766	5.37E-13	0.111202523	0.021088863	0.016579767
-0.268356457	1.58E-08	-0.161379984	0.000782968	-0.002355888
0.29902894	2.48E-10	0.200375839	2.85E-05	0.010652975
0.408753383	9.52E-19	0.251890437	1.20E-07	-0.008685185
0.249692736	1.55E-07	0.230608249	1.34E-06	0.004672483
0.376810852	5.91E-16	0.270865896	1.15E-08	-0.030478309
0.324799177	5.07E-12	0.115597922	0.016477824	0.015495866
0.571089554	1.38E-38	0.370637108	1.89E-15	-0.060822434
-0.353414898	4.28E-14	-0.138992242	0.003878918	0.113832039
-0.195877857	4.32E-05	-0.049966328	0.301251355	0.074765528
-0.283096515	2.29E-09	-0.153948408	0.001364016	0.040142619
-0.352767045	4.79E-14	-0.222081549	3.32E-06	0.092918575
0.173799693	0.000293363	0.176855942	0.000228002	-0.061416657
0.441478744	6.14E-22	0.286706632	1.40E-09	-0.043444705
-0.086730363	0.072394269	0.000225992	0.996271795	-0.092961404
-0.462231924	3.79E-24	-0.252675506	1.09E-07	0.11579873
-0.415607134	2.18E-19	-0.239151845	5.23E-07	-0.019218514
0.558939033	1.06E-36	0.313751994	2.81E-11	-0.086549836
0.370282466	2.02E-15	0.168221237	0.000459787	-0.005131796
-0.220536429	3.90E-06	-0.068823592	0.154246296	0.009372186
-0.376235115	6.60E-16	-0.220741814	3.82E-06	0.033924203
0.171070851	0.000366115	0.226576295	2.07E-06	-0.014684427
-0.001274184	0.978982053	0.172060139	0.00033799	0.030497616
-0.35312307	4.50E-14	-0.166964071	0.000507821	0.078218056
0.497582344	2.80E-28	0.331854639	1.63E-12	-0.126497504
0.137042433	0.004414116	0.123182417	0.010567839	0.066269703
0.527667664	3.41E-32	0.28568282	1.61E-09	-0.077534069
0.396418123	1.24E-17	0.120376151	0.012489742	-0.012893051
-0.229773406	1.47E-06	-0.168626114	0.000445241	-0.033012153
-0.206954534	1.52E-05	0.033689108	0.485955193	0.142437858
-0.223221991	2.95E-06	-0.062879574	0.193124577	0.00694727
0.456195381	1.73E-23	0.28115868	2.97E-09	-0.006829711

0.277179095	5.04E-09	0.192359426	5.95E-05	-0.026623861
0.118675163	0.013799337	0.082597718	0.087132586	0.004101518
-0.276799779	5.30E-09	-0.257445549	6.15E-08	0.060209251
0.111823639	0.020375894	-0.026543858	0.583060581	0.020225083
-0.103235571	0.032336322	0.009049144	0.851578574	0.00248386
-0.366699904	3.93E-15	-0.188077132	8.72E-05	0.146741857
0.302415958	1.52E-10	0.206728446	1.55E-05	0.008176484
0.078637134	0.103433697	-0.011738524	0.808226172	-0.014667607
0.292283612	6.46E-10	0.228902363	1.61E-06	0.036347947
0.182519827	0.000141341	0.109923529	0.022625488	0.11505534
-0.063557525	0.188358455	0.134760641	0.005124519	0.045827799
-0.113311239	0.01875291	-0.056585841	0.241634256	-0.090082685
-0.082678669	0.086822314	-0.197327183	3.78E-05	0.060695951
-0.419788791	8.74E-20	-0.202900004	2.24E-05	0.032573836
0.615866972	2.97E-46	0.46613585	1.40E-24	0.007522578
-0.272344282	9.48E-09	-0.02931247	0.544383772	0.038618149
0.008292282	0.863866142	0.067081199	0.164972466	-0.046061444
0.359694446	1.40E-14	0.076060764	0.115274777	-0.027339384
-0.268345995	1.58E-08	-0.08954734	0.063565307	0.127392241
0.177522468	0.000215687	0.100491024	0.037247949	0.014554017
0.057630988	0.233036131	0.023789846	0.622754409	0.065214226
-0.057214264	0.236438109	0.032878834	0.496511248	-0.02784867
0.076176497	0.114721006	0.118944809	0.013583998	-0.009078268
0.212293756	8.99E-06	0.165610849	0.000564708	0.216297209
-0.006867963	0.887076662	-0.101374658	0.035601752	0.022421743
-0.21116089	1.01E-05	-0.070525384	0.144288162	-0.049159675
0.146829951	0.002269985	0.055260864	0.252850984	-0.024183128
0.491690817	1.48E-27	0.348172771	1.06E-13	-0.179455759
0.341468892	3.33E-13	0.127733164	0.008004212	-0.028792582
-0.128771641	0.007503459	-0.136631834	0.004534986	0.018551727
0.471887704	3.14E-25	0.349755063	8.10E-14	0.007983161
0.122886889	0.010757078	-0.020003561	0.679140246	0.036801446
0.473763707	1.92E-25	0.168501453	0.000449673	-0.033575234
-0.158251236	0.000991994	-0.145707643	0.002454923	0.021195896
-0.032567737	0.500596279	0.118578851	0.013876976	0.028620087
0.378653813	4.16E-16	0.1668929	0.000510675	-0.003369429
0.414721727	2.65E-19	0.29413782	4.98E-10	0.012162407
0.44275025	4.54E-22	0.435808925	2.32E-21	0.053457916
0.246187863	2.34E-07	0.077750933	0.107392934	0.008671485
0.268990942	1.46E-08	0.18563771	0.000107961	0.000264574
0.301082161	1.85E-10	0.09285967	0.054337893	-0.012633332
0.412980376	3.86E-19	0.357551082	2.06E-14	-0.048309808
-0.235355187	7.99E-07	-0.145395634	0.002508724	0.070126627
0.154791084	0.00128235	0.207853579	1.39E-05	-0.032015444
0.655715856	3.40E-54	0.383861688	1.52E-16	-0.063564689
0.303565747	1.29E-10	0.158696648	0.000959384	0.049226765
-0.230592269	1.34E-06	-0.057994867	0.23009397	0.095083794
0.390865544	3.80E-17	0.219966859	4.13E-06	0.067727097
-0.155240176	0.001240688	-0.071708859	0.137657808	0.171314083
0.465161439	1.79E-24	0.368347243	2.90E-15	-0.043784666
-0.212129553	9.14E-06	0.051481772	0.286809097	0.116309689

0.208024586	1.37E-05	0.019083547	0.693131555	0.073896866
-0.436559941	1.95E-21	-0.242335594	3.65E-07	0.030769771
-0.455821242	1.89E-23	-0.329382954	2.44E-12	0.118327989
0.088240029	0.067545784	0.249993263	1.50E-07	0.125848151
0.24631893	2.31E-07	0.152268172	0.001541293	0.01681775
-0.187292513	9.34E-05	-0.007701017	0.873488289	-0.005506776
-0.027205696	0.573699599	0.089292701	0.064325107	-0.065705381
-0.14925578	0.001912944	-0.13379891	0.005453622	-0.098024822
0.510006715	7.55E-30	0.381336317	2.48E-16	-0.035424388
0.020894363	0.665697299	0.032164189	0.505921638	0.085198397
0.492038932	1.34E-27	0.227669049	1.84E-06	-0.032506565
0.170693642	0.000377402	-0.027412176	0.570793754	0.013105032
0.554527254	4.89E-36	0.346887456	1.33E-13	-0.032456723
0.604329948	3.67E-44	0.427972626	1.40E-20	-0.081979248
-0.246605296	2.23E-07	-0.160049072	0.000866333	0.104127793
0.646242506	3.36E-52	0.404032815	2.58E-18	-0.055852477
0.230278126	1.39E-06	0.071683795	0.137795759	0.05914081
0.443604219	3.71E-22	0.161770689	0.000759943	-0.029274026
-0.039205383	0.41740848	0.123227846	0.010539012	-0.069136927
0.233956842	9.32E-07	0.114195302	0.017842758	-0.001860777
-0.003267526	0.946136057	-0.069957099	0.147557391	0.129849086
-0.348890766	9.40E-14	-0.046048264	0.340791259	-0.006925249
0.483946813	1.25E-26	0.308666725	6.05E-11	-0.074722082
0.387167287	7.93E-17	0.217424684	5.36E-06	0.071113149
-0.23663653	6.93E-07	-0.142198147	0.003125366	0.083403757
0.491133475	1.73E-27	0.259250842	4.93E-08	0.012097345
0.498230735	2.33E-28	0.258755095	5.24E-08	-0.044454507
-0.511478339	4.87E-30	-0.31107828	4.22E-11	0.097471298
-0.263071334	3.07E-08	-0.064962874	0.178755015	-0.004019291
0.412525347	4.25E-19	0.309002907	5.76E-11	-0.034599951
0.588835337	1.76E-41	0.346396745	1.44E-13	-0.087664772
-0.37241719	1.36E-15	-0.260275586	4.35E-08	0.003020321
-0.018318259	0.704850061	0.038416178	0.426852425	0.010384996
-0.403896784	2.65E-18	-0.260261692	4.35E-08	0.02773945
-0.203900361	2.04E-05	0.102673426	0.033294653	0.045625072
0.19404998	5.11E-05	0.126256345	0.008767679	-0.024908103
-0.092624929	0.054952944	-0.005267495	0.913272361	-0.017917124
0.01450699	0.76420502	-0.011453358	0.812797739	0.0022726
-0.20490473	1.85E-05	-0.011512784	0.811844541	0.078976763
0.383886537	1.51E-16	0.147056146	0.002234288	0.008454019
-0.309728867	5.16E-11	-0.197469688	3.73E-05	0.06733233
-0.270450803	1.21E-08	-0.099051035	0.040068868	0.066506476
-0.141308325	0.003319921	-0.096722575	0.045011632	0.054962201
0.604535501	3.38E-44	0.492426836	1.20E-27	-0.074262103
0.239993009	4.76E-07	0.107526885	0.025767193	0.002551734
-0.295308069	4.22E-10	-0.172603712	0.000323407	0.042823625
0.260814065	4.07E-08	0.119266222	0.013331188	-0.031534005
0.10153382	0.035311907	0.050051354	0.300428494	-0.064954717
0.263835115	2.80E-08	0.185237573	0.000111786	0.047872428
0.281547915	2.82E-09	0.088468184	0.066836738	-0.009522917
0.542103203	3.24E-34	0.27821597	4.39E-09	-0.043937467

0.390001988	4.52E-17	0.259716103	4.66E-08	0.026918918
0.332256912	1.53E-12	0.493318487	9.37E-28	0.055938842
-0.31186322	3.75E-11	-0.137845303	0.004186143	0.074474975
0.498595213	2.10E-28	0.445199789	2.53E-22	-0.091261414
0.247417144	2.03E-07	0.244631655	2.80E-07	8.38E-05
-0.019340895	0.689207108	0.142323388	0.00309883	-0.094262152
0.365394762	4.99E-15	0.199931889	2.97E-05	-0.028993015
-0.387599302	7.28E-17	-0.231244875	1.25E-06	0.069641006
0.132286057	0.006009908	0.017981477	0.710029464	0.056340403
0.453119573	3.69E-23	0.271969906	9.95E-09	0.007107986
-0.102469657	0.033647964	-0.087351435	0.0703663	0.032200172
0.334408043	1.08E-12	0.180428194	0.000168923	-0.025457075
-0.301473018	1.74E-10	-0.204833162	1.86E-05	0.126403463
0.416668209	1.73E-19	0.144439413	0.00268037	-0.023746696
0.073133526	0.129989623	-0.105215125	0.029146857	0.081364593
-0.17787386	0.000209448	-0.018063222	0.708771063	-0.063621938
0.727274356	5.81E-72	0.512997743	3.09E-30	-0.119945787
0.427087707	1.72E-20	0.242371701	3.63E-07	0.002690053
0.503506961	5.09E-29	0.337613828	6.35E-13	-0.051340574
0.456054213	1.79E-23	0.25883474	5.19E-08	-0.060389538
0.317009307	1.71E-11	0.220790424	3.80E-06	-0.034059499
-0.052553783	0.276878723	0.027035309	0.576102741	0.090019739
0.428780023	1.17E-20	0.131441528	0.006342134	-0.037850277
-0.501109817	1.02E-28	-0.341168564	3.51E-13	0.052533564
-0.43538635	2.56E-21	-0.315203712	2.26E-11	-0.030824646
-0.116822816	0.015361505	-0.087740301	0.069120357	-0.001396758
-0.171736554	0.000346959	-0.089584066	0.063456336	0.02451303
0.501549323	8.98E-29	0.373303042	1.15E-15	0.072697819
-0.319478062	1.17E-11	-0.18489785	0.000115134	-0.035847232
0.328265295	2.92E-12	0.19027426	7.17E-05	0.016625982
0.046370783	0.337416635	0.024889434	0.606765693	-0.05944242
-0.145077619	0.002564664	0.077251581	0.109675999	0.056620435
-0.223214196	2.95E-06	-0.046224221	0.338947497	0.036700921
-0.513412125	2.73E-30	-0.191754032	6.28E-05	0.098853307
-0.19525919	4.57E-05	-0.049929332	0.301609865	-0.015170998
0.630276176	5.43E-49	0.303116445	1.37E-10	-0.053037252
-0.24634633	2.30E-07	-0.095326216	0.04821448	0.058747768
-0.566416199	7.49E-38	-0.3820894	2.14E-16	0.013411882
-0.42392975	3.49E-20	0.090165078	0.061752748	0.081331731
0.4662969	1.34E-24	0.265265066	2.34E-08	-0.000166535
-0.360129412	1.30E-14	-0.087809903	0.068899267	0.15905611
0.416427671	1.83E-19	0.395984273	1.35E-17	-0.009100913
0.319852469	1.10E-11	0.189930503	7.40E-05	-0.021891572
0.644819773	6.61E-52	0.365915243	4.53E-15	-0.037301261
-0.177782702	0.00021105	-0.038108874	0.430563063	0.020152911
0.353378274	4.30E-14	0.291545087	7.17E-10	-0.002310284
0.655311953	4.15E-54	0.369229314	2.46E-15	-0.066713561
-0.093951319	0.051552349	-0.012114261	0.802212595	-0.087355867
0.323194973	6.53E-12	0.195794424	4.35E-05	-0.06898039
-0.44702691	1.63E-22	-0.255600605	7.69E-08	0.1048107
0.209928324	1.14E-05	0.144099397	0.002743938	0.001626249

-0.382144895	2.12E-16	-0.059897455	0.215139016	0.083679485
-0.336116136	8.13E-13	-0.150701497	0.001725395	0.092641547
-0.540253084	5.95E-34	-0.348124464	1.07E-13	0.141374911
0.427424678	1.59E-20	0.173558256	0.000299209	-0.036319962
0.263525129	2.91E-08	0.038969881	0.420213667	-0.016350725
0.290488382	8.30E-10	0.142948682	0.002969382	0.005489617
0.158509586	0.000972957	0.008279318	0.864076898	0.011707907
0.2379361	5.99E-07	0.217934052	5.09E-06	0.000304436
0.210314822	1.09E-05	0.138546252	0.003995872	0.006779984
-0.342070379	3.01E-13	-0.2024211	2.35E-05	0.070223566
-0.219972982	4.13E-06	-0.066714481	0.167299613	0.015569768
0.357770791	1.98E-14	0.157953236	0.001014379	-0.035878351
0.120397087	0.012474331	0.153117184	0.001449231	0.080239976
0.521299316	2.48E-31	0.290030044	8.85E-10	0.048498038
-0.385246327	1.16E-16	-0.360646637	1.18E-14	0.135638311
0.272826587	8.90E-09	0.028050076	0.561860826	0.123148129
0.162880827	0.000697894	0.248736989	1.74E-07	-0.065970397
0.154110105	0.001347985	0.000422793	0.993025225	0.055889994
-0.429735565	9.41E-21	-0.366700394	3.93E-15	-0.034675344
-0.362248923	8.85E-15	-0.009921072	0.837467494	0.046677555
-0.252083599	1.17E-07	-0.083427715	0.083993317	-0.046578772
0.379131477	3.79E-16	0.292827703	5.99E-10	-0.022145955
-0.145257457	0.002532891	-0.24275105	3.48E-07	-0.063316352
0.321373801	8.69E-12	0.163664439	0.000656958	0.000275187
-0.481518592	2.42E-26	-0.318625178	1.33E-11	0.004760826
-0.292730861	6.07E-10	-0.185276204	0.000111411	-0.011606759
0.314604219	2.47E-11	0.252343822	1.14E-07	0.001861663
-0.219715482	4.24E-06	-0.150285884	0.001777508	0.028204445
-0.228119513	1.75E-06	-0.015346711	0.750994337	0.050313027
0.279220879	3.84E-09	0.109472188	0.023190426	-0.015663617
-0.124974904	0.009481985	-0.078731204	0.103020303	0.057632045
0.060384514	0.211425371	0.079739012	0.098673148	0.067430297
-0.139110525	0.003848424	0.085984342	0.074893011	0.048900583
0.394049603	2.01E-17	0.24945516	1.60E-07	-0.019239661
-0.488147317	3.96E-27	-0.275521939	6.26E-09	0.078935085
0.527718003	3.35E-32	0.377174229	5.52E-16	-0.097825299
-0.454349136	2.73E-23	-0.208877688	1.26E-05	0.144483919
-0.27603389	5.86E-09	-0.11429532	0.017742257	0.071441193
-0.325756251	4.36E-12	-0.176398885	0.000236823	0.031554718
0.220215871	4.03E-06	0.236936605	6.70E-07	0.068986921
0.373842294	1.04E-15	0.224072075	2.70E-06	0.039886809
0.155735195	0.001196211	0.058687378	0.22456757	0.007793097
-0.322531495	7.25E-12	-0.293659377	5.33E-10	0.10002072
0.115706301	0.016376265	-0.079422977	0.100020361	-0.038442274
-0.18945623	7.72E-05	-0.00868967	0.857410312	0.005553168
0.506789738	1.95E-29	0.28940281	9.65E-10	-0.059273262
-0.340707621	3.79E-13	-0.170606906	0.000380042	0.016577109
0.320757883	9.57E-12	0.138564145	0.003991119	0.003424906
-0.45210689	4.74E-23	-0.278038486	4.50E-09	0.056335402
-0.217030076	5.58E-06	-0.102743376	0.033174101	0.074240616
-0.399459422	6.65E-18	-0.162503046	0.000718463	0.095793019

-0.123136375	0.010597126	-0.174257201	0.000282575	0.041683124
0.240475353	4.50E-07	0.117531749	0.014746193	-0.055729536
0.36570634	4.71E-15	0.239177903	5.21E-07	-0.021666121
0.045745108	0.343982851	-0.093545091	0.052574673	-0.050934247
0.036451478	0.450894695	-0.144092508	0.00274524	0.012847147
-0.32484179	5.04E-12	-0.176998848	0.000225307	0.026467774
0.423703152	3.67E-20	0.155033038	0.001259747	-0.04586661
0.19965246	3.04E-05	0.01237576	0.798034223	-0.020526738
0.20159304	2.54E-05	0.117812619	0.014508468	-0.104819523
0.21224003	9.04E-06	0.177092923	0.00022355	-0.006309136
-0.278992966	3.96E-09	-0.243908518	3.05E-07	0.038952854
-0.220228787	4.02E-06	-0.118783423	0.013712523	0.033525695
0.53882632	9.50E-34	0.305261593	1.00E-10	-0.052843653
-0.374652446	8.90E-16	-0.263700112	2.84E-08	-0.008342626
0.426811025	1.83E-20	0.2963767	3.63E-10	-0.046313691
-0.243042885	3.36E-07	-0.205257737	1.79E-05	0.030332048
0.539170982	8.49E-34	0.274143305	7.50E-09	-0.030923949
0.164248938	0.000627885	0.156071983	0.001166795	-0.027057874
-0.258207833	5.60E-08	-0.129208888	0.007301131	0.021503926
-0.238796752	5.44E-07	0.025537355	0.597430951	0.041415589
-0.607902421	8.44E-45	-0.39034841	4.22E-17	0.077748224
-0.428438703	1.26E-20	-0.256591336	6.82E-08	0.003056711
-0.131715629	0.006232544	-0.02221853	0.645911889	-0.065268341
0.18313874	0.000134027	-0.049277633	0.307971462	0.081856861
-0.378316855	4.43E-16	-0.14771523	0.002133178	0.056590006
0.114470867	0.017567058	-0.00118357	0.980476444	0.031217751
-0.473929119	1.84E-25	-0.365728843	4.69E-15	0.056276455
0.179785771	0.000178359	0.004761049	0.921582601	-0.012332246
-0.485418027	8.39E-27	-0.311651339	3.87E-11	0.078962951
0.047563924	0.325119198	0.113924151	0.01811772	-0.017049888
0.387795673	7.00E-17	0.292854933	5.97E-10	-0.01833101
-0.107588049	0.025682565	0.129843217	0.007016275	0.039556243
0.187425601	9.23E-05	0.14045543	0.003516653	0.013040713
0.405565923	1.87E-18	0.194720366	4.80E-05	-0.057706535
0.338296561	5.67E-13	0.432123222	5.45E-21	-0.048962891
0.017198471	0.72212272	0.018227178	0.706249475	0.084361886
-0.07831281	0.104869032	-0.185443715	0.0001098	0.003620149
0.300450122	2.02E-10	0.191327758	6.53E-05	-0.050461915
0.686270827	3.75E-61	0.407054878	1.36E-18	-0.028570383
-0.381586182	2.36E-16	-0.293777374	5.24E-10	0.040041094
0.229939602	1.44E-06	0.007364302	0.878976375	-0.011126473
-0.429554892	9.80E-21	-0.295715385	3.98E-10	-0.000855709
-0.123230097	0.010537585	-0.072363166	0.134093961	-0.138929204
0.323171427	6.55E-12	0.213953864	7.62E-06	-0.023585114
-0.174455548	0.000278014	-0.091436712	0.058155606	-0.031571565
-0.177470135	0.000216631	-0.113106879	0.018968971	-0.068201392
0.085119621	0.077876686	-0.051794422	0.28388849	0.092490762
0.347324751	1.23E-13	0.134101534	0.00534807	0.121615771
-0.381305685	2.49E-16	-0.145409879	0.002506244	0.0939027
-0.426278697	2.06E-20	-0.255604441	7.68E-08	0.14477993
-0.267303811	1.81E-08	-0.088953599	0.065348514	0.091553033

-0.2300795	1.42E-06	-0.002969518	0.951042136	0.007428876
-0.285230795	1.71E-09	-0.176828984	0.000228513	0.012889186
-0.514198512	2.15E-30	-0.345112568	1.80E-13	0.127009115
0.429331543	1.03E-20	0.444269105	3.16E-22	0.013688786
0.384322082	1.39E-16	0.255705159	7.59E-08	-0.061857448
0.264102946	2.70E-08	0.0473245	0.327563287	0.079632035
-0.035127928	0.46751263	0.080309586	0.096277464	0.01017405
0.335394036	9.16E-13	0.193217158	5.51E-05	-0.025773981
0.060203232	0.21280214	0.049577725	0.305031182	0.061089756
0.637214989	2.31E-50	0.423722708	3.65E-20	-0.056058102
0.339392145	4.72E-13	0.15833848	0.000985527	-0.009689069
0.318585599	1.34E-11	0.115900292	0.016195851	-0.048657242
0.456134594	1.75E-23	0.399721052	6.30E-18	-0.104504111
-0.282996908	2.32E-09	-0.228406081	1.70E-06	0.118900764
-0.231227084	1.26E-06	-0.050839409	0.292872966	0.043451384
0.394069748	2.00E-17	0.363447984	7.12E-15	-0.073457752
0.11292798	0.01915989	0.033663926	0.486281428	0.096167917
-0.182062068	0.000146989	-0.070248598	0.145873479	-0.014578991
-0.185029705	0.000113823	-0.153455995	0.001413924	0.056451478
0.331749132	1.66E-12	0.1335004	0.005559571	0.026056397
-0.345714512	1.62E-13	-0.120634277	0.012300906	0.211444484
0.134564906	0.005190012	0.037913088	0.432936841	-0.013327088
0.140376817	0.003535309	0.078108129	0.105782953	-0.001763326
0.208153982	1.35E-05	0.052903952	0.273686197	-0.040511629
-0.112852473	0.019240971	0.032867103	0.496664971	0.066627953
0.203935489	2.03E-05	0.051502126	0.286618353	0.055065676
0.54569153	9.82E-35	0.551738632	1.27E-35	-0.02211624
0.19418124	5.04E-05	0.101925439	0.03460727	-0.028876512
-0.433020994	4.43E-21	-0.255031097	8.23E-08	0.180821623
-0.047923156	0.321474313	0.247785668	1.94E-07	0.038270196
0.359231556	1.52E-14	0.169317262	0.000421393	0.013175347
0.169188184	0.000425754	0.264018328	2.73E-08	-0.000151595
-0.358639318	1.69E-14	-0.104170729	0.030794258	0.134915466
-0.325244021	4.72E-12	-0.216841583	5.69E-06	0.0091528
0.067067925	0.165056279	-0.064529882	0.181674364	0.041626651
-0.139060097	0.003861398	-0.081737899	0.090483461	-0.078508955
0.664031663	5.24E-56	0.415684176	2.15E-19	-0.15306012
0.560003818	7.29E-37	0.295147114	4.32E-10	-0.058950702
-0.230982597	1.29E-06	-0.287780022	1.21E-09	-0.050165089
0.374086059	9.91E-16	0.264082111	2.71E-08	0.029884287
-0.317746285	1.53E-11	-0.132335805	0.005990835	0.130024912
0.534350032	4.06E-33	0.371412501	1.64E-15	-0.076857437
0.771087651	6.18E-86	0.436844404	1.83E-21	-0.086181919
-0.2388557	5.41E-07	0.033518533	0.488167308	-0.003527764
-0.488543648	3.55E-27	-0.268013039	1.65E-08	-0.0304067
0.240153571	4.67E-07	0.155198057	0.001244542	-0.046587757
-0.012724547	0.792470106	0.055010027	0.255014533	0.035392249
-0.520896521	2.80E-31	-0.350974062	6.55E-14	0.07132425
-0.299373452	2.36E-10	-0.303784881	1.24E-10	0.06097579
0.223607929	2.83E-06	-0.007110192	0.883121948	0.090593607
0.257824939	5.87E-08	0.180364196	0.000169841	-0.019812319

-0.422246679	5.07E-20	-0.187413973	9.24E-05	0.097441213
-0.325660152	4.42E-12	-0.216615029	5.82E-06	0.086224562
-0.101219466	0.035886312	-0.187380809	9.27E-05	0.038134193
-0.398401356	8.27E-18	-0.189908445	7.41E-05	0.157135685
0.179978521	0.000175477	0.040943042	0.397052661	0.037128961
0.379487001	3.54E-16	0.29832236	2.75E-10	-0.016815079
-0.381200873	2.54E-16	-0.342387767	2.85E-13	-0.021986358
0.516073747	1.22E-30	0.213908581	7.65E-06	-0.066182952
0.081016567	0.093373631	0.04607899	0.340468839	0.109345816
0.187837741	8.90E-05	0.195699738	4.39E-05	0.025721634
0.231361932	1.24E-06	0.11325395	0.018813262	-0.033322535
-0.188189344	8.63E-05	-0.123135342	0.010597784	0.005099006
0.288425336	1.10E-09	0.139148675	0.003838636	-0.090266706
-0.369714602	2.25E-15	-0.134505639	0.005209991	0.129721935
-0.46703431	1.11E-24	-0.226874425	2.00E-06	0.104618856
0.32156746	8.43E-12	0.069121459	0.15246672	0.008740759
-0.136262775	0.004646168	0.073710744	0.12697851	0.009797632
0.093692415	0.052201979	0.074371185	0.123599922	0.078431369
0.251387984	1.27E-07	0.066120486	0.171121051	-0.020456344
0.319723794	1.12E-11	0.19494123	4.71E-05	0.003007608
0.664269315	4.64E-56	0.571529367	1.18E-38	-0.065274295
0.450368913	7.25E-23	0.191342385	6.52E-05	-0.008075556
0.243224721	3.29E-07	0.128571249	0.00759785	-0.049540203
0.255265926	8.00E-08	0.174689697	0.000272718	-0.042564392
0.510700622	6.14E-30	0.202640977	2.30E-05	-0.045802688
0.101358582	0.035631139	0.115478379	0.016590482	-0.0497849
-0.00753232	0.876237122	0.040102447	0.40682429	-0.093701075
-0.162697634	0.000707799	0.068478495	0.156327662	-0.008971859
0.384373125	1.37E-16	0.354451565	3.56E-14	-0.052493743
0.509640961	8.41E-30	0.259014561	5.07E-08	-0.031726122
0.039194182	0.417541656	-0.013722665	0.776606355	0.276181009
0.014744251	0.76046525	0.003818281	0.937074479	-0.049935813
0.463703382	2.60E-24	0.318677825	1.32E-11	-0.007240298
0.272471311	9.32E-09	0.176271897	0.00023933	0.138226898
-0.276551865	5.47E-09	-0.10574112	0.028346181	0.100222248
0.363782835	6.70E-15	0.201190212	2.64E-05	-0.128045281
-0.508358786	1.23E-29	-0.407755002	1.18E-18	-0.014881548
-0.24762364	1.98E-07	-0.178724718	0.000195034	-0.075824904
-0.526812152	4.46E-32	-0.268642157	1.52E-08	0.104947358
0.310618044	4.52E-11	0.121948864	0.011377774	0.078525824
-0.280936032	3.06E-09	-0.020585577	0.670345302	0.069094522
0.072567314	0.132996691	-0.115679462	0.016401364	0.104539491
0.700016499	1.43E-64	0.413110289	3.75E-19	0.008599084
0.440758758	7.28E-22	0.389543847	4.95E-17	-0.023052752
0.565997023	8.70E-38	0.253754704	9.60E-08	-0.046806854
0.068495947	0.156221897	-0.034371784	0.477156221	0.07091165
0.187688467	9.02E-05	0.103107814	0.032552025	-0.019976518
-0.128714384	0.007530321	-0.076208473	0.114568374	0.039115676
-0.330790269	1.94E-12	-0.094305732	0.050674014	0.133120648
0.198811833	3.29E-05	0.019253533	0.69053841	-0.013288537
0.106852973	0.02671566	0.117732358	0.014576054	0.03394107

0.576236567	2.08E-39	0.335370417	9.20E-13	-0.058234841
0.131634463	0.006264817	0.013096065	0.786554941	0.06065738
0.314346063	2.57E-11	0.24394296	3.03E-07	0.067756935
0.271401316	1.07E-08	0.218092794	5.01E-06	0.000365566
0.266079679	2.11E-08	0.077930407	0.106581597	0.029578483
0.448625007	1.11E-22	0.318288458	1.40E-11	-0.01227685
0.683541848	1.70E-60	0.533919836	4.66E-33	-0.087566865
-0.297205989	3.22E-10	-0.233882144	9.39E-07	0.081845197
0.195832493	4.34E-05	0.068376348	0.156947786	-0.03103488
0.355897342	2.76E-14	0.224999321	2.44E-06	0.012565135
-0.372264207	1.40E-15	-0.185385359	0.000110359	0.024989818
-0.291190831	7.53E-10	-0.098313017	0.041583298	0.118038616
0.110211785	0.022270951	-0.008657161	0.857938079	-0.030727405
0.165285549	0.000579238	0.006655007	0.890555778	0.093772864
0.435188733	2.68E-21	0.445387961	2.42E-22	0.028782748
0.34073009	3.77E-13	0.266161856	2.09E-08	-0.016182988
0.326211062	4.05E-12	0.162343766	0.000727302	-0.052453306
0.231916568	1.16E-06	0.146819302	0.002271678	0.012760212
0.406174344	1.64E-18	0.188372451	8.49E-05	-0.023957391
0.450239507	7.48E-23	0.308762933	5.97E-11	-0.049503785
0.479874028	3.77E-26	0.357264726	2.16E-14	0.025860865
0.411521949	5.27E-19	0.215893491	6.27E-06	-0.030869784
-0.246183349	2.34E-07	-0.111781526	0.020423564	0.087687852
-0.392289329	2.86E-17	-0.303581023	1.28E-10	0.027261541
-0.162059884	0.000743305	0.06788031	0.159985645	-0.088306852
-0.280565739	3.21E-09	-0.060182968	0.212956443	0.098448222
0.203257626	2.17E-05	0.026692166	0.58095676	0.033564983
0.379515779	3.52E-16	0.232813343	1.06E-06	0.071044115
0.678650134	2.44E-59	0.365427274	4.96E-15	-0.109119714
-0.129358125	0.007233199	-0.140596842	0.00348332	0.058434907
0.328972619	2.60E-12	0.245342174	2.58E-07	0.031515699
0.573376078	5.99E-39	0.321823967	8.10E-12	-0.035888039
0.404712018	2.23E-18	0.109259575	0.023460753	-0.013314687
0.322460561	7.33E-12	0.259760618	4.63E-08	0.039838836
-0.046038575	0.340892971	-0.042030888	0.384618404	-0.028962663
0.293575	5.39E-10	0.183591788	0.000128901	0.001213303
-0.254243295	9.05E-08	-0.211722063	9.52E-06	0.082759972
-0.156991641	0.001089829	0.014651909	0.761920111	0.075105203
0.357582189	2.05E-14	0.185501736	0.000109247	0.050661827
-0.058404508	0.226813407	-0.10241811	0.033737845	0.022718191
0.496462961	3.85E-28	0.34230306	2.90E-13	-0.099193114
0.314647012	2.46E-11	0.305706237	9.39E-11	0.017589405
0.07348598	0.128144515	-0.060202899	0.21280468	0.068548528
-0.117134781	0.015088016	-0.106097358	0.027814701	0.01067939
0.098668986	0.040846918	0.071559192	0.138483145	0.049495976
-0.204247821	1.97E-05	-0.035434741	0.463630561	0.112280968
0.231535888	1.21E-06	0.154485577	0.001311424	0.017319866
0.259941972	4.53E-08	0.095790947	0.047127972	0.011760252
0.167747683	0.000477362	0.178024106	0.000206832	-0.018748013
0.411603191	5.18E-19	0.124092109	0.010003712	-0.038280516
0.357111687	2.22E-14	0.198192208	3.49E-05	0.148067072

-0.118706488	0.013774168	-0.010288528	0.831535953	0.006133053
-0.148100111	0.002076085	0.001005463	0.983413948	-0.070230731
-0.022529058	0.641307302	0.120030859	0.012746344	-0.047418069
-0.176802431	0.000229018	0.02396227	0.620235248	0.075800993
0.010146223	0.833831945	0.128574423	0.007596347	0.04840902
-0.593930939	2.39E-42	-0.393100277	2.43E-17	0.025340998
0.24952183	1.59E-07	0.236233301	7.25E-07	-0.02835594
0.268331781	1.59E-08	0.124208702	0.009933377	0.066542591
0.180625078	0.000166126	-0.016840222	0.727679119	0.000997218
0.463730102	2.59E-24	0.423123017	4.18E-20	-0.021376353
0.518389996	6.05E-31	0.270095741	1.27E-08	-0.047695141
0.466906637	1.15E-24	0.237802073	6.08E-07	-0.022845471
0.431118233	6.86E-21	0.348290097	1.04E-13	0.011554504
0.544527984	1.45E-34	0.541977448	3.37E-34	-0.073330333
0.468952197	6.75E-25	0.175107272	0.000263507	-0.028962404
0.679291384	1.73E-59	0.373387742	1.13E-15	-0.083399071
-0.506137082	2.36E-29	-0.278060983	4.48E-09	0.203738854
0.30678342	8.01E-11	0.359272445	1.51E-14	0.026526379
0.143545007	0.002850531	-0.045246761	0.349270559	0.021401222
0.268554985	1.54E-08	0.26803288	1.65E-08	0.045813674
0.533457477	5.41E-33	0.36697204	3.73E-15	-0.027149691
-0.367142649	3.62E-15	-0.238850127	5.41E-07	0.003875934
0.021926594	0.650253148	-0.149880595	0.00182971	0.050177872
0.107525365	0.025769299	0.055929308	0.247147809	0.091756832
0.589962688	1.13E-41	0.308512215	6.19E-11	-0.051156876
0.577796061	1.17E-39	0.389496024	5.00E-17	-0.074653135
0.194971199	4.69E-05	0.256593902	6.82E-08	0.057820131
-0.189772863	7.50E-05	-0.192535545	5.86E-05	-0.067181572
0.273604627	8.05E-09	0.084732533	0.079243213	-0.052311821
0.075965423	0.115732546	0.018426749	0.703184486	0.066099483
-0.353594947	4.14E-14	-0.222053691	3.33E-06	0.053301067
0.546311462	7.98E-35	0.482283419	1.97E-26	-0.054802654
0.057603716	0.233257706	0.050612988	0.295030662	0.179221609
-0.449516596	8.92E-23	-0.135746485	0.004805833	0.08681106
-0.327695637	3.20E-12	-0.173828916	0.000292662	0.02411308
0.055715863	0.248959087	0.053163366	0.271337329	0.039500189
0.278429782	4.27E-09	0.252043881	1.18E-07	-0.05015108
0.238950531	5.35E-07	0.088790372	0.06584588	-0.027979004
0.500083234	1.37E-28	0.320214903	1.04E-11	-0.013543598
0.374681174	8.86E-16	0.160673743	0.000826234	-0.034553277
-0.144485345	0.002671887	-0.003706352	0.938915427	0.012951675
0.684457724	1.02E-60	0.435731217	2.37E-21	-0.086883383
0.415675372	2.15E-19	0.344493958	2.00E-13	-0.024016987
0.129726752	0.007067818	-0.048539395	0.315283935	0.109855901
-0.138660021	0.003965738	-0.048266259	0.318018009	0.049209685
0.214033932	7.56E-06	0.143475927	0.002864074	0.026954973
0.585986755	5.26E-41	0.374365291	9.40E-16	-0.110499962
-0.41883306	1.08E-19	-0.323890921	5.85E-12	0.065283495
0.558094574	1.42E-36	0.304968829	1.05E-10	-0.060375278
0.040244986	0.405157331	0.091376726	0.058321304	-0.054234684
-0.407606268	1.21E-18	-0.188265585	8.57E-05	0.141436978

0.474754035	1.48E-25	0.428167825	1.34E-20	0.01052496
0.059333443	0.219497645	-0.090351227	0.061214985	0.020023721
0.147757094	0.002126899	-0.006970405	0.885403816	0.050600223
0.23822647	5.80E-07	0.130979705	0.006530691	-0.023139517
0.48946773	2.75E-27	0.206954722	1.52E-05	-0.039485144
0.143275757	0.002903648	0.135889553	0.004761101	0.031256207
0.102899912	0.03290567	0.038000449	0.43187671	0.005583368
0.519361781	4.49E-31	0.400924878	4.92E-18	-0.033251584
-0.049228508	0.308454565	-0.196737202	3.99E-05	-0.079223752
-0.483871793	1.28E-26	-0.296763157	3.43E-10	-0.041931786
0.522606407	1.65E-31	0.280698175	3.16E-09	-0.018415849
0.447885325	1.33E-22	0.4774901	7.15E-26	-0.016448676
-0.326222731	4.04E-12	-0.199332948	3.14E-05	0.126456059
0.534785809	3.53E-33	0.466187033	1.38E-24	-0.036319583
0.210575454	1.07E-05	0.190008759	7.35E-05	-0.142847993
-0.194757677	4.79E-05	-0.036166441	0.454445095	-0.033336581
0.169957483	0.000400368	0.057601696	0.23327412	0.004469745
0.038371065	0.427395995	0.009250824	0.848310248	0.049229003
0.227467502	1.88E-06	0.075751244	0.116766098	0.029231067
0.468611384	7.38E-25	0.345919769	1.57E-13	-0.03853316
-0.11464971	0.017390127	-0.210468154	1.08E-05	0.09626329
0.336017643	8.27E-13	0.272464719	9.33E-09	-0.094455595
-0.182802751	0.000137952	-0.046392145	0.33719388	0.074051462
0.603188851	5.85E-44	0.471870194	3.16E-25	-0.099929628
-0.305677592	9.43E-11	-0.17600915	0.000244596	0.18789374
0.358781114	1.65E-14	0.136792522	0.004487332	-0.041280592
0.179928736	0.000176217	0.083481546	0.083792907	-0.02522021
0.328275081	2.91E-12	0.211771544	9.47E-06	0.019090146
0.36215127	9.00E-15	0.174997157	0.000265907	-0.056756963
0.237667207	6.18E-07	0.052716617	0.275391015	-0.007470459
0.6119446	1.56E-45	0.446249599	1.97E-22	-0.064095271
-0.106754067	0.02685736	0.042644121	0.377714845	0.063379742
0.186800916	9.75E-05	0.114337249	0.017700273	0.105936345
-0.220771947	3.80E-06	-0.060430205	0.211079377	0.112982981
-0.037955744	0.432419023	-0.044512555	0.357154004	0.02887479
0.3845891	1.32E-16	0.238420598	5.68E-07	-0.041038899
-0.166857991	0.000512081	-0.032541498	0.500941645	0.045084082
0.023275828	0.630290437	-0.066671151	0.167576199	0.036842671
0.288468038	1.10E-09	0.139875658	0.003656359	0.01415885
0.471646819	3.35E-25	0.222501302	3.18E-06	-0.01993927
0.216768502	5.73E-06	0.023280705	0.630218757	0.017706212
0.619032891	7.64E-47	0.457406413	1.28E-23	-0.136899018
0.627002599	2.34E-48	0.379969386	3.23E-16	-0.045843626
0.400543819	5.32E-18	0.290527714	8.26E-10	-0.035152477
0.070013031	0.147233141	0.057321548	0.235558961	0.032823228
0.321890641	8.02E-12	0.048669332	0.313988699	-0.006012743
0.488114401	3.99E-27	0.216786506	5.72E-06	-0.010140599
0.570377287	1.79E-38	0.425103957	2.68E-20	-0.031218821
0.011498312	0.812076639	0.013404682	0.781650545	-0.053238346
0.23593834	7.49E-07	0.242162776	3.72E-07	0.029365417
0.471906012	3.13E-25	0.216226509	6.06E-06	-0.038812895

0.227808493	1.81E-06	0.32316394	6.56E-12	0.042798203
0.408865206	9.30E-19	0.209830434	1.15E-05	-0.106461066
-0.220609778	3.87E-06	-0.013064944	0.787049967	-0.027442824
0.069204836	0.151971391	0.045006091	0.351842512	0.036497752
0.114833732	0.017209698	0.179519588	0.000182412	-0.050570294
0.580961888	3.56E-40	0.403094919	3.13E-18	-0.025428236
0.097055654	0.044274629	0.04872291	0.313455634	-0.00158664
0.355806841	2.80E-14	0.213437899	8.02E-06	-0.094300444
-0.41221523	4.55E-19	-0.236669669	6.90E-07	0.08802643
-0.057823892	0.231473101	0.040762841	0.399135466	0.039309213
0.29964248	2.27E-10	0.181076822	0.000159871	0.010539478
0.091069954	0.059174815	0.160543032	0.00083448	0.085143466
0.436417598	2.02E-21	0.231021612	1.28E-06	-0.085927327
0.232714609	1.07E-06	0.138939292	0.003892639	0.186729953
0.630963559	3.98E-49	0.37964582	3.44E-16	-0.04301912
-0.255974672	7.35E-08	-0.315412862	2.18E-11	0.043431223
0.465387452	1.69E-24	0.327510107	3.29E-12	-0.006310033
-0.220422562	3.94E-06	-0.112392623	0.01974125	0.017640375
0.550142294	2.19E-35	0.252591915	1.10E-07	-0.04171413
-0.254940605	8.32E-08	-0.142531829	0.00305512	0.020274401
0.47726628	7.59E-26	0.26436361	2.62E-08	-0.014453276
0.147889365	0.002107172	-0.010971056	0.820544072	0.080976101
0.072252803	0.134690044	0.179837209	0.000177586	0.022251991
0.292055282	6.67E-10	0.277474824	4.85E-09	-0.048437044
0.312399201	3.46E-11	0.23753498	6.27E-07	0.027998283
-0.360329975	1.25E-14	-0.244736328	2.77E-07	0.007588895
-0.135489883	0.004887015	-0.097409431	0.043502971	0.082021985
0.023776296	0.622952564	-0.174180039	0.000284368	-0.102682188
0.235258203	8.07E-07	0.211767529	9.47E-06	0.02145147
-0.389536208	4.96E-17	-0.229335692	1.54E-06	0.002378814
0.283913113	2.05E-09	0.271576419	1.05E-08	0.022267359
0.577833837	1.15E-39	0.394143409	1.97E-17	-0.050025374
-0.302329622	1.54E-10	-0.141658105	0.003242169	0.085646443
0.300520919	2.00E-10	0.177093635	0.000223537	-0.061526919
0.312954973	3.18E-11	0.082664008	0.08687844	-0.016263326
0.403146827	3.10E-18	0.136518225	0.004568953	-0.025210714
-0.112353636	0.01978418	-0.09032827	0.061281095	-0.013222247
0.12470181	0.009640738	0.020561923	0.670701877	0.014496345
0.281455002	2.85E-09	0.227425805	1.89E-06	-0.097969197
-0.480855958	2.90E-26	-0.274955751	6.75E-09	0.181570766
-0.260508229	4.22E-08	-0.082219313	0.088594795	0.102194668
0.395683599	1.44E-17	0.241064265	4.21E-07	-0.015107982
0.331427488	1.75E-12	0.159902381	0.000876007	-0.05835462
0.346545581	1.41E-13	0.234828595	8.46E-07	-0.032556796
0.334385643	1.08E-12	0.201665156	2.52E-05	-0.012525211
-0.274063513	7.58E-09	-0.100489331	0.037251163	0.074338534
0.423928268	3.49E-20	0.314131363	2.66E-11	-0.056523812
0.199443502	3.10E-05	0.088174266	0.067751297	-0.006348763
0.103887829	0.031253977	0.023303496	0.629883807	0.007430983
0.381689946	2.31E-16	0.125139571	0.009387389	-0.019731937
0.560720821	5.66E-37	0.359990294	1.33E-14	0.000886108

-0.383569468	1.61E-16	-0.073553051	0.127795709	-0.015660702
-0.16971747	0.000408133	-0.102719696	0.033214868	0.063529398
0.399593989	6.47E-18	0.254050712	9.26E-08	-0.001213853
0.513865388	2.38E-30	0.284812077	1.81E-09	-0.002922043
0.225252608	2.38E-06	0.137585374	0.004258756	0.029029891
0.268501944	1.55E-08	0.044716565	0.354952352	-0.003407554
0.293613275	5.36E-10	0.080694489	0.094687719	-0.073194801
0.284838937	1.81E-09	0.164430503	0.000619099	0.000937793
0.267714436	1.72E-08	0.053155483	0.271408504	-0.007584939
-0.171175695	0.000363034	-0.06579003	0.173275021	-0.058552808
-0.410309026	6.84E-19	-0.251146356	1.31E-07	0.135103808
0.391534908	3.33E-17	0.323087808	6.64E-12	-0.030761911
0.615087041	4.14E-46	0.286377595	1.46E-09	-0.060571157
0.314873094	2.37E-11	0.136497289	0.004575238	0.017988867
0.573719374	5.28E-39	0.346707558	1.37E-13	-0.052378914
0.235130156	8.19E-07	0.092618147	0.054970798	0.035934749
0.08178012	0.09031654	0.231340549	1.24E-06	-0.021189697
0.281829851	2.71E-09	0.061831766	0.200663594	0.020740895
0.253000528	1.05E-07	0.200817154	2.73E-05	-0.004259006
-0.46780308	9.09E-25	-0.278890413	4.02E-09	0.044675793
0.506194396	2.32E-29	0.336329272	7.85E-13	-0.035705849
0.141458479	0.003286338	0.138880611	0.003907897	0.091353683
0.15407454	0.001351496	0.089776301	0.06288845	0.017296643
0.275507524	6.28E-09	0.170366228	0.000387461	-0.041347146
0.339237986	4.84E-13	0.149473404	0.001883568	-0.043126192
0.375115537	8.16E-16	0.109963726	0.022575757	0.033348445
-0.071461531	0.139023743	-0.023072316	0.633284811	0.059079299
0.277337408	4.93E-09	0.094304672	0.050676622	-0.046428752
-0.159466119	0.000905369	-0.006810365	0.888017442	0.127652081
-0.146754487	0.00228201	-0.029501059	0.541796189	0.032398916
-0.308241007	6.45E-11	-0.16184786	0.00075547	0.088681554
-0.18858595	8.33E-05	-0.127545663	0.008097737	-0.022399128
0.229704795	1.48E-06	0.163089436	0.000686772	0.009393082
0.246470301	2.27E-07	0.193475194	5.38E-05	0.001783158
-0.48245649	1.88E-26	-0.34015605	4.15E-13	0.137883912
0.289597463	9.40E-10	0.222437645	3.20E-06	-0.023970179
-0.190566299	6.99E-05	-0.090438666	0.060963721	0.017995056
0.223146329	2.97E-06	0.245560491	2.52E-07	-0.103830618
0.33073339	1.96E-12	0.311820187	3.77E-11	-0.051111504
-0.308849851	5.89E-11	-0.206538134	1.58E-05	0.251041289
0.555364808	3.67E-36	0.343927265	2.20E-13	-0.058213903
0.488768592	3.33E-27	0.230121178	1.41E-06	-0.019106304
0.728971672	1.88E-72	0.465499012	1.65E-24	-0.077591597
-0.268777928	1.50E-08	-0.085535791	0.076428889	0.056169622
0.032937414	0.495744017	0.059200258	0.220536045	0.034797324
0.661076033	2.34E-55	0.415850741	2.07E-19	-0.087462533
0.261358462	3.80E-08	0.267596329	1.74E-08	-0.002086623
0.348458387	1.01E-13	0.124576924	0.009714119	-0.074973725
0.547768341	4.89E-35	0.220237722	4.02E-06	-0.010569106
0.169457284	0.000416709	0.077310622	0.109404087	0.013630315
0.36779736	3.21E-15	0.244185869	2.95E-07	0.0135185

0.314473078	2.52E-11	0.103348402	0.032146834	-0.026665557
0.393970345	2.04E-17	0.156296192	0.001147583	-0.014522438
0.214247142	7.40E-06	0.12653931	0.00861656	0.001271987
-0.426368116	2.02E-20	-0.185618163	0.000108145	0.092372209
-0.200917418	2.70E-05	-0.008415526	0.861862974	0.100913297
-0.34644512	1.43E-13	-0.097882903	0.042487975	0.040270056
-0.131020102	0.006513999	-0.02853879	0.555062922	0.002761107
0.171608656	0.000350566	0.115179838	0.016874779	-0.020912775
0.098996641	0.040178871	0.062837628	0.193422337	0.072408392
0.354333494	3.64E-14	0.187713247	9.00E-05	-0.025306221
0.40382192	2.69E-18	0.2748215	6.87E-09	-0.002133843
0.306553258	8.28E-11	0.198996788	3.24E-05	-0.026030335
0.019964002	0.679739628	-0.263140659	3.05E-08	-0.094288481
-0.015657789	0.746118564	-0.074285387	0.12403484	0.079133644
0.3229952	6.74E-12	0.136585671	0.00454876	-0.0153393
0.454556333	2.59E-23	0.261024207	3.96E-08	-0.043308508
-0.237522564	6.28E-07	-0.034651132	0.47358094	0.057467115
0.089959619	0.062350811	0.112100031	0.020065425	-0.020331911
-0.342088064	3.00E-13	-0.153999563	0.001358926	0.134023503
0.361901232	9.42E-15	0.207565636	1.43E-05	0.071207272
0.347630764	1.17E-13	0.099051099	0.040068737	0.017698533
0.409572645	8.00E-19	0.179400777	0.000184249	-0.010455086
0.506385173	2.20E-29	0.384587118	1.32E-16	-0.029649783
0.313569957	2.89E-11	0.184880881	0.000115303	-0.000527057
-0.465961234	1.46E-24	-0.221864924	3.40E-06	0.053711096
-0.232109343	1.14E-06	-0.025611402	0.596368285	0.034669528
-0.006634079	0.8908978	0.136561182	0.004556083	-0.039354515
0.189792282	7.49E-05	0.193421625	5.40E-05	0.028000003
-0.229964303	1.44E-06	-0.16569854	0.00056085	-0.014017351
0.175585604	0.000253313	0.050035445	0.300582344	-0.043554734
0.018093415	0.708306455	-0.007156869	0.882360209	-0.020308437
0.735112652	2.95E-74	0.47614183	1.02E-25	-0.082741407
0.103292482	0.032240627	0.018833947	0.696945699	-0.008753296
0.345964955	1.55E-13	0.253985001	9.33E-08	0.121460233
0.649036433	8.82E-53	0.449324221	9.35E-23	-0.05526574
-0.145227304	0.002538194	0.174098855	0.000286266	0.016596744
0.362750729	8.08E-15	0.09817353	0.041874889	-0.047918021
0.649163583	8.30E-53	0.42225423	5.07E-20	-0.025223841
-0.315727687	2.08E-11	-0.084812996	0.078957567	0.052058609
-0.261734489	3.63E-08	-0.080987722	0.093490721	0.021047529
0.241144149	4.18E-07	0.218252416	4.93E-06	0.00625931
0.416650683	1.74E-19	0.388190747	6.48E-17	0.00298781
0.221267514	3.61E-06	0.136073808	0.004704043	-0.018410467
0.208957353	1.25E-05	0.236203343	7.27E-07	0.015293939
-0.335542147	8.94E-13	-0.101127223	0.036056364	0.106344007
0.40234083	3.66E-18	0.30080676	1.92E-10	-0.027802868
-0.296299758	3.67E-10	-0.163460414	0.000667397	0.079913625
0.519610618	4.16E-31	0.306903033	7.87E-11	-0.042488865
0.289524363	9.49E-10	0.120480507	0.012413093	-0.045635396
-0.140810674	0.003433461	-0.089702237	0.063106744	0.003692048
0.093114972	0.053675476	-0.058038713	0.229741233	0.101566139

0.006544662	0.89235934	-0.104495236	0.030274063	0.083395012
-0.351737677	5.73E-14	-0.232726908	1.07E-06	0.009533351
0.400710694	5.14E-18	0.231774754	1.18E-06	-0.017695971
0.116515677	0.015635	-0.002760604	0.954482593	-0.06454661
0.271739053	1.02E-08	0.195722723	4.38E-05	-0.081544005
0.434289949	3.31E-21	0.211132755	1.01E-05	-0.037853101
-0.34018357	4.14E-13	-0.192837535	5.70E-05	0.125084905
-0.26448083	2.58E-08	-0.253178278	1.03E-07	0.052003744
0.21344337	8.02E-06	0.049612239	0.304694212	-0.010288654
-0.336558434	7.56E-13	-0.165470747	0.000570924	-0.025103391
0.505770785	2.63E-29	0.310914045	4.32E-11	-0.088964058
0.662428006	1.18E-55	0.286733196	1.39E-09	-0.063653122
-0.196995948	3.90E-05	-0.073388685	0.12865182	-0.004283689
-0.205405671	1.76E-05	0.059399289	0.218985563	0.015759631
-0.159531914	0.000900883	-0.059808031	0.215825896	0.072436396
-0.291712292	7.00E-10	-0.155925689	0.00117949	0.030498509
0.665521932	2.44E-56	0.499746314	1.51E-28	-0.010142724
0.0185138	0.701849065	-0.03074024	0.524947257	0.079745932
0.462552844	3.49E-24	0.253258193	1.02E-07	-0.031105916
0.330544162	2.02E-12	0.065852435	0.172866716	-0.020849404
0.264637886	2.53E-08	0.088099998	0.067984005	0.005435343
0.49639519	3.93E-28	0.290574499	8.20E-10	-0.149706536
-0.152433523	0.001522953	-0.074933689	0.120777853	-0.007090932
0.091773392	0.057232822	0.001017299	0.983218723	0.035024575
0.010318037	0.83106003	-0.058917525	0.222752037	0.015577291
-0.076844447	0.111565586	-0.044050096	0.362176467	-0.037247303
0.275830043	6.02E-09	0.273200808	8.48E-09	-0.04034339
-0.479170827	4.56E-26	-0.266664804	1.96E-08	-0.050407531
-0.125028081	0.009451344	0.123263726	0.010516294	0.005848074
0.394270984	1.92E-17	0.13455857	0.005192144	-0.014564158
0.386553409	8.95E-17	0.113244148	0.018823604	-0.014988673
0.131074301	0.006491665	0.026285112	0.586739466	0.105283442
0.345457988	1.70E-13	0.109775633	0.02280928	-0.004680803
0.282290713	2.55E-09	0.041668204	0.388737318	0.013956883
-0.480832935	2.92E-26	-0.355479457	2.97E-14	-0.002287479
0.151398171	0.001641173	0.114907236	0.017138088	0.032060783
0.228848605	1.62E-06	0.087930313	0.068518151	-0.014492129
0.515689895	1.37E-30	0.438231839	1.32E-21	0.037554686
-0.26546416	2.28E-08	-0.163518893	0.000664389	0.056001742
0.090832983	0.059841171	-0.061345346	0.204235246	0.067982392
0.028704923	0.552761198	-0.061857274	0.200477556	-0.005665335
0.601858241	1.01E-43	0.339148449	4.92E-13	-0.093052346
0.511655653	4.62E-30	0.336665983	7.43E-13	-0.140664496
-0.150142498	0.001795818	-0.066720242	0.167262866	-0.026859658
-0.123232552	0.01053603	-0.078782189	0.102796793	-0.08300954
-0.093075761	0.053776776	0.011586168	0.810667829	0.045758134
0.298307829	2.75E-10	0.247295631	2.06E-07	-0.018442867
-0.175118007	0.000263274	0.058715651	0.224343972	0.050089743
-0.334943721	9.87E-13	-0.131725014	0.006228822	0.097880953
0.602546753	7.60E-44	0.33930706	4.79E-13	-0.085628554
-0.212972232	8.40E-06	-0.087295784	0.070546101	0.08433435

0.546038161	8.74E-35	0.303492051	1.30E-10	-0.020853857
-0.302134636	1.58E-10	-0.207818789	1.40E-05	0.138979719
0.209395282	1.20E-05	0.036393324	0.451617791	-0.115539279
0.35489638	3.29E-14	0.294629597	4.65E-10	-0.026809217
0.043504933	0.368153442	0.077508448	0.108496862	-0.089948624
0.360697242	1.17E-14	0.105359748	0.028924799	-0.019322738
0.496751072	3.55E-28	0.223160899	2.97E-06	0.024023354
0.313391055	2.97E-11	0.154591475	0.001301278	0.014280827
-0.27401975	7.62E-09	-0.185923219	0.000105307	0.049001482
-0.164782354	0.000602398	-0.065824827	0.173047259	0.073568127
0.303962405	1.21E-10	0.32405724	5.70E-12	0.010548749
-0.038254598	0.428801159	0.177263561	0.000220395	0.080750314
0.131268782	0.006412085	-0.105267635	0.029066063	0.052238475
-0.002770517	0.954319329	-0.069945723	0.147623405	0.084311972
-0.233504251	9.79E-07	-0.255027814	8.24E-08	0.045929196
-0.256454814	6.93E-08	-0.049141169	0.309314686	0.082643798
-0.453112193	3.70E-23	-0.278056021	4.49E-09	0.065579541
-0.192674969	5.78E-05	-0.032022079	0.507804012	0.012133812
-0.303878185	1.23E-10	-0.225178877	2.40E-06	0.048487372
0.10756293	0.025717291	0.090580145	0.060558974	0.027690018
0.319634137	1.14E-11	0.115673581	0.016406868	0.026515927
-0.150636051	0.001733507	-0.027445844	0.5703206	0.092469409
-0.328594269	2.77E-12	-0.172142451	0.000335743	0.102182092
-0.423494828	3.84E-20	-0.217233482	5.47E-06	0.055961854
0.287366775	1.28E-09	0.437438494	1.59E-21	-0.027054986
-0.439334483	1.02E-21	-0.31429172	2.59E-11	0.108079366
-0.267616114	1.74E-08	-0.159754485	0.000885861	0.122435614
-0.445286032	2.48E-22	-0.243602206	3.15E-07	0.181800207
0.184166143	0.000122666	0.101206145	0.035910828	-0.018342528
-0.253548724	9.84E-08	-0.072541351	0.133135854	0.092943129
0.27039698	1.22E-08	0.133942212	0.005403408	0.008259607
-0.376947413	5.76E-16	-0.138295609	0.004062991	0.022220846
-0.084330367	0.080683487	-0.213022579	8.36E-06	0.029642743
0.494850507	6.08E-28	0.298136308	2.82E-10	0.000932035
0.283466558	2.18E-09	0.218597533	4.76E-06	-0.015187092
0.430359306	8.16E-21	0.462075404	3.94E-24	0.023543103
0.204358575	1.95E-05	-0.044214959	0.360380963	0.012497965
-0.129311948	0.007254157	0.00389246	0.935854612	0.000399763
0.240723556	4.38E-07	0.090185777	0.061692758	-0.099213812
0.176161906	0.000241521	0.10470322	0.029944629	0.000610366
0.329876263	2.25E-12	0.24452628	2.84E-07	0.023796805
0.264511264	2.57E-08	0.183191044	0.000133426	-0.105448367
-0.351233283	6.26E-14	-0.091459359	0.058093152	0.091769068
-0.319082873	1.24E-11	-0.206724515	1.55E-05	0.055624004
-0.197412615	3.75E-05	0.172132656	0.000336009	0.102095583
0.599469264	2.64E-43	0.382173787	2.11E-16	0.03534496
0.644828892	6.59E-52	0.379651151	3.43E-16	-0.090521197
0.585586004	6.14E-41	0.29241141	6.35E-10	-0.042629607
0.300134376	2.12E-10	0.048818035	0.312510675	0.014716935
0.680960964	6.98E-60	0.530527949	1.38E-32	-0.073879614
0.678122729	3.25E-59	0.464695639	2.02E-24	-0.039144863

-0.402911937	3.25E-18	-0.111809098	0.020392344	0.121150696
0.269452324	1.37E-08	0.116259727	0.015866166	0.000121528
0.508554337	1.16E-29	0.297727544	2.99E-10	-0.017986424
-0.27725995	4.98E-09	-0.189862399	7.44E-05	-0.11232052
0.500583376	1.19E-28	0.37554593	7.52E-16	-0.057742636
-0.261387634	3.79E-08	-0.131960529	0.00613607	0.150031196
0.382932171	1.82E-16	0.138413414	0.004031318	0.042442583
0.486680113	5.93E-27	0.235056032	8.25E-07	-0.086236297
0.571798488	1.07E-38	0.434630477	3.06E-21	-0.048225184
0.480213329	3.45E-26	0.218542736	4.78E-06	-0.052376015
0.314370233	2.56E-11	0.223091156	2.99E-06	-0.027960567
0.280354533	3.31E-09	0.040517346	0.401983477	-0.009544256
0.331582841	1.71E-12	0.175677797	0.000251392	0.028020479
-0.211499959	9.73E-06	-0.057097555	0.237397122	0.044341903
0.628936614	9.89E-49	0.383323418	1.69E-16	-0.053510524
0.356634588	2.42E-14	0.332274477	1.53E-12	-0.028916744
0.288783723	1.05E-09	0.130726251	0.006636296	-0.101957423
0.245042861	2.67E-07	0.311909947	3.72E-11	-0.057719137
-0.377883696	4.82E-16	-0.199297342	3.15E-05	0.071966508
-0.159799905	0.000882824	-0.028148193	0.560492827	0.035633245
0.179865856	0.000177156	0.071928259	0.136454771	-0.094996375
-0.40089274	4.95E-18	-0.222080885	3.32E-06	0.126951921
0.108424442	0.024549128	0.062901265	0.192970728	0.073352052
0.214317512	7.34E-06	0.208608012	1.29E-05	-0.013880609
0.351880013	5.59E-14	0.104227898	0.030702062	-0.007196
0.271244865	1.09E-08	0.215332887	6.63E-06	0.03809087
0.192643852	5.80E-05	0.124215841	0.009929085	0.028794966
0.480974109	2.81E-26	0.30777663	6.91E-11	-0.020947633
-0.150046508	0.001808173	0.021838254	0.65156917	0.084668358
0.329419525	2.42E-12	0.067363296	0.1631989	-0.023627285
-0.391661887	3.24E-17	-0.116047423	0.016060179	0.186538607
0.042181757	0.382912877	-0.059483812	0.21832948	0.003954895
-0.207377072	1.46E-05	0.008487282	0.86069708	-0.026186568
-0.196227538	4.18E-05	-0.137973889	0.004150637	-0.060150489
0.055596593	0.24997522	-0.079346152	0.100350055	0.079878734
-0.068342449	0.157153994	0.009841357	0.838755493	-0.09144782
-0.535946763	2.42E-33	-0.370099002	2.09E-15	0.146623606
-0.084470635	0.080178756	0.038272696	0.428582624	-0.000284549
0.739065108	1.91E-75	0.452778863	4.02E-23	-0.082968893
0.264963735	2.43E-08	0.202640777	2.30E-05	-0.007121297
0.496793224	3.51E-28	0.400942384	4.90E-18	-0.115203901
0.22049094	3.92E-06	0.073072181	0.130312851	0.069617885
0.204622298	1.90E-05	0.022926489	0.635434128	-0.029519208
-0.067199413	0.164227492	-0.005146563	0.915255901	-0.054816295
0.17357965	0.000298687	0.162091152	0.000741527	0.081337832
0.175180911	0.000261913	-0.003679271	0.93936089	0.022788491
-0.025188284	0.60245202	-0.099159182	0.039850913	-0.115764388
0.20411397	2.00E-05	0.133842511	0.0054383	-0.061060994
-0.412900133	3.92E-19	-0.343049381	2.55E-13	0.143624667
-0.510959445	5.68E-30	-0.291638179	7.07E-10	0.006963922
-0.067402639	0.162952693	-0.029746786	0.538433814	0.120602702

0.673345087	4.16E-58	0.366840705	3.82E-15	-0.059475707
0.22909881	1.58E-06	0.147330599	0.002191664	0.010958775
0.068419669	0.156684563	-0.01906617	0.693396842	0.031651723
0.528984875	2.25E-32	0.297901754	2.92E-10	-0.047921571
-0.304098018	1.19E-10	-0.117990358	0.014359776	-0.011955209
0.303790366	1.24E-10	0.110559391	0.021849829	-0.183298357
0.264618227	2.53E-08	0.04895147	0.311188311	0.016571733
0.25825753	5.57E-08	0.209261241	1.21E-05	-0.016612828
0.18411235	0.000123238	0.126131543	0.008835077	-0.064819243
-0.166828964	0.000513252	-0.088224204	0.06759519	0.07758765
-0.038373305	0.427368998	-0.171583285	0.000351285	-0.070228866
0.52498791	7.89E-32	0.332931559	1.37E-12	0.011279895
0.246775874	2.19E-07	0.044204059	0.360499504	0.013983759
0.273778464	7.87E-09	0.21680888	5.71E-06	-0.115369405
0.5556487	3.32E-36	0.237895531	6.02E-07	-0.056525146
0.358465922	1.75E-14	0.099660678	0.038853275	-0.024902593
0.56355101	2.08E-37	0.340448698	3.96E-13	-0.026212566
0.109367893	0.023322694	0.087622456	0.069496014	0.169220019
0.103065835	0.03262317	0.090790616	0.059960959	0.047648112
0.229509979	1.51E-06	0.134414148	0.005240968	0.022381092
0.186181183	0.000102962	0.029106956	0.547210545	-0.029686641
0.452345497	4.47E-23	0.228111157	1.76E-06	-0.027117677
-0.079646325	0.099066755	0.068122993	0.158493906	0.071199924
0.093646091	0.052318931	-0.019884479	0.680945136	0.112925877
-0.338209417	5.75E-13	-0.112023236	0.020151273	0.050850302
0.095931609	0.046803187	-0.013436006	0.781153245	0.068243134
-0.273476615	8.18E-09	-0.164330574	0.000623921	0.026256902
-0.157710489	0.001032957	0.048845961	0.312233617	0.058858171
0.488471731	3.62E-27	0.260315333	4.33E-08	-0.051672019
-0.145453131	0.002498729	-0.094406901	0.050425593	-0.006094741
-0.380015084	3.20E-16	-0.183421522	0.000130806	0.110861888
-0.029318658	0.544298777	-0.151607422	0.001616622	0.037898328
0.181932725	0.000148622	0.004480209	0.926194633	-0.013387864
0.463122821	3.02E-24	0.466137797	1.40E-24	-0.046455347
0.142028748	0.003161586	0.073640431	0.127342384	-0.000622355
0.607693751	9.20E-45	0.343224599	2.48E-13	-0.058315619
0.577235332	1.44E-39	0.35844693	1.75E-14	-0.048325125
0.361111019	1.09E-14	0.10043744	0.037349818	0.00243219
0.034230972	0.47896399	-0.133237456	0.005654425	-0.063854447
0.611304002	2.04E-45	0.367708302	3.26E-15	-0.038059844
0.194066616	5.10E-05	0.112297301	0.019846357	-0.010840852
0.578329084	9.56E-40	0.362741368	8.09E-15	-0.009172763
0.316237444	1.93E-11	0.127529295	0.008105948	-0.012796913
0.168158101	0.000462095	0.073847783	0.126271637	0.060347347
0.100713318	0.036827855	0.007898922	0.870265466	0.01615768
-0.37435596	9.42E-16	-0.204566044	1.91E-05	0.061865043
0.372401888	1.36E-15	0.07261438	0.132744703	0.015097657
-0.158502059	0.000973506	-0.125451517	0.009210483	0.029442858
0.552025371	1.15E-35	0.253034937	1.05E-07	-0.037155505
-0.352920738	4.66E-14	-0.155328757	0.001232619	0.107403027
0.171974784	0.000340334	0.019357237	0.68895819	0.034255576

0.27052431	1.20E-08	0.032189362	0.50558857	-0.007543011
0.340063878	4.22E-13	0.123824816	0.010166629	-0.113782753
-0.09746378	0.043385434	-0.178749267	0.000194632	-0.023438809
0.67930424	1.72E-59	0.367736476	3.24E-15	-0.06698388
0.198796499	3.30E-05	0.200828008	2.73E-05	0.025663021
0.124923962	0.009511421	-0.061523591	0.202921129	-0.052052989
-0.053811333	0.265530535	0.09471851	0.049666819	-0.015834567
0.354389819	3.60E-14	0.18254126	0.000141081	-0.056607385
0.213889596	7.67E-06	0.197865573	3.60E-05	-0.082215029
0.451962291	4.91E-23	0.216040535	6.17E-06	-0.007315708
0.436345082	2.05E-21	0.154534744	0.001306704	-0.019989678
0.339497474	4.64E-13	0.200735767	2.75E-05	-0.032503677
-0.61856394	9.35E-47	-0.491064492	1.76E-27	0.023346388
0.155284716	0.001236625	0.158748326	0.000955665	0.069356508
-0.459429723	7.68E-24	-0.342271405	2.91E-13	0.184141833
-0.173699846	0.000295768	0.035174424	0.466923171	0.011194888
-0.019650315	0.684499617	0.142602256	0.003040479	0.012241976
0.689546024	5.98E-62	0.348051282	1.09E-13	-0.051636598
-0.133049459	0.005723133	0.079639256	0.099096824	0.024924151
-0.410733514	6.24E-19	-0.251831529	1.21E-07	-0.015729902
0.479643054	4.02E-26	0.251346765	1.28E-07	-0.038593459
-0.369296778	2.43E-15	-0.162390631	0.000724691	0.088130185
0.015764505	0.744448239	0.041172175	0.394413733	-0.047960265
0.323396402	6.33E-12	0.263138156	3.05E-08	0.006490373
0.557986845	1.48E-36	0.293281004	5.62E-10	0.042767571
0.37528148	7.91E-16	0.237193071	6.51E-07	-0.015227342
-0.022829829	0.636860479	-0.181363637	0.000156016	0.053716417
0.357694112	2.01E-14	0.129424401	0.007203212	-0.044236274
-0.320133348	1.05E-11	-0.133114864	0.005699145	0.063964219
-0.537143239	1.64E-33	-0.262610475	3.26E-08	0.099067831
0.623421324	1.14E-47	0.367185034	3.59E-15	-0.088269471
-0.380850775	2.72E-16	-0.217894003	5.11E-06	0.014477476
-0.151533204	0.001625291	-0.212984666	8.39E-06	-0.02625657
-0.119132701	0.013435703	0.04062416	0.400742829	0.126110828
0.469617691	5.68E-25	0.358603893	1.70E-14	-0.064587637
-0.214704254	7.06E-06	-0.175843668	0.000247968	0.049089011
-0.220898267	3.75E-06	-0.061838861	0.200611836	0.058372287
0.592531032	4.15E-42	0.284713799	1.84E-09	-0.119213187
0.668278629	5.90E-57	0.430573374	7.77E-21	-0.039691265
-0.127005777	0.008372502	-0.052880654	0.273897831	0.040142482
0.044496388	0.357328844	0.049060868	0.310106898	0.047760217
-0.08587444	0.075266981	-0.10135098	0.035645044	-0.090439208
-0.388118588	6.57E-17	-0.129567864	0.007138682	0.152373072
0.226608873	2.06E-06	0.257715481	5.95E-08	-0.031070668
0.607404391	1.04E-44	0.301808111	1.66E-10	-0.057624264
0.051582872	0.285862476	0.0186704	0.699449012	-0.033973739
-0.525924125	5.89E-32	-0.318466818	1.37E-11	0.10885216
-0.25313335	1.03E-07	-0.076751534	0.11200038	0.044054469
0.124236231	0.009916834	0.065383923	0.175949682	-0.025073386
0.057030557	0.237948892	0.027231848	0.573331164	0.00866901
0.417663238	1.39E-19	0.275917178	5.95E-09	-0.030291656

-0.520095996	3.59E-31	-0.356328102	2.56E-14	0.011562957
0.379833453	3.31E-16	0.330881139	1.91E-12	-0.068297191
-0.092277956	0.055872642	0.07384954	0.126262598	0.125776203
0.138663196	0.0039649	0.196303049	4.15E-05	0.050371878
-0.301151919	1.83E-10	-0.199052577	3.22E-05	0.080349985
0.108885548	0.023942929	0.063601515	0.188052209	0.055662651
0.08634832	0.073665261	0.05309709	0.27193611	-0.026479468
-0.248663156	1.75E-07	-0.108291789	0.024725959	-0.020964322
0.334961394	9.84E-13	0.212299446	8.99E-06	-0.00070028
0.363908761	6.54E-15	0.459695039	7.19E-24	-0.017063343
-0.213762971	7.76E-06	-0.09464357	0.049848423	0.066392537
-0.152659539	0.001498209	0.039216515	0.41727616	0.003944017
0.015467598	0.749098397	-0.090494807	0.060802843	-0.067618888
0.397539062	9.86E-18	0.321205676	8.92E-12	0.023008455
0.348249447	1.05E-13	0.227422753	1.89E-06	0.014035778
0.520178004	3.50E-31	0.542991654	2.41E-34	0.041205155
-0.279943776	3.49E-09	-0.134302262	0.005279077	0.110312505
0.258681248	5.29E-08	0.084376669	0.080516594	-0.048612569
-0.315175592	2.27E-11	-0.232833772	1.05E-06	-0.014248358
-0.183015829	0.000135451	-0.119212258	0.013373342	0.015464784
0.430120442	8.62E-21	0.191825741	6.24E-05	-0.054749974
0.109329586	0.023371439	-0.139418273	0.0037701	0.082931648
0.157924338	0.001016575	-0.04508568	0.350990653	-0.023321315
0.413242713	3.64E-19	0.200377267	2.84E-05	-0.021888739
0.418884943	1.07E-19	0.230944812	1.29E-06	-0.067715408
0.505907487	2.53E-29	0.240317689	4.58E-07	-0.050415378
-0.343919051	2.20E-13	-0.245499743	2.54E-07	0.073750264
0.508696613	1.11E-29	0.302391916	1.53E-10	-0.018407233
-0.121950884	0.011376405	0.00794448	0.869523869	-0.016097693
-0.298875892	2.54E-10	-0.122873994	0.010765403	-0.050797458
0.176855733	0.000228006	-0.05480385	0.256802446	-0.067016146
0.39585034	1.39E-17	0.187664521	9.04E-05	0.003862382
0.130823204	0.00659572	-0.038039227	0.431406625	0.007325973
0.397411489	1.01E-17	0.209461844	1.19E-05	-0.044652402
-0.370208006	2.05E-15	-0.138465439	0.004017402	0.093940435
0.332383492	1.50E-12	0.104772578	0.029835454	0.037911972
-0.317519906	1.58E-11	-0.119146627	0.013424768	0.114296907
0.357818601	1.96E-14	0.253953323	9.37E-08	-0.09774349
0.405360148	1.95E-18	0.514428731	2.01E-30	-0.018203732
0.216265111	6.03E-06	0.096588002	0.045312309	0.004429833
0.213783521	7.75E-06	0.161697108	0.000764231	-0.028211285
0.235574129	7.79E-07	0.183790112	0.000126715	-0.05899432
-0.042470185	0.379665181	0.066603048	0.168011602	-0.029955888
-0.209217542	1.22E-05	-0.057118451	0.237225215	0.070349887
0.548159921	4.29E-35	0.237218073	6.49E-07	-0.044192568
0.204681633	1.89E-05	-0.09400304	0.051423382	-0.013575043
-0.348641782	9.82E-14	-0.094532479	0.050118652	-0.013544294
-0.025517551	0.597715302	-0.11483898	0.017204576	0.034456655
0.104858113	0.029701287	0.050563683	0.295501912	0.006914998
0.328053947	3.02E-12	0.082510967	0.087466078	0.086226374
0.186928779	9.64E-05	0.046881596	0.332115783	-0.084511231

-0.282427209	2.50E-09	-0.142613042	0.003038242	0.020176911
0.730978306	4.90E-73	0.442528835	4.79E-22	-0.078388453
0.402916274	3.25E-18	0.12097105	0.012058325	-0.09200758
-0.545173449	1.17E-34	-0.343571103	2.34E-13	0.184736567
0.114651847	0.017388022	0.020546901	0.670928368	0.099053398
0.280501875	3.24E-09	0.183082367	0.000134678	0.029252793
0.393175771	2.39E-17	0.144849835	0.002605428	-0.115209965
-0.290494014	8.30E-10	-0.229423133	1.53E-06	0.015339034
0.160027534	0.000867747	0.012725138	0.792460692	-0.094985826
0.062055551	0.199035743	-0.123397325	0.010432083	0.127042083
-0.399973736	5.98E-18	-0.183753468	0.000127116	0.172358068
0.511233443	5.24E-30	0.212964965	8.41E-06	-0.062992861
0.496656818	3.65E-28	0.480960625	2.82E-26	-0.008884141
0.12446517	0.009780204	0.062816061	0.193575563	-0.051002836
-0.252851616	1.07E-07	-0.109039263	0.023743742	0.102841378
-0.297194937	3.23E-10	-0.078235731	0.105212461	0.086964029
-0.270959229	1.13E-08	-0.068656659	0.155250476	-0.027307431
0.019190993	0.691492038	-0.143347557	0.002889396	-0.035933693
0.213830961	7.71E-06	0.060547717	0.210191395	-0.023537232
0.29505116	4.38E-10	0.354468017	3.55E-14	0.025996474
0.518078295	6.65E-31	0.457691559	1.19E-23	-0.004229621
-0.406268654	1.61E-18	-0.236650451	6.92E-07	0.119759555
0.149462627	0.001885013	-0.041771444	0.387562122	-0.033041587
-0.116239192	0.015884841	0.061062919	0.206330066	0.032577674
-0.223855868	2.76E-06	-0.036656154	0.448354965	0.079390622
0.374085638	9.91E-16	0.323996014	5.76E-12	0.00706874
0.546701242	7.00E-35	0.389078482	5.43E-17	-0.038271802
0.54297313	2.43E-34	0.344235673	2.09E-13	-0.06171058
0.420196502	7.99E-20	0.130780489	0.006613569	0.035241788
0.688687946	9.69E-62	0.471576288	3.41E-25	-0.078372114
0.347773016	1.14E-13	0.179497774	0.000182748	-0.05197928
0.638276006	1.42E-50	0.351268536	6.22E-14	-0.006925038
0.056501322	0.242339168	-0.004814874	0.920698978	0.010199885
0.200099931	2.92E-05	-0.022663852	0.639312806	0.012926422
0.454826464	2.42E-23	0.275003063	6.70E-09	-0.089873076
0.12652094	0.0086263	-0.082771264	0.086468502	-0.102619704
-0.398957752	7.37E-18	-0.26095866	4.00E-08	0.092719296
0.224583385	2.55E-06	-0.015137501	0.754279052	-0.020297503
0.561149924	4.87E-37	0.343098555	2.53E-13	-0.081522599
0.197477343	3.73E-05	0.034962792	0.469609484	0.042331093
0.185886762	0.000105642	0.07426443	0.124141254	0.01911017
0.303250566	1.35E-10	0.117452482	0.0148139	0.087153619
-0.351717838	5.75E-14	-0.249539978	1.58E-07	-0.050621506
0.309910764	5.03E-11	0.132204206	0.006041409	-0.02152315
0.602588741	7.47E-44	0.324245	5.53E-12	-0.081073399
0.45524632	2.18E-23	0.427629853	1.52E-20	0.001250496
-0.185277041	0.000111403	0.052290909	0.279291923	0.067427636
-0.143720069	0.002816472	0.100866822	0.036540114	-0.053925179
0.116009866	0.016094716	0.075303748	0.11894885	0.018661327
-0.148020839	0.002087729	0.045291507	0.348793692	0.003563106
0.287044103	1.34E-09	0.114807578	0.017235241	0.137503966

0.810011895	2.94E-101	0.508302354	1.25E-29	-0.119804847
-0.216368633	5.97E-06	-0.007526316	0.876334985	0.060483835
0.559097982	1.00E-36	0.369209204	2.47E-15	-0.026677331
0.191872102	6.22E-05	0.125353399	0.009265803	-0.036215347
-0.48084675	2.90E-26	-0.360276629	1.26E-14	0.117140793
0.156267512	0.001150024	0.030322051	0.530603281	-0.064233836
0.05374213	0.26614661	-0.07473283	0.121779733	0.015026242
0.358446937	1.75E-14	0.215533385	6.50E-06	-0.064120341
0.15126995	0.001656385	0.116744656	0.015430702	0.043119711
0.124044035	0.010032842	0.039740159	0.411079474	0.059250625
0.422678897	4.61E-20	0.216260693	6.04E-06	-0.058209812
0.360969958	1.11E-14	0.276942407	5.20E-09	-0.021514439
0.089772685	0.062899092	-0.081755411	0.090414197	0.000159659
0.538209427	1.16E-33	0.280692412	3.16E-09	-0.02271796
0.156850646	0.001101317	0.08285413	0.086152852	0.046864821
0.537971489	1.26E-33	0.249967001	1.51E-07	-0.162233744
0.434035266	3.51E-21	0.341238422	3.46E-13	-0.037973473
0.285756502	1.59E-09	0.311023394	4.25E-11	0.004515601
0.033283967	0.491218094	-0.033744025	0.485244157	0.018661382
0.495133086	5.62E-28	0.294665861	4.62E-10	-0.07824003
-0.056012121	0.246447553	0.23345685	9.84E-07	0.050277926
-0.279413914	3.75E-09	-0.246663515	2.22E-07	0.077232657
-0.369372408	2.40E-15	-0.125248224	0.009325431	0.105706428
-0.413924436	3.14E-19	-0.104838594	0.029731858	0.06455385
0.200620524	2.78E-05	0.211105261	1.01E-05	-0.003886284
0.040193493	0.405759056	-0.031450333	0.515414159	0.049494452
0.400250462	5.65E-18	0.179560791	0.000181779	-0.035165032
-0.356085377	2.67E-14	-0.171933658	0.00034147	0.115448179
-0.30464751	1.10E-10	-0.112056528	0.020114018	0.130610536
0.536446026	2.06E-33	0.426978432	1.76E-20	-0.070024567
-0.167713158	0.000478668	-0.071363624	0.139567323	0.063283089
0.464854987	1.94E-24	0.341404077	3.37E-13	0.029349585
-0.165339991	0.000576782	-0.202636826	2.30E-05	-0.044176986
0.527309091	3.81E-32	0.317383842	1.61E-11	0.046696719
-0.261176109	3.89E-08	-0.160805071	0.000818025	0.010958815
0.283438683	2.18E-09	0.138724596	0.003948726	0.077764618
-0.241722512	3.91E-07	-0.176243377	0.000239896	-0.00836856
0.420373299	7.68E-20	0.176293675	0.000238898	-0.043576208
0.307244389	7.48E-11	0.101295804	0.035746098	-0.033947684
-0.242556204	3.56E-07	-0.090409059	0.061048705	0.087943032
0.036225928	0.453702836	-0.109409546	0.023269791	0.087005243
-0.258121266	5.66E-08	-0.029620159	0.540165199	0.077068816
0.393541163	2.22E-17	0.260698684	4.13E-08	-0.037161819
-0.170162296	0.000393851	-0.310697257	4.47E-11	0.010040142
-0.165684954	0.000561446	-0.057446835	0.234535193	-0.015277661
-0.518505605	5.84E-31	-0.342733152	2.69E-13	-0.003750694
-0.003333746	0.945046144	0.028632072	0.553769953	0.105734013
-0.35847841	1.74E-14	-0.276002048	5.88E-09	0.047058652
0.105197605	0.029173856	-0.080539962	0.095323416	0.021731834
-0.094352143	0.050559925	0.060043781	0.214018441	0.129998288
0.080973263	0.09354946	0.022635298	0.639735089	0.024162271

0.340524763	3.91E-13	0.239242652	5.18E-07	-0.028114242
-0.434739129	2.98E-21	-0.247373716	2.04E-07	0.097652321
-0.487299616	5.00E-27	-0.132819979	0.005808018	0.03167858
-0.427648181	1.51E-20	-0.202809037	2.26E-05	0.063157202
0.41403172	3.07E-19	0.307026263	7.72E-11	-0.000608279
0.030130896	0.533198892	0.017024831	0.724814047	-0.026386846
0.357103476	2.23E-14	0.15131708	0.001650778	0.010910576
0.469739922	5.50E-25	0.224985334	2.45E-06	-0.030341461
-0.344894577	1.87E-13	-0.158316187	0.000987176	0.074068408
0.263781555	2.81E-08	0.124347258	0.009850364	-0.02515687
-0.142665643	0.003027355	-0.018858607	0.69656852	-0.008724464
0.031607824	0.513312027	-0.079916833	0.097921489	0.009280842
0.270927956	1.14E-08	0.228707118	1.65E-06	0.042257644
-0.171634273	0.00034984	0.068835847	0.154172768	0.020603823
-0.551075759	1.60E-35	-0.36065626	1.18E-14	0.122104667
0.015242159	0.752635299	0.170275949	0.000390278	0.006470015
-0.283318559	2.22E-09	-0.219278393	4.44E-06	-0.04638809
-0.619833191	5.41E-47	-0.262066981	3.48E-08	0.118999807
-0.131905939	0.006157458	-0.028737331	0.55231274	0.001553701
0.198679505	3.33E-05	0.014008324	0.772082904	0.018706811
0.276131209	5.78E-09	0.175507586	0.000254951	-0.064054504
-0.042096084	0.383880812	0.068339162	0.157174004	-0.070574458
-0.076823255	0.111664637	-0.02447206	0.612813083	0.034033566
-0.015511807	0.748405419	-0.071406935	0.139326657	-0.079815954
-0.252819633	1.07E-07	-0.019446733	0.687595533	-0.011577839
-0.091166397	0.058905383	0.110175779	0.022314973	-0.044216586
0.48217635	2.03E-26	0.241170915	4.16E-07	-0.0158094
-0.14749531	0.002166442	-0.111420831	0.02083584	0.044424765
-0.275036174	6.68E-09	-0.166529407	0.000525486	0.064902833
0.085854292	0.075335706	0.047886332	0.32184672	-0.050524991
0.250685196	1.38E-07	0.217791721	5.17E-06	-0.050421047
0.582910602	1.70E-40	0.514897739	1.75E-30	-0.016142548
-0.148649284	0.001997037	-0.095267499	0.048353236	0.14111425
-0.172713659	0.000320529	-0.150386762	0.001764728	0.029312268
0.40837702	1.03E-18	0.211272935	9.95E-06	0.010973712
0.024471972	0.61281436	-0.136585898	0.004548693	-0.027306611
-0.164792847	0.000601906	0.036364217	0.451979946	-0.022261155
0.096726927	0.045001935	-0.003444185	0.943228669	0.104852758
0.225579102	2.30E-06	0.200732389	2.75E-05	0.005907596
-0.008631231	0.858359108	0.040690903	0.39996877	0.043455287
0.28437738	1.92E-09	0.157522312	0.001047574	0.006622724
-0.205339947	1.78E-05	-0.015905445	0.742244079	0.045512642
0.329898302	2.24E-12	0.120045147	0.012735634	-0.024559664
0.165748945	0.000558643	0.117264911	0.014975205	0.074248364
0.188348247	8.51E-05	0.021267517	0.660097447	-0.036539699
0.074016297	0.125406606	0.043416808	0.369125327	-0.003000305
0.18693129	9.64E-05	0.079296247	0.100564685	-0.042116073
0.083066092	0.085349669	0.113534444	0.01851938	0.063031038
0.02946769	0.542253595	-0.03848488	0.426025415	0.023384639
0.096225435	0.046130806	0.093636023	0.052344377	0.000473816
0.345167319	1.78E-13	0.098383499	0.041436613	-0.024179812

0.191640138	6.35E-05	0.142176063	0.003130067	-0.084879132
0.130295911	0.00681911	-0.105991581	0.027971611	-0.000964521
0.105161534	0.029229511	0.110073189	0.02244081	-0.009406744
-0.420425218	7.60E-20	-0.191018555	6.71E-05	0.062428722
0.068264908	0.157626447	0.015863389	0.742901569	0.086840476
0.251643613	1.23E-07	0.168453518	0.000451389	0.005007979
0.161462579	0.000778048	0.045745836	0.343975165	0.039609423
0.442162141	5.22E-22	0.122163822	0.011232803	-0.040388059
0.38708466	8.06E-17	0.181539773	0.000153691	-0.138348655
0.439305135	1.03E-21	0.137298303	0.004340273	-0.004122527
0.37732522	5.36E-16	0.213034935	8.35E-06	0.095922383
-0.138016074	0.004139048	-0.040754036	0.399237407	-0.015476841
-0.227018581	1.97E-06	-0.110821095	0.02153735	0.128737387
-0.467271579	1.04E-24	-0.177468452	0.000216661	0.065692536
0.229785874	1.47E-06	0.140162236	0.003586686	0.056477282
-0.316456983	1.86E-11	-0.124924494	0.009511113	0.104396224
0.508606185	1.14E-29	0.341082939	3.56E-13	-0.066947001
0.367692793	3.27E-15	0.214530591	7.19E-06	-0.001733662
-0.540672106	5.19E-34	-0.302345776	1.54E-10	0.150912407
0.414098395	3.03E-19	0.373516025	1.10E-15	0.030200628
0.032871788	0.496603576	0.153861527	0.001372703	0.150812386
0.4391378	1.07E-21	0.262187063	3.43E-08	0.024199946
-0.203580568	2.10E-05	-0.331206284	1.82E-12	0.00354737
-0.449137502	9.78E-23	-0.212768711	8.58E-06	0.128136199
0.444952109	2.69E-22	0.255311019	7.96E-08	-0.089308001
0.420146241	8.08E-20	0.255730215	7.57E-08	0.047337958
-0.318637147	1.33E-11	-0.126596399	0.008586354	0.094130628
0.257641562	6.00E-08	0.05068107	0.294380754	0.000750241
0.400752403	5.09E-18	0.321802061	8.13E-12	0.010270382
0.124532663	0.009740245	0.033103556	0.493571478	0.031917251
-0.1504587	0.001755666	0.101872507	0.034701805	0.016341599
-0.033402982	0.489668904	-0.110318044	0.02214148	-0.052946228
0.126385287	0.008698527	0.041869753	0.386445075	-0.022207159
-0.283771948	2.09E-09	-0.134624782	0.005169897	0.090998582
0.517467397	8.01E-31	0.265944592	2.15E-08	-0.008030897
0.511534771	4.79E-30	0.188761202	8.21E-05	-0.020786719
0.451666571	5.28E-23	0.224194113	2.66E-06	0.057256958
0.239525806	5.01E-07	0.09639579	0.045744696	0.21703886
-0.239955325	4.78E-07	0.005035685	0.917075006	0.112036679
0.3512303	6.27E-14	0.133927165	0.005408661	-0.00754508
0.649451961	7.22E-53	0.354771425	3.37E-14	-0.047213462
0.309098329	5.67E-11	0.199376653	3.12E-05	-0.01121286
0.590983873	7.61E-42	0.33330355	1.29E-12	-0.018731866
-0.076380746	0.113748785	-0.038328666	0.427907226	-0.035022094
0.417356547	1.49E-19	0.177217701	0.000221239	-0.046636648
0.27145399	1.06E-08	0.152935273	0.001468521	-0.029104858
-0.528241305	2.84E-32	-0.338139046	5.82E-13	0.059518149
-0.243820191	3.08E-07	-0.119063869	0.013489862	0.057052268
0.268419323	1.57E-08	0.070007088	0.147267569	0.078952174
0.220353375	3.97E-06	0.142886093	0.002982114	0.002833174
-0.275398558	6.37E-09	-0.069308752	0.151355752	0.108660485

0.238150464	5.85E-07	0.113578063	0.01847404	-0.01073256
-0.166311543	0.000534553	-0.052227309	0.27987791	-0.016002218
-0.322942407	6.80E-12	-0.081687535	0.090682898	0.074626208
-0.257318285	6.24E-08	-0.049516459	0.305629957	-0.049926662
0.416910134	1.64E-19	0.140693935	0.003460601	-0.040258556
-0.034991194	0.469248473	0.071302753	0.139906099	-0.040403194
-0.300494063	2.01E-10	-0.133958114	0.005397862	0.03788151
0.097871627	0.042511914	-0.031829297	0.510363427	0.05037788
0.297463092	3.11E-10	0.13655244	0.004558699	-0.022589562
0.222359872	3.22E-06	0.193453788	5.39E-05	0.051965853
0.418059966	1.28E-19	0.12112867	0.011946249	-0.03938717
-0.158338376	0.000985535	-0.021167487	0.661596732	0.059633707
0.186697883	9.84E-05	0.254804609	8.46E-08	0.111094734
-0.027023626	0.576267689	-0.048158388	0.319102041	0.046085909
-0.480523664	3.17E-26	-0.292428704	6.33E-10	0.160001825
0.045963081	0.341686141	-0.079186201	0.10103925	0.081397199
0.461471828	4.59E-24	0.243671636	3.13E-07	-0.017956458
0.298795259	2.57E-10	0.018030266	0.709278289	0.013857593
0.37867462	4.14E-16	0.124237313	0.009916185	-0.019407075
-0.202514352	2.33E-05	-0.090116041	0.061895056	0.042049023
0.776024565	1.05E-87	0.469645479	5.64E-25	-0.08504545
0.619593862	6.00E-47	0.30928722	5.52E-11	-0.024170745
0.398026481	8.93E-18	0.158682603	0.000960397	-0.050911182
-0.354653164	3.44E-14	-0.173406496	0.00030294	0.060980314
0.28929445	9.80E-10	0.160568278	0.000832881	0.012518139
-0.148165685	0.002066498	-0.056429669	0.242937896	0.075078193
0.247107156	2.10E-07	0.097960873	0.042322757	-0.100343482
0.192098272	6.09E-05	0.008297726	0.863777639	-0.005359084
0.204792537	1.87E-05	0.223757321	2.79E-06	0.057846527
0.101587925	0.035213835	0.02161583	0.654887429	-0.172176747
0.459219439	8.10E-24	0.463727907	2.59E-24	0.001492262
0.49240476	1.21E-27	0.172818361	0.000317812	-0.036030956
0.582718776	1.83E-40	0.546003048	8.85E-35	-0.03801284
0.521846632	2.09E-31	0.406430442	1.56E-18	-0.067129211
-0.098633458	0.040919914	0.07598013	0.115661842	0.0655619
-0.059107441	0.221261777	-0.083653259	0.083156202	-0.038060245
-0.353348206	4.33E-14	-0.025741277	0.594506512	0.065231105
-0.297694559	3.01E-10	-0.225346572	2.36E-06	0.017346942
-0.157216704	0.00107172	-0.090158052	0.061773121	0.068614502
0.514135402	2.20E-30	0.460638882	5.67E-24	-0.041323429
-0.065446954	0.17553255	-0.146319972	0.002352385	0.051703452
-0.049840774	0.302469171	-0.012412771	0.797443309	0.068694079
0.385831504	1.03E-16	0.256859249	6.60E-08	-0.032562684
0.523104082	1.42E-31	0.333226392	1.31E-12	-0.013728519
-0.529294047	2.04E-32	-0.257132259	6.38E-08	0.06942561
-0.150738823	0.001720784	0.091527804	0.057904731	0.045428163
0.132753956	0.005832648	0.103014506	0.03271034	0.004051638
-0.286050844	1.53E-09	-0.040884819	0.397724892	0.031821569
0.178080765	0.000205854	0.129690425	0.007083964	-0.018536965
0.274607038	7.06E-09	0.163861336	0.000647028	-0.060185949
0.590912957	7.82E-42	0.483049345	1.60E-26	-0.083387838

0.183777976	0.000126848	0.056605965	0.241466623	0.058135576
0.214768444	7.02E-06	0.027126004	0.574822987	0.000835318
0.384871934	1.25E-16	0.16327736	0.000676893	-0.068192806
0.344945692	1.85E-13	0.178762216	0.00019442	0.001227766
-0.35673487	2.38E-14	-0.195507038	4.47E-05	0.044713459
0.041235641	0.393684676	-0.002085667	0.965603114	-0.003271286
0.174651655	0.000273572	-0.033170306	0.492700045	0.065018899
0.441511542	6.10E-22	0.171126355	0.000364481	-0.024068621
-0.044855853	0.353454087	-0.042988171	0.373875187	-0.080080302
-0.547272819	5.78E-35	-0.312202759	3.56E-11	0.097397073
0.36012136	1.30E-14	0.148529934	0.002013979	0.036080511
0.290516325	8.27E-10	0.157237604	0.001070053	-0.018076486
0.226455086	2.10E-06	0.220534736	3.90E-06	0.066962904
0.360015234	1.32E-14	0.259830676	4.59E-08	0.005302939
0.163260676	0.000677765	-0.004441732	0.926826711	0.024745347
0.3212695	8.83E-12	0.128905547	0.007440971	-0.04106983
0.001807899	0.970181779	-0.066853313	0.166415728	0.066400469
0.263386466	2.96E-08	0.248151337	1.86E-07	0.044977496
-0.142079514	0.003150692	0.211024992	1.02E-05	0.0722391
0.658383661	9.04E-55	0.361417623	1.03E-14	-0.070580785
-0.035970822	0.45689075	-0.132587182	0.005895283	0.032342018
-0.394014111	2.02E-17	-0.143886925	0.002784354	0.119602429
0.192904338	5.66E-05	0.121518058	0.011673309	-0.013269633
0.181092727	0.000159655	0.127265682	0.008239207	0.065458689
-0.055716459	0.248954018	-0.01210162	0.802414729	0.061621521
0.643560299	1.20E-51	0.36901509	2.56E-15	-0.055090915
-0.252962047	1.06E-07	0.15960857	0.000895683	0.019639295
0.13183666	0.006184697	0.30883792	5.90E-11	-0.055541927
0.364052163	6.37E-15	0.212980897	8.40E-06	-0.061169596
0.118128186	0.014245397	0.166292995	0.000535331	-0.001043136
-0.271532839	1.05E-08	-0.158991136	0.00093837	-0.02292722
0.542317106	3.02E-34	0.374805939	8.65E-16	-0.044931942
0.300198052	2.10E-10	0.067870714	0.16004485	0.00817328
0.153918906	0.00136696	0.070072934	0.146886479	0.034648292
-0.17615038	0.000241752	-0.047264	0.328182758	-0.077555299
0.674220326	2.62E-58	0.430455787	7.98E-21	-0.072509354
-0.191735963	6.29E-05	-0.069600149	0.149639511	-0.011424417
-0.415894401	2.05E-19	-0.22238373	3.22E-06	0.129756216
-0.258273335	5.56E-08	-0.147307207	0.002195268	0.013808275
-0.2251747	2.40E-06	-0.140427063	0.003523375	0.133910693
-0.168774131	0.00044003	0.004212602	0.930591635	0.074408197
-0.047455207	0.326227537	-0.064299909	0.183239162	-0.02501087
0.129459485	0.007187383	0.211876872	9.37E-06	-0.054726573
0.185518839	0.000109084	0.112785223	0.019313437	-0.047318574
0.542409085	2.92E-34	0.344070326	2.15E-13	-0.018590043
0.471489519	3.49E-25	0.150764787	0.001717584	-0.014769773
-0.246019034	2.39E-07	0.081319219	0.092152127	0.070458593
-0.506593263	2.07E-29	-0.277256383	4.99E-09	0.096523574
-0.102841782	0.033005134	0.001653176	0.972732638	-0.011292778
0.744509852	4.07E-77	0.442860263	4.43E-22	-0.047910736
0.207345428	1.46E-05	-0.018377974	0.70393312	-0.024490559

0.083028316	0.085492372	-0.018935434	0.695393938	0.003366417
-0.130242423	0.006842144	-0.014712534	0.760964858	0.004723422
-0.174962517	0.000266667	-0.00120564	0.980112457	0.072016047
0.14229945	0.003103887	0.062911397	0.1928989	0.00040259
-0.016956167	0.72587924	0.195130789	4.63E-05	-0.070956972
0.485720128	7.72E-27	0.327619496	3.24E-12	-0.118087693
0.274793334	6.89E-09	0.192987946	5.62E-05	-0.005644987
-0.118102937	0.01426629	-0.008339498	0.863098601	0.040258349
0.293986257	5.09E-10	0.175738213	0.00025014	-0.018803559
0.190937574	6.76E-05	0.092383807	0.055590725	-0.077918432
-0.331341187	1.78E-12	-0.166567276	0.000523924	0.040584948
0.361083961	1.09E-14	0.086199187	0.074166312	-0.004963338
-0.309199269	5.59E-11	-0.105691235	0.028421298	0.079752748
-0.393426167	2.27E-17	-0.086246343	0.074007581	0.004412751
0.536023691	2.36E-33	0.344446619	2.01E-13	-0.053202478
0.482064462	2.09E-26	0.277997262	4.52E-09	-0.026150699
-0.213041998	8.35E-06	-0.129008544	0.007393223	-0.017747377
0.241759764	3.89E-07	0.274430383	7.23E-09	-0.01605374
0.371632971	1.57E-15	0.307580137	7.11E-11	-0.017888644
0.734720789	3.86E-74	0.483764991	1.32E-26	-0.152180941
-0.143808372	0.002799433	-0.103308186	0.032214263	0.067245452
0.253453461	9.95E-08	0.197345182	3.77E-05	0.014839545
-0.051739388	0.28440113	0.048822153	0.312469813	-0.066896815
0.151818523	0.001592196	0.14074706	0.003448226	-0.050766283
-0.597085812	6.85E-43	-0.336371899	7.80E-13	0.147797465
-0.264404332	2.60E-08	-0.172711349	0.00032059	-0.042011154
0.594700583	1.77E-42	0.329682457	2.32E-12	-0.089904999
-0.486336481	6.52E-27	-0.289279802	9.82E-10	0.025777873
0.149649255	0.001860133	-0.038036435	0.431440463	0.065921662
0.236989683	6.66E-07	0.175454296	0.000256074	-0.012638823
0.139369006	0.003782541	0.028359377	0.557553854	-0.028719719
0.631793366	2.74E-49	0.550586175	1.88E-35	-0.085453052
0.318951142	1.27E-11	0.040939795	0.397090127	0.034975579
-0.386816268	8.50E-17	-0.26594967	2.14E-08	0.0998593
0.405608668	1.85E-18	0.186554539	9.97E-05	-0.035804171
-0.334235216	1.11E-12	-0.152126681	0.001557148	0.065319673
0.231604235	1.20E-06	0.228436411	1.70E-06	-0.010158805
0.596991005	7.12E-43	0.345867745	1.58E-13	-0.066643105
0.119046004	0.013503951	0.009250055	0.848322701	-0.030210352
-0.172916878	0.000315274	-0.043535134	0.367820743	-0.095137673
0.18987694	7.43E-05	0.134381861	0.00525194	-0.028529386
0.157706366	0.001033276	-0.045187168	0.349906298	-0.01487455
-0.244538693	2.83E-07	-0.069652973	0.149329983	0.048880439
-0.031483032	0.514977346	-0.14383486	0.00279434	0.048859964
-0.302833339	1.43E-10	-0.182501813	0.000141559	0.050609178
0.41982021	8.68E-20	0.553517163	6.93E-36	0.00941166
0.04375375	0.365417946	-0.109236775	0.023489905	0.063753727
-0.08323852	0.084700752	-0.18224404	0.000144718	0.079398917
0.070630877	0.143687421	0.01246351	0.7966334	-0.053818742
-0.468200232	8.21E-25	-0.223270829	2.93E-06	0.118564088
-0.134815694	0.005106232	0.058244144	0.228093686	-0.014242013

-0.102666364	0.033306844	0.09800188	0.042236082	0.082075682
-0.242178479	3.71E-07	-0.20776796	1.40E-05	-0.023285762
-0.257160995	6.36E-08	-0.04707716	0.33010063	0.062144608
0.483967633	1.25E-26	0.222671139	3.12E-06	-0.029378646
-0.233250069	1.01E-06	-0.135045163	0.005030644	-0.045219141
0.253544261	9.84E-08	0.126265765	0.008762611	-0.098550354
0.09579742	0.047112984	0.017756347	0.71349923	0.02013939
0.388142773	6.54E-17	0.16903066	0.000431134	-0.004522758
-0.408748191	9.53E-19	-0.283628695	2.13E-09	0.061302221
0.429580931	9.74E-21	0.170360414	0.000387641	-0.047970061
0.016144391	0.738512013	0.136626713	0.004536512	0.002145506
0.425945265	2.22E-20	0.346092664	1.52E-13	0.004693091
0.536253466	2.19E-33	0.251827998	1.21E-07	-0.059141535
0.06494646	0.178865046	-0.022840558	0.636702081	0.005820445
0.119628834	0.013050955	0.014969453	0.756920708	-0.006317476
0.127033391	0.008358249	0.137463591	0.004293167	0.100003882
-0.350099066	7.63E-14	-0.159230873	0.000921577	0.046962912
0.588791935	1.79E-41	0.457977139	1.11E-23	-0.040000972
0.184573394	0.000118419	0.13359812	0.005524685	0.077569426
0.44741269	1.49E-22	0.247448452	2.02E-07	-0.039006577
-0.301443919	1.75E-10	-0.180742175	0.000164483	0.145931279
0.205246962	1.79E-05	0.177082095	0.000223751	0.049555264
0.454618066	2.55E-23	0.157137518	0.00107806	0.001875244
-0.06105737	0.206371377	0.100330097	0.0375546	-0.047790603
0.700086877	1.37E-64	0.406890101	1.41E-18	-0.048600116
-0.168052882	0.000465965	-0.024758909	0.608654034	0.043274724
0.470452308	4.57E-25	0.209032649	1.24E-05	-0.031476352
-0.417880569	1.33E-19	-0.211263178	9.96E-06	0.158540634
0.330171387	2.15E-12	0.17694452	0.000226328	-0.044571894
-0.169382618	0.000419201	-0.072717215	0.132195414	-0.034523283
0.262332893	3.37E-08	0.058760343	0.223990842	-0.00580568
-0.311181876	4.15E-11	-0.191131684	6.64E-05	0.019552363
0.13226477	0.006018087	0.188257386	8.58E-05	0.059697036
0.526712628	4.60E-32	0.354973297	3.25E-14	-0.054998101
-0.018351821	0.704334653	0.150327831	0.001772184	0.028468738
0.0853414	0.077102403	-0.073607638	0.127512364	0.039386499
0.155943633	0.001177926	0.062650345	0.194755906	0.061092907
-0.220040774	4.10E-06	-0.183726443	0.000127413	0.029756199
-0.054772767	0.257072741	-0.084586798	0.079762707	0.064433287
0.299025007	2.48E-10	0.171181969	0.00036285	-0.052591597
-0.023091762	0.632998434	0.041324791	0.392661942	-0.064817213
-0.477356244	7.41E-26	-0.322750446	7.00E-12	-0.028384719
-0.335674903	8.75E-13	-0.297819249	2.95E-10	0.067986707
0.500770036	1.12E-28	0.224016321	2.71E-06	-0.059104337
-0.086849205	0.072002573	0.0403998	0.403351438	0.033753903
0.151609841	0.00161634	0.061388652	0.20391541	0.056980525
-0.404503238	2.33E-18	-0.159173164	0.000925594	0.096892535
-0.041950578	0.385528157	-0.127772345	0.00798479	-0.021226819
-0.233114836	1.02E-06	-0.050714561	0.294061403	0.039432503
-0.391508063	3.34E-17	-0.224308872	2.63E-06	0.125215416
-0.278810999	4.06E-09	0.029279766	0.544833127	-0.013092694

-0.382020025	2.17E-16	-0.252278506	1.14E-07	0.102420587
-0.077965309	0.10642438	0.132678321	0.00586098	0.051250631
0.501285751	9.69E-29	0.298272462	2.77E-10	-0.032088393
-0.143263468	0.002906094	0.024437858	0.61330981	-0.026274148
0.254254163	9.04E-08	0.084602934	0.079705049	0.190999797
0.465277074	1.74E-24	0.159774536	0.000884519	-0.022066638
0.076478473	0.113285903	-0.00908387	0.851015633	0.04516883
0.666377134	1.57E-56	0.441655522	5.89E-22	-0.036732767
0.059763678	0.216167166	-0.133357077	0.005611094	0.111534708
0.143328131	0.002893246	0.016019712	0.740458597	0.085780996
-0.309978759	4.97E-11	-0.102819668	0.033043041	0.051168471
0.363243757	7.39E-15	0.060421264	0.211147048	-0.032455808
-0.282469608	2.49E-09	-0.00712314	0.882910632	-0.001828927
0.306127971	8.82E-11	0.156796449	0.001105762	-0.037949127
0.42954533	9.82E-21	0.280094488	3.42E-09	0.006793407
0.182737689	0.000138725	0.120926352	0.012090276	0.011225574
0.82464939	5.73E-108	0.598166899	4.45E-43	-0.115711977
0.277889483	4.59E-09	0.010452206	0.828896902	0.009142302
-0.178357745	0.000201133	-0.162826655	0.00070081	-0.022187891
-0.14023205	0.003569897	0.086912157	0.071795786	-0.058192486
0.747107407	6.26E-78	0.492534665	1.17E-27	-0.103827197
0.424224407	3.26E-20	0.278529343	4.21E-09	-0.090938054
0.175309196	0.000259158	-0.067804015	0.160456802	0.167626255
-0.017051254	0.724404281	0.045758026	0.343846462	0.013133349
0.363895303	6.56E-15	0.284064171	2.01E-09	0.008491843
-0.521650898	2.22E-31	-0.381737437	2.29E-16	0.115462745
-0.205369478	1.77E-05	-0.127633214	0.008053946	-0.072301956
0.054376045	0.260539871	0.022606504	0.640161047	0.012793104
0.23595422	7.47E-07	0.207072349	1.50E-05	0.067386052
-0.293063967	5.79E-10	-0.085929683	0.075078813	0.09775845
0.354083935	3.80E-14	0.230380478	1.38E-06	-0.053110348
0.564469598	1.50E-37	0.44317043	4.11E-22	-0.021633378
-0.37498814	8.36E-16	-0.254072705	9.24E-08	0.07960603
0.402475544	3.56E-18	0.335727142	8.67E-13	-0.018264089
-0.110410053	0.022029898	-0.076407852	0.113620248	-0.057495485
0.341552803	3.29E-13	0.184773561	0.000116382	0.001113088
0.253617166	9.76E-08	0.234113281	9.16E-07	0.009717915
0.408929559	9.17E-19	0.178470906	0.000199233	0.010473552
-0.253286012	1.01E-07	-0.093638473	0.052338184	0.132674511
-0.418259699	1.22E-19	-0.175789264	0.000249086	0.032023356
0.111383064	0.020879423	-0.064620744	0.18105884	0.059290987
-0.322318766	7.49E-12	-0.169024868	0.000431333	-0.028014865
-0.269785752	1.32E-08	-0.059332296	0.219506573	0.106187148
0.203598969	2.10E-05	0.180410614	0.000169175	-0.086126132
0.052191465	0.280208529	-0.118779923	0.013715321	-0.028835282
-0.043586969	0.367250145	0.100656352	0.036935126	-0.081638023
-0.431321264	6.55E-21	-0.140816458	0.003432122	-0.027383827
-0.249300501	1.63E-07	-0.147495335	0.002166438	0.099381504
0.104000958	0.03106944	0.055611861	0.249844986	0.022572817
0.060796524	0.208320179	-0.002363451	0.961025317	0.028987337
0.313019058	3.15E-11	0.069975766	0.147449114	-0.025283889

0.292080636	6.65E-10	0.111948449	0.020235184	0.027958869
-0.220837725	3.78E-06	-0.076960786	0.11102304	0.106835859
-0.143299177	0.002898992	-0.12426396	0.009900196	-0.023530449
-0.140561616	0.003491597	-0.186169419	0.000103068	0.115087511
0.538301794	1.13E-33	0.360621894	1.19E-14	-0.000777516
0.087653395	0.069397227	0.09790018	0.042451317	-0.041741828
-0.292425093	6.34E-10	-0.163972211	0.000641497	0.089919464
0.369476199	2.35E-15	0.262461415	3.32E-08	0.064787091
-0.316127706	1.96E-11	-0.097809139	0.04264478	0.149105017
0.524744152	8.51E-32	0.279590182	3.66E-09	-0.021052474
0.168734862	0.000441407	-0.024097861	0.618257326	-0.062522986
0.532345935	7.72E-33	0.340434285	3.97E-13	-0.045901469
0.267593163	1.74E-08	0.143140837	0.002930602	0.008127745
0.570818458	1.53E-38	0.456689727	1.53E-23	-0.039413524
0.180437469	0.00016879	0.025678544	0.595405469	0.103327985
0.215873536	6.28E-06	0.213578279	7.91E-06	-0.014219015
0.303167799	1.36E-10	0.184466074	0.000119524	-0.016732682
0.435271465	2.63E-21	0.19194506	6.18E-05	0.006782983
-0.181192333	0.000158308	-0.216077146	6.15E-06	0.014910111
-0.397991602	8.99E-18	-0.283389396	2.20E-09	0.00549875
0.375933496	6.99E-16	0.22570326	2.27E-06	-0.055585969
0.186450303	0.000100568	0.081974577	0.089550937	-0.01941729
0.117270407	0.014970457	0.157089864	0.001081892	0.032526431
-0.458756679	9.10E-24	-0.185327041	0.00011092	0.105571814
-0.246439707	2.27E-07	-0.076334047	0.113970499	0.128023613
-0.316321319	1.90E-11	-0.2350013	8.30E-07	0.09897823
-0.268513911	1.55E-08	-0.064705707	0.180484679	-0.029843339
-0.350983434	6.54E-14	-0.220030177	4.11E-06	0.008019526
0.336594518	7.52E-13	0.212572202	8.75E-06	0.025217976
0.499578176	1.58E-28	0.248959735	1.69E-07	-0.029615976
-0.393089626	2.43E-17	-0.227089055	1.96E-06	0.121948752
-0.19335344	5.44E-05	0.070504396	0.144407913	0.065631375
0.524805877	8.35E-32	0.256076604	7.26E-08	-0.100044618
0.442870947	4.41E-22	0.318888928	1.28E-11	-0.039580584
0.108766659	0.024097977	0.059961784	0.214645863	-0.043786469
-0.309495706	5.35E-11	-0.192212116	6.03E-05	0.128098175
0.011442551	0.812971113	-0.085685889	0.075912114	0.00136816
0.0452304	0.349445029	-0.006521787	0.892733302	0.063781554
-0.382276504	2.07E-16	-0.130133136	0.006889426	0.073218171
0.574988974	3.31E-39	0.505347046	2.98E-29	-0.048906983
0.368428992	2.85E-15	0.304023832	1.20E-10	0.008873828
-0.375717134	7.28E-16	-0.163709047	0.000654696	0.078496353
-0.177028264	0.000224756	-0.20609176	1.65E-05	0.006474096
-0.133388985	0.005599587	-0.06216588	0.198236742	0.002455939
-0.129755211	0.007055192	-0.001842264	0.969615268	-0.053746018
0.162615145	0.000712302	0.101162786	0.035990721	-0.026577163
-0.301604364	1.71E-10	-0.122142944	0.011246812	0.074248055
0.203930912	2.03E-05	0.27159785	1.04E-08	0.007386237
0.376711505	6.02E-16	0.257224955	6.31E-08	-0.055226052
0.656721042	2.07E-54	0.291961992	6.76E-10	-0.104075869
0.29215352	6.58E-10	0.263287434	2.99E-08	-0.046862652

0.253566808	9.81E-08	0.236316624	7.18E-07	-0.016451042
0.1435761	0.002844455	0.093315556	0.053159759	0.112008087
-0.102215341	0.034093395	0.04802369	0.320459047	0.005348788
0.311618467	3.89E-11	0.153443034	0.00141526	0.005303759
0.515477373	1.46E-30	0.287415418	1.27E-09	-0.052517067
-0.028996047	0.548739052	0.192829695	5.70E-05	0.023991263
-0.309249941	5.55E-11	-0.065056669	0.178127232	0.03776868
0.456035968	1.79E-23	0.198148486	3.50E-05	-0.058913577
0.509280663	9.36E-30	0.519373949	4.48E-31	0.001752333
0.374160869	9.77E-16	0.267736079	1.71E-08	-0.027460313
0.378496762	4.28E-16	0.113787472	0.018257717	-0.013124316
0.406544439	1.52E-18	0.225204255	2.39E-06	-0.032095241
-0.287711774	1.22E-09	-0.08296705	0.085724215	0.078840963
-0.128653764	0.007558855	-0.09051558	0.060743406	0.109953696
-0.111622254	0.020604731	-0.007039111	0.884282153	0.017278119
0.404847231	2.17E-18	0.243889639	3.05E-07	0.032319798
-0.152291331	0.001538713	0.175255238	0.000260314	-0.058729028
0.335467472	9.05E-13	0.272113713	9.76E-09	-0.002088819
0.372042472	1.46E-15	0.117702162	0.014601553	-0.02082736
0.117328533	0.01492032	-0.034034956	0.481486679	0.014081383
0.003686607	0.939240227	-0.1742664	0.000282362	0.065280258
-0.508376222	1.22E-29	-0.292994439	5.85E-10	0.082906863
0.377063442	5.63E-16	0.305455046	9.74E-11	-0.010084532
-0.178635914	0.000196494	-0.200494694	2.81E-05	0.06174654
-0.202618381	2.30E-05	-0.064727383	0.180338416	0.027469406
0.339200686	4.87E-13	0.182164507	0.000145707	-0.101673011
0.335944496	8.37E-13	0.257292025	6.26E-08	-0.000970898
0.05181324	0.283713341	0.002589869	0.95729494	0.039269848
-0.429187374	1.07E-20	-0.284404946	1.92E-09	0.00897059
-0.341703402	3.20E-13	-0.172330053	0.000330674	0.097401915
-0.204349782	1.95E-05	-0.186169089	0.000103071	0.041420253
-0.396853003	1.13E-17	-0.254060697	9.25E-08	0.2174279
-0.366207584	4.30E-15	-0.310234819	4.79E-11	0.095617256
0.330811076	1.94E-12	0.017558801	0.716548737	-0.039521444
0.092950483	0.054101491	-0.001464889	0.975837216	0.033327313
0.525089767	7.64E-32	0.442957301	4.32E-22	-0.030791767
0.339389455	4.72E-13	0.22562691	2.29E-06	-0.036254859
-0.235427596	7.92E-07	-0.081914688	0.089786173	0.052921365
-0.07936088	0.100286784	-0.019057229	0.693533361	0.002672981
0.260064012	4.46E-08	0.119739091	0.012966782	0.045956009
0.134346858	0.005263858	0.127936444	0.007903905	-0.035456653
0.365786217	4.64E-15	0.251342257	1.28E-07	-0.03465614
0.314328991	2.58E-11	0.085481252	0.076617368	0.092958127
0.207501562	1.44E-05	0.105719625	0.028378527	0.06194922
-0.222469663	3.19E-06	-0.026612158	0.582091278	0.07542239
0.469393576	6.02E-25	0.422787833	4.50E-20	0.004464054
0.244256845	2.93E-07	0.144438949	0.002680456	0.113877025
-0.407876483	1.15E-18	-0.220892076	3.76E-06	0.041436437
-0.148643621	0.001997838	0.008584507	0.859117842	0.073666699
-0.40635077	1.58E-18	-0.287288063	1.29E-09	0.135718297
0.435301074	2.62E-21	0.406927908	1.40E-18	-0.005806744

0.577645944	1.23E-39	0.341146183	3.52E-13	-0.037147748
0.318504226	1.36E-11	0.037513306	0.437807287	0.05569923
0.253800332	9.54E-08	0.160414551	0.000842659	0.049275168
0.229584371	1.50E-06	0.117292997	0.014950954	0.065620798
0.541870987	3.49E-34	0.364692856	5.67E-15	-0.014220912
0.628897029	1.01E-48	0.412708894	4.09E-19	-0.051714751
0.531382675	1.05E-32	0.279837091	3.54E-09	-0.060856969
0.645642057	4.48E-52	0.430676036	7.59E-21	-0.073583849
-0.401141531	4.70E-18	-0.230463236	1.36E-06	0.147978145
0.442626068	4.68E-22	0.213885409	7.67E-06	-0.030545602
0.347859435	1.12E-13	0.291415138	7.30E-10	-0.075084826
0.538935482	9.17E-34	0.374613663	8.97E-16	-0.028492991
0.537415452	1.50E-33	0.427915561	1.42E-20	-0.032411135
0.541128162	4.46E-34	0.401435517	4.42E-18	-0.048343839
0.321485413	8.54E-12	0.294103836	5.00E-10	-0.020321588
0.652129205	1.97E-53	0.574245508	4.35E-39	-0.045576538
-0.039262821	0.416725978	-0.186156765	0.000103182	0.023031271
-0.194212974	5.03E-05	-0.05161629	0.285550032	0.171136923
-0.494835136	6.11E-28	-0.371612576	1.58E-15	-0.003345299
0.154606344	0.001299859	0.175619578	0.000252604	-0.076170108
-0.119777378	0.012937665	0.017898661	0.711305153	0.12027435
-0.191497637	6.43E-05	-0.114327577	0.01770995	0.045680939
0.115837012	0.01625451	-0.129280901	0.00726828	0.011574581
0.422405172	4.90E-20	0.185505926	0.000109207	-0.032911158
-0.34790398	1.11E-13	-0.082755277	0.086529507	0.07610894
0.391418438	3.40E-17	0.124386095	0.009827206	-0.081516205
-0.349306972	8.75E-14	-0.14556712	0.002479022	0.077419494
0.539810024	6.88E-34	0.272984527	8.72E-09	-0.059654334
0.531558116	9.93E-33	0.487849021	4.30E-27	-0.048787432
0.237184235	6.52E-07	0.225995297	2.20E-06	0.01682475
-0.428978218	1.12E-20	-0.189036147	8.01E-05	0.016863376
-0.346345449	1.46E-13	-0.264247608	2.65E-08	0.174782822
0.295039719	4.38E-10	0.177482763	0.000216403	0.022003626
0.060321883	0.211900298	0.113974	0.018066895	0.012517761
-0.306411936	8.46E-11	-0.156824134	0.001103489	0.118067684
-0.195048268	4.66E-05	-0.075696958	0.117029206	0.037998501
0.403711643	2.75E-18	0.203176695	2.18E-05	-0.008763245
-0.209031046	1.24E-05	-0.061909849	0.200094508	-0.010671144
0.000302146	0.995015494	-0.159378649	0.000911365	0.007620696
-0.248518636	1.78E-07	0.047309383	0.327718002	0.020720628

MEmagenta	MEpink	MEpink	MEred	MEred	MEturquoise
0.156279168	0.087650775	0.069405588	-0.132184285	0.006049098	-0.281254872
0.84847596	0.122118807	0.011263026	0.193286526	5.47E-05	0.201918158
0.277769321	0.008871198	0.854464388	-0.217528276	5.31E-06	-0.296273758
0.650327906	-0.053307991	0.270033809	-0.033322038	0.49072225	-0.078944935
0.762201259	0.036876852	0.445625503	0.080569143	0.095203111	0.024063147
0.699333124	-0.017635393	0.715365852	-0.035010123	0.469007955	0.124830897
0.623119271	0.06474449	0.180223041	0.298878441	2.54E-10	0.315871159
0.046235194	0.060103372	0.2135633	-0.145808232	0.002437804	-0.280660296
0.614827261	-0.086812298	0.072124031	0.063759434	0.186955842	-0.031392222
0.243011447	-0.02461243	0.610776277	0.374197312	9.70E-16	0.299126952
0.2643651	-0.013428228	0.781276722	-0.039471688	0.414249668	-0.127947483
0.033618794	-0.025987386	0.59098579	-0.143649907	0.002830078	0.080210608
0.011014315	0.060126343	0.213388034	0.14514674	0.002552409	-0.127511969
0.090634601	0.083648766	0.083172814	-0.037536752	0.437520787	-0.107290737
0.713965569	0.146449969	0.002331125	-0.015623764	0.746651379	0.065075091
0.13198555	0.080979548	0.093523923	-0.102008398	0.03445955	-0.119750928
0.449315598	-0.042288628	0.381707536	-0.286536692	1.43E-09	-0.291463083
0.332469892	0.077168438	0.110059819	0.201564438	2.54E-05	0.204616412
0.000289716	0.009630314	0.842167502	-0.209556831	1.18E-05	-0.262387791
0.620584496	0.307042548	7.70E-11	0.302938828	1.41E-10	0.299025953
0.167573248	-0.067198147	0.164235458	0.090764483	0.060034945	0.04591279
0.838013736	0.251433212	1.27E-07	0.19685137	3.95E-05	0.016679745
0.594582224	0.129851055	0.007012818	0.135824526	0.004781386	0.027935611
0.649147868	0.092446548	0.055424183	0.212177226	9.10E-06	0.074544616
0.36679925	-0.077938171	0.106546606	-0.064807453	0.179798877	-0.099058453
0.347526785	0.042642727	0.377730453	-0.134035977	0.005370779	-0.230798088
0.491333068	0.042689476	0.377207294	0.007194328	0.88174899	-0.040855946
0.228643313	0.152124798	0.00155736	0.150612799	0.001736398	0.188427715
0.060474603	-0.007144433	0.882563152	-0.178919622	0.000191865	-0.301414085
0.46441536	0.050889529	0.292396772	0.220670977	3.84E-06	0.179511822
0.420963376	-0.089064605	0.065012036	-0.260284782	4.34E-08	-0.198579649
0.549561964	0.015558518	0.747673433	0.232034382	1.15E-06	0.350732627
0.889710226	-0.036107725	0.455178396	-0.056481985	0.242500643	-0.155167717
0.733146982	0.022569743	0.640705034	-0.043829262	0.364590281	-0.028749052
0.544113125	0.001203658	0.980145143	-0.03907627	0.418945048	-0.042086409
0.003695151	0.196000235	4.27E-05	0.183053883	0.000135009	-0.043444844
0.61365364	0.065626343	0.174349415	0.075119778	0.11985539	0.020327652
0.295847254	0.09424445	0.050824988	-0.118047985	0.014311855	-0.267326363
0.957656837	0.069237631	0.151776893	0.033562466	0.487597038	0.083458869
0.090160072	0.138064887	0.004125674	0.183838579	0.000126186	0.195700762
0.062168064	-0.24869667	1.75E-07	-0.035060227	0.468371647	0.07469681
0.989153614	0.331515541	1.73E-12	0.253673618	9.69E-08	0.314207186
0.204609742	0.116330741	0.01580173	0.143939467	0.002774309	0.287396212
0.839591327	0.029550014	0.541125492	0.021826416	0.651745594	0.113260986
0.657801331	0.056603435	0.241487694	-0.374713331	8.80E-16	-0.34204503
0.943751128	-0.032012339	0.507933158	0.091577632	0.05776788	0.096562566
0.817321588	0.018827698	0.697041281	0.022040617	0.648556151	-0.027948566
0.210195263	0.045113666	0.350691417	0.370458444	1.96E-15	0.287153022
0.006799667	0.049281658	0.307931908	0.200171114	2.90E-05	0.079889908
0.069177419	0.197395043	3.76E-05	0.153214257	0.001439033	-0.005353866

0.181843522	0.101774715	0.034877036	0.061779784	0.201043106	-0.076658125
0.649207831	0.049717486	0.303668175	-0.000705576	0.988360443	0.01703346
0.010593911	0.033225524	0.491979802	-0.028972333	0.549066143	-0.08551486
0.163092755	0.059496323	0.218232492	-0.023315589	0.629706116	0.06177589
0.036218699	-0.030394563	0.529620349	0.003260997	0.946243518	0.131664318
0.656515979	0.051070669	0.290680078	-0.105345984	0.02894587	-0.058074487
0.044013405	0.038747552	0.422872043	0.020014849	0.678969261	-0.095569462
0.336166613	-0.148171485	0.002065652	-0.074699694	0.121945636	0.253421655
0.983387484	0.140706601	0.003457647	0.40371505	2.75E-18	0.178479593
0.078230959	0.028609408	0.554083953	0.136545652	0.004560732	0.056972286
0.890989032	0.076511443	0.113130071	-0.181788101	0.00015047	-0.00509045
0.08112344	0.068608592	0.155540534	0.349985233	7.78E-14	0.105944552
0.105485883	0.007513316	0.876546891	0.13021373	0.00685453	0.148607525
0.903617707	0.053770438	0.265894479	0.001793073	0.970426206	0.101842159
0.053427377	0.012473533	0.796473431	0.076849083	0.111543925	0.079303639
0.435854786	-0.021579303	0.655433017	-0.006785635	0.888421428	0.060771887
0.247368353	0.021027069	0.663703659	-0.038001633	0.43186236	-0.065785329
0.011758989	0.039964599	0.408440264	0.13903438	0.00386803	0.211889668
0.365429779	0.040187872	0.405824774	0.019100353	0.692875012	0.056512348
0.424782773	0.074380819	0.123551161	-0.053975289	0.264074858	-0.078625289
0.02968665	-0.012661311	0.793478117	-0.058419432	0.226694523	0.05138666
0.68996818	-0.085744566	0.075710871	0.19088301	6.79E-05	0.423336696
0.874693671	-0.010188168	0.833155046	0.052160137	0.280497714	0.083589639
0.403247159	-0.016238173	0.737048913	0.003071205	0.94936785	0.132814825
0.324577251	-0.015077389	0.755223668	0.014704422	0.761092664	0.04500599
0.689172649	0.197442677	3.74E-05	0.005415996	0.910837374	-0.026291447
0.82631906	0.014910519	0.757847806	0.051486489	0.286764884	0.090538923
0.912716475	-0.033650289	0.486458146	0.134714783	0.005139796	0.203293051
0.97336218	0.069056811	0.152851621	0.061805185	0.200857588	0.200844825
0.976828003	0.041186107	0.394253616	0.052871055	0.273985051	0.035484
0.016361167	-0.02684552	0.578785093	0.056883252	0.239165198	0.163986458
0.76131526	0.289293453	9.80E-10	0.253871702	9.46E-08	0.179156483
0.910485184	0.009958331	0.836865628	-0.034110759	0.480510255	-0.039352715
0.648629936	0.098753498	0.040673717	-0.027957357	0.563155033	-0.025086731
0.400691724	-0.031464212	0.515228729	0.010199417	0.832973543	0.034899469
0.14828176	0.10797353	0.025154696	-0.106442541	0.027307905	-0.145608272
0.735494296	0.216917289	5.65E-06	0.144470814	0.002674568	0.070985148
0.101523099	0.026562389	0.582797518	0.02972892	0.538677937	0.00178599
0.202041413	0.101589363	0.035211232	-0.019357149	0.688959518	0.007818812
0.711263071	0.010919727	0.821369521	0.129281397	0.007268054	0.163511227
0.081870914	0.04868993	0.313783691	-0.211216978	1.00E-05	-0.223461771
0.307534244	-0.019983989	0.679436776	0.102785931	0.033100942	0.272625457
0.025301423	-0.004811012	0.920762378	-0.136463082	0.004585523	-0.125490159
0.207592599	0.049480904	0.305977792	0.08760199	0.069561425	0.064693797
0.865644338	-0.169076271	0.00042957	-0.105338537	0.028957276	-0.117474116
0.386929993	-0.029050454	0.54798897	0.029491322	0.54192964	0.019083121
0.058729647	0.093937314	0.051587316	-0.100599785	0.037041907	0.060247858
0.578297712	0.055590611	0.250026259	0.276207275	5.73E-09	0.269117321
0.819944547	0.014300916	0.767457663	0.085381076	0.076964543	-0.004782505
0.988630312	-0.149814678	0.001838332	0.006887183	0.886762778	0.211345336
0.854469398	-0.062345378	0.196941815	-0.286032704	1.53E-09	-0.273733002

0.854225005	0.066828944	0.166570624	0.060952338	0.207154487	0.207265294
0.994130209	-0.006161616	0.898624388	0.03970964	0.411439131	0.152440956
0.952196545	-0.028672138	0.553215057	0.034580206	0.474487297	0.034652245
0.197574233	0.124813262	0.009575666	0.006563859	0.892045535	-0.149801732
0.676347402	0.206857449	1.53E-05	0.080644784	0.094891827	-0.065995391
0.334145812	0.044656065	0.355604367	-0.288406712	1.11E-09	-0.336622295
0.460154644	0.037347029	0.439842219	0.043235707	0.371127581	0.118603383
0.640780645	-0.066036716	0.171665174	-0.136299674	0.004634943	-0.101059006
0.733436737	0.051134868	0.290073275	0.100086327	0.038023189	0.086409811
0.220423227	0.118329668	0.014079635	0.160214194	0.000855562	0.31019387
0.606643996	-0.120426731	0.012452539	0.030591379	0.526957076	0.137288613
0.635385164	0.001150691	0.981018688	0.131070504	0.006493227	0.055012701
0.830640199	-0.002555931	0.957854039	0.201601568	2.54E-05	0.148041731
0.508443378	0.020926348	0.665216566	-0.035654731	0.460858113	-0.127174129
0.010013464	-0.060958728	0.207106784	-0.028292175	0.558488275	-0.004790749
0.618553223	0.007868298	0.870764029	-0.013530019	0.779661214	-0.032084148
0.764849787	-0.009776131	0.839809698	-0.148620143	0.002001162	-0.284115989
0.376264657	-0.032018804	0.507847437	0.002442072	0.959729881	0.027308302
0.173441967	0.059751825	0.216258429	0.080964794	0.093583877	0.212639321
0.855795764	0.045034655	0.351536632	-0.000374757	0.993817655	-0.255902238
0.508918103	0.084873818	0.078742203	-0.021121045	0.662293279	0.023112181
0.476338677	0.193929699	5.16E-05	0.214633061	7.12E-06	0.285057095
0.549842018	0.061719949	0.201480591	0.056737923	0.240369473	0.126168443
0.000129616	0.074300822	0.123956515	-0.004938479	0.918670156	-0.044054664
0.210652416	0.083162689	0.084985643	0.047217356	0.328660877	0.022087758
0.818370406	0.160066876	0.000865166	0.151926648	0.001579817	0.298010741
0.860767337	-0.112000133	0.020177162	-0.051533604	0.286323521	0.118540742
0.126963657	0.031447512	0.515451859	-0.0608989	0.207553741	-0.051570344
0.098598827	0.141183188	0.003348146	0.107390324	0.025957011	-0.174226057
0.26640316	0.15389091	0.001369759	0.157449767	0.00105326	0.243142818
0.14759553	-0.05566797	0.249366768	-0.143978893	0.002766794	-0.158270598
0.105077357	-0.017015742	0.724955025	0.033034538	0.494473364	0.072786833
0.010931763	0.00401779	0.933793906	0.019523398	0.686429059	-0.207384476
0.52966237	-0.067883861	0.159963743	0.105830744	0.028211654	0.037980673
0.746238647	0.055626082	0.249723723	0.142063426	0.003154141	0.206504583
0.777057209	0.101193801	0.035933558	-0.029210589	0.545784202	-0.10573185
0.849445455	-0.187597798	9.09E-05	-0.160586339	0.000831739	-0.27627935
0.808895652	-0.119830345	0.012897479	-0.010295489	0.831423677	-0.006798151
0.961869609	-0.033765441	0.484967023	0.268311935	1.59E-08	0.317497682
0.393668881	0.095892627	0.046893008	0.239274282	5.16E-07	0.141644986
0.350217022	-0.072245288	0.134730705	-0.082642258	0.08696176	0.02615055
0.019921973	-0.031891273	0.509539893	0.062666445	0.194641002	0.222883063
0.478099426	0.021539919	0.656021475	0.01983471	0.681700025	0.154596928
0.069317029	-0.018051337	0.708953972	-0.283666208	2.12E-09	-0.333171034
0.937700599	0.005452667	0.910236193	0.122359034	0.011102567	0.021250731
0.752428659	0.072280565	0.134539903	0.053180778	0.271180168	0.11272241
0.730033561	-0.103490114	0.031910188	-0.228111348	1.76E-06	-0.111561442
0.057157426	-0.082303407	0.088268152	0.123209212	0.010550828	0.037184883
0.533036467	0.094798981	0.049472428	0.22909656	1.58E-06	0.326205786
0.490930255	0.067869601	0.160051716	-0.107177293	0.026255525	-0.110412665
0.931630719	0.041363468	0.392218733	0.038301837	0.428230903	0.118158887

0.610432412	0.170613591	0.000379838	0.039738445	0.411099669	-0.012405176
0.810951341	0.170080866	0.00039643	0.157527504	0.001047169	0.063561886
0.001110071	0.046270482	0.338463821	-0.068830344	0.154205785	-0.249835331
0.158187531	-0.043848496	0.364379641	-0.04199839	0.384986379	-0.083193945
0.222491629	0.035404479	0.464012675	0.111802855	0.02039941	0.055867442
0.830791854	-0.142101254	0.003146037	-0.004568106	0.924750882	0.19127071
0.545099312	-0.097586044	0.043121999	0.069543551	0.149971696	0.187598002
0.75055476	0.06201978	0.199295303	0.12447904	0.009771981	0.113172329
0.67120899	0.033062473	0.494108223	-0.027233343	0.573310109	0.070904938
0.910585149	0.086995906	0.07152144	-0.026996297	0.576653639	-0.029015136
0.655022535	-0.128861749	0.007461358	-0.068152581	0.158312755	-0.115680246
0.562433653	0.097305509	0.043728463	0.119584689	0.013084791	0.128528421
0.863642224	-0.04035954	0.403820596	0.04093585	0.397135654	0.213219503
0.740153737	0.007192618	0.881776897	-0.086686369	0.072539714	-0.086858863
0.021792749	0.150312814	0.001774088	0.181244385	0.000157608	0.04358611
0.008017647	0.039377062	0.41537047	-0.113404514	0.01865501	-0.325069302
0.574170091	-0.151301152	0.001652671	-0.145117867	0.002557522	-0.069890642
0.959047168	-0.088152704	0.067818792	0.023043689	0.633706495	-0.10068573
0.200302921	0.093042711	0.053862281	-0.034431762	0.476387339	-0.049461096
0.565615082	0.059827243	0.215678192	0.001123763	0.981462808	-0.034589724
0.727886802	0.022051601	0.648392769	-0.098183552	0.041853883	-0.197772077
0.346691482	-0.123102595	0.01061866	-0.082663542	0.086880226	-0.016093232
0.877968228	0.182845731	0.000137444	0.15156411	0.001621676	-0.116613258
0.073680957	0.024615946	0.610725291	0.041803512	0.38719754	0.16420117
0.168367957	0.135489023	0.004887289	0.231962733	1.16E-06	0.240790445
0.331849214	0.070046127	0.147041534	0.115578449	0.01649613	0.21262652
0.833583643	0.025955419	0.591442545	0.004576675	0.924610156	0.138544193
0.831441072	0.099272809	0.039622994	-0.131205115	0.00643804	-0.236656851
0.048251161	0.071949022	0.136341339	-0.104926075	0.029595053	-0.163457299
0.691310605	0.028826499	0.551079761	0.181527461	0.000153853	0.109935796
0.359004942	-0.001482657	0.975544234	-0.047632045	0.324425965	-0.07612419
0.742699135	-0.018170526	0.707120395	-0.058343284	0.22730159	-0.090834903
0.947564386	-0.01928922	0.689994461	0.329223391	2.50E-12	0.346598342
0.37216824	0.046519653	0.335866201	-0.297694665	3.00E-10	-0.298840758
0.76228897	0.027311162	0.572214476	0.057936797	0.230561724	0.119717936
0.204330184	0.076791884	0.111811397	0.029298615	0.544574123	0.274930435
0.114604966	0.025408882	0.599276712	-0.098478136	0.041240341	-0.166651186
0.03323657	-0.048281609	0.317863946	-0.030600123	0.526838916	0.113462161
0.676147091	0.126578651	0.008595735	-0.009544679	0.843552837	-0.017466709
0.681254706	0.136245575	0.00465141	-0.006410275	0.894556611	-0.188946256
0.415265648	-0.005174058	0.914804877	-0.049130571	0.309419168	0.039027628
0.52384241	0.119533121	0.013124414	0.183289773	0.000132297	0.081628628
0.591193726	-0.098329871	0.041548182	-0.129742899	0.007060652	-0.001304381
7.76E-05	0.043604888	0.367053018	-0.104582865	0.030134888	-0.228581196
0.204465901	0.023602774	0.625492569	-0.182477399	0.000141855	-0.207622797
0.390152772	0.087026426	0.071421672	0.235998871	7.44E-07	0.352548155
0.213711991	0.062799159	0.193695714	0.063173364	0.191048448	0.105706547
0.526626441	0.131477431	0.006327682	-0.008449671	0.861308144	-0.092847994
0.001260874	0.065213454	0.1770815	0.010451291	0.828911642	0.192770439
0.232146768	0.089742734	0.062987309	0.17551667	0.000254759	-0.025814636
0.267062282	-0.02565583	0.595731112	0.176390428	0.000236989	0.28218072

0.959556202	0.004133474	0.931892187	-0.01080376	0.823235148	-0.017142767
0.744096058	0.036796706	0.4466156	-0.048664078	0.314041004	-0.001093306
0.415743651	0.010772184	0.823743308	0.002229666	0.963229903	0.031009953
0.666124417	0.059521615	0.218036504	0.088863264	0.065623389	0.101344127
0.157846227	0.128018063	0.007863948	0.236021336	7.42E-07	0.37235507
0.257986442	-0.051905048	0.2828599	-0.075144133	0.119735067	-0.002738173
0.014082443	-0.089668102	0.063207565	-0.029291208	0.544675895	0.031249637
0.103707509	-0.001815879	0.970050224	-0.251274354	1.29E-07	-0.303717787
0.06935682	0.010878251	0.822036646	-0.140654729	0.003469759	-0.148149715
0.732321303	0.148756884	0.001981876	0.068068522	0.158827816	-0.085626148
0.414569657	0.171157915	0.000363555	-0.121011764	0.012029287	-0.051125366
0.413485472	0.036082716	0.455490931	-0.081580548	0.091107726	-0.154588475
0.972084036	-0.073770923	0.126667719	0.046543399	0.335619321	0.146499006
0.469032533	0.07749244	0.108570053	0.006241604	0.89731559	-0.079926092
0.789313061	0.089502681	0.063698024	0.066012354	0.17182366	-0.043104516
0.078767146	0.005017003	0.91738155	-0.06814854	0.158337484	-0.178555133
0.002930094	-0.010005367	0.836105952	-0.19784012	3.60E-05	-0.036227451
0.165749215	-0.011391727	0.813786611	-0.114463483	0.017574397	0.207752299
0.041331403	-0.175784815	0.000249178	-0.179587534	0.000181369	-0.064674045
0.097388875	-0.025115362	0.603503332	-0.166150353	0.000541354	-0.337378349
0.6318513	0.137680047	0.004232178	0.237724981	6.14E-07	0.206237044
0.042461786	-0.010471552	0.828585092	-0.119967439	0.012793976	-0.034013107
0.938364027	0.057661522	0.232788227	-0.042172513	0.383017249	-0.075949887
0.697705616	0.152787865	0.001484324	0.097166643	0.04403131	0.064340196
0.423258247	0.019650273	0.684500247	-0.035194333	0.466670898	-0.013956746
0.92681539	0.065206877	0.177125275	-0.091263184	0.058636009	-0.396075427
0.793975526	-0.106759556	0.026849478	-0.230095411	1.42E-06	-0.141171028
0.114278274	0.08692859	0.071741888	0.037664014	0.435967566	-0.010896626
0.983332841	-0.056788894	0.239946613	0.2600416	4.47E-08	0.416432274
0.065773964	0.067495919	0.162370059	0.218580862	4.77E-06	0.295490658
0.607377351	0.022629282	0.639824086	0.065893589	0.172597846	-0.022331212
0.652241503	0.0917589	0.057272291	0.204077313	2.00E-05	0.083337008
0.636199031	0.153211484	0.001439324	0.169075314	0.000429603	0.127133971
0.171896239	-0.028118256	0.560910046	-0.03200737	0.507999051	0.005815433
0.865302188	-0.060395468	0.211342385	-0.09595696	0.046744854	0.118904154
0.690809875	0.047008998	0.330802101	0.136234474	0.004654795	0.234462975
0.828755351	0.179389007	0.000184431	0.148287031	0.002048864	0.012832368
0.004220071	-0.06661608	0.167928219	-0.093840113	0.05183055	0.0070627
2.57E-05	0.050981486	0.29152443	0.059826519	0.215683761	-0.181092086
0.044421961	0.167384701	0.000491254	0.285002501	1.77E-09	0.338836334
0.0439068	0.010972762	0.820516633	0.045490633	0.346676564	0.285666628
0.918178438	0.083702908	0.082972836	0.076579636	0.112808299	0.278983984
0.052753542	0.087026004	0.07142305	0.089166799	0.064703529	0.123846773
0.723222943	-0.042655874	0.377583282	-0.250090678	1.48E-07	-0.211961296
0.322380339	0.001463396	0.975861848	-0.071066668	0.141225996	-0.183160838
0.002229008	-0.021485348	0.656837224	0.072517745	0.133262479	-0.079636804
0.101310217	0.020318647	0.674373426	0.324009593	5.74E-12	0.26334791
0.020900865	0.006253398	0.89712262	-0.098114557	0.041998687	-0.201941187
0.994798139	0.060470117	0.210777478	0.089557025	0.063536555	0.019468402
0.169564005	0.218626337	4.74E-06	-0.020560152	0.670728576	0.144346212
0.995851756	-0.141749031	0.00322223	-0.019281013	0.690119547	-0.014874677

0.518715586	0.000903623	0.98509369	-0.054981079	0.255265039	-0.100389973
0.343678842	0.05137463	0.287814598	-0.213640259	7.86E-06	-0.032817153
0.269934698	0.041280439	0.393170544	-0.070798336	0.14273773	-0.122571803
0.001384159	-0.015288318	0.751910689	-0.054764576	0.257144002	-0.016719957
0.404942592	-0.015949469	0.741556019	0.010069641	0.835068133	-0.01133589
0.095152804	0.08407121	0.081622773	-0.059327518	0.219543771	-0.065030218
0.220700858	-0.009659086	0.84170215	-0.101692035	0.035025777	-0.231478255
0.364770866	-0.003246738	0.946478216	0.023219291	0.631121687	0.157594586
0.562306985	-0.068890817	0.153843299	-0.038557201	0.425155842	0.039829029
0.603695273	0.100484854	0.037259667	-0.018194539	0.7067512	0.079629802
0.413147002	0.049822179	0.302649814	0.253064353	1.04E-07	0.273066587
0.22800221	0.097630489	0.043026571	-0.019235747	0.690809566	0.047233739
0.639221568	0.013037837	0.787481216	-0.293564056	5.40E-10	-0.296661652
0.144704641	0.020358963	0.673764439	-0.09449341	0.050213978	0.098683575
0.324178298	-0.026440479	0.584529151	-0.105458222	0.028774433	-0.190292359
0.559788951	0.079623894	0.099162199	0.126266527	0.008762201	-0.111894719
0.241291484	-0.030325518	0.530556269	-0.134753341	0.005126948	-0.233833933
0.818836972	0.025834218	0.593175811	-0.056859439	0.239362233	-0.113043429
0.978485792	-0.002179055	0.964063971	-0.044891548	0.353070772	0.249226163
0.770194999	-0.084784136	0.079059926	-0.173309184	0.000305355	0.035367882
0.791309626	0.302310128	1.54E-10	0.007674572	0.8739191	-0.04091223
0.404643107	-0.140216615	0.003573603	-0.231131482	1.27E-06	-0.307724976
0.893783266	0.070585389	0.143946223	0.006572855	0.891898481	0.088193418
0.451791194	-0.008046371	0.867865698	0.237882691	6.03E-07	0.124984308
0.497457443	0.082584959	0.08718157	0.324290893	5.49E-12	0.355370689
0.3220087	-0.040386556	0.403505731	-0.040597649	0.40105054	-0.002062292
0.013313183	0.093635039	0.052346863	-0.053922611	0.264541965	-0.028736797
0.00701945	0.009527543	0.843830102	-0.096836314	0.04475881	-0.185726281
0.812824453	-0.009190655	0.849285047	-0.099749113	0.038679561	0.030725699
0.013128711	-0.020558976	0.670746301	-0.198882956	3.27E-05	-0.213516347
0.342534669	0.023633844	0.625037448	0.027483621	0.569789911	0.124001265
0.711116832	0.053834582	0.265323787	0.020213739	0.675959108	0.030948408
0.03217797	0.039583887	0.412923038	-0.061071296	0.206267708	-0.236686306
0.262559334	-0.019590105	0.685414691	0.10564631	0.028489092	0.150892091
0.251463142	0.040663616	0.400285121	0.040955807	0.396905367	0.057882329
0.318830103	-0.056148444	0.245297831	0.027966107	0.563032841	0.031567478
0.000296711	0.017751537	0.713573426	0.022857381	0.636453767	0.010726207
0.49752282	0.102835222	0.033016375	0.018866729	0.696444318	0.115149396
0.129927943	0.062959565	0.192557674	0.30610984	8.84E-11	0.358997715
0.579881396	-0.001122976	0.981475791	0.038626373	0.424325098	0.271463969
0.221280277	0.045442651	0.34718596	0.092603672	0.055008922	0.314966344
0.328619043	-0.063542017	0.188466498	0.0315091	0.514629248	0.050293123
0.058104857	-0.091027029	0.059295062	-0.139498619	0.003749891	-0.269675497
0.08090892	-0.033577007	0.487408375	0.204503923	1.92E-05	0.386475438
0.383343495	-0.000216974	0.996420557	-0.077570067	0.108215496	-0.180837869
0.445782051	0.424336293	3.18E-20	0.140678274	0.003464256	0.07287357
0.034953432	0.131599724	0.006278676	0.101351404	0.035644267	0.012487346
0.560319738	0.047907734	0.321630244	0.155117799	0.001251916	0.188984898
0.360020974	0.02150405	0.65655761	0.073963908	0.125675042	0.292605068
0.916835613	0.103744954	0.031488374	-0.034005179	0.48187054	-0.031933071
0.620221487	-0.03782935	0.433954397	-0.187991304	8.78E-05	-0.06411357

0.806800406	-0.004819676	0.920620147	0.057258163	0.236078094	0.155670589
0.613359795	0.057348062	0.235342041	-0.054275201	0.261426314	-0.114627261
0.08149241	0.099074994	0.040020495	0.075895145	0.116070888	0.213487357
0.3808362	0.114301015	0.01773655	-0.054671568	0.257954117	0.125491592
0.05767789	0.004344039	0.928431752	-0.004342926	0.928450032	-0.170636784
0.797798827	0.041025591	0.396100714	-0.055042813	0.254731014	0.21378846
0.537614547	-0.095363198	0.048127258	-0.202477059	2.33E-05	-0.25052367
0.551646122	0.05124614	0.289023567	0.308853996	5.89E-11	0.212136019
0.962517452	0.040374555	0.403645591	0.069539602	0.14999489	0.014696563
0.554455599	0.06895169	0.153479067	-0.013627348	0.778117395	0.028623226
0.220278794	-0.083618789	0.0832837	0.289096076	1.01E-09	0.435057301
0.355011908	0.049427487	0.306500883	0.154268748	0.001332424	0.189088436
0.31488539	-0.082490533	0.087544779	-0.167952303	0.000469693	-0.236561791
0.786262926	0.042432282	0.380090997	-0.333580825	1.23E-12	-0.144452111
0.235088014	-0.102516433	0.033566578	0.224576711	2.56E-06	0.244904147
0.031680169	0.009153019	0.849894912	-0.19769744	3.65E-05	-0.040690674
0.296652968	0.109244429	0.023480115	0.096154326	0.046292781	0.399343135
0.263376248	0.05511197	0.254133703	-0.134254198	0.005295524	-0.129556876
0.472217927	0.101778761	0.034869773	0.140904705	0.003411742	0.115880362
0.095785261	-0.059087862	0.221415086	0.017854986	0.711978234	0.041079721
0.013890335	-0.034602958	0.474196445	-0.204006283	2.02E-05	-0.388073515
0.035469096	0.180246894	0.000171537	0.279627908	3.64E-09	0.290694412
0.508629697	-0.13178853	0.006203685	-0.18087891	0.000162584	-0.106751924
0.293046492	-0.033951482	0.482563163	-0.069204099	0.151975762	0.097005288
0.183067934	0.037778593	0.43457186	0.186655037	9.88E-05	0.226258159
0.362191322	0.101380381	0.035591295	0.277907779	4.58E-09	0.377631727
0.248323479	-0.072011606	0.135999879	-0.049718545	0.303657862	0.079451077
0.328318963	0.130144099	0.00688467	0.044994408	0.351967658	0.143845246
0.754070153	0.030578967	0.52712483	-0.219383554	4.39E-06	-0.251223069
0.001729801	0.005335124	0.91216334	-0.059748908	0.216280893	-0.232643789
0.423863111	0.028210441	0.559625771	0.051918442	0.282735538	0.092744921
0.073645964	0.087242098	0.070719909	-0.145665113	0.002462194	-0.157910447
0.036192794	0.006655382	0.890549645	-0.040105493	0.40678862	-0.02696871
0.351787539	0.029677924	0.539375022	0.116437895	0.015704936	0.180814578
0.699436031	0.083703858	0.08296933	0.00704579	0.884173118	0.195418016
0.574464303	0.001099646	0.981860554	0.16055594	0.000833662	0.001338926
0.245118999	0.183945654	0.000125025	0.175302585	0.000259299	-0.041274935
0.988017392	0.033755611	0.485094218	0.488007544	4.11E-27	0.314200771
0.200628193	0.030493919	0.528275026	0.49678564	3.52E-28	0.221237732
0.038781882	0.028335217	0.557889708	0.217544456	5.30E-06	0.174851645
0.492357385	-0.004059303	0.933111443	0.017764478	0.713373806	-0.061215391
0.26593419	0.002434531	0.959854126	-0.080401062	0.095897731	-0.112523629
0.602255464	-0.014990004	0.756597509	0.112997151	0.019085873	0.182253836
0.75193492	0.049559307	0.305211109	-0.299041029	2.48E-10	-0.459885142
0.113373699	-0.084605054	0.079697479	-0.011747726	0.808078754	0.022218786
0.207606126	0.032608203	0.500063918	0.013939979	0.773164469	0.136857231
0.000712481	0.030368696	0.529970876	-0.181624318	0.000152587	-0.29364296
0.129512059	0.025193563	0.602375945	-0.197662934	3.66E-05	-0.247482015
0.855778279	-0.026919043	0.577745283	0.024001403	0.619664122	0.093978279
0.986017185	0.017684324	0.714610514	0.056015615	0.246418037	0.079963295
0.386402471	-0.103981403	0.031101271	-0.014408316	0.765761955	0.175580633

0.41041551	0.025549974	0.597249795	0.145993106	0.002406624	0.122467866
0.050349558	-0.021579381	0.655431848	-0.047112116	0.329741264	-0.061693404
0.724508518	-0.083411299	0.084054512	0.052849083	0.274184781	-0.034785253
0.343100294	0.041949074	0.385545209	-0.108040382	0.025064109	-0.217970012
0.119203963	0.013853861	0.774527897	-0.277262055	4.98E-09	-0.38313261
0.312401269	-0.005714651	0.905942834	0.184897709	0.000115135	0.270571608
0.902876664	0.057303128	0.235709739	0.146710367	0.002289067	0.094468846
0.240397112	-0.006510458	0.892918516	-0.001518073	0.974960251	0.017966199
0.138057204	0.016404033	0.734463646	-0.198478452	3.40E-05	-0.284684904
0.877334486	0.055087332	0.254346385	0.013313348	0.783101088	0.043657223
0.06804548	0.085294375	0.077266051	-0.100061478	0.038071233	-0.092498065
0.149651267	0.002852974	0.952961309	0.313014252	3.15E-11	0.174528822
0.076700421	-0.027639163	0.567607384	0.080453577	0.095680272	0.016056034
0.051421865	0.087522035	0.069817447	-0.125925376	0.008947425	-0.048623134
0.067454752	-0.139897451	0.003651017	-0.179956377	0.000175806	-0.007876393
0.000204484	0.102234967	0.034058843	0.203374487	2.14E-05	-0.017039146
0.726776275	0.130291687	0.006820926	0.298427702	2.71E-10	0.184268037
0.685909782	0.035554402	0.462121375	-0.022015392	0.648931407	-0.201599728
0.055729563	-0.03877981	0.422485737	-0.116899198	0.015294144	-0.143454358
0.064793834	0.025660535	0.595663657	-0.355735895	2.84E-14	-0.416400541
0.262870579	0.079858631	0.098167009	0.139277569	0.00380573	-0.002098983
0.9212003	0.05062529	0.294913151	0.206137692	1.64E-05	0.240629179
0.197239597	0.057846162	0.231293138	0.038309247	0.428141494	-0.051585732
0.774948822	-0.030426933	0.529181854	-0.120046663	0.012734498	-0.127761648
0.426385951	-0.046569282	0.335350357	0.181711182	0.000151461	0.272693608
0.196506227	0.030550162	0.527514252	-0.03193084	0.509014477	-0.057616695
0.87449949	0.099762587	0.038653153	0.183137928	0.000134037	0.317566249
0.754857885	0.02146327	0.657167354	-0.018298309	0.705156501	-0.061241448
0.496597499	0.11517698	0.016877522	-0.01418389	0.769306602	0.026278861
0.949760713	0.046418694	0.336917157	-0.015557141	0.747695011	-0.062679401
0.86025717	-0.018829299	0.6970168	-0.036250658	0.453394458	0.069409751
0.498545577	0.202278508	2.38E-05	0.072299822	0.13443584	-0.069980053
0.126647652	-0.004674563	0.923002627	-0.191691669	6.32E-05	-0.384890362
0.343542609	-0.139222259	0.003819818	-0.368922792	2.60E-15	-0.330166655
0.040352278	0.056833502	0.239576976	-0.148428021	0.002028549	-0.300310811
0.060814461	0.051345428	0.288089068	0.073857149	0.12622344	-0.133407665
0.182057192	-0.035040144	0.46862664	-0.270036376	1.28E-08	-0.320206586
0.472836016	-0.015185192	0.753529878	-0.090966715	0.059464362	0.001146264
0.000527392	0.032980837	0.495175717	0.181033458	0.000160462	0.030356558
0.542217955	0.016935217	0.726204355	-0.048864585	0.312048941	0.001650501
0.277409941	0.053367108	0.269502214	0.256412231	6.97E-08	0.475544229
0.388943704	-0.024986913	0.605357147	-0.197678066	3.66E-05	-0.251002441
0.469832147	-0.017254922	0.721248493	-0.164286144	0.000626075	-0.300360569
0.021480691	0.03658336	0.449257295	0.076964675	0.111004943	0.179924133
0.041753074	-0.003256784	0.94631286	-0.191334732	6.52E-05	-0.294807213
0.972282211	0.15724884	0.001069157	0.006398036	0.894756766	0.048596209
0.016402584	0.041278797	0.393189385	0.04066367	0.400284495	-0.168590639
0.319627713	-0.046052167	0.340750294	-0.133360936	0.005609701	-0.208767088
0.28382497	-0.104386069	0.030448214	0.015807212	0.743780132	0.038186045
0.156808067	-0.102215171	0.034093695	-0.109918776	0.022631375	0.014721403
0.078821252	0.066940096	0.165865001	0.186712933	9.83E-05	0.005433619

0.699096292	0.234953587	8.35E-07	0.083464352	0.083856879	0.091965455
0.514216769	0.062723312	0.194235538	-0.043676311	0.36626796	-0.029822803
0.016591353	0.007638321	0.874509722	0.002743107	0.954770788	0.126546859
0.845337104	0.033287699	0.491169482	0.012012361	0.803842344	0.04882911
0.588571126	-0.023293087	0.63003677	0.078494531	0.104062877	0.137474031
0.475273272	0.06993955	0.147659237	-0.051022361	0.291137235	0.005390675
0.364202064	0.010883673	0.821949426	-0.016550108	0.732189245	-0.03110811
0.332385799	0.117925394	0.014413967	0.083788588	0.082657166	0.237192778
0.189920953	-0.087272473	0.070621524	-0.138771883	0.003936311	0.17165019
0.759475889	0.005930703	0.902404244	0.103572924	0.031772593	0.206303974
0.094215185	-0.017944025	0.71060627	-0.124109247	0.009993346	-0.155673227
0.611562066	0.016133926	0.738675334	-0.059684186	0.21677977	-0.055506455
0.673014066	-0.066040634	0.1716397	0.078417002	0.104406209	0.098187575
0.231096567	0.121775223	0.011496087	0.085845715	0.075364975	0.037517765
0.041494078	0.080918598	0.093771797	0.088558165	0.066558788	0.349823522
0.020730083	0.024225253	0.616401534	0.077977073	0.10637143	0.311556213
0.9280371	0.159002332	0.000937579	-0.018231047	0.706190007	-0.011666889
0.062492559	-0.020143649	0.677019316	0.054627953	0.258334619	0.203010507
0.808805396	-0.010847023	0.822539042	0.499964733	1.42E-28	0.532814655
0.882331366	-0.003449747	0.943137144	-0.11959985	0.013073162	0.004439474
0.214526399	0.038546738	0.425281588	0.061417832	0.203700098	-0.190387259
0.404589526	-0.063291521	0.190218097	0.075227455	0.119324143	0.095475943
0.963906231	-0.060070035	0.213817836	-0.183555605	0.000129303	-0.161281056
0.856480089	-0.015077217	0.755226368	0.071150975	0.140753564	0.011569011
0.000229439	0.046673872	0.334264891	-0.011374535	0.814062499	-0.229323752
0.195831078	0.006238837	0.897360848	-0.131274816	0.00640963	-0.311895632
0.003581743	-0.138304121	0.004060695	0.012919759	0.789360512	0.220078929
0.783462751	0.011350237	0.814452471	-0.014981055	0.756738239	-0.02777649
2.13E-06	-0.009537554	0.843668123	-0.06023047	0.212594867	-0.257868235
0.24511108	0.002125847	0.96494088	-0.06733501	0.163376089	-0.036182654
0.60644671	0.038000147	0.431880374	0.17822215	0.000203431	0.16192049
0.708011912	0.199080267	3.21E-05	0.226176543	2.16E-06	0.117272811
0.00074154	-0.068886549	0.153868857	-0.172548561	0.000324859	-0.194132202
0.032957882	0.023780326	0.622893624	0.175956172	0.000245671	0.296726071
0.073401592	-0.019256589	0.690491827	-0.026282025	0.58678343	0.032675628
0.727928264	0.078874946	0.102391153	0.322258326	7.57E-12	0.260552987
0.307593337	-0.025453357	0.598637446	0.042887701	0.374993957	0.26392573
0.079109863	0.049752176	0.303330492	-0.273022415	8.68E-09	-0.330907448
0.541504542	0.076702405	0.112230822	0.075022794	0.120335462	-0.111573122
0.345532529	-0.043512562	0.368069386	0.208492912	1.31E-05	0.282712231
0.622676667	0.185454501	0.000109697	0.092656871	0.054868913	-0.064298963
0.968883755	0.054107911	0.262901399	-0.007067775	0.883814265	0.016525667
0.943055678	0.118914455	0.01360809	0.25193757	1.19E-07	0.162450745
0.997273583	0.204982394	1.84E-05	0.189438844	7.73E-05	0.224295808
0.995313235	-0.031364539	0.516561185	0.077649617	0.107853099	0.131381479
0.329438664	0.040121957	0.406595881	-0.035898656	0.457794845	0.046501362
0.925362428	-0.031912952	0.509251982	-0.102869974	0.032956864	0.025959041
0.474264247	0.05116156	0.289821237	0.134468056	0.005222696	0.182056753
0.330656866	-0.008995766	0.852444024	-0.0356078	0.461448795	0.09201414
0.19641557	-0.054634651	0.258276154	-0.081649156	0.09083511	-0.100654053
0.374307844	-0.027967437	0.56301426	-0.302451461	1.51E-10	-0.090144745

0.940636996	-0.103432592	0.032006064	0.05369901	0.266530971	0.146135375
0.184732806	0.026644648	0.581630443	0.100436977	0.0373507	0.00269385
0.016946464	0.003967509	0.934620584	0.149919583	0.001824628	0.161188344
0.155567331	0.070111874	0.146661463	-0.055341174	0.252160995	-0.088272814
0.442286415	0.094887288	0.04925984	-0.13644049	0.004592327	-0.312559994
0.004103938	-0.017899601	0.71129066	0.130685093	0.006653588	0.024094631
0.069283521	0.018811136	0.697294657	-0.000329993	0.994556114	0.012198598
0.556116515	0.00822374	0.864980583	-0.039806927	0.410293297	0.006292717
0.952154057	0.105671585	0.028450933	0.067949393	0.159559932	0.122095756
0.146093375	0.019158722	0.691984314	0.020028459	0.67876311	-0.192925653
0.994339202	0.03634224	0.452253509	0.147404936	0.002180248	0.059459719
0.823337572	0.121413923	0.011745757	-0.061987885	0.199526938	0.086012231
0.028106327	0.067863556	0.160089019	-0.034310981	0.477936371	-0.054340154
0.051995307	0.068413439	0.156722397	0.171033034	0.000367232	0.206512846
0.635001474	0.025187971	0.602456526	0.051424703	0.287344379	0.043887651
0.371367195	0.181288291	0.00015702	0.074881239	0.121038849	-0.147165778
0.717183263	0.093557846	0.052542319	0.240656467	4.41E-07	0.433801103
0.178824824	0.021274067	0.659999325	-0.011416413	0.813390483	0.008171123
0.604964365	0.084332689	0.080675108	0.238389795	5.70E-07	0.085708812
0.308218056	-0.036451439	0.450895188	-0.057373531	0.235133805	-0.088303782
0.11884056	-0.021345064	0.658936068	0.046903609	0.331888562	-0.089051742
0.823599047	-0.007972589	0.869066369	-0.028435016	0.556503029	-0.076130169
0.39543786	0.130869422	0.006576455	0.133099644	0.005704719	0.165654923
0.03750036	0.114776322	0.017265811	-0.17902457	0.000190179	-0.24623356
0.533635515	-0.006014377	0.901034307	0.126709475	0.008526804	0.133227465
0.858237095	0.016424463	0.734145412	8.28E-06	0.999863344	0.074624946
0.07055711	-0.008094361	0.867084909	0.022442671	0.642586908	0.039888008
0.005322497	0.061822646	0.200730137	0.053003326	0.272784789	0.168528655
0.3958336	0.103404072	0.032053692	0.071582158	0.138356254	0.098059184
0.437505868	0.005900146	0.902904596	0.28913164	1.00E-09	0.316469931
0.593364511	-0.030521433	0.527902781	0.049640511	0.304418373	0.107781363
0.508462347	0.059230536	0.22029967	-0.035418219	0.463839164	0.021543563
0.117531122	0.173888217	0.000291246	-0.100006912	0.038176911	-0.069389713
0.428813263	-0.006320682	0.896021934	0.146838595	0.002268611	0.1182881
0.351099831	0.020545875	0.670943834	-0.269905098	1.30E-08	-0.296754232
0.266177849	0.037166941	0.442052247	0.087759447	0.069059481	0.112894836
0.378921459	0.159484414	0.00090412	0.076198085	0.11461794	0.138775213
0.964160136	0.020629495	0.669683458	0.028358309	0.557568698	0.195722885
0.946217043	-0.000123656	0.997960035	-0.034666546	0.473384077	-0.015900298
0.926595141	-0.005300601	0.912729448	-0.00151278	0.975047526	0.033380453
0.495139462	0.186136997	0.00010336	0.269456834	1.37E-08	0.162221159
0.579669697	-0.04500742	0.351828271	-0.037472165	0.438310271	0.220539104
0.134196518	0.105665736	0.028459759	-0.052688967	0.275643258	-0.060121426
0.723318476	0.030903796	0.522743545	-0.127357063	0.008192794	-0.160278676
0.024828042	-0.058522518	0.225874538	-0.04933473	0.307410601	0.013695612
0.078347125	0.086561342	0.072954352	0.029751379	0.53837107	-0.024067427
0.529150466	-0.011204665	0.816789817	-0.075774217	0.116654893	0.048957136
0.73103273	-0.04706969	0.330177459	-0.15675109	0.001109496	-0.309927427
0.159073526	-0.030274103	0.531253738	-0.128560885	0.007602761	-0.044350433
0.67748726	-0.001025597	0.983081863	-0.082458938	0.087666579	-0.020777012
0.305730579	0.051695229	0.284812918	-0.04785543	0.322159447	-0.10642754

0.241147473	0.072473343	0.133500908	0.041002222	0.39637006	0.007701474
0.316615112	0.05077294	0.293505285	-0.236626953	6.93E-07	-0.205170953
0.677744884	0.036996799	0.444146041	0.388847285	5.69E-17	0.351224828
0.679747842	0.045461173	0.346989269	0.204850241	1.86E-05	0.228598573
0.109147659	0.00460344	0.924170582	-0.070316467	0.145483531	-0.312022416
0.94947569	0.01819572	0.706733042	-0.038938769	0.420585089	-0.035262254
0.630373548	-0.019330346	0.689367821	0.242904256	3.42E-07	0.287513126
0.531909357	0.109518946	0.02313134	0.252813482	1.07E-07	0.162651811
0.087908091	0.015002874	0.75639512	-0.327066796	3.53E-12	-0.175062146
0.386080435	0.252875359	1.07E-07	0.009066321	0.851300114	0.046497574
0.207514414	0.037753323	0.434879456	-0.100273705	0.037662564	-0.146433527
0.001774227	0.020749367	0.667878274	0.046134198	0.339890006	-0.119249818
0.215692704	-0.06252725	0.195636068	-0.071129653	0.140872932	-0.142397512
0.045914094	-0.012372937	0.798079303	0.201181466	2.64E-05	0.370076417
0.618503902	0.058448635	0.226462017	0.08997114	0.062317151	0.195792662
0.713167167	-0.029505106	0.541740722	-0.286766063	1.39E-09	-0.314545249
0.001135684	-0.028407365	0.556887072	-0.015864162	0.742889485	-0.298451064
0.176954005	-0.028764774	0.55193313	0.05035763	0.297476814	-0.023332474
0.946833113	0.026631153	0.581821834	0.034766686	0.472106292	-0.000784698
0.121428462	-0.00083719	0.986189457	0.192147108	6.06E-05	0.213631654
0.876746629	-0.010296462	0.831407993	0.065839552	0.172950947	0.151316118
0.271821234	-0.015591818	0.747151743	0.048489793	0.315779304	0.009050092
0.359982489	0.009768409	0.839934527	-0.042400152	0.380452198	-0.136873367
0.293489333	-0.035872932	0.458117359	-0.095255646	0.048381287	-0.16748109
0.331537375	0.05266499	0.275862114	0.013579715	0.778872834	0.260209952
0.497634644	0.404462563	2.35E-18	0.101681803	0.035044221	0.148769394
0.366936114	0.048769317	0.312994398	-0.131731766	0.006226146	0.011969351
0.585849049	0.048038543	0.320309232	-0.193143426	5.54E-05	-0.088526024
0.186100999	-0.002229974	0.963224827	-0.067351105	0.163275248	-0.199963452
0.18645774	-0.061934713	0.199913545	0.013880148	0.774111644	0.041883187
0.641583499	0.092822099	0.054435946	0.252526642	1.11E-07	0.176973961
0.624015586	-0.021753739	0.652829218	0.10118804	0.035944169	0.017131999
0.411924539	0.010898167	0.821716286	-0.011011889	0.819887556	0.14367346
0.968238496	0.012460115	0.796687578	0.447479941	1.46E-22	0.284163199
0.191827846	-0.077031973	0.110692085	-0.014620579	0.762413906	-0.022596327
0.914198333	0.030947228	0.522159157	-0.055396155	0.251689373	-0.071246348
0.522057096	-0.024226697	0.616380503	-0.02203871	0.648584509	0.009787185
0.259283454	-0.135970803	0.004735864	-0.301615965	1.71E-10	-0.292930541
0.635389466	-0.041457967	0.391137128	-0.275863011	5.99E-09	-0.302275998
0.481259519	-0.055557338	0.25031029	0.017466734	0.717971505	-0.012073653
0.496448166	0.221890011	3.39E-06	0.266301511	2.05E-08	0.128249953
0.103178855	0.045262781	0.349099781	0.014197529	0.769091058	0.242353656
0.02314788	-0.062895588	0.193010983	-0.134862258	0.005090812	-0.221754414
0.718870925	0.046799726	0.33296175	0.014379054	0.766223861	0.131881131
0.87032042	-0.020635375	0.669594855	0.106033429	0.027909444	0.205470599
0.054445894	0.069990055	0.147366273	0.346739678	1.36E-13	0.470908977
0.740671877	0.084615321	0.079660815	0.184925077	0.000114862	-0.012055021
0.128291254	0.045469762	0.346898081	-0.187343214	9.30E-05	-0.245151769
0.581636629	0.017283249	0.720809942	-0.102458048	0.033668188	0.121896141
0.598291945	-0.060764805	0.208558062	-0.178764078	0.00019439	-0.173152762
0.228493988	-0.125674873	0.009085644	0.142085201	0.003149474	0.365740755

0.204222986	0.166309566	0.000534636	-0.056028282	0.246311055	-0.232509069
0.367059129	0.004810337	0.920773447	-0.0541248	0.262752221	-0.10442363
0.028578406	0.05141365	0.287448135	0.006493311	0.893198859	0.19293175
0.307085185	0.057375825	0.235115055	-0.140889185	0.003415318	-0.160151078
0.999314725	0.160853922	0.00081499	0.228775518	1.64E-06	0.307289
0.106401892	0.075615233	0.117426176	0.054061391	0.263312609	0.125753733
0.10761877	0.030868085	0.523224305	-0.253166767	1.03E-07	-0.383939605
0.148052602	0.054397527	0.26035131	0.373895159	1.03E-15	0.148700957
0.001385136	-0.024116073	0.617991871	0.167125548	0.000501401	0.317334631
0.044105916	0.1207642	0.012206815	-0.14799523	0.002091503	-0.166699589
0.263857488	0.105694585	0.028416248	0.062429441	0.196337494	-0.119423178
0.023406844	-0.22572119	2.27E-06	-0.061916487	0.200046186	0.160616585
0.558783097	0.13436356	0.005258168	0.039697884	0.411577714	0.016100355
0.936880711	0.004423767	0.92712183	-0.004506271	0.92576652	0.024123074
0.823069757	0.092261969	0.055915324	-0.000667379	0.988990518	0.020863545
0.736215285	0.194098952	5.08E-05	0.253359255	1.01E-07	0.44327747
0.495992452	0.439660457	9.44E-22	-0.023506689	0.626900976	-0.249211329
0.963353173	0.067679228	0.161229664	0.004469253	0.926374604	0.080363432
0.547730435	-0.026025488	0.590441573	-0.027293813	0.572458652	-0.091992368
0.1238482	0.072077619	0.135640419	0.024601786	0.610930614	-0.064615892
0.637443368	-0.20742087	1.45E-05	-0.125036227	0.009446658	-0.099714395
0.449429825	-0.008720912	0.856903159	-0.243185136	3.31E-07	-0.30764832
0.12062984	-0.036064791	0.455715019	-0.101922791	0.034611995	0.091946135
0.221111113	-0.067019548	0.165361992	0.010651537	0.82568558	0.21579659
0.636157704	0.12366014	0.010268168	0.15456781	0.001303539	0.126873781
0.256485754	0.036594452	0.449119735	0.094402232	0.050437037	0.183633241
0.359144463	0.04856267	0.31505167	0.029985593	0.535176163	0.069047292
0.001292005	-0.019445103	0.687620353	0.044207053	0.36046694	-0.043839534
0.774287392	-0.01664423	0.730725001	-0.03429268	0.478171313	-0.215116101
0.934168909	-0.091761665	0.057264759	0.097606988	0.043077007	0.279012235
0.207814506	0.222290155	3.25E-06	-0.006775311	0.888590077	0.001525627
0.754411334	-0.027624554	0.567812204	-0.010230894	0.832465676	0.109678219
0.010774554	0.004118893	0.932131863	-0.090223595	0.06158328	-0.116790534
0.494960955	0.188708853	8.24E-05	-0.027337548	0.571843198	-0.039070192
0.230320523	0.033332825	0.490581802	0.101181464	0.035956286	-0.221847954
0.012472287	0.044281117	0.359662017	0.090857955	0.05977066	-0.031336514
0.925504621	-0.075679535	0.117113747	0.08015112	0.096938117	0.09578328
0.873902222	-0.134936668	0.005066256	-0.091439313	0.05814843	-0.036261856
0.759358284	0.029016112	0.548462372	-0.007351779	0.879180598	0.081021189
0.210819289	-0.010471206	0.828590669	-0.039225979	0.417163679	0.112235113
0.372682729	0.085581649	0.076270701	0.039967737	0.40840344	-0.345684328
0.195842576	0.026620079	0.581978915	0.216870964	5.67E-06	0.242741481
0.266122426	0.041385969	0.391961032	-0.04868606	0.313822201	0.214672378
0.09088531	0.019132528	0.692383986	-0.071067338	0.141222232	0.08998165
0.827213427	0.021174426	0.661492689	0.138709101	0.003952802	0.175807445
0.02076334	0.103427285	0.032014923	-0.074724903	0.121819408	-0.218841727
0.121947445	0.120371744	0.012492988	0.051977173	0.282190643	0.038097722
0.02890857	0.117048058	0.015163611	-0.00664846	0.890662777	0.077443112
0.000151679	0.165269883	0.000579946	-0.037312764	0.440262221	-0.147280103
0.680051792	0.035813509	0.458862847	0.103390679	0.032076079	0.044923397
0.730753377	0.00673	0.889330365	0.016209721	0.737492689	0.037278454

0.518801468	-0.26587147	2.17E-08	0.080401273	0.095896855	0.370843135
0.001397709	0.019609051	0.685126706	-0.091688029	0.05746563	-0.279041637
0.87470685	0.060390708	0.211378441	0.165139639	0.000585867	0.069540979
0.075142121	-0.064821343	0.179705406	-0.105749345	0.028333813	-0.019108806
0.688343076	0.129418856	0.007205716	-0.1263411	0.00872217	-0.397963981
0.979973592	-0.12243102	0.01105488	-0.156281711	0.001148815	-0.166463975
0.134153819	0.000840703	0.986131515	-0.002176009	0.964114167	-0.083872128
0.143274758	0.051516321	0.286485377	-0.013454157	0.780865116	0.355099823
0.036343199	-0.056848351	0.239454017	0.126406785	0.008687045	0.297888912
0.941432763	0.051560014	0.286076316	0.185918634	0.000105349	0.143727974
0.527008909	0.057857542	0.231201208	0.156753867	0.001109267	0.231297536
0.316965714	-0.220714974	3.83E-06	-0.12618825	0.008804396	-0.084824831
0.07913628	-0.20754412	1.43E-05	-0.195801967	4.35E-05	-0.129334264
0.666201371	0.045415035	0.347479363	-0.204542085	1.92E-05	-0.146088836
0.598050805	-0.022408161	0.64309837	0.134713469	0.005140234	0.047696935
0.513860656	0.239959735	4.77E-07	-0.105443384	0.028797047	-0.310713405
0.556544001	0.050059015	0.300354426	0.020486398	0.671840878	-0.038708692
0.193001766	-0.165682093	0.000561571	-0.116750831	0.015425225	-0.100844765
0.676918548	-0.050128025	0.299687776	-0.071939489	0.13639341	-0.112705764
0.067092402	0.065484797	0.175282467	-0.021906469	0.650552862	-0.079286799
0.335029952	0.079241497	0.100800567	0.073767674	0.12668448	0.009258504
0.665013464	0.07301159	0.130632722	-0.072884817	0.131303941	0.005924547
0.794435859	0.107824596	0.025357524	-0.016942846	0.726085961	-0.014517941
0.487961244	0.054028495	0.263603651	0.054919575	0.255797837	0.058881711
0.800629786	0.078441229	0.104298826	0.059231999	0.220288251	0.094923755
0.647152611	0.036661567	0.448287901	0.021145043	0.661933314	-0.056044894
0.147611033	0.021458013	0.657245973	-0.145046411	0.002570214	-0.329729109
0.370210034	0.052762609	0.274971807	-0.097612618	0.04306492	-0.028915233
0.8181723	-0.003710807	0.938842143	0.340985958	3.61E-13	0.320569541
0.737626781	-0.049535557	0.305443219	0.125933517	0.008942965	0.24629809
0.875688768	-0.015948431	0.74157223	-0.011950588	0.804830727	-0.096974357
0.51139919	0.152365497	0.001530474	0.408892063	9.25E-19	0.298249929
0.013454716	0.092421694	0.055490104	0.173623934	0.000297608	0.197949464
0.400440873	0.093847718	0.051811486	0.022544041	0.64108548	0.164851536
0.707315235	-0.033968065	0.482349199	0.135886475	0.004762059	0.077544113
0.048400437	-0.010557169	0.827205533	-0.098660279	0.040864796	-0.205093171
0.959753803	0.09699953	0.0443981	-0.204113328	2.00E-05	-0.143026524
0.003073007	-0.00261499	0.956881118	0.13867254	0.003962435	-0.050378219
0.91116104	0.032394686	0.502876291	-0.129453493	0.007190084	-0.278201828
0.211769082	0.092555095	0.05513703	0.004338637	0.928520511	-0.036711752
0.790672733	0.089076712	0.064975423	0.074623544	0.122327565	0.059370637
0.167007219	0.006797944	0.888220344	-0.058092742	0.229307111	-0.082482209
0.471677441	0.018503849	0.70200167	0.182826605	0.00013767	0.34621531
0.000558939	-0.011515617	0.811799101	-0.129807762	0.00703193	-0.193678627
0.066612394	-0.093464678	0.052779033	-0.103518165	0.031863522	-0.119462871
0.870733332	-0.146906327	0.002257874	-0.080924589	0.093747408	0.115711921
0.976870351	0.28097301	3.04E-09	0.112802428	0.019294875	0.161009611
0.124446532	-0.078453345	0.104245153	-0.132109273	0.00607813	-0.136450578
0.726950999	0.036309732	0.452658307	0.1211954	0.011899078	0.129251407
0.659713526	-0.041661306	0.388815915	0.269679123	1.34E-08	0.25136227
0.457900464	-0.052918707	0.273552231	0.015904071	0.742265562	0.004262408

0.21583471	0.049459754	0.306184839	-0.102534116	0.033535856	-0.058536732
0.141379499	0.041790072	0.387350314	0.032049637	0.507438691	0.004710422
0.483723233	0.165821211	0.000555493	-0.037053025	0.443453486	-0.129999763
0.033600042	-0.027824209	0.565016075	-0.012171799	0.801292727	0.008809029
0.029078459	-0.091566096	0.057799538	-0.164864931	0.000598539	0.060880307
0.733594287	0.011599044	0.810461409	-0.027070581	0.575604869	-0.013196944
0.465865292	0.084461629	0.080211088	0.12971938	0.007071092	0.102656374
0.001277288	0.055990332	0.246631665	-0.057384625	0.235043142	-0.158466235
0.357961298	0.078814124	0.102656994	-0.015953362	0.741495181	0.182164646
0.352257792	0.0149478	0.757261294	0.248271325	1.84E-07	0.158957624
0.767772775	0.104528808	0.030220677	0.121041302	0.012008258	0.200548329
0.090472594	0.004775372	0.921347459	-0.201887284	2.47E-05	-0.336780577
0.267726942	-0.047967994	0.321021245	0.076217825	0.114523762	0.171422922
0.047277891	-0.054610532	0.258486706	0.004336519	0.928555304	0.096432553
0.057596454	0.039932361	0.408818729	0.09794514	0.042356052	0.217176421
0.684525398	-0.037737498	0.435072149	0.020934646	0.665091874	0.008479285
0.539212209	0.048142469	0.319262226	0.103784761	0.031422916	0.03767338
0.106462808	0.062672685	0.19459648	0.030296363	0.530951718	0.001389784
0.576175886	0.102048163	0.034388933	-0.001079745	0.982188793	0.065519268
0.645846557	0.113984226	0.018056484	0.019868135	0.681193008	0.064698698
0.363900265	0.33109806	1.85E-12	0.056771768	0.240088633	-0.134647935
0.247496536	0.111811787	0.020389301	0.017361971	0.719591675	-0.402324205
0.399237657	-0.096760263	0.044927725	-0.051953838	0.282407059	-0.006730887
0.297343084	0.054487689	0.259560923	0.003891457	0.935871105	0.072228543
0.724224301	0.040594275	0.401089717	0.166011577	0.000547274	0.167421019
0.217913076	0.019423125	0.687954884	-0.212303815	8.98E-06	-0.190187782
4.13E-05	0.018157752	0.707316836	-0.166138273	0.000541867	-0.296967437
0.031870292	-0.001567554	0.974144385	-0.236614938	6.94E-07	-0.429300768
0.931507311	0.085377811	0.07697588	0.227068626	1.96E-06	0.321360204
0.743692386	0.032317328	0.503897266	0.064906243	0.179134847	0.099463803
0.181986664	-0.107665657	0.025575529	0.252121077	1.17E-07	0.406273777
0.926238596	-0.019735736	0.683202164	0.03690273	0.445306075	0.074228487
0.041162767	0.038496858	0.425881319	-0.022402242	0.643186123	0.127759918
0.224709194	0.082727293	0.086636373	0.113419863	0.018638943	0.175181978
0.024087044	-0.006807552	0.888063403	0.040859771	0.398014308	-0.030245126
0.925497629	0.215241453	6.69E-06	0.15730989	0.001064304	0.00393644
0.406397999	0.111877602	0.020314952	0.091780981	0.057212161	0.158449981
0.506242727	0.090133723	0.06184371	0.105295447	0.029023348	0.209999841
0.709689831	0.151556454	0.001622571	-0.033264673	0.49146949	-0.060512855
0.059767886	0.203881033	2.04E-05	0.037721528	0.435266657	0.258335816
0.119168171	0.05251471	0.277236514	0.03416158	0.479856234	0.105879388
0.6589146	-0.048576773	0.314910993	-0.147074275	0.00223145	-0.096790294
0.155654326	-0.007159202	0.882322153	-0.054414297	0.260204173	0.054008884
0.116821924	-0.009041275	0.851706141	-0.072404369	0.133871939	-0.085663095
0.61735102	0.011346072	0.814519321	-0.12189717	0.011412884	-0.082198182
0.477070269	0.029306577	0.544464728	0.142662185	0.00302807	0.19277548
0.152089223	-0.01485058	0.758791069	0.133572873	0.005533679	0.144184756
0.095933708	0.033938421	0.482731713	-0.00126904	0.979066881	-0.040874669
0.199859084	0.006127853	0.89917692	-0.167002736	0.000506277	-0.011194687
0.089683989	-0.000154357	0.997453558	-0.038190777	0.42957228	-0.125189064
0.129797359	-0.037813513	0.434147003	-0.079520245	0.099604174	-0.218543997

0.495188429	0.108407314	0.024571899	-0.007492159	0.876891757	0.012258112
0.019589115	-0.011978665	0.804381454	0.107236651	0.026172052	0.314016627
0.35799822	-0.074163651	0.124653977	-0.072829184	0.131599332	0.136604286
0.625364535	0.128336481	0.007709781	0.023551401	0.626245422	0.203224851
0.871956222	-0.041884696	0.386275458	0.027828154	0.564960889	-0.175136933
0.931809217	0.170652375	0.000378656	0.083719273	0.082912468	0.083263105
0.794576434	-0.025660246	0.595667792	-0.038661477	0.423903872	0.059984456
0.266470506	-0.011796749	0.807293546	0.102543114	0.033520231	0.131179071
0.159645298	0.106645052	0.027014289	-0.044873758	0.353261778	-0.37541383
0.229149015	0.032113303	0.50659525	0.054795462	0.256875362	0.250081181
0.793616455	-0.078137527	0.105651301	-0.219362876	4.40E-06	-0.063622336
0.972246483	-0.068839242	0.154152403	-0.044995971	0.35195092	0.071090903
5.40E-05	-0.012934258	0.789129681	0.338049913	5.90E-13	0.242300867
0.69197919	-0.144819025	0.002610987	-0.052357847	0.278676082	0.076111632
0.292142194	0.02429463	0.615391886	-0.094594636	0.049967304	0.024809438
0.115111698	-0.052892465	0.273790529	-0.200357161	2.85E-05	-0.33645082
0.922803195	0.053971182	0.264111259	0.429119786	1.08E-20	0.278254081
0.365219887	-0.035112677	0.46770607	-0.146863919	0.002264591	-0.126128121
0.752946749	-0.176259377	0.000239578	-0.268608594	1.53E-08	-0.365705662
0.731727748	0.019875254	0.681085041	0.065040676	0.178234162	0.139894034
0.961149926	-0.006029932	0.900779662	-0.041581986	0.389720395	-0.034506267
0.825662434	0.04699741	0.330921445	0.114023608	0.018016439	0.269456877
0.857483118	0.36475032	5.61E-15	0.012354903	0.798367273	-0.030756136
0.923036779	0.032909998	0.496103017	-0.134161661	0.005327319	-0.187135057
0.528486284	0.085688331	0.075903731	0.145339706	0.00251848	0.103973321
0.74865526	0.240662767	4.41E-07	0.233923895	9.35E-07	0.20224155
0.208126017	0.086397345	0.073501154	0.031391484	0.516200809	-0.068693613
0.018211966	0.023515151	0.626776886	-0.07497214	0.120586796	-0.056733851
0.121616198	0.06188711	0.200260113	-0.078521818	0.103942249	0.024654797
0.406354077	0.018490665	0.702203875	-0.245863854	2.43E-07	-0.208932633
0.054184453	0.07667314	0.112368267	-0.072426424	0.133753211	0.013318273
0.203708768	0.058503657	0.226024411	-0.055104957	0.254194226	-0.022243019
0.368817495	-0.07641603	0.113581493	-0.007961474	0.869247268	0.162108467
0.054073119	0.01166186	0.809454546	-0.101365529	0.035618438	0.244369035
0.016290086	-0.005214414	0.914142932	-0.202840662	2.25E-05	-0.355749559
0.69107234	-0.022321457	0.64438416	0.160290653	0.000850617	0.199610752
0.072992605	-0.048106946	0.319619852	-0.134292106	0.005282548	-0.147783816
0.915498151	0.040335402	0.404102044	0.391963429	3.05E-17	0.327566241
0.846344754	0.214839447	6.97E-06	0.016042468	0.74010319	0.08333058
0.482915233	0.106686991	0.026953824	-0.187835384	8.91E-05	-0.215616589
0.761407692	0.087025053	0.07142616	0.132477482	0.005936812	-0.087270916
0.528225004	0.025373332	0.599787918	-0.183292417	0.000132267	-0.227625518
0.105291338	0.041212973	0.393944975	-0.113681868	0.01836653	-0.210943167
0.00863874	-0.086771542	0.072258349	0.039634488	0.412325553	0.284567503
0.170154999	0.165979557	0.000548649	0.021222621	0.660770197	0.136778281
0.108379802	-0.013033092	0.787556714	-0.096977313	0.044447055	-0.037763549
0.789785758	-0.045287542	0.348835927	0.091887317	0.056923334	0.088222696
0.494766078	-0.084772148	0.079102473	-0.055565319	0.250242144	0.02726476
0.003074756	-0.001400396	0.976900714	-0.102990321	0.032751482	-0.422077185
0.885781556	-0.14454707	0.002660525	0.038379804	0.427290671	0.137003277
0.887701436	0.067332178	0.163393837	0.193327249	5.45E-05	0.071420349

0.581925256	-0.002062841	0.965979332	0.091921278	0.056831344	0.108273419
0.932417469	0.177722253	0.000212119	0.004794448	0.921034297	0.066235688
0.212756327	0.067012465	0.165406787	0.059756184	0.216224864	-0.002378657
0.675787569	0.008387297	0.862321715	0.138946236	0.003890837	0.075398136
0.9590414	-0.054547593	0.259036702	-0.029056905	0.547900069	0.07945333
0.002284028	0.035164222	0.467052466	-0.14939255	0.001894434	-0.285965562
0.865749083	0.122146354	0.011244522	-0.007428202	0.877934429	-0.045927317
0.76167272	0.034998981	0.469149515	0.221269684	3.61E-06	0.321426768
0.452182456	0.058760008	0.223993487	0.064269807	0.183444719	-0.084328439
0.016994591	0.039500391	0.413910054	0.049637843	0.304444399	-0.03086309
0.343110403	0.059961408	0.214648739	-0.257361669	6.21E-08	-0.369250445
0.061992012	-0.171214618	0.000361896	0.027486095	0.569755166	0.084333803
0.209075108	0.030853876	0.523415657	0.244294775	2.91E-07	0.190874308
0.500516031	-0.003292601	0.945723329	-0.168693441	0.000442863	-0.086102698
0.876395923	0.130261854	0.006833768	0.1840914	0.000123461	-0.003030883
0.424423818	0.046536279	0.335693335	-0.145742742	0.002448937	-0.132827093
0.340652933	8.95E-05	0.998524088	-0.088540589	0.066613006	0.063562759
0.571817372	-0.037948765	0.43250371	-0.003763686	0.937972394	0.046215017
0.008174989	0.095622041	0.047520463	-0.136719937	0.004508802	-0.356163508
0.763463334	0.061232046	0.20507376	0.028245427	0.559138722	0.132505358
0.177076363	0.111086086	0.021224903	0.017497649	0.717493648	-0.059896865
0.564673957	0.006703425	0.889764567	-0.10933123	0.023369345	-0.227011814
0.851106454	0.085565914	0.076324949	0.223199233	2.95E-06	0.132739118
6.01E-06	0.019161162	0.691947097	0.041458971	0.391125653	-0.006675942
0.642897055	0.068805701	0.154353682	0.073390402	0.128642855	0.257543514
0.309132309	0.042548589	0.378785275	-0.131748516	0.00621951	0.027606449
0.617014916	-0.029187676	0.546099405	0.049932702	0.301577194	0.218244859
0.000183396	-0.063597508	0.188080087	0.081410253	0.09178723	0.36561759
0.551548599	0.049981493	0.301104486	0.059659694	0.216968772	0.207923506
0.701267513	-0.025912089	0.592061934	0.01713898	0.723044406	-0.064062579
0.868894309	0.090107356	0.06192029	0.256424547	6.96E-08	0.130861
0.446557019	-0.011193653	0.816966684	0.022154863	0.646857646	0.142496925
0.48743137	0.003039822	0.949884556	0.144060715	0.002751256	0.129645813
0.661170795	-0.053498508	0.268323203	-0.09670729	0.045045699	-0.078996597
0.553935997	0.084750915	0.079177883	-0.206406453	1.60E-05	-0.291937446
0.944458881	0.107603183	0.025661662	0.07614294	0.114881359	0.000222169
0.801442864	0.187097513	9.50E-05	0.093737376	0.052088678	0.053726591
0.268687044	0.163469697	0.000666918	0.047459849	0.326180163	0.027929281
0.857705534	0.037651887	0.436115441	0.253215786	1.02E-07	0.241759346
0.995635313	0.093545483	0.05257368	0.45396172	3.00E-23	0.315279353
0.79392423	0.062643513	0.19480468	0.002349509	0.961255036	0.031138772
0.317581055	0.086089914	0.074535198	0.080625621	0.094970612	-0.019710141
0.14657628	0.075146482	0.119723469	-0.054754281	0.25723358	-0.074713401
0.507891984	0.05729504	0.235775971	0.039596941	0.41276885	0.021838265
0.18830855	0.040294301	0.40458155	0.062240925	0.197694603	-0.023078353
0.308471715	0.08230588	0.088258562	-0.023620891	0.625227175	-0.166315499
0.048789518	0.011976123	0.804422127	-0.237119549	6.56E-07	-0.29975457
0.160932857	0.048457604	0.316101039	0.054730713	0.257438746	0.063480711
0.000359004	0.212160325	9.11E-06	0.036870087	0.445709022	-0.034522529
0.365078941	0.103138046	0.032500871	-0.093768385	0.052010655	-0.15031221
0.015820808	0.129693815	0.007082456	0.011647867	0.809678812	-0.101214479

0.126019203	0.065023135	0.178351492	0.217467323	5.34E-06	0.293879404
0.524549014	-0.070924388	0.142026041	-0.256949049	6.53E-08	-0.237257797
0.014081009	0.052428044	0.278031243	-0.006227599	0.897544723	0.142663065
0.008989834	0.05758537	0.233406845	-0.11663818	0.015525409	-0.444419936
0.728028131	0.267368529	1.79E-08	0.163678464	0.000656246	0.098123963
0.909349242	-0.021961071	0.649739845	-0.250794918	1.36E-07	-0.171720147
0.173830017	-0.003340694	0.944931799	-0.021593496	0.655220996	0.055014832
0.042187655	-0.03911344	0.418502356	-0.097013018	0.0443684	0.122356441
0.463761264	0.021996168	0.64921746	-0.020111698	0.677502823	-0.041158155
0.077600939	0.046846533	0.332477918	-0.228611064	1.66E-06	-0.395917239
0.501401626	0.087066099	0.071292157	0.050210293	0.298894324	0.13400575
0.78641231	0.245970862	2.40E-07	0.069314968	0.151318987	0.12164658
0.502058307	-0.01920818	0.69122993	-0.065132639	0.177619953	-0.233858266
0.089532612	0.06496194	0.178761277	0.026302499	0.586491914	-0.019462857
0.030863656	0.088417905	0.066992465	0.079431338	0.099984531	-0.015105363
0.24779873	0.140834577	0.003427928	-0.106430093	0.027326041	-0.193556845
0.221000668	0.106565508	0.027129292	0.237664117	6.18E-07	0.172959835
0.544912004	-0.009362622	0.84649962	0.097841167	0.042576635	0.025609977
0.152374735	-0.081650318	0.090830497	-0.200763857	2.74E-05	-0.106709564
0.969310068	0.019242646	0.690704391	-0.002413931	0.960193547	0.058422129
0.007013686	0.193690482	5.27E-05	0.025248606	0.60158299	-0.054020508
0.886141139	-0.155870765	0.001184289	-0.054336192	0.260889945	-0.190302977
0.121833526	-0.058925208	0.222691613	-0.117766878	0.014546951	-0.040165157
0.140965378	0.011217581	0.816582356	0.3443868	2.03E-13	0.075545162
0.084082639	-0.010207339	0.832845713	0.008400666	0.862104445	-0.033610279
0.802483084	0.011428971	0.813188989	0.02481739	0.607807649	0.089584971
0.357782016	0.042374668	0.380738827	-0.102265305	0.03400549	-0.130627766
0.043369196	0.006999138	0.884934698	-0.085562089	0.07633814	-0.204642179
0.933769221	-0.036220113	0.453775355	-0.178886415	0.000192402	-0.04108191
0.474234884	0.109740315	0.022853362	0.01487269	0.758443074	-0.057442871
0.06936093	0.094509319	0.050175143	0.021375644	0.658478329	0.314958423
0.950205634	-0.05278763	0.274743926	0.048771392	0.312973788	0.291407367
0.829980321	0.0540203	0.263676191	-0.166448755	0.000528825	-0.107073606
0.56620231	0.080492998	0.09551729	-0.143444713	0.002870213	0.024923872
0.345251816	-0.034394739	0.47686188	0.111953184	0.020229861	0.10982707
0.606495817	0.144266712	0.002712488	0.049460422	0.306178296	0.122697099
0.711020681	0.034386878	0.476962657	-0.026435296	0.584602824	0.157044226
0.962522378	0.076305004	0.11410856	0.044862987	0.353377461	0.171432452
0.101947355	0.017272552	0.720975539	-0.219577477	4.30E-06	-0.187638182
0.861237491	-0.03344244	0.489155865	0.017769272	0.71329986	0.002314499
0.163392887	-0.075368985	0.118628674	-0.088964701	0.065314798	-0.313622458
0.168630464	0.046865151	0.332285595	-0.186299446	0.000101903	-0.081966451
0.255428491	0.062839339	0.193410188	-0.020614434	0.669910394	-0.050522207
0.124153078	0.090112014	0.061906754	0.151148998	0.001670853	0.057320882
0.957923181	-0.009032052	0.85185568	0.140744338	0.003448859	0.114945874
0.375708531	0.012808954	0.791125154	-0.147821517	0.00211727	-0.071728137
0.514296787	-0.030908408	0.522681478	-0.047876191	0.321949325	-0.096112893
0.178809686	0.026500559	0.583675467	0.054442146	0.259959963	0.221743919
0.321987404	0.159019372	0.000936377	0.186916345	9.65E-05	0.119047114
0.843904966	-0.008360243	0.862761416	0.023887416	0.621328335	0.085075545
0.3634063	0.229078111	1.58E-06	0.380042503	3.18E-16	0.452530557

0.577747042	0.030156972	0.532844443	-0.03969607	0.411599104	-0.172017512
0.247067123	0.052248336	0.279684084	-0.039624294	0.412445882	-0.37034287
0.123075388	0.044066375	0.361998929	-0.214478098	7.23E-06	-0.470478807
0.058640926	0.018343333	0.704464989	-0.078471349	0.104165442	-0.137580519
0.998616958	-0.026100022	0.589377664	-0.024808146	0.6079414	-0.074193182
0.050781339	-0.150037535	0.001809332	-0.243208181	3.30E-07	-0.129654113
0.548780875	0.111498768	0.020746151	-0.134677407	0.005152277	-0.075522935
0.149400063	-0.013057503	0.787168334	-0.204017258	2.02E-05	-0.236025683
0.243685256	0.024514004	0.612204139	0.333715952	1.21E-12	0.267939536
0.883157958	0.419477168	9.36E-20	0.03272083	0.498583794	0.001972131
0.505445584	-0.01435681	0.76657504	-0.02996143	0.535505334	-0.043958528
0.598584023	-0.034190549	0.479483641	0.04937018	0.307062708	0.061429448
0.008688818	-0.056940393	0.238692866	-0.047057923	0.330298507	-0.181083593
0.623385542	-0.001042481	0.982803385	0.063387838	0.189543188	0.143847582
0.091970106	0.11627131	0.01585564	0.04291792	0.374657234	0.017698431
0.187910152	-0.033872193	0.483586877	-0.002591754	0.957263899	0.02244264
0.012810274	0.041419743	0.391574417	0.0071972	0.881702124	0.151953264
0.955644652	-0.011236051	0.816285734	0.321795553	8.14E-12	0.129676964
0.288134701	-0.016506912	0.732861574	-0.049380013	0.306966259	-0.081600939
0.211387302	0.153475369	0.001411929	0.03728629	0.4405869	0.037087921
0.481170425	0.046833823	0.332609257	0.105261373	0.029075687	0.038729845
0.062175318	0.003982297	0.93437744	-0.061591577	0.202421518	-0.211528334
0.433699978	0.023527336	0.626598213	0.235389138	7.96E-07	0.341031751
0.277063833	-0.099912555	0.03836024	-0.299680306	2.26E-10	-0.260296404
0.523809398	-0.084213836	0.081104751	-0.193269785	5.48E-05	-0.068790284
0.976960702	-0.022780716	0.637585709	-0.123551667	0.010335542	-0.076950636
0.612218289	-0.017875422	0.711663272	-0.176755013	0.000229923	-0.03528839
0.132298884	-0.008463869	0.861077465	0.083074467	0.08531806	-0.081063229
0.458439696	-0.178420889	0.000200071	-0.10100113	0.036289924	0.070305983
0.731008815	0.029501905	0.541784595	-0.047205811	0.328779281	-0.172864865
0.218650598	-0.038564822	0.425064272	-0.000966682	0.984053578	0.231824696
0.24134614	0.039664683	0.411969264	0.05204374	0.281573921	-0.154062655
0.44780054	0.038523069	0.425566106	-0.163316597	0.000674847	-0.139751742
0.040469965	-0.046092097	0.340331361	-0.181103126	0.000159514	-0.281713012
0.753752832	0.062362623	0.196817734	-0.074560376	0.122645089	-0.110314585
0.272477509	0.017763004	0.71339654	0.123218831	0.010544727	0.111700091
0.224090161	0.05515918	0.253726504	-0.088347434	0.067211231	-0.081371226
0.781536229	-0.070669691	0.143466873	-0.132033417	0.006107617	-0.049067257
0.092101908	-0.064801367	0.179839845	-0.09633683	0.045878021	-0.09110079
0.997252664	0.150397194	0.001763411	-0.030772713	0.524509348	-0.239021879
0.000933791	0.036697775	0.447839491	-0.118723711	0.013760346	-0.337178468
0.85073939	-0.009019464	0.852059777	-0.061827275	0.200696361	-0.289775453
0.650774759	0.068876505	0.153929028	0.056349232	0.243611268	0.086646118
0.440403282	0.084940431	0.078506879	0.056875308	0.239230917	0.01509725
0.676879184	0.057459902	0.234428605	-0.094786621	0.049502244	-0.142424417
0.961901394	0.134549926	0.005195055	0.018320623	0.704813761	0.021282136
0.167305484	0.019959417	0.679809111	-0.074639759	0.12224616	-0.078655802
0.070351995	0.082887596	0.086025635	-0.14864693	0.00199737	-0.042710188
0.153307568	0.057016731	0.238062869	0.030611712	0.526682316	0.267914548
0.029775593	0.03592432	0.457473216	0.06750957	0.162284924	0.053024821
0.973176606	0.09844352	0.041312042	0.005292801	0.912857364	0.020383446

0.083059301	-0.050826328	0.292997328	-0.145731258	0.002450894	-0.305699695
0.054909213	0.01296729	0.788603877	-0.216690474	5.78E-06	-0.248917666
0.003304991	-0.001439839	0.976250292	0.084686765	0.079406064	-0.110118494
0.452530895	-0.039384084	0.415287243	-0.057934089	0.230583554	-0.054525319
0.73529424	0.003879605	0.936066004	0.00432471	0.928749341	0.100301437
0.909630501	0.106727598	0.02689539	0.090341825	0.061242053	0.049283419
0.80871668	0.103058507	0.032635602	0.09773767	0.042797171	0.275088875
0.994977719	0.015815712	0.743647179	0.04650664	0.33600155	-0.087913475
0.888513743	0.005625243	0.907407736	-0.007681188	0.873811317	-0.065387494
0.146017506	0.080544723	0.095303781	-0.027951879	0.563231543	0.078350636
0.747497179	-0.052425356	0.278055913	-0.042781775	0.376175698	0.057509337
0.458049409	0.004135929	0.931851833	0.003342565	0.944901004	0.014389716
0.096567227	0.078074046	0.105935746	0.146889192	0.002260586	-0.058419112
0.315696929	0.03324428	0.491735285	0.045167065	0.350120915	-0.044969721
0.004839907	0.047749767	0.323230244	0.044037115	0.362318081	0.183963814
0.010589642	-0.017673468	0.714778067	0.007421585	0.878042305	0.00553228
0.172096867	0.006597941	0.891488445	-0.201792269	2.49E-05	-0.066164535
0.247480735	0.033757316	0.485072149	0.070421076	0.144884044	0.185233768
0.473271739	-0.15783868	0.001023108	-0.108182738	0.02487215	-0.018950056
0.33422671	-0.075459387	0.1181861	-0.103299279	0.032229213	-0.065162779
0.33525177	-0.111129493	0.0211741	0.006238159	0.897371947	0.037317777
0.646990006	-0.023813188	0.622413118	-0.064293412	0.183283512	-0.10048451
0.190043932	-0.062782243	0.193816011	0.050733504	0.293880872	0.295666551
0.995460233	0.165667035	0.000562233	0.108970342	0.023832873	0.085927187
0.921586269	-0.038853685	0.421601801	-0.109111712	0.023650358	-0.022350972
0.810337732	0.056903494	0.238997799	-0.275435401	6.34E-09	-0.209766677
0.969295468	-0.000855052	0.98589483	0.118852187	0.01365763	0.123066427
0.559709258	-0.140781555	0.003440213	-0.105373763	0.028903358	-0.063077948
0.297905461	0.044735092	0.35475284	0.005448776	0.910299978	-0.133066478
0.746027325	-0.010585547	0.826748398	0.030761549	0.524659875	0.088022037
0.233027548	0.073637631	0.127356889	-0.211977878	9.28E-06	-0.083561091
0.162779777	-0.038359467	0.427535808	0.202122981	2.41E-05	0.071451837
0.31169217	0.003862034	0.936354955	-0.374716865	8.80E-16	-0.44944587
0.690749904	-0.047335472	0.327451027	-0.049585954	0.304950818	-0.024769126
0.102128835	0.063362119	0.189723239	-0.164464397	0.000617472	-0.276110247
0.042610386	0.012220425	0.800515565	0.02984018	0.537158611	0.089846919
0.00267215	0.042977722	0.373991441	-0.186821315	9.74E-05	-0.419855493
0.139136521	0.021547042	0.655915043	-0.066239511	0.170350134	0.024658171
0.514020367	-0.009057832	0.851437736	-0.155291006	0.001236052	-0.155392818
0.153268563	0.091077274	0.059154328	0.187716313	9.00E-05	-0.105282212
0.40935386	0.22158084	3.50E-06	0.373285999	1.15E-15	0.363815084
0.871988539	0.057750697	0.232065297	0.148706542	0.001988956	0.184414179
0.038150144	0.106365808	0.027419872	0.032815671	0.497339213	0.127858819
0.426538184	0.208302851	1.33E-05	0.112476134	0.019649564	0.131467991
0.90858888	0.019552174	0.685991413	-0.254356802	8.93E-08	-0.276471261
0.219966424	-0.019094444	0.692965209	-0.055580414	0.250113284	-0.069601614
0.731769089	-0.031564254	0.513893142	-0.174717357	0.000272099	-0.139199578
0.943545913	0.097913749	0.042422548	0.106559724	0.027137671	0.141476608
0.243727171	0.050784252	0.293397611	-0.006535608	0.892507355	0.132468983
0.124262263	0.0691713	0.152170473	0.138764779	0.003938174	0.09296188
0.047123173	0.021060187	0.663206498	0.024561954	0.611508357	0.023966286

0.388567343	0.059974632	0.214547464	-0.068246363	0.1577396	-0.0330002
0.248842782	0.174916603	0.000267676	0.079061788	0.101577915	0.274143355
0.65413656	-0.03390088	0.483216356	-0.059838732	0.2155899	-0.047986586
0.291972344	0.012747353	0.792106657	-0.073496316	0.128090714	0.094303062
0.790516783	0.004007115	0.933969413	0.002898955	0.952204091	0.062554568
0.584141242	0.010856634	0.822384402	-0.20518828	1.80E-05	-0.166361746
0.342701419	-0.019231367	0.690876358	0.006510411	0.89291928	0.028868424
0.671232405	0.092953911	0.054092582	0.122998762	0.010685091	0.1990404
0.029761754	0.015999505	0.740774235	-0.103940806	0.031167444	0.009510093
0.896210809	0.152922151	0.001469921	0.03510218	0.467839236	0.057466142
0.420416909	0.135848991	0.004773745	-0.10733292	0.026037159	-0.082146359
0.488074315	0.023410096	0.628318181	-0.059886831	0.215220539	-0.024215832
0.274234162	0.025407998	0.599289417	-0.066347322	0.169654096	-0.116821743
0.863047756	-0.148126771	0.002072183	-0.074739994	0.12174389	0.089542492
0.338012441	0.085848421	0.075355739	0.012281149	0.799545305	-0.095711324
0.530467714	-0.096348883	0.045850741	-0.158942098	0.000941839	-0.194805101
0.522472336	0.024762166	0.608606875	0.179938305	0.000176075	0.130760915
0.575784208	0.042937936	0.374434306	-0.038280861	0.428484066	-0.126796134
0.656559461	0.020424418	0.672776161	-0.311597628	3.90E-11	-0.169640207
0.391621955	-0.097986302	0.04226899	-0.059099715	0.221322264	-0.211042366
0.107405221	-0.028563724	0.554717168	-0.136168964	0.004674819	-0.183096948
0.949606482	-0.02453371	0.611918157	-0.042375064	0.380734368	0.030104375
0.176716496	0.042922896	0.37460181	0.020378906	0.673463264	0.045652037
0.090013776	0.058306357	0.227596394	0.342998737	2.57E-13	0.275058136
0.241599555	-0.005341531	0.912058279	-0.232311477	1.12E-06	-0.143563391
0.51852672	0.024732539	0.60903585	0.223198189	2.95E-06	0.132127588
0.244221629	-0.114485579	0.017552444	-0.13035895	0.006792051	-0.016396087
0.798729125	0.170042556	0.000397649	0.033154501	0.492906316	0.11461926
0.102007467	-0.072475222	0.133490815	-0.21258461	8.73E-06	-0.246716609
0.72442546	0.16963825	0.000410727	0.189811652	7.48E-05	0.160149327
0.704654236	0.129388509	0.007219437	0.088891057	0.065538717	-0.082266066
0.41324967	-0.019668341	0.684225744	-0.078958225	0.102028043	-0.123584461
0.787435467	0.013007281	0.787967411	0.003886895	0.935946124	0.079529801
0.232423112	0.096397226	0.045741454	0.031159757	0.519304328	0.147054612
0.311075298	0.051582711	0.285863981	0.054509502	0.259369947	-0.051048488
0.080569847	-0.013551277	0.779323949	-0.040769151	0.399062418	-0.102261525
0.940333474	0.040762114	0.399143885	0.090173072	0.061729574	0.322712109
0.296476202	-0.026872659	0.578401169	-0.102573987	0.033466671	-0.171302999
0.55462484	0.157533755	0.00104668	0.168684971	0.000443162	0.089682335
0.407543055	-0.103317671	0.032198349	-0.102758412	0.033148235	0.073115051
0.818045944	0.026718022	0.580590347	0.071030394	0.141429638	0.166786454
0.985883997	-0.234199034	9.07E-07	-0.062836976	0.19342697	0.097542142
0.003895258	-0.082751207	0.086545041	-0.224220017	2.65E-06	-0.037586748
0.625751327	0.112059442	0.020110759	-0.00947927	0.844611294	0.072457164
0.51379561	-0.046065846	0.34060674	-0.123732742	0.010223291	0.011550062
0.15801425	-0.143112114	0.002936369	-0.108839218	0.024003248	0.158446873
0.055307067	0.247588197	1.99E-07	0.277005963	5.15E-09	0.279106713
0.011605689	0.083901989	0.082240854	0.130688248	0.006652261	0.044218916
0.051673825	0.028381791	0.557242365	0.009605924	0.842562018	0.02126096
0.002618056	0.065592683	0.174570964	0.034809241	0.47156387	0.017527393
0.057835407	0.063212321	0.190774383	-0.232057448	1.15E-06	-0.340888515

0.877923436	-0.042225414	0.382420216	-0.170441142	0.000385137	-0.205951428
0.789847299	0.01565867	0.746104778	-0.157745515	0.001030257	-0.1670943
0.008370778	0.071120125	0.140926296	0.026003373	0.590757414	-0.059916851
0.777143334	0.103747116	0.031484816	-0.037668115	0.435917575	-0.228696422
0.200476288	0.043866142	0.364186473	-0.008609879	0.858705811	0.000862972
0.09912755	0.107441164	0.025886204	0.030720522	0.52521325	0.05494135
0.833382876	-0.037887298	0.433250087	-0.07359424	0.127581865	-0.019509868
0.594038117	-0.01826982	0.705594182	0.084981648	0.07836156	0.089603339
0.206130345	-0.02182267	0.651801429	-0.018055203	0.708894479	-0.240451903
0.246059337	0.021359927	0.658713573	-0.031922353	0.509127155	-0.08366436
0.841217292	0.072184118	0.135062044	-0.023130739	0.632424584	-0.054039501
0.31410907	0.098062138	0.042108985	0.020935029	0.665086127	0.068593813
0.030259942	-0.025789738	0.593812493	-0.085413446	0.07685222	-0.133280249
0.013618969	-0.048571499	0.314963595	-0.218115155	5.00E-06	-0.2534887
0.368743817	0.053176451	0.271219214	-0.203376061	2.14E-05	-0.308421335
0.12829154	0.036902961	0.445303222	-0.010111909	0.834385798	-0.015122042
0.046261787	0.151076122	0.001679626	0.379034742	3.86E-16	0.251417473
0.763069533	0.0998463	0.038489414	-0.037465449	0.438392422	-0.020842875
0.242755549	-0.084798145	0.079010228	0.038285877	0.428423512	0.200869055
0.590000259	-0.015729187	0.745000913	-0.001103728	0.98179324	-0.016858113
9.78E-06	0.146103138	0.002388239	0.001959419	0.967684034	-0.109430004
0.782882826	0.124675932	0.009655903	0.148945001	0.001955621	0.227462561
0.970916616	0.079825359	0.098307585	0.064218314	0.183796742	0.148320055
0.402049941	-0.021550956	0.655856543	0.01254934	0.795263854	-0.021685696
0.167852275	0.043535788	0.367813536	-0.123937755	0.010097507	-0.233159317
0.254533435	0.258869069	5.17E-08	0.073502473	0.128058674	-0.032621674
0.647431649	0.121968468	0.011364485	-0.012303269	0.799191953	-0.259895898
0.550388791	0.019182076	0.691628059	0.014935769	0.757450562	-0.022987784
0.000163377	0.080153973	0.096926193	0.04996862	0.301229156	-0.071801978
0.428612813	0.051975866	0.282202766	-0.174294934	0.000281702	-0.17250692
0.785294216	0.042331469	0.381225011	-0.01583381	0.743364113	-0.028419328
0.997499123	-0.048491259	0.31576466	0.118095059	0.014272815	0.171491588
0.005073242	0.030823791	0.523820923	0.070410366	0.144945336	-0.171093146
0.849898453	-0.039112481	0.418513776	-0.164125536	0.000633922	0.064948599
0.389210923	-0.023623245	0.625192697	0.102647946	0.033338658	0.077610506
0.103999099	-0.098211572	0.041795194	-0.141133592	0.003359393	0.116117019
0.001455257	0.043246183	0.371011577	-0.067453641	0.162633935	0.188015397
0.222491184	0.076530252	0.113041248	0.01822922	0.706218096	0.083049319
0.299330128	-0.052403207	0.278259287	0.061905808	0.200123934	0.330417328
0.5365569	0.19821513	3.48E-05	0.153431911	0.001416407	-0.126883322
0.006936535	0.112841782	0.019252475	-0.031755283	0.511347844	-0.189817898
0.111504907	-0.002130149	0.964869983	0.040540316	0.401716486	0.040662232
0.074224506	0.114457311	0.017580533	0.008739145	0.856607207	-0.035158152
0.941853417	-0.152150174	0.001554505	-0.027452582	0.57022592	-0.072810163
0.529455909	-0.134994241	0.005047331	-0.150766071	0.001717426	-0.116647844
0.335158449	-0.07865615	0.103350023	-0.069301006	0.15140158	0.153092009
0.464167147	0.162107505	0.000740598	-0.190759511	6.87E-05	-0.332510752
0.139786386	-0.114418733	0.01761893	-0.075919436	0.115953856	-0.112247087
0.20697945	0.056853487	0.2394115	0.008242739	0.864671636	0.186325754
0.060520575	0.080199453	0.096736231	0.017986118	0.709958001	0.16470952
0.682039734	0.055537729	0.250477787	-0.001938478	0.968029221	0.097209097

0.043434206	0.002313702	0.961845075	-0.257656338	5.99E-08	-0.350115616
0.074080862	0.03490977	0.470283838	-0.023552058	0.626235795	-0.114674065
0.430256638	0.042626658	0.377910376	0.094210873	0.050907867	0.154546906
0.001078207	0.015150557	0.754073935	-0.178108597	0.000205375	-0.312902762
0.442519144	0.178101973	0.000205489	0.133177349	0.005676311	0.209638094
0.72806962	0.095134944	0.048667712	0.134363347	0.00525824	0.041569362
0.649363454	-0.074513397	0.122881649	-0.068009656	0.159189263	0.125236037
0.170716136	-0.01071162	0.824718186	0.10133249	0.035678881	0.275552413
0.023350776	0.035761845	0.459511545	0.047876876	0.321942393	-0.054228226
0.594787923	0.077634432	0.1079222	0.180473782	0.000168271	0.045749553
0.490715779	0.095618825	0.047527964	0.264266492	2.65E-08	0.289878909
0.916036093	-0.081040333	0.093277244	-0.049867773	0.302207014	0.025598814
0.061458675	0.240635649	4.42E-07	-0.070861354	0.142381591	-0.060606616
0.007069957	0.077416201	0.108919161	-0.185991503	0.000104681	-0.339783852
0.030077883	-0.029370789	0.543582934	-0.189940674	7.39E-05	-0.294835387
0.856581008	0.261683255	3.65E-08	0.406050874	1.69E-18	0.229789778
0.839462161	0.011710374	0.808677152	-0.226631528	2.06E-06	-0.449165554
0.104342518	0.007643784	0.874420713	0.103053315	0.032644413	-0.082241277
0.672294334	0.016322955	0.735727041	0.252996789	1.05E-07	0.190648076
0.950414955	0.035912042	0.457627069	0.009770369	0.839902844	0.06779465
0.176676931	0.038952229	0.420424372	-0.18707168	9.52E-05	-0.346659814
0.867390846	0.016570166	0.731877129	-0.055906255	0.247342995	-0.081089978
0.305397809	-0.097183448	0.043994567	-0.057597907	0.233304917	0.053936637
0.37860808	0.005522291	0.909094952	-0.068814375	0.154301612	0.015440922
0.343375192	0.07116009	0.140702556	0.097983607	0.042274686	0.092921406
0.303012165	-0.04632607	0.337883201	-0.060952887	0.20715039	-0.056762076
0.052180141	-0.061052668	0.206406393	-0.057170822	0.236794761	0.157240276
0.852831701	0.113396763	0.018663129	0.003766355	0.937928491	0.033108808
0.277428647	0.12560834	0.009122672	-0.008217425	0.865083263	-0.186636267
0.511735966	0.159343553	0.000913781	0.128262873	0.007745178	0.160054331
5.75E-09	0.041744053	0.38787371	0.188695792	8.25E-05	0.02695349
0.301547039	0.044623038	0.355960628	-0.143602605	0.002839284	0.107021965
0.880998982	0.074175375	0.124594243	0.105236745	0.029113568	0.095569432
0.004081568	0.060036812	0.214071712	0.079366992	0.100260534	-0.21238643
0.037761307	0.072212274	0.134909453	-0.160954843	0.000808755	-0.327202863
0.007850663	-0.084104252	0.081502528	0.027698252	0.566779313	0.356335038
0.758303674	-0.156647349	0.001118078	-0.13509814	0.005013337	0.007005381
0.116409826	-0.153006845	0.001460903	-0.129122692	0.007340626	-0.102604897
0.029561851	-0.096016284	0.046608583	-0.175202136	0.000261456	-0.138825793
0.103924551	0.350360018	7.29E-14	0.040564555	0.401434856	0.006121945
0.152627007	-0.011984682	0.804285179	-0.219370065	4.40E-06	-0.274699145
0.030203706	0.121384604	0.011766226	0.226456304	2.10E-06	0.2737316
0.85888111	0.049141344	0.309312967	0.210737583	1.05E-05	0.140078
0.633572982	0.11171341	0.020500873	0.097759054	0.042751527	-0.038926539
0.332888038	-0.01355614	0.779246804	-0.044613154	0.356067282	-0.031542347
0.142097834	0.190664538	6.93E-05	0.225631368	2.29E-06	0.175624421
0.679549973	0.003770598	0.937858711	0.005497158	0.909506898	0.139026516
0.418475726	-0.023117751	0.632615779	0.02753338	0.569091275	-0.089491955
0.005697028	0.034005306	0.481868892	-0.262108137	3.47E-08	-0.375238567
0.783495261	0.022022157	0.648830769	0.085601809	0.076201245	0.237004015
0.482697518	0.01689367	0.726849234	0.217859085	5.13E-06	0.146909475

0.228168116	-0.035119414	0.467620612	-0.102974756	0.032777983	-0.181951506
0.209365161	0.024293568	0.615407347	0.027897238	0.563994972	0.082789419
0.160748063	0.18187116	0.000149406	0.252957503	1.06E-07	0.159620015
0.993969274	0.096489404	0.045533678	-0.003115812	0.948633468	0.006749681
0.54073564	0.039386448	0.415259217	0.054289934	0.261296674	0.112864317
0.799613996	0.008584161	0.859123455	0.237109681	6.57E-07	0.234996493
0.069673804	0.019414594	0.688084759	-0.072363206	0.134093744	-0.106689071
0.090059739	-0.055789033	0.248337138	0.046485498	0.336221509	0.273363561
0.520980789	0.03235767	0.503364695	0.283091145	2.29E-09	0.220840695
0.795011884	0.151627409	0.001614295	0.028322933	0.558060501	0.089759256
0.60531519	-0.181746823	0.000151001	-0.246127776	2.36E-07	-0.324096758
0.014319637	0.034281007	0.478321206	-0.147458035	0.002172126	-0.194488941
0.525120393	0.067471768	0.162520756	0.179764517	0.00017868	0.217657271
0.051999393	-0.026279684	0.586816754	0.090040385	0.062115146	0.169342025
0.551684562	0.122122738	0.011260384	0.004865303	0.919871185	-0.263089777
0.737909746	0.02683212	0.578974701	-0.03490693	0.470319971	-0.093978864
0.277799429	-0.037922131	0.432827028	0.059739321	0.216354738	0.103578702
0.791901746	0.361361512	1.04E-14	0.304779779	1.08E-10	0.251319435
0.620306462	0.158526509	0.000971721	0.044855234	0.35346074	0.102509984
0.305753911	0.047935771	0.321346803	0.266720976	1.95E-08	0.232173563
0.592794544	0.014810309	0.759425022	0.002113481	0.965144696	-0.005828805
0.523201418	0.354439478	3.57E-14	-0.011901769	0.805612065	-0.059748678
0.069287345	0.011240849	0.816208682	-0.057277822	0.235916996	-0.137701958
0.572912989	-0.079045256	0.101649666	-0.182198705	0.000145281	0.003161329
0.06733748	-0.019837798	0.681653175	-0.094389321	0.050468688	0.081347285
0.041302295	0.010555739	0.827228573	-0.04011817	0.406640213	-0.248285548
0.487564378	0.071327243	0.139769723	0.079210781	0.100933096	0.081112989
0.141352578	0.095662277	0.047426719	-0.062824754	0.193513795	-0.062832157
0.023640063	0.067920876	0.159735558	0.009306641	0.847406161	0.064811749
0.226571294	0.052249035	0.279677645	0.106431017	0.027324695	0.16400834
0.514541138	0.024240369	0.616181482	0.344264503	2.08E-13	0.061485799
0.457927935	0.034284412	0.478277488	0.123801782	0.010180778	0.052894727
0.783079824	-0.014211318	0.768873149	0.006435712	0.894140642	0.032716804
0.409917882	0.016567333	0.731921202	0.18051912	0.000167625	-0.033377254
0.549199554	-0.032631304	0.499760145	-0.009993184	0.836302713	0.051964184
0.979986083	0.049316516	0.307589443	0.140111925	0.00359883	0.004842317
0.086511588	0.132736203	0.005839287	0.09602808	0.046581527	0.061237652
0.119927444	0.033278768	0.491285828	-0.050250968	0.29850255	0.052611811
0.29456435	0.154879661	0.001274033	0.04994415	0.301466235	-0.026630037
0.638509509	-0.028305623	0.558301219	0.091303948	0.058522861	0.05048205
0.039782734	0.016180109	0.737954654	0.266641801	1.96E-08	0.4171243
0.716075998	0.083883076	0.082310167	0.171109341	0.000364981	0.257409
0.155903568	-0.044849733	0.353519829	0.291563039	7.15E-10	0.132932705
0.825237092	-0.02366438	0.624590276	0.039432605	0.414712378	0.116620107
0.305830313	0.010884487	0.82193634	0.30921993	5.57E-11	0.231427801
0.019864415	0.115575009	0.016499366	-0.173985623	0.000288933	-0.160341119
0.720243183	0.133226144	0.005658539	-0.000673789	0.98888478	-0.00923476
0.807878106	-0.045856319	0.342809829	0.261328957	3.82E-08	0.429455364
0.698260637	0.098354804	0.041496279	-0.114448992	0.017588807	-0.228058874
0.428488224	-0.043634219	0.366730493	-0.030209544	0.532130191	0.042009624
0.002080931	0.124432879	0.009799374	0.122964812	0.010706891	0.00529814

0.899091818	-0.046379005	0.337330887	0.07114534	0.140785101	0.129842983
0.145976268	0.033779086	0.484790486	-0.289175234	9.96E-10	-0.180183405
0.3266067	0.019478279	0.687115472	-0.369515758	2.33E-15	-0.455205534
0.116525384	0.032646202	0.499564282	-0.132577868	0.005898799	-0.167084749
0.316587054	0.154007408	0.001358147	0.028987008	0.548863726	-0.007147276
0.60025304	-0.11295815	0.019127576	-0.177215267	0.000221283	-0.113069046
0.55760163	0.061073879	0.206248488	0.193548579	5.34E-05	0.135857851
0.168398828	0.283797463	2.08E-09	0.030392349	0.529650345	0.107947977
0.983549931	0.098887011	0.040401358	0.035328688	0.464970408	0.228868436
0.658467714	0.123735502	0.010221589	0.07343794	0.128394805	0.012601063
0.32378473	-0.027875781	0.564294889	-0.076528138	0.11305123	-0.012499687
0.636629561	0.185329298	0.000110898	0.092225078	0.056013917	0.082950441
0.811175513	-0.001789089	0.970491886	0.128034258	0.007856041	0.09973381
0.128956813	0.059265082	0.220030194	0.023199194	0.631417279	-0.13288383
0.549203133	0.001271153	0.979032038	-0.052414336	0.278157086	-0.033658934
0.084100117	-0.017524346	0.71708107	0.028161813	0.560303053	0.051385647
2.07E-05	0.120387289	0.012481541	-0.137791469	0.004201089	0.013887361
0.583308763	-0.000950777	0.984315928	0.007993216	0.868730664	-0.119316859
0.658095539	0.033217137	0.492089159	0.035357162	0.464610466	0.195079256
0.343259332	0.068525484	0.156043014	-0.043408248	0.369219814	-0.294833158
0.574488968	0.084105325	0.081498626	0.169999247	0.000399031	0.150080473
0.93612637	-0.04200702	0.384888638	-0.1544569	0.001314183	0.018717121
0.29920685	-0.020317773	0.674386638	0.144283264	0.002709395	0.313920809
0.057277923	0.031122295	0.519806964	0.002520302	0.958441003	0.022077473
0.289865463	0.006075262	0.900037671	-0.080124657	0.097048794	-0.141301634
0.12217904	-0.013087376	0.786693135	0.102127631	0.034248179	0.245326006
0.2315035	0.059199736	0.22054012	0.260146026	4.42E-08	0.120349767
0.164339766	0.002628796	0.956653695	-0.255660009	7.63E-08	-0.109882767
0.279099431	0.012253132	0.799992924	0.079169125	0.10111305	0.085497914
0.171257349	0.041293724	0.393018153	-0.001297938	0.978590311	0.075087534
0.270096118	0.015755491	0.744589291	-0.047917806	0.321528402	0.174926094
0.256812842	-0.032077984	0.507063066	-0.079282247	0.100624959	-0.094163501
0.000187051	0.074874863	0.121070608	0.269019124	1.45E-08	0.209995732
0.072128107	-0.040880575	0.397773922	-0.115900685	0.016195486	-0.276159306
0.618035503	-0.108648062	0.024253508	-0.002451741	0.959570571	0.085724363
0.413912434	0.289268461	9.83E-10	0.012130072	0.801959797	-0.112924704
0.299465278	0.071976397	0.136191902	-0.072906532	0.131188775	-0.219137366
0.562852745	-0.021378742	0.658431962	0.123045168	0.010655355	0.128521366
0.779445763	0.11830621	0.014098846	0.193947916	5.15E-05	0.067313621
0.47483167	0.022425243	0.642845188	0.004689422	0.922758633	0.014423453
0.788852415	0.003932845	0.935190544	0.114513503	0.017524736	0.009832488
0.071890243	0.076748622	0.11201403	0.011619861	0.810127708	-0.013706709
0.619436741	0.171754129	0.000346467	0.020731032	0.668154263	-0.148800438
0.022709371	0.062460294	0.196116038	0.05372505	0.266298807	0.141282716
0.308639804	0.058141483	0.228915972	-0.057471973	0.234330162	0.031440983
0.57723745	0.068059033	0.158886036	0.210448569	1.08E-05	0.311293255
0.021921335	0.050599197	0.295162422	0.141627903	0.003248817	0.395387825
0.176615812	-0.035309214	0.465216674	-0.129359923	0.007232384	-0.046764117
0.211495358	-0.051628415	0.285436727	-0.091387144	0.058292498	-0.167339882
0.261783045	-0.033569948	0.48749995	-0.034370491	0.47717281	0.060679718
0.003291128	0.092577885	0.055076897	-0.113930655	0.018111082	-0.265384292

0.827724467	0.018578143	0.700862585	0.100439328	0.037346225	0.116512013
0.678834868	-0.008335561	0.863162599	-0.044496994	0.357322296	-0.035661235
0.295152622	0.058695722	0.22450156	0.084733359	0.079240276	0.00903358
0.632295375	0.027386501	0.571154702	-0.14989149	0.001828289	-0.101016593
0.414090441	-0.019794249	0.682313952	0.186953774	9.62E-05	0.181765451
0.518011403	0.005750717	0.90535199	0.022096296	0.647728127	-0.051860419
0.908093939	0.016960862	0.725806394	0.008661761	0.857863401	0.069506616
0.491640072	0.069865281	0.148090851	0.021509737	0.656472599	-0.142559373
0.100877117	-0.073706677	0.126999534	0.078588534	0.103647783	0.225998767
0.385741222	-0.183321874	0.000131932	-0.227662553	1.84E-06	0.0234201
0.703351751	0.03666359	0.448262837	0.006956311	0.885633929	-0.040713752
0.733768302	-0.028616562	0.553984827	0.096537296	0.045426041	0.060673734
0.008660778	0.059384532	0.219100257	-0.033184698	0.492512273	-0.181492676
0.45253562	0.070638306	0.143645185	0.06277318	0.193880489	-0.092092917
0.002989889	-0.019208345	0.691227405	-0.004252269	0.929939735	0.14632187
0.490532913	0.011174738	0.817270531	0.001741231	0.971280873	0.132493137
0.926366522	-0.01015216	0.833736134	0.122423465	0.011059877	0.047367302
0.308449693	-0.005245502	0.91363305	0.022155464	0.646848703	0.052822796
0.545502584	0.066151773	0.170918156	0.363044566	7.66E-15	0.282356694
0.425444796	-0.044969638	0.35223311	0.022818094	0.637033723	-0.066905532
0.046044773	-0.088287468	0.067397849	0.023835378	0.622088742	0.01442778
0.050306389	-0.049438418	0.306393789	0.008940816	0.853335146	0.196340091
0.125226676	-0.026878726	0.578315364	-0.218195252	4.96E-06	-0.279770899
0.038327013	-0.000362433	0.994020949	0.006046651	0.900505989	0.126263638
8.86E-05	0.006508408	0.892952027	0.013230548	0.784416754	-0.072215311
0.393168793	0.003194602	0.947336407	0.005804421	0.90447231	0.088003842
0.601992014	0.023608491	0.625408818	0.126597598	0.008585721	0.383502637
0.693030815	0.141193063	0.003345911	0.39745803	1.00E-17	0.217438759
0.240211452	0.055187871	0.253479252	0.194075779	5.09E-05	0.086510736
0.877245491	-0.025803733	0.593612146	-0.005207542	0.914255648	0.070679086
0.184639927	-0.058754026	0.224040735	-0.07670668	0.112210752	-0.112704203
0.189599854	-0.070691795	0.143341388	-0.277985035	4.53E-09	-0.200347799
0.028053851	0.141584254	0.003258446	-0.026627111	0.581879165	-0.003012265
0.019101015	0.044564616	0.356591358	-0.116535804	0.015616948	-0.172573476
0.550412575	0.000970429	0.983991784	-0.173658581	0.000296767	-0.232764803
0.395947384	0.010974477	0.820489064	0.003063015	0.949502691	-0.018851492
0.351007738	0.029145587	0.546678633	-0.214641723	7.11E-06	-0.301105679
0.446047613	-0.037174252	0.44196241	0.313733245	2.82E-11	0.181573936
0.769702402	0.010429945	0.829255717	0.197441274	3.74E-05	0.24929178
0.680114463	0.081946824	0.089659884	0.055563578	0.250257005	0.139838676
0.71427273	0.50268062	6.47E-29	0.122728872	0.01085949	0.143830115
0.004455999	0.044780632	0.354262719	-0.132163363	0.006057183	-0.192042074
0.342943587	0.007134888	0.882718915	-0.065459416	0.17545017	-0.116479657
0.467201358	0.036471779	0.450642425	0.029989131	0.535127978	0.300453838
0.49724011	-0.009377518	0.846258424	0.215715896	6.38E-06	0.114661816
0.901061055	0.021662534	0.654190106	0.0919375	0.056787446	0.235452097
0.833922717	0.140892865	0.00341447	0.152026032	0.001568516	0.108362408
0.518512379	-0.005017012	0.917381401	0.029453794	0.542444125	-0.011597651
0.270660987	-0.040140367	0.406380424	-0.180690509	0.000165206	-0.029452372
0.543656687	0.106141949	0.027748782	0.070419699	0.144891923	-0.089607488
0.422089732	-0.06228632	0.197367182	-0.020688176	0.668799528	-0.021987512

0.375992277	-0.019055402	0.693561255	-0.10584625	0.028188436	-0.251287558
0.027280932	0.02573151	0.594646439	0.111457289	0.020793843	0.379389984
0.570363032	-0.048950671	0.311196212	-0.141878166	0.003194103	-0.152626939
0.45031979	0.091214532	0.058771288	0.038769073	0.422614296	0.061908077
0.295438702	-0.091916085	0.056845401	-0.089373644	0.064082779	-0.026286415
0.59899849	0.036516977	0.450081064	0.162314422	0.000728941	-0.003167994
0.973829685	0.340486944	3.93E-13	-0.02627601	0.586869078	-0.129191393
0.050687028	-0.009707047	0.840926578	-0.105440113	0.028802034	-0.029029196
0.068215166	0.0481595	0.31909085	-0.228723485	1.64E-06	-0.213628092
0.416175215	-0.094761927	0.049561859	-0.088462839	0.06685328	-0.122872298
0.827490558	0.243121681	3.33E-07	0.202977943	2.23E-05	0.033723051
0.077793137	0.123571483	0.010323206	0.012394517	0.797734742	-0.064489681
0.07508683	-0.008966038	0.852926089	0.215285706	6.66E-06	0.357798008
9.81E-05	0.118679549	0.013795811	0.211503909	9.72E-06	-0.066361823
0.373530973	0.025535307	0.597460347	0.00411117	0.932258818	-0.038662778
0.368966242	-0.043559239	0.367555327	-0.193638888	5.30E-05	-0.156459625
0.896196129	0.002245438	0.962969977	-0.032178235	0.505735777	-0.000726517
0.715288922	0.059541834	0.217879919	-0.203986801	2.02E-05	-0.357267683
0.388214273	-0.001084013	0.982118402	0.251558913	1.25E-07	0.264482508
0.675042033	0.05194016	0.28253396	0.044441565	0.35792213	0.176708984
0.765052433	0.142626939	0.003035362	0.200951859	2.70E-05	0.221803269
0.09353793	0.024736304	0.608981331	0.135334189	0.004936873	0.276358001
0.645415066	0.005260531	0.913386581	0.163488729	0.000665939	0.174388288
0.316306661	0.037180823	0.441881662	-0.093958357	0.051534782	-0.118251115
0.562583593	0.331790668	1.65E-12	0.102844307	0.033000809	0.127966302
0.875315105	-0.110349036	0.02210384	-0.111988269	0.020190467	-0.024151159
0.089365078	0.023225298	0.631033354	0.138132407	0.00410724	0.015514888
0.033279532	-0.043917038	0.363629655	0.077530643	0.108395448	0.258666057
0.657343838	-0.024784721	0.608280402	0.010866797	0.82222091	-0.035959716
0.960772169	0.064870404	0.179375536	-0.118591064	0.01386711	-0.195941686
0.645186937	-0.005646632	0.907057263	0.356400677	2.52E-14	0.099404605
0.300679761	0.226713786	2.04E-06	-0.015049861	0.755656361	-0.069089117
0.076047649	0.074006735	0.125455567	-0.237261208	6.46E-07	-0.367517915
0.20289665	0.180019962	0.000174863	0.09661242	0.045257627	0.171375946
0.736656651	0.012075265	0.802836174	0.128421733	0.007668967	0.199830557
0.602128821	0.034046288	0.481340645	0.251251088	1.29E-07	0.203472212
0.784548677	-0.05476832	0.257111427	-0.084778175	0.079081079	0.033753633
0.764372935	0.118351521	0.014061758	0.082046642	0.089268538	0.259176816
0.04230515	-0.044263016	0.359858637	-0.072087444	0.135586985	-0.03261452
0.000153286	-0.005847162	0.903772288	-0.069024346	0.153045191	-0.401174739
0.034129824	0.065236361	0.1769291	-0.122928717	0.010730113	-0.18180773
0.754742879	0.054291852	0.261279798	-0.013835184	0.77482369	-0.033326947
0.227211144	-0.053684943	0.266656443	0.17314398	0.000309496	0.206375074
0.50074028	0.012338506	0.798629141	-0.053084542	0.27204958	0.01888178
0.7956488	0.117996787	0.014354423	-0.036003951	0.456476057	-0.159444971
0.123765294	0.02020658	0.676067368	0.016589213	0.731580776	0.031924449
0.242151447	0.125462906	0.009204081	0.291841633	6.87E-10	0.360244847
0.895562631	-0.015608443	0.746891338	0.108058673	0.025039374	0.216566544
0.877889082	-0.010765908	0.823844326	-0.092907708	0.054212735	-0.096450765
0.683259848	-0.002031343	0.966498495	0.010474871	0.828531606	0.046931883
0.985382583	0.096999641	0.044397855	0.049869058	0.302194541	-0.00355462

0.746072951	-0.095986561	0.046676817	-0.017623838	0.71554426	0.068941203
0.188554456	-0.027379731	0.57124989	-0.020242783	0.675519962	-0.072536324
0.979977003	-0.003119535	0.948572183	-0.031387966	0.516247851	-0.015865706
0.951823895	0.046789926	0.333063099	0.264004496	2.74E-08	0.119956686
0.54827241	0.141639821	0.003246192	0.181628053	0.000152539	-0.016871411
0.943831458	0.033107055	0.493525774	0.037462551	0.438427866	0.05060234
0.129667376	0.064908268	0.179121252	0.243806258	3.08E-07	0.329836757
0.984530071	-0.003842983	0.936668247	0.122843645	0.010785019	0.304879938
0.875379574	0.139274508	0.003806508	0.120266071	0.012571048	0.080698885
0.225634	-0.03436709	0.47721642	-0.240551498	4.47E-07	-0.08107361
0.005011488	0.028052865	0.561821919	-0.094967643	0.04906706	-0.218211637
0.524654991	0.013156332	0.785596535	0.026081318	0.589644556	-0.008317182
0.21001459	0.071673566	0.137852091	0.09545187	0.047918661	0.131129932
0.709915665	0.027375285	0.57131242	-0.012359355	0.798296186	-0.036434551
0.278482447	0.007472838	0.877206723	-0.002195288	0.963796452	0.029055039
0.457342553	0.166752308	0.000516357	0.360816096	1.15E-14	0.229572478
0.661263729	0.03192881	0.509041432	-0.250353797	1.44E-07	-0.281100316
0.668005785	-0.000217174	0.996417266	0.175258076	0.000260253	0.239927414
0.929829022	-0.048101112	0.319678609	0.264880773	2.45E-08	0.365664033
0.35539168	-0.080963523	0.093589043	-0.058996926	0.222128117	-0.003012172
0.460215214	0.011990976	0.804184473	0.203730941	2.07E-05	0.229565446
0.058385058	0.094025904	0.051366458	0.065796769	0.173230897	0.178937599
0.720602617	0.016992041	0.725322666	0.013036655	0.787500018	-0.05397786
0.392405735	0.166803132	0.000514296	-0.066287763	0.170038349	-0.135004181
0.372341639	0.117740027	0.014569584	0.101345317	0.035655404	0.271341641
0.490378477	0.153045653	0.001456788	0.105817984	0.028230774	0.280220856
0.221482153	0.04398864	0.362847207	-0.15901431	0.000936734	-0.218948387
0.336812368	0.076648577	0.112483727	0.251354268	1.28E-07	0.316611548
0.008044537	0.024796233	0.608113796	-0.169375538	0.000419438	-0.266124963
0.502820487	-0.086926475	0.071748822	0.101972015	0.03452427	-0.04316677
0.066179181	0.129061018	0.007369002	-0.061469487	0.203319362	-0.005497573
0.643232283	-0.004373578	0.927946404	-0.007819072	0.871565554	0.00383295
0.846006441	0.024804215	0.607998286	-0.049243186	0.308310166	0.014215131
0.970589658	-0.024606579	0.610861121	0.036005315	0.456458982	0.032728559
0.004175453	0.15807558	0.001005133	-0.144248798	0.002715839	-0.300166016
0.620119802	-0.013930636	0.773312346	-0.030794415	0.524216787	-0.084055776
0.709820365	0.011921878	0.8052902	-0.023020858	0.634042892	-0.156863599
0.031347655	0.041303221	0.392909247	0.140394812	0.00353103	0.277186804
0.290294018	0.012494325	0.796141631	-0.089394514	0.064020421	-0.016723721
1.33E-07	0.066844798	0.166469842	0.065824518	0.173049281	-0.017787912
0.228335686	0.061914736	0.200058931	-0.089869306	0.062615207	-0.040795897
0.692784186	0.070853207	0.142427597	-0.001980869	0.967330463	0.057218856
0.10811732	0.155173403	0.001246803	0.022366461	0.643716636	-0.033284936
0.245119558	0.003406243	0.943853045	0.091103825	0.059080073	0.074704548
0.471715736	0.124212018	0.009931383	0.062742206	0.194100959	0.122320653
0.070008478	0.020585827	0.670341529	0.01364668	0.777810865	-0.053659926
0.965587357	0.072614192	0.132745708	0.125319448	0.009285014	0.253844884
0.120578923	0.018774626	0.697853309	-0.010880508	0.822000345	0.14060656
0.827013238	0.038297208	0.428286757	0.140395824	0.00353079	0.088024451
0.778070354	0.02238742	0.643405859	0.122312144	0.011133727	0.058895845
0.779843973	0.038908262	0.420949459	-0.006311315	0.896175169	0.098215955

0.581333961	0.153349993	0.001424884	0.170717882	0.000376667	0.35313145
0.763961348	0.05384079	0.265268604	-0.012973131	0.788510897	0.06264287
0.97901828	0.079611324	0.099215714	0.189357762	7.78E-05	0.070802284
0.055621557	0.051631069	0.285411935	-0.022801347	0.637281023	0.046603033
0.036453375	0.11274377	0.019358224	-0.095415945	0.048003081	-0.232855603
0.404864563	0.094311768	0.050659163	0.14366855	0.002826457	0.096991397
0.954474309	-0.146155109	0.002379601	-0.166812754	0.000513907	-0.122965014
0.665420559	0.108221132	0.024820594	0.007886595	0.870466144	-0.113288277
0.133850275	0.157815496	0.001024883	0.129745979	0.007059285	0.209181291
0.600753478	-0.00311856	0.948588226	0.145836313	0.002433044	0.060924686
0.964809099	0.096397291	0.045741306	0.144351707	0.002696638	0.050521052
0.590372361	0.082503431	0.087495095	0.315385979	2.19E-11	0.520076582
0.050716476	-0.088901568	0.065506719	0.080383083	0.095972271	0.211618836
0.101266527	0.035696086	0.460337971	0.146490174	0.002324586	0.147406918
0.75111062	0.105670143	0.028453108	0.019343274	0.689170868	-0.027010335
0.370321889	0.121318668	0.011812374	0.190478184	7.04E-05	0.053238618
0.234369782	-0.029992191	0.535086298	-0.064780381	0.179981165	-0.300433229
0.674173051	-0.050719629	0.294013095	0.145298427	0.002525704	0.151942022
0.005375109	0.014695739	0.76122946	-0.041147137	0.394701578	0.02914259
0.140438765	0.033806882	0.484430996	0.045807293	0.343326629	0.102834878
0.714391219	0.074618714	0.122351822	0.115961076	0.01613968	0.118387925
0.828850472	0.06547832	0.17532525	0.04768826	0.32385462	0.090153067
0.539759897	0.162800521	0.000702221	0.089440487	0.063883234	0.202400578
0.991305248	0.005256477	0.913453063	-0.012442748	0.796964779	0.002779856
0.266423197	0.076700512	0.112239705	-0.092320435	0.055759362	-0.138939577
0.473346	-0.024240755	0.616175868	-0.109318046	0.02338614	-0.233774917
0.4156378	0.010281643	0.831647016	-0.06055346	0.210148068	0.000222732
0.562559584	-0.034384486	0.476993332	-0.060201757	0.212813374	-0.248473913
0.771940094	-0.039564111	0.413156691	-0.092865965	0.054321477	0.049451759
0.367604915	0.074467867	0.123111255	0.149407389	0.001892435	0.225625535
0.674527691	-0.016810527	0.728140325	0.060077635	0.213759784	0.174282237
0.086582463	0.07342283	0.128473609	0.030529766	0.527790078	0.093876438
0.856377528	0.136915872	0.004451058	0.050265166	0.298365871	0.07617317
0.01171349	0.180404054	0.000169269	-0.038167395	0.429855001	-0.270337629
0.252809059	0.081011207	0.093395382	0.145270675	0.00253057	0.077439808
0.731463609	-0.031921197	0.509142512	-0.236552034	6.99E-07	-0.218378817
0.321526233	-0.046026849	0.34101609	0.023452029	0.627702777	0.055164926
0.6019397	0.139270271	0.003807586	0.061109651	0.205982375	0.056949915
0.281436278	0.00916848	0.849644369	-0.194228362	5.02E-05	-0.404721568
0.663396491	0.019868986	0.681180107	-0.06035888	0.211619659	-0.008200257
0.897025906	0.157220378	0.001071427	0.032099896	0.506772806	0.10868031
0.950740941	-0.027744514	0.566131399	-0.057761933	0.231974321	-0.143798364
0.703434351	-0.023741453	0.623462238	-0.064545689	0.181567172	0.020064202
0.751822475	0.110454258	0.021976463	0.084223731	0.081068913	-0.025399106
0.027451755	0.046153699	0.339685699	-0.22835492	1.71E-06	-0.331731156
0.565314637	0.080105026	0.097130965	0.001956341	0.96773476	-0.091753696
0.09793501	0.137208568	0.004366042	-0.072520337	0.13324857	-0.104690854
0.379455426	0.109608412	0.023018644	-0.018433792	0.703076398	-0.052360424
0.345142554	0.0163627	0.735107618	-0.041423101	0.39153599	-0.021482196
0.939150712	0.031392425	0.516188221	-0.30169616	1.69E-10	-0.22986559
0.035253297	0.065803771	0.173185055	0.157805823	0.001025624	0.161358573

0.084115257	0.030289291	0.53104766	0.14180278	0.003210495	0.143841331
0.843736136	-0.099328027	0.039512634	0.012241611	0.80017702	0.163100337
0.714430759	0.082367501	0.088019844	0.363021853	7.69E-15	0.209790126
0.18156093	0.08174482	0.090456079	0.258224995	5.59E-08	0.236830467
0.091253195	0.112064996	0.02010455	0.001587036	0.973823144	0.204906666
0.433665652	0.03841524	0.426863732	-0.04997004	0.301215405	-0.038305837
0.009418699	0.048258638	0.318094521	0.022201571	0.646163756	0.006297764
0.281944364	0.09464797	0.049837745	-0.115141237	0.016911848	-0.306344554
0.831533933	0.005533994	0.908903134	0.222974682	3.02E-06	0.310281901
0.603675993	0.013965187	0.772765493	-0.222092328	3.32E-06	-0.100788125
0.06531675	0.011671614	0.809298239	0.082648596	0.086937474	0.15811429
0.187693402	0.010821245	0.822953793	0.009608146	0.842526072	-0.000676028
0.929423408	0.031338736	0.516906413	-0.014399208	0.765905723	0.187670732
0.744524505	-0.087659608	0.069377405	0.056704744	0.240645001	0.143383531
0.133699556	-0.001238125	0.979576712	-0.102991233	0.03274993	-0.119842302
0.528212927	-0.092325772	0.055745143	-0.068245323	0.157745947	0.035623684
0.833888422	0.078540769	0.10385854	-0.021724874	0.653259793	-0.226822159
0.098643812	0.120494864	0.01240258	0.136298943	0.004635165	0.032315955
0.52002679	-0.000996358	0.983564122	-0.061815222	0.200784322	-0.063563527
0.666373252	0.09455858	0.050055052	0.203919515	2.03E-05	0.328169998
0.910520192	0.031911314	0.509273733	0.02230087	0.644689598	0.063148387
0.001852558	-0.08958691	0.063447902	-0.157525965	0.001047289	0.067245621
0.883436285	0.158143394	0.001000042	-0.006052957	0.900402766	0.096279976
0.468824369	0.032809777	0.497416517	0.063260003	0.19043933	0.209012433
0.747379308	-0.015249756	0.752516035	0.224246877	2.65E-06	0.15471118
0.441065267	-0.001872187	0.969121974	-0.045004177	0.351863011	0.149873694
0.404008891	-0.14559608	0.002474038	-0.07228485	0.13451674	-0.099338123
0.296997732	-0.033871297	0.483598454	-0.068707593	0.15494356	0.141622831
0.903757344	-0.153356506	0.001424209	-0.14509887	0.002560891	-0.286618182
0.763303415	-0.033146223	0.493014365	-0.02236124	0.64379406	0.050078976
0.756618441	-0.023503666	0.62694531	0.106043208	0.027894933	0.136973044
0.02904178	0.057902285	0.230840037	0.025668966	0.595542778	0.09370952
0.922900161	-0.001613616	0.97338489	0.052484062	0.277517381	0.079435658
0.772896913	0.058678759	0.224635764	0.049016321	0.310546954	0.003749551
0.962277181	-0.185174453	0.000112401	-0.186131016	0.000103414	-0.117928709
0.507290981	0.086369743	0.073593513	0.102889195	0.032923989	-0.021771465
0.76443944	0.007161046	0.882292049	0.199677104	3.04E-05	0.210145636
0.437301715	0.227463796	1.88E-06	0.143694099	0.002821501	-0.163623011
0.246535235	0.079643932	0.099076933	-0.359743936	1.39E-14	-0.261647241
0.159356876	0.032059072	0.507313652	0.115133135	0.016919638	-0.015751359
0.906750805	0.017109352	0.723503586	0.02113947	0.662016912	0.182299939
0.053837342	-0.020028505	0.678762415	0.014573713	0.763152757	0.032856582
0.003467475	-0.011108401	0.818336338	0.183069188	0.000134831	0.396363016
0.578585085	-0.106623601	0.027045261	-0.182703337	0.000139134	-0.161167094
0.085563369	-0.076862598	0.111480801	-0.029253047	0.545200367	0.156006972
0.343845327	0.016128525	0.738759625	-0.113558512	0.018494351	-0.214084238
0.702937145	0.129281083	0.007268197	0.1164747	0.01567181	0.152567423
0.300057466	-0.09800044	0.042239122	-0.21903381	4.55E-06	-0.229929884
0.042492114	0.037751609	0.434900329	-0.149278525	0.001909854	-0.094338927
0.076109177	0.070209016	0.146101268	0.098823766	0.04053018	0.042798788
0.080669119	0.068443696	0.156538714	-0.128568157	0.007599315	-0.227691189

0.666306293	0.036208873	0.45391557	-0.048879652	0.311899585	-0.035265123
0.003882159	0.135753386	0.004803667	0.104349815	0.03050624	0.087724204
0.016533006	-0.019456774	0.687442725	-0.036346453	0.452201049	0.08147476
0.579298847	0.032325613	0.503787872	-0.031400236	0.516083776	-0.054571658
0.062382952	-0.044662899	0.355530685	-0.129605989	0.00712162	0.042535132
0.689483733	-0.031182437	0.519000148	0.029729585	0.538668841	0.037433859
0.619343853	0.114556001	0.01748264	0.043470611	0.368531778	-0.017453961
0.767774963	-0.004591206	0.924371499	0.215992004	6.20E-06	0.159500239
0.310693625	0.006953741	0.885675892	0.022126996	0.64727177	0.288027327
0.127717405	0.108760124	0.024106525	0.214726616	7.05E-06	0.259155094
0.827341182	0.086215944	0.074109874	-0.03266968	0.499255713	-0.16032079
0.094458898	0.072139445	0.135304425	-0.071761819	0.13736667	-0.170856966
0.279774968	0.034577089	0.474527159	0.169221799	0.000424615	0.208442494
0.080749866	0.091314699	0.05849305	0.045962411	0.34169318	0.143165267
0.342042535	-0.046429805	0.33680139	-0.291039652	7.69E-10	-0.181856489
0.08695586	0.002982297	0.950831719	-0.128940558	0.007424709	-0.061653199
0.174657519	-0.086187847	0.074204524	-0.162840851	0.000700045	-0.020979831
0.801900005	0.046253619	0.338640081	-0.212467236	8.84E-06	-0.266625006
0.315803493	-0.006241449	0.897318113	-0.088447404	0.066901065	-0.032317815
0.566894673	0.011111738	0.81828272	-0.1015453	0.035291078	-0.078845578
0.583457193	0.020724476	0.668252955	0.303900321	1.22E-10	0.405805512
0.055363601	0.087045431	0.071359605	-0.120630643	0.012303546	-0.181665779
0.034152	-0.054962796	0.255423336	-0.157159015	0.001076335	-0.108151605
0.246872442	-0.087741213	0.069117456	-0.210496644	1.07E-05	-0.255908718
0.575824972	0.077154444	0.110124526	0.088747483	0.065977081	-0.112758506
0.025011415	0.035937251	0.457311208	-0.125027268	0.009451812	-0.205838852
0.011051843	0.117691775	0.014610333	-0.049878907	0.302098958	0.006985194
0.000150314	-0.006122086	0.899271297	-0.086501714	0.073152779	-0.282314741
0.70447736	0.004567583	0.924759479	-0.043023086	0.373486883	0.033180829
0.054120601	0.063809776	0.186607332	-0.196219443	4.19E-05	-0.381488756
0.864397377	-0.00249176	0.958911234	-0.002417779	0.960130153	0.046129126
0.645877504	-0.04125159	0.393501586	-0.250577162	1.40E-07	-0.361681323
0.5398562	0.026535051	0.583185618	0.184378756	0.000120431	0.174813818
0.98462505	0.109519175	0.02313105	-0.010328144	0.830897041	-0.078204917
0.753500038	0.153618744	0.001397246	-0.018836372	0.696908608	0.062440832
0.626367057	0.08160307	0.091018161	0.0281835	0.560000949	-0.321545426
0.796083531	0.210923372	1.03E-05	0.333558696	1.24E-12	0.376483189
0.993405129	0.029728303	0.538686367	-0.239808229	4.86E-07	-0.284962915
0.039741195	-0.00302768	0.950084477	-0.041768637	0.387594055	0.003985142
0.989930975	0.316907947	1.74E-11	0.024000787	0.619673109	-0.090894985
0.622652647	0.014724049	0.760783456	-0.073081024	0.130266222	-0.233627583
0.02878945	0.004762604	0.921557075	0.090814511	0.059893373	0.13558744
0.057244595	0.036038715	0.45604111	-0.163342833	0.000673482	-0.287231904
0.24974144	0.018130437	0.707736925	-0.157805431	0.001025654	-0.146666803
0.034304882	-0.064482529	0.181995752	-0.167910906	0.000471235	-0.262308585
0.464764696	0.080035084	0.097424167	0.371301784	1.67E-15	0.21142831
0.060727342	0.140366668	0.003537723	0.013664309	0.777531358	-0.02635246
0.37787735	0.120624272	0.012308178	-0.029186515	0.546115383	-0.064287567
0.760895533	0.074821629	0.121336009	0.159217063	0.000922537	0.18698243
0.126107886	0.071530146	0.138643759	-0.03327503	0.491334533	-0.135349263
0.41812831	0.033258894	0.491544803	0.234001731	9.27E-07	0.082937861

0.011930661	-0.019393712	0.688402699	-0.010654862	0.825632043	-0.003335365
0.997995139	0.055658397	0.249448318	0.156972642	0.001091371	0.128269827
0.709953286	0.087149178	0.07102156	0.136469932	0.004583461	0.107225377
0.019820709	-0.10909416	0.023672953	-0.157109962	0.001080274	0.037656505
0.232130582	0.009751814	0.840202801	-0.021252296	0.660325489	0.006894785
0.001810151	0.02920277	0.545891761	-0.043439553	0.368874338	0.021741495
0.379975249	0.137632416	0.004245531	-0.035682093	0.460513927	0.103974371
0.074041373	0.012144313	0.801732122	0.037128192	0.442528602	0.105500539
0.3184305	0.039435585	0.414677083	0.094509084	0.050175715	-0.075159948
0.27850909	0.00999961	0.836198935	-0.039318294	0.416067445	-0.016605685
0.563110212	-0.033620159	0.486848709	-0.036707493	0.447719185	-0.071425914
0.843559675	0.027399755	0.570968352	0.017931254	0.710802999	0.168678507
0.562273797	0.036060552	0.455768018	0.439820789	9.09E-22	0.345800612
0.359002231	-0.003179302	0.947588282	-0.244677396	2.79E-07	-0.319284108
0.26821556	0.094210134	0.050909693	0.088960622	0.065327183	0.093232017
0.54983327	0.132839757	0.005800657	-0.021781388	0.652416883	-0.272398411
0.034550255	0.009741136	0.840375428	0.083723284	0.082897677	0.215800465
0.232320966	-0.05309846	0.271923729	0.129927878	0.006979018	0.196875393
0.136245867	0.048610209	0.314577613	0.1543814	0.001321475	0.070394333
0.46112849	-0.025033044	0.604691073	-0.142597758	0.003041412	-0.094314975
0.048998283	0.160673644	0.00082624	0.046056632	0.340703427	0.22607842
0.008400361	0.002736289	0.95488308	-0.034351945	0.477410699	-0.051931746
0.12884323	0.037974999	0.432185392	0.013037212	0.787491165	-0.03093521
0.774104346	0.021092373	0.662723467	0.064638491	0.180938797	0.000760437
0.881721708	-0.020134942	0.677151072	0.051730529	0.284483706	0.00558367
0.430781038	0.024325017	0.614949906	0.02908553	0.54750567	-0.070100791
0.551515641	0.041951715	0.385515265	0.296836045	3.40E-10	0.151235674
0.664896747	-0.062557881	0.195416774	-0.065542663	0.174900578	-0.158544878
0.079471633	0.012924856	0.78927935	-0.223260961	2.94E-06	-0.315162628
0.625133518	-0.008043929	0.867905439	0.09465328	0.04982486	0.105334657
9.98E-05	0.030951448	0.522102384	0.071595889	0.13828043	-0.07371073
0.934827981	-0.095906522	0.046860976	-0.046826362	0.332686359	-0.041664626
0.588143394	-0.139665044	0.003708342	0.144784769	0.00261718	0.026882996
0.213203918	-0.008332621	0.863210393	0.161079603	0.000801107	0.314083532
0.098082151	0.005836404	0.903948472	0.018975752	0.694777822	-0.060493372
0.058124966	-0.225175359	2.40E-06	-0.137754443	0.004211397	0.024071003
0.002303002	-0.061250547	0.204936666	-0.032306536	0.50403979	-0.098308339
0.995305779	0.015474248	0.748994144	-0.120298891	0.012546758	0.017049709
0.085717233	0.061534852	0.202838317	0.087530919	0.069788961	0.164908016
0.882940723	0.04730189	0.327794706	0.132524195	0.005919096	0.113091801
0.016851708	-0.048407786	0.316599407	0.120397787	0.012473816	0.243831482
0.149535528	0.039483606	0.41410863	0.003201745	0.947218829	-0.229295754
0.541547494	0.01632165	0.735747377	0.077340361	0.109267322	0.190633603
0.256694277	-0.088017534	0.068243165	-0.057616454	0.233154196	0.048883274
0.092077426	0.048893115	0.311766165	-0.020922131	0.665279948	-0.103123079
0.637470873	-0.005812536	0.904339389	0.135517023	0.004878371	0.155311219
0.016322059	0.044120833	0.361405402	0.032282757	0.5043539	0.188295118
0.206344396	-0.038139776	0.430189088	0.052944528	0.273317898	0.15602223
0.002834987	0.106148012	0.027739829	-0.140948649	0.003401634	-0.296677905
0.885509667	-0.177675812	0.000212943	-0.135059069	0.005026096	-0.148587265
0.012323869	0.147442919	0.002174436	-0.015787756	0.744084468	-0.072104563

0.21839233	0.091996827	0.056627147	0.014582691	0.763011191	-0.046707624
0.820741549	0.033694524	0.485885047	0.059288664	0.219846379	0.072417902
0.512726873	0.032952993	0.495540089	0.192565339	5.84E-05	0.116885438
0.321490336	-0.0148752	0.758403578	0.036702123	0.447785664	0.044606491
0.804756782	-0.18036627	0.000169811	-0.342224228	2.93E-13	-0.353394535
0.0001322	0.008969891	0.852863607	-0.100128705	0.037941374	0.201225389
0.731852739	0.014926098	0.757602701	0.320757543	9.57E-12	0.239531531
0.731213404	0.003593615	0.940769992	0.075278913	0.119070914	-0.027724528
0.179719536	-0.051228404	0.289190717	0.055577065	0.250141866	0.245513744
0.108135313	-0.073486553	0.128141533	0.091790428	0.057186453	0.01000658
0.145986999	-0.048214033	0.318542548	-0.049948669	0.301422443	0.155887725
0.815581704	0.034943773	0.469851318	0.083994168	0.081903704	0.080423686
0.772471595	0.129631119	0.007110395	0.231738493	1.19E-06	0.146753369
0.016693767	0.001471365	0.975730445	-0.027189685	0.57392522	0.256950163
0.242140323	0.282215483	2.58E-09	0.184194808	0.000122363	0.208124037
0.606575472	0.030068086	0.534053154	0.122075516	0.01129216	0.117614065
0.587772851	0.121658621	0.011576145	0.0250381	0.604618093	-0.06001987
0.000424675	0.095772736	0.047170158	0.23685641	6.76E-07	0.149327343
0.324262602	0.04759117	0.324841812	0.269902393	1.30E-08	0.367067778
0.643499679	0.02594703	0.591562443	0.040713178	0.399710636	-0.026129802
0.539255837	0.033661676	0.486310581	0.240665784	4.41E-07	0.265296953
0.574940425	-0.033985274	0.482127227	0.071279326	0.140036651	-0.036612723
0.140479822	-0.001681935	0.972258475	-0.284483643	1.90E-09	-0.300224307
0.019162144	0.04123454	0.393697317	0.054646855	0.258169666	0.026574624
0.292769426	-0.013518025	0.779851522	-0.251964732	1.19E-07	-0.38503207
0.157759307	0.071026134	0.141453564	0.217086428	5.55E-06	0.183530553
0.587141209	0.012196999	0.800889949	0.08159464	0.091051678	0.221715959
0.223219254	0.039418422	0.414880358	-0.246468614	2.27E-07	-0.254071396
0.285029514	0.001063871	0.982450593	-0.066322758	0.169812493	0.023858238
0.899718851	-0.036070077	0.455648931	-0.178436515	0.000199809	-0.225446429
0.021488993	-0.022833407	0.636807652	-0.122532105	0.010988223	-0.124244586
0.433116099	0.075800261	0.116528921	0.100365395	0.037487157	0.08746586
0.781917583	0.053112094	0.271800479	0.039828677	0.410037383	0.224096461
0.336535372	0.187539429	9.14E-05	-0.061132153	0.205815115	-0.072266241
0.989733211	0.142656561	0.003029233	0.242175677	3.71E-07	0.133674341
0.227522429	-0.032445998	0.502199659	-0.051089467	0.290502317	-0.001467072
0.317427463	-0.043819087	0.36470174	0.084106487	0.081494399	0.066528374
0.95989271	0.074560063	0.122646664	0.309771336	5.13E-11	0.323868113
0.186298483	0.013995342	0.772288308	0.22611595	2.17E-06	0.346141117
0.431156811	0.153416839	0.001417963	0.295558547	4.07E-10	0.237487573
0.82263832	-0.019595576	0.685331527	0.179696248	0.000179713	0.302399437
0.849574959	0.005299311	0.9127506	0.097532196	0.043237856	0.034889205
0.791316977	0.085429599	0.076796219	0.098948883	0.040275664	0.048010168
0.211707117	0.029983945	0.535198612	0.130178506	0.006869762	0.099482861
0.738304632	-0.049966622	0.30124851	0.108422008	0.024552363	0.067405161
0.20042092	-0.03768501	0.435711626	-0.242152094	3.72E-07	-0.375830998
0.754905128	0.138096141	0.004117132	0.013665212	0.777517049	0.05141482
0.542594093	0.112451895	0.019676137	0.177426804	0.000217415	0.166333635
0.442192811	-0.007102794	0.883242689	-0.010546669	0.827374702	-0.012076168
0.025939303	0.070523061	0.144301417	-0.075410834	0.118423635	-0.272806395
0.478647855	-0.04759708	0.324781665	0.047565238	0.325105818	0.040093114

0.876062883	0.147189233	0.002213526	0.077609442	0.108035999	0.228622055
0.018262568	0.082485085	0.08756577	0.236018103	7.42E-07	0.445120954
0.62789677	-0.061808969	0.200829969	0.129572103	0.007136783	0.183050163
0.165587663	0.069889458	0.147950243	0.077699845	0.107624779	0.157497324
0.595628009	0.010887512	0.821887682	0.021099961	0.662609607	-0.021407997
0.281488298	-0.04011839	0.406637638	-0.049032955	0.310382583	0.082346308
0.743352283	-0.01494835	0.75725265	-0.271680568	1.03E-08	-0.271079016
0.241454798	0.043637944	0.366689545	0.117931464	0.014408896	0.070510584
0.088611461	-0.084231691	0.081040091	0.007550328	0.875943628	0.11118056
0.879768892	0.088539495	0.066616381	0.168266093	0.000458154	0.098011917
0.679350576	0.163248022	0.000678426	0.22756531	1.86E-06	0.20209939
0.501439662	-0.012208435	0.80070718	-0.029135403	0.54681883	-0.058684844
0.629253653	-0.035208807	0.466487537	-0.093764879	0.052019471	0.101941393
0.151073468	0.092614254	0.05498105	0.039769014	0.410739598	0.070031785
0.000122924	0.010269482	0.831843165	-0.122519813	0.010996309	-0.146404172
0.816946844	0.000882514	0.98544186	-0.249711231	1.55E-07	-0.248199223
0.800171182	-0.091606723	0.057688107	-0.094682345	0.049754389	-0.054247777
0.285360278	0.007262066	0.880643877	0.22132604	3.59E-06	0.100845798
0.606263882	-0.038628688	0.424297308	-0.221685703	3.46E-06	-0.119434954
0.744989718	-0.038845458	0.421700185	-0.023360419	0.629047573	-0.025243435
0.424720277	-0.043763849	0.365307189	-0.072571233	0.132975694	-0.141931416
0.067889339	-0.007074493	0.883704601	-0.294748563	4.57E-10	-0.359374075
0.321099317	0.018546087	0.701353979	0.001202558	0.980163285	0.004953175
0.893246885	0.030392373	0.529650018	-0.009328285	0.847055631	-0.192971333
0.376334347	-0.036182373	0.454246223	0.042158319	0.383177537	0.159882779
0.752867949	0.010956065	0.820785128	-0.036210768	0.45389193	-0.063818788
0.266375759	0.0789131	0.10222467	0.029739023	0.538539883	0.075571971
0.360149239	0.039701023	0.411540709	0.09409401	0.051197204	0.373611362
0.185541128	0.018975997	0.694774089	-0.043676877	0.366261741	-0.078052655
0.040034951	-0.04364895	0.366568582	-0.00673947	0.889175639	-0.037192432
0.067453939	0.132656584	0.005869145	0.167079293	0.000503232	0.154245096
0.764670613	0.014789378	0.759754577	-0.2160091	6.19E-06	-0.090684702
0.587145942	0.082148522	0.088870516	0.03665778	0.448334817	0.237164794
0.008846308	0.071142673	0.140800028	-0.058671494	0.224693259	-0.081532297
0.181282938	0.016124733	0.738818808	0.017088567	0.723825777	-0.09975968
0.309829104	0.033059872	0.494142211	-0.076504533	0.11316272	0.004895478
0.227070236	0.09924769	0.039673284	-0.139182002	0.003830103	-0.160227017
0.013372615	0.029178262	0.546228942	0.125002221	0.009466234	0.373338807
0.411655762	0.086129778	0.074400453	0.121385268	0.011765762	0.075033086
0.406355675	0.113474659	0.01858168	-0.03555487	0.462115481	-0.146771655
0.323124235	0.007906259	0.870146026	-0.060763423	0.208568428	-0.234485828
0.060962167	-0.112871155	0.019220882	-0.010189566	0.833132491	0.175624996
0.001529635	-0.024195368	0.616836661	0.134474004	0.005220683	0.001391592
0.520500048	0.012986078	0.788304845	-0.211245509	9.98E-06	-0.243031319
0.233090743	-0.01650297	0.732922937	-0.020124626	0.677307173	0.009143683
0.482276008	-0.082844569	0.086189225	-0.013494259	0.78022864	-0.030581711
0.023986385	-0.039364435	0.415520169	0.009809988	0.83926246	-0.099377142
0.362128774	-0.070303511	0.145557908	-0.310840441	4.37E-11	-0.230161098
0.604108856	0.07083313	0.142541014	0.081162371	0.092783562	0.156952529
0.857745707	0.025537499	0.597428886	0.205583951	1.73E-05	0.207333266
0.531015573	0.098200342	0.041818708	0.167631475	0.000481769	0.182825151

0.811039974	-0.14376543	0.002807707	-0.261487243	3.74E-08	-0.240331327
0.157429619	-0.0517693	0.284122423	0.160220676	0.000855142	0.373542548
0.009029507	-0.051988007	0.282090208	-0.122730627	0.010858348	-0.248734015
0.297339975	0.129891939	0.006994812	0.046637372	0.334643442	-0.076526104
0.096109611	0.074626905	0.122310686	-0.004816229	0.920676721	0.009196515
0.249412078	0.09626787	0.046034373	0.053868406	0.265023198	0.018570157
0.583975086	0.0571478	0.236983915	-0.022701284	0.638759405	0.035680825
0.664646009	-0.12216013	0.011235279	-0.191672889	6.33E-05	-0.089012058
0.988447796	0.44509257	2.60E-22	0.058110232	0.229166701	0.054234633
0.724216837	-0.030695589	0.525549696	-0.10419822	0.030749894	-0.368506428
0.169362816	0.056452903	0.242743645	-0.281958784	2.67E-09	-0.381155194
0.935006844	-0.092131217	0.056265418	-0.045899555	0.342354476	-0.026317441
0.161604385	0.012384555	0.797893802	0.155464111	0.001220383	0.448213476
0.634225664	0.058116244	0.229118447	0.258017501	5.73E-08	0.05995084
0.771648579	0.165005269	0.000592034	0.064101833	0.184594884	0.152611645
0.394034778	0.099020203	0.040131189	0.010841214	0.822632491	-0.314055796
0.022148212	0.039501267	0.413899684	0.009044606	0.851652149	-0.015864347
0.314554096	0.171868146	0.000343285	-0.002247023	0.962943865	0.22946728
0.768287887	0.006560237	0.892104742	-0.099584975	0.039002505	0.000190155
0.749142516	0.105116882	0.029298534	-0.075845903	0.116308421	-0.059126978
0.257271068	-0.051731026	0.284479075	0.035577973	0.461824412	0.011732355
0.085858414	0.077502966	0.108521923	0.10578799	0.02827576	0.216025915
0.629621991	0.030887933	0.522957073	0.222431096	3.20E-06	0.174474422
0.65081695	0.020334311	0.674136796	0.012867098	0.790199038	0.053067724
0.161005301	0.03133663	0.516934595	0.168302043	0.000456849	0.428608556
0.296922448	-0.044949259	0.352451592	-0.046077293	0.340486644	-0.028820707
0.126774342	-0.007258459	0.880702708	-0.00079049	0.986959761	-0.030057922
0.703483989	0.058029667	0.229813976	0.209830927	1.15E-05	0.072721213
0.739240895	0.123872344	0.01013749	-0.089484032	0.063753513	-0.012206336
0.293271935	-0.023291303	0.63006299	-0.148962321	0.00195322	-0.089357895
0.165383506	-0.054185016	0.262220814	0.06647322	0.168843973	0.238762046
0.936349224	0.013365919	0.782266076	0.112705279	0.01939989	0.044962886
0.879601476	-0.063043661	0.191962996	0.144540089	0.002661808	0.015254449
0.355643869	0.066024801	0.171742677	0.178419651	0.000200092	0.163403884
0.051579521	0.024034426	0.619182332	-0.142639407	0.003032781	-0.274236412
0.432950383	0.041043046	0.395899613	0.147560585	0.00215652	0.025359046
0.017740667	0.026076173	0.589717984	-0.152660801	0.001498071	-0.214052782
0.042784745	-0.022594772	0.640334636	0.061791825	0.200955147	0.222837562
0.706609876	0.0885694	0.066524149	-0.078087132	0.105877062	-0.33349506
0.927022176	0.010061246	0.835203673	0.054660553	0.258050173	-0.003506322
0.559614012	0.03035506	0.530155717	0.021214827	0.660887009	0.048889282
0.222148576	0.043487778	0.368342513	-0.022058732	0.648286699	0.150750281
0.535580839	0.098998895	0.040174308	-0.139496791	0.00375035	-0.143618696
0.145291804	0.08704809	0.071350927	-0.06623799	0.170359973	-0.114157838
0.360624493	-0.000755938	0.987529709	0.049636266	0.304459776	0.09948522
0.778946942	-0.019046067	0.693703795	0.187942972	8.82E-05	0.289286625
0.779434723	-0.097697121	0.042883836	-0.171827476	0.000344417	-0.133385327
0.476068425	0.019545688	0.686090051	0.056655423	0.241055	0.070411033
0.886308526	0.235745564	7.65E-07	0.137204614	0.004367181	0.037968259
0.074074762	0.209077789	1.24E-05	0.033971683	0.482302523	0.035918115
0.080033158	0.273891034	7.75E-09	0.12050508	0.012395105	0.138061343

0.676516098	0.013915694	0.773548881	-0.243739779	3.11E-07	-0.153394948
0.104532862	0.066708636	0.167336907	-0.011963523	0.804623731	-0.075883016
0.056598133	-0.061028209	0.206588589	-0.091964898	0.056713371	0.047056748
0.000116756	0.052386933	0.278408773	-0.04161434	0.389351307	-0.18302278
0.040064095	0.027537609	0.569031915	0.001017269	0.983219211	-0.011294724
0.545203861	0.165082896	0.000588464	0.025590047	0.596674674	0.030646465
0.016845898	-0.045551948	0.346026303	0.132520738	0.005920405	0.440042138
0.751114797	0.033106432	0.493533916	-0.049221179	0.30852668	-0.066091995
0.049023526	-0.056410392	0.243099151	0.029696528	0.539120661	0.291137334
0.008353767	0.115363558	0.016699326	0.142166761	0.003132048	0.194909443
0.000329923	0.078500519	0.104036393	-0.046802046	0.332937755	-0.278967741
0.192322062	0.134649958	0.005161461	-0.028913149	0.549882895	0.095913007
0.854254427	0.107934458	0.025207771	-0.015366774	0.750679573	-0.268764676
0.291322147	-0.020568928	0.670596261	0.011380446	0.813967642	0.192981422
0.033005827	-0.010563593	0.82710204	-0.14778353	0.002122943	-0.360521784
0.07162576	0.022522253	0.64140806	-0.145800488	0.002439118	-0.357807617
0.572266987	-0.059393392	0.219031388	-0.149568057	0.001870921	-0.032629149
0.457355781	-0.127863326	0.007939855	0.275367285	6.39E-09	0.383128764
0.626453137	0.150671701	0.001729084	-0.014897765	0.758048492	0.066140866
0.590855964	0.128547071	0.00760931	-0.082250352	0.08847412	-0.391986509
0.930311939	0.08484142	0.078856861	-0.0264528	0.584354038	-0.195102232
0.012951212	-0.045404396	0.347592443	0.134329732	0.005269697	0.048725234
0.494381216	-0.002139856	0.964709999	0.047276992	0.328049665	0.210572507
0.500465524	-0.054913623	0.255849443	-0.06207765	0.198875512	-0.171844839
0.100159106	0.002714052	0.95524935	-0.192249435	6.01E-05	-0.204217678
0.883798506	0.014900861	0.757999776	0.157900224	0.00101841	0.225208064
0.428593428	0.077505847	0.108508753	0.066823207	0.166607101	0.004240831
0.201549153	0.019372413	0.688727043	0.031490444	0.514878359	0.064897815
0.46606988	0.336515672	7.61E-13	0.099420678	0.039328043	0.155589313
0.104605406	0.044401586	0.358355167	0.031269425	0.517834349	0.061156049
0.282171115	0.016205884	0.737552542	-0.048220796	0.31847459	0.109460213
0.886144578	0.256467924	6.92E-08	0.104958627	0.029544284	0.04121558
0.83296598	-0.046035863	0.340921447	0.033731459	0.485406797	0.117205467
0.789254427	-0.05513384	0.253945009	0.160544891	0.000834362	0.059494792
0.062604151	0.034105878	0.4805731	0.152420346	0.001524407	0.241334683
0.033387493	-0.069451474	0.150513303	0.224942022	2.46E-06	0.445610601
0.054704997	0.024145275	0.617566331	-0.207542746	1.43E-05	-0.301458505
0.674692897	-0.046012069	0.341171323	-0.027265295	0.572860131	0.006953017
0.091338496	0.024811347	0.607895088	-0.095584713	0.047607574	-0.121665927
0.381229242	0.006024236	0.900872911	0.225729966	2.26E-06	0.199885825
0.692725186	0.232779781	1.06E-06	0.09827123	0.041670473	0.177998124
0.07100712	0.197440864	3.74E-05	0.072161361	0.135185475	0.139856588
0.294949291	-0.179819861	0.000177846	-0.167239733	0.000496906	-0.08313554
0.656272098	-0.025686939	0.59528513	0.010283617	0.831615174	0.041785532
0.093143276	0.06092763	0.207339022	0.046252576	0.33865098	0.15453535
0.979372708	0.006039466	0.900623599	-0.037543303	0.437440761	-0.05947677
0.162796407	0.00994957	0.837007132	-0.252677834	1.09E-07	-0.223107295
0.264519178	-0.018454853	0.702753253	-0.202775449	2.27E-05	-0.223111102
0.699587982	0.068833187	0.154188725	0.197006808	3.89E-05	0.110905583
0.941271935	-0.009635711	0.842080208	-0.254137878	9.17E-08	-0.392177864
0.004281732	0.106558899	0.027138866	0.175103563	0.000263588	0.156911045

0.01291681	-0.002243402	0.963003543	-0.021810835	0.65197785	0.04399025
0.210673782	0.004777478	0.92131288	0.153875078	0.001371345	-0.259453
0.581167049	0.05965082	0.217037278	0.115725185	0.016358626	0.148773562
0.453834811	-0.17655394	0.000233795	-0.131701728	0.006238061	-0.159747783
0.015082788	-0.05356832	0.267698242	-0.110382796	0.022062903	-0.285938178
0.183690574	0.077957663	0.106458805	0.185335657	0.000110837	0.291220946
0.756027694	-0.140933405	0.003405137	-0.056069288	0.245964959	0.133027223
0.184467894	0.18450827	0.000119088	0.095665357	0.047419548	0.143202455
0.372413563	0.176481672	0.000235202	0.2520485	1.18E-07	0.179921343
0.220142934	0.021144847	0.66193626	0.01330994	0.78315523	0.035280485
0.228368438	-0.059411085	0.218893915	-0.069898849	0.14789565	-0.107011094
0.656402308	0.019911333	0.680537971	0.356289352	2.57E-14	0.411224743
0.997366093	-0.029267026	0.54500822	-0.105733323	0.02835791	-0.157250699
0.638512925	0.108931383	0.023883384	0.056893971	0.239076543	0.018238777
0.332289003	0.006281235	0.896667218	-0.021285485	0.659828279	-0.108188611
0.000733466	-0.130720584	0.006638674	0.088017398	0.068243592	0.217524639
0.432203902	0.130112084	0.006898567	-0.008322565	0.863373846	-0.018445983
0.925613281	-0.055933168	0.247115143	-0.095623151	0.047517876	-0.221868223
0.699587151	0.061889965	0.200239317	0.35469553	3.41E-14	0.337332546
0.105193285	-0.005057545	0.916716329	-0.106954792	0.026570461	-0.120779467
0.29824308	-0.011135009	0.817908789	-0.059643412	0.217094481	-0.055872565
0.10976327	0.118707261	0.013773547	0.057266501	0.236009759	0.214499256
0.028398402	-0.006724254	0.889424232	-0.30111341	1.84E-10	-0.455585458
0.181511845	-0.179346231	0.000185098	-0.219181364	4.48E-06	-0.188317837
0.935956162	0.004065291	0.933013	-0.041065524	0.395640715	-0.128353834
0.305845218	-0.005084779	0.916269504	0.143916401	0.002778715	0.073861545
0.467042211	-0.031370416	0.516482569	-0.002251192	0.962875163	0.073209604
0.01661905	0.071302754	0.13990609	-0.20714321	1.49E-05	-0.437916132
0.006685016	0.073381189	0.128690968	-0.16851884	0.000449053	-0.297463378
0.147166336	0.044185981	0.360696159	-0.006151103	0.898796435	0.186541047
0.190277266	0.078155614	0.105570369	-0.053895481	0.264782746	-0.073337368
0.543874039	0.254048682	9.26E-08	0.357854121	1.95E-14	0.096446674
0.360794034	-0.173494464	0.000300772	-0.064761981	0.180105139	0.066738205
0.334028082	0.313605603	2.88E-11	-0.020375432	0.673515728	-0.078972531
0.820740908	-0.018710342	0.698837346	-0.009162084	0.849748005	0.175324643
0.107330902	0.095776133	0.047162287	0.2104018	1.08E-05	0.276344112
0.862626238	-0.024777637	0.608382932	-0.212283629	9.00E-06	-0.011555502
0.367368547	-0.046216981	0.339023235	-0.04166322	0.388794102	-0.027922522
0.482612171	-0.020086877	0.677878543	-0.00045713	0.992458783	0.100122424
0.068477996	0.008562491	0.859475406	-0.005336895	0.912134295	0.106294272
0.071490905	0.026476652	0.584015093	0.367071379	3.67E-15	0.433412201
0.110521105	-0.041188977	0.394220639	-0.174701534	0.000272453	-0.218020145
0.442115204	0.081690752	0.090670148	0.110665049	0.021723202	0.019347772
0.835544419	0.09313629	0.053620468	-0.060945771	0.207203525	0.000425902
0.75207797	0.099029512	0.040112363	-0.025427676	0.599006544	-0.205749439
0.938186077	-0.100353906	0.037509097	-0.150479991	0.001752993	-0.108977854
0.028356873	0.035372662	0.464414593	0.155950755	0.001177306	0.130784865
0.330291006	0.023750055	0.623336404	-0.316105745	1.96E-11	-0.36888073
0.65315595	-0.040864401	0.397960803	0.093614268	0.052399398	0.235968948
0.006948169	0.084239985	0.081010068	-0.194459898	4.92E-05	-0.293897831
0.617318725	0.056410655	0.243096951	0.000155893	0.99742822	-0.024447558

0.560966007	0.112661568	0.019447303	0.00447136	0.926339996	-0.169005431
0.04297976	-0.006907008	0.886439006	-0.137918543	0.004165886	-0.244800865
0.51236905	-0.207790827	1.40E-05	-0.152638084	0.001500542	-0.168107533
0.191162235	-0.044404329	0.358325442	0.055970363	0.246800481	-0.008293514
0.989965401	0.059365331	0.219249544	0.044945771	0.352488992	-0.003434513
0.585291726	-0.018938297	0.695350194	-0.007685742	0.873737124	0.127409639
0.821516697	0.083120286	0.085145285	0.189876487	7.43E-05	0.125744324
0.530340079	0.01805485	0.708899908	0.055956556	0.246917253	0.03630185
0.125140042	-0.044529869	0.356966823	-0.022901122	0.635808328	0.052269075
0.602904808	0.053174235	0.271239221	-0.005519382	0.909142618	0.007687646
0.856845508	0.011850701	0.806429601	-0.075774523	0.116653411	-0.064353859
0.847824014	-0.063680788	0.187501259	0.075208878	0.119415665	0.233323833
0.382056745	0.02374097	0.623469313	-0.044039525	0.362291792	-0.189955133
0.670070299	0.061859174	0.200463705	-0.196843399	3.95E-05	-0.263608772
0.011272535	-0.041932579	0.385732234	-0.20082031	2.73E-05	-0.279298629
0.89357975	-0.020065142	0.678207596	-0.196626496	4.03E-05	-0.338968359
0.337236156	0.122896611	0.010750805	-0.046141987	0.33980839	0.105822745
0.013540442	-0.076168823	0.11475766	-0.08852341	0.066666033	-0.1910388
0.974372796	-0.02256185	0.640821856	0.024103335	0.61817754	0.053880464
0.698891419	0.031487437	0.514918516	-0.00103669	0.982898906	-0.037590656
0.18491992	0.03820272	0.42942792	-0.017752808	0.713553812	0.014646934
0.14400847	-0.173586403	0.000298522	-0.260290062	4.34E-08	-0.193251016
0.481504601	0.036234809	0.45359208	-0.118965597	0.01356752	-0.048317725
0.098347348	-0.046210249	0.339093674	-0.05975688	0.216219504	0.123233983
0.810801368	-0.036484662	0.450482388	-0.10491822	0.029607314	0.01667399
0.36036327	-0.135336745	0.00493605	-0.002633568	0.956575078	-0.0528154
0.743745905	0.007216189	0.881392318	-0.013633739	0.778016062	-0.071149376
0.358104063	-0.006100388	0.899626415	0.076915078	0.111235951	0.210298029
0.179157736	0.177774953	0.000211187	0.004947059	0.918529338	-0.217670767
0.29587209	0.079102017	0.101403488	0.045725088	0.344194279	0.048532831
0.29686806	0.082899502	0.085980414	-0.016437221	0.733946695	-0.183424739
0.738540771	0.048439435	0.316282744	0.234277781	8.99E-07	0.048689025
0.003363788	0.121984609	0.011353554	-0.004764202	0.921530843	-0.205136444
0.544386549	0.011378582	0.813997559	-0.021480242	0.656913562	-0.007668667
0.820501354	0.125653272	0.009097651	-0.015023029	0.75607821	-0.151397706
0.572278523	0.137164275	0.004378813	-0.135712178	0.004816616	-0.073430625
0.645279031	-0.083529234	0.08361569	-0.222208076	3.28E-06	-0.34731303
0.029709671	0.019688107	0.683925476	0.051087148	0.290524236	-0.070250713
0.902782604	0.044324112	0.359195262	0.002821131	0.953485734	-0.009699514
0.36870077	0.100714983	0.036824723	0.091182054	0.058861736	-0.148066078
0.891083381	-0.016938592	0.726151979	-0.03477814	0.471960264	-0.050250746
0.346443059	-0.005751268	0.90534297	-0.186660558	9.87E-05	-0.271372665
0.61154157	0.033601382	0.487092202	0.121108045	0.011960862	0.17770596
0.124222881	0.15627908	0.001149039	0.250678393	1.38E-07	0.155022084
0.449799011	0.015673616	0.745870763	0.151443946	0.001635773	0.153670092
0.950535209	0.066361733	0.169561221	0.138928638	0.003895405	0.230116224
0.383654842	-0.00128509	0.978802197	-0.031512658	0.514581737	-0.037028252
0.192052169	0.037847897	0.433728911	0.197123667	3.85E-05	0.172440759
0.628691918	-0.103195644	0.032403603	0.237961745	5.98E-07	0.305839348
0.992183522	-0.026116662	0.589140255	-0.064522918	0.181721603	-0.016494969
0.617063217	-0.02389186	0.62126342	0.096267473	0.046035273	0.071424519

0.078723409	-0.095618016	0.04752985	-0.104576243	0.030145385	0.009345167
0.984089232	0.007285608	0.880259844	0.275865536	5.99E-09	0.297130952
0.845785257	-0.019657877	0.684384719	0.058020713	0.229885998	0.094913295
0.19634266	0.010204429	0.832892661	-0.090125719	0.061866949	-0.278535474
0.072031285	0.077517638	0.108454861	0.37828326	4.46E-16	0.276337164
0.917529618	0.11533807	0.016723571	0.009884496	0.838058421	-0.101308737
0.412621449	0.106061463	0.027867864	0.010041637	0.835520276	0.055731578
0.403488217	0.01034049	0.830697937	0.070813103	0.142654218	0.161878781
0.004048701	0.134005746	0.005381279	0.144330399	0.002700603	0.37660168
0.932072136	0.017149316	0.722884244	0.136652962	0.004528694	0.07980314
0.046824432	0.296129743	3.76E-10	0.178536848	0.000198134	0.091321115
0.748953492	-0.142361614	0.003090772	0.047100595	0.329859685	0.209236381
0.007519519	-0.02884987	0.550756829	-0.09897081	0.040231199	-0.067520081
0.173914346	-0.045473583	0.346857519	-0.000447955	0.992610131	-0.074109253
0.242539929	0.065248311	0.176849632	0.32594659	4.23E-12	0.167898338
0.030431978	-0.030958467	0.522007988	-0.151253511	0.001658345	-0.193281736
0.165821234	0.209838809	1.15E-05	0.06879504	0.1544177	0.080304703
0.971405662	0.06554053	0.174914644	-0.042983812	0.373923681	0.034882989
0.001699489	0.095235203	0.048429698	-0.065689058	0.173937184	-0.063274563
0.532251289	-0.032561091	0.50068375	0.054789757	0.256924966	-0.102043775
0.00171173	0.060819964	0.20814452	0.231728839	1.19E-06	0.045965533
0.616769994	0.241754447	3.90E-07	-0.052077298	0.281263351	-0.210802137
0.941530837	-0.125504658	0.009180643	0.19724034	3.81E-05	0.294279717
0.007806432	-0.020480637	0.671927792	-0.115740173	0.016344637	-0.249777092
0.064279245	0.010332957	0.830819411	0.089662375	0.063224493	0.287498748
0.327425589	0.073626364	0.12741528	0.124220832	0.009926085	-0.013410761
0.051106396	0.082315744	0.088220314	0.032166073	0.505896709	0.03956351
0.98762368	0.127954617	0.007894993	0.069547749	0.149947037	0.178846933
0.831828656	0.090334042	0.061264469	0.189603841	7.61E-05	0.247093677
0.509194899	-0.042582449	0.378405666	0.015928242	0.741887755	0.02313128
0.735436456	-0.047323564	0.327572867	-0.219808045	4.20E-06	-0.362936401
0.273302467	-0.040435374	0.402937138	0.086326156	0.073739554	0.086723098
0.646080753	0.172727024	0.000320181	0.0977558	0.042758469	-0.024575692
0.059374862	0.053667645	0.266810793	-0.011419906	0.813334439	-0.078809154
0.868117478	0.068640101	0.155350346	0.037003877	0.444058822	0.079617012
0.667316167	0.129252174	0.007281368	0.167800829	0.000475359	0.228814139
0.236087974	0.06102793	0.20659067	0.223601087	2.83E-06	0.234889389
5.58E-06	-0.004811625	0.920752301	0.29500697	4.40E-10	0.102618529
0.020136223	0.054664435	0.258016318	0.225596393	2.30E-06	0.07965669
0.876029156	0.149158542	0.001926204	0.200152007	2.91E-05	0.045669509
0.328700809	0.101248823	0.035832336	0.150689704	0.001726855	0.127256597
0.816658193	0.058221726	0.22827307	0.005135097	0.915443999	0.053045116
0.698507814	0.066649677	0.167713394	0.058247739	0.22806493	-0.063733246
0.468855886	-0.091570479	0.057787509	0.015172668	0.753726592	0.072438682
0.33465095	0.051923323	0.282690227	-0.048922337	0.311476711	-0.045154504
0.547239444	-0.024288769	0.615477158	0.284334011	1.93E-09	0.256587211
0.218063357	-0.006999358	0.884931117	-0.146354467	0.002346726	-0.255844419
0.237769988	-0.101576075	0.035235295	-0.05550699	0.250740501	-0.001267068
0.10205439	0.008703285	0.857189284	0.014850822	0.758787257	0.060091159
0.953287398	0.022768731	0.637762739	0.049795061	0.302913371	0.045569709
0.024237176	-0.042680593	0.377306664	-0.24700059	2.13E-07	-0.363720669

0.824381088	0.010064787	0.835146511	-0.022302945	0.644658814	-0.01733045
0.740731866	-0.020336795	0.674099264	-0.138329967	0.00405373	-0.142545858
0.122314186	0.062953971	0.192597278	-0.129286899	0.007265549	-0.088969386
0.301635747	-0.02445379	0.6130784	-0.093519553	0.052639505	0.019507962
0.404998838	0.190226541	7.20E-05	-0.042146709	0.383308672	0.043486717
0.403311891	-0.083169218	0.084961085	-0.192656928	5.79E-05	-0.129341547
0.43332041	0.071320675	0.13980629	0.240151897	4.67E-07	0.297011198
0.297282335	0.141829725	0.003204627	0.09606292	0.046501691	0.185961407
0.640411726	-0.024224727	0.616409186	0.061947195	0.199822739	0.068580394
0.282295615	0.094148674	0.051061693	0.057802221	0.23164832	-0.14421747
0.415250656	0.095140113	0.048655418	-0.033699226	0.485824141	0.003722794
0.217169439	0.017165953	0.722626475	-0.245043795	2.67E-07	-0.292537681
0.021214773	0.022007243	0.649052659	0.051690613	0.28485598	-0.164556666
0.340396257	-0.032205659	0.505373012	0.004595362	0.924303247	-0.069761591
0.000869438	0.022869528	0.636274506	-0.166574512	0.000523627	-0.358295526
0.091839484	-0.034085225	0.480839039	0.13086731	0.006577335	0.136136473
0.710414765	0.081026465	0.093333479	0.011438123	0.813042154	-0.071009656
0.774468796	0.062141287	0.198414638	0.119726465	0.012976397	0.17736996
0.688199236	-0.025734614	0.594601971	0.086929279	0.071739628	0.06406144
0.384413143	0.07049413	0.144466511	-0.051712459	0.284652199	-0.146893724
0.078137038	0.095833064	0.047030528	0.096282878	0.046000308	0.022126552
0.617195289	0.126166615	0.00881609	0.081581524	0.091103844	-0.025624214
0.292191207	-0.064208962	0.183860729	0.147400321	0.002180955	0.211351043
0.206945692	0.02408476	0.618448315	-0.270560577	1.19E-08	-0.341019729
0.79576164	0.08493927	0.078510976	0.127617429	0.008061826	0.204406088
0.120061056	0.067049161	0.1651748	0.237529042	6.27E-07	0.093587249
0.037529014	0.093843558	0.051821914	-0.028140186	0.560604406	0.132407349
0.911770462	0.02907187	0.547693859	-0.001007298	0.98338367	0.010559447
0.231290182	0.289801812	9.13E-10	0.048667267	0.314009259	0.087185794
0.000334811	-0.060981362	0.206937873	0.070946507	0.141901438	0.209525078
0.975385853	0.237603362	6.22E-07	-0.025828694	0.593254874	-0.123404916
0.456138161	0.013506422	0.780035627	0.127005751	0.008372515	0.111203343
0.431726463	0.055753556	0.248638559	0.01832613	0.704729188	-0.048270749
0.164669595	-0.026383392	0.585340849	0.036777172	0.44685711	0.059157487
0.174773758	0.079534013	0.099545375	-0.241287048	4.11E-07	-0.340648426
0.431151955	0.071359256	0.13959161	0.017510147	0.717300502	0.156156432
0.176964062	-0.040681762	0.400074725	-0.104345999	0.030512352	-0.048024802
0.719824204	0.014183895	0.769306523	-0.24832078	1.83E-07	0.009203626
0.155504849	0.012232678	0.800319764	-0.086299031	0.073830556	-0.159383974
0.392677553	0.086486037	0.073205023	-0.131012519	0.00651713	-0.245857403
0.284736202	-0.013098323	0.786519027	0.043042114	0.373275365	0.095201656
0.155024947	0.058541549	0.225723389	-0.110588971	0.021814315	-0.149396728
0.500662777	-0.026058216	0.589974297	-0.049934827	0.301556591	-0.046054326
0.776513584	0.095602396	0.047566291	-0.111709098	0.020505777	-0.189523263
0.150665705	-0.069705189	0.149024495	-0.190204119	7.22E-05	-0.247591408
0.347339867	0.069124377	0.152449363	-0.251090108	1.32E-07	-0.361824673
0.933237447	0.009125352	0.850343285	0.066715562	0.167292716	-0.027025275
0.510466165	-0.007632769	0.874600181	0.025518699	0.597698811	-0.06928055
0.701493848	0.060117316	0.213456891	0.253103631	1.04E-07	0.136539135
0.212933735	0.101523201	0.035331181	0.238228003	5.80E-07	0.238223106
0.084142026	0.084802635	0.078994302	-0.021885445	0.650866026	-0.023341717

0.22896335	0.113847277	0.018196345	0.288004737	1.17E-09	0.220709266
0.986220329	-0.005620013	0.907493443	0.004544642	0.925136267	0.054500507
0.158066726	0.161922668	0.000751157	0.093492878	0.052707291	0.34091412
0.979747565	0.045049791	0.35137462	0.098629698	0.040927647	0.139755657
0.354985806	0.071101789	0.141029039	-0.015851205	0.743092085	0.20738454
0.946074162	-0.007484934	0.877009526	0.154052472	0.001353679	0.141505505
0.178379836	0.494345413	7.02E-28	0.040624864	0.400734655	0.18668444
0.618683637	0.140326628	0.003547265	0.187208101	9.41E-05	0.297352749
0.097234529	0.049253148	0.308212186	-0.160145662	0.000860017	-0.247938324
0.043529735	-0.022335842	0.644170753	-0.0698303	0.148294477	-0.20602285
0.455518491	0.008430352	0.861622051	0.027560438	0.568711542	0.027724217
0.708566944	0.077721797	0.107525109	-0.121236109	0.011870383	-0.084075836
0.165720483	-0.048288749	0.317792302	-0.001674603	0.972379364	-0.058476837
0.912691107	-0.017837601	0.712246224	0.036044828	0.455964658	0.173032782
0.608850391	0.013641587	0.777891618	0.17023422	0.000391586	0.167000324
0.395591136	0.03854279	0.425329031	0.144386067	0.002690254	0.221264855
0.169311758	-0.024655039	0.6101586	0.022871162	0.636250387	0.021181927
0.352148881	0.111023968	0.021297791	0.21105209	1.02E-05	0.208906564
0.134764197	0.004939475	0.918653796	-0.118093642	0.014273989	-0.123390162
0.143972438	0.15367193	0.001391835	0.195450847	4.49E-05	0.091265389
0.503571284	0.05107457	0.290643179	0.144786567	0.002616855	0.159139425
0.013071184	0.070743905	0.143045889	-0.015915922	0.74208031	-0.132190855
0.783795624	-0.027055855	0.575812714	0.113548636	0.018504617	0.339484588
0.175454976	0.126698375	0.008532634	0.07326358	0.129306407	-0.020426836
0.202201756	-0.042121338	0.383595336	0.026733628	0.580369235	0.008568225
0.254315454	0.017428846	0.718557303	0.021644786	0.654455067	0.028107957
0.684667058	-0.060124838	0.213399511	-0.114438442	0.017599305	-0.228780063
0.250441921	0.030397462	0.529581066	-0.133934031	0.005406264	0.045124446
0.205537	-0.064765532	0.180081204	-0.038755221	0.422780181	0.096032975
0.98279258	0.001959735	0.967678821	0.026407279	0.585001151	0.014415698
0.635423348	-0.098236158	0.041743757	-0.120332066	0.012522247	0.000558234
0.352637309	0.063777143	0.186833192	0.146216607	0.002369415	0.112593342
0.865801192	0.058083977	0.229377497	-0.006928415	0.88608944	0.002552091
0.473617214	0.137799309	0.00419891	0.345383325	1.72E-13	0.330010198
0.108282876	-0.128209626	0.007770874	-0.064439739	0.182286541	0.2668744
0.133307512	0.001879629	0.968999305	-0.132237799	0.006028463	-0.260682194
0.813262061	-0.068367793	0.156999809	0.027385896	0.571163215	0.010292874
0.007054746	0.111940986	0.020243573	-0.102731188	0.033195077	0.015116022
0.775249908	-0.057837999	0.23135909	-0.245383589	2.57E-07	-0.231845398
0.005414417	0.108071618	0.02502188	-0.05613078	0.245446594	-0.218425549
0.123412672	0.042081056	0.384050749	-0.255524983	7.76E-08	-0.308448501
0.605011204	-0.056581049	0.241674177	-0.108704326	0.024179615	-0.019782803
0.257474795	0.146057853	0.00239579	-0.080650003	0.094870379	-0.022350506
0.32762393	-0.04513522	0.350461074	0.06118956	0.20538883	0.241452123
0.700680198	0.21524877	6.69E-06	0.020083363	0.677931737	-0.088886244
0.760063293	0.104683669	0.029975465	0.154876744	0.001274306	0.198291054
0.144669506	-0.083283641	0.084531604	0.124986035	0.009475564	-0.020603512
0.045456858	0.055723366	0.248895263	0.032197746	0.505477677	-0.087574188
0.815374861	0.050744154	0.293779413	-0.182865481	0.000137211	-0.193018373
0.321599891	0.007273564	0.88045632	0.090939417	0.059541119	0.020908711
0.612544478	-0.006925673	0.886134208	0.089611205	0.063375909	0.211946622

0.944508448	0.140158363	0.00358762	-0.02208204	0.64794009	0.110495166
0.92220038	-0.058829845	0.223442468	-0.149694541	0.001854142	-0.206327296
0.13597567	-0.012729546	0.792390434	-0.294498365	4.73E-10	-0.506119161
0.993358492	0.015467111	0.749106035	-0.011086147	0.818693956	0.106153587
0.141842512	0.051537409	0.286287896	-0.242039995	3.77E-07	-0.154129863
0.014278918	-0.061141911	0.20574261	-0.123945917	0.010092528	-0.012179541
0.907084213	-0.008198516	0.865390771	0.001515914	0.974995849	-0.093323628
0.405001256	0.053769834	0.265899857	-0.113900306	0.018142077	-0.066489061
0.697410585	0.109853211	0.022712713	0.036356273	0.452078816	-0.008633603
0.106635581	0.004840444	0.920279231	0.077583775	0.108152979	0.233162604
0.401198009	0.010453604	0.828874364	-0.191898869	6.20E-05	-0.127653278
0.918262181	0.080066372	0.097292918	0.339344404	4.76E-13	0.494741396
0.098614923	0.012546198	0.795313979	-0.147389956	0.002182544	-0.079641359
0.92730281	-0.062573535	0.195304779	0.031395091	0.516152577	0.126415835
0.270984388	-0.007097536	0.883328499	0.009325799	0.847095903	0.014656656
0.588654794	-0.007900048	0.870247144	0.011735386	0.808276438	-0.061708871
0.713637601	-0.056711827	0.240586168	-0.145028846	0.002573343	0.121258525
0.739927153	-0.013300287	0.783308577	-0.014699409	0.761171633	0.052428367
0.711459504	0.044041921	0.362265648	0.081684083	0.090696577	0.141201266
0.00155105	-0.016876655	0.727113384	0.044808025	0.353968105	0.167908764
0.163938048	-0.03803032	0.431514567	0.073423255	0.128471392	-0.204174789
0.758964769	0.045917154	0.342169245	0.124680047	0.00965349	0.26215117
0.166139489	-0.141122457	0.003361922	-0.256169044	7.18E-08	-0.239129361
0.293568665	-0.040061206	0.40730735	-0.010290703	0.831500879	0.026007491
0.00212086	0.056396233	0.243217646	-0.075032125	0.120289209	-0.18899766
0.384841825	0.028157098	0.56036875	-0.063934173	0.185748199	0.077268488
0.062510605	0.009318401	0.847215698	0.034802867	0.471645094	0.062479361
0.593982387	-0.017455939	0.718138394	-0.09585782	0.046973329	-0.157667221
0.172414609	0.325736976	4.37E-12	0.032407583	0.502706181	0.081331472
0.793836673	0.041207834	0.394003997	-0.004798288	0.920971258	0.052913962
0.552556435	-0.016076213	0.739576254	0.056603597	0.241486344	0.055509641
0.07671497	0.009701479	0.841016619	0.19479528	4.77E-05	0.260390976
0.469446932	0.087469773	0.069985209	0.212013725	9.24E-06	0.146563818
0.038464039	0.108003307	0.025114312	-0.077458449	0.108725595	-0.103381929
0.458980058	-0.041621598	0.389268537	-0.042187648	0.38284637	-0.035930786
0.176375634	-0.00411322	0.932225111	-0.166729871	0.000517269	-0.15496017
0.833628894	0.032850423	0.496883587	0.034381747	0.477028461	-0.043379986
0.167755403	-0.04257533	0.378485456	-0.009597946	0.842691065	-0.064515829
0.532119217	0.057862691	0.231159623	0.114921719	0.017124009	0.218808704
0.048661221	-0.136801707	0.004484622	-0.289128158	1.00E-09	-0.253152619
0.555193343	-0.017582791	0.716178157	-0.023150625	0.6321319	0.030083448
0.758413806	-0.006331749	0.895840917	-0.00512827	0.915555994	-0.121140214
0.311891777	0.071634589	0.138066897	-0.204861448	1.86E-05	-0.166080221
0.312094759	0.093339151	0.053099367	0.074739576	0.121745982	0.220398511
0.295067057	0.000310411	0.994879151	0.008276375	0.86412475	0.090546005
0.845705671	0.0946621	0.049803467	0.017950631	0.71050452	-0.173106639
0.186995385	0.246268589	2.32E-07	0.282302732	2.55E-09	0.271514112
0.100123523	0.145446808	0.002499827	0.106723807	0.026900841	-0.087639973
0.265464641	0.087213245	0.070813464	0.081211686	0.092584659	0.197707465
0.013888911	-0.017988606	0.709919685	-0.084589092	0.079754507	-0.181553279
0.768388133	-0.126493414	0.008640913	-0.077777504	0.107272513	-0.193537261

0.089154939	0.064613241	0.181109613	-0.177417943	0.000217576	-0.306602122
0.630144427	-0.013467303	0.780656448	-0.039455222	0.414444571	0.2159702
0.198390612	-0.012106639	0.802334474	0.032596395	0.500219237	-0.208884094
0.543475094	-0.032189056	0.505592622	-0.006116304	0.899365925	0.025852136
0.34956512	0.087224531	0.070776855	-0.122369641	0.01109553	-0.046473239
0.041091095	-0.068022759	0.159108752	0.036827554	0.446234373	0.115804737
0.677083755	0.006353529	0.895484679	0.152168394	0.001552458	0.147249214
0.925495717	0.062016997	0.199315504	0.26262506	3.25E-08	0.209739377
0.204554113	0.022304751	0.644632015	-0.078789101	0.102766523	-0.010889943
0.321000375	-0.040213095	0.405529934	-0.037562953	0.437200753	0.007577828
0.96461689	-0.137273788	0.0043473	-0.199195817	3.18E-05	-0.265966417
0.922698387	0.306393512	8.48E-11	0.246104953	2.36E-07	0.164944496
0.220994997	0.032679363	0.499128477	-0.072246868	0.134722154	-0.076269953
0.904209851	-0.041994745	0.385027669	0.307580134	7.11E-11	0.381387002
0.896074387	-0.016866061	0.727277873	0.101010252	0.036272985	0.071599412
0.038182787	0.195991469	4.28E-05	0.056701132	0.240675015	0.090562745
0.331276922	-0.174499427	0.000277014	-0.132778269	0.005823567	-0.112397037
0.408013504	0.119266496	0.013330974	-0.037260904	0.440898348	-0.246749319
0.10821842	0.061683964	0.201744031	0.023427323	0.628065329	0.015348997
0.419775839	-0.035655824	0.460844366	0.031785195	0.510949875	0.020265652
0.002417011	0.01667181	0.730296126	-0.062811252	0.193609748	-0.289076436
0.305250611	0.075352552	0.118709258	-0.112558966	0.01955899	-0.307107991
0.969071584	0.019063331	0.693440194	-0.054704293	0.257668873	-0.088334041
0.322816135	-0.152106165	0.001559459	-0.237592098	6.23E-07	-0.241689931
0.314678225	0.117784547	0.014532075	0.090208489	0.061626992	0.02614761
0.370695648	-0.009986422	0.836411915	-0.121839597	0.011452099	-0.06585078
0.515066571	0.304404842	1.14E-10	-0.043407466	0.369228448	-0.05282415
0.000970692	0.09913811	0.039893302	0.006911883	0.886359403	-0.245967041
0.356512744	0.005518743	0.909153105	0.006079832	0.899962862	0.021493635
0.475215408	-0.161200237	0.000793776	-0.235624708	7.75E-07	-0.156110223
0.904451681	0.125221024	0.009340907	0.15689691	0.001097535	0.149960092
0.685988532	0.031059331	0.52065232	-0.127225932	0.00825947	-0.278778908
0.216680658	0.149310413	0.00190553	0.269722568	1.33E-08	0.084327741
0.255117716	-0.006860546	0.887197803	0.12109355	0.011971141	0.24006793
0.556034867	0.037814237	0.434138204	-0.110825445	0.021532189	-0.135520473
0.415258616	0.016167286	0.738154738	-0.016032483	0.740259125	-0.02897476
0.206106909	0.033133395	0.493181828	-0.033651933	0.486436843	-0.034717051
0.538305225	0.131605393	0.006276412	-0.107775396	0.025424835	-0.155818064
0.182330411	0.051372752	0.287832245	0.379351134	3.64E-16	0.278911686
0.276532757	0.011465864	0.812597122	0.0705181	0.144329716	0.208531896
0.179733196	-0.007565747	0.875692338	0.043920552	0.363591225	0.184776388
0.557201679	-0.149318392	0.00190445	-0.121666996	0.011570379	0.023619688
0.159330339	0.013091721	0.78662402	0.186305108	0.000101853	0.258197391
0.221286078	0.01030734	0.831232543	0.048038249	0.320312195	0.105797775
0.485116311	0.036727218	0.447475055	-0.036852593	0.445925061	0.083900421
0.238361523	0.139807426	0.003673127	0.140145288	0.003590773	0.134804514
0.044634283	-0.07109736	0.141053864	0.004384683	0.927763947	-0.055482459
0.660707278	0.078697908	0.103166474	0.155860121	0.001185221	0.195270458
0.414713587	0.033120717	0.493347359	-0.206463472	1.59E-05	-0.185109477
0.009344101	-0.081455959	0.091604455	-0.248736557	1.74E-07	-0.30697926
0.786608546	-0.151081133	0.001679022	0.051564201	0.286037136	0.146475246

0.033733521	0.084224162	0.081067352	-0.169307631	0.000421717	-0.31349417
0.28898125	0.039061517	0.419120841	-0.23944351	5.06E-07	-0.392218338
0.50692516	0.163815216	0.000649341	0.269333579	1.40E-08	0.131753404
0.586895597	0.024730779	0.609061343	-0.129645757	0.007103863	-0.114096412
6.72E-05	0.252067567	1.17E-07	0.135762752	0.004800728	0.056590628
0.648169124	0.364420243	5.96E-15	0.031548289	0.514106162	0.050070047
0.350102072	0.027529306	0.569148468	0.267967895	1.66E-08	0.258241171
0.447406387	0.053073119	0.272152907	-0.161757327	0.00076072	-0.363258065
0.020704904	0.088202325	0.067663547	0.100178179	0.037846047	0.07205634
0.075586143	0.154374202	0.001322172	0.165937874	0.000550443	0.140171894
0.289756008	0.025025097	0.6048058	-0.185305829	0.000111125	-0.033905652
0.502070364	-0.012347014	0.798493263	0.176186217	0.000241035	0.157275605
0.96983513	-0.0087558	0.85633689	-0.074320962	0.123854362	-0.174925348
0.432499322	0.134894387	0.005080196	-0.017391633	0.719132818	-0.061900516
0.888294469	0.219959307	4.14E-06	0.088656971	0.066254669	-0.076636122
0.816453996	-0.0371848	0.441832806	0.081092007	0.093067952	0.009404465
0.016370962	-0.011294516	0.815346958	-0.09253953	0.05517813	-0.101807775
0.850068586	0.059882873	0.215250916	0.221775316	3.43E-06	0.335739256
0.646366951	-0.16469213	0.00060664	-0.029648482	0.539777699	0.062527564
0.228507187	-0.294317478	4.86E-10	-0.280228299	3.36E-09	-0.225003506
0.031353265	0.084607851	0.079687489	0.020094723	0.677759759	-0.007822514
0.059544953	0.051502862	0.286611456	0.186866311	9.70E-05	0.403338068
0.000481968	0.076208027	0.114570501	0.063454942	0.189074024	0.151255679
0.78596198	0.1021964	0.034126771	0.07036086	0.145228893	0.187195123
0.860622971	0.330209809	2.13E-12	0.067896259	0.159887284	-0.056092516
0.016605266	0.032000659	0.508088057	-0.203184214	2.18E-05	-0.273887589
0.134424306	-0.062220768	0.197840111	-0.017987854	0.709931255	0.370715323
0.791377668	-0.04118739	0.394238873	0.208056857	1.36E-05	0.088310299
0.163056462	0.141181227	0.00334859	0.210890209	1.03E-05	0.35886026
0.042752816	0.001198119	0.980236497	-0.078668216	0.103296962	-0.227430436
0.271816261	-0.064978915	0.178647534	0.142309294	0.003101807	0.207891198
0.654625395	0.192590279	5.83E-05	-0.065498502	0.175191968	-0.33096568
0.09923826	0.116925402	0.015271095	-0.184527234	0.000118893	-0.184132473
0.705682247	0.038820142	0.422003014	-0.04749808	0.325790168	-0.123767412
0.234138507	0.073444226	0.128362033	-0.17741475	0.000217634	0.021469026
0.981638858	0.154455932	0.001314277	0.129038613	0.007379335	0.045781589
0.840750858	0.137596039	0.004255755	-0.009593917	0.842756244	-0.0403664
0.828552863	0.056636156	0.241215297	-0.043842997	0.364439855	-0.054125987
0.00586241	0.163022255	0.000690336	-0.048779788	0.312890395	-0.205043946
0.50778708	-0.156226686	0.001153507	-0.352329815	5.17E-14	-0.465234107
0.219828284	0.165141585	0.000585778	0.201678561	2.52E-05	0.277159487
0.562352148	-0.020831817	0.666637747	-0.212419982	8.88E-06	-0.081140555
0.0276821	-0.037991335	0.431987245	-0.215562042	6.48E-06	-0.414294145
0.074412769	-0.003961071	0.934726436	-0.154283373	0.001330998	-0.185359497
0.550958384	-0.085442278	0.076752286	-0.085013537	0.078249276	0.1855048
0.090879304	-0.055601878	0.249930131	-0.136270909	0.004643692	0.103394143
0.571192305	-0.078040791	0.106084997	-0.175706269	0.000250801	-0.301555287
0.039406002	0.015618371	0.746735844	-0.142692904	0.003021727	-0.254833294
0.640659536	-0.015263859	0.752294628	0.074714906	0.12186945	-0.064798749
0.548859191	0.079254859	0.100742961	0.1547936	0.001282113	0.203307404
0.60107494	0.117110867	0.015108828	0.027023509	0.576269341	0.019326518

0.563133919	0.017724385	0.713992317	0.143369773	0.002885	0.157000038
0.026740133	0.022060511	0.648260254	-0.01698892	0.725371084	-0.14983766
0.626552579	-0.055208543	0.253301217	0.002770996	0.954311439	0.098067978
0.01696356	0.059864855	0.21538924	0.124821493	0.009570876	0.122739412
0.987173775	0.063022285	0.192114023	-0.061810601	0.200818052	-0.386863834
0.387899026	0.0131163	0.786233107	0.110531473	0.021883396	0.079237479
0.062468253	0.11528908	0.016770259	-0.183140189	0.000134011	-0.229563727
0.179935973	0.155338471	0.001231737	0.149308312	0.001905815	0.035759846
0.00193354	0.031678871	0.512365171	-0.17748597	0.000216345	-0.229560537
0.663322264	0.019967986	0.679679252	-0.006015657	0.901013354	-0.007569563
0.195666607	0.017546984	0.716731294	0.008947529	0.853226274	0.010696093
0.342334329	0.0271249	0.574838557	-0.025339806	0.600270193	-0.005626164
0.866541832	0.03557809	0.461822941	0.357195563	2.19E-14	0.223991852
0.41493838	0.128210166	0.007770613	0.009107577	0.850631368	-0.060695222
0.032181051	0.141812994	0.00320827	-0.054296334	0.261240374	-0.068863362
0.768751516	-0.022524405	0.641376206	-0.061052197	0.206409899	-0.074690005
0.729349852	-0.001786375	0.970536629	0.394213286	1.94E-17	0.201395009
0.88846474	0.052049996	0.281516002	0.176608758	0.000232734	0.16606601
0.757854219	-0.017558833	0.716548231	0.021981961	0.6494289	0.151736953
0.909480796	-0.104438666	0.030364201	-0.058472874	0.226269159	-0.067246732
0.250065874	-0.109231332	0.023496868	0.026975112	0.576952892	0.118545326
0.688043719	0.012261899	0.799852848	-0.012101872	0.802410694	0.001321072
0.501140009	-0.006236059	0.897406299	0.010757994	0.8239717	-0.008470345
0.028601815	-0.021434315	0.657600431	-0.070688144	0.143362107	-0.305911247
0.007861237	0.088475224	0.066814957	-0.228340651	1.71E-06	-0.315283896
0.040216163	0.068094963	0.158665667	-0.104181206	0.030777344	-0.181658852
0.537115503	-0.037315049	0.440234209	-0.263878095	2.78E-08	-0.33855357
0.868302521	-0.041926261	0.385803883	-0.139976368	0.003631736	-0.153601235
0.602024188	0.102211317	0.034100483	-0.076109314	0.115042218	-0.132170234
0.540222434	-0.024677456	0.609833752	0.150804889	0.001712651	0.06633857
0.01137785	0.028749875	0.552139208	-0.073090423	0.130216669	-0.022655848
0.17431631	-0.071524107	0.138677171	-0.06079401	0.20833903	-0.225912234
0.038103859	-0.00533421	0.912178325	0.008588619	0.859051064	0.180111214
0.412962061	-0.01802002	0.709436025	0.108125502	0.024949176	0.206573669
0.365059183	0.024748504	0.608804676	0.094342965	0.05058247	0.266909234
0.007824904	-0.00658839	0.891644554	-0.040771814	0.3990316	-0.236572086
0.977432293	0.227590847	1.86E-06	0.043605437	0.367046982	0.003532242
0.18680265	-0.016226512	0.737230784	0.140553529	0.003493499	0.037244225
0.129544639	-0.044693153	0.355204579	-0.271970217	9.94E-09	-0.458907844
0.311628778	-0.030474672	0.528535505	-0.101362221	0.035624486	-0.131142774
0.854421732	0.023490092	0.627144392	0.022414178	0.643009181	-0.006969049
0.104054818	-0.003554701	0.941410221	-0.195290036	4.56E-05	-0.425635093
0.893513013	-0.003582638	0.940950593	-0.050645209	0.294722957	-0.062886818
0.959501412	0.106663207	0.026988101	-0.003537815	0.941688044	-0.12252036
0.266111967	-0.012993712	0.788183346	0.006446888	0.893957897	0.205686726
0.582587827	-0.027071832	0.575587221	-0.044845902	0.353560994	-0.099065113
0.124224454	0.018271593	0.705566943	-0.344231495	2.09E-13	-0.387533106
0.878618676	0.092076354	0.056412858	0.10783214	0.025347216	0.257045544
0.253150485	0.103116086	0.032538021	0.077220266	0.109820439	-0.128578125
0.030947759	0.030418262	0.529299301	0.062157611	0.19829654	0.333665995
0.332311407	0.250669343	1.39E-07	-0.049220002	0.308538259	-0.138856071

0.733731458	-0.084848525	0.078831704	0.039999607	0.408029513	0.140161366
0.020168245	0.115105493	0.016946237	0.211074878	1.01E-05	0.0428118
0.911939296	-0.008446722	0.861356054	-0.25007503	1.49E-07	-0.190441295
0.912677657	0.168698163	0.000442697	0.396991011	1.10E-17	0.240404572
0.277214928	0.006944175	0.885832087	-0.049373063	0.307034425	-0.03296707
0.619812083	0.059380239	0.219133623	-0.335529175	8.96E-13	-0.437560703
0.434692507	0.021145078	0.661932793	-0.173166353	0.000308932	-0.321494688
0.222783098	-0.073098046	0.130176494	-0.043855472	0.364303267	-0.005059352
0.97109785	0.00535813	0.91178611	0.07185291	0.136867021	-0.197887529
0.570117312	0.176139764	0.000241965	0.021017505	0.663847257	0.008944175
0.786105634	0.051160104	0.289834987	0.023140321	0.632283555	0.091239924
0.506834455	0.186812877	9.74E-05	0.080872295	0.093960452	0.078561445
0.102539621	0.016065739	0.739739795	-0.188693968	8.26E-05	-0.306369569
0.022588157	0.08347755	0.083807769	0.035670197	0.460663546	0.087736507
0.720889353	0.065877539	0.172702666	-0.137896236	0.004172046	-0.303771217
0.503864658	0.067798834	0.160488835	0.391985552	3.04E-17	0.221671298
0.224238228	-0.036684823	0.447999866	-0.127744886	0.007998397	-0.03565244
0.965551172	0.32709709	3.52E-12	0.083178296	0.084926948	0.063342286
0.666704788	-0.033061135	0.494125702	-0.015832027	0.743392004	0.053384759
0.770927242	0.020708201	0.66849799	0.224387231	2.61E-06	0.252021213
0.176637315	0.176212985	0.000240501	0.21079668	1.04E-05	0.356622304
0.085952465	-0.015509343	0.748444034	0.020852986	0.666319384	-0.019282002
0.83482774	0.003012995	0.950326258	-0.028593169	0.554308996	-0.061462244
0.201286085	0.021169725	0.661563175	-0.021531929	0.656140893	0.225792801
0.569989579	0.041476979	0.39091975	0.083975293	0.081972649	0.099563246
0.035060076	0.088035047	0.068188057	0.029967801	0.535418524	0.133701483
0.98398405	0.050418621	0.296891339	-0.067111168	0.164783368	-0.198706166
0.416642526	0.294745733	4.57E-10	0.09553438	0.047725242	0.051208458
0.852852275	-0.029139751	0.546758975	-0.075151739	0.119697511	0.123903875
0.043519247	0.133442776	0.005580235	-0.071059284	0.141267427	-0.146180595
0.391568575	0.009764744	0.839993772	-0.235850772	7.56E-07	-0.050866425
5.36E-06	0.112740005	0.019362296	0.049797105	0.302893498	-0.086648824
0.047531622	-0.056049237	0.246134148	0.018252096	0.705866533	-0.000465436
0.413661052	-0.047572795	0.325028862	0.024259217	0.615907159	0.193711307
0.490653568	0.053297642	0.270126941	0.018073939	0.708606143	0.078705399
0.524252488	0.009869417	0.838302061	0.171309885	0.000359126	0.063344444
0.453342089	-0.025097184	0.603765526	-0.014401976	0.765862026	-0.023096802
0.273528101	0.080124367	0.097050009	0.020676841	0.66897023	-0.101167108
0.955925855	0.182613299	0.000140213	-0.170068423	0.000396826	-0.154348731
0.341760506	0.124495456	0.009762256	0.199242393	3.16E-05	0.081425412
0.463354006	0.020939649	0.665016695	0.052021621	0.281778746	0.183655477
0.473516974	0.083989898	0.081919297	0.039304195	0.41623476	0.338955523
0.054081632	0.00871053	0.857071676	0.319108042	1.24E-11	0.239994815
0.199808012	-0.004962264	0.918279811	0.022856749	0.636463092	0.035940442
0.118367068	-0.019700239	0.683741203	-0.224495164	2.58E-06	-0.364017228
0.92646001	0.092114397	0.056310586	0.112786245	0.019312334	0.013029605
0.018165885	0.24030984	4.59E-07	0.394611729	1.79E-17	0.10216557
0.391383401	-0.003703113	0.938968714	-0.084184413	0.0812114	0.093088268
0.12720635	0.099490523	0.039189372	-0.017492225	0.717577485	-0.024668776
0.004814692	0.066424104	0.169159679	-0.031389383	0.516228901	-0.115005919
0.904434258	0.022429236	0.642786007	0.291800199	6.91E-10	0.089181869

0.442288163	0.020249345	0.675420759	-0.16786232	0.000473051	-0.110246399
0.24910062	0.038523781	0.425557551	0.087956143	0.068436621	0.134230083
0.307995689	0.098509384	0.041175708	0.214471208	7.23E-06	0.150470411
0.174385899	0.068851443	0.154079236	0.012542607	0.795371265	-0.056874545
0.768721537	0.027698839	0.566771095	0.363112374	7.56E-15	0.27801726
0.284630825	0.07365802	0.127251286	0.153331123	0.001426844	0.134250801
0.207867417	-0.039205602	0.417405873	-0.048665848	0.314023386	-0.086281724
0.127635784	0.002956502	0.951256464	0.107934645	0.025207517	0.165530385
0.002094024	0.067029931	0.165296338	-0.04006838	0.407223304	-0.121122739
0.527575912	-0.011848876	0.80645882	0.082280896	0.088355495	-0.010060047
0.120028231	0.069924294	0.147747817	-0.079870556	0.098116664	-0.004715537
0.555698274	0.092907133	0.054214229	0.042210462	0.382588901	-0.007184412
0.50265933	0.136089861	0.004699102	-0.015658636	0.746105311	-0.169212715
0.317239874	0.029227656	0.545549478	-0.152003941	0.001571022	-0.286659916
0.67432899	0.034398707	0.476811003	0.011324883	0.814859444	-0.061178301
0.345765729	0.076552845	0.112934629	0.154471525	0.001312775	0.043935936
0.63388946	0.08691011	0.071802503	0.016272987	0.736506014	0.140716775
0.00036417	0.04164406	0.389012455	0.083053564	0.085396974	-0.257365782
0.944856006	-0.041658177	0.388851571	-0.058081987	0.229393479	0.080279906
0.114751525	-0.095781685	0.047149423	-0.047440048	0.32638227	0.101937606
0.012564916	0.013092974	0.786604094	-0.164918582	0.000596045	-0.262585524
0.34466084	-0.026924178	0.577672691	-0.090451134	0.060927962	-0.198388058
0.810853594	0.058372459	0.227068863	0.214199616	7.43E-06	0.243542763
0.496087821	-0.039374246	0.415403851	0.066752166	0.167059346	0.023053791
0.115044007	-0.120110219	0.012686959	-0.121141037	0.011937495	-0.116971969
0.091363987	-0.01231273	0.79904083	0.015182782	0.753567735	0.203887951
0.108904068	-0.019484513	0.687020617	-0.08559255	0.076233136	-0.120444832
0.217010145	0.090152492	0.061789247	0.1510245	0.001685866	0.153159036
0.312814476	0.081982946	0.089518106	-0.120610368	0.01231829	-0.219306718
0.727919418	-0.07119179	0.140525284	0.030641496	0.526279985	-0.060553581
0.727319571	-0.056721302	0.24050747	-0.156964085	0.001092066	-0.101573042
0.000270638	0.015229551	0.752833271	-0.04501881	0.351706288	-0.138361891
0.649106492	0.152506169	0.001514959	0.236552955	6.99E-07	0.125826974
0.795767667	0.115516548	0.016554438	-0.069438972	0.150586955	-0.012771635
0.014295507	0.014691918	0.761289656	-0.206235496	1.63E-05	-0.327867005
0.431900334	0.022819394	0.63701454	-0.171859663	0.000343521	-0.155565514
0.856216054	0.04130848	0.392848938	0.058163498	0.228739458	-0.016266267
0.825369866	0.151436861	0.001636608	-0.012709699	0.792706765	-0.107377533
0.874796899	-0.011508523	0.811912876	0.226309142	2.13E-06	0.467897456
0.668310883	0.010241994	0.832286599	-0.040833754	0.398315054	0.10297843

MEturquoise	MEblack	MEblack	MEblue	MEblue
2.93E-09	0.005850527	0.903717167	0.116834112	0.015351527
2.46E-05	0.058475892	0.226245161	0.071921067	0.13649408
3.68E-10	-0.040519462	0.401958874	0.450444218	7.12E-23
0.102085923	0.109564855	0.023073451	0.059651691	0.217030554
0.618763456	0.287964664	1.18E-09	-0.310529609	4.58E-11
0.009565406	0.017491159	0.717593955	0.201017019	2.68E-05
2.04E-11	0.020078076	0.678011776	-0.159666563	0.000891767
3.17E-09	0.060700942	0.209037602	0.243372117	3.24E-07
0.516190937	-0.061700759	0.201621044	-0.142746249	0.003010741
2.45E-10	0.008450703	0.861291377	-0.29037021	8.44E-10
0.00789849	-0.028375295	0.557332636	-0.340793756	3.73E-13
0.096689686	-0.167889593	0.000472031	0.13185931	0.00617578
0.008114647	0.03911731	0.418456279	-0.035599256	0.461556373
0.026096193	0.068357434	0.157062817	-0.24313094	3.33E-07
0.178004124	0.104503937	0.030260218	0.05937741	0.219155623
0.012957774	0.044797108	0.354085497	0.080173692	0.096843792
7.25E-10	-0.236957768	6.68E-07	0.075587974	0.117558815
1.90E-05	0.189369498	7.78E-05	-0.243684452	3.12E-07
3.35E-08	0.130682165	0.00665482	0.207185638	1.49E-05
2.48E-10	0.211326382	9.90E-06	-0.11887547	0.013639088
0.342215172	-0.032696285	0.498906165	-0.425276599	2.58E-20
0.730172758	0.258146636	5.64E-08	-0.19035857	7.12E-05
0.563458787	0.026724986	0.580491671	-0.261978482	3.52E-08
0.122724409	0.100120059	0.037958053	-0.088442292	0.066916896
0.040053884	-0.018239495	0.706060181	-0.261648764	3.67E-08
1.32E-06	-0.091743907	0.057313148	0.078707168	0.103125806
0.398058521	-0.009131929	0.850236695	0.245262078	2.61E-07
8.45E-05	0.062160205	0.198277784	0.228915645	1.61E-06
1.76E-10	0.041426008	0.391502725	0.246601814	2.23E-07
0.000182531	0.115046419	0.017003205	-0.053986655	0.263974149
3.37E-05	-0.108423946	0.024549788	0.066375035	0.169475519
6.83E-14	0.080268199	0.096449661	0.247145335	2.09E-07
0.001247325	0.014470954	0.7647735	-0.310990244	4.27E-11
0.552150598	0.032785701	0.497732332	0.230220673	1.40E-06
0.383990217	-0.03315844	0.492854899	0.259281766	4.91E-08
0.368815969	0.400287902	5.61E-18	-0.038013853	0.431714188
0.674237387	0.075865302	0.116214796	0.5114365	4.93E-30
1.80E-08	0.060386285	0.211411949	0.257283718	6.27E-08
0.083877286	0.349696981	8.18E-14	0.045402902	0.347608318
4.39E-05	0.458519151	9.66E-24	-0.04407487	0.361906308
0.121960084	-0.337096005	6.92E-13	-0.06991405	0.147807324
2.63E-11	0.263927143	2.76E-08	0.002465194	0.959348934
1.27E-09	0.232696649	1.07E-06	0.111270035	0.021010332
0.018805841	0.306956805	7.80E-11	-0.143681071	0.002824027
3.02E-13	0.079637882	0.09910267	0.26584467	2.17E-08
0.045369331	0.103506346	0.031883178	-0.356484545	2.49E-14
0.563277814	0.014728125	0.76071925	-0.148038547	0.002085123
1.32E-09	0.132257353	0.006020938	-0.010570375	0.826992799
0.098035011	0.107075931	0.026398596	-0.127822903	0.007959792
0.911856028	0.40296175	3.22E-18	-0.057310535	0.235649098

0.112438835	0.03493734	0.469933124	-0.091396241	0.058267355
0.724680222	0.154637207	0.001296919	-0.327252122	3.43E-12
0.076501178	-0.031873063	0.509781799	0.192914659	5.66E-05
0.20107155	0.146812222	0.002272805	-0.256400846	6.98E-08
0.006252929	-0.125552511	0.009153847	-0.138922979	0.003896875
0.229453722	-0.01244479	0.796932179	-0.22535063	2.36E-06
0.047643201	0.117864757	0.014464711	0.355377505	3.03E-14
9.99E-08	-0.200924736	2.70E-05	0.107641787	0.025608409
0.000199088	0.068978634	0.153318057	-0.255071974	8.19E-08
0.238429519	0.037718266	0.435306391	-0.114563067	0.017475648
0.91617645	-0.116960265	0.015240477	0.357927444	1.92E-14
0.028041618	0.112458524	0.019668867	-0.157426038	0.001055126
0.00200295	0.164230056	0.000628805	-0.05431627	0.261065058
0.034756105	0.287269959	1.30E-09	-0.030728177	0.525109969
0.100532868	0.25664439	6.77E-08	-0.14994839	0.001820881
0.208504935	0.121537772	0.011659639	-0.26319067	3.03E-08
0.173305809	0.223217117	2.95E-06	-0.028816998	0.551211079
9.36E-06	0.129140618	0.007332396	0.255211003	8.06E-08
0.24224712	0.098327875	0.04155234	-0.159209199	0.000923084
0.103485842	0.294968456	4.43E-10	-0.179055618	0.000189683
0.287701582	0.007710288	0.873337264	-0.192114998	6.08E-05
3.98E-20	0.169361094	0.000419921	-0.160015296	0.000868552
0.083391645	0.062079919	0.198859071	-0.197171059	3.83E-05
0.005809937	0.034715749	0.472756012	-0.30632869	8.56E-11
0.351843589	-0.010634296	0.825963228	0.257768375	5.91E-08
0.58664926	0.133807331	0.005450659	-0.125784122	0.009025132
0.060676672	0.165088608	0.000588202	-0.18373171	0.000127355
2.16E-05	0.103630248	0.031677643	-0.154095143	0.001349461
2.72E-05	0.284018124	2.02E-09	0.045025988	0.351629426
0.463008965	0.270927186	1.14E-08	-0.056728612	0.240446767
0.00064079	0.0476369	0.324376599	-0.065118067	0.177717171
0.00018808	0.106783128	0.026815659	-0.016770303	0.728765224
0.415659145	0.153416241	0.001418025	-0.14630707	0.002354504
0.603916317	0.226404216	2.11E-06	-0.258955259	5.11E-08
0.470414918	0.034949119	0.469783328	-0.210420144	1.08E-05
0.002471943	0.008248946	0.864570717	-0.048171035	0.318974819
0.141683959	0.108853166	0.023985074	-0.0346937	0.473037413
0.970542979	0.162196395	0.000735569	-0.053877838	0.26493941
0.871569779	0.266680582	1.96E-08	-0.033325372	0.490678837
0.000664783	0.341813685	3.15E-13	-0.120975489	0.012055156
2.87E-06	-0.019378531	0.688633878	0.282749759	2.40E-09
9.14E-09	-0.015324426	0.75134401	0.31208011	3.63E-11
0.009188776	-0.038323823	0.42796564	-0.129701896	0.007078862
0.180565084	0.072934649	0.131039778	-0.212377084	8.92E-06
0.014795395	-0.25335813	1.01E-07	-0.036851454	0.445939131
0.693138065	0.013976601	0.772584859	-0.379141444	3.78E-16
0.212462626	0.003237496	0.946630342	-0.012588275	0.79464278
1.43E-08	0.197215714	3.82E-05	-0.035600566	0.461539883
0.921230347	0.177594255	0.000214399	-0.319751632	1.12E-11
9.88E-06	-0.201866321	2.47E-05	0.291927445	6.79E-10
7.91E-09	-0.001047266	0.982724464	0.217819569	5.15E-06

1.47E-05	0.315635653	2.11E-11	-0.158900212	0.000944812
0.001522134	0.05539922	0.251663093	0.375351105	7.80E-16
0.473566724	0.01945553	0.687461645	-0.097067746	0.044248065
0.00184003	0.344618658	1.96E-13	-0.111270943	0.021009278
0.171934076	-0.041587279	0.389659998	-0.090344199	0.061235219
7.48E-13	0.016898339	0.726776758	0.26990748	1.30E-08
0.013857164	0.326785685	3.70E-12	0.168106224	0.000464
0.036182562	-0.104574378	0.030148342	0.013113969	0.78627018
0.073459474	0.264185377	2.68E-08	0.029417573	0.542940929
4.82E-11	0.155116238	0.00125206	0.090752246	0.060069613
0.004343049	-0.108354932	0.024641651	0.193935881	5.16E-05
0.254991399	0.056341987	0.243671982	-0.171357504	0.000357749
0.002084654	0.118784756	0.013711457	-0.055835315	0.247944295
0.008285944	-0.171514381	0.000353246	0.001299495	0.978564646
0.92109502	0.01856177	0.701113557	0.054245199	0.261690432
0.506981394	0.077029285	0.110704568	-0.267353812	1.80E-08
1.99E-09	0.032854208	0.496833968	0.137974214	0.004150547
0.572254726	-0.005829442	0.904062489	-0.477499701	7.13E-26
8.69E-06	0.163639039	0.000658249	0.097022455	0.044347632
7.41E-08	0.019513706	0.686576486	-0.117205994	0.015026189
0.632697786	0.016931258	0.726265793	-0.367083284	3.66E-15
1.75E-09	0.008512287	0.860290857	-0.000479602	0.992088075
0.008815102	0.374350165	9.43E-16	-0.110515732	0.021902341
0.362126641	0.520540791	3.13E-31	-0.141236401	0.003336117
0.647855079	0.225537585	2.31E-06	-0.167989516	0.000468311
2.87E-10	0.090980765	0.059424888	0.04486693	0.353335103
0.013907803	-0.086944759	0.071688886	0.047999914	0.320698966
0.285979659	0.201191736	2.64E-05	-0.044324946	0.359186209
0.000283297	0.103809884	0.031381666	-0.321329473	8.75E-12
3.32E-07	0.24946111	1.60E-07	-0.056055537	0.246080982
0.000990555	-0.071012866	0.141528116	-0.006840215	0.887529864
0.131824549	-0.001337144	0.977943772	0.236675204	6.90E-07
1.46E-05	0.046058719	0.340681528	0.335539727	8.94E-13
0.432116562	0.037259853	0.440911248	-0.227707528	1.83E-06
1.59E-05	0.099507453	0.039155822	-0.114551667	0.017486928
0.028360128	0.072718866	0.132186613	-0.395774984	1.41E-17
5.67E-09	-0.303397449	1.32E-10	-0.211568075	9.66E-06
0.888216966	-0.155441322	0.001222435	-0.139155361	0.003836922
1.59E-11	0.029288403	0.544714431	-0.142551717	0.003050979
0.003245055	0.237337193	6.41E-07	-0.182522364	0.00014131
0.588656914	-0.245134061	2.64E-07	0.101410229	0.0355368
3.05E-06	-0.014456497	0.765001616	-0.142117324	0.0031426
0.001300757	0.043877077	0.364066798	0.090427191	0.060996648
1.32E-12	0.005793753	0.90464705	0.07997766	0.09766542
0.660348939	0.194025627	5.12E-05	-0.32622582	4.04E-12
0.019381336	0.368866885	2.63E-15	0.010739108	0.824275692
0.02067427	-0.163206665	0.000680594	-0.052244909	0.279715668
0.441831787	-0.131002674	0.006521196	0.088499368	0.066740305
4.05E-12	0.225862056	2.23E-06	0.25392588	9.40E-08
0.022026737	-0.076462759	0.113360232	0.325689009	4.40E-12
0.014220029	0.064634046	0.18096886	-0.31196245	3.69E-11

0.797564568	0.291903275	6.82E-10	-0.093435897	0.052852336
0.188328072	0.25363839	9.73E-08	0.088253941	0.067502371
1.53E-07	0.073948155	0.125755845	0.323620712	6.11E-12
0.084868126	-0.038206646	0.429380469	0.198094004	3.52E-05
0.247671851	0.143721366	0.002816221	-0.258982995	5.09E-08
6.56E-05	-0.275635774	6.17E-09	-0.006611119	0.891273055
9.09E-05	-0.146236211	0.002366177	0.238244433	5.79E-07
0.018899539	0.397778642	9.39E-18	-0.137489742	0.004285757
0.142135676	0.440982909	6.91E-22	-0.083779639	0.08269009
0.548475823	0.180411646	0.00016916	-0.028854188	0.550697165
0.01640063	-0.156451271	0.001134465	0.00401726	0.933802617
0.00761816	0.387063424	8.09E-17	0.015386182	0.75037512
8.20E-06	0.091988915	0.056648503	-0.113281947	0.018783747
0.071970814	-0.092954761	0.054090375	0.112939827	0.019147195
0.367259587	0.092754876	0.054611755	-0.165797059	0.000556544
4.86E-12	0.022496569	0.641788415	0.079309256	0.100508702
0.147943357	-0.337425207	6.55E-13	-0.132169391	0.006054853
0.036879772	-0.10712376	0.026331002	0.096106868	0.046401148
0.306171695	0.251829086	1.21E-07	-0.181378594	0.000155817
0.474365609	0.266808719	1.92E-08	-0.068590345	0.155650754
3.63E-05	0.022751625	0.638015455	0.186658863	9.87E-05
0.739310538	-0.14956157	0.001871785	-0.068411967	0.156731337
0.015547649	0.077538378	0.10836012	-0.115788967	0.01629917
0.000630215	0.028495745	0.555660045	-0.021755071	0.652809343
4.35E-07	0.054887353	0.256077282	-0.06787074	0.160044686
8.70E-06	0.145625493	0.002468985	-0.05657164	0.241752593
0.003996419	0.21063956	1.06E-05	-0.09262123	0.054962681
6.91E-07	0.029265122	0.545034383	0.10257791	0.03345987
0.000667557	0.217079678	5.56E-06	0.374883579	8.52E-16
0.022610302	-0.026395372	0.585170462	-0.087337993	0.070409693
0.114971033	0.050044047	0.300499152	-0.368388479	2.87E-15
0.059835747	0.079257257	0.100732623	-0.164526215	0.000614514
1.39E-13	0.019160409	0.691958578	-0.42453305	3.05E-20
2.55E-10	0.00203845	0.966381358	0.255696114	7.60E-08
0.012982895	0.084342575	0.080639456	0.186634365	9.90E-05
6.77E-09	0.137678328	0.004232659	-0.092333461	0.055724665
0.00052048	-0.000283935	0.995315923	-0.360409984	1.23E-14
0.018594727	0.122258275	0.011169621	0.018582855	0.700790355
0.717971891	0.305584931	9.56E-11	-0.165228711	0.000581812
8.07E-05	0.336341594	7.84E-13	0.010109966	0.83441716
0.419524792	0.095147549	0.048637734	0.065780986	0.173334258
0.090916612	0.128202802	0.007774172	-0.003379253	0.944297209
0.978484064	-0.283943757	2.04E-09	-0.090124025	0.061871868
1.67E-06	0.077616217	0.108005141	0.330072752	2.18E-12
1.42E-05	0.004555618	0.924955999	0.169763696	0.000406627
4.98E-14	0.106634883	0.027028968	-0.041807995	0.387146584
0.028398223	0.444217638	3.20E-22	-0.146306222	0.002354644
0.054368347	0.223368344	2.90E-06	-0.103148186	0.032483729
5.73E-05	0.129175988	0.007316183	0.026506776	0.583587163
0.593456072	0.291072496	7.65E-10	-0.077668212	0.107768528
2.59E-09	-0.087462935	0.070007186	-0.287918975	1.18E-09

0.722985729	0.005845851	0.903793754	-0.162621709	0.000711942
0.981965131	0.158289253	0.000989171	0.029820196	0.537431354
0.52131576	0.040866213	0.397939863	-0.356816953	2.34E-14
0.035657581	0.122279011	0.011155792	-0.109254589	0.023467126
1.37E-15	0.216565867	5.85E-06	0.235255554	8.07E-07
0.954852046	-0.13476569	0.005122839	0.036686154	0.447983379
0.51809943	-0.116598808	0.015560557	-0.245122287	2.65E-07
1.26E-10	-0.007494413	0.876855014	0.431819108	5.84E-21
0.002068829	0.209306757	1.21E-05	0.288386318	1.11E-09
0.076117456	0.196490889	4.08E-05	-0.111607211	0.020621913
0.290163035	0.229095349	1.58E-06	0.211683498	9.55E-06
0.001301564	0.12219402	0.011212568	-0.267979851	1.66E-08
0.002323152	-0.153610662	0.00139807	0.09826037	0.041693153
0.097882476	0.25132947	1.28E-07	-0.230222637	1.40E-06
0.372582221	0.06457353	0.181378485	-0.401556661	4.31E-18
0.00019783	0.131837446	0.006184388	0.212700573	8.63E-06
0.453683841	0.046076625	0.34049365	-0.020348237	0.673926431
1.41E-05	-0.006021813	0.900912578	0.308835648	5.90E-11
0.180698491	-0.266570014	1.98E-08	0.130154591	0.00688012
6.60E-13	-0.084173879	0.081249608	0.024003762	0.619629693
1.63E-05	0.28646388	1.45E-09	0.175850484	0.000247828
0.481768317	0.03876166	0.422703069	0.014066184	0.771167625
0.115807274	0.036949977	0.44472322	-0.279114017	3.90E-09
0.182964319	0.375060667	8.24E-16	-0.161368238	0.00078367
0.772899093	0.058135041	0.228967639	-0.218077359	5.02E-06
1.33E-17	0.020874229	0.665999979	-0.324612731	5.22E-12
0.0033509	-0.133325466	0.005622516	0.261592279	3.69E-08
0.821741082	0.056707564	0.240621581	-0.092098741	0.056352655
1.82E-19	0.096715763	0.045026811	-0.278153982	4.43E-09
4.11E-10	0.182569996	0.000140734	0.012601616	0.794430011
0.644239441	0.014593572	0.762839643	-0.394396519	1.87E-17
0.084331891	0.036899408	0.445347074	-0.143040215	0.002950851
0.008306518	0.079079056	0.101503017	-0.09840967	0.041382256
0.904291949	0.002563246	0.957733531	0.17570616	0.000250803
0.013616275	0.026568757	0.582707125	0.23561779	7.76E-07
8.81E-07	0.301147213	1.83E-10	-0.144611633	0.002648687
0.790752177	0.139037479	0.00386723	0.065162695	0.177419559
0.883897089	-0.281288583	2.92E-09	-0.228817565	1.63E-06
0.000159664	0.098698763	0.04078582	0.244734241	2.77E-07
5.18E-13	0.316110732	1.96E-11	0.153810713	0.001377807
1.61E-09	0.026044384	0.590171765	0.049633407	0.304487669
3.97E-09	0.154707593	0.001290236	-0.05316876	0.271288637
0.010153158	0.371283616	1.68E-15	-0.047215662	0.328678244
9.29E-06	-0.155941341	0.001178126	0.202804651	2.26E-05
0.000133773	0.013197849	0.784936486	-0.217082399	5.55E-06
0.099107258	0.098052157	0.042130015	0.175696677	0.000251
2.97E-08	0.146257052	0.002362738	-0.349866764	7.94E-14
2.46E-05	-0.021011281	0.663940713	-0.083030877	0.085482689
0.687265755	0.012270844	0.79970994	-0.24675973	2.19E-07
0.00269766	0.235250906	8.08E-07	0.069547461	0.149948728
0.758411813	-0.233376311	9.93E-07	-0.123127145	0.010603006

0.037440256	0.146431063	0.002334206	-0.09385273	0.051798923
0.497319775	0.070526746	0.144280394	0.348735452	9.66E-14
0.010962143	0.067781824	0.160594034	0.243179613	3.31E-07
0.729547633	0.087749852	0.069089983	0.433118141	4.33E-21
0.814682764	0.043462781	0.368618119	-0.471182855	3.78E-25
0.178304104	0.009352102	0.846669953	-0.242757635	3.47E-07
1.22E-06	-0.165269123	0.000579981	-0.070842595	0.142487536
0.001041938	0.142065274	0.003153744	-0.049902519	0.301869868
0.410033244	0.104760635	0.02985423	-0.280659399	3.17E-09
0.099137054	0.306188501	8.74E-11	-0.049929995	0.301603429
8.63E-09	0.095355637	0.048145078	-0.13526028	0.0049607
0.328492888	-0.037725785	0.435214805	-0.08273344	0.086612889
3.48E-10	-0.139383319	0.003778923	0.204998477	1.83E-05
0.040816973	0.051797498	0.283859848	0.009933824	0.837261491
7.16E-05	-0.011921989	0.805288429	-0.167879108	0.000472423
0.020295654	0.048763782	0.313049395	-0.349444576	8.54E-14
9.44E-07	-0.021026704	0.663709137	-0.37763153	5.05E-16
0.019036493	0.086540792	0.073022688	-0.334600621	1.04E-12
1.64E-07	0.071462417	0.13901883	0.319042181	1.25E-11
0.464475001	-0.229898714	1.45E-06	0.151931136	0.001579305
0.397408327	0.11603846	0.016068415	0.013612678	0.778350033
6.96E-11	-0.430528438	7.85E-21	-0.034765266	0.472124406
0.067691393	0.205353012	1.77E-05	-0.096279685	0.046007552
0.00947656	0.060300761	0.212060643	-0.102267674	0.034001326
3.03E-14	0.212863123	8.50E-06	0.076949567	0.111075269
0.965988384	0.072225157	0.134839679	-0.330274337	2.11E-12
0.552320122	0.10022977	0.037746859	0.098277626	0.041657119
0.000107131	0.101111379	0.03608564	0.17185986	0.000343516
0.525143407	0.043120436	0.372405509	0.145605754	0.002472375
7.96E-06	0.073944447	0.125774873	0.195255816	4.57E-05
0.010058821	-0.008116778	0.866720235	0.302241485	1.56E-10
0.522143279	0.169557061	0.000413401	-0.048349996	0.317178167
6.89E-07	0.025589998	0.596675377	0.137050751	0.004411698
0.001701969	0.090006118	0.062215043	-0.083779024	0.082692353
0.231001075	0.057455232	0.234466694	-0.019989068	0.679359817
0.513850133	-0.001154935	0.9809487	-0.136962595	0.004437388
0.824483361	0.274720808	6.96E-09	-0.289162873	9.98E-10
0.016904008	0.083985574	0.081935087	0.209910831	1.14E-05
1.59E-14	0.261201753	3.88E-08	0.018945185	0.695244921
1.06E-08	0.139398789	0.003775016	-0.062174945	0.198171198
2.34E-11	0.139938528	0.00364097	0.450662432	6.75E-23
0.298096876	0.014565325	0.763285018	-0.335255465	9.37E-13
1.34E-08	-0.159305375	0.000916415	-0.077157465	0.110110557
9.09E-17	-0.064681078	0.180650983	0.0742587	0.124170364
0.000163152	0.035976943	0.456814112	0.194367956	4.96E-05
0.131363614	0.301927796	1.63E-10	-0.200898812	2.71E-05
0.79625299	0.126026063	0.008892399	0.218291276	4.91E-06
8.05E-05	0.035976818	0.456815685	-0.163145768	0.000683797
6.18E-10	0.328820809	2.67E-12	-0.102918792	0.03287342
0.508984863	0.384846643	1.25E-16	-0.181146688	0.000158924
0.184514348	-0.058857193	0.223226957	0.072886014	0.13129759

0.001201931	0.195466339	4.49E-05	-0.31937721	1.19E-11
0.01741225	0.198730535	3.32E-05	-0.090441944	0.06095432
7.98E-06	0.092488285	0.055313624	-0.003530957	0.941800884
0.009187972	0.05771339	0.232367548	0.232637094	1.08E-06
0.000379131	0.049827736	0.302595821	0.027255894	0.572992499
7.74E-06	0.226704752	2.04E-06	0.218628775	4.74E-06
1.41E-07	-0.211654591	9.58E-06	-0.159699497	0.000889551
9.13E-06	0.283552503	2.15E-09	-0.2705467	1.19E-08
0.761216467	0.418805033	1.09E-19	-0.112561091	0.019556672
0.553892504	0.123102303	0.010618845	-0.162630713	0.00071145
2.77E-21	-0.075760928	0.11671921	-0.317056705	1.70E-11
7.97E-05	0.226888125	2.00E-06	0.106339535	0.027458299
6.99E-07	-0.140025597	0.003619754	0.074406056	0.123423496
0.002678023	0.157650513	0.001037596	0.482561533	1.83E-26
2.72E-07	-0.130557059	0.00670764	-0.189802999	7.48E-05
0.399971427	0.0801604	0.096899329	0.259590509	4.73E-08
6.81E-18	0.29686851	3.38E-10	0.054368759	0.260603851
0.007143606	0.032905483	0.49616215	0.286883656	1.37E-09
0.016214305	0.333601468	1.23E-12	-0.177800707	0.000210733
0.395477251	-0.002994558	0.950629842	-0.38787801	6.89E-17
6.63E-17	-0.167898702	0.000471691	0.131052243	0.006500746
8.07E-10	0.505047384	3.25E-29	0.096795029	0.044850441
0.026860437	-0.228812332	1.63E-06	-0.098109858	0.042008566
0.044385419	-0.14355782	0.002848026	0.196385661	4.12E-05
2.14E-06	0.01270635	0.792760143	-0.14410021	0.002743784
5.05E-16	0.158681721	0.00096046	0.056389278	0.243275858
0.099899988	-0.110561355	0.02184747	0.107072155	0.026403938
0.002792345	0.125531834	0.009165417	-0.182821486	0.00013773
1.30E-07	-0.028429181	0.556584062	0.137930128	0.00416269
1.08E-06	0.121147758	0.011932739	0.311992752	3.67E-11
0.054637829	0.148645007	0.001997642	-0.133081286	0.005711448
0.001017631	-0.081800233	0.090237109	0.196880258	3.94E-05
0.577043338	0.026864192	0.578520934	-0.094180014	0.050984137
0.000163475	0.219795243	4.21E-06	-0.264176242	2.68E-08
4.51E-05	0.238773328	5.46E-07	0.17529179	0.00025953
0.977914382	0.055523041	0.250603291	-0.176857151	0.000227979
0.393233697	0.45823531	1.04E-23	-0.101361438	0.035625916
2.63E-11	0.042559519	0.378662711	-0.251793654	1.21E-07
3.62E-06	0.062905932	0.192937638	-0.237982399	5.96E-07
0.000269111	0.164037763	0.000638248	-0.113081864	0.018995566
0.205197226	0.213904206	7.66E-06	-0.374569279	9.05E-16
0.019597586	0.035298209	0.465355874	-0.298493251	2.68E-10
0.000144597	0.249837482	1.53E-07	-0.379074757	3.83E-16
6.85E-24	0.034087869	0.480804993	0.338180219	5.78E-13
0.64590809	-0.039557801	0.413231259	-0.066971593	0.165665455
0.00446827	-0.01597655	0.741132857	-0.079598172	0.099271737
5.34E-10	0.073977546	0.125605121	-0.020024386	0.678824794
2.01E-07	0.024119048	0.617948521	0.158987875	0.0009386
0.05148509	0.068828357	0.154217701	-0.275219472	6.52E-09
0.097725847	0.111479195	0.020768644	-0.040889907	0.397666125
0.000253417	-0.198736234	3.32E-05	0.099520934	0.039129123

0.011030542	0.25745125	6.14E-08	-0.16527689	0.000579629
0.201674896	-0.037248677	0.441048408	-0.236996328	6.66E-07
0.471869586	-0.019594938	0.685341224	-0.029709373	0.538945081
5.07E-06	0.05071958	0.294013559	-0.238118809	5.87E-07
1.75E-16	-0.097083306	0.044213903	0.198537968	3.38E-05
1.19E-08	0.212706201	8.63E-06	-0.015534052	0.748056793
0.050273989	0.500644009	1.17E-28	-0.139277303	0.003805797
0.710264742	0.068551586	0.155885066	-0.105522985	0.028675907
1.84E-09	0.016285079	0.736317481	0.080252513	0.096514988
0.366477661	0.126103136	0.008850482	-0.069399116	0.150821946
0.055287742	0.154750483	0.00128618	0.394152503	1.96E-17
0.000276346	0.202200094	2.40E-05	-0.37612114	6.74E-16
0.739891333	0.047415079	0.326637241	0.095237842	0.048423448
0.314448809	0.110831324	0.021525215	0.382106957	2.14E-16
0.870632241	-0.262660675	3.24E-08	-0.055442602	0.251291426
0.724592049	0.178370383	0.00020092	0.210901081	1.03E-05
0.00012159	0.199218271	3.17E-05	-0.155684355	0.001200711
2.54E-05	0.009339212	0.846878693	-0.384783447	1.27E-16
0.002868315	-0.067583153	0.161826608	0.026901394	0.577994804
1.84E-19	0.066730711	0.167196101	0.322958511	6.78E-12
0.965383646	0.028289793	0.558521405	-0.263922826	2.77E-08
4.43E-07	0.258757732	5.24E-08	-0.012990173	0.788239672
0.285835721	0.137851973	0.004184294	-0.265885696	2.16E-08
0.007990089	-0.109801851	0.022776606	0.116926191	0.015270402
9.06E-09	0.102013225	0.034450971	-0.262941593	3.13E-08
0.233152235	-0.020342648	0.674010858	-0.173298219	0.000305628
1.57E-11	0.243654571	3.14E-07	-0.040924858	0.397262533
0.20500408	0.002116801	0.965089983	-0.29008111	8.79E-10
0.586828474	0.148447545	0.00202575	-0.074190742	0.124515986
0.194548568	0.282099403	2.62E-09	-0.06678625	0.166842251
0.150759213	-0.256788557	6.66E-08	-0.074311318	0.123903272
0.147424258	0.060562821	0.210077458	-0.049893818	0.301954268
1.24E-16	-0.103269448	0.032279328	0.12329556	0.010496173
2.15E-12	-0.155784728	0.001191842	0.188767357	8.20E-05
2.06E-10	0.019044259	0.693731408	0.390573274	4.03E-17
0.005592859	0.164272658	0.000626731	0.176391887	0.000236961
1.04E-11	-0.132152968	0.006061204	0.197471466	3.73E-05
0.981091707	0.00296575	0.951104181	0.138157984	0.004100277
0.530135412	0.128828774	0.00747674	-0.248676119	1.75E-07
0.972776745	0.03077078	0.524535412	-0.000946871	0.984380348
1.20E-25	0.131208846	0.006436516	-0.077251266	0.109677453
1.33E-07	-0.139664664	0.003708436	0.205061758	1.82E-05
2.05E-10	-0.097903973	0.042443273	0.002962443	0.951158643
0.000176286	0.120955946	0.012069114	0.369195323	2.48E-15
4.53E-10	0.065600229	0.174521277	0.321447159	8.59E-12
0.314717176	0.483875853	1.28E-26	-0.043683207	0.366192209
0.000446498	0.007835037	0.871305592	0.265891873	2.16E-08
1.27E-05	-0.21952284	4.33E-06	-0.048602857	0.314650896
0.429629487	-0.032571872	0.500541865	-0.273208416	8.47E-09
0.760825139	-0.008931591	0.853484752	0.240484712	4.50E-07
0.910548464	0.423632905	3.73E-20	-0.039831236	0.410007274

0.056711868	0.345856639	1.58E-13	0.051012041	0.291234968
0.53739577	0.124207261	0.009934244	-0.236844745	6.77E-07
0.008612561	0.087129124	0.071086803	0.157042083	0.001085746
0.312400784	0.195068103	4.65E-05	-0.151261726	0.001657365
0.004290208	0.025570937	0.596948899	-0.02702855	0.576198172
0.911252503	0.043178841	0.371757676	-0.014950561	0.757217868
0.519997353	0.033967621	0.482354926	-0.309217715	5.57E-11
6.51E-07	0.237176627	6.52E-07	0.08269559	0.086757572
0.000349391	-0.057461934	0.234412029	0.425225825	2.61E-20
1.62E-05	0.153352451	0.001424629	-0.068792955	0.154430224
0.001201697	0.037093486	0.442955503	0.357506693	2.07E-14
0.250745078	0.021417802	0.657847467	0.235945067	7.48E-07
0.041845451	-0.222517197	3.17E-06	-0.2190308	4.55E-06
0.437752793	0.046753646	0.333438503	-0.216299283	6.01E-06
8.00E-14	0.11704203	0.015168878	0.164514276	0.000615084
3.92E-11	-0.136704436	0.004513398	-0.233153255	1.02E-06
0.809373965	0.418641831	1.13E-19	0.055428108	0.251415566
2.22E-05	0.216595584	5.84E-06	-0.239450089	5.06E-07
6.64E-33	0.048582085	0.314858006	-0.292010405	6.71E-10
0.926863795	-0.202864096	2.25E-05	0.113711611	0.018335826
7.10E-05	0.092755182	0.054610952	-0.28869668	1.06E-09
0.047862163	0.047416471	0.326623019	-0.504858854	3.44E-29
0.0007889	-0.132402578	0.005965319	0.153596675	0.001399497
0.810942895	0.031067011	0.520549161	-0.22138134	3.57E-06
1.54E-06	0.129832004	0.007021222	0.128061812	0.007842604
3.73E-11	-0.015160073	0.753924443	-0.324094505	5.67E-12
4.09E-06	-0.050987639	0.291466127	-0.018696089	0.699055601
0.565683766	0.23398802	9.28E-07	-0.215305304	6.65E-06
5.84E-08	-0.00919407	0.849229709	0.073850187	0.126259267
0.454242722	-0.025774254	0.594034206	-0.507923704	1.40E-29
0.000751282	0.074826029	0.121314059	-0.181276767	0.000157174
0.01496838	0.119066711	0.013487623	-0.116056573	0.016051775
5.07E-05	0.062663107	0.194664819	0.314498487	2.51E-11
3.45E-10	-0.002320891	0.961726613	0.078730909	0.103021597
0.499177557	-0.009353375	0.846649339	-0.161676882	0.000765413
4.20E-08	0.146974788	0.002247068	-0.098577184	0.041035762
2.76E-08	-0.036551022	0.449658491	0.283152344	2.27E-09
1.91E-12	-0.029676112	0.539399802	0.433676868	3.81E-21
0.020660898	0.139958818	0.003636016	-0.153935905	0.001365263
2.41E-09	-0.089358607	0.064127742	0.096425132	0.045678469
0.183245623	0.052970042	0.273086474	-0.19309555	5.57E-05
0.732569638	-0.115413094	0.016652292	0.228459954	1.69E-06
0.000721354	0.027125145	0.574835105	-0.17300618	0.00031299
2.63E-06	0.171381965	0.000357043	-0.009851109	0.838597903
0.006366372	0.019345383	0.689138752	-0.385807602	1.04E-16
0.336056458	0.202016527	2.44E-05	-0.116264647	0.015861694
0.591390792	-0.136578027	0.004551045	0.114526977	0.017511379
0.000147055	0.252076128	1.17E-07	-0.211699964	9.54E-06
0.056580439	0.245675125	2.48E-07	-0.059617244	0.217296627
0.036939461	-0.006175946	0.898389896	-0.333947466	1.16E-12
0.061811724	0.025386368	0.599600428	0.408608032	9.82E-19

0.002382878	-0.002650853	0.956290358	-0.425829804	2.28E-20
0.955582111	0.151794665	0.00159494	0.006456803	0.893795778
0.000794496	0.05478909	0.256930773	0.185587881	0.00010843
0.067443518	0.252617236	1.10E-07	0.188262409	8.58E-05
3.37E-11	0.032922314	0.495941727	0.043943031	0.363345485
0.618304423	-0.114630987	0.017408576	0.013783899	0.775636062
0.800864394	0.167443924	0.000488962	-0.223608221	2.83E-06
0.896479387	0.087763004	0.069048178	0.387332133	7.68E-17
0.01127853	0.313035509	3.14E-11	-0.008550176	0.859675424
5.65E-05	0.082170971	0.088783008	-0.444449189	3.03E-22
0.218516354	0.073214103	0.129565999	-0.396680121	1.18E-17
0.074798353	0.181468239	0.000154631	-0.102818073	0.033045776
0.260855123	0.074224758	0.124342894	0.320328841	1.02E-11
1.59E-05	0.06782582	0.160322037	0.323435114	6.29E-12
0.363951091	0.11085579	0.021496216	-0.216587693	5.84E-06
0.002217172	0.110699059	0.021682577	0.114371802	0.01766574
3.70E-21	0.235655573	7.72E-07	0.039916393	0.409006276
0.865836273	0.125417478	0.009229641	-0.175281018	0.000259761
0.075833445	0.0880015	0.068293648	-0.23812909	5.86E-07
0.067347037	0.04052258	0.401922631	-0.2912721	7.44E-10
0.065050952	-0.128067193	0.007839983	-0.073226796	0.129499362
0.114942432	0.051218013	0.289288669	-0.153998014	0.00135908
0.000562766	0.387315449	7.70E-17	-0.176351107	0.000237763
2.33E-07	0.126354371	0.008715063	0.359839347	1.37E-14
0.005658058	0.167905187	0.000471449	-0.085266169	0.077364349
0.122320524	0.153917463	0.001367105	-0.168492714	0.000449986
0.409339775	0.279622922	3.64E-09	0.105969584	0.028004337
0.000448703	0.159751633	0.000886052	0.281216765	2.94E-09
0.042115209	0.177147299	0.00022254	-0.340118821	4.18E-13
1.86E-11	-0.029059517	0.547864071	0.301925235	1.63E-10
0.025416663	0.092118305	0.056300088	-0.251041912	1.33E-07
0.65596703	0.109419049	0.023257736	-0.13253745	0.005914077
0.150877423	0.114564303	0.017474426	-0.090609635	0.060474887
0.014113694	0.026441477	0.584514968	-0.215850464	6.29E-06
3.44E-10	-0.013930495	0.773314575	0.208572038	1.30E-05
0.019195444	0.164509994	0.000615289	0.109137973	0.023616587
0.003935437	0.267484293	1.77E-08	-0.044814038	0.353903453
4.38E-05	0.017433576	0.718484164	0.332706453	1.42E-12
0.742324531	0.179281676	0.000186107	-0.261155599	3.90E-08
0.489961959	0.042950148	0.374298342	-0.136089712	0.004699148
0.000734174	0.088799916	0.065816712	-0.177313526	0.000219479
3.90E-06	-0.012159154	0.801494872	0.362119302	9.06E-15
0.213425537	0.065368736	0.176050294	-0.19372297	5.26E-05
0.00085139	0.046857272	0.33236698	0.340422025	3.97E-13
0.777035139	-0.099128675	0.039912294	-0.355511365	2.95E-14
0.618701037	0.141120228	0.003362429	0.266681979	1.95E-08
0.311132236	-0.012348269	0.798473219	0.181447678	0.000154903
5.01E-11	0.040133585	0.406459795	-0.393971468	2.04E-17
0.358909707	-0.039794689	0.410437323	-0.152220466	0.001546622
0.667462228	-0.066951569	0.16579229	-0.29690325	3.36E-10
0.027329763	0.190895916	6.79E-05	0.229287418	1.55E-06

0.87348085	0.058651802	0.224849147	-0.323877095	5.86E-12
1.80E-05	-0.029107268	0.547206244	0.200328432	2.86E-05
6.27E-14	0.081414852	0.091768823	-0.053383501	0.26935493
1.67E-06	0.163723764	0.000653951	-0.042112306	0.383697418
3.66E-11	-0.04658696	0.335166723	0.22043798	3.94E-06
0.465810812	0.109495424	0.023161047	-0.136684956	0.004519182
1.25E-09	-0.055047886	0.254687167	-0.070605756	0.143830301
0.000710297	0.038133572	0.430264147	-0.0944802	0.050246243
0.000264488	-0.06202239	0.199276353	0.293774414	5.24E-10
0.336095859	0.337515863	6.45E-13	0.014082665	0.770906963
0.002333805	0.014677317	0.761519725	0.434957753	2.83E-21
0.01334399	0.081611008	0.090986609	0.152556997	0.001509389
0.003083222	-0.025368617	0.599855724	-0.235607483	7.77E-07
2.10E-15	-0.026464402	0.584189153	-0.16574533	0.000558801
4.35E-05	0.230724053	1.33E-06	0.235429791	7.92E-07
2.49E-11	-0.073666655	0.127206576	0.357068895	2.24E-14
2.70E-10	-0.002661469	0.956115489	-0.070093948	0.146765016
0.629458042	-0.016259511	0.736716143	-0.398012006	8.95E-18
0.9870553	-0.011398433	0.813678989	-0.18823423	8.60E-05
7.87E-06	0.206468919	1.59E-05	0.071082769	0.141135677
0.001650893	0.119939135	0.012815285	-0.282491408	2.48E-09
0.851563209	-0.07574483	0.116797158	0.041276632	0.393214222
0.004463528	-0.015343405	0.751046219	-0.322035985	7.83E-12
0.000487529	0.003033705	0.949985274	-0.270787724	1.16E-08
4.38E-08	0.146276323	0.002359563	-0.000381312	0.993709521
0.00198012	0.085229662	0.077491721	-0.074218698	0.124373715
0.804530477	0.039863753	0.409624875	0.345275419	1.75E-13
0.066657963	0.012815254	0.791024784	0.24714284	2.10E-07
2.96E-05	0.023121433	0.632561571	-0.326398795	3.93E-12
0.386292585	-0.004675374	0.922989317	-0.385616883	1.08E-16
0.000225774	0.130214004	0.006854411	0.078169865	0.105506637
0.723152588	0.022693565	0.638873503	-0.34058354	3.87E-13
0.002825503	0.214309837	7.35E-06	-0.111987371	0.020191476
1.98E-09	0.007020615	0.88458409	-0.347169305	1.26E-13
0.640311628	-0.012975858	0.788467504	-0.395237582	1.58E-17
0.140220584	0.118058407	0.014303204	0.378428273	4.34E-16
0.83963102	0.177233209	0.000220953	0.109045548	0.023735628
5.90E-10	-0.207238533	1.48E-05	0.026313775	0.586331412
1.55E-10	-0.144312736	0.002703895	0.073495208	0.128096483
0.802861962	0.014792399	0.759707013	-0.303382476	1.32E-10
0.007751406	0.245289267	2.60E-07	0.086138518	0.074370937
3.64E-07	0.045849573	0.342880917	0.313968686	2.72E-11
3.43E-06	0.060206725	0.212775558	0.124235658	0.009917178
0.006167199	0.109088174	0.023680663	-0.035943453	0.457233518
1.75E-05	0.103340293	0.03216042	-0.061712864	0.201532435
4.06E-25	0.300307672	2.06E-10	0.100766731	0.036727516
0.803159949	-0.006793436	0.888293988	-0.183356036	0.000131545
2.64E-07	0.118504632	0.013937068	0.283311653	2.22E-09
0.011413584	0.015747613	0.744712556	0.304956729	1.05E-10
0.000309274	-0.15747405	0.001051354	-0.131371565	0.006370382
4.68E-15	-0.259440969	4.82E-08	0.124255032	0.009905551

1.09E-06	-0.001414568	0.976667006	-0.402797646	3.33E-18
0.030388197	0.022863469	0.636363925	-0.425649595	2.37E-20
5.65E-05	0.168607499	0.0004459	0.090069505	0.062030356
0.000859664	0.039516535	0.413719097	0.33613582	8.11E-13
7.43E-11	0.267446568	1.77E-08	0.090264902	0.061463885
0.009041928	0.14774813	0.002128242	0.245547763	2.52E-07
1.49E-16	0.04233426	0.381193591	0.21180502	9.44E-06
0.001989743	0.076056458	0.115295421	-0.261569786	3.71E-08
1.63E-11	0.227529966	1.87E-06	-0.077418104	0.108910437
0.000518503	0.173509468	0.000300404	0.311541978	3.93E-11
0.013209245	0.090624778	0.060431748	-0.046581787	0.335220459
0.00082983	-0.329150972	2.53E-12	-0.044702213	0.355106957
0.739199341	0.049131437	0.309410624	-0.199007581	3.23E-05
0.617889849	0.29857064	2.65E-10	-0.110824723	0.021533045
0.666160623	0.302772561	1.44E-10	-0.086241062	0.07402534
4.01E-22	0.217031071	5.58E-06	0.106391254	0.027382698
1.65E-07	0.196381122	4.12E-05	-0.176688607	0.000231195
0.096053794	0.370016107	2.13E-15	-0.188810705	8.17E-05
0.056639183	0.061105212	0.206015384	-0.108855313	0.023982278
0.181091672	0.353582289	4.15E-14	-0.207362061	1.46E-05
0.038747679	-0.299143777	2.44E-10	-0.028710412	0.552685227
7.04E-11	-0.056417165	0.243042491	0.310133008	4.86E-11
0.05676409	-0.015898895	0.742346465	0.347990612	1.10E-13
6.33E-06	-0.326866343	3.65E-12	0.116067555	0.016041692
0.00844093	0.157964167	0.00101355	-0.120785869	0.012191184
0.000128441	0.085107783	0.077918192	0.000810838	0.986624131
0.152908359	0.05581479	0.24811846	-0.146715734	0.002288207
0.364477777	-0.064419866	0.182421707	-0.011765827	0.807788807
6.78E-06	-0.038976915	0.420129727	-0.416122319	1.95E-19
3.95E-09	-0.075596837	0.117515676	0.213411555	8.04E-06
0.974835701	0.178995248	0.000190649	-0.013701362	0.776943989
0.022931043	0.058738798	0.224161023	-0.113307388	0.018756962
0.015390052	0.000811537	0.986612602	0.040230286	0.405329058
0.419017465	0.442271085	5.09E-22	-0.051091026	0.290487573
3.40E-06	0.075330878	0.118815615	-0.027508269	0.569443801
0.516936149	0.006079206	0.899973109	0.4079314	1.13E-18
0.047145729	-0.000636633	0.989497694	0.165503887	0.000569448
0.45325486	-0.253560156	9.82E-08	0.048423163	0.316445527
0.093354878	0.108216794	0.024826414	-0.058844807	0.223324544
0.019915191	-0.128456851	0.00765221	0.461742272	4.29E-24
1.63E-13	0.040540477	0.401714621	0.221161847	3.65E-06
3.48E-07	0.075445227	0.118255338	-0.000305132	0.994966238
7.09E-06	0.279542355	3.68E-09	0.166418235	0.000530094
0.062286454	0.029039297	0.548142753	0.181722428	0.000151315
0.000248712	0.051864716	0.28323461	-0.072043816	0.135824393
4.64E-06	0.15034413	0.001770119	-0.134548909	0.005195397
0.430698071	0.26891569	1.47E-08	-0.116067181	0.016042036
0.108795832	0.07951581	0.09962312	0.104986552	0.029500793
0.00219945	0.084058395	0.081669448	0.386303706	9.40E-17
0.352728983	0.163837373	0.000648229	-0.163096953	0.000686374
0.440683025	0.116474695	0.015671815	-0.236883242	6.74E-07

1.82E-15	-0.167915257	0.000471073	0.086218108	0.074102589
3.94E-09	0.102156171	0.034197748	0.209786218	1.15E-05
0.149986801	0.118967895	0.0135657	-0.359941994	1.34E-14
0.692746002	-0.112578963	0.01953718	0.349673995	8.21E-14
9.04E-18	0.005243394	0.913667623	-0.425474193	2.47E-20
0.000528194	-0.189802782	7.48E-05	0.022387099	0.643410613
0.082350312	-0.028685218	0.553033967	0.009842302	0.838740218
3.18E-14	0.119089797	0.013469439	0.095482607	0.04784653
2.92E-10	0.031969846	0.508496799	-0.290586532	8.19E-10
0.002814942	0.234110487	9.16E-07	-0.113254051	0.018813156
1.25E-06	0.206633462	1.57E-05	0.011135526	0.817900492
0.078915622	-0.326843872	3.66E-12	-0.040890301	0.397661572
0.007244022	-0.323770764	5.96E-12	0.0025021	0.958740886
0.002390622	0.118378405	0.014039794	0.440501323	7.74E-22
0.323766508	0.019831134	0.681754272	-0.371603187	1.58E-15
4.45E-11	0.034675816	0.473265721	-0.359009084	1.59E-14
0.423337702	0.047715542	0.323577572	0.241212113	4.14E-07
0.036581342	-0.301687015	1.69E-10	-0.058046703	0.229677004
0.019399365	-0.090891857	0.059675044	0.07140604	0.139331626
0.100605357	-0.033549879	0.487760383	0.137778447	0.004204712
0.84818584	0.046640248	0.334613605	-0.213275867	8.15E-06
0.902505039	0.040113636	0.406693292	0.107542836	0.0257451
0.764032289	0.387186063	7.90E-17	-0.176742318	0.000230166
0.223033868	0.288775065	1.05E-09	-0.243952942	3.03E-07
0.049172275	0.098486957	0.041222087	0.33916826	4.90E-13
0.246170805	0.196885748	3.94E-05	-0.143545833	0.00285037
2.31E-12	-0.016966738	0.72571522	0.377802475	4.89E-16
0.549854131	0.185089638	0.000113232	0.031586339	0.513598543
9.85E-12	0.106644701	0.027014796	-0.201007256	2.68E-05
2.31E-07	-0.097325009	0.043686076	0.117290334	0.014953252
0.044453572	0.151920499	0.001580519	0.122144812	0.011245558
2.78E-10	0.297577179	3.06E-10	-0.169550936	0.000413603
3.57E-05	0.412813754	4.00E-19	0.064690332	0.180588485
0.000599164	0.244686222	2.78E-07	0.067484051	0.162444102
0.108333936	0.124876712	0.009538796	-0.231998564	1.15E-06
1.82E-05	0.013587537	0.778748775	0.418385367	1.19E-19
0.002953616	0.098422928	0.041354743	0.376323302	6.49E-16
0.297279084	0.077290483	0.109496778	-0.199676469	3.04E-05
4.40E-09	-0.041907415	0.386017654	0.098659412	0.040866578
0.447666464	0.012180693	0.801150572	-0.052627815	0.276201671
0.219208286	0.238490375	5.63E-07	-0.339146199	4.92E-13
0.087576854	0.045185412	0.349925044	-0.270957697	1.13E-08
1.49E-13	0.336649623	7.45E-13	0.137037741	0.004415481
5.28E-05	-0.005528077	0.909000113	0.081296247	0.09224439
0.013178563	-0.025376755	0.599738685	0.32323566	6.49E-12
0.016371014	-0.222545073	3.16E-06	0.052368971	0.278573831
0.000805389	0.229375273	1.53E-06	0.014668953	0.761651511
0.004589287	-0.251844065	1.21E-07	-0.145869631	0.002427408
0.007281718	-0.010401837	0.829708812	0.31990054	1.09E-11
1.28E-07	0.005984259	0.901527377	-0.048182414	0.318860387
0.92977312	0.013855394	0.774503632	-0.382663412	1.92E-16

0.225761642	0.007646062	0.874383593	-0.042038898	0.384527736
0.922413818	0.081216093	0.0925669	-0.365555218	4.84E-15
0.006947524	0.099933487	0.038319506	-0.240744487	4.37E-07
0.855473082	-0.102140345	0.034225705	0.246415325	2.28E-07
0.207692786	-0.200700647	2.76E-05	0.078819242	0.102634606
0.78495088	0.228240937	1.73E-06	0.107397805	0.025946581
0.033324098	0.205472199	1.75E-05	0.024175387	0.617127665
0.000976127	0.145983056	0.002408309	0.159058651	0.000933612
0.000145705	0.269725406	1.33E-08	0.090897852	0.059658148
0.000940739	0.054652599	0.258119552	-0.307249731	7.47E-11
2.80E-05	0.153472258	0.001412249	-0.348200732	1.06E-13
7.29E-13	-0.009696837	0.84109167	0.061956205	0.199757209
0.000355865	-0.004715967	0.922322772	-0.065718852	0.173741607
0.045661731	-0.033185218	0.492505483	-0.090320868	0.061302424
5.50E-06	0.116512069	0.015638238	0.39109952	3.63E-17
0.860826997	0.017438456	0.718408706	-0.12765126	0.008044947
0.435853389	0.065263326	0.176749819	-0.324345962	5.45E-12
0.977075695	0.07001931	0.147196777	-0.261292462	3.83E-08
0.1750549	0.264199119	2.67E-08	0.087291208	0.070560901
0.180531992	0.401674085	4.21E-18	-0.087532111	0.069785139
0.005162138	0.22553026	2.31E-06	-0.148318271	0.002044347
3.68E-18	0.0948522	0.049344219	-0.206044216	1.66E-05
0.889315861	-0.146815146	0.00227234	0.126957565	0.008397438
0.134821347	-0.032658965	0.499396522	0.365787243	4.64E-15
0.000489847	0.036086844	0.455439343	-0.209682222	1.16E-05
7.23E-05	-0.019958514	0.679822791	0.117802124	0.014517289
3.33E-10	-0.015479715	0.748908445	0.220049544	4.10E-06
1.04E-20	-0.023593244	0.62563221	0.15634734	0.001143241
8.71E-12	0.213461826	8.00E-06	0.152597686	0.001504943
0.039242373	0.203184415	2.18E-05	-0.206542053	1.58E-05
1.61E-18	-0.06589412	0.172594377	-0.194227297	5.02E-05
0.124323928	0.025856831	0.59285226	-0.260479035	4.24E-08
0.007990945	0.134770477	0.005121247	0.438551178	1.23E-21
0.00026189	0.243769787	3.09E-07	-0.029706933	0.538978431
0.531647042	0.096005022	0.046634427	0.016768374	0.728795204
0.93513143	0.093827659	0.051861786	-0.148956668	0.001954004
0.000977319	0.3251201	4.82E-12	0.058101932	0.229233326
1.13E-05	0.303636782	1.27E-10	0.085763862	0.075644785
0.210454551	0.11146238	0.020787984	-0.069092607	0.152638406
5.51E-08	0.394372087	1.88E-17	0.144847222	0.002605899
0.028138869	0.097606267	0.043078555	0.088996842	0.065217268
0.04486096	-0.196895533	3.93E-05	0.216026799	6.18E-06
0.263777262	-0.022724669	0.638413763	-0.161611572	0.000769243
0.075990407	-0.320719101	9.63E-12	0.053427356	0.268961188
0.088677025	-0.01261593	0.794201743	-0.093060094	0.053817295
5.73E-05	0.112593497	0.01952134	-0.064620103	0.181063176
0.002727852	0.035001123	0.469122304	-0.294671451	4.62E-10
0.397842155	-0.0077656	0.872436344	0.312437415	3.44E-11
0.81695007	0.026651961	0.581526735	0.348336663	1.03E-13
0.009359121	-0.118626698	0.013838358	-0.075206052	0.119429591
4.78E-06	0.061933434	0.199922848	-0.145208376	0.002541527

0.799913368	0.116315717	0.015815344	0.217859042	5.13E-06
2.70E-11	0.1052643	0.029071189	0.438882891	1.13E-21
0.004543201	-0.183804948	0.000126553	0.225000157	2.44E-06
2.17E-05	0.136726889	0.004506741	0.061291385	0.20463429
0.000262864	0.019928258	0.680281381	-0.400628504	5.23E-18
0.084608554	0.384312891	1.39E-16	0.001601638	0.973582387
0.214472249	0.102120318	0.03426111	-0.197313854	3.78E-05
0.006448684	-0.084315595	0.080736789	-0.112927633	0.019160262
7.71E-16	0.09625183	0.046070804	-0.345722969	1.62E-13
1.48E-07	0.178450013	0.000199583	0.13767173	0.004234507
0.187907386	-0.011902235	0.805604597	-0.22092429	3.74E-06
0.141090065	-0.016562399	0.731997982	-0.295155354	4.31E-10
3.66E-07	0.123758979	0.010207116	-0.180143332	0.000173048
0.11503112	-0.107925988	0.025219289	0.238995127	5.32E-07
0.607922703	0.141687116	0.003235795	0.016955796	0.725885005
7.70E-13	-0.153018596	0.001459656	-0.02242101	0.642907919
4.37E-09	0.264090591	2.71E-08	-0.066009066	0.171845063
0.008836931	-0.227704819	1.83E-06	0.169309389	0.000421658
4.71E-15	-0.342860571	2.64E-13	-0.232003852	1.15E-06
0.003651854	0.331845213	1.64E-12	-0.132412426	0.005961564
0.475433186	0.017900006	0.711284425	0.35467106	3.43E-14
1.37E-08	0.285255794	1.71E-09	0.114758136	0.017283618
0.524732869	0.13979854	0.003675316	-0.211903251	9.35E-06
9.47E-05	0.113330998	0.018732135	0.094079135	0.051234132
0.031114435	-0.001679446	0.972299523	-0.333895595	1.17E-12
2.39E-05	0.42690426	1.79E-20	0.081825702	0.090136608
0.155027752	0.040210738	0.405557483	-0.345634515	1.64E-13
0.240403271	0.109987653	0.0225462	0.203359414	2.15E-05
0.610162111	0.087547207	0.069736762	0.293009151	5.84E-10
1.25E-05	0.01185521	0.806357416	0.345827061	1.59E-13
0.783022855	0.138065088	0.004125619	0.310351226	4.70E-11
0.645548261	0.219101973	4.52E-06	-0.10571914	0.028379257
0.000740544	0.088118555	0.0679258	0.126638442	0.00856417
2.89E-07	0.008718128	0.856948346	0.222797027	3.08E-06
2.83E-14	-0.031237312	0.51826456	0.03890686	0.420966208
3.06E-05	0.081253815	0.092415007	0.238548606	5.60E-07
0.0021229	-0.019622789	0.684917897	-0.3385828	5.40E-13
3.26E-12	0.03526782	0.465740372	-0.088978973	0.065271473
0.084355925	0.062800278	0.19368776	0.096839281	0.044752233
6.44E-06	0.062774881	0.193868388	0.08125232	0.092421024
0.070626566	0.079278563	0.100640824	-0.093766976	0.052014197
1.85E-06	-0.018555973	0.701202425	0.404889314	2.15E-18
1.03E-05	-0.006596996	0.891503891	0.213680903	7.83E-06
1.87E-09	-0.037881779	0.433317139	0.044060715	0.362060651
0.004491537	0.220762262	3.81E-06	0.225999351	2.20E-06
0.434754969	-0.01354361	0.779445582	-0.223561228	2.84E-06
0.067599898	0.027356684	0.571574011	-0.341180288	3.50E-13
0.572867666	-0.154960905	0.001266447	-0.07725577	0.109656693
5.27E-20	0.032527173	0.501130242	0.298651671	2.62E-10
0.004425516	-0.265457303	2.28E-08	0.034792065	0.471782768
0.139252185	0.091649942	0.057569758	-0.303277246	1.34E-10

0.024750534	0.019483065	0.687042636	-0.122481939	0.011021259
0.170374859	0.234082871	9.19E-07	0.076739566	0.112056479
0.960774761	0.07537181	0.118614824	0.316264661	1.92E-11
0.118485821	0.064024479	0.185126335	-0.287662841	1.23E-09
0.099890342	-0.024159166	0.617363959	0.2615848	3.70E-08
1.55E-09	0.028879374	0.550349272	0.161378837	0.000783037
0.342062305	0.363165151	7.49E-15	0.022910519	0.635669687
8.62E-12	-0.004497302	0.925913858	-0.134709579	0.005141532
0.080690441	0.040099986	0.406853104	-0.278981216	3.97E-09
0.523291571	0.182185181	0.000145449	0.07152004	0.138699674
2.45E-15	0.020147942	0.676954364	0.354972885	3.25E-14
0.08067109	-0.228227229	1.73E-06	0.03429804	0.478102503
6.80E-05	0.173470043	0.000301373	-0.387487436	7.44E-17
0.074491966	-0.124360409	0.009842516	0.300928063	1.89E-10
0.950031736	0.216804174	5.71E-06	-0.105956477	0.028023852
0.005805369	0.150442874	0.001757656	0.365084499	5.28E-15
0.188321992	0.003829269	0.936893771	0.293645671	5.34E-10
0.339043788	0.034101994	0.48062311	-0.354352136	3.63E-14
2.63E-14	0.013803216	0.775330053	0.399295318	6.88E-18
0.005926234	0.258681787	5.29E-08	0.104166438	0.030801188
0.215143539	0.124377124	0.00983255	-0.089681377	0.063168342
1.98E-06	-0.083731459	0.082867537	0.150813173	0.001711633
0.005838196	0.012256625	0.799937125	-0.150579078	0.001740598
0.890213671	0.119030181	0.01351644	-0.081200287	0.092630602
6.07E-08	0.272084158	9.80E-09	-0.118845968	0.013662587
0.568066099	-0.016898796	0.726769652	0.174351917	0.000280388
4.93E-06	0.148513768	0.002016283	-0.119620743	0.013057151
4.79E-15	0.014811977	0.759398749	0.157764991	0.001028759
1.38E-05	0.297235117	3.21E-10	-0.105253832	0.029087282
0.184864436	0.066694333	0.167428179	0.075707912	0.116976078
0.006579962	0.384215545	1.42E-16	-0.159001612	0.00093763
0.0030624	0.244913101	2.71E-07	-0.10048579	0.037257888
0.007103837	0.13036155	0.006790937	-0.259714192	4.66E-08
0.10186108	-0.06216566	0.198238329	0.082117608	0.088991138
6.78E-10	-0.005923522	0.902521818	0.258003094	5.74E-08
0.996334855	0.030003263	0.534935508	-0.369583712	2.30E-15
0.266285077	0.345040456	1.82E-13	0.081216612	0.09256481
0.563547221	0.307576817	7.12E-11	0.21672901	5.76E-06
3.89E-07	0.296309295	3.66E-10	-0.068669714	0.155171764
2.23E-11	0.108625558	0.024283118	0.04194978	0.385537202
0.519585857	0.23035675	1.38E-06	-0.090494922	0.060802515
0.683590826	0.044300751	0.35944882	-0.233613152	9.67E-07
0.12187699	0.024127442	0.617826195	0.271539623	1.05E-08
0.651568997	0.030113958	0.533429192	-0.245125319	2.65E-07
0.633195899	0.028366822	0.557450378	-0.334866694	9.99E-13
0.000534387	0.261581648	3.70E-08	-0.249505111	1.59E-07
2.24E-10	0.046066523	0.340599637	0.486269957	6.64E-27
0.188894087	0.190409442	7.09E-05	-0.117647296	0.014647984
0.475225062	0.059518987	0.218056861	0.175446682	0.000256235
0.001774165	-0.032535096	0.50102592	-0.12457104	0.009717588
0.035895489	0.155957958	0.001176679	0.280441697	3.27E-09

5.16E-10	0.161186873	0.000794585	-0.016933978	0.726223578
6.46E-07	-0.032820662	0.49727377	0.143419936	0.002875094
0.003027888	0.176788159	0.00022929	0.435449542	2.53E-21
3.05E-22	0.108209223	0.024836576	-0.246179023	2.34E-07
0.041978921	0.372491353	1.34E-15	-0.06824259	0.157762629
0.00034742	-0.296469191	3.58E-10	0.186183546	0.00010294
0.254972966	-0.146520525	0.00231966	0.172081094	0.000337416
0.011104289	-0.235635776	7.74E-07	0.199182452	3.18E-05
0.394574894	0.259062731	5.05E-08	-0.126786113	0.008486653
1.37E-17	0.046596483	0.335067834	0.290200468	8.64E-10
0.005381278	0.27196315	9.95E-09	-0.044115181	0.361466975
0.011584441	0.339113134	4.95E-13	0.077243983	0.109711031
9.42E-07	0.0348208	0.471416593	-0.396081557	1.33E-17
0.687350145	-0.041922738	0.385843836	-0.219823837	4.19E-06
0.754784031	0.214845805	6.96E-06	0.287145215	1.32E-09
5.34E-05	0.048493828	0.31573899	-0.251513995	1.25E-07
0.000314173	0.33788012	6.07E-13	-0.077436236	0.108827334
0.596388735	0.14939669	0.001893876	-0.318413806	1.38E-11
0.026921328	-0.182878749	0.000137055	0.101591446	0.03520746
0.226673041	0.05057156	0.295426595	-0.362703677	8.15E-15
0.26367435	0.143305696	0.002897698	-0.228744713	1.64E-06
7.16E-05	-0.30673424	8.06E-11	-0.240503798	4.49E-07
0.406090414	-0.010239087	0.832333498	0.077534786	0.108376527
0.117767379	0.092361216	0.055650792	-0.30827279	6.42E-11
0.486976814	0.097356335	0.043618057	0.101392834	0.035568551
0.063453652	0.211136292	1.01E-05	0.04221405	0.382548411
0.006677741	0.038907546	0.420958013	-0.375385749	7.75E-16
1.90E-05	0.027704941	0.566685614	0.032971591	0.495296692
0.395452048	0.005643514	0.907108353	0.207235665	1.48E-05
0.234567544	0.067806445	0.160441776	-0.141512501	0.003274332
2.34E-11	0.203377707	2.14E-05	0.144042372	0.002754732
7.30E-10	-0.048686418	0.313818645	0.343494461	2.37E-13
0.026401885	0.119405494	0.013222935	0.430131753	8.59E-21
0.606267902	0.155737698	0.00119599	0.435599327	2.44E-21
0.022745214	-0.047919727	0.321508986	0.306288496	8.61E-11
0.010880186	0.170519848	0.00038271	0.173971509	0.000289267
0.001085573	0.062385758	0.196651362	0.174952463	0.000266888
0.000355591	0.140922669	0.003407606	-0.021038505	0.663531962
9.06E-05	0.113419978	0.018638822	0.498649228	2.07E-28
0.961831931	0.042671804	0.377405	-0.381068942	2.61E-16
2.87E-11	-0.118715652	0.013766812	-0.135343624	0.004933839
0.089582825	0.207094785	1.50E-05	0.256852951	6.60E-08
0.29589873	0.022292371	0.644815719	0.134042593	0.005368483
0.235564412	0.008697259	0.857287108	-0.164222002	0.000629198
0.01710055	0.136664008	0.004525408	-0.142892577	0.002980793
0.137551779	0.072620555	0.132711673	0.478196885	5.92E-26
0.046387379	-0.006202115	0.897961689	-0.225080184	2.42E-06
3.44E-06	-0.057900325	0.230855846	-0.028842921	0.550852837
0.013503075	0.394662245	1.77E-17	0.135428907	0.004906487
0.078031315	0.337877948	6.08E-13	-0.153281371	0.001432021
4.27E-23	0.102224734	0.034076855	-0.039927391	0.408877097

0.000339159	0.042274367	0.381868241	-0.415195409	2.39E-19
2.00E-15	0.002135461	0.964782436	-0.297172669	3.24E-10
4.54E-25	-0.047970017	0.321000823	0.187664363	9.04E-05
0.004260123	0.005657104	0.906885672	-0.187901682	8.85E-05
0.124503564	0.029035681	0.548192597	-0.255330353	7.94E-08
0.007100136	-0.274354311	7.30E-09	-0.029225341	0.545581314
0.117875772	0.308352396	6.34E-11	0.15895903	0.00094064
7.41E-07	0.066546159	0.168375961	0.259092419	5.03E-08
1.67E-08	0.29815506	2.81E-10	-0.015134195	0.754330988
0.967474494	0.208832759	1.26E-05	-0.119072118	0.013483362
0.363176137	0.039194483	0.417538074	0.279093709	3.91E-09
0.203614434	0.020579797	0.670432427	-0.20104819	2.67E-05
0.000159779	-0.063487007	0.188850138	0.140488347	0.003508869
0.002791897	0.097486693	0.043335962	-0.316357353	1.89E-11
0.714392796	0.379100001	3.82E-16	-0.013431674	0.781222005
0.642587359	-0.041716028	0.388192666	0.109638736	0.022980555
0.001576783	0.059493222	0.218256529	0.034114147	0.480466649
0.007089955	0.04998628	0.30105813	-0.274080254	7.56E-09
0.091026632	-0.00216604	0.96427846	-0.334949835	9.86E-13
0.443023974	0.046227908	0.338908939	-0.383701617	1.57E-16
0.423084194	0.159514612	0.000902061	-0.314452769	2.53E-11
9.70E-06	0.048197249	0.318711237	0.237925896	6.00E-07
3.59E-13	0.211897845	9.35E-06	0.011725473	0.808435246
4.34E-08	-0.207665839	1.42E-05	-0.076571645	0.112845966
0.154446263	-0.312562367	3.37E-11	-0.071767046	0.137337964
0.111070291	-0.05292953	0.273453994	0.168907038	0.0004354
0.465480085	-0.015910367	0.742167134	0.405565485	1.87E-18
0.093184463	0.087555314	0.069710792	-0.246116285	2.36E-07
0.145543715	-0.21766869	5.23E-06	0.057614491	0.233170146
0.000316611	0.084594985	0.07973345	-0.378362757	4.39E-16
1.18E-06	-0.122738643	0.010853132	-0.106005937	0.027950271
0.001352671	0.045064493	0.351217289	0.067583053	0.161827232
0.003686863	0.024202801	0.616728429	0.398130842	8.74E-18
2.76E-09	-0.116286061	0.015842245	-0.078837954	0.102552772
0.022145684	-0.000464706	0.9923338	0.271403913	1.07E-08
0.02051602	0.199281489	3.15E-05	-0.234822842	8.47E-07
0.091943521	0.057945308	0.230493126	0.30426899	1.16E-10
0.310043817	-0.285040047	1.76E-09	-0.087083294	0.071236082
0.059088558	-0.150432708	0.001758935	0.016624486	0.731032079
5.31E-07	0.173710449	0.000295511	-0.370853578	1.82E-15
6.82E-13	0.031224762	0.518432747	0.059658788	0.216975763
9.17E-10	0.007818169	0.871580252	-0.438179033	1.34E-21
0.072672992	0.254596452	8.67E-08	0.076434058	0.11349609
0.754911529	0.030326114	0.53054818	-0.084144141	0.081357552
0.003077574	0.027279737	0.572656798	0.475468224	1.22E-25
0.659878442	0.300258933	2.08E-10	0.157402003	0.001057019
0.103351553	0.097034841	0.044320382	-0.230800959	1.31E-06
0.376975652	0.025873567	0.59261284	0.363525408	7.02E-15
1.67E-08	0.015220328	0.752978091	0.06448368	0.18198794
0.272590076	0.048751924	0.313167221	0.040193491	0.405759087
0.67339472	0.119918131	0.012831118	0.042629987	0.377873096

9.40E-11	-0.102882521	0.032935401	0.050548161	0.295650381
1.70E-07	-0.012965342	0.788634882	0.375842639	7.11E-16
0.022385163	0.025883922	0.592464736	0.087430258	0.070112272
0.259231534	0.036726423	0.447484884	-0.333944094	1.16E-12
0.037609437	0.364722046	5.64E-15	-0.063053552	0.191893137
0.307914601	0.233653582	9.63E-07	-0.118388672	0.014031414
6.63E-09	0.342177029	2.96E-13	-0.128544532	0.007610515
0.068571342	0.029475004	0.54215332	-0.06512866	0.177646498
0.17592603	0.04742873	0.32649783	-0.06711175	0.164779692
0.10470082	0.13390376	0.005416841	0.331701624	1.68E-12
0.234025648	0.025919298	0.591958865	0.221794234	3.42E-06
0.766055545	0.024243537	0.616135368	-0.300652192	1.96E-10
0.226697069	0.188333572	8.52E-05	-0.103895384	0.031241623
0.35223222	0.199954453	2.96E-05	-0.211216924	1.00E-05
0.00012483	0.161469904	0.000777613	0.341548599	3.29E-13
0.908931226	0.061651979	0.201978393	-0.224318865	2.63E-06
0.170835446	-0.10537338	0.028903944	0.308258817	6.43E-11
0.000111823	0.264552703	2.56E-08	0.01739131	0.71913782
0.695170473	-0.293831471	5.20E-10	-0.013193286	0.785009028
0.177418995	-0.158214897	0.000994699	0.041184447	0.394272692
0.440200763	-0.127246034	0.008249217	-0.099693935	0.038787871
0.03726032	0.024348594	0.614607056	-0.354464894	3.55E-14
4.01E-10	-0.087126927	0.071093951	-0.211523683	9.71E-06
0.075087305	0.170205984	0.000392474	-0.227148612	1.95E-06
0.643946343	-0.201488199	2.56E-05	-0.090026128	0.062156693
1.15E-05	0.059388436	0.219069906	0.246035519	2.38E-07
0.010641758	0.152153413	0.001554141	-0.062849994	0.193334517
0.191720922	-0.187321225	9.32E-05	0.010408832	0.829596046
0.005716882	0.052208587	0.280050569	0.204154739	1.99E-05
0.068228991	0.131416794	0.006352108	-0.031107901	0.520000147
0.083497469	-0.050045528	0.300484827	0.313851649	2.77E-11
0.139077491	-0.009622698	0.842290682	-0.101766492	0.034891807
9.07E-23	-0.033335217	0.490550666	0.343085907	2.54E-13
0.608506128	0.014695004	0.761241038	-0.31365442	2.86E-11
5.80E-09	0.065917525	0.172441604	0.02087855	0.665935019
0.06268089	0.001669174	0.972468879	-0.264619594	2.53E-08
8.62E-20	0.055854746	0.247779495	-0.005778815	0.90489172
0.61011322	0.162644049	0.000710721	0.35739832	2.11E-14
0.001226814	-0.07950746	0.099658799	0.015805047	0.743813984
0.029043668	0.060962323	0.207079952	-0.151728728	0.001602545
6.66E-15	0.174694758	0.000272605	-0.013424209	0.781340515
0.000120063	0.28273883	2.40E-09	0.047861535	0.322097643
0.007942075	0.156364083	0.001141823	0.34060818	3.85E-13
0.006331479	-0.026419928	0.584821303	-0.239226638	5.19E-07
5.53E-09	-0.001434482	0.976338632	0.240401598	4.54E-07
0.149630922	0.079928258	0.097873355	-0.344182014	2.11E-13
0.003825609	-0.010525319	0.827718682	0.274826898	6.86E-09
0.003282305	0.494871498	6.05E-28	0.091188871	0.058842742
0.00594004	0.107269284	0.02612626	0.203848021	2.05E-05
0.054071882	0.173902714	0.0002909	0.315047752	2.31E-11
0.620176619	0.004514985	0.92562339	0.257511594	6.10E-08

0.4949224	0.245871667	2.43E-07	0.304585156	1.11E-10
7.50E-09	0.186561582	9.96E-05	0.075470737	0.118130629
0.320833511	-0.000709843	0.988290054	-0.037060679	0.443359262
0.050680583	0.083556275	0.083515334	-0.24206819	3.76E-07
0.195440486	0.069495852	0.15025208	-0.405829004	1.77E-18
0.000532451	0.11285684	0.019236273	0.315424782	2.18E-11
0.550500508	0.033478945	0.48868148	-0.366007964	4.46E-15
3.22E-05	0.2650687	2.40E-08	0.135247949	0.004964686
0.844112475	-0.045975596	0.341554576	-0.032933542	0.495794717
0.234377715	0.13645958	0.004586577	-0.036141396	0.454757799
0.088878955	-0.102730142	0.033196879	0.23102888	1.28E-06
0.616538684	-0.008397919	0.862149094	0.195395087	4.52E-05
0.015362452	0.037791784	0.434411346	-0.40908371	8.88E-19
0.063579704	-0.237970287	5.97E-07	0.044816819	0.353873553
0.047312655	0.072017346	0.135968592	-0.346847882	1.34E-13
4.77E-05	-0.204235292	1.97E-05	0.047752403	0.323203504
0.006621763	0.175468872	0.000255767	-0.239762477	4.88E-07
0.008481416	0.114591875	0.017447173	-0.113801107	0.018243709
0.000410662	0.021741608	0.653010153	0.269862383	1.30E-08
1.02E-05	-0.255940617	7.38E-08	0.043616241	0.366928154
0.00013451	0.019084846	0.693111727	0.104583795	0.030133412
0.533559512	-0.087225247	0.070774535	0.099054748	0.040061368
0.344966494	-0.152143041	0.001555307	0.242301597	3.66E-07
6.66E-09	0.169804935	0.000405287	-0.110519738	0.021897517
0.002846937	0.050709694	0.294107792	0.158798419	0.000952073
0.00607103	0.117373925	0.01488127	0.061553987	0.202697645
0.734587434	-0.20636916	1.61E-05	-0.051390902	0.28766174
0.017420141	0.071705382	0.137676943	-0.149983126	0.001816373
2.20E-07	-0.112379264	0.019755951	0.06188036	0.200309294
0.000859779	0.114037412	0.01800242	0.08195513	0.08962727
0.088413073	0.033448889	0.489072033	-0.342443353	2.83E-13
0.010315132	-0.075367554	0.118635686	0.234884602	8.41E-07
0.09956336	0.096356845	0.045832725	-0.116950078	0.015249418
0.002234529	0.409298053	8.48E-19	-0.056093983	0.245756693
0.290889924	-0.011067383	0.818995521	-0.301259683	1.80E-10
0.034012133	0.032168305	0.505867164	-0.192838957	5.70E-05
7.05E-12	0.252287369	1.14E-07	0.021574877	0.655499144
0.000359326	0.001021155	0.983155129	-0.419208819	9.93E-20
0.063165512	0.295268258	4.24E-10	-0.098872315	0.040431259
0.1300869	-0.173228169	0.000307379	0.049033081	0.31038134
0.000514972	0.215411522	6.58E-06	-0.035594749	0.461613123
0.043216437	-0.35383711	3.97E-14	-0.125902398	0.008960025
0.436910215	-0.345656166	1.64E-13	-0.112199726	0.019954453
0.133587867	0.241262921	4.12E-07	0.101900733	0.034651368
0.811246738	-0.21791457	5.10E-06	0.153989078	0.001359968
0.000977547	-0.233128417	1.02E-06	0.059231314	0.220293599
3.90E-09	0.320167918	1.05E-11	0.094132623	0.051101452
0.360337937	0.349620787	8.29E-14	-0.086826872	0.072076047
0.660195681	0.020560448	0.670724116	0.476668915	8.89E-26
0.717033995	0.140197081	0.003578298	0.246748662	2.19E-07
3.67E-13	0.13282369	0.005806636	0.29594793	3.85E-10

1.67E-05	-0.148286621	0.002048924	0.259985315	4.51E-08
0.000502637	0.13381559	0.005447755	0.238547517	5.60E-07
0.214990235	0.111707529	0.02050756	0.107468676	0.025847956
1.65E-06	0.176157368	0.000241612	-0.171219823	0.000361745
0.985764202	-0.006640148	0.89079862	-0.153950436	0.001363814
0.255609116	0.576888117	1.64E-39	-0.042807834	0.375884771
0.686634871	0.054981339	0.255262788	0.133455162	0.005575787
0.063399211	0.035771295	0.459392856	-0.264163297	2.68E-08
4.52E-07	0.047759886	0.323127593	-0.272256025	9.58E-09
0.083115175	-0.027299472	0.572378991	-0.286914424	1.36E-09
0.263506253	0.244119744	2.97E-07	-0.155818854	0.001188841
0.155629801	0.190254409	7.19E-05	-0.235092716	8.22E-07
0.00563889	-0.060645228	0.209456598	-0.288616603	1.08E-09
9.91E-08	0.015042395	0.755773744	0.106348457	0.027445244
6.28E-11	0.040204961	0.405625001	0.14623072	0.002367083
0.754521943	-0.042487029	0.379476038	-0.080989759	0.093482448
1.27E-07	0.458274525	1.03E-23	0.018362457	0.704171349
0.666471432	-0.051607454	0.285632627	-0.018838154	0.696881347
2.72E-05	-0.159476267	0.000904676	0.261373849	3.80E-08
0.727401288	0.055168663	0.253644762	-0.318448356	1.37E-11
0.023243846	0.286405153	1.46E-09	0.220854885	3.77E-06
1.88E-06	0.090762964	0.060039246	-0.172447135	0.000327546
0.002044089	0.141844118	0.003201497	0.066334698	0.169735486
0.65384439	0.029029889	0.54827244	-0.330224023	2.13E-12
1.02E-06	-0.141542087	0.003267773	0.032398786	0.502822205
0.499886758	0.078783298	0.102791938	-0.062432528	0.196315331
4.55E-08	0.049104047	0.309680752	-0.221391684	3.57E-06
0.634530335	0.076485284	0.113253695	-0.175383765	0.000257569
0.137146218	0.148775215	0.001979303	0.363165787	7.49E-15
0.00032596	0.121108522	0.011960523	0.409135153	8.78E-19
0.556720904	0.284777137	1.82E-09	-0.129016899	0.007389362
0.000353897	-0.001433082	0.976361715	-0.312447924	3.43E-11
0.000365458	0.178205593	0.000203714	0.121500877	0.011685235
0.178850702	-0.133249278	0.00565013	0.254100864	9.21E-08
0.108031152	0.067612802	0.161642211	-0.268380416	1.58E-08
0.015996352	-0.139096127	0.003852125	0.16425309	0.000627683
8.77E-05	0.0672543	0.163882463	0.001562842	0.974222065
0.085413009	0.062906317	0.192934912	-0.160231012	0.000854472
2.06E-12	-0.099953541	0.038280515	0.19935338	3.13E-05
0.008435967	0.150987296	0.001690377	-0.076724291	0.112128119
7.47E-05	0.163654971	0.000657439	0.063347177	0.189827894
0.400301177	0.032188409	0.505601188	-0.073823942	0.126394393
0.467129412	0.041444524	0.391290889	-0.227115499	1.95E-06
0.131700447	-0.231548629	1.21E-06	-0.091560482	0.057814952
0.015516791	-0.264939361	2.43E-08	0.01767636	0.714733439
0.001451887	0.093956528	0.051539347	0.060055585	0.213928233
1.47E-12	0.00826222	0.864354897	0.299523039	2.31E-10
0.019901921	-0.195228702	4.58E-05	-0.093816086	0.051890823
0.000101669	0.193741517	5.25E-05	0.410900269	6.03E-19
0.000605821	0.32520546	4.75E-12	-0.059587504	0.217526531
0.043938538	0.079409251	0.100079205	-0.009850474	0.83860816

7.61E-14	-0.013279853	0.78363323	0.161465919	0.000777849
0.017366152	-0.008392672	0.86223436	0.000731479	0.987933164
0.001305539	0.283627126	2.13E-09	-0.163309562	0.000675213
3.20E-11	0.076047981	0.115336071	0.182942519	0.000136307
1.17E-05	0.409968153	7.35E-19	0.161736862	0.000761911
0.389864458	0.071885048	0.136691068	-0.134121355	0.005341222
0.009332362	-0.245306638	2.59E-07	-0.057731241	0.23222289
6.24E-09	-0.024409794	0.613717529	-0.053179078	0.271195504
0.261839934	0.207752022	1.41E-05	-0.303235122	1.35E-10
0.34393591	0.103097651	0.032569236	0.00698863	0.885106249
9.04E-10	0.073469006	0.128232908	-0.147440011	0.00217488
0.596548878	-0.141449823	0.003288266	0.11393295	0.01810874
0.209747342	0.108570494	0.024355701	-0.173260874	0.00030656
4.42E-13	0.102265084	0.034005878	0.402163492	3.80E-18
4.51E-10	-0.01066144	0.825526123	0.144986966	0.002580817
1.47E-06	0.165010347	0.0005918	-0.218763724	4.68E-06
9.71E-23	-0.063800951	0.186668394	0.3886396	5.93E-17
0.08850939	0.018441817	0.702953255	-0.280556789	3.22E-09
6.94E-05	0.186441097	0.000100649	-0.152113353	0.001558649
0.160514708	0.15884831	0.000948508	0.054236259	0.261769173
1.38E-13	0.001545918	0.974501124	-0.124257353	0.009904158
0.09307616	0.215716171	6.38E-06	-0.323300464	6.42E-12
0.264417533	-0.029277923	0.544858455	-0.411305104	5.53E-19
0.749516648	0.034139931	0.480134778	-0.00587684	0.903286257
0.054177089	0.039775745	0.410660345	-0.350787483	6.77E-14
0.240169034	-0.238960133	5.34E-07	0.090658294	0.060336354
0.00106984	-0.144993876	0.002579582	0.1692887	0.000422355
0.493502881	0.059467507	0.218455933	0.539114825	8.64E-34
9.89E-05	0.018173475	0.707075054	-0.233457584	9.84E-07
0.000865988	0.384920382	1.23E-16	0.119103882	0.013458355
0.5772584	0.216198619	6.08E-06	-0.132600628	0.005890211
0.026475041	0.272895143	8.82E-09	-0.066439693	0.169059428
0.047643271	0.523799642	1.14E-31	0.014998099	0.75647021
8.91E-06	0.049443092	0.30634801	-0.429783649	9.30E-21
3.46E-12	0.121663483	0.011572797	0.033336541	0.490533429
2.55E-14	-0.135988669	0.004730331	0.058621739	0.225087295
0.884832783	-0.310632345	4.51E-11	-0.081043044	0.093266257
0.03341312	-0.247369015	2.04E-07	-0.111466732	0.020782978
0.003922199	-0.136527762	0.004566093	0.089153635	0.064743201
0.899273603	0.466580908	1.25E-24	0.136314647	0.004630394
6.98E-09	0.025992382	0.590914415	0.114869769	0.017174557
7.91E-09	0.128880238	0.007452745	0.003667391	0.939556323
0.00360704	0.097547925	0.043203986	-0.252540905	1.11E-07
0.420731141	0.244125866	2.97E-07	0.075267256	0.119128244
0.514185458	0.027790119	0.565493031	-0.256535972	6.86E-08
0.000252503	0.325632806	4.44E-12	-0.177874383	0.000209439
0.003870059	0.207577325	1.43E-05	0.012628618	0.793999397
0.063729934	-0.074205214	0.124442322	-0.022542738	0.641104778
7.97E-16	0.044101124	0.361620131	0.15625065	0.001151462
6.65E-07	0.220278824	4.00E-06	0.026924616	0.577666496
0.002257376	0.109919996	0.022629864	-0.024951936	0.605862392

0.000148384	0.001571988	0.97407127	-0.335780106	8.60E-13
0.086399266	0.170851151	0.00037265	-0.05678909	0.239944992
0.000894909	0.288339038	1.12E-09	0.165911953	0.000551561
0.889008808	0.381083191	2.60E-16	0.037331264	0.440035433
0.019228233	0.29729617	3.18E-10	-0.173681406	0.000296214
8.31E-07	0.145925334	0.002418012	-0.337145466	6.86E-13
0.026950828	-0.006316741	0.896086412	-0.168594082	0.000446376
8.30E-09	0.00233146	0.961552451	0.249161794	1.65E-07
3.78E-06	0.114224761	0.017813105	-0.299119469	2.45E-10
0.062938631	0.565838774	9.21E-38	0.083319638	0.084396851
5.66E-12	-0.344689838	1.93E-13	-0.166315571	0.000534384
4.91E-05	0.048184917	0.31883522	0.338958635	5.08E-13
5.24E-06	0.092106879	0.056330785	-0.093114451	0.053676821
0.000420561	0.072115876	0.135432433	-0.231930726	1.16E-06
3.07E-08	0.241373687	4.07E-07	-0.243165205	3.32E-07
0.051483633	0.033656044	0.486383567	-0.10958479	0.023048353
0.031763013	0.011383423	0.813919866	-0.319220989	1.22E-11
1.28E-07	0.192302754	5.98E-05	0.092491673	0.055304656
0.033577789	0.11789038	0.014443249	-0.256855846	6.60E-08
1.13E-06	0.045699126	0.344468598	-0.114318511	0.017719026
0.904072934	0.072189742	0.135031554	-0.026483347	0.583919976
0.216282669	0.2021615	2.40E-05	-0.030111172	0.533467075
0.004226048	0.11351942	0.018535018	0.227791525	1.82E-06
0.94788415	-0.108701001	0.024183976	0.098457071	0.041283961
0.092039503	-0.101171051	0.035975482	0.034605382	0.474165472
1.83E-07	-0.083816936	0.082552938	0.106608547	0.027067016
0.092983075	0.06934688	0.151130344	-0.316993946	1.71E-11
0.193461199	0.20881772	1.27E-05	-0.137992729	0.004145457
0.179769962	0.032746801	0.498242813	-0.25957168	4.74E-08
0.000639705	0.099908695	0.038367756	0.34187562	3.11E-13
0.203199238	0.153807262	0.001378154	-0.203306849	2.16E-05
0.273769987	0.200063569	2.93E-05	-0.145137552	0.002554035
0.498636664	0.222093341	3.32E-06	-0.299869047	2.20E-10
0.490003573	0.036650974	0.448419132	-0.161051765	0.000802808
0.282311091	-0.111176469	0.021119237	-0.288250582	1.13E-09
0.920248487	0.048358501	0.317092946	-0.3761272	6.73E-16
0.205032211	0.189507637	7.68E-05	0.17623809	0.000240002
0.276347933	-0.028055479	0.561785449	0.231823697	1.18E-06
0.58183766	0.046081641	0.340441029	-0.183589499	0.000128926
0.296283275	0.239682524	4.93E-07	-0.211526171	9.70E-06
1.57E-19	-0.045348775	0.348183976	-0.148170219	0.002065837
6.17E-08	0.1847047	0.000117079	0.115798337	0.016290452
0.00576618	-0.026618546	0.58200066	-0.382925878	1.82E-16
0.015541534	0.055266675	0.252801016	-0.270018554	1.28E-08
1.23E-06	0.068605157	0.155561278	-0.250274525	1.45E-07
0.000847367	0.20991308	1.14E-05	0.285335276	1.69E-09
0.848570471	0.219970961	4.13E-06	0.064559088	0.181476343
1.00E-20	0.094204269	0.050924183	-0.108499163	0.024450004
1.77E-06	0.10703993	0.026449572	-0.087467826	0.069991466
0.384859147	0.0874864	0.069931801	-0.339782329	4.42E-13
0.912769808	0.447181666	1.57E-22	-0.046296782	0.338189037

0.007016378	-0.184588015	0.000118269	0.22769398	1.84E-06
0.000172462	-0.052257776	0.279597095	0.333191524	1.31E-12
2.21E-23	-0.094204707	0.0509231	0.311638678	3.88E-11
0.000503016	-0.050070707	0.300241412	0.1961973	4.19E-05
0.882516758	0.150341494	0.001770452	-0.035316144	0.465129037
0.019009207	-0.201913538	2.46E-05	-0.026594516	0.582341575
0.004770981	0.136585432	0.004548832	0.053844801	0.265232951
0.025189396	0.187682646	9.03E-05	-0.07529285	0.119002402
1.62E-06	0.295472402	4.12E-10	0.215032962	6.83E-06
0.794438826	-0.004114142	0.93220996	-0.249796221	1.54E-07
0.796056055	0.120366032	0.012497196	-0.161737297	0.000761886
0.085787151	0.323076974	6.65E-12	-0.033059121	0.494152033
0.038709574	0.106093626	0.027820224	-0.057819786	0.231506294
0.005784286	0.003566553	0.941215224	-0.220031884	4.11E-06
0.486346119	0.031128907	0.519718226	-0.281488966	2.84E-09
0.287711098	0.029435613	0.54269347	-0.236402104	7.11E-07
0.773997447	0.232156202	1.13E-06	0.360336627	1.25E-14
0.013291738	-0.013494579	0.780223569	-0.177166986	0.000222175
4.65E-05	0.342190917	2.95E-13	-0.010258115	0.832026534
4.51E-10	0.041967925	0.385331542	-0.314981561	2.33E-11
0.001803792	0.192931045	5.65E-05	-0.143566909	0.00284625
0.698733548	-0.071460965	0.139026879	0.106404928	0.027362739
2.74E-11	0.257938292	5.79E-08	-0.141244398	0.003334313
0.648008005	0.053502146	0.268290608	0.003745633	0.938269319
0.003321425	0.027134543	0.574702563	-0.291939865	6.78E-10
2.59E-07	0.038408204	0.426948474	-0.017677654	0.714713465
0.012509187	0.002022384	0.96664616	-0.206161498	1.64E-05
0.022676015	-0.068456811	0.156459148	0.168868347	0.000436743
0.076559745	0.047846365	0.322251217	-0.390743708	3.90E-17
0.120014836	0.141295325	0.003322843	0.041391817	0.391894075
0.000267467	0.105833954	0.028206847	0.422589969	4.70E-20
0.051024989	0.007604969	0.875053164	-0.144049634	0.002753355
1.13E-05	0.277142214	5.06E-09	0.055278641	0.252698146
5.76E-09	-0.074143458	0.124756906	0.124627369	0.009684419
0.075780112	-0.078925532	0.102170467	0.089480154	0.063765058
0.019163401	0.263408711	2.95E-08	0.005445931	0.910346621
4.50E-06	0.021873523	0.651043627	-0.264294282	2.64E-08
0.007621511	0.013817237	0.775107957	-0.379420098	3.59E-16
0.163510172	0.074642713	0.122231337	-0.110185467	0.022303121
0.765523059	0.09183653	0.057061129	-0.357377708	2.12E-14
0.838898823	0.001072973	0.982300472	0.231815778	1.18E-06
0.776859252	0.094976501	0.049045848	-0.225663534	2.28E-06
0.001975768	0.046317686	0.33797073	-0.171686899	0.000348355
0.00332568	0.38404922	1.46E-16	-0.061487999	0.203183045
0.515539101	0.058647966	0.224879524	0.277304978	4.96E-09
4.08E-11	0.12692477	0.008414438	-0.103788461	0.031416838
1.53E-17	0.099508449	0.039153849	0.214839163	6.97E-06
0.33333012	-0.017479839	0.717768927	0.219812508	4.20E-06
0.000492995	-0.002174057	0.964146342	-0.306138406	8.81E-11
0.209197148	-0.158035976	0.001008118	0.185274916	0.000111424
2.30E-08	0.178910697	0.000192009	0.225418087	2.34E-06

0.015638289	0.303216574	1.35E-10	-0.045001738	0.351889138
0.460776284	-0.029312338	0.544385593	-0.290470189	8.32E-10
0.851830905	0.372280787	1.39E-15	-0.230576188	1.35E-06
0.036261213	0.142849333	0.002989615	-0.116359038	0.015776119
0.000150761	0.15987783	0.000877635	-0.145081225	0.002564023
0.283274552	0.101170837	0.035975876	-0.194437482	4.93E-05
0.150188775	0.044670267	0.355451251	-0.242345088	3.64E-07
0.003049386	0.069843954	0.148214972	-0.147301751	0.002196109
2.20E-06	-0.064087266	0.184694881	-0.259465323	4.80E-08
0.628171346	-0.382516455	1.97E-16	-0.000468274	0.992274948
0.399703985	0.130573523	0.006700667	-0.142602016	0.003040529
0.209242144	-0.038431066	0.426673129	-0.209144894	1.23E-05
0.00015431	-0.007466282	0.877313599	0.045774349	0.343674175
0.056368311	-0.00949589	0.84434232	-0.268688056	1.52E-08
0.002352073	-0.071725039	0.137568814	-0.141076561	0.003372368
0.005930869	0.040931176	0.397189609	0.481345292	2.54E-26
0.32712548	0.015074483	0.755269338	-0.046182426	0.339384868
0.274423865	0.022661335	0.63935003	-0.203037073	2.21E-05
2.53E-09	0.155485156	0.00121849	-0.329924068	2.23E-12
0.166084181	0.008976208	0.852761177	-0.320981532	9.24E-12
0.765454772	-0.073037891	0.130493801	-0.217540612	5.30E-06
4.14E-05	-0.077670508	0.107758089	0.202378231	2.36E-05
3.57E-09	0.032200487	0.505441417	0.436648652	1.91E-21
0.008763755	-0.059201303	0.220527882	0.099631269	0.03891119
0.134893002	0.121500527	0.011685478	0.41310548	3.75E-19
0.068286271	0.078874647	0.102392457	-0.267402452	1.78E-08
1.63E-16	0.102335724	0.033881923	-0.094318118	0.050643545
5.36E-06	0.186660036	9.87E-05	-0.011616972	0.810174015
0.073122729	0.003621082	0.940318117	-0.351087644	6.42E-14
0.143413525	0.108967556	0.023836482	-0.260380409	4.29E-08
0.019401057	0.002913563	0.951963538	-0.23674465	6.84E-07
2.85E-05	-0.033283547	0.491223573	0.263217774	3.02E-08
0.950338291	0.344056088	2.15E-13	0.166685656	0.000519071
0.000324202	0.164070587	0.000636627	0.292581446	6.20E-10
1.06E-06	-0.185311454	0.00011107	0.214283127	7.37E-06
0.696677341	0.097221074	0.043912395	-0.105770026	0.028302733
1.84E-10	-0.010518511	0.827828368	0.207530026	1.44E-05
0.000153244	0.040673252	0.400173393	-0.337082997	6.93E-13
1.63E-07	0.267014638	1.87E-08	-0.137793822	0.004200435
0.003665438	0.144584064	0.002653736	-0.046115051	0.340090678
0.002795251	0.176460521	0.000235615	-0.049792881	0.302934567
6.12E-05	-0.046954281	0.331365888	-0.164997703	0.000592383
0.015667353	0.117777221	0.014538241	-0.277415134	4.88E-09
2.02E-10	0.206706345	1.56E-05	0.118297457	0.014106021
0.017378207	-0.007981358	0.868923646	-0.137603944	0.004253531
7.90E-07	0.288638475	1.07E-09	-0.1222717	0.011160666
0.024631686	0.18070973	0.000164936	-0.160665652	0.000826742
0.810483739	0.07500256	0.12043581	-0.252182034	1.16E-07
0.542463625	-0.060213671	0.212722689	0.220860924	3.77E-06
0.06338692	0.160725634	0.000822981	-0.212699342	8.64E-06
0.649346289	0.01142508	0.813251425	-0.339673011	4.50E-13

1.29E-07	-0.020604943	0.670053414	-0.216189599	6.08E-06
3.61E-16	0.052293452	0.279268509	0.10293834	0.032840058
0.001501755	-0.083582283	0.083418903	0.016807087	0.728193761
0.200107412	0.25736928	6.20E-08	0.086482153	0.073217968
0.586720913	-0.165296496	0.000578743	0.215864601	6.28E-06
0.94777442	0.249768482	1.54E-07	-0.270687086	1.17E-08
0.007309132	0.081469246	0.091551378	-0.199046562	3.22E-05
0.548281988	-0.068710543	0.154925797	0.021385169	0.658335771
7.87E-06	-0.018607011	0.70042015	0.194778928	4.78E-05
0.010766499	-0.152716079	0.001492076	0.076712745	0.112182291
0.485515644	0.166370089	0.000532102	0.102111464	0.034276774
0.181947184	0.127302009	0.008220729	-0.238703052	5.50E-07
1.97E-14	0.043418444	0.369107277	-0.15770916	0.00103306
0.169560639	0.276833219	5.27E-09	-0.055342167	0.25215247
0.423888267	0.162155736	0.000737866	-0.235285657	8.05E-07
0.001133763	0.013969464	0.772697814	-0.236804139	6.80E-07
0.988015008	0.23166128	1.20E-06	-0.160850828	0.000815182
2.16E-14	0.055263552	0.252827873	0.187489657	9.18E-05
2.58E-08	0.18235539	0.000143346	-0.259183542	4.97E-08
0.000230804	-0.019540807	0.686164278	0.209150991	1.23E-05
3.42E-06	0.392580276	2.70E-17	0.055511214	0.250704388
5.61E-09	0.423281453	4.03E-20	0.114386268	0.017651301
0.000279553	0.038624519	0.424347353	-0.23310998	1.02E-06
0.014144059	0.182417261	0.000142588	-0.152239417	0.001544503
0.007889267	0.198946894	3.25E-05	-0.007599638	0.875140037
0.617480611	-0.202180823	2.40E-05	0.008592972	0.858980378
0.748357124	0.04238083	0.380669507	0.23754046	6.26E-07
5.30E-08	-0.136362875	0.004615772	-0.316622619	1.81E-11
0.45702982	0.021775973	0.652497632	-0.17224399	0.00033299
4.29E-05	-0.187398501	9.25E-05	0.017362339	0.719585974
0.039360013	0.106964571	0.026556551	-0.266516664	2.00E-08
0.152659183	0.189598614	7.62E-05	0.031440644	0.515543632
3.38E-15	0.071429926	0.139199035	0.218914503	4.61E-06
0.000357217	-0.078031933	0.106124782	-0.334213145	1.11E-12
2.99E-05	0.201890538	2.47E-05	-0.278193613	4.41E-09
2.12E-05	0.209576453	1.18E-05	-0.214498317	7.21E-06
0.485119815	-0.145611742	0.002471347	0.203738797	2.07E-05
4.98E-08	0.284513028	1.89E-09	0.034746344	0.472365704
0.499980841	-0.079259504	0.100722939	-0.380573813	2.87E-16
4.67E-18	-0.068708519	0.154937986	0.011503631	0.811991332
0.000150218	0.126402607	0.008689275	0.179375954	0.000184634
0.49065833	0.043193118	0.371599423	-0.352843815	4.73E-14
1.61E-05	0.042068299	0.384195046	-0.251503946	1.25E-07
0.696214172	0.017174793	0.722489507	-0.300169324	2.11E-10
0.000906815	0.07040798	0.14495899	-0.413297061	3.60E-19
0.509099324	-0.066583384	0.168137476	0.297755664	2.98E-10
1.27E-14	0.066176492	0.170757978	-0.11623198	0.015891405
5.85E-06	0.214710918	7.06E-06	-0.061263893	0.204837814
0.045620676	-0.079655522	0.099027642	-0.329906366	2.24E-12
0.331596854	0.322048407	7.82E-12	-0.21732106	5.42E-06
0.941411554	0.104096493	0.030914329	-0.2511211	1.31E-07

0.153541768	-0.210741339	1.05E-05	0.021124953	0.662234662
0.133162809	-0.059196249	0.220567355	-0.033675356	0.486133336
0.742865343	0.050628796	0.294879675	-0.3172551	1.65E-11
0.012802067	0.098986982	0.040198432	-0.239856544	4.83E-07
0.727194805	0.120442142	0.012441223	-0.187568036	9.12E-05
0.295132393	0.334215194	1.11E-12	-0.097020385	0.044352186
2.27E-12	0.089415909	0.063956548	-0.119361719	0.013256876
1.06E-10	0.317019179	1.71E-11	0.066548728	0.168359495
0.094669684	0.345522662	1.68E-13	-0.229989876	1.44E-06
0.093142422	-0.387860647	6.91E-17	0.006226594	0.897561164
4.95E-06	0.201101227	2.66E-05	0.25740993	6.17E-08
0.863461347	0.023139109	0.632301388	-0.281776284	2.73E-09
0.006468811	0.099686316	0.038802845	-0.266821478	1.92E-08
0.451105098	0.184449619	0.000119695	-0.317179328	1.67E-11
0.547925787	0.039442908	0.414590364	-0.284994771	1.77E-09
1.50E-06	0.140824379	0.003430288	-0.180570783	0.000166893
2.99E-09	-0.097905707	0.042439598	0.226074355	2.18E-06
4.79E-07	0.183893198	0.000125593	-0.365054419	5.31E-15
4.75E-15	0.116947418	0.015251753	-0.187540351	9.14E-05
0.950339816	-0.101685726	0.035037148	0.047823417	0.322483621
1.50E-06	0.185581115	0.000108494	-0.080988814	0.093486288
0.000191575	0.399510571	6.58E-18	0.238264089	5.78E-07
0.26405208	-0.001834904	0.969736589	-0.160284802	0.000850994
0.005044069	0.011467508	0.812570752	-0.177931458	0.000208442
1.08E-08	0.119244025	0.013348513	-0.206713531	1.55E-05
3.36E-09	0.479963876	3.68E-26	0.080546971	0.095294509
4.59E-06	-0.004491469	0.926009668	0.253364449	1.01E-07
1.82E-11	0.249639992	1.56E-07	0.180401875	0.0001693
2.10E-08	-0.025353535	0.600072677	0.39488457	1.69E-17
0.371891513	-0.132443633	0.005949679	0.181923651	0.000148738
0.909500091	0.221305659	3.60E-06	0.27190685	1.00E-08
0.936833234	-0.059918455	0.214977932	0.074212895	0.124403239
0.768812886	0.083169586	0.084959701	-0.255976642	7.34E-08
0.498482305	-0.012586493	0.794671215	-0.185474722	0.000109504
2.11E-10	0.006159873	0.898652907	0.046841401	0.332530942
0.08167899	0.071997876	0.136074733	-0.433799462	3.70E-21
0.001100257	-0.140386184	0.003533081	-0.042523199	0.379070079
5.03E-09	-0.029149671	0.546622417	-0.066146731	0.170950839
0.72948913	-0.107075883	0.026398664	-0.274807964	6.88E-09
0.713012372	0.091217511	0.058762996	0.217821293	5.15E-06
0.398752904	0.122523151	0.010994113	-0.124364847	0.00983987
0.236400433	0.168038637	0.000466492	-0.155189521	0.001245324
0.491205478	0.092800314	0.054492867	0.037234056	0.441227881
0.121921325	0.008151268	0.866159209	0.15252869	0.001512489
0.011128067	0.170480336	0.000383927	-0.00712843	0.8828243
0.266879687	0.006541398	0.892412703	-0.288447396	1.10E-09
9.49E-08	0.317203273	1.66E-11	0.002211837	0.963523726
0.00348104	-0.036023605	0.456230128	-0.290644364	8.12E-10
0.068221395	0.461569321	4.48E-24	-0.218743038	4.69E-06
0.222922613	0.215429714	6.57E-06	0.076234244	0.114445473
0.041786021	0.322122803	7.73E-12	0.05391826	0.264580568

4.49E-14	0.240581441	4.45E-07	0.089898911	0.062528435
0.194809268	0.520922466	2.78E-31	-0.141700828	0.003232786
0.142715399	0.256767053	6.67E-08	-0.123969934	0.010077889
0.33499983	-0.018325291	0.704742075	0.057906923	0.230802618
1.05E-06	0.101538885	0.035302716	0.27411389	7.53E-09
0.044416016	0.190590813	6.97E-05	0.081261686	0.092383343
0.010706762	-0.144838079	0.002607548	0.138081009	0.004121266
0.018777079	0.036177869	0.45430244	-0.247368689	2.04E-07
1.22E-05	0.133714815	0.005483284	0.01463628	0.762166435
0.207361023	0.020664019	0.669163356	-0.372964143	1.22E-15
0.295909787	0.042108122	0.383744721	-0.304672612	1.09E-10
3.61E-31	0.154436694	0.001316131	-0.035255412	0.465897411
9.61E-06	-0.081860648	0.089998854	-0.395851639	1.39E-17
0.002179944	0.064317188	0.183121248	-0.110539441	0.02187381
0.576455374	0.023803586	0.622553507	-0.090878457	0.059712821
0.270658544	0.066470178	0.168863514	-0.356557969	2.45E-14
2.03E-10	-0.042324229	0.381306533	0.07282516	0.131620721
0.001578064	-0.039832685	0.409990239	-0.092253237	0.055938646
0.546719899	0.065567417	0.174737399	0.284269177	1.95E-09
0.033016966	0.274038369	7.60E-09	0.100694441	0.036863372
0.014032023	0.098462847	0.041271995	-0.306915506	7.85E-11
0.061787579	0.179860578	0.000177235	-0.137902921	0.004170199
2.35E-05	0.257113449	6.40E-08	0.136116425	0.004690935
0.954165525	0.137626194	0.004247278	-0.11283487	0.019259917
0.003892565	0.058482921	0.226189257	0.226693895	2.04E-06
9.50E-07	-0.032429305	0.502419736	0.293523534	5.43E-10
0.996325575	-0.10044876	0.037328277	0.328724202	2.71E-12
1.79E-07	0.046679949	0.33420189	-0.416836145	1.67E-19
0.306263124	-0.02402434	0.619329473	0.252382034	1.13E-07
2.29E-06	0.009587332	0.842862775	-0.013993192	0.772322337
0.000281995	0.046284026	0.338322291	0.036729437	0.447447594
0.05173954	0.016822151	0.727959785	-0.029677912	0.539375197
0.114736899	0.061138013	0.205771568	-0.279552725	3.68E-09
1.23E-08	0.229177137	1.57E-06	-0.10557622	0.028595137
0.108810969	0.051323187	0.288298222	-0.217001381	5.60E-06
4.87E-06	-0.057747001	0.232095226	0.413231586	3.65E-19
0.253676975	0.019739909	0.683138794	-0.417826463	1.35E-19
0.238614214	0.28963138	9.35E-10	-0.168258	0.000458449
2.23E-18	-0.082007745	0.089420873	0.164678146	0.0006073
0.865362462	-0.006227796	0.897541502	0.352463171	5.05E-14
0.024211132	0.215705266	6.39E-06	0.115043046	0.017006462
0.002801359	0.003495807	0.942379242	-0.248865866	1.71E-07
0.678221829	-0.008288349	0.863930079	-0.085357779	0.077045467
0.599417263	0.055858725	0.247745752	-0.012115015	0.802200535
1.67E-12	0.01868932	0.699159254	0.238768977	5.46E-07
0.057286467	0.171921609	0.000341803	-0.172320867	0.00033092
0.029964129	0.065543879	0.174892554	0.12279652	0.010815542
0.27865239	0.140951161	0.003401057	-0.346329857	1.46E-13
0.656884353	0.145841653	0.00243214	-0.444299762	3.14E-22
1.45E-06	0.096126076	0.046357263	0.303877855	1.23E-10
0.000784248	0.234899244	8.40E-07	-0.098633367	0.040920102

0.002793097	0.066341903	0.169689033	-0.200350145	2.85E-05
0.000686195	-0.121892046	0.011416369	0.104429856	0.030378259
1.15E-05	0.192752123	5.74E-05	-0.074759068	0.121648495
6.78E-07	-0.003270443	0.946088038	-0.180384228	0.000169553
1.85E-05	0.108700495	0.02418464	0.278755592	4.09E-09
0.428182633	0.065832874	0.172994625	-0.239149835	5.23E-07
0.896396835	0.133588703	0.005528039	0.209554481	1.18E-05
8.54E-11	0.095271469	0.048343844	0.070649636	0.143580797
4.75E-11	0.169409485	0.000418302	0.062093199	0.198762834
0.036687392	-0.064410583	0.182484869	0.109402628	0.023278571
0.001002224	0.076700382	0.112240318	0.017729992	0.713905809
0.988847848	0.070563554	0.144070577	-0.294179183	4.95E-10
9.04E-05	0.105398546	0.028865476	0.260039762	4.47E-08
0.00288228	-0.028397263	0.557027398	0.325682257	4.41E-12
0.012888422	0.102524297	0.033552912	0.257313933	6.25E-08
0.46124883	-0.127957941	0.007893363	0.213995494	7.59E-06
2.02E-06	0.166768656	0.000515693	-0.181104442	0.000159496
0.503915405	0.046205602	0.339142298	-0.120085746	0.012705246
0.188316646	0.008246133	0.864616454	-0.392052133	3.00E-17
2.96E-12	0.000827012	0.986357343	-0.104859484	0.029699141
0.191224315	0.186673684	9.86E-05	-0.186139785	0.000103335
0.163936983	-0.107799592	0.025391712	0.066885912	0.166208693
0.046006893	0.011348463	0.814480953	0.190663441	6.93E-05
1.24E-05	0.151664367	0.00161	0.026520628	0.58339043
0.001289897	0.137064948	0.004407573	-0.211787603	9.45E-06
0.001830611	-0.107502182	0.025801441	0.111043122	0.021275293
0.039492484	-0.139334609	0.003791249	-0.440761772	7.28E-22
0.003249934	-0.217880445	5.12E-06	0.188264994	8.57E-05
1.42E-09	-0.302417529	1.52E-10	-0.069082199	0.152700378
0.300161497	0.034124727	0.480330454	-0.358090446	1.87E-14
0.004434336	0.232083759	1.14E-06	-0.22535727	2.35E-06
0.05215885	0.224506578	2.58E-06	-0.138340016	0.004051025
0.099966025	0.262595015	3.26E-08	-0.177097262	0.000223469
0.938204872	0.197180818	3.83E-05	-0.144262799	0.00271322
0.014411198	-0.301777812	1.67E-10	-0.070127718	0.14656998
0.652564851	0.047712003	0.323613502	-0.150118171	0.001798942
1.11E-05	0.071680809	0.137812202	-0.231709891	1.19E-06
0.000659065	0.233514677	9.78E-07	-0.079276528	0.100649591
3.67E-08	0.091833248	0.057070044	0.273280659	8.39E-09
0.744653942	0.14488526	0.00259905	-0.202429847	2.34E-05
0.000144028	0.273681782	7.97E-09	-0.0444173	0.358184914
0.496802851	-0.03356646	0.487545217	-0.315847813	2.04E-11
1.25E-17	-0.075513221	0.117923165	-0.12174636	0.011515859
0.000795784	-0.243576177	3.16E-07	-0.112863702	0.019228894
0.001172421	-0.183426565	0.000130749	0.157706603	0.001033257
7.52E-06	-0.069816826	0.148372968	0.365987933	4.47E-15
0.001508249	0.283812929	2.08E-09	0.000299562	0.995058115
1.44E-06	0.016335752	0.735527578	0.144877908	0.002600373
0.050592392	0.176271436	0.000239339	0.357832583	1.96E-14
0.375985748	-0.000913543	0.984930064	-0.269460997	1.37E-08
1.84E-06	0.044773398	0.354340542	0.25776393	5.91E-08

0.465774507	0.139325368	0.003793592	-0.170678893	0.00037785
0.069171572	0.160249563	0.000853271	0.131841154	0.006182927
0.091529359	-0.031416919	0.515860733	-0.175773417	0.000249413
0.258826312	0.021903874	0.65059151	-0.084493408	0.080097057
0.37893621	-0.068714289	0.154903245	0.313154328	3.08E-11
0.438778905	0.158521904	0.000972057	-0.321318819	8.77E-12
0.718168971	0.209371941	1.20E-05	-0.170314413	0.000389075
0.00090304	0.072626835	0.132678085	-0.124828304	0.009566914
1.17E-09	0.152874471	0.00147502	0.39030579	4.25E-17
4.99E-08	0.337737258	6.22E-13	-0.118913708	0.013608684
0.000848675	0.022455359	0.642398898	-0.337840293	6.11E-13
0.000372475	0.133505866	0.005557614	0.290603318	8.17E-10
1.31E-05	0.278354414	4.31E-09	-0.263232975	3.01E-08
0.002925704	0.245504562	2.53E-07	-0.230838321	1.31E-06
0.000149593	0.07789854	0.106725302	0.205846164	1.69E-05
0.20196945	0.126883966	0.008435632	0.206416657	1.60E-05
0.664413035	-0.091419555	0.05820296	0.155994572	0.001173497
1.97E-08	0.153584396	0.001400751	0.216516377	5.88E-06
0.503890836	0.191243226	6.58E-05	0.165545498	0.0005676
0.102519448	0.132599197	0.005890751	-0.091048591	0.059234634
1.78E-18	0.180980154	0.000161191	-0.207865024	1.39E-05
0.000152049	0.061938358	0.199887025	0.28033668	3.31E-09
0.024914021	0.021250378	0.660354235	0.243140699	3.33E-07
7.41E-08	-0.163353338	0.000672936	0.052740177	0.275176216
0.019342292	0.07852257	0.103938927	-0.069525491	0.150077809
1.69E-05	0.05013071	0.299661852	0.061578204	0.202519726
0.885162352	0.190192473	7.23E-05	0.307271115	7.45E-11
2.54E-09	0.038034603	0.431462656	0.086363758	0.073613552
0.492562746	0.036144143	0.454723488	-0.210860217	1.04E-05
2.41E-16	0.095048986	0.048872554	0.239787422	4.87E-07
0.339943153	0.140528437	0.003499409	-0.130101482	0.006903175
9.80E-15	-0.024976886	0.605501974	0.058036518	0.229758888
0.000269949	0.088043403	0.06816178	-0.317050728	1.70E-11
0.105350005	0.410247901	6.93E-19	-0.135903501	0.00475676
0.196255711	0.235428784	7.92E-07	0.036186161	0.454198956
8.46E-12	0.031893124	0.50951531	-0.269320522	1.40E-08
6.29E-16	0.22409024	2.69E-06	-0.106270393	0.02755965
1.78E-09	0.046323924	0.337905607	0.284101118	2.00E-09
0.93433067	0.139151055	0.003838026	-0.407310671	1.29E-18
0.059666228	0.047167979	0.329167495	-0.246872637	2.16E-07
9.66E-07	0.048950864	0.311194305	-0.482588494	1.81E-26
0.004856006	-0.06619999	0.170605819	-0.162940453	0.000694698
1.30E-09	0.059142519	0.220987301	0.192892385	5.67E-05
0.002296054	0.001227486	0.979752175	0.140938073	0.003404064
3.38E-08	-0.077788447	0.107222947	0.101505721	0.035362929
9.80E-06	0.274618148	7.05E-09	-0.074781955	0.121534103
0.585780875	0.10590955	0.02809382	-0.07650458	0.113162493
0.183323425	0.387875608	6.89E-17	-0.259211951	4.95E-08
9.60E-05	0.452373049	4.44E-23	-0.070239478	0.145925939
0.004932026	0.007588382	0.875323459	-0.125616985	0.009117854
0.085834848	0.096321438	0.045912881	-0.181253673	0.000157484

0.945019489	0.071287548	0.139990821	0.056165525	0.245154039
0.007741828	0.400705844	5.14E-18	-0.18543883	0.000109846
0.026187889	0.164398736	0.000620628	-0.223337483	2.91E-06
0.436059123	-0.321269969	8.83E-12	-0.024130969	0.617774785
0.886638621	0.092439394	0.055443149	-0.116855536	0.015332617
0.653011843	0.026236038	0.587438415	0.264967046	2.43E-08
0.031112725	0.441797878	5.70E-22	0.137745618	0.004213858
0.028710022	0.036397786	0.451562277	-0.232060274	1.15E-06
0.119656988	0.003353172	0.944726428	-0.242147672	3.73E-07
0.731324518	-0.000698444	0.988478074	-0.424191692	3.29E-20
0.139221296	0.024405032	0.613786719	-0.429704653	9.47E-21
0.00044339	0.37010159	2.09E-15	-0.016900264	0.726746875
1.60E-13	0.168940142	0.000434254	-0.067284827	0.163690796
1.20E-11	0.00928535	0.847750987	0.291457817	7.25E-10
0.053374041	0.137588886	0.004257768	-0.143141328	0.002930503
9.41E-09	0.048561434	0.315064001	-0.299031804	2.48E-10
6.33E-06	0.019503261	0.686735378	-0.324173024	5.60E-12
3.94E-05	-0.130588069	0.006694513	-0.259767051	4.63E-08
0.14503712	0.018511156	0.701889601	0.25389487	9.44E-08
0.050651276	-0.009722141	0.840682533	0.184128751	0.000123063
2.18E-06	0.064902403	0.179160622	-0.136374375	0.004612291
0.282612038	0.000145358	0.997602014	0.252326115	1.14E-07
0.522320825	0.226507461	2.08E-06	-0.04676989	0.333270382
0.987455495	0.050220881	0.29879231	0.046886391	0.33206628
0.908089003	0.005659529	0.906845939	-0.337374315	6.60E-13
0.146725476	0.193991267	5.13E-05	-0.358413441	1.76E-14
0.001660473	0.092409576	0.05552227	-0.125222137	0.009340274
0.000970382	-0.005394709	0.911186372	-0.247231376	2.07E-07
2.27E-11	-0.038332175	0.427864904	0.29099378	7.74E-10
0.028963221	0.035689936	0.460415299	-0.346297342	1.47E-13
0.126978583	0.079814369	0.098354051	0.454125895	2.88E-23
0.388778086	-0.129144803	0.007330476	-0.189533847	7.66E-05
0.578254975	-0.33352173	1.25E-12	0.034547105	0.47491063
2.68E-11	-0.065869702	0.172753864	0.251083094	1.32E-07
0.210601715	0.110578395	0.021827007	-0.217160108	5.51E-06
0.618648901	-0.341637749	3.24E-13	0.045333559	0.348345914
0.041593051	-0.080636833	0.094924508	0.087875518	0.068691371
0.724428237	0.070636112	0.143657662	0.263616281	2.87E-08
0.000596535	0.045222303	0.349531386	0.047841751	0.322297933
0.018984997	0.204813161	1.87E-05	0.026921123	0.577715869
3.07E-07	-0.073991393	0.125534159	-0.150123857	0.001798211
1.55E-06	0.132632453	0.005878221	-0.245715925	2.47E-07
6.95E-05	0.130161349	0.006877192	-0.265067893	2.40E-08
0.311863687	-0.206762898	1.55E-05	0.108228443	0.024810788
0.032526187	0.173063856	0.000311523	-0.200944893	2.70E-05
0.001234213	0.243536254	3.18E-07	-0.112564101	0.019553388
8.55E-05	-0.048771544	0.312972278	0.103272775	0.032273736
0.001171098	-0.010186161	0.833187441	-0.239687177	4.92E-07
3.47E-10	-0.053788539	0.26573335	0.068721051	0.154862542
0.002005824	-0.313387599	2.97E-11	-0.181073709	0.000159914
0.135493913	0.343662131	2.30E-13	0.235975465	7.46E-07

0.333915092	0.212782023	8.56E-06	-0.306400669	8.47E-11
0.133799077	0.086713463	0.072450113	-0.014640355	0.762102207
0.01530626	0.227878497	1.80E-06	-0.219239112	4.45E-06
0.3561392	0.044587424	0.356345038	-0.271412593	1.07E-08
4.29E-14	-0.46571698	1.56E-24	-0.094845006	0.049361533
2.63E-05	-0.001311254	0.978370723	0.108285861	0.024733887
5.01E-07	0.258619956	5.33E-08	-0.167626981	0.000481941
0.566411263	-0.026285654	0.586731753	-0.13150051	0.006318407
2.53E-07	0.003312328	0.945398646	-0.193455527	5.39E-05
0.836086375	-0.057311451	0.235641599	-0.064619692	0.181065962
0.001182805	0.019064035	0.693429436	-0.30468931	1.09E-10
0.095803998	0.316561713	1.83E-11	-0.147768027	0.002125262
0.002282188	0.360203849	1.28E-14	0.214007105	7.58E-06
6.53E-08	0.089115612	0.064857902	0.153904652	0.001368385
1.36E-05	0.264980143	2.42E-08	-0.162397251	0.000724322
0.01467617	0.104993471	0.029490025	-0.166927989	0.000509266
0.214201264	0.033140772	0.49308552	-0.250531863	1.41E-07
0.001903238	0.360655615	1.18E-14	0.133030349	0.005730159
3.67E-15	0.223393731	2.89E-06	0.086689591	0.072529055
0.588952813	0.038224914	0.429159715	-0.145766678	0.002444863
2.33E-08	0.135972796	0.004735247	-0.255986855	7.34E-08
0.448893197	0.050498505	0.296125665	-0.303372509	1.32E-10
2.09E-10	0.057007045	0.238142741	0.293924814	5.13E-10
0.582623865	0.074974958	0.120572804	-0.153195298	0.00144102
1.21E-16	0.007075934	0.883681081	0.096090821	0.046437839
0.000129583	0.172619397	0.000322995	0.009235415	0.848559871
3.45E-06	-0.104880718	0.029665915	0.121717182	0.011535876
9.24E-08	0.046515198	0.335912534	0.301023908	1.86E-10
0.62175465	0.098460191	0.041277497	-0.102924058	0.032864431
2.33E-06	-0.072821889	0.131638107	0.176617373	0.000232567
0.009911818	0.084005461	0.081862474	0.15677432	0.001107582
0.069997786	0.231569295	1.21E-06	-0.005088669	0.916205678
2.69E-06	0.150501023	0.001750355	-0.130594517	0.006691786
0.134617351	0.066666842	0.167603719	-0.009745787	0.840300233
0.005497612	0.127328142	0.008207458	-0.254000795	9.32E-08
0.97580123	0.184778673	0.00011633	-0.244409969	2.87E-07
0.168489987	0.05226217	0.279556616	-0.249718517	1.55E-07
5.87E-12	0.159697929	0.000889656	0.010450101	0.828930824
1.51E-13	-0.008434438	0.86155566	-0.250843132	1.36E-07
6.30E-07	0.012218171	0.800551589	-0.153310642	0.001428973
1.52E-10	0.068748548	0.154697106	-0.034268678	0.478479555
0.470545532	0.048295645	0.31772312	-0.084054388	0.081684049
0.320595477	0.145687841	0.002458306	-0.255251437	8.02E-08
0.039204563	0.328636696	2.75E-12	-0.032916877	0.496012933
0.162936922	-0.07167267	0.137857025	-0.035071193	0.468232447
7.12E-16	-0.022038906	0.648581589	0.07857322	0.10371532
0.287437147	0.377598696	5.09E-16	-0.212846053	8.51E-06
0.000533627	0.226302115	2.13E-06	0.222428945	3.20E-06
0.802821738	0.278444275	4.26E-09	-0.311721162	3.83E-11
8.92E-09	0.10034238	0.037531121	-0.067332944	0.163389036
0.406933576	0.090425614	0.061001175	-0.33423114	1.11E-12

1.66E-06	0.374274302	9.56E-16	0.078442086	0.104295029
2.58E-22	0.146494861	0.002323825	-0.070563097	0.14407318
0.000135052	0.074444415	0.123229652	-0.212891898	8.47E-06
0.00104953	0.169790247	0.000405764	-0.158543648	0.000970472
0.657994177	0.096033899	0.046568184	-0.021460334	0.657211274
0.088101888	-0.121316573	0.011813843	-0.216248425	6.04E-06
1.12E-08	-0.320031122	1.07E-11	0.155729434	0.00119672
0.1443726	0.119062294	0.013491104	-0.247544326	2.00E-07
0.021114466	-0.034831453	0.471280875	-0.29888065	2.54E-10
0.042214889	0.305865657	9.17E-11	-0.021669125	0.654091723
2.42E-05	0.209910309	1.14E-05	-0.299326061	2.38E-10
0.224587618	0.05721591	0.236424607	-0.036341058	0.452268223
0.034578821	-0.099028953	0.040113493	0.148531202	0.002013798
0.147124546	0.285339982	1.69E-09	0.000476786	0.992134532
0.002338595	-0.030240984	0.531703275	0.087946864	0.068465899
1.85E-07	0.007518413	0.876463808	0.389421634	5.07E-17
0.26166773	-0.115091839	0.016959389	0.131324128	0.006389598
0.03657941	0.095876512	0.046930181	-0.24556353	2.52E-07
0.013200136	0.169162709	0.00042662	0.326323013	3.98E-12
0.601657458	-0.070540749	0.14420055	0.105083553	0.029350142
0.00318257	-0.002903406	0.952130801	-0.392316887	2.84E-17
1.49E-14	0.093657275	0.052290675	0.217538827	5.30E-06
0.918428966	0.113361157	0.018700462	-0.014960735	0.757057839
5.63E-05	0.006825378	0.887772222	-0.442373141	4.97E-22
0.000877307	0.145036825	0.002571921	0.008427222	0.861672916
0.186544991	0.031764825	0.511220869	-0.275673901	6.14E-09
0.117636742	0.331305079	1.79E-12	-0.181716489	0.000151392
1.08E-15	0.394988673	1.66E-17	0.087840693	0.068801646
0.106031731	0.082444786	0.08772118	0.22088571	3.76E-06
0.441739041	0.026448358	0.584417166	0.120811088	0.012173015
0.001334734	0.125355706	0.009264499	-0.065914967	0.1724583
0.060261281	-0.043675701	0.366274659	0.184520535	0.000118962
6.53E-07	0.125421033	0.009227639	0.320435951	1.01E-11
0.091299843	0.175300008	0.000259355	0.249650668	1.56E-07
0.038658849	-0.022251844	0.645417245	-0.407914084	1.14E-18
0.919375899	0.154641809	0.001296481	0.376224303	6.61E-16
0.000854731	0.143824149	0.002796398	0.32617784	4.07E-12
1.14E-15	-0.001332592	0.978018827	-0.082461609	0.087656278
0.120284445	0.117616213	0.014674347	-0.107328713	0.026043042
0.002279269	0.120818	0.01216804	0.213844109	7.70E-06
8.79E-07	0.053405794	0.269154732	-0.367654489	3.29E-15
0.000252491	-0.106079467	0.027841187	0.103607065	0.031716014
0.977045887	0.068999495	0.15319349	0.048429727	0.316379855
3.37E-07	-0.081713443	0.09058026	0.228461605	1.69E-06
0.850046203	0.069283081	0.15150766	-0.295212671	4.28E-10
0.527087739	-0.05256217	0.276801965	-0.381771698	2.28E-16
0.039414689	-0.027792817	0.565455285	0.048779325	0.312894989
1.41E-06	0.085269322	0.077353355	0.308598575	6.11E-11
0.001093005	0.081912627	0.089794276	-0.002614005	0.95689735
1.46E-05	0.013552034	0.779311932	-0.060831617	0.20805723
0.000137687	0.117873858	0.014457085	-0.173737523	0.000294858

4.58E-07	-0.275207946	6.53E-09	-0.09552924	0.047737272
1.10E-15	0.046440607	0.336688869	0.050893994	0.292354375
1.74E-07	0.062940328	0.192693899	0.213027719	8.36E-06
0.113060831	0.034305926	0.478001253	-0.046180489	0.33940515
0.849190098	0.274178519	7.47E-09	0.270261761	1.24E-08
0.700984997	0.034295524	0.4781348	-0.02763257	0.567699825
0.460529872	0.053788187	0.26573648	-0.175563048	0.000253786
0.065171137	-0.395033862	1.64E-17	0.084348828	0.080616909
0.261783498	0.368702147	2.71E-15	0.011439015	0.813027841
2.81E-15	-0.002633226	0.956580722	-0.282182545	2.59E-09
2.57E-16	0.089838095	0.062706795	0.381591378	2.36E-16
0.586279227	-0.048354549	0.317132548	0.194923987	4.71E-05
1.22E-22	0.151652083	0.001611426	-0.177272768	0.000220226
0.214729702	0.132378099	0.005974661	-0.156563706	0.001125041
0.001503421	0.394761356	1.74E-17	0.082812928	0.086309682
2.69E-11	0.14043175	0.003522263	-0.123989512	0.01006597
0.742886593	0.16284009	0.000700086	0.31408351	2.68E-11
1.52E-06	0.120605362	0.012321933	0.004158011	0.931488874
0.996862999	0.066700002	0.167391997	0.110423592	0.022013521
0.221108878	0.091146996	0.058959501	0.408818396	9.39E-19
0.808324997	0.004784771	0.921193161	-0.336756076	7.32E-13
6.18E-06	0.315196435	2.26E-11	-0.179221955	0.000187046
0.000277583	0.055384582	0.251788593	-0.4324435	5.06E-21
0.272201714	0.211804108	9.44E-06	-0.234680059	8.60E-07
1.22E-20	0.142165722	0.00313227	-0.063572717	0.188252646
0.55115982	0.047428656	0.326498583	-0.305226223	1.01E-10
0.534191456	-0.095216298	0.048474504	0.104409869	0.030410174
0.132174097	0.049612072	0.304695846	-0.251701722	1.23E-07
0.800740729	0.103839783	0.031332632	0.501830574	8.28E-29
0.06412987	-0.088878973	0.06557552	0.09617591	0.046243566
5.46E-07	0.273578666	8.07E-09	-0.237006566	6.65E-07
0.352305486	0.154041481	0.001354767	-0.158047638	0.001007238
0.752442358	-0.117921169	0.014417497	-0.27494694	6.75E-09
0.000670316	0.110812377	0.021547697	-0.316041332	1.98E-11
7.41E-09	0.064622533	0.181046738	0.203858393	2.05E-05
0.599993395	0.085885762	0.075228384	-0.205391437	1.77E-05
7.54E-06	0.068044809	0.158973342	0.241315289	4.09E-07
3.07E-06	0.035885546	0.457959188	0.012040255	0.803396137
1.25E-12	-0.018332244	0.704635279	-0.19071466	6.90E-05
0.942206223	-0.002106013	0.965267775	-0.330868467	1.92E-12
0.311804141	0.134854046	0.005093528	-0.189662442	7.58E-05
0.001719371	-0.055955293	0.246927938	0.064319849	0.183103092
0.002836149	-0.007747802	0.872726226	0.164853544	0.00059907
0.01788053	0.167796482	0.000475523	0.254553163	8.72E-08
0.039199886	0.134263809	0.005292231	-0.299892313	2.19E-10
9.81E-10	0.212519657	8.79E-06	-0.101651896	0.03509818
0.005600905	-0.241406636	4.05E-07	0.031754801	0.511354253
0.144941518	0.105917613	0.028081786	-0.164112942	0.000634541
0.432267158	0.308035851	6.65E-11	-0.042275207	0.381858781
0.457550968	0.520148223	3.53E-31	0.047713811	0.323595153
0.004126644	0.036049785	0.455902659	0.019887765	0.680895311

0.001420226	0.087313654	0.070488322	0.269102485	1.44E-08
0.116129363	0.040349358	0.403939311	-0.227358916	1.90E-06
0.330310596	-0.101131169	0.036049075	-0.152974423	0.001464349
0.00013537	0.078643316	0.103406488	0.072029579	0.13590194
0.815343615	0.201443367	2.57E-05	-0.031344706	0.516826527
0.526212879	0.494559169	6.61E-28	0.071028899	0.141438032
8.63E-22	-0.051056372	0.290815325	-0.112233379	0.019917113
0.171305965	0.076634458	0.112550139	0.177405976	0.000217793
7.59E-10	-0.152477624	0.001518096	-0.307052074	7.69E-11
4.72E-05	0.244603369	2.81E-07	-0.172636255	0.000322553
3.98E-09	0.096296516	0.045969371	0.139417539	0.003770286
0.046846031	0.148177905	0.002064716	-0.12721194	0.008266613
1.50E-08	0.194703624	4.81E-05	-0.141328694	0.003315347
5.62E-05	0.036162376	0.454495841	0.079319459	0.100464808
1.21E-14	-0.019364408	0.688848957	0.159578813	0.000897699
1.97E-14	0.058143981	0.228895935	0.166205906	0.000539001
0.499788474	-0.171707999	0.000347762	-0.092125518	0.056280717
1.75E-16	-0.079573086	0.099378657	-0.360632539	1.18E-14
0.170988865	0.18878513	8.19E-05	-0.016028647	0.740319043
3.04E-17	0.041569433	0.38986365	-0.21406864	7.53E-06
4.64E-05	0.034852523	0.47101253	-0.063901467	0.185973796
0.313432532	-0.012454098	0.796783614	0.167217654	0.000497772
1.07E-05	0.075206882	0.119425504	0.004239061	0.930156805
0.000343933	-0.202111451	2.42E-05	0.152528922	0.001512463
1.98E-05	0.016207692	0.737524336	0.340993221	3.61E-13
2.39E-06	0.201365267	2.59E-05	0.051465781	0.286959018
0.93012771	0.088673845	0.066202847	-0.165977274	0.000548747
0.179191429	0.002971522	0.951009149	-0.155149698	0.00124898
0.001209164	0.241100209	4.20E-07	0.195323025	4.55E-05
0.205637594	0.000269913	0.995547232	-0.07200333	0.136044995
0.02320558	0.156955306	0.001092779	-0.01155077	0.811235373
0.393915032	0.290536466	8.25E-10	-0.054966828	0.25538842
0.015026645	0.005051286	0.916819016	-0.215703294	6.39E-06
0.218244355	0.068433563	0.156600212	-0.316700373	1.79E-11
4.09E-07	0.086345843	0.073673561	-0.058508011	0.225989808
2.29E-22	-0.113773498	0.018272083	-0.334754491	1.02E-12
1.75E-10	0.039785351	0.410547252	0.153881363	0.001370715
0.885687728	-0.017845993	0.712116867	-0.230730803	1.32E-06
0.011571114	-0.002957787	0.951235306	-0.327975524	3.06E-12
2.98E-05	0.306003163	8.99E-11	-0.171224422	0.00036161
0.000207283	0.310275813	4.76E-11	-0.006646141	0.890700667
0.003661038	0.527254132	3.88E-32	0.069364853	0.151024179
0.085087829	-0.346744582	1.36E-13	-0.1057619	0.028314942
0.38740193	0.029240736	0.545369629	-0.327685326	3.20E-12
0.001306646	0.158118061	0.001001941	0.168007325	0.00046765
0.218384091	0.330930558	1.90E-12	-0.139667205	0.003707805
2.98E-06	0.07471174	0.121885303	0.249636073	1.56E-07
2.98E-06	-0.128014916	0.007865485	0.029680344	0.539341932
0.021437301	0.038838885	0.421778802	-0.194553513	4.88E-05
2.92E-17	-0.03132635	0.517072175	0.368317765	2.91E-15
0.001096382	0.240796046	4.34E-07	-0.23142136	1.23E-06

0.362829629	0.008764894	0.856189299	-0.086097621	0.074509133
4.81E-08	0.00268606	0.955710425	0.108922334	0.02389513
0.001979535	0.17148459	0.000354097	0.041035028	0.395991977
0.00088631	-0.215281557	6.67E-06	-0.183174426	0.000133617
1.55E-09	-0.02594334	0.59161518	0.058156146	0.228798397
7.50E-10	0.170166703	0.000393712	0.01472549	0.760760764
0.005731309	0.037190319	0.441765001	-0.127525519	0.008107843
0.002918264	0.264171207	2.68E-08	0.245254159	2.61E-07
0.000176327	0.259492732	4.79E-08	0.1461634	0.002378225
0.465580102	0.375176953	8.06E-16	-0.143578783	0.002843931
0.026490464	-0.021425933	0.657725821	-0.458636543	9.38E-24
5.62E-19	0.038375392	0.427343847	-0.087084536	0.071232033
0.001069009	-0.140221561	0.003572415	-0.230776	1.32E-06
0.706071216	0.325030009	4.89E-12	-0.110053529	0.022464996
0.024864257	0.005319219	0.912424152	-0.223245584	2.94E-06
5.31E-06	-0.127177565	0.008284185	-0.131440512	0.006342543
0.702889339	-0.005520909	0.90911759	-0.24866245	1.75E-07
3.39E-06	0.043651289	0.366542876	-0.39968421	6.35E-18
6.65E-13	0.151265765	0.001656884	-0.112934988	0.01915238
0.012195801	0.052451643	0.277814685	-0.378987003	3.90E-16
0.247628426	0.016499779	0.732972606	0.324439456	5.37E-12
7.21E-06	0.212122145	9.15E-06	0.422299977	5.01E-20
2.01E-23	-0.026066979	0.589849206	0.155832801	0.001187617
8.53E-05	-0.245507523	2.53E-07	-0.03160903	0.513295944
0.007701458	0.066106463	0.171212044	-0.172452612	0.0003274
0.126200822	0.035369745	0.464451461	-0.133734493	0.00547633
0.129589622	0.086038062	0.074710763	-0.344597027	1.96E-13
1.42E-21	0.082219538	0.08859392	0.148437504	0.002027189
3.11E-10	0.020539768	0.671035916	0.163895964	0.000645296
9.98E-05	0.063447368	0.189126938	-0.033188827	0.492458402
0.128920017	0.071820064	0.137047022	0.303159088	1.36E-10
0.045629896	0.203510545	2.12E-05	0.013153658	0.785639046
0.167148323	-0.242286474	3.67E-07	0.015030665	0.755958142
0.101965768	0.484647171	1.04E-26	0.08388009	0.082321116
0.000258828	-0.130282709	0.006824789	0.321278346	8.82E-12
5.62E-09	0.397690866	9.56E-18	0.278227504	4.39E-09
0.811159497	0.001007227	0.98338484	0.271750906	1.02E-08
0.563641645	0.031580377	0.513678057	-0.345471342	1.69E-13
0.037953491	0.038846108	0.421692409	-0.24226936	3.67E-07
0.027524611	0.096775206	0.044894495	0.192852014	5.69E-05
4.05E-21	0.298985735	2.50E-10	-0.025656131	0.595726789
5.05E-06	-0.057343369	0.235380422	0.135457214	0.004897439
0.689102358	0.147503274	0.002165229	-0.32363817	6.09E-12
0.992973938	-0.058031786	0.229796934	-0.237287404	6.44E-07
1.71E-05	-0.005403267	0.911046064	0.161901933	0.00075235
0.023823144	-0.272782328	8.95E-09	-0.112121278	0.02004173
0.006611738	0.048521073	0.315466854	-0.153184069	0.001442198
2.62E-15	-0.038550861	0.425232029	0.051413621	0.287448404
7.46E-07	0.066640748	0.16777047	-0.353161608	4.47E-14
5.15E-10	0.117549105	0.014731405	0.10490014	0.029635555
0.613168911	0.122004949	0.011339791	-0.124731848	0.009623162

0.000432001	0.122832479	0.010792245	-0.157105154	0.001080661
2.75E-07	-0.139651152	0.003711794	0.058364869	0.227129394
0.000463952	-0.303759698	1.25E-10	-0.126572479	0.008598999
0.863846118	-0.108691496	0.024196447	0.243605503	3.15E-07
0.943387823	0.387508587	7.41E-17	-0.118857642	0.013653284
0.008166196	0.108608148	0.024306047	-0.144209666	0.002723174
0.009047134	0.152609199	0.001503688	-0.238336283	5.73E-07
0.4527565	0.263301097	2.99E-08	-0.204296895	1.96E-05
0.279493004	-0.075631635	0.117346421	0.303985025	1.21E-10
0.87370611	0.194798387	4.77E-05	-0.350834849	6.71E-14
0.182871185	0.093294995	0.053212432	0.071345542	0.139667891
9.99E-07	0.056576422	0.241712734	-0.093481019	0.052737451
7.38E-05	0.028331058	0.557947521	-0.377828804	4.87E-16
2.88E-08	0.059427158	0.218769085	0.226280242	2.13E-06
3.80E-09	0.043054013	0.373143135	0.100531211	0.037171703
5.07E-13	-0.102426636	0.033722965	0.2840627	2.01E-09
0.02822364	0.046272883	0.33843873	0.220038048	4.10E-06
6.70E-05	-0.119027522	0.013518539	-0.020603783	0.670070902
0.264916095	-0.062117383	0.19858767	0.052932453	0.273427465
0.436862507	0.351407036	6.08E-14	0.112564178	0.019553304
0.76199852	0.074202475	0.124456261	-0.269506127	1.36E-08
5.49E-05	-0.336685999	7.40E-13	0.038911218	0.420914144
0.317501656	-0.126900558	0.008427008	0.360917503	1.13E-14
0.010535123	-0.169893791	0.000402415	0.098534891	0.041123012
0.730262231	0.108614285	0.024297962	0.272220216	9.63E-09
0.274491157	-0.279772235	3.57E-09	0.101668474	0.035068261
0.14076251	0.094326121	0.050623868	-0.370170763	2.06E-15
1.10E-05	0.062712126	0.194315244	0.142310688	0.003101512
5.23E-06	0.045258143	0.349149222	0.151259232	0.001657662
0.315349464	-0.009482475	0.844559422	-0.313871687	2.76E-11
0.000130769	-0.033629071	0.486733172	-0.356364039	2.54E-14
0.313792693	0.12700854	0.008371075	-0.247896316	1.92E-07
1.81E-05	0.251126485	1.31E-07	0.299208029	2.42E-10
0.874015312	0.059422774	0.218803129	0.137912539	0.004167543
0.001641228	0.225897188	2.22E-06	-0.064717506	0.180405055
0.12843295	0.215513219	6.51E-06	-0.098862127	0.040452002
1.23E-13	-0.248284943	1.83E-07	0.115068583	0.016981811
0.145861316	0.173447649	0.000301924	-0.189480566	7.70E-05
0.841048392	0.053446709	0.268787561	0.017784478	0.713065337
0.002081077	0.044324131	0.359195052	-0.259056016	5.05E-08
0.298504681	0.036585331	0.449232853	-0.275063869	6.65E-09
1.07E-08	0.108661226	0.024236202	0.432388587	5.13E-21
0.000212408	0.185045785	0.000113665	-0.203920312	2.03E-05
0.001260763	0.135487456	0.004887789	0.031563182	0.513907448
0.001392022	0.005602294	0.907783789	-0.470523718	4.49E-25
1.42E-06	0.129677944	0.007089519	0.020615998	0.66988682
0.443758549	-0.023815947	0.622372783	-0.313920853	2.74E-11
0.000327716	0.110544321	0.021867943	0.074828675	0.121300857
9.21E-11	0.075469623	0.118136069	-0.172079039	0.000337472
0.733047485	-0.020332419	0.674165376	-0.084424229	0.080345456
0.139229041	0.1710513	0.000366692	-0.283077497	2.29E-09

0.846782257	-0.128926475	0.007431246	-0.349551909	8.39E-14
3.26E-10	0.212808289	8.54E-06	-0.260778925	4.09E-08
0.049197379	0.02689661	0.578062451	-0.068429738	0.156623429
4.21E-09	-0.009962988	0.836790396	0.17975678	0.000178796
5.63E-09	0.226954679	1.99E-06	-0.177965554	0.000207848
0.035722389	0.033249401	0.491668525	-0.084264822	0.080920215
0.248825419	0.267146294	1.84E-08	0.272549385	9.23E-09
0.000753684	0.215992166	6.20E-06	-0.259628974	4.71E-08
6.15E-16	0.002257856	0.962765349	-0.026765651	0.579915662
0.09840155	0.261119304	3.92E-08	-0.099796148	0.038587438
0.058475265	0.436538893	1.96E-21	0.041947159	0.385566923
1.22E-05	0.011224172	0.816476502	0.121089505	0.011974011
0.162219397	0.014294695	0.767555922	0.415287964	2.34E-19
0.124931409	-0.238953255	5.35E-07	-0.071034264	0.1414079
0.000471704	0.045940726	0.341921232	-0.221539482	3.51E-06
5.47E-05	-0.03400004	0.481936803	0.287936335	1.18E-09
0.096297766	0.137139723	0.004385906	-0.152216298	0.001547089
0.470624652	0.200288816	2.87E-05	0.078000201	0.106267392
0.190337107	0.181814729	0.000150128	0.323081402	6.65E-12
0.034396719	0.079209964	0.100936625	-0.267511293	1.76E-08
0.341660364	0.130464768	0.006746846	-0.330556213	2.02E-12
1.04E-05	0.166835733	0.000512979	-0.292605143	6.18E-10
4.88E-10	-0.015838521	0.743290437	-0.439800163	9.13E-22
1.54E-07	0.058649843	0.224864659	0.119103982	0.013458277
1.26E-09	-0.00400927	0.933933972	-0.086901205	0.071831728
0.781554022	0.341371375	3.39E-13	0.049875914	0.302127999
0.413163784	0.088392474	0.067071346	0.35243104	5.08E-14
0.000193041	0.338635557	5.36E-13	-0.009396783	0.84594652
2.11E-07	0.208565309	1.30E-05	0.215257752	6.68E-06
0.63241663	0.042910045	0.374744961	-0.069022307	0.153057352
7.81E-15	-0.081141175	0.092869153	0.362090134	9.10E-15
0.072418271	0.037284512	0.440608711	-0.127481174	0.008130129
0.611309069	0.092471671	0.055357611	-0.25317963	1.03E-07
0.102678743	0.086568855	0.072929379	0.21849846	4.81E-06
0.099191498	0.483457304	1.43E-26	-0.121835641	0.011454798
1.63E-06	0.148861498	0.001967236	-0.145909837	0.002420622
8.41E-07	0.306673168	8.14E-11	0.069040843	0.152946808
0.033389526	0.065359395	0.176112203	-0.255143745	8.12E-08
0.099022675	0.119703055	0.01299424	0.340320038	4.04E-13
0.344781695	0.129529134	0.007156052	-0.065356677	0.176130216
0.008243835	0.078593234	0.103627065	-0.203894548	2.04E-05
0.272406318	0.302948263	1.41E-10	0.037494601	0.438035936
0.187137328	0.123888919	0.010127345	-0.317839946	1.50E-11
0.133687259	-0.128990836	0.007401412	0.12174647	0.011515784
0.35025507	0.139296231	0.003800987	-0.27088706	1.14E-08
6.82E-08	-0.001249942	0.979381837	-0.175117643	0.000263282
7.46E-08	-0.031107812	0.520001345	0.15053366	0.001746269
0.979099404	-0.055438206	0.25132907	0.171932512	0.000341501
0.213656523	0.266636173	1.97E-08	-0.062197074	0.198011259
0.345838081	0.24040377	4.54E-07	-0.194924448	4.71E-05
6.77E-15	0.007358588	0.879069571	0.301262972	1.80E-10

0.720079396	0.098256654	0.041700916	-0.125841005	0.008993768
0.003052198	-0.114644293	0.017395462	0.240156182	4.67E-07
0.065300573	0.058105735	0.229202795	0.250874513	1.35E-07
0.686663862	0.020949437	0.66486963	0.417277901	1.52E-19
0.368354209	0.022331224	0.644239257	-0.26241756	3.34E-08
0.007240717	-0.186234784	0.000102481	0.205898739	1.68E-05
3.31E-10	0.012593886	0.794553297	0.187933853	8.83E-05
0.000104956	0.211076737	1.01E-05	-0.090650472	0.060358605
0.155710883	0.083286227	0.084521916	-0.154909158	0.001271274
0.002721709	0.285205757	1.72E-09	-0.28445854	1.90E-09
0.938644986	0.146334373	0.002350021	-0.226565492	2.07E-06
6.24E-10	-0.02468552	0.609716905	0.34972919	8.13E-14
0.000613062	0.194546783	4.88E-05	-0.25748751	6.11E-08
0.148695058	-0.027856026	0.564571099	-0.225396242	2.34E-06
1.80E-14	0.005280027	0.913066838	0.103795078	0.031405971
0.004684779	0.138476463	0.004014459	-0.09127872	0.058592864
0.141546157	0.202988061	2.22E-05	-0.306170314	8.77E-11
0.000218449	0.410563695	6.48E-19	-0.048935879	0.31134263
0.184872259	0.050432009	0.296762917	-0.371881363	1.50E-15
0.002259868	0.173944548	0.000289906	0.217222074	5.48E-06
0.647278374	0.159420344	0.000908502	-0.11842382	0.014002758
0.596184505	0.311441412	3.99E-11	-0.243699705	3.12E-07
9.87E-06	0.027126577	0.574814901	-0.33304703	1.35E-12
3.59E-13	0.024245924	0.616100632	0.262212626	3.42E-08
1.94E-05	0.091466773	0.058072716	-0.092469731	0.05536275
0.052467798	-0.008816684	0.855348856	0.372290129	1.39E-15
0.005963499	0.151187086	0.001666285	0.17710055	0.000223408
0.827168831	0.035469319	0.463194181	-0.050334833	0.297695844
0.070902566	0.402579632	3.49E-18	0.17456112	0.000275614
1.18E-05	-0.262251857	3.40E-08	-0.176834089	0.000228416
0.010427316	0.164388076	0.000621142	-0.083927943	0.082145813
0.021087907	0.10600411	0.027952986	-0.31607025	1.98E-11
0.317972939	0.083680916	0.083054017	-0.039367286	0.415486368
0.220870261	0.073805318	0.126490353	0.100722498	0.036810594
3.83E-13	0.11489394	0.017151022	0.360577017	1.20E-14
0.001159524	0.060002681	0.214332764	-0.218741676	4.69E-06
0.320447831	-0.040566486	0.401412429	0.20379773	2.06E-05
0.849074878	0.11946497	0.013176943	0.292070771	6.66E-10
0.000910999	-0.016964683	0.725747111	0.11959943	0.013073484
2.43E-07	-0.076761347	0.111954397	-0.328308025	2.90E-12
0.048509231	0.120585312	0.012336532	-0.235580634	7.79E-07
0.001893871	0.014967034	0.75695876	0.184097701	0.000123394
0.34072763	0.037472711	0.4383036	-0.269660893	1.34E-08
7.67E-05	0.143018706	0.002955196	-0.242765393	3.47E-07
1.99E-07	-0.126950065	0.008401323	-0.007253984	0.880775711
9.55E-15	-0.043217481	0.371329461	0.178940348	0.000191531
0.576244404	0.080048102	0.097369541	-0.256290969	7.07E-08
0.151522645	0.063864554	0.186228655	0.155159203	0.001248107
0.004562684	0.03343235	0.489287022	-0.071171133	0.140640785
5.80E-07	0.106672361	0.026974904	-0.116568032	0.01558808
0.629322265	-0.076286901	0.114194678	-0.250687162	1.38E-07

3.83E-06	0.335963638	8.34E-13	0.111569402	0.020665156
0.259448693	0.124468653	0.009778139	-0.14354803	0.00284994
3.66E-13	0.167146555	0.000500571	0.041487701	0.39079718
0.003685896	0.302022521	1.61E-10	0.079004957	0.101824731
1.46E-05	0.115694298	0.016387486	0.463023677	3.10E-24
0.003275884	0.046896938	0.331957413	-0.172304464	0.000331361
9.85E-05	0.434268277	3.32E-21	0.182923394	0.000136531
3.16E-10	0.281757537	2.74E-09	0.167268339	0.000495786
1.91E-07	-0.201365873	2.59E-05	-0.040270394	0.40486062
1.66E-05	-0.045436953	0.347246494	-0.127981756	0.0078817
0.566415629	0.115113367	0.016938656	-0.205999533	1.67E-05
0.081605929	0.076144164	0.114875507	-0.196450912	4.10E-05
0.226237639	0.044992796	0.351984935	-0.045215901	0.349599678
0.000312313	0.016023328	0.740402119	-0.17344946	0.000301879
0.000506373	0.174744295	0.000271497	-0.072170174	0.135137666
3.61E-06	-0.01879211	0.697585767	-0.177730896	0.000211966
0.661380215	0.0020411	0.966337674	0.016586101	0.731629188
1.26E-05	0.404914244	2.14E-18	0.029970451	0.535382431
0.010436583	0.008347285	0.862972036	0.28116925	2.96E-09
0.058629883	0.039250801	0.41686875	-0.163171624	0.000682435
0.00092795	0.107891266	0.025266554	-0.25350038	9.89E-08
0.006046562	0.100232852	0.03774094	-0.04135506	0.392315062
4.65E-13	0.223852593	2.76E-06	0.064780152	0.179982704
0.672739665	0.055937968	0.247074522	-0.072708543	0.132241667
0.859382271	-0.056425779	0.242970435	0.117490023	0.0147818
0.561053617	0.141785942	0.003214167	-0.217817875	5.15E-06
1.63E-06	-0.285416624	1.67E-09	-0.041635753	0.389107155
0.350576199	-0.182069548	0.000146895	0.417548269	1.43E-19
0.046570303	-0.024938586	0.606055273	-0.022542271	0.641111685
0.765645448	0.09130281	0.058526018	-0.220044832	4.10E-06
0.990790946	-0.264373285	2.61E-08	-0.018277248	0.705480051
0.019521509	0.158269762	0.000990617	-0.228970047	1.60E-06
0.9579173	0.081892196	0.089874645	-0.185207172	0.000112082
2.20E-12	0.341684363	3.21E-13	-0.146141981	0.00238178
1.91E-08	-0.254385458	8.90E-08	0.189334018	7.80E-05
4.13E-08	-0.006281247	0.896667029	-0.27144761	1.06E-08
0.831465858	-0.133838621	0.005439665	0.014811252	0.759410164
0.754616528	0.238675914	5.52E-07	0.352368961	5.14E-14
1.17E-06	-0.019961364	0.679779603	0.149071248	0.001938181
4.84E-06	0.163469946	0.000666906	0.196025711	4.26E-05
6.25E-11	0.127324992	0.008209056	0.359536276	1.44E-14
0.682487664	-0.100430559	0.037362917	-0.234339259	8.93E-07
0.643953252	0.158383553	0.000982201	-0.034777522	0.471968142
4.03E-07	-0.172613281	0.000323155	-0.262915921	3.14E-08
0.065553375	0.164957516	0.00059424	-0.005070631	0.916501629
3.46E-05	0.193411285	5.41E-05	-0.131398048	0.006359676
0.670074981	-0.086795568	0.072179143	-0.03388639	0.483403488
0.069650363	0.160033494	0.000867356	0.152363722	0.001530671
5.61E-05	-0.012070231	0.802916692	0.172632444	0.000322653
0.665481638	0.049040108	0.310311918	-0.19289378	5.67E-05
9.31E-06	0.074789746	0.121495182	-0.003585606	0.940901755

0.021927114	0.306495291	8.35E-11	0.149572639	0.001870311
1.61E-05	-0.029584061	0.540659277	0.187439909	9.22E-05
2.38E-29	-0.078293367	0.104955578	0.209856357	1.14E-05
0.027731599	0.094228647	0.050863981	0.039646008	0.412189603
0.001346038	-0.058312541	0.227547009	0.4684261	7.74E-25
0.801168976	-0.111423203	0.020833106	-0.230570736	1.35E-06
0.05313909	0.008079447	0.867327542	-0.446057499	2.06E-22
0.168742248	0.086020206	0.074771303	0.483386447	1.46E-26
0.858320595	0.040905087	0.397490808	-0.354124639	3.77E-14
1.02E-06	0.059831057	0.215648877	0.13849857	0.004008563
0.008043941	0.023057344	0.633505332	0.292819323	6.00E-10
6.27E-28	0.221113599	3.67E-06	0.016708871	0.729719956
0.099087879	0.126837059	0.008460055	0.410083488	7.17E-19
0.008682215	-0.237873713	6.03E-07	-0.098563724	0.041063514
0.7618453	0.127380569	0.008180892	-0.250512309	1.41E-07
0.201561666	0.060914025	0.207440685	-0.339221739	4.86E-13
0.011854607	-0.038948398	0.420470112	0.176706	0.000230861
0.278028283	0.055827543	0.248010233	-0.036381621	0.451763382
0.003344055	0.079133355	0.101267779	-0.078595773	0.103615873
0.000471315	-0.092633544	0.054930269	-0.088588996	0.066463769
1.99E-05	0.051897734	0.282927824	-0.373062545	1.20E-15
3.45E-08	0.317112245	1.68E-11	0.117060906	0.01515239
5.24E-07	-0.266965949	1.89E-08	-0.033544008	0.48783659
0.59069859	0.014960693	0.7570585	-0.196702475	4.00E-05
8.04E-05	-0.046809299	0.332862753	0.188548496	8.36E-05
0.109598079	-0.042998073	0.373765035	0.250932148	1.34E-07
0.195979271	0.006210908	0.897817811	-0.33817226	5.79E-13
0.001036301	-0.017928791	0.710840938	0.213482166	7.99E-06
0.092102947	0.299874591	2.20E-10	-0.000720295	0.98811764
0.273595308	0.106331286	0.027470375	-0.268976981	1.46E-08
0.25071784	-0.011885782	0.805867966	-0.292701014	6.10E-10
4.29E-08	-0.030072023	0.533999586	-0.113797463	0.018247451
0.002312651	0.096176211	0.04624288	-0.303228709	1.35E-10
0.032090712	0.146300318	0.002355614	0.174091005	0.00028645
0.457392194	0.11000546	0.022524224	-0.362173302	8.97E-15
0.001266516	0.004735772	0.921997608	0.136504796	0.004572983
0.369531897	-0.03659681	0.449090504	-0.071198168	0.140489637
0.181769697	-0.009826923	0.838988754	-0.308777791	5.95E-11
4.66E-06	0.135517328	0.004878274	-0.228302847	1.72E-06
1.03E-07	-0.277843463	4.61E-09	-0.104945157	0.029565283
0.533844147	0.045200759	0.349761246	-0.305710042	9.38E-11
0.011938077	-0.017774231	0.713223374	-0.563113273	2.43E-37
0.000544339	0.04611074	0.340135876	0.439664545	9.43E-22
3.95E-06	0.217736402	5.20E-06	-0.002251532	0.962869565
0.060656439	0.019961332	0.679780092	0.231477032	1.22E-06
0.000310439	0.198337091	3.44E-05	-0.149856976	0.001832795
1.05E-08	0.334144505	1.12E-12	0.079931717	0.097858782
0.069440071	0.320889511	9.37E-12	-0.025815823	0.593439081
3.65E-05	0.029661968	0.539593235	0.071329764	0.139755691
0.000153515	-0.023301815	0.629908516	0.072022151	0.135942409
5.35E-05	-0.194738137	4.79E-05	-0.056136388	0.245399356

8.22E-11	0.138018444	0.004138398	0.257611133	6.02E-08
6.22E-06	-0.104690075	0.029965358	0.140138763	0.003592348
1.26E-05	0.128421014	0.00766931	0.144141528	0.002735987
0.592919434	0.103676753	0.031600792	-0.329246791	2.49E-12
0.336349105	0.012544987	0.795333287	0.069385219	0.150903946
0.016284499	-0.047589993	0.32485379	-0.032354301	0.503409163
0.002204226	0.201645143	2.52E-05	-0.193061871	5.58E-05
1.16E-05	0.124794494	0.009586596	-0.157915094	0.001017278
0.821848567	-0.031527291	0.514386401	0.288029243	1.17E-09
0.875495455	0.017757209	0.713485928	-0.31875295	1.31E-11
2.14E-08	-0.205085393	1.82E-05	0.035003724	0.469089257
0.000594843	0.106622655	0.027046629	-0.058959265	0.222423895
0.114275352	0.170077462	0.000396539	-0.309149429	5.63E-11
2.45E-16	0.129584152	0.007131388	-0.316646026	1.81E-11
0.138260981	0.07467241	0.122082374	0.020183014	0.676423789
0.060608632	0.168750695	0.000440851	0.086467925	0.073265417
0.019736394	-0.236007055	7.43E-07	-0.107759192	0.025447038
2.19E-07	0.112964755	0.019120508	-0.257731523	5.94E-08
0.750958482	0.325533088	4.51E-12	-0.021544275	0.655956383
0.675174265	0.05350716	0.268245692	-0.336864158	7.19E-13
1.01E-09	0.083886731	0.08229677	0.171960093	0.00034074
7.63E-11	0.162283965	0.000730646	-0.270435616	1.21E-08
0.067252874	-0.004751918	0.921732523	-0.279896551	3.51E-09
3.92E-07	-0.275500458	6.28E-09	-0.036163785	0.454478245
0.588698843	0.195977847	4.28E-05	-0.15920164	0.00092361
0.172877533	0.091493593	0.057998844	0.402877696	3.28E-18
0.27441155	0.094490894	0.050220122	-0.226943346	1.99E-06
2.40E-07	0.060470163	0.21077713	0.158105045	0.001002918
0.656713315	0.052033232	0.281671214	-0.38342308	1.65E-16
0.001163498	-0.313805075	2.79E-11	-0.049819479	0.302676049
0.001819361	0.355352214	3.04E-14	-0.264098398	2.71E-08
4.08E-09	0.055104246	0.254200366	0.137008956	0.004423861
0.080692959	0.129704368	0.007077763	-0.148013772	0.00208877
4.72E-07	0.266556246	1.99E-08	0.204997086	1.83E-05
0.004877273	0.056063583	0.246013087	0.519214569	4.70E-31
0.549032667	0.14630857	0.002354258	-0.284188098	1.97E-09
0.472739409	0.004951823	0.918451162	-0.082319301	0.088206524
0.00118891	0.244398357	2.88E-07	0.279206403	3.85E-09
4.01E-09	0.158639087	0.000963541	-0.336278561	7.92E-13
1.30E-05	0.119478637	0.013166394	-0.256858877	6.60E-08
0.000116353	-0.144492798	0.002670513	0.237514668	6.28E-07
0.625244792	-0.366942747	3.75E-15	-0.086215001	0.074113049
5.61E-08	0.168522902	0.000448908	0.399188434	7.03E-18
0.028261077	0.06918676	0.152078671	-0.302191999	1.57E-10
0.0822466	-0.015022357	0.756088771	0.144558324	0.002658458
0.005109941	0.339238449	4.84E-13	0.087304986	0.070516342
0.250950295	-0.068147448	0.158344167	-0.026595019	0.582334446
4.57E-05	0.173145103	0.000309468	-0.251144821	1.31E-07
0.000113038	-0.029052966	0.547954357	0.154812897	0.001280297
7.78E-11	0.031488773	0.514900677	0.019798067	0.682256002
0.002327012	-0.176815041	0.000228778	-0.047962974	0.321071949

2.93E-11	-0.095378555	0.048091077	-0.055675083	0.249306196
2.90E-17	-0.004142024	0.931751648	0.321833937	8.09E-12
0.006217575	0.072985821	0.130768943	-0.135636066	0.004840617
0.017942613	0.014459149	0.764959769	0.114769516	0.017272473
0.241594369	0.287970086	1.18E-09	-0.074977415	0.120560602
0.300247789	0.211775313	9.47E-06	-0.160468099	0.000839241
5.58E-08	0.271401056	1.07E-08	-0.217469299	5.34E-06
7.37E-15	0.034495034	0.475576972	-0.214326496	7.34E-06
0.135756209	0.173525235	0.000300017	-0.016023423	0.740400627
0.003584359	0.233421985	9.88E-07	-0.057317643	0.235590919
0.483154736	-0.058089302	0.229334733	0.336475826	7.66E-13
0.001067027	0.1823346	0.000143601	-0.243283727	3.27E-07
0.000267484	0.025729384	0.594676894	0.076026928	0.115437073
0.200162471	0.052365691	0.278603978	-0.109163854	0.023583346
0.112542311	0.071211596	0.140414612	-0.189887087	7.43E-05
0.845822148	0.025221805	0.601969032	-0.340465796	3.94E-13
0.034817713	0.001243294	0.97949148	0.000366224	0.993958422
8.65E-13	0.183191574	0.00013342	-0.017311719	0.720369273
0.195633816	-0.287629513	1.23E-09	-0.01911977	0.692578667
2.44E-06	-0.404618368	2.28E-18	-0.064985764	0.178601657
0.871509504	0.014626107	0.762326779	-0.209669191	1.17E-05
2.98E-18	0.216721918	5.76E-06	0.110767664	0.021600831
0.001658086	0.480568774	3.13E-26	-0.078861169	0.10245132
9.42E-05	0.143954182	0.002771502	0.051388439	0.287684873
0.245769066	0.170051821	0.000397354	-0.106651471	0.027005028
7.76E-09	0.07070232	0.143281665	0.123173773	0.010573332
1.87E-15	-0.202359938	2.36E-05	0.283159092	2.27E-09
0.067326747	0.007977011	0.868994402	-0.273572213	8.08E-09
1.63E-14	0.338562536	5.42E-13	-0.028742581	0.552240109
1.89E-06	0.070201206	0.146146245	0.164742478	0.00060427
1.39E-05	0.088038267	0.06817793	-0.085834277	0.075404025
1.89E-12	0.077095335	0.110398166	-0.084863211	0.078779727
0.000123024	0.066692901	0.16743732	0.215066415	6.81E-06
0.010201923	0.030586571	0.527022053	-0.113908738	0.018133461
0.657081285	0.003679903	0.939350491	0.282838314	2.37E-09
0.343597779	0.319791166	1.11E-11	-0.100369254	0.03747979
0.40374063	0.048494593	0.315731342	-0.065740707	0.173598246
0.262741732	0.124425858	0.009803546	-0.134944646	0.00506363
1.83E-05	0.219909999	4.16E-06	0.28985503	9.07E-10
1.76E-24	-0.344205988	2.10E-13	-0.261846062	3.58E-08
5.05E-09	0.220613907	3.87E-06	0.023611006	0.625371977
0.092871661	-0.003850852	0.93653884	0.303724129	1.26E-10
2.90E-19	0.028700441	0.552823232	0.263610655	2.88E-08
0.000110607	-0.079542701	0.099508286	-0.352271884	5.22E-14
0.000109218	0.078019218	0.106181906	-0.025373777	0.599781512
0.032070288	-0.10263479	0.033361399	0.283202922	2.25E-09
1.72E-10	-0.159887371	0.000877002	-0.018493655	0.702158015
8.43E-08	0.028361641	0.557522393	0.215162103	6.75E-06
0.179857471	0.004986674	0.917879225	-0.091728182	0.057356025
2.16E-05	0.320433593	1.01E-11	-0.015787926	0.744081811
0.689426143	0.136252648	0.004649254	-0.226975148	1.98E-06

0.001089148	0.113619368	0.018431195	-0.237045651	6.62E-07
0.001835322	0.016036195	0.740201149	0.205656538	1.72E-05
0.042096685	-0.013295593	0.783383153	-0.230247722	1.40E-06
0.010852632	0.251148456	1.31E-07	-0.042136062	0.383428957
8.42E-17	-0.017094269	0.723737391	-0.452010929	4.85E-23
0.100817899	0.195530435	4.46E-05	0.010717966	0.824616026
1.50E-06	0.192834517	5.70E-05	0.32989383	2.25E-12
0.459536658	0.083820008	0.082541646	-0.189800789	7.48E-05
1.50E-06	0.054062695	0.263301069	0.388826599	5.71E-17
0.87563015	0.207657824	1.42E-05	-0.255760689	7.54E-08
0.824968162	2.19E-05	0.999637985	-0.51680197	9.81E-31
0.907392651	0.099046986	0.040077048	-0.14085961	0.003422142
2.72E-06	0.228841616	1.62E-06	-0.197791099	3.62E-05
0.209080593	0.086212406	0.074121788	-0.142360249	0.00309106
0.154007786	0.245414063	2.56E-07	0.085154217	0.077755489
0.121994178	0.123910765	0.010113988	-0.092723057	0.054695134
2.59E-05	-0.005181727	0.914679091	-0.219832495	4.19E-06
0.000544945	0.254640236	8.63E-08	-0.157247966	0.001069227
0.001601594	0.192223653	6.02E-05	-0.24247893	3.59E-07
0.163930002	-0.065970747	0.172094588	0.016208334	0.73751432
0.013904092	-0.125058754	0.00943371	-0.248254734	1.84E-07
0.978208805	0.088359179	0.06717473	-0.184303474	0.000121218
0.860972241	0.066242063	0.170333632	-0.044025415	0.362445745
9.11E-11	-0.036558784	0.449562171	0.037580012	0.436992443
2.23E-11	0.188210835	8.62E-05	0.230491352	1.36E-06
0.000152139	-0.058388955	0.226937357	-0.024924946	0.606252393
5.43E-13	-0.078605496	0.103573027	-0.114540568	0.017497916
0.001399032	-0.068480144	0.156317663	0.109205031	0.023530541
0.006054527	0.311256445	4.11E-11	-0.001694617	0.972049394
0.169710518	0.0481688	0.318997299	-0.350304271	7.36E-14
0.639431166	0.024642419	0.610341513	0.298819611	2.56E-10
2.22E-06	0.008774784	0.856028786	0.114230358	0.017807477
0.000173519	0.007627664	0.874683362	-0.022829841	0.636860293
1.58E-05	0.170363503	0.000387545	-0.035065008	0.468310955
1.90E-08	0.133997671	0.005384087	-0.101750711	0.034920165
6.98E-07	-0.012973904	0.788498603	0.298421648	2.71E-10
0.941779737	0.332688052	1.43E-12	-0.282154964	2.60E-09
0.441103053	0.031496795	0.514793543	-0.067363555	0.163197281
8.76E-24	-0.016774812	0.728695169	0.178630267	0.000196587
0.006463546	0.006053077	0.900400804	-0.146453582	0.002330537
0.885425958	0.086008323	0.07481161	-0.260451987	4.25E-08
2.38E-20	-0.110723967	0.021652867	-0.012177591	0.801200156
0.193073189	0.124255559	0.009905235	0.176401401	0.000236774
0.010995949	0.111761897	0.020445816	0.142405734	0.003081495
1.72E-05	-0.084716767	0.07929928	0.256138659	7.20E-08
0.040040439	-0.039509733	0.413799544	-0.346074764	1.53E-13
7.38E-17	0.033752796	0.485130642	0.164743615	0.000604216
6.45E-08	0.312218912	3.55E-11	0.13614028	0.004683611
0.007594594	0.014287812	0.767664626	-0.18814907	8.66E-05
1.22E-12	0.067918163	0.159752274	0.010694998	0.824985792
0.003914294	0.129166845	0.007320371	-0.196462903	4.09E-05

0.003586896	-0.049138318	0.309342789	-0.305386508	9.84E-11
0.375840496	0.291254175	7.46E-10	0.027050275	0.575891471
7.07E-05	-0.064381184	0.182685014	0.500659044	1.16E-28
4.54E-07	0.298283197	2.76E-10	-0.107827691	0.025353294
0.495355855	-0.022607818	0.640141605	-0.154778241	0.001283561
1.55E-21	0.034907569	0.47031184	0.389189647	5.31E-17
8.53E-12	-0.026648404	0.581577178	0.093595223	0.052447603
0.916686673	-0.001045959	0.982746031	-0.359023555	1.58E-14
3.59E-05	0.000391538	0.993540813	-0.286950036	1.35E-09
0.853280658	0.18738324	9.27E-05	0.014637683	0.762144319
0.058700651	0.301588023	1.71E-10	-0.069665423	0.149257103
0.103767269	0.139570194	0.00373197	-0.268185686	1.62E-08
8.51E-11	0.050760416	0.293624531	0.246707381	2.20E-07
0.069132425	0.265836657	2.17E-08	0.209445386	1.19E-05
1.25E-10	0.140273985	0.003559846	0.30881138	5.92E-11
3.46E-06	0.056938672	0.238707081	-0.273050029	8.65E-09
0.460886937	-0.061158366	0.205620386	0.282625312	2.44E-09
0.189862159	0.206345968	1.61E-05	0.040910111	0.397432795
0.269343631	0.02944881	0.542512465	-0.334204839	1.11E-12
1.18E-07	0.37956956	3.49E-16	-0.16173567	0.000761981
2.43E-14	0.432512588	4.98E-21	-0.1077133	0.025510012
0.690104471	0.011649673	0.809649867	0.371133996	1.73E-15
0.203372719	0.167950535	0.000469759	-0.082982144	0.085667046
2.25E-06	0.030632637	0.52639965	0.385769593	1.04E-16
0.039045427	-0.023982797	0.619935635	0.339181911	4.89E-13
0.005488	-0.008028248	0.868160592	-0.173825872	0.000292735
3.33E-05	0.084107194	0.081491828	-0.330260705	2.12E-12
0.289378765	0.27197804	9.93E-09	-0.067339053	0.163350753
0.010118199	-0.099709833	0.038756637	0.203308343	2.16E-05
0.002375375	0.072931393	0.131057028	0.304221547	1.17E-10
0.292616223	0.126934347	0.00840947	0.487258724	5.06E-27
0.072664024	0.204086552	2.00E-05	0.321521158	8.49E-12
0.992321767	-0.106100157	0.027810559	0.012461028	0.796673006
5.26E-05	0.056014404	0.246428267	-0.254893033	8.37E-08
0.103133575	0.093797895	0.051936494	-0.124180752	0.009950198
0.189847041	0.032784606	0.497746693	-0.249200176	1.65E-07
0.63292421	-0.009821885	0.83907018	-0.402288288	3.70E-18
0.03598275	0.170734279	0.00037617	0.326224699	4.04E-12
0.001324642	-0.164054994	0.000637397	0.088607244	0.066407584
0.091726576	0.415204155	2.38E-19	-0.067445046	0.16268762
0.000128195	0.056423931	0.242985891	-0.15568377	0.001200762
5.08E-13	0.130394109	0.006777001	0.232316388	1.11E-06
4.76E-07	0.131203373	0.006438751	-0.226986101	1.98E-06
0.457271228	0.038401667	0.427027228	-0.217235861	5.47E-06
6.42E-15	-0.021223379	0.660758838	0.180703636	0.000165022
0.787612199	0.228762974	1.64E-06	-0.138972954	0.003883911
0.034181153	0.087229815	0.070759724	-0.183246983	0.000132785
0.053744446	0.015601538	0.746999494	0.184008137	0.000124353
0.609959526	0.111382983	0.020879517	0.089522913	0.063637872
0.017042358	0.209668261	1.17E-05	0.349472977	8.50E-14
0.064658135	0.097521896	0.043260047	-0.167027183	0.000505303

0.022228704	0.115966271	0.016134887	0.098253599	0.041707299
0.005303793	0.311559784	3.92E-11	-0.211107373	1.01E-05
0.001754195	0.216367974	5.97E-06	0.020452678	0.672349644
0.239237234	0.227257314	1.92E-06	-0.065439098	0.175584504
4.51E-09	0.042535716	0.378929653	-0.11191425	0.020273654
0.005296688	0.038678166	0.423703698	-0.057540045	0.23377559
0.073888667	0.005075724	0.916418059	-0.390340968	4.22E-17
0.00056827	0.012903193	0.789624266	-0.002329149	0.961590533
0.01195045	0.025393135	0.599503123	0.0824714	0.087618521
0.835223039	0.008614294	0.858634122	-0.409819501	7.59E-19
0.922329846	0.055063878	0.254548973	0.009106244	0.850652985
0.88191079	0.259028395	5.07E-08	-0.006119528	0.899313159
0.000424922	0.054074828	0.263193781	-0.236653279	6.91E-07
1.41E-09	-0.023406253	0.628374597	-0.262199243	3.43E-08
0.205472385	0.022703956	0.638719906	-0.142723898	0.00301534
0.363423044	0.161457128	0.000778371	-0.109481091	0.023179165
0.003455275	0.242323912	3.65E-07	-0.070358882	0.145240231
6.21E-08	0.081245534	0.092448337	0.197430535	3.74E-05
0.096400924	-0.172842686	0.000317183	0.061002127	0.206783002
0.034585572	-0.104091389	0.0309226	0.018167997	0.707159288
3.27E-08	0.052747068	0.275113411	0.375355458	7.80E-16
3.43E-05	-0.131671342	0.006250135	-0.108391827	0.024592504
3.18E-07	0.386206089	9.58E-17	-0.041929593	0.385766099
0.63355767	0.027276479	0.572702662	-0.308455419	6.25E-11
0.015230209	-0.173488363	0.000300922	0.029260693	0.545095275
2.04E-05	0.102979393	0.032770087	-0.073547497	0.127824562
0.012439249	0.007349468	0.879218292	0.14426348	0.002713092
0.001444826	0.018413015	0.703395248	-0.289763084	9.18E-10
4.42E-06	-0.084859624	0.078792422	-0.309091301	5.68E-11
0.210147157	-0.028233101	0.559310291	-0.344001471	2.17E-13
0.035240788	-0.089235197	0.064497719	0.18780348	8.93E-05
0.004045143	0.164809022	0.000601149	0.218524316	4.79E-06
0.009001496	0.188936664	8.08E-05	0.080763512	0.094404869
0.791719719	0.052576344	0.276672278	0.410297722	6.85E-19
3.11E-12	0.096082297	0.04645734	0.442721082	4.57E-22
0.001211289	0.092368229	0.05563214	0.453138433	3.68E-23
0.736610802	0.023587055	0.625722883	-0.434985373	2.81E-21
0.025974851	0.150624538	0.001734938	0.215315705	6.64E-06
8.87E-25	0.156142023	0.001160762	-0.1139418	0.018099711
0.032771727	-0.107472961	0.025842003	0.08585566	0.075331037

MEgrey	MEgrey
0.110645915	0.021746086
-0.130324764	0.006806713
0.131539676	0.006302696
0.176564048	0.000233599
-0.034590467	0.474356113
-0.126390934	0.008695509
-0.103915199	0.031209245
-0.027827824	0.56496551
-0.1366476	0.00453029
-0.235904868	7.51E-07
-0.164321226	0.000624373
0.366358295	4.18E-15
-0.255283672	7.99E-08
-0.084437819	0.080296609
0.129017227	0.00738921
0.045076634	0.351087402
0.245190326	2.63E-07
-0.100288369	0.037634464
-0.106072538	0.027851452
-0.120978129	0.012053272
-0.494113997	7.49E-28
-0.037328836	0.440065196
-0.02720924	0.573649658
-0.092819205	0.054443504
-0.003373082	0.944398759
-0.18320395	0.000133278
0.156643271	0.001118416
0.115546821	0.0165259
-0.018339668	0.704521274
0.117360232	0.01489304
0.185522934	0.000109045
-0.080501012	0.095484189
-0.077990091	0.106312861
0.014480658	0.7646204
-0.110023702	0.022501732
-0.137492816	0.004284887
0.178490574	0.000198905
0.068536687	0.15597521
-0.182554765	0.000140918
-0.229604457	1.50E-06
0.13114239	0.006463703
-0.007793365	0.871984165
-0.062661388	0.194677088
-0.181736075	0.000151139
0.121179735	0.011910137
-0.157525084	0.001047358
-0.169620053	0.000411325
0.098208657	0.041801297
-0.147045886	0.002235896
-0.119432301	0.013202187

0.111966973	0.020214371
-0.087962066	0.068417936
-0.156032	0.001170252
-0.211606524	9.63E-06
-0.053339034	0.269754577
-0.007905647	0.870155985
0.026944596	0.577384096
0.146721177	0.002287336
-0.149022852	0.001944849
-0.22648659	2.09E-06
0.080897714	0.093856848
-0.12445268	0.009787615
-0.105141911	0.029259826
-0.150409042	0.001761917
-0.130103391	0.006902345
-0.141395302	0.00330043
-0.026482411	0.583933268
-0.201364319	2.59E-05
-0.10799853	0.025120788
-0.110731409	0.021643998
-0.038255031	0.428795924
-0.288252083	1.13E-09
-0.083420436	0.084020448
-0.226534481	2.08E-06
-0.093482435	0.052733849
-0.115019187	0.017029522
-0.038009133	0.431771408
-0.26662544	1.97E-08
0.014046629	0.771476924
-0.157094749	0.001081498
-0.266784382	1.93E-08
-0.19578694	4.36E-05
0.070939535	0.141940703
-0.099283404	0.039601798
-0.11106654	0.021247814
0.310979639	4.28E-11
0.032027945	0.507726237
0.032024184	0.507776098
0.046905303	0.331871081
0.154044751	0.001354443
-0.057284275	0.235864135
-0.096354388	0.045838284
-0.066932054	0.165915977
-0.106691112	0.026947889
-0.055958233	0.246903067
-0.210806636	1.04E-05
0.223882326	2.75E-06
-0.033419118	0.489459065
-0.131695996	0.006240337
0.09972614	0.038724624
0.105222121	0.029136081

-0.221560779	3.50E-06
0.061332468	0.204330434
-0.060686869	0.209143378
-0.135007279	0.005043053
0.1437612	0.002808523
-0.054064609	0.263284145
0.093241237	0.053350356
0.44672966	1.75E-22
0.039452151	0.414480929
0.047911133	0.321595871
0.026497541	0.583718331
-0.12671733	0.008522681
0.208883583	1.26E-05
0.150191925	0.001789487
-0.230881394	1.30E-06
-0.084266541	0.080913998
-0.040841804	0.398221981
-0.212719551	8.62E-06
0.183025658	0.000135336
-0.063254309	0.190479318
-0.154467279	0.001313184
0.180646437	0.000165825
-0.066863786	0.166349195
-0.071199974	0.140479545
-0.205668846	1.72E-05
-0.247515388	2.01E-07
-0.049967257	0.301242354
0.187615508	9.08E-05
-0.256326615	7.04E-08
-0.142670084	0.003026438
-0.102028042	0.03442465
-0.055847847	0.247837998
0.078220772	0.105279215
-0.248263393	1.84E-07
-0.317840913	1.50E-11
-0.149979915	0.001816789
0.11415033	0.017888109
0.203323573	2.15E-05
-0.280099749	3.42E-09
-0.231864128	1.17E-06
0.243762928	3.10E-07
0.188314478	8.54E-05
0.025691877	0.595214352
-0.0113768	0.814026151
-0.232514494	1.09E-06
-0.022032658	0.648674543
0.343600986	2.33E-13
-0.064174756	0.184094913
0.001568934	0.974121616
0.063051264	0.191909297
-0.282110816	2.61E-09

-0.256876249	6.59E-08
0.041564326	0.389921944
-0.032863436	0.496713029
-0.15184929	0.001588665
-0.187883965	8.87E-05
0.349037007	9.17E-14
-0.018401321	0.703574739
-0.086450247	0.073324403
-0.019222228	0.691015706
0.038015216	0.431697658
0.494996596	5.84E-28
0.090120055	0.061883398
0.051291348	0.288597817
0.303553145	1.29E-10
-0.133771717	0.005463197
-0.113776149	0.018269358
0.316072665	1.97E-11
0.072548528	0.133097376
-0.081302107	0.092220848
-0.053275945	0.270322276
-0.211871446	9.38E-06
0.244283744	2.92E-07
-0.147115024	0.002225081
0.146853793	0.002266198
-0.127330893	0.008206062
0.051611242	0.285597217
0.052369824	0.278565987
-0.12780538	0.007968448
-0.016389777	0.734685731
0.178735477	0.000194858
-0.19989954	2.98E-05
-0.089308512	0.064277712
-0.286378559	1.46E-09
0.109416842	0.023260536
-0.079671451	0.098959931
-0.131800052	0.006199135
-0.134448252	0.005229402
0.040040713	0.407547521
-0.101334792	0.035674666
-0.054669916	0.257968517
0.096413032	0.045705769
0.257150732	6.37E-08
0.093064575	0.053805702
0.094033385	0.051347845
-0.058724549	0.224273633
-0.066475437	0.168829736
-0.094426015	0.050378773
-0.094550178	0.050075516
0.021051211	0.663341222
-0.154704901	0.001290491
-0.32867692	2.73E-12

-0.0669781	0.16562425
-0.159720086	0.000888167
-0.172302738	0.000331407
0.023858715	0.621747691
-0.009645045	0.841929237
0.43621678	2.11E-21
0.127150545	0.008298021
0.28782778	1.20E-09
0.010175578	0.833358206
-0.139457722	0.003760165
-0.063717439	0.187246938
-0.029703415	0.539026524
0.111223847	0.021064031
-0.258165049	5.63E-08
-0.264087196	2.71E-08
-0.045626267	0.34523916
0.027818709	0.565093015
0.068172821	0.158188922
0.506538389	2.10E-29
0.084435909	0.080303474
-0.206300401	1.62E-05
-0.086598101	0.072832245
-0.122096893	0.011277765
-0.081394159	0.091851656
-0.027695599	0.566816479
-0.117787562	0.014529537
-0.012135541	0.801872361
-0.017459726	0.718079855
-0.328514414	2.80E-12
-0.038555448	0.425176911
-0.136813488	0.004481148
-0.164785114	0.000602269
-0.062163253	0.198255735
-0.054387423	0.260439988
-0.151832967	0.001590537
-0.189269216	7.84E-05
0.035022372	0.46885236
-0.157107636	0.001080461
-0.038864971	0.421466846
-0.233060311	1.03E-06
-0.018318729	0.704842852
0.203625739	2.09E-05
-0.106896938	0.026652881
0.025191245	0.602409345
-0.083167976	0.084965756
-0.234218462	9.05E-07
-0.276373592	5.60E-09
-0.138392912	0.004036814
-0.03812002	0.43042815
-0.018791277	0.697598504
-0.003782275	0.937666647

-0.066328036	0.169778451
-0.010569837	0.827001462
0.000480199	0.992078227
-0.106261768	0.027572315
-0.16155535	0.000772555
-0.063384055	0.189569666
-0.230258042	1.39E-06
-0.087251698	0.070688801
-0.236864128	6.75E-07
-0.034986005	0.469314415
-0.017120951	0.723323812
-0.206056677	1.66E-05
0.147764654	0.002125767
0.164245611	0.000628047
-0.020693111	0.668725209
-0.211576246	9.66E-06
-0.115783901	0.016303886
-0.120315461	0.012534511
-0.004210046	0.930633657
0.356732846	2.38E-14
-0.106261015	0.027573422
-0.036285286	0.452962858
-0.09570338	0.047331112
-0.000952125	0.98429368
-0.072548185	0.13309921
-0.124476064	0.009773745
-0.16277802	0.000703437
0.152496196	0.001516054
-0.219751845	4.23E-06
-0.206041511	1.66E-05
-0.005321512	0.912386544
-0.049807227	0.302795112
-0.023301851	0.629907985
-0.14595094	0.002413703
-0.304884593	1.06E-10
0.022142313	0.647044133
-0.150911379	0.001699615
-0.107067638	0.026410331
-0.104315453	0.030561323
0.034878972	0.470675787
-0.03494445	0.469842704
-0.16328398	0.000676547
-0.000231727	0.99617718
0.177362987	0.000218576
-0.148515503	0.002016036
-0.238104318	5.88E-07
0.004566817	0.924772061
0.041060645	0.395696896
-0.170148927	0.000394274
-0.083969094	0.081995302
0.053239785	0.270648024

-0.251590677	1.24E-07
-0.038130217	0.43030475
-0.135746794	0.004805736
0.016782697	0.728572661
-0.209853049	1.14E-05
0.019352911	0.689024081
-0.142102631	0.003145742
-0.202453668	2.34E-05
-0.129903989	0.006989513
-0.074950583	0.12069388
-0.188652001	8.29E-05
-0.019206664	0.691253045
0.192184965	6.04E-05
0.131445847	0.006340394
0.05089653	0.292330295
-0.190678479	6.92E-05
-0.092054007	0.056473005
0.013741321	0.776310701
-0.068033209	0.15904457
-0.187032891	9.56E-05
0.19797358	3.56E-05
-0.373782459	1.05E-15
-0.049204476	0.308691078
0.162259407	0.000732024
-0.03479602	0.471732357
0.031361421	0.516602895
-0.146851787	0.002266516
-0.10325208	0.032308536
0.317806498	1.51E-11
-0.057123534	0.23718341
-0.096741362	0.044969789
0.022801395	0.637280316
-0.038224507	0.429164634
-0.310821747	4.38E-11
0.106130875	0.02776514
-0.19108565	6.67E-05
-0.142266024	0.003110959
-0.146572942	0.002311176
-0.14206041	0.003154788
-0.283065801	2.30E-09
-0.200826545	2.73E-05
-0.065550226	0.17485071
-0.263831851	2.80E-08
0.215300208	6.65E-06
0.001904486	0.968589538
-0.154899683	0.001272159
-0.138072607	0.004123563
-0.092013553	0.056582022
-0.251236327	1.30E-07
-0.097457636	0.043398707
-0.083494083	0.083746288

-0.096340627	0.045869424
0.00559242	0.90794561
-0.165500692	0.00056959
-0.210397474	1.08E-05
0.151729868	0.001602413
0.057439014	0.234599014
-0.1522891	0.001538961
0.007056589	0.883996842
-0.141478547	0.003281874
-0.240960098	4.26E-07
-0.06371488	0.187264686
-0.228856796	1.62E-06
-0.094584568	0.049991794
-0.041477301	0.390916065
0.291427628	7.28E-10
-0.130644172	0.006670821
-0.109549601	0.023092672
-0.278603573	4.17E-09
0.154751142	0.001286117
0.082009279	0.089414862
-0.180106497	0.000173588
-0.167303795	0.000494401
-0.251385007	1.27E-07
0.042766706	0.376344005
-0.336370135	7.80E-13
-0.132907258	0.0057756
-0.280421414	3.28E-09
0.030937443	0.522290787
-0.035481016	0.463046614
-0.015382278	0.750436362
-0.136218008	0.004659821
0.16625744	0.000536827
-0.072189234	0.135034312
0.12210894	0.011269661
0.10232171	0.033906483
-0.09313207	0.053631353
0.113596855	0.018454537
-0.131188108	0.006444989
-0.206585744	1.57E-05
-0.008933271	0.853457509
-0.048587505	0.314803962
0.113298967	0.018765824
-0.016339072	0.735475833
-0.058710152	0.224387446
9.31E-05	0.998463624
-0.009495911	0.844341978
0.013188563	0.785084112
-0.149592503	0.001867667
-0.257080663	6.42E-08
-0.241272807	4.11E-07
-0.035806668	0.458948708

-0.052691546	0.275619717
-0.09146889	0.058066884
-0.041725132	0.38808903
-0.10110316	0.036100837
-0.279689593	3.61E-09
-0.073345865	0.128875576
-0.101730067	0.034957292
0.166442749	0.000529075
-0.054384303	0.260467372
-0.26264743	3.24E-08
0.045863037	0.34273905
-0.095309539	0.048253856
-0.161024788	0.000804459
-0.237207123	6.50E-07
0.099438035	0.039293543
-0.02049152	0.671763599
-0.108713929	0.024167022
-0.17038595	0.000386848
-0.26229306	3.39E-08
0.047631676	0.324429719
-0.103766659	0.031452668
-0.231635699	1.20E-06
0.216378776	5.97E-06
-0.112910155	0.019179004
-0.153347186	0.001425176
-0.070818082	0.142626064
0.055473938	0.251023198
-0.109919221	0.022630824
-0.057741068	0.232143282
-0.214778266	7.01E-06
-0.001287234	0.978766837
-0.254944315	8.32E-08
-0.18642885	0.000100757
0.17104733	0.000366809
0.063455699	0.189068736
0.134343562	0.005264981
-0.094514801	0.050161765
0.154747633	0.001286449
-0.080574293	0.095181892
-0.039715014	0.411375784
-0.132023615	0.006111436
0.019289957	0.689983242
-0.106545316	0.027158553
0.091215343	0.058769029
-0.253608346	9.77E-08
0.064600745	0.181194188
-0.054131921	0.262689336
-0.195539692	4.46E-05
-0.00366115	0.939658981
-0.09685203	0.044723972
0.012524697	0.795657003

-0.305290225	9.98E-11
0.078507985	0.104003385
0.012630047	0.793976613
0.010020436	0.835862609
-0.074841338	0.121237696
-0.155678316	0.001201246
-0.041812012	0.387100935
0.016277255	0.73643947
0.019619313	0.684970726
-0.363668166	6.84E-15
-0.362008881	9.24E-15
-0.271985021	9.93E-09
-0.04801577	0.320538954
-0.030970586	0.521844999
-0.178023296	0.000206846
-0.098346883	0.041512762
-0.037521787	0.437703646
0.050928665	0.292025303
-0.12524851	0.009325269
-0.072841789	0.131532361
-0.081756407	0.090410256
-0.06020326	0.212801931
-0.18802505	8.76E-05
-0.109429683	0.023244253
0.017921541	0.710952625
-0.122291614	0.011147395
-0.128671555	0.007550471
0.131838115	0.006184124
-0.2801363	3.40E-09
-0.046956031	0.331347853
0.068948361	0.153498971
0.110021157	0.022504869
0.058931536	0.222641849
-0.22698309	1.98E-06
0.124804538	0.009580746
0.250214295	1.46E-07
-0.136622239	0.004537846
-0.093662801	0.052276717
-0.138132848	0.00410712
-0.087255864	0.070675306
-0.025403326	0.5993566
0.047556973	0.325189986
-0.016061988	0.739798356
-0.027256564	0.572983066
-0.12483682	0.009561962
-0.117785215	0.014531512
-0.273720944	7.93E-09
-0.130582099	0.006697038
-0.030749898	0.524816997
0.023204698	0.631336317
0.290365362	8.45E-10

-0.175144168	0.000262707
-0.02545488	0.598615566
-0.134533195	0.005200693
-0.082200192	0.088669202
0.055839983	0.247904695
-0.248472443	1.79E-07
0.144462825	0.002676043
-0.101911921	0.034631392
0.239289742	5.15E-07
-0.076994629	0.110865607
0.273067241	8.63E-09
-0.064591258	0.181258421
-0.065206779	0.177125933
-0.093623796	0.052375295
0.05809233	0.229310421
0.13608904	0.004699354
-0.119723991	0.012978282
-0.159690037	0.000890187
-0.102404121	0.033762272
-0.205626006	1.73E-05
-0.228969587	1.60E-06
0.110840674	0.021514129
-0.077784171	0.107242313
-0.025610308	0.596383981
-0.219615794	4.29E-06
-0.221754312	3.43E-06
0.125069103	0.009427767
-0.05435197	0.260751312
-0.07583918	0.116340879
-0.141911368	0.003186907
-0.001603168	0.973557161
-0.191859586	6.22E-05
0.077768975	0.107311155
-0.171154459	0.000363656
-0.141689045	0.003235371
0.111500191	0.020744516
0.114041546	0.017998224
-0.022575603	0.640618307
0.398971815	7.35E-18
-0.126863533	0.008446264
-0.068401769	0.156793282
-0.134066158	0.005360313
0.012323543	0.798868125
-0.156515236	0.001129095
-0.004797202	0.920989083
-0.164741462	0.000604317
-0.202032273	2.43E-05
-0.068356359	0.157069353
-0.084964706	0.078421265
-0.191857361	6.22E-05
0.102049616	0.034386355

-0.205942324	1.68E-05
-0.251634591	1.24E-07
0.020353158	0.673852106
0.093993083	0.051448189
0.014472599	0.764747546
0.174526905	0.00027639
0.005491922	0.909592717
-0.262892263	3.14E-08
-0.287038002	1.34E-09
-0.121042822	0.012007177
-0.02402534	0.619314873
0.169167112	0.00042647
-0.065166179	0.177396335
-0.152726729	0.001490924
-0.13016094	0.006877369
-0.008372103	0.862568656
-0.056918308	0.238875342
-0.228967277	1.60E-06
-0.076494468	0.113210284
-0.229476796	1.52E-06
0.336256359	7.95E-13
0.22726883	1.92E-06
0.038106039	0.430597377
0.236461494	7.06E-07
-0.122512527	0.011001105
0.043403894	0.369267881
-0.158999499	0.000937779
0.089677244	0.063180551
-0.132464382	0.005941789
0.268395845	1.57E-08
-0.168680879	0.000443306
-0.013147745	0.78573307
-0.004588765	0.924411602
-0.030294591	0.530975751
-0.111330053	0.020940732
0.162505402	0.000718333
0.142270757	0.003109957
0.493115945	9.92E-28
-0.022083126	0.64792394
0.190118387	7.27E-05
0.18933311	7.80E-05
-0.375975168	6.93E-16
-0.195441291	4.50E-05
-0.069513174	0.150150214
0.071436469	0.139162732
-0.061535697	0.202832104
-0.237648419	6.19E-07
0.162919368	0.000695827
-0.127999404	0.007873066
-0.09253685	0.055185209
-0.184518883	0.000118979

-0.111952744	0.020230356
-0.102524199	0.033553084
-0.258960772	5.11E-08
-0.027097034	0.575231625
-0.143858418	0.002789817
-0.143971209	0.002768257
0.297531909	3.08E-10
-0.190211621	7.21E-05
-0.372454977	1.35E-15
-0.154392324	0.001320418
-0.131439218	0.006343065
0.322340195	7.47E-12
0.259914314	4.54E-08
0.144771265	0.002619625
-0.173154074	0.000309241
-0.088099109	0.067986795
-0.052457611	0.277759937
0.030363764	0.530037723
0.013479179	0.780467959
-0.112920372	0.019168046
0.020951974	0.664831511
0.114343516	0.017694006
-0.146283075	0.002358451
-0.201530955	2.55E-05
0.217019711	5.59E-06
-0.234141453	9.13E-07
0.179768475	0.00017862
-0.072757657	0.131979875
-0.181555962	0.000153479
-0.198486709	3.39E-05
0.181114238	0.000159363
-0.148854931	0.001968152
-0.030343816	0.530308145
0.070609364	0.143809774
-0.205945592	1.67E-05
0.15639196	0.001139466
0.194544517	4.88E-05
-0.10121525	0.035894069
-0.248275355	1.84E-07
0.042661152	0.377524208
-0.236123029	7.33E-07
-0.149394649	0.001894151
-0.164091559	0.000635593
-0.091209432	0.058785483
0.103450211	0.031976672
0.320087909	1.06E-11
-0.115571352	0.016502806
-0.106824116	0.026756936
-0.007482889	0.877042861
-0.210364455	1.09E-05
-0.136590083	0.004547442

0.003130256	0.948395684
-0.210017466	1.13E-05
-0.088432283	0.066947902
-0.117510259	0.014764522
0.524092526	1.04E-31
0.086558583	0.072963522
-0.253048529	1.04E-07
-0.181180213	0.000158471
-0.112232082	0.019918551
-0.30947447	5.36E-11
-0.265822152	2.18E-08
-0.121908932	0.011404887
-0.108345053	0.024654825
0.070590387	0.143917768
0.045462005	0.346980436
-0.039754469	0.410910908
-0.176920806	0.000226775
-0.146282499	0.002358546
-0.019766639	0.682733014
-0.144739568	0.002625373
-0.188726981	8.23E-05
-0.046909345	0.331829373
-0.00651024	0.892922075
0.025196714	0.602330535
-0.250153687	1.47E-07
0.06851334	0.156116545
-0.020547139	0.670924776
0.024350469	0.6145798
-0.104815338	0.029768318
-0.029614771	0.540238926
-0.173786154	0.000293688
-0.198296716	3.46E-05
0.121317265	0.011813358
-0.010351278	0.830523987
-0.166128959	0.000542263
-0.232391389	1.11E-06
-0.050562307	0.295515072
-0.029051411	0.547975786
0.052074544	0.281288834
-0.215718596	6.38E-06
-0.079388531	0.100168079
0.212200818	9.07E-06
-0.208823105	1.27E-05
0.299838624	2.21E-10
0.462048962	3.97E-24
-0.012685298	0.793095721
-0.14893574	0.001956906
0.129399863	0.007214301
0.071603605	0.138237836
0.069197911	0.152012484
-0.117641388	0.014652992

0.108318264	0.02469058
-0.133880286	0.005425056
0.275682542	6.13E-09
-0.207819796	1.40E-05
-0.192154264	6.06E-05
-0.148855337	0.001968095
-0.114195589	0.017842469
-0.237755129	6.12E-07
-0.222045006	3.33E-06
0.028165068	0.560257706
-0.304427865	1.13E-10
-0.457519445	1.24E-23
-0.170594491	0.000380422
-0.076028417	0.11542993
0.010499887	0.828128469
-0.037024432	0.443805595
-0.028997815	0.548714673
0.37997207	3.23E-16
-0.023208655	0.631278127
-0.176158495	0.00024159
-0.154027495	0.001356154
0.054759187	0.257190889
-0.097231905	0.043888763
0.030839743	0.523606012
-0.124134098	0.009978332
0.106696606	0.026939978
-0.195828165	4.34E-05
-0.125225171	0.009338546
0.089458764	0.063828761
0.15502113	0.001260851
-0.173833208	0.000292559
-0.163550583	0.000662765
0.07781035	0.107123795
0.159014547	0.000936717
-0.175962818	0.000245536
-0.204326112	1.96E-05
-0.002901361	0.952164484
0.04477292	0.354345682
-0.120134991	0.012668473
-0.344387996	2.03E-13
-0.080285717	0.096376745
0.171389334	0.000356831
-0.056194081	0.244913779
-0.051378225	0.287780828
-0.065172963	0.177351133
0.098843819	0.040489297
-0.18593798	0.000105171
0.085964864	0.074959178
0.112622743	0.019489501
0.068753341	0.154668287
-0.148794823	0.001976555

-0.003964694	0.934666865
-0.024174105	0.617146344
-0.26118692	3.88E-08
-0.112148077	0.020011878
0.232102839	1.14E-06
0.003273089	0.946044479
0.059062867	0.22161091
-0.340788957	3.74E-13
-0.295246897	4.26E-10
0.038319023	0.428023541
0.019533497	0.686275445
0.296521135	3.55E-10
-0.357140483	2.21E-14
-0.015753764	0.744616309
-0.023869673	0.62158756
-0.005813761	0.904319325
0.445238217	2.51E-22
-0.179556759	0.000181841
0.104366214	0.03047998
-0.091458012	0.058096865
-0.016907514	0.726634319
0.061294048	0.204614587
-0.10665087	0.027005894
0.022231845	0.645714169
-0.171318266	0.000358883
0.036240025	0.453527039
-0.18511827	0.000112951
0.081523629	0.091334389
-0.12929809	0.007260458
0.059753523	0.216245358
-0.132297196	0.006005633
0.026055196	0.590017405
-0.094993142	0.049006018
0.388164261	6.51E-17
-0.130436913	0.00675872
-0.152961377	0.001465738
0.024486707	0.612600411
-0.188321242	8.53E-05
-0.039164717	0.417892086
-0.078133409	0.105669736
-0.088984029	0.065256131
-0.106830687	0.026747533
-0.157344064	0.001061596
-0.220019979	4.11E-06
-0.09154877	0.057847117
-0.048788316	0.312805703
0.130051026	0.006925141
-0.058448616	0.226462171
0.1626255	0.000711735
0.214613133	7.13E-06
0.014683837	0.761416981

-0.171292486	0.000359631
-0.09418887	0.05096224
-0.117372278	0.014882685
-0.223847958	2.76E-06
-0.049229671	0.308443118
0.479208423	4.51E-26
0.42798954	1.40E-20
0.514006999	2.28E-30
-0.07611091	0.115034577
0.231012766	1.28E-06
0.117930597	0.014409621
-0.135963065	0.004738262
-0.140754221	0.003446561
-0.000859168	0.985826942
-0.078970891	0.101972907
0.116168745	0.015949057
-0.38344007	1.65E-16
-0.248709761	1.74E-07
-0.013783302	0.77564553
-0.166294323	0.000535275
-0.204825952	1.87E-05
-0.194166728	5.05E-05
0.253376055	1.00E-07
-0.107100983	0.026363173
-0.013899032	0.773812664
-0.014279647	0.767793596
-0.133963152	0.005396106
-0.215109819	6.78E-06
-0.227509511	1.87E-06
-0.118605385	0.013855548
-0.003452111	0.943098242
-0.085951869	0.075003351
0.16465697	0.000608301
-0.058600793	0.225253324
0.020157332	0.676812294
-0.172134115	0.00033597
-0.111281174	0.020997399
-0.232771937	1.06E-06
0.122620819	0.010930016
-0.176226272	0.000240237
-0.033067811	0.494038464
-0.124982495	0.009477605
0.29238119	6.37E-10
-0.107568933	0.025708989
-0.077392655	0.109027163
-0.032836322	0.497068438
-0.052053036	0.281487862
-0.322517419	7.26E-12
-0.209574922	1.18E-05
-0.067952834	0.159538747
-0.103113324	0.032542697

-0.207580458	1.43E-05
-0.201417616	2.58E-05
0.056090235	0.245788295
0.127551845	0.008094639
-0.067627874	0.161548538
0.417373541	1.49E-19
-0.133873488	0.005427437
-0.059992008	0.21441444
-0.094764583	0.049555445
-0.083980916	0.081952104
0.028313448	0.558192399
-0.095544167	0.047702342
-0.03505607	0.468424422
-0.080607449	0.09504537
-0.061167036	0.205556009
0.156783689	0.001106811
-0.035205773	0.466525972
-0.10661849	0.027052646
-0.102578576	0.033458716
-0.100395103	0.037430473
-0.20607684	1.65E-05
0.026199228	0.587962939
0.006249369	0.897188546
0.036311959	0.452630574
0.280670903	3.17E-09
0.245611567	2.50E-07
-0.061168875	0.205542352
-0.05256295	0.276794824
-0.034891768	0.47051292
-0.135149496	0.004996611
0.139095605	0.003852259
-0.066158253	0.170876152
0.033014269	0.494738402
-0.032730217	0.498460536
-0.022667286	0.639262024
0.00949516	0.844354131
-0.044545318	0.356799862
-0.06546211	0.175432366
-0.143980493	0.002766489
-0.148646746	0.001997396
0.042006449	0.384895102
-0.161402233	0.00078164
0.013298738	0.783333193
-0.202463097	2.34E-05
0.112528361	0.019592414
0.074893895	0.120975833
-0.005903641	0.902847359
0.162008795	0.00074622
0.215627786	6.44E-06
-0.039941442	0.408712105
-0.183154536	0.000133845

-0.05626627	0.244307137
0.00399609	0.934150674
-0.059817751	0.215751157
-0.137594885	0.00425608
-0.053369206	0.26948336
-0.177323711	0.000219293
-0.185861193	0.000105878
-0.178852549	0.00019295
-0.049893841	0.301954048
-0.106384744	0.027392205
-0.155601933	0.001208038
-0.168755618	0.000440679
-0.137796786	0.004199611
-0.157003076	0.001088902
-0.113038736	0.019041496
-0.118703553	0.013776524
0.552930847	8.47E-36
-0.13361285	0.005519444
-0.14793309	0.002100687
0.081653688	0.090817124
-0.106349662	0.027443482
-0.125737663	0.009050822
-0.288743651	1.06E-09
-0.096856651	0.044713732
0.0251491	0.603016826
0.071933249	0.136427502
-0.211425641	9.80E-06
0.154996199	0.001263165
-0.091251515	0.058668431
-0.03607636	0.455570388
0.077128284	0.110245566
-0.43250242	4.99E-21
0.153956709	0.001363189
-0.123625538	0.010289617
-0.176642459	0.000232083
-0.062947904	0.192640238
-0.092226205	0.056010902
-0.084124503	0.0814289
0.013124125	0.786108671
-0.154521274	0.001307995
0.007352703	0.879165542
0.047178351	0.329061029
-0.035444386	0.463508816
-0.318877129	1.28E-11
-0.048739014	0.313295527
-0.152886784	0.001473702
0.098320979	0.041566706
0.031590759	0.51353959
-0.12388413	0.010130275
-0.051134245	0.290079161
-0.261685759	3.65E-08

0.386861171	8.42E-17
-0.132407017	0.005963626
0.425706171	2.34E-20
-0.142864503	0.002986518
-0.306366876	8.52E-11
0.016685479	0.73008361
-0.13952698	0.003742781
-0.116615709	0.015545461
0.146377741	0.002342916
-0.122750444	0.010845459
-0.069549244	0.149938255
-0.127562936	0.008089081
-0.153949723	0.001363885
0.324727708	5.13E-12
-0.245243975	2.61E-07
0.147838279	0.002114771
-0.154415971	0.001318132
-0.01167332	0.809270906
0.018628812	0.700086108
0.154589214	0.001301494
-0.163479498	0.000666414
0.134018037	0.005377008
0.254974104	8.29E-08
-0.135537583	0.004871831
-0.131663349	0.006253314
-0.056391496	0.243257296
-0.286078689	1.52E-09
-0.194044277	5.11E-05
-0.1370562	0.004410114
-0.066337921	0.169714707
-0.199610366	3.06E-05
-0.107826451	0.025354988
-0.113360251	0.018701412
-0.149427286	0.001889759
-0.23105575	1.28E-06
-0.305452239	9.75E-11
-0.275845621	6.00E-09
-0.041919707	0.385878215
-0.017673788	0.714773125
0.170707476	0.000376982
-0.060817079	0.208166137
0.108226121	0.024813903
0.164171463	0.000631669
0.177992285	0.000207384
0.378395315	4.37E-16
0.251463379	1.26E-07
-0.106628399	0.027038331
-0.263874344	2.78E-08
0.055671956	0.24933282
-0.072707627	0.132246557
-0.019059741	0.693495001

-0.036607885	0.44895318
-0.105609131	0.028545301
-0.24389976	3.05E-07
-0.076644859	0.112501214
0.183841147	0.000126158
-0.113459812	0.018597181
0.398154093	8.70E-18
-0.073096612	0.130184052
-0.027893785	0.564043234
-0.090966329	0.059465447
-0.192974872	5.63E-05
-0.118068673	0.014294686
0.097408966	0.043503978
-0.19880687	3.29E-05
0.013198168	0.784931414
-0.022304372	0.644637643
-0.23443773	8.84E-07
0.123395165	0.01043344
0.217096854	5.55E-06
-0.154382863	0.001321334
-0.127143312	0.008301728
-0.392175575	2.93E-17
-0.232017312	1.15E-06
-0.15028792	0.001777249
0.088185945	0.067714761
-0.081153729	0.092818449
-0.141683582	0.003236571
-0.06663559	0.167803444
-0.085129686	0.07784141
0.211673318	9.56E-06
-0.164869648	0.00059832
-0.249644663	1.56E-07
-0.194509879	4.90E-05
-0.152185039	0.00155059
-0.131284012	0.00640589
0.275367878	6.39E-09
-0.047197931	0.328860115
-0.236052085	7.39E-07
0.230896687	1.30E-06
-0.137020268	0.004420566
-0.062631517	0.19489034
-0.045658854	0.344894386
-0.032870378	0.496622054
-0.031463374	0.515239925
-0.055157037	0.253744976
0.179690874	0.000179794
0.284333997	1.93E-09
0.135054539	0.005027577
-0.107498162	0.025807018
-0.209051994	1.24E-05
-0.150769208	0.001717039

-0.040242878	0.405181953
-0.043446945	0.368792787
-0.295040397	4.38E-10
-0.140687153	0.003462183
-0.192515279	5.87E-05
-0.016005357	0.740682819
0.09291477	0.054194354
0.045075381	0.351100806
-0.157009471	0.001088384
-0.200967741	2.69E-05
-0.243987289	3.02E-07
0.074536573	0.122764902
-0.056910314	0.238941416
-0.007530993	0.876258761
-0.100589182	0.037061951
-0.19022149	7.21E-05
0.141009463	0.003387691
-0.185333443	0.000110858
-0.289357844	9.71E-10
0.127885709	0.007928834
0.035810005	0.458906832
-0.127326513	0.008208285
-0.031557581	0.51398217
0.059888117	0.215210665
-0.138321736	0.004055947
0.284411165	1.91E-09
0.215635537	6.43E-06
0.223801749	2.77E-06
-0.071689075	0.137766692
0.016096406	0.739260985
-0.116995681	0.015209427
-0.143001178	0.002958742
-0.080617715	0.095003129
-0.242650214	3.52E-07
-0.021259929	0.660211137
0.156557001	0.001125601
0.138510876	0.004005284
0.130765453	0.006619862
-0.032534422	0.501034798
-0.069207878	0.151953338
0.001605131	0.973524803
-0.296836477	3.40E-10
-0.144780361	0.002617978
0.012446038	0.796912259
-0.012150075	0.801639997
-0.124933035	0.009506172
-0.011770633	0.807711821
0.140620197	0.003477843
-0.024774924	0.608422192
0.097534998	0.043231821
-0.245982469	2.40E-07

-0.060090152	0.213664208
-0.028584634	0.554427294
-0.085321286	0.077172366
-0.003309853	0.94543939
-0.351965116	5.51E-14
-0.250987334	1.33E-07
0.173072669	0.000311299
-0.125262471	0.009317334
-0.290375897	8.43E-10
-0.008205212	0.865281881
0.130761868	0.006621364
-0.149365371	0.001898099
-0.24733632	2.05E-07
-0.168610243	0.000445803
-0.074662187	0.122133635
-0.09996108	0.038265867
-0.147674695	0.002139274
-0.275943627	5.93E-09
-0.234555028	8.72E-07
-0.025276902	0.60117553
0.008215282	0.865118114
0.152271343	0.00154094
0.03979713	0.410408593
-0.04797389	0.320961708
0.08315833	0.085002046
-0.005742643	0.905484263
-0.225970542	2.21E-06
-0.007812968	0.871664952
-0.135839829	0.004776605
0.00159666	0.973664468
-0.114936659	0.017109497
-0.102229309	0.0340688
-0.135770441	0.004798317
-0.146914063	0.002256651
-0.147697173	0.002135892
-0.272755515	8.98E-09
0.085455746	0.07670564
0.263038764	3.09E-08
-0.12399752	0.010061098
-0.178690666	0.000195592
0.061339297	0.204279956
-0.102322696	0.033904755
-0.246936756	2.15E-07
-0.234507529	8.77E-07
-0.081745784	0.090452265
-0.033858954	0.483757921
-0.056619951	0.241350169
-0.26684153	1.92E-08
-0.303011757	1.39E-10
-0.140868171	0.003420166
-0.048537175	0.315306096

0.18092044	0.000162011
0.127974482	0.007885261
0.289344442	9.73E-10
-0.137761483	0.004209436
-0.25218638	1.16E-07
-0.112119615	0.020043584
0.09965705	0.038860416
-0.077981132	0.106353164
-0.154373703	0.001322221
-0.040199577	0.405687939
-0.125123076	0.009396827
-0.081705638	0.090611171
-0.025378919	0.599707559
-0.141048588	0.003378748
-0.102711724	0.033228603
-0.067289897	0.16365898
-0.136524158	0.004567173
0.00437949	0.927849273
0.037712105	0.435381448
-0.218797223	4.66E-06
-0.104495975	0.030272885
-0.257662157	5.99E-08
-0.249323308	1.62E-07
-0.067630797	0.161530377
-0.123482321	0.01037882
0.231220899	1.26E-06
-0.134348402	0.005263331
-0.072857372	0.131449598
-0.234956372	8.35E-07
-0.17275459	0.000319464
-0.135525714	0.004875605
-0.109778609	0.022805569
-0.26284809	3.16E-08
-0.088771183	0.065904553
0.122710887	0.010871201
-0.10125374	0.035823301
-0.207783429	1.40E-05
-0.222662117	3.12E-06
-0.034183128	0.479579076
-0.20079039	2.74E-05
0.046133594	0.339896335
-0.071116158	0.140948521
-0.092122303	0.056289352
-0.19600221	4.27E-05
-0.059905844	0.215074657
0.160902361	0.000811992
0.225750437	2.26E-06
-0.223433577	2.88E-06
-0.03202537	0.507760384
0.471895934	3.14E-25
-0.206850324	1.53E-05

-0.131063473	0.006496121
-0.239092085	5.26E-07
-0.131532833	0.006305438
-0.241320712	4.09E-07
-0.073600105	0.127551439
-0.154022832	0.001356616
-0.127133466	0.008306777
-0.145361398	0.002514692
-0.322053549	7.81E-12
0.061633753	0.202112032
-0.188105144	8.70E-05
0.023524627	0.626637937
-0.067202751	0.164206496
-0.026434068	0.584620283
0.159408008	0.000909348
0.200345771	2.85E-05
-0.005507364	0.909339607
-0.11033518	0.022120662
-0.242554434	3.56E-07
-0.08703055	0.071408202
-0.48505881	9.25E-27
0.124631747	0.009681845
0.113411044	0.018648173
0.114008522	0.01803177
-0.027954682	0.563192393
-0.019413541	0.688100794
-0.215326977	6.64E-06
-0.068483544	0.156297057
-0.289605081	9.39E-10
-0.140475327	0.003511946
-0.140586207	0.003485817
0.160086352	0.00086389
-0.103737424	0.031500769
0.179469348	0.000183186
0.204213638	1.98E-05
-0.072470392	0.133516769
0.056113898	0.245588829
-0.224582354	2.55E-06
0.033226611	0.491965634
-0.173029087	0.000312407
-0.194630895	4.84E-05
0.04291435	0.37469701
-0.123662477	0.010266721
0.0158847	0.742568372
0.201503115	2.56E-05
-0.243673488	3.13E-07
-0.031528822	0.514365968
-0.113799364	0.0182455
0.179058721	0.000189634
-0.156019032	0.001171375
-0.115299534	0.016760287

-0.242697312	3.50E-07
0.035168145	0.467002749
0.330759208	1.95E-12
-0.155965235	0.001176046
0.31797577	1.47E-11
-0.139591279	0.003726706
-0.214918503	6.91E-06
0.173196958	0.000308162
0.10506063	0.029385685
0.074993643	0.120480051
0.005142915	0.91531575
-0.277276602	4.97E-09
-0.148738737	0.001984425
0.00531231	0.912537442
-0.083267674	0.084591429
-0.281064767	3.01E-09
-0.175175454	0.000262031
0.001685676	0.972196801
-0.158521771	0.000972067
-0.197821184	3.61E-05
0.117149317	0.015075378
-0.007876313	0.870633536
-0.234761129	8.53E-07
0.164087233	0.000635806
-0.081572831	0.091138429
0.283938453	2.04E-09
0.038547867	0.42526802
0.013507404	0.780020052
-0.041596653	0.389553054
-0.265648682	2.23E-08
-0.145162387	0.002549643
-0.230170476	1.41E-06
-0.119365261	0.013254127
-0.026228849	0.587540835
-0.146412095	0.002337301
-0.125842341	0.008993033
0.151170111	0.001668319
-0.125615371	0.009118753
-0.214329732	7.34E-06
-0.126843834	0.008456524
0.006983251	0.885194077
-0.122200901	0.011207962
-0.050012659	0.300802792
-0.211823252	9.42E-06
-0.207157949	1.49E-05
-0.167260429	0.000496096
-0.063311458	0.19007825
-0.023973183	0.620075955
-0.237089811	6.59E-07
-0.099283099	0.039602408
-0.134564996	0.005189981

-0.054983353	0.255245349
0.058741785	0.224137427
-0.201562492	2.54E-05
-0.144198012	0.002725361
-0.23196725	1.16E-06
-0.13647147	0.004582999
-0.146622415	0.002303194
0.064142803	0.184313866
-0.141551127	0.003265772
0.293416794	5.51E-10
-0.132731665	0.005840985
-0.103306256	0.032217502
-0.093614681	0.052398353
-0.091018164	0.05931992
-0.093455189	0.052803191
-0.057481982	0.234248563
0.009347439	0.846745474
-0.270407507	1.22E-08
-0.111885773	0.020305738
-0.112104002	0.020060994
-0.257666324	5.98E-08
-0.145372267	0.002512796
-0.07370733	0.12699616
-0.107607451	0.02565577
-0.272079058	9.81E-09
-0.104717339	0.029922377
0.0595925	0.2174879
-0.109836534	0.022733443
0.118481081	0.013956184
-0.130669419	0.006660184
-0.012710066	0.792700914
-0.023824668	0.622245291
-0.199426048	3.11E-05
-0.138176319	0.004095292
-0.214085343	7.52E-06
-0.16970966	0.000408388
0.167083371	0.00050307
-0.120262246	0.012573881
0.090226722	0.061574234
-0.184634184	0.000117797
-0.006473065	0.893529879
-0.116008391	0.016096074
0.081508906	0.091393094
-0.181594594	0.000152975
-0.080293508	0.096344327
-0.149828832	0.001836478
-0.161960711	0.000748972
-0.314620442	2.47E-11
-0.152215174	0.001547214
-0.019971099	0.679632085
0.136827056	0.00447715

-0.081849497	0.090042795
-0.103783974	0.031424209
-0.176157978	0.0002416
0.025584038	0.59676089
0.051173196	0.289711413
-0.122228223	0.01118969
0.237071563	6.60E-07
-0.359029165	1.58E-14
-0.010688409	0.825091869
-0.111288435	0.020988973
-0.022543954	0.641086772
-0.106966606	0.026553658
-0.30483942	1.07E-10
-0.493058524	1.01E-27
0.10287348	0.032950866
-0.223710148	2.80E-06
-0.026132254	0.588917849
0.070363411	0.145214274
-0.127751684	0.007995027
0.041798255	0.38725729
-0.177470643	0.000216622
-0.138724973	0.003948627
0.106135967	0.027757616
-0.08656669	0.072936576
-0.069018043	0.153082794
0.170082563	0.000396377
-0.053070456	0.272176995
-0.255016827	8.25E-08
-0.264562524	2.55E-08
-0.132348403	0.005986013
-0.119262503	0.013334089
0.140110315	0.00359922
-0.173503164	0.000300559
-0.135129557	0.005003099
-0.120961394	0.012065221
0.204290102	1.96E-05
-0.191733718	6.29E-05
-0.127974723	0.007885143
-0.071602505	0.13824391
0.207371782	1.46E-05
-0.06475292	0.180166212
8.08E-05	0.998667262
0.257461488	6.13E-08
-0.119315946	0.013292449
-0.087369553	0.070307842
-0.030602139	0.526811675
-0.079550217	0.099476206
-0.140030157	0.003618646
-0.239458054	5.05E-07
-0.072960497	0.130902921
-0.131754321	0.006217212

-0.187434621	9.22E-05
-0.146008315	0.002404075
-0.029147692	0.54664966
-0.188327647	8.53E-05
-0.010649518	0.825718103
-0.084154524	0.081319852
-0.120718029	0.01224018
-0.099698019	0.038779846
-0.24053141	4.48E-07
-0.133605547	0.005522042
-0.081738886	0.090479553
-0.091435525	0.058158883
0.089340942	0.064180593
0.093917608	0.05163655
0.074564195	0.122625875
-0.10495272	0.029553491
-0.095265967	0.048356862
-0.043418246	0.369109459
-0.16454756	0.000613496
-0.095587535	0.047600982
-0.107508077	0.025793265
0.255062407	8.20E-08
0.066146243	0.170954001
-0.163046605	0.000689042
-0.193919945	5.17E-05
0.341873192	3.11E-13
-0.231507316	1.22E-06
0.029990952	0.535103177
0.077925246	0.106604857
-0.195381809	4.52E-05
-0.099485129	0.039200066
-0.244006507	3.01E-07
-0.075083037	0.120037083
-0.043058061	0.373098152
0.093870626	0.051754093
0.022702825	0.638736615
-0.317655924	1.55E-11
-0.112920049	0.019168392
-0.12249434	0.011013084
-0.213109842	8.29E-06
-0.025555491	0.597170601
-0.111271792	0.021008291
0.017031138	0.724716232
0.379197367	3.74E-16
0.29405194	5.04E-10
0.100225886	0.037754319
-0.098726224	0.040729544
-0.048975748	0.310948104
0.056018328	0.246395121
-0.082501984	0.087500669
-0.18489595	0.000115153

0.075331771	0.118811232
-0.052664357	0.275867887
0.037771068	0.434663449
-0.107008628	0.026493962
0.388077765	6.62E-17
-0.141482011	0.003281103
-0.017769332	0.713298935
-0.136819242	0.004479452
-0.021532215	0.656136623
-0.427270009	1.65E-20
-0.259166492	4.98E-08
0.117854272	0.014473501
-0.237513253	6.28E-07
-0.374717885	8.80E-16
-0.119017125	0.013526752
-0.094737414	0.049621097
-0.011787454	0.807442404
-0.013550298	0.779339482
0.015149475	0.75409094
-0.063485096	0.188863479
-0.293856601	5.18E-10
0.144665169	0.002638908
0.000108753	0.99820589
0.143532282	0.002853022
-0.174361373	0.000280171
0.036303007	0.452742078
-0.138655292	0.003966987
-0.269573479	1.35E-08
0.10463385	0.030054163
0.04379425	0.364973891
-0.10857746	0.024346509
-0.012029505	0.803568088
-0.356341204	2.55E-14
-0.112143468	0.020017009
0.024297213	0.615354308
-0.100619494	0.037004672
-0.009740163	0.840391151
0.067895569	0.159891544
-0.146360501	0.002345738
-0.204090744	2.00E-05
-0.170807852	0.000373951
0.106374054	0.027407821
-0.171037832	0.00036709
-0.151422206	0.001638336
-0.015818743	0.743599757
-0.034211236	0.479217663
-0.05084079	0.292859836
-0.079690262	0.098880014
-0.168094961	0.000464414
-0.011977518	0.804399807
0.104950164	0.029557476

-0.000785116	0.987048404
-0.11174236	0.020467986
-0.198732253	3.32E-05
0.197902873	3.58E-05
0.057022487	0.238015413
-0.047444399	0.326337846
-0.006719265	0.889505759
-0.056725793	0.240470181
-0.077988762	0.106318839
-0.181085266	0.000159757
-0.147665252	0.002140696
-0.128088408	0.007829654
-0.113811462	0.018233077
0.14598767	0.002407535
-0.057622806	0.23310259
-0.249514982	1.59E-07
-0.223557226	2.85E-06
-0.209551777	1.18E-05
-0.299974922	2.17E-10
0.007560885	0.875771575
-0.071065212	0.141234163
-0.12004962	0.012732283
0.036086634	0.455441967
-0.218289521	4.91E-06
-0.161022114	0.000804623
-0.178757831	0.000194492
-0.225338923	2.36E-06
-0.12470412	0.009639385
0.043223239	0.371265679
-0.17283533	0.000317373
0.01870071	0.698984838
-0.154026905	0.001356212
0.079521491	0.099598852
-0.005387175	0.911309888
-0.245027271	2.68E-07
0.297287785	3.18E-10
-0.24321159	3.30E-07
0.095840227	0.047013971
0.252745543	1.08E-07
0.135707722	0.004818019
-0.135602069	0.004851372
-0.203518352	2.11E-05
-0.178576789	0.000197471
0.193759669	5.24E-05
-0.12437131	0.009836016
-0.093088928	0.053742742
0.073041054	0.130477099
-0.133748383	0.005471427
-0.084700263	0.079358007
0.066415262	0.16921656
0.097454071	0.043406411

-0.116820398	0.015363641
-0.083495747	0.083740102
-0.193599003	5.32E-05
-0.088540803	0.066612346
0.114589537	0.017449482
-0.011052806	0.819229824
-0.165622627	0.000564188
0.139573287	0.003731198
0.21073018	1.05E-05
0.094906723	0.049213156
-0.327702031	3.19E-12
-0.126099191	0.008852623
-0.066494973	0.168704293
-0.016884129	0.726997343
-0.178750565	0.000194611
0.096949509	0.044508386
-0.092887835	0.054264482
-0.034991619	0.469243073
-0.108158949	0.024904139
-0.166762053	0.000515961
-0.233126782	1.02E-06
-0.125881994	0.008971227
0.198502213	3.39E-05
-0.115815741	0.016274269
0.053830903	0.265356496
-0.091378777	0.058315633
-0.239678885	4.93E-07
0.039358956	0.415585133
-0.155387462	0.001227298
0.017237753	0.721514329
-0.229552482	1.50E-06
-0.254753387	8.51E-08
-0.074577123	0.122560844
0.06364681	0.187737262
-0.277482906	4.84E-09
-0.093437679	0.052847795
-0.0414685	0.391016691
-0.083500371	0.083722914
-0.257514356	6.09E-08
-0.018130817	0.707731083
0.106964905	0.026556076
-0.050527528	0.295847807
0.013581557	0.778843621
-0.126988738	0.008381307
0.090127691	0.061861224
0.056323769	0.243824695
-0.112684609	0.019422299
-0.086110381	0.074465991
-0.133936123	0.005405534
0.037450844	0.438571071
-0.172415751	0.000328382

-0.059373053	0.219189498
-0.065381302	0.175967042
-0.129560576	0.007141947
-0.072931764	0.131055061
0.128340016	0.007708085
-0.017148453	0.722897615
0.514239634	2.13E-30
-0.204489817	1.93E-05
0.039658429	0.412043042
-0.124690505	0.00964736
-0.122457723	0.011037237
-0.156638956	0.001118775
0.026534909	0.583187643
-0.094294519	0.050701611
-0.204194106	1.98E-05
0.119141023	0.013429167
-0.023188916	0.631568477
-0.035768307	0.459430378
0.029286804	0.544736409
-0.203594405	2.10E-05
-0.130127682	0.006891793
-0.126453092	0.008662358
-0.146719237	0.002287646
-0.196002502	4.27E-05
-0.042898255	0.374876339
-0.066532701	0.168462238
-0.373549781	1.10E-15
-0.041341527	0.392470127
0.010754323	0.824030792
-0.147484726	0.002168054
-0.043486277	0.368359055
0.029083907	0.547528023
0.109205665	0.023529729
-0.036843218	0.44604086
-0.253776303	9.57E-08
0.068741907	0.154737054
-0.078202411	0.105361196
-0.03755311	0.43732096
0.081321298	0.092143783
0.045558798	0.345953701
-0.115757135	0.01632882
0.297189104	3.23E-10
0.162415365	0.000723316
0.517507865	7.91E-31
-0.024746927	0.608827513
-0.093696157	0.052192542
-0.21089365	1.03E-05
-0.010824244	0.822905548
-0.22874879	1.64E-06
-0.296230006	3.70E-10
-0.279826201	3.55E-09

0.014117844	0.770350672
0.064248046	0.183593426
0.0235149	0.62678057
0.06363829	0.187796475
-0.039940263	0.408725945
-0.107653709	0.025591983
0.007835189	0.871303114
0.160433696	0.000841435
0.019780677	0.682519933
-0.116864082	0.015325081
0.150405872	0.001762317
-0.025171199	0.602698259
-0.271944617	9.98E-09
-0.143733851	0.002813806
-0.019211272	0.691182771
-0.093090001	0.053739969
0.020887062	0.665807057
-0.062180489	0.198131119
-0.167503144	0.00048668
0.116582721	0.015574938
-0.146019187	0.002402255
-0.286299316	1.48E-09
-0.359971381	1.33E-14
-0.17073443	0.000376166
-0.088826155	0.065736581
-0.102297209	0.033949459
-0.119290952	0.013311909
-0.166686256	0.000519047
0.172194704	0.000334324
-0.216164621	6.10E-06
-0.175927537	0.000246254
-0.219953602	4.14E-06
-0.359511691	1.45E-14
-0.154608455	0.001299658
-0.187568374	9.12E-05
-0.130292774	0.006820458
-0.284196483	1.97E-09
0.1442653	0.002712752
-0.138297883	0.004062377
-0.188240303	8.59E-05
0.10946542	0.02319899
-0.091046919	0.059239318
0.195564026	4.45E-05
0.059960616	0.214654807
-0.122630472	0.010923699
-0.259367423	4.86E-08
-0.182839266	0.00013752
-0.287764205	1.21E-09
-0.192569965	5.84E-05
-0.006826765	0.887749563
-0.14414394	0.002735533

-0.010593549	0.826619499
-0.047580233	0.324953138
-0.036090128	0.455398287
-0.19570892	4.39E-05
-0.032044467	0.507507221
-0.120609702	0.012318775
-0.089798104	0.062824306
-0.09541964	0.047994392
-0.22971568	1.48E-06
-0.342506551	2.80E-13
-0.074210776	0.124414019
0.11797212	0.014374972
0.006094736	0.899718926
-0.001849056	0.96950329
0.07850125	0.104033164
-0.005839577	0.903896506
0.06977441	0.148620256
-0.196413583	4.11E-05
-0.077356287	0.10919414
-0.101796375	0.034838161
-0.083078513	0.085302792
-0.102840816	0.03300679
-0.037733152	0.435125079
0.022080678	0.647960349
0.158626909	0.000964423
-0.108298905	0.024716445
0.095171988	0.048579655
-0.014841439	0.758934958
-0.014218235	0.768763841
0.023754736	0.623267915
0.204189734	1.98E-05
0.086772763	0.072254323
-0.379224913	3.72E-16
-0.206204738	1.63E-05
-0.019542842	0.686133334
-0.326265104	4.02E-12
-0.104663593	0.030007157
-0.172147985	0.000335592
-0.190928476	6.77E-05
-0.096560058	0.045374956
-0.03684662	0.445998835
-0.176894141	0.000227279
0.19381942	5.21E-05
-0.130664765	0.006662144
0.191593219	6.37E-05
-0.070505505	0.144401583
-0.019361284	0.688896538
-0.023254214	0.630608166
-0.306549613	8.29E-11
0.063601301	0.188053694
-0.116857848	0.015330578

0.196144191	4.22E-05
-0.02350389	0.626942015
0.005136111	0.915427368
0.192104546	6.09E-05
-0.197438323	3.74E-05
-0.079086186	0.101472103
-0.032927234	0.495877307
-0.128706865	0.007533855
0.001471101	0.975734793
-0.068865886	0.15399266
-0.196107768	4.23E-05
-0.131975773	0.006130109
-0.320535709	9.91E-12
-0.12602384	0.008893611
-0.010933907	0.821141457
0.014359695	0.766529477
-0.057261038	0.236054533
-0.180173572	0.000172605
-0.199890307	2.98E-05
-0.155828758	0.001187971
0.169130682	0.000427711
-0.056232921	0.244587253
0.072302613	0.134420762
-0.011885273	0.80587612
-0.114696819	0.01734378
-0.155142437	0.001249648
-0.101985981	0.034499414
-0.030187813	0.53242537
-0.115369377	0.016693794
-0.053606946	0.267352887
-0.107040055	0.026449395
-0.041356233	0.392301623
0.331224319	1.81E-12
-0.102007557	0.034461044
0.063786853	0.186765968
-0.075231301	0.119305203
0.124037983	0.010036514
-0.133939746	0.005404269
-0.148560531	0.002009623
0.109492782	0.023164386
-0.199463561	3.10E-05
0.012813985	0.791045007
-0.208890144	1.26E-05
-0.500120313	1.36E-28
0.027324724	0.572023636
-0.080125261	0.097046268
-0.091836407	0.057061464
0.064059287	0.184887055
-0.100516003	0.037200541
0.029782835	0.537941422
-0.084099681	0.081519154

-0.157067858	0.001083665
-0.090786412	0.059972854
-0.19712953	3.85E-05
-0.027024341	0.576257592
0.044016373	0.362544427
-0.057381175	0.235071333
-0.168557254	0.000447684
-0.110410122	0.022029814
0.051113778	0.290272528
0.119809574	0.012913225
-0.09950578	0.039159136
-0.184955097	0.000114563
-0.076270286	0.114273768
-0.026450327	0.584389181
-0.169758421	0.000406798
0.444211265	3.21E-22
0.073864187	0.12618723
-0.191034325	6.70E-05
0.087915596	0.068564638
0.001697069	0.972008959
-0.150309592	0.001774497
0.430959708	7.11E-21
-0.036150493	0.454644195
0.425400698	2.51E-20
-0.090115341	0.061897091
0.180749025	0.000164387
-0.128435677	0.007662309
0.421917771	5.46E-20
-0.120689351	0.012260943
-0.28367109	2.12E-09
-0.217709956	5.21E-06
-0.158638406	0.000963591
-0.037131922	0.442482742
0.01368457	0.777210172
-0.194439962	4.93E-05
-0.123339411	0.010468514
0.299799278	2.22E-10
-0.198886469	3.27E-05
0.243278232	3.27E-07
-0.285798149	1.58E-09
-0.034015897	0.481732353
0.081772371	0.090347154
-0.052684552	0.275683546
-0.016647947	0.730667193
-0.276060815	5.84E-09
-0.214295852	7.36E-06
-0.192697342	5.77E-05
-0.040020829	0.407780622
-0.260939623	4.01E-08
-0.032880975	0.496483207
-0.17602645	0.000244246

0.032803128	0.497503715
-0.380817905	2.74E-16
-0.189670948	7.57E-05
-0.078596955	0.103610665
-0.307830781	6.85E-11
0.255918149	7.40E-08
0.027589968	0.568297257
-0.14954087	0.001874545
-0.172877807	0.000316278
-0.095724808	0.047281335
-0.068286383	0.157495494
-0.045480909	0.34677976
-0.035827569	0.458686394
-0.203902188	2.04E-05
-0.123892991	0.010124854
-0.028022179	0.562250065
-0.07358985	0.127604645
0.263140452	3.05E-08
-0.123226558	0.010539828
-0.156778409	0.001107246
-0.185297888	0.000111202
-0.102091923	0.034311364
-0.470358591	4.69E-25
-0.32244846	7.34E-12
-0.104309809	0.030570378
-0.065534243	0.174956105
0.02042042	0.672836511
-0.014317031	0.767203159
-0.069785795	0.148553849
-0.133837005	0.005440233
0.208608613	1.29E-05
-0.168806742	0.00043889
-0.302883101	1.42E-10
-0.310687252	4.47E-11
-0.122484073	0.011019851
-0.21881834	4.65E-06
-0.120392036	0.012478048
-0.195993389	4.27E-05
-0.022124713	0.647305706
-0.159832992	0.000880617
-0.048633385	0.314346679
0.084434213	0.08030957
-0.153238264	0.001436521
0.220041639	4.10E-06
-0.12039221	0.012477919
0.014618496	0.762446748
-0.017750222	0.713593715
0.167974562	0.000468866
-0.068993245	0.153230803
-0.118614408	0.013848269
0.039720823	0.411307322

-0.072479407	0.133468326
0.136129333	0.004686971
-0.083614732	0.08329872
-0.038829126	0.421895532
-0.08101485	0.093380599
0.172782282	0.000318746
-0.035564845	0.461989793
-0.325264455	4.71E-12
-0.020505539	0.671552139
-0.244134113	2.97E-07
-0.130672606	0.006658842
0.016777056	0.728660312
-0.222698116	3.11E-06
-0.180792367	0.000163783
0.0105052	0.828042863
-0.409655287	7.86E-19
-0.121669701	0.011568517
-0.055473588	0.251026199
-0.18223287	0.000144857
-0.104103802	0.03090249
-0.088270686	0.067450151
-0.152190256	0.001550006
-0.18360214	0.000128786
0.089007607	0.065184628
-0.049961527	0.301297862
0.040933682	0.397160675
-0.132822091	0.005807231
0.155071219	0.001256214
-0.052691598	0.275619247
-0.108948759	0.023860845
-0.055130382	0.25397484
-0.219947197	4.14E-06
0.137345465	0.004326785
0.19423494	5.02E-05
0.069603335	0.149620827
-0.078678335	0.103252474
-0.014381188	0.766190178
-0.127688083	0.00802661
-0.142230528	0.003118486
-0.032374722	0.503139675
-0.045883615	0.342522309
0.498885458	1.93E-28
-0.071666008	0.137893724
-0.188502834	8.40E-05
0.034323345	0.477777675
0.060019688	0.214202656
-0.094470936	0.050268881
-0.309419302	5.41E-11
-0.24085184	4.32E-07
-0.235465287	7.89E-07
-0.001430242	0.976408538

-0.290015251	8.87E-10
-0.143704504	0.002819485
0.099837381	0.038506831
0.05418667	0.262206223
-0.081533903	0.091293443
-0.110060911	0.022455912
-0.196237844	4.18E-05
-0.089284336	0.064350192
0.246711277	2.20E-07
-0.174492881	0.000277163
-0.067126961	0.164683782
-0.214471716	7.23E-06
0.034432861	0.476373255
-0.073831964	0.126353081
0.011980779	0.804347616
-0.052688804	0.275644742
-0.230529538	1.35E-06
-0.125053982	0.009436452
0.057414332	0.234800484
-0.084044715	0.081719298
-0.310256069	4.77E-11
-0.181517298	0.000153986
0.029338238	0.544029864
-0.037947769	0.4325158
0.193160695	5.53E-05
-0.125118857	0.009399242
0.056194816	0.244907594
0.3913281	3.47E-17
0.341281892	3.44E-13
-0.151388916	0.001642266
0.366265185	4.25E-15
-0.129696739	0.007081155
-0.086368915	0.073596286
-0.258446604	5.44E-08
0.34579692	1.60E-13
-0.041201456	0.394077268
0.085339812	0.077107925
-0.08997791	0.062297375
0.052466404	0.277679289
0.125961207	0.008927809
0.043786545	0.365058352
-0.133629514	0.00551352
-0.015062213	0.755462197
-0.081678652	0.090718108
0.044131981	0.36128397
-0.139097178	0.003851854
-0.056601155	0.24150668
-0.132274806	0.00601423
0.033681732	0.486050743
-0.074135855	0.124795679
-0.037420981	0.438936505

0.098211612	0.04179511
0.481148056	2.68E-26
0.244390644	2.88E-07
0.04860516	0.314627944
0.316867561	1.75E-11
-0.009609748	0.842500156
-0.151764729	0.001598388
0.216602517	5.83E-06
-0.250850473	1.36E-07
-0.143580435	0.002843609
0.11950559	0.013145612
-0.254494136	8.78E-08
-0.067658073	0.161360968
-0.112399048	0.019734183
-0.004671483	0.923053205
-0.183158516	0.0001338
0.03406118	0.481148773
0.175357756	0.000258122
0.095377768	0.048092931
0.058593442	0.22531161
-0.273763864	7.88E-09
-0.141613531	0.003251984
0.44250482	4.82E-22
-0.077510609	0.108486984
-0.122412768	0.011066954
-0.020145079	0.67699768
-0.103737367	0.031500863
-0.063807485	0.18662318
-0.124476037	0.009773761
-0.094417108	0.050400588
-0.306715626	8.09E-11
0.060981389	0.206937677
-0.230891811	1.30E-06
-0.070915174	0.142077972
-0.12791003	0.007916875
-0.113197342	0.018873063
-0.011232355	0.816345091
-0.090011331	0.062199837
-0.153930446	0.001365808
-0.042992887	0.373822717
-0.126371889	0.00870569
-0.274514018	7.15E-09
0.039858591	0.409685566
-0.017972771	0.710163526
-0.066076318	0.171407778
-0.132613697	0.005885285
-0.379115076	3.80E-16
-0.216060767	6.16E-06
-0.094705148	0.049699159
-0.124522703	0.009746133
0.163950485	0.000642578

-0.029836942	0.537202801
0.213622019	7.88E-06
-0.034960122	0.46964343
-0.131737842	0.006223738
-0.063995756	0.185323959
0.166227629	0.000538084
-0.206813187	1.54E-05
-0.036139101	0.454786456
-0.103907342	0.03122208
-0.112382774	0.019752088
-0.07988865	0.098040315
0.02351024	0.626848899
-0.09790146	0.042448604
-0.226060816	2.19E-06
-0.18299258	0.000135722
-0.028195846	0.559829003
-0.304803448	1.07E-10
-0.112525882	0.019595123
-0.035710341	0.46015875
-0.185167561	0.000112468
0.065674782	0.174030957
-0.257594938	6.04E-08
-0.043127848	0.372323255
0.514531874	1.95E-30
-0.098179725	0.041861903
0.24414655	2.96E-07
-0.196313313	4.15E-05
-0.213428239	8.03E-06
-0.207933145	1.38E-05
0.451034245	6.16E-23
-0.211084474	1.01E-05
-0.008925341	0.853586125
-0.12250926	0.011003256
0.019642681	0.684615604
0.215962125	6.22E-06
-0.311006418	4.26E-11
0.046906885	0.331854753
-0.048077996	0.319911496
-0.35895342	1.60E-14
-0.206422668	1.60E-05
0.256714041	6.72E-08
0.068935396	0.153576499
-0.030305744	0.530824456
-0.190872063	6.80E-05
0.166844766	0.000512614
-0.317165249	1.67E-11
-0.127608234	0.008066419
-0.255412552	7.86E-08
-0.1302299	0.006847547
-0.238629101	5.55E-07
-0.057815906	0.231537663

-0.022172317	0.646598305
0.152008494	0.001570505
-0.141316255	0.00331814
0.207595874	1.43E-05
-0.107758439	0.025448071
-0.154804499	0.001281087
-0.226459874	2.09E-06
0.013430456	0.781241342
-0.360533622	1.21E-14
0.005833131	0.904002085
0.06244786	0.196205265
-0.072725063	0.13215357
-0.278987865	3.97E-09
-0.077136855	0.110205896
-0.028528262	0.555208938
-0.151672854	0.001609015
0.215429275	6.57E-06
-0.214828238	6.98E-06
0.087791661	0.068957156
0.324842729	5.03E-12
-0.027516436	0.569329129
-0.070741354	0.143060348
-0.105511602	0.028693203
-0.33661796	7.49E-13
-0.093549621	0.05256318
-0.223930005	2.74E-06
0.185281209	0.000111363
-0.18150929	0.000154091
0.034519749	0.475260628
-0.121007582	0.012032267
0.170339638	0.000388288
-0.065372883	0.176022814
0.004270942	0.929632874
-0.101374784	0.035601521
0.406094864	1.67E-18
-0.116154138	0.0159624
-0.125430694	0.009222199
-0.091254602	0.058659851
-0.068431477	0.156612872
0.032344127	0.503543452
-0.084426647	0.080336766
-0.007574375	0.875551725
-0.007625	0.874726766
-0.142147471	0.003136162
0.156258343	0.001150806
0.451349382	5.70E-23
-0.038984406	0.420040332
-0.047215665	0.328678218
-0.0299088	0.536222639
-0.087777648	0.069001653
-0.215235165	6.70E-06

-0.009169641	0.849625554
-0.145930007	0.002417225
-0.15198752	0.001572887
-0.356291193	2.57E-14
-0.125319677	0.009284885
-0.18302032	0.000135399
-0.020005241	0.679114791
-0.058809714	0.223601208
0.063398398	0.189469301
-0.145941233	0.002415336
-0.317202694	1.66E-11
0.023813252	0.622412188
-0.128065368	0.007840872
-0.091769492	0.057243441
-0.142087564	0.003148968
0.16858516	0.000446692
-0.111186464	0.021107581
-0.213393618	8.06E-06
-0.359896177	1.35E-14
-0.063657778	0.187661055
-0.151497197	0.001629512
-0.11067747	0.021708358
-0.067408207	0.162917868
-0.036125505	0.454956267
-0.023928941	0.620721847
0.082846928	0.086180249
0.092495999	0.055293211
-0.110085075	0.022426199
-0.087972698	0.068384407
-0.139275285	0.00380631
-0.146622357	0.002303204
-0.015135225	0.75431481
-0.032012159	0.507935546
-0.112964521	0.019120758
0.076997636	0.110851625
-0.045559036	0.345951174
-0.314741062	2.42E-11
-0.073867053	0.126172485
-0.050238418	0.298623396
-0.099440332	0.039288979
-0.100719479	0.036816269
0.039309731	0.416169067
0.116408817	0.015731152
-0.105876611	0.02814302
0.309786011	5.12E-11
0.033880138	0.483484234
0.060725215	0.208855242
-0.0234712	0.627421512
0.003730895	0.938511726
-0.009647002	0.84189759
-0.08823896	0.067549118

-0.166019356	0.000546941
-0.055948654	0.246984096
0.21712521	5.53E-06
-0.14106379	0.003375279
-0.030303596	0.530853586
0.185370597	0.000110501
-0.097522856	0.043257977
-0.13059476	0.006691683
-0.137296804	0.004340703
0.07633028	0.113988399
-0.124275424	0.009893325
-0.134197032	0.005315146
0.022591527	0.640382661
0.026660846	0.581400762
0.130881831	0.006571292
-0.152746876	0.001488746
0.289880414	9.03E-10
-0.124870998	0.009542111
-0.179571511	0.000181615
-0.193766986	5.24E-05
-0.272811963	8.92E-09
0.030380469	0.529811331
-0.094788632	0.049497392
-0.0584876	0.226152049
0.240861662	4.31E-07
-0.035009439	0.469016644
-0.107103598	0.026359477
-0.085649116	0.076038456
-0.143730276	0.002814497
-0.021976831	0.649505257
0.099049781	0.0400714
-0.225252177	2.38E-06
0.058525104	0.225853998
-0.093119035	0.05366499
-0.121864363	0.011435216
-0.117413197	0.014847557
-0.136395565	0.004605884
-0.060574051	0.209992774
0.074253238	0.124198114
-0.145774316	0.002443564
-0.084010095	0.081845563
0.077277723	0.109555538
-0.063708147	0.187311389
-0.077587093	0.108137852
0.035770321	0.459405082
-0.041069398	0.395596104
-0.106464574	0.027275827
-0.032307031	0.504033255
0.052228955	0.279862738
-0.107697852	0.025531239
-0.069544405	0.149966677

0.204242206	1.97E-05
-0.107803077	0.025386946
-0.069349737	0.151113464
-0.198061726	3.53E-05
-0.06357213	0.188256737
-0.051006411	0.291288288
-0.132895649	0.005779903
0.103025348	0.032691911
-0.169451164	0.000416913
-0.211408828	9.82E-06
0.171993641	0.000339815
0.052322007	0.279005698
-0.155792152	0.001191189
-0.13432867	0.00527006
-0.127863187	0.007939923
-0.125163549	0.009373684
-0.447451444	1.47E-22
-0.029062952	0.547816744
-0.025373039	0.59979212
-0.069921686	0.147762968
0.083675191	0.083075163
0.223533483	2.85E-06
-0.190443027	7.07E-05
-0.121767841	0.011501141
-0.061119829	0.205906709
-0.109466994	0.023196999
-0.110600251	0.021800784
-0.140795057	0.003437081
-0.006721373	0.889471317
-0.225024089	2.44E-06
0.098134952	0.041955838
-0.202417351	2.35E-05
0.013312218	0.783119043
0.548964527	3.27E-35
0.059560686	0.217733999
0.019732531	0.683250832
-0.210770171	1.05E-05
-0.026265219	0.587022753
-0.17981635	0.000177899
-0.177025392	0.00022481
